# Optimizing genomic medicine in epilepsy through a gene-customized approach to missense variant interpretation

**DOI:** 10.1101/gr.226589.117

**Published:** 2017-10

**Authors:** Joshua Traynelis, Michael Silk, Quanli Wang, Samuel F. Berkovic, Liping Liu, David B. Ascher, David J. Balding, Slavé Petrovski

**Affiliations:** 1 Department of Medicine, The University of Melbourne, Austin Health and Royal Melbourne Hospital, Melbourne, Victoria 3010, Australia;; 2 Simcere Diagnostics, Nanjing, 210042, China;; 3 Epilepsy Research Centre, Department of Medicine, University of Melbourne, Austin Health, Heidelberg, Victoria 3084, Australia;; 4 Department of Mathematics, North Carolina A&T State University, Greensboro, North Carolina 27411, USA;; 5 Department of Biochemistry and Molecular Biology, The University of Melbourne, Parkville, Victoria 3010, Australia;; 6 Centre for Systems Genomics, School of BioSciences and School of Mathematics and Statistics, The University of Melbourne, Parkville, Victoria 3010, Australia

## Abstract

Gene panel and exome sequencing have revealed a high rate of molecular diagnoses among diseases where the genetic architecture has proven suitable for sequencing approaches, with a large number of distinct and highly penetrant causal variants identified among a growing list of disease genes. The challenge is, given the DNA sequence of a new patient, to distinguish disease-causing from benign variants. Large samples of human standing variation data highlight regional variation in the tolerance to missense variation within the protein-coding sequence of genes. This information is not well captured by existing bioinformatic tools, but is effective in improving variant interpretation. To address this limitation in existing tools, we introduce the missense tolerance ratio (MTR), which summarizes available human standing variation data within genes to encapsulate population level genetic variation. We find that patient-ascertained pathogenic variants preferentially cluster in low MTR regions (*P* < 0.005) of well-informed genes. By evaluating 20 publicly available predictive tools across genes linked to epilepsy, we also highlight the importance of understanding the empirical null distribution of existing prediction tools, as these vary across genes. Subsequently integrating the MTR with the empirically selected bioinformatic tools in a gene-specific approach demonstrates a clear improvement in the ability to predict pathogenic missense variants from background missense variation in disease genes. Among an independent test sample of case and control missense variants, case variants (0.83 median score) consistently achieve higher pathogenicity prediction probabilities than control variants (0.02 median score; Mann-Whitney *U* test, *P* < 1 × 10^−16^). We focus on the application to epilepsy genes; however, the framework is applicable to disease genes beyond epilepsy.

Over the past decade, the rapid progress in genomic technologies has made research and clinical sequencing increasingly accessible. Exome and gene-panel sequencing data are routinely generated and available to researchers, treating physicians, and patient families. Occasionally, the clinical reports are definitive. Currently, however, variants of uncertain significance remain the overwhelming majority class of variants described in genetic reports (Richards et al. 2015). This is especially true in cases in which parental sequence is unavailable for interpretation (Lee et al. 2014; Farwell et al. 2015). Moreover, once the reports are shared the patient's physician, patient and family may generate their own impressions over the relevance of uncertain findings in known disease genes (Fogel 2011).

Two of the more reliable clues to pathogenicity are when the precise variant has been previously reported in unrelated cases (a recurring pathogenic mutation), or when the accompanying segregation data includes confirmation that the variant arose de novo (germline/mosaic) in the affected child of unaffected (or mosaic carrier) parents (Richards et al. 2015). For the latter, although identifying a de novo epilepsy gene variant in an epilepsy-ascertained patient carries great weight in variant interpretation, the background mutation rate in every gene (including disease genes) implies that some de novo mutations in disease genes have no clinical relevance. Regarding the recurring mutations, variant catalogs such as ClinVar (Landrum et al. 2016) and HGMD (Stenson et al. 2003) have proven invaluable in guiding variant classifications among known disease genes. In the absence of these data, it is difficult to interpret a novel missense variant. Currently, the final variant interpretation process often reflects a cumulative assessment of different sources of evidence, with the clinical geneticist or genetic counselor's previous experience influencing how much weight they choose to assign to each source. As a result, differing knowledge bases between individuals interpreting the genetic data can make this process highly subjective, which can contribute to the variability reported in variant classification (Amendola et al. 2015, 2016; Rehm et al. 2015). Efforts from the American College of Medical Genetics and Genomics (ACMG) and others have drastically reduced variability in classifications by systematizing the variant interpretation process (MacArthur et al. 2014; Richards et al. 2015). Yet, one of the major current tasks in medical genomics remains the reduction of the rate of Variants of Uncertain Significance (VUS) by better predicting disease potential of novel variants found in known disease genes. This has important implications for the integrity of precision diagnostics with the rapid emergence of precision medicine opportunities, particularly in the epilepsies (EpiPM Consortium 2015).

With little promising diagnostic data emerging from large-scale common variant studies in epilepsy (International League Against Epilepsy Consortium on Complex Epilepsies 2014), next-generation sequencing has achieved high rates of molecular diagnoses for epilepsy patients on the basis of a single clinically relevant dominant mutation in a growing list of epilepsy genes, each with a growing allelic series (Epi4K Consortium and Epilepsy Phenome/Genome Project 2013, 2017; EuroEPINOMICS-RES Consortium et al. 2014). Achieving precision diagnostics is important for precision medicine trials in epilepsies as they will frequently be restricted in sample size; thus, selection of the appropriate patients for enrollment in these trials will benefit from the continuous refinement of tools that facilitate accurate classification of pathogenic variants.

The increased sequencing of large and diverse populations has also enhanced our understanding of the patterns typical of standing variation in individual genes (Petrovski et al. 2013; Samocha et al. 2014; Lek et al. 2016). Public resources of human standing variation data continue to grow and have proven invaluable in modern-day interpretation of individual variants found among Mendelian disease genes, including the epilepsies (Minikel et al. 2016; Epi4K Consortium and Epilepsy Phenome/Genome Project 2017; Kobayashi et al. 2017; Walsh et al. 2017). In this study, we take 11 dominant-acting epilepsy genes, seek subregions that are intolerant to missense variation and measure the concentration of pathogenic classified variants within these missense-intolerant regions. We also assess the utility of various publicly available bioinformatic tools to find those that best discriminate the pathogenic missense variants from the empirical (background missense variant) null distribution in a gene. Finally, we combine the population genetic and gene-specific bioinformatic features to show that we can improve interpretation of novel missense variants.

## Results

### Pathogenic and background missense variants among epilepsy genes

We selected 11 dominant epilepsy genes (EpiPM Consortium 2015) of which each had at least 20 epilepsy-associated “pathogenic” missense variants among the combination of ClinVar (accessed May 1st 2016) and HGMD (hgmd2016.3) variant databases (Supplemental Table S1): *CDKL5, GRIN2A, KCNQ2, KCNT1, LGI1, PCDH19*, *SCN1A, SCN2A, SCN8A, SLC2A1,* and *STXBP1*.

Our systematic screen of case-ascertained variants in these 11 genes accompanied by individual review of 1043 pathogenic-reported missense variants resulted in a set of 606 qualifying case-ascertained pathogenic variants (Methods; Supplemental Tables S1, S2; Supplemental Data S1). In this study, the criteria adopted for qualifying genetic support were that either the variant had been reported to have arisen de novo (germline or somatic) or the variant had been reported as pathogenic in a pedigree in which the variant was present among all (and more than three) affected family members that were genotyped and was not present in more than one of the reported unaffected family members. This additional requirement of segregation evidence was performed to refine the list of pathogenic reported variants to a subset that is enriched for clinically relevant missense variants (Supplemental Data S1). We acknowledge that some of the 606 qualified missense variants in the 11 studied epilepsy genes may not be clinically relevant.

Although population reference cohorts can influence whether a variant was reported as pathogenic in ClinVar and HGMD, we did not directly use presence or frequency of an allele in the population reference cohorts to qualify pathogenic-reported variants. Thus, we could evaluate the differences in population frequencies of the 606 variants that we designated as qualified, versus the 437 unqualified missense variants. Comparing against population reference cohorts—ExAC v1, ExAC v2, ESP6500SI, the 1000 Genomes Project, and gnomAD—we find that nine (1.5%) of the 606 qualified variants were observed at least once in the population reference cohorts, with allelic count of at most two; among the 437 unqualified variants, we observe a significantly higher rate of 44 variants (10.1%) reported at least once (Fisher's exact test *P* = 5 × 10^−10^), with allelic count ranging up to 351 (Supplemental Data S2). Only one of the 606 qualified variants was observed more than once among the 277,264 chromosomes in the ExAC v2 and gnomAD data set, compared with 28 of the 437 unqualified variants (0.2% versus 6.4%; Fisher's exact test *P* = 3 × 10^−10^) (Supplemental Data S2).

To identify a benign-enriched set of missense variants within the 11 epilepsy genes, the ExAC reference cohorts were used to define three mutually exclusive groups of presumed benign missense variants. We considered only variants passing the quality control checks imposed by the database creators. For the first group of presumed benign missense variants (Control Group 1), we took singleton missense variants reported exactly once in the ExAC v1 reference sample of 60,706 individuals (release 0.3.1) (Lek et al. 2016). For the second group (Control Group 2), we took singleton missense variants reported in the approximately twofold larger ExAC v2 and gnomAD reference sample of 138,632 individuals (release 2.0) restricting to variants that were not reported in the preceding ExAC v1 release 0.3.1. A third group of presumed benign missense variants (Control Group 3) was created using variants present at a consistently low minor allele frequency (MAF) <0.05% across each of the seven ancestral groups reported in the combined ExAC v2 and gnomAD data, excluding missense variants reported in Control Groups 1 and 2 (Methods).

### Identifying missense-intolerant subregions of genes

We previously introduced a regression framework to quantify the extent to which protein-coding genes are tolerant to common functional variation in the human population. This score, known as the residual variation intolerance score (RVIS), is currently among the most widely used measures of human-lineage purifying selection at the gene level (Petrovski et al. 2013). We and others have shown that epilepsy genes are, in general, highly intolerant (Petrovski et al. 2013; Samocha et al. 2014). Important to interpreting variation within disease genes, we and others have subsequently showed that clinically relevant missense variants are more likely to occur within the more intolerant exons or conserved domains database (CDD) structures of disease-associated genes (Gussow et al. 2016; Amr et al. 2017). More recently, we have demonstrated that identifying subregions of disease-associated genes that are less (or more) tolerant to missense variation can improve interpretation of newly identified missense variants (Swanger et al. 2016; Ogden et al. 2017).

In this study, we adopt a heuristic sliding window approach to identify missense-intolerant regions independent of known gene structures. This Missense Tolerance Ratio (MTR) heuristic compares the observed proportion of missense variation to the expected proportion given the sequence context of the protein-coding region of interest, and has an important advantage of being sensitive to variability within gene structures (Methods). The MTR is calculated for windows of 31 codons, reflecting 93 protein-coding nucleotides (Methods). We previously demonstrated the research utility of this approach after applying it to three glutamate receptor genes *GRIN1*, *GRIN2A*, and *GRIN2B* (Ogden et al. 2017). Here, we report the MTR plots for the 11 epilepsy genes using the ExAC v1 (release 0.3.1) and subsequently, the more recent ExAC v2 combined with gnomAD data (release 2.0) (Lek et al. 2016). We supplement each window's MTR estimate with a binomial exact test for deviation from MTR = 1 adjusting for the study-wide false discovery rate using the Benjamini-Hochberg procedure (Supplemental Data S3). Our MTR viewer is publicly available and supports more than 85,000 distinct Ensembl transcripts spanning more than 18,000 distinct HGNC gene symbols (http://mtr-viewer.mdhs.unimelb.edu.au).

**Figure 1.**
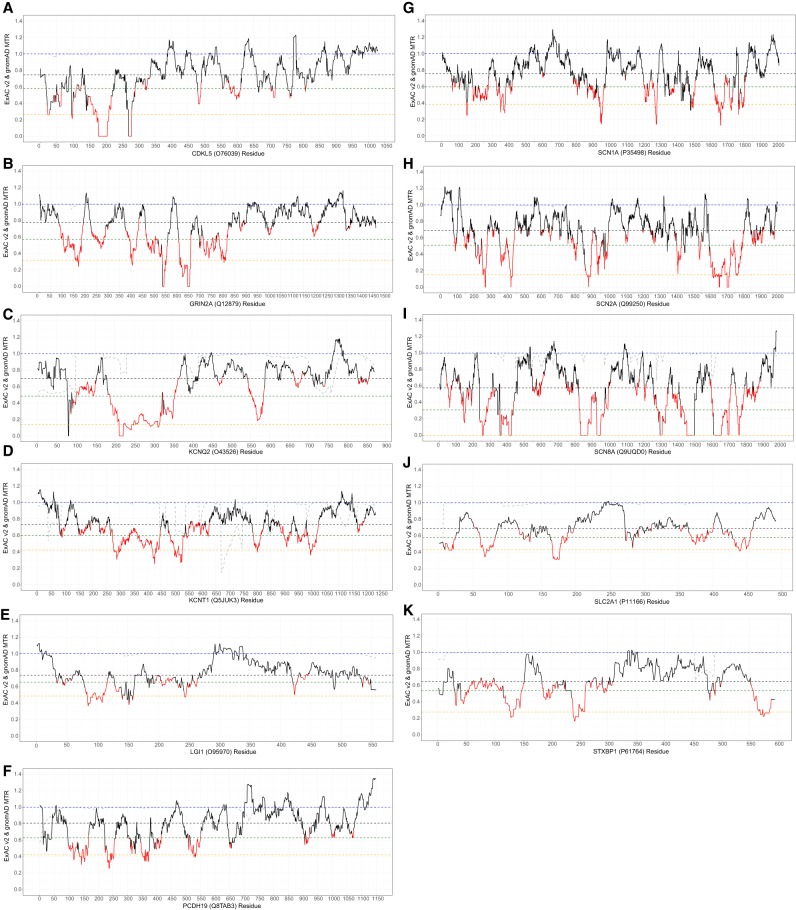
ExAC v2 MTR plots for the 11 epilepsy genes: (*A*) *CDKL5*; (*B*) *GRIN2A*; (*C*) *KCNQ2*; (*D*) *KCNT1*; (*E*) *LGI1*; (*F*) *PCHD19*; (*G*) *SCN1A*; (*H*) *SCN2A*; (*I*) *SCN8A*; (*J*) *SLC2A1*; and (*K*) *STXBP1*. Regions in red achieved a study-wide FDR < 0.05 (Supplemental Data S3). MTR = 1 is depicted by the dashed blue line. Multiple gene-specific estimates are also depicted, including a gene's median MTR (black dashed line), 25th percentile MTR (dark green dashed line), and 5th percentile lowest MTR estimates (orange dashed line). The gray dashed line reflects how well that region of the gene was covered in the ExAC v2 sample data by showing the proportion of all ExAC v2 samples that achieved at least 10-fold coverage at the sites relevant to that codon (Methods).

By not focusing on known gene structures, the MTR sliding window can represent how purifying selection has shaped a gene's landscape of human-lineage missense intolerance (Fig. 1). Each codon is assigned an estimate of missense intolerance based on the preferential depletion of missense variation at that codon and 15 flanking codons on either side. For practical purposes, we can also define gene-specific thresholds of missense depletion corresponding to, for example, the 5th percentile most missense depleted regions in a specific gene (dashed orange lines) or the 25th percentile most missense depleted (dashed dark green lines) (Methods). Supplemental Figure S1 shows MTR plots in the context of the distribution of pathogenic (qualified and unqualified) missense variants.

Figure 1 presents the ExAC v2 data as this is the largest single sample of standing variation currently available. However, we also generated MTR sliding windows restricted to the ExAC v1 data (Supplemental Fig. S2; Supplemental Data S3). To be able to use the novel genetic variation observed exclusively in the ExAC v2 sample as background missense variation, all subsequent case versus control enrichment comparisons rely on ExAC v1 (release 0.3.1) MTR estimates.

### Evaluating the predictive utility of the MTR estimates among epilepsy genes

**Figure 2.**
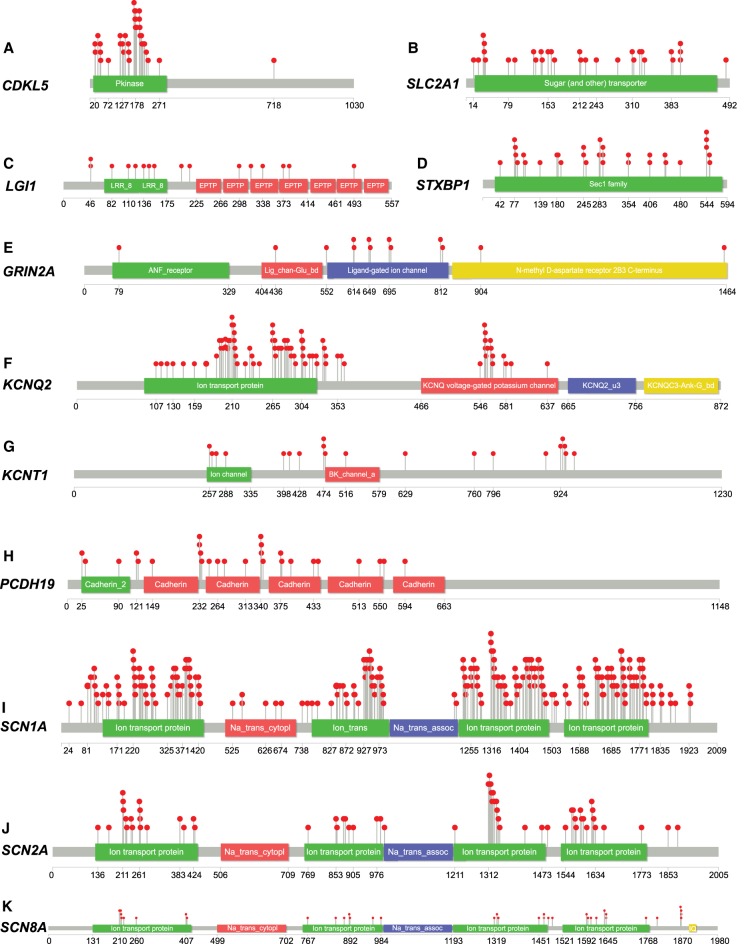
The distribution of the 606 qualified pathogenic variants (red circles) among the 11 epilepsy genes. The distribution of the 606 qualified pathogenic variants across genes: (*A*) *CDKL5*; (*B*) *SLC2A1*; (*C*) *LGI1*; (*D*) *STXBP1*; (*E*) *GRIN2A*; (*F*) *KCNQ2*; (*G*) *KCNT1*; (*H*) *PCDH19*; (*I*) *SCN1A*; (*J*) *SCN2A*; and (*K*) *SCN8A*.

An aspect that often influences variant interpretation is the proximity of a novel patient-ascertained variant to previously reported pathogenic variants. Here, we use lollipops-v.1.3.1 (Jay and Brouwer 2016) to plot the distribution of the 606 qualified pathogenic variants across the linear gene structure of the 11 epilepsy genes (Methods; Fig. 2).

To verify the predictive utility of standing variation data for epilepsy risk, we performed two analyses. First, using the ExAC v2 MTR estimates (Fig. 1; Supplemental Data S3), we adopt a binomial exact test to determine whether the 606 qualified pathogenic missense variants preferentially occur within the most intolerant 25th percentile of a gene's MTR distribution (Supplemental Data S3). We find current evidence for this preferential enrichment among six epilepsy genes: *CDKL5* (*P* = 1.1 × 10^−12^), *KCNQ2* (*P* = 1.1 × 10^−23^), *PCDH19* (*P* = 0.003), *SCN1A* (*P* = 9.7 × 10^−9^), *SCN2A* (*P* = 1.9 × 10^−4^), and *SCN8A* (*P* = 3.5 × 10^−4^) (Table 1). Although additional genes showed an elevated rate of pathogenic variants in the lower quartile of MTR, the limited number of pathogenic variants makes this test insufficiently powered to achieve statistical significance (Table 1). Larger catalogs of pathogenic alleles will clarify the predictive utility of MTR in such genes. For other genes, it is possible that critical codons may not be sufficiently concentrated in subregions of a gene to enable current 31-codon window size to be sensitive to smaller critical regions (Supplemental Fig. S1). Larger catalogs of human standing variation will enable smaller MTR windows.

**Table 1.**
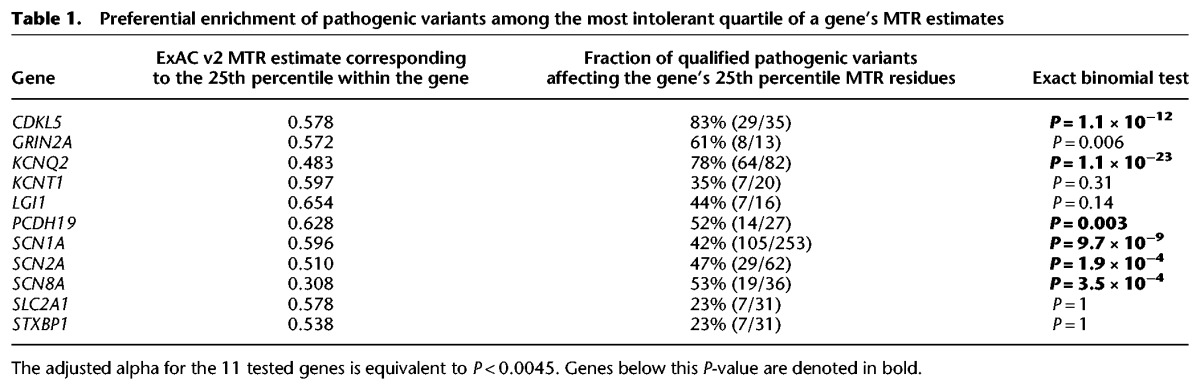
Preferential enrichment of pathogenic variants among the most intolerant quartile of a gene's MTR estimates

**Figure 3.**
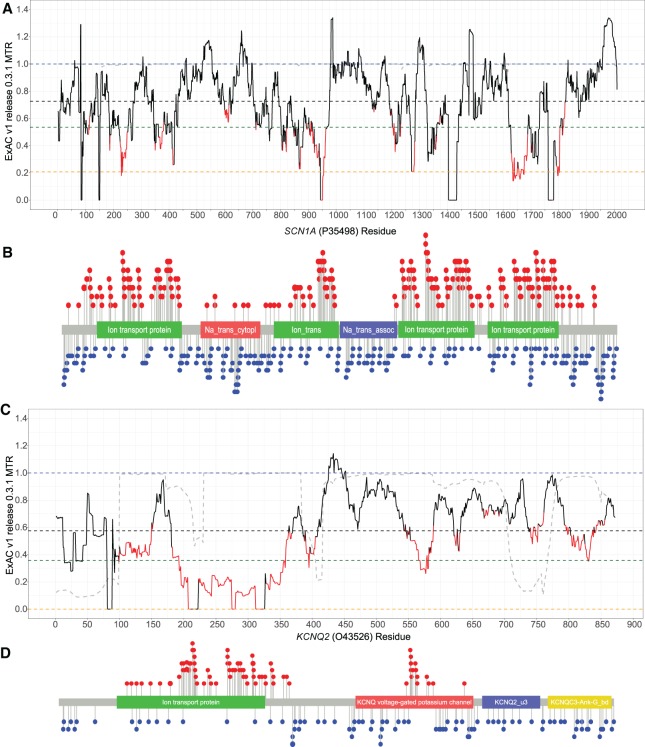
ExAC v1 MTR plot with case and control missense variant distributions. The ExAC v1 MTR plots with the case-ascertained qualified pathogenic (red circles) and ExAC v2 Control Group 2 benign (blue circles) missense variants across epilepsy genes *SCN1A* (*A*,*B*) and *KCNQ2* (*C*,*D*).

Next, we use the MTR estimates generated from the smaller ExAC v1 (release 0.3.1) data (Supplemental Data S3) to perform an empirical test comparing the 606 pathogenic missense variants and the 1377 Control Group 2 variants (Methods; Supplemental Fig. S3). This case-control design does not depend on a defined threshold of intolerance. Despite using the less informative ExAC v1 MTR estimates in this particular evaluation, the data continue to show significant evidence for pathogenic missense variants preferentially affecting codons with lower MTRs for six of the most informed epilepsy genes (Table 2). We illustrate this comparison using *SCN1A* and *KCNQ2* examples, the genes with the longest list of pathogenic missense variants available in this study (Fig. 3).

**Table 2.**
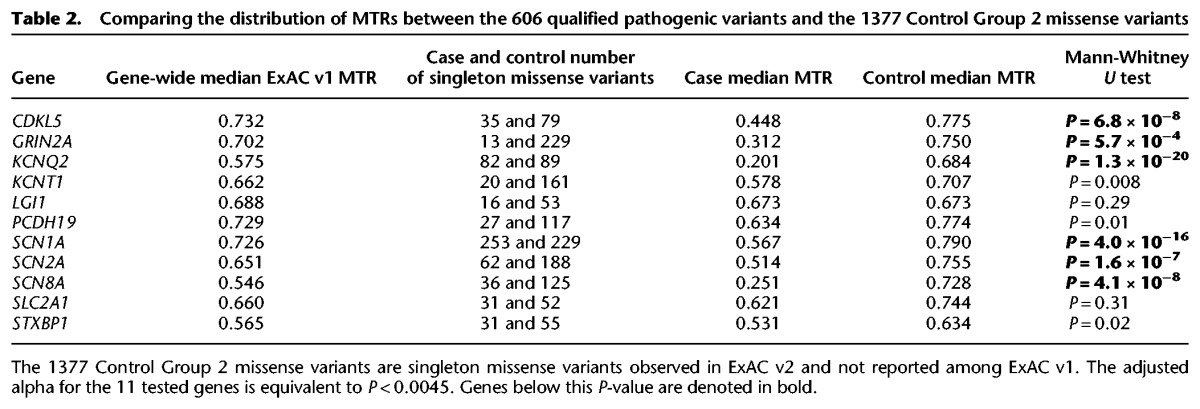
Comparing the distribution of MTRs between the 606 qualified pathogenic variants and the 1377 Control Group 2 missense variants

### Evaluating the predictive utility of variant-level bioinformatic tools

Our next strategy in this framework was to evaluate existing bioinformatic tools that help predict the deleteriousness of specific missense variants (Cooper and Shendure 2011), ranging from legacy tools such as GERP++ (Davydov et al. 2010), PolyPhen-2 (Adzhubei et al. 2013), and SIFT (Ng and Henikoff 2003), to more contemporary ensemble-based tools such as CADD (Kircher et al. 2014). Although these tools frequently capture similar information, they can also detect various signals of deleteriousness that might not be as apparent when the tool's performance is evaluated based on the entire exome. Here, we determine the score distribution (based on all possible missense variants in a gene) and the empirical null distribution (based on a control variant sample in a gene) for each bioinformatic tool in each of the 11 genes. We use these data to evaluate which tools best discriminate the 606 qualified pathogenic variants from the 1514 ExAC v1 Control Group 1 background variants within each of our studied epilepsy genes (Methods; Supplemental Data S4).

**Figure 4.**
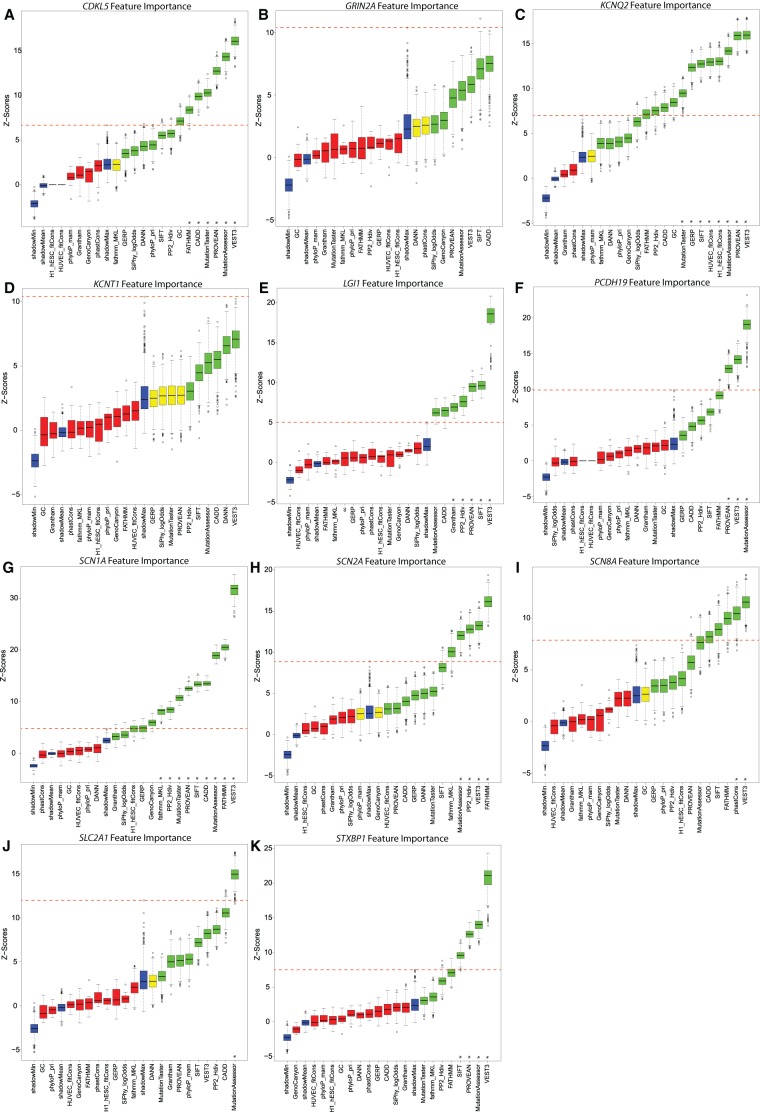
Boruta feature evaluations: (*A*) *CDKL5*; (*B*) *GRIN2A*; (*C*) *KCNQ2*; (*D*) *KCNT1*; (*E*) *LGI1*; (*F*) *PCHD19*; (*G*) *SCN1A*; (*H*) *SCN2A*; (*I*) *SCN8A*; (*J*) *SLC2A1*; and (*K*) *STXBP1*. Blue box plots correspond to minimal, average, and maximum *Z*-score of a shadow feature. Red, yellow, and green box plots represent *Z*-scores of uninformative, inconclusive, and informative features, respectively. (*) Indicates the “highly informative” features for which the minimum nonoutlier random forest *Z*-score exceeded the maximum random forest *Z*-score of the best performing randomized shadow feature (red dashed line).

We began with 31 tools (herein referred to as features) and used them to score all 88,865 possible missense variants that span the 11 epilepsy genes of interest (Supplemental Data S4). These features were primarily accessed through dbNSFP version 3.2c, and to standardize scores within a [0–1] range, we adopted the dbNSFP rank scores (Methods; Supplemental Table S4). We assessed the variance of each feature and the pairwise correlations between the features to prune the list to a subset of 20 features with absolute pairwise correlations |*r*| < 0.75 (Methods; Supplemental Fig. S4). We then evaluated the importance of the 20 bioinformatic features by adopting a permutation-based Boruta algorithm (Kursa and Rudnicki 2010). We used the Boruta algorithm to ensure that all features were independently evaluated for their ability to discriminate pathogenic variants from the ExAC v1 singleton (background) variants (Methods). The Boruta algorithm uses a random forest approach to compare the *Z*-scores of the original features with *Z*-scores of shadow features created for each original feature by randomly shuffling the information within the original features. Features that do not perform as well as the randomized features are considered uninformative (Fig. 4, red box plots). We define a “highly informative” category representing features for which the minimum (excluding outliers) random forest *Z*-score of an original feature outperforms all permutations (including outliers) of the best performing randomized feature for that gene (Fig. 4, dashed red line).

Nine of the 11 genes revealed at least one highly informative feature (Fig. 4; Supplemental Table S5). Although *GRIN2A* and *KCNT1* highlight multiple informative features (Fig. 4, green box plots), no single feature met the highly informative criteria using our current sample. Larger catalogs of pathogenic variants will enable some features to achieve this in future iterations. Through this process, we also identified three features that consistently excelled in discriminating pathogenic from background variants among the studied epilepsy genes: VEST 3.0 (eight genes) (Carter et al. 2013), MutationAssessor (seven genes) (Reva et al. 2011), and PROVEAN (six genes) (Choi and Chan 2015) were consistently highly informative (Fig. 4; Supplemental Table S5). Recently, VEST 3.0 was found to be the top-performing individual tool in Ioannidis et al. (2016). Although features can share information, by design, the Boruta permutation evaluates each feature's importance independent of signals that might be shared with other features (Methods).

Generating the annotations from 31 bioinformatic features also provided an opportunity to evaluate whether existing bioinformatic tools correlate highly with the new information captured by the empirical depletion of missense variation surrounding a variant, namely the MTR estimates. We found that VEST 3.0, a machine learning method that predicts the functional significance of missense mutations (Carter et al. 2013), and Condel, a feature using a consensus deleteriousness score combining tools (González-Pérez and López-Bigas 2011), achieved the highest absolute correlation with the ExAC v1 MTR estimates, Pearson's |*r*| = 0.27. Closely following were CADD v1.3 (Kircher et al. 2014) and PolyPhen-2 (Adzhubei et al. 2013), achieving Pearson's |*r*| of 0.26 and 0.25, respectively (Supplemental Fig. S4).

### Validating the top performing bioinformatic tools

To evaluate the predictive utility of features in an individual gene, we compared each feature's score distribution between the mutually exclusive Group 1, Group 2, and Group 3 control variants and the 606 qualified and the 437 unqualified pathogenic variants (Supplemental Fig. S5). Among the highest ranking features, we continued to observe significant differences in rank score distributions (Mann-Whitney *U* test, *P* < 0.001) when comparing the 437 unqualified pathogenic variants with the 1377 Group 2 Control variants (ExAC v2 singletons absent among ExAC v1) that were not used in the Boruta assessments. As a negative control, we found no significant difference for any feature when comparing Control Groups 1 and 2 (*P* > 0.05) (Supplemental Table S6).

Supplemental Figure S5 comprehensively demonstrates how tools can dramatically vary in their score distributions across different genes. This stresses the value of interpreting a novel variant's score relative to the empirical null distribution of that tool in that gene. By placing a novel missense variant in the context of these gene-specific score distributions, we can also assess how often we expect to come across a variant of similar score in that gene based on a sample of presumed benign background variants sampled from population reference cohorts. Although important to interpreting the scores generated by predictive tools, the empirical null distribution of a tool is rarely discussed when tools are cited as support for variants.

### Evaluating the predictive utility of a gene-specific pathogenicity prediction model

So far we have introduced mutation tolerance as a predictor of pathogenicity and assessed the predictive utility of a range of available bioinformatic tools. Next, we explore creating a multivariate logistic regression model for each gene to evaluate whether leveraging information from multiple features can improve predictions of pathogenicity. For the epilepsy genes where ExAC v1 MTR achieved a *P* < 0.0045 in Table 1, the ExAC v1 MTR was included in the model. We then used forward–backward selection to add to the model the top ranking bioinformatic features (Supplemental Table S5) for the gene while estimating the Akaike information criterion (AIC) for each model. Comparing the AIC across models, the addition of features was halted when no subsequent model was likely (*P* > 0.05) to reduce information loss compared to the previous model (Methods; Supplemental Table S7). Notably, the features in the final model do not include all features observed as highly informative based on the Boruta assessments. In the Boruta feature evaluations, each feature was assessed independent of other features; however, in the model building process, certain informative features may not be necessary due to correlation in their information.

For five epilepsy genes *GRIN2A* (CADD), *KCNT1* (VEST3), *LGI1* (VEST3), *SLC2A1* (MutationAssessor), and *STXBP1* (VEST3), there was no evidence in the current sample to support the addition of features beyond the gene's top ranked feature (Supplemental Table S5). With the exception of *SLC2A1*, these genes were the least informed in our current study—containing the smallest sample of pathogenic missense variants (Supplemental Table S2). Our ability to assess these genes will improve with larger catalogs of pathogenic missense variants in future iterations. For the remaining six epilepsy genes, we built a regression model containing the features with significant contributions, and we refer to a prediction from these models as a gene-specific probability of pathogenicity (GPP) score. The training data used to fit the model was taken from the 606 qualified pathogenic and 1514 Control Group 1 missense variants that overlapped the gene being modeled (Methods; Supplemental Table S8). All six models achieved an AUC ≥ 0.92 when reapplied to the training data. To assess each model's performance on an independent test sample, for each gene, we took missense variants that were not used to select features or fit the model from the 437 unqualified case variants and the 2443 Control Group 2 and Control Group 3 variants. We found that all six models achieved AUC ≥ 0.83 when predicting this independent test sample (Table 3; Supplemental Fig. S6).

**Table 3.**
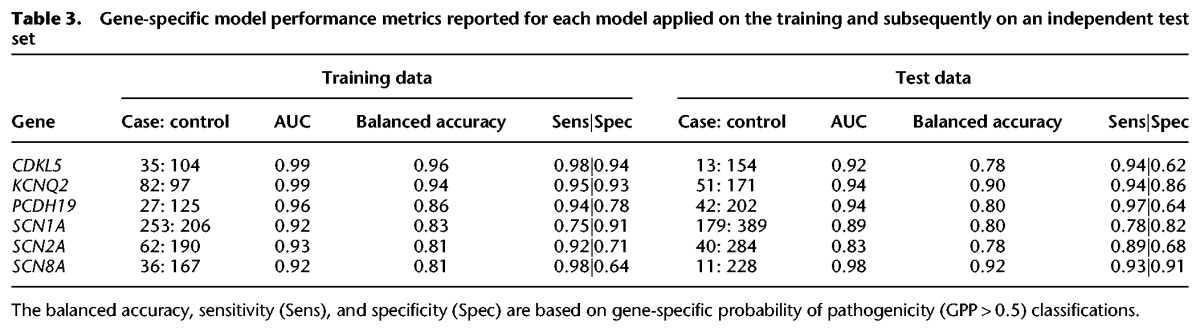
Gene-specific model performance metrics reported for each model applied on the training and subsequently on an independent test set

**Figure 5.**
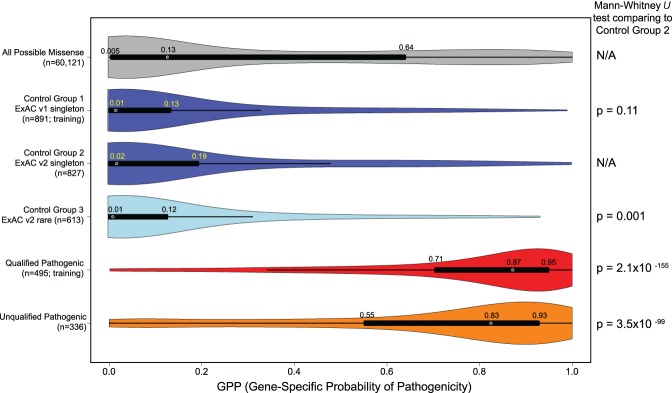
The distribution of the GPP scores from the collection of the six gene-specific logistic models. The tallies of missense variants reported per group reflect the number of missense variants in that group that belong to the six genes for which a multivariate customized logistic model was described in Supplemental Table S5. Control Group 1 and Qualified Pathogenic were the only two groups used to fit the gene-specific models. Control Groups 2 and 3 (presumed enriched for benign) as well as the Unqualified Pathogenic group (presumed enriched for pathogenic variants above that found in population controls) represent missense variants not involved in feature evaluation or model fitting. The Mann-Whitney *U* tests compare the GPP score distributions from each group to the ExAC v2 Control Group 2 GPP score distribution.

Each of the gene-specific models computed the predicted probability that a missense variant is pathogenic for all possible missense variants within each of the individual genes (Supplemental Data S4). We find that these GPP score distributions differ significantly when comparing both the training (Mann-Whitney *U* test, *P* = 2.1 × 10^−155^) and the test (Mann-Whitney *U* test, *P* = 3.5 × 10^−99^) case missense variants to the Control Group 2 (independent test) sample (Fig. 5). There is no significant difference in the GPP distributions between Control Group 1 and Control Group 2 (Mann-Whitney *U* test, *P* = 0.11) (Fig. 5; Supplemental Fig. S7).

We generated another set of predictions by swapping the qualified pathogenic case variants for the unqualified case variants and using the ExAC v2 Control Group 2 and 3 missense variants to fit a new logistic model and continued to observe improvement in the GPP scores among qualified pathogenic variants (Supplemental Fig. S8). An evaluation limited to the six epilepsy genes for which a customized prediction model was constructed, GPP was found to be a superior predictor of pathogenic missense variants than MPC, a recently available integrated missense variant score that includes an alternative subregional intolerance component (DeLong's test for two correlated ROC curves *P* = 4.9 × 10^−17^) (Supplemental Fig. S9; Samocha et al. 2017).

We also generated a global model using the information from all 11 genes to undergo the model building that was performed for the gene-specific models (Supplemental Fig. S10). Although the global model was significantly predictive of pathogenic test variants (Mann-Whitney *U* test, *P* = 2.9 × 10^−88^), compared to the GPP scores from the gene-specific models, the global model achieved lower GPP scores for test case variants (median GPP score of 0.76 versus 0.83) and higher GPP scores for the population control test variants (median GPP score of 0.04 versus 0.02). This was reflected by a weaker difference in score distributions when comparing the test case to the test control samples using the global model (Mann-Whitney *U* test, *P* = 2.9 × 10^−88^) (Supplemental Fig. S10) versus using the gene-specific models (Mann-Whitney *U* test, *P* = 3.5 × 10^−99^) (Fig. 5). These differences provide support for use of a gene-specific pathogenicity prediction model.

### Real-time validation: *SCN2A*

**Figure 6.**
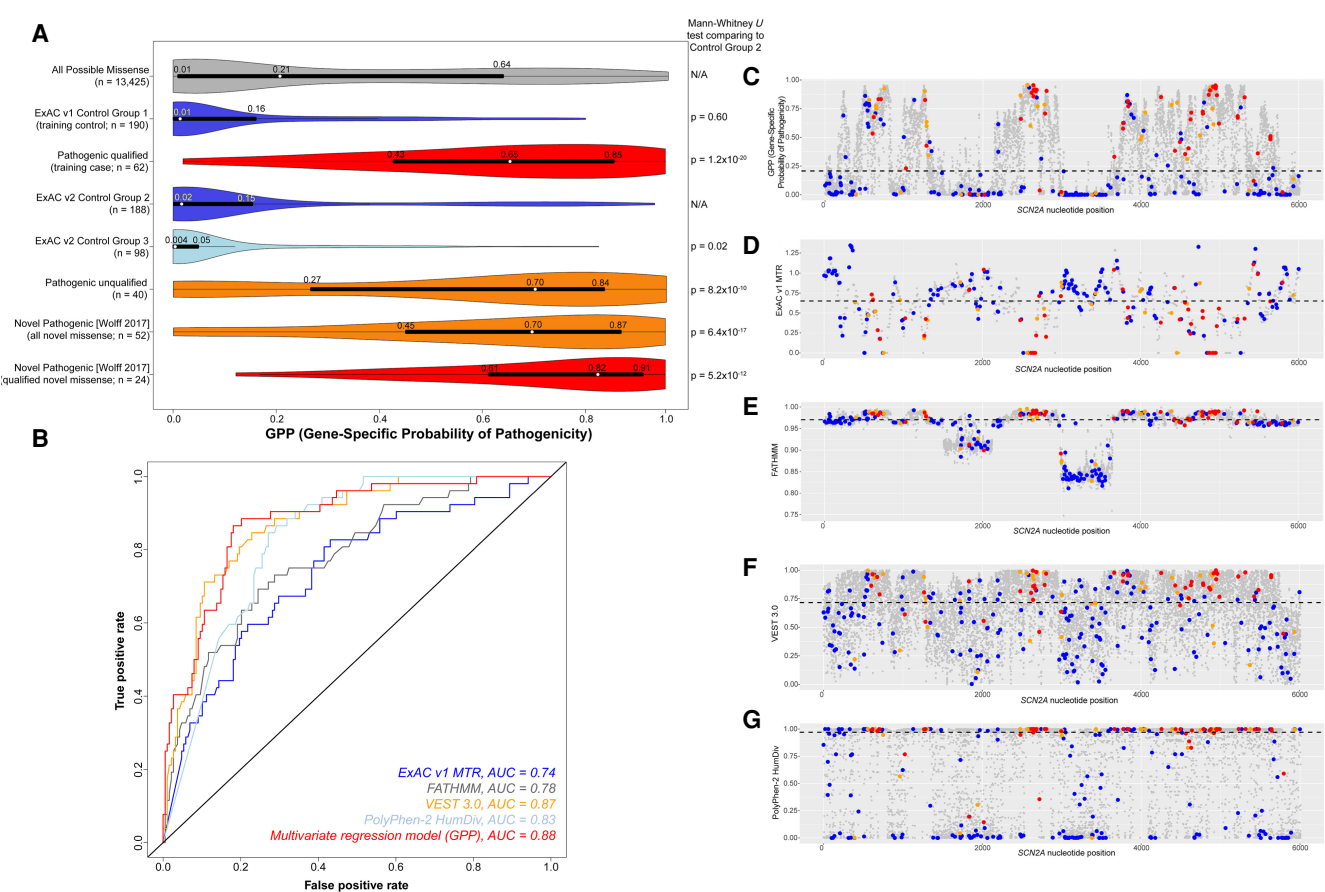
Real-time validation of a *SCN2A* gene-specific model. (*A*) *SCN2A* gene distributions of the GPP scores. All Mann-Whitney *U* tests compare groups to ExAC v2 Control Group 2. Control Groups 1–3 are mutually exclusive presumed benign missense variants. Pathogenic qualified, unqualified, and novel are mutually exclusive presumed pathogenic missense variants. For the *bottom* two plots of novel variants in Wolff et al. (2017), the “qualified novel” group is a “de novo” and severe pediatric epilepsy subset of the ‘all novel’ group. (*B*) ROC curves for the model and individual features accurately predicting the 52 novel case and 188 Control Group 2 variants. (*C*–*G*) Distribution of the model and individual feature scores across all 13,425 possible *SCN2A* missense variants (gray) with the median *SCN2A* score depicted by a dashed black line. Also plotted are the 188 ExAC v2 Control Group 2 (blue), the 52 novel variants from Wolff et al. (2017) (red), and the 40 *SCN2A* unqualified pathogenic test variants (orange).

After the conclusion of our analysis of *SCN2A*, a new paper with a large catalog of novel pathogenic-reported variants provided an ideal opportunity to validate our approach (Wolff et al. 2017). This study reported 52 distinct *SCN2A* pathogenic missense changes not found among our case or control collections. Overall, we found that the newly available *SCN2A* case variants had a significantly higher GPP score (*n* = 52; median GPP score of 0.70) compared to the Control Group 2 variants (*n* = 188; median GPP score of 0.02; Mann-Whitney *U* test, *P* = 6.4 × 10^−17^) (Fig. 6A). Among the subset of novel *SCN2A* missense variants in Wolff et al. (2017) that qualified as having occurred de novo and restricting to severe epileptic encephalopathies, we observed a shift toward higher GPP scores (*n* = 24; median GPP score of 0.82) compared to the remaining novel missense variants (*n* = 28; median GPP score of 0.64; Mann-Whitney *U* test, *P* = 0.02). There was no significant difference between the GPP score distribution of the 52 novel variants and the *SCN2A* pathogenic qualified variants used to fit the *SCN2A* model (*n* = 62; median GPP score of 0.65; Mann-Whitney *U* test, *P* = 0.84). Applied to the new variants, the *SCN2A* gene-specific model achieved an AUC of 0.88, with the ExAC v1 MTR estimate (AUC = 0.74) and each of the independent bioinformatic features achieving high prediction accuracy in this novel case sample: FATHMM (AUC = 0.78), VEST release 3 (AUC = 0.87), and PolyPhen-2 HumDiv (AUC = 0.83) (Fig. 6B).

## Discussion

Evaluating the clinical relevance of a novel missense variant found in an established disease gene is recognized as one of the central challenges facing modern medical genomics (MacArthur et al. 2014; Richards et al. 2015). Although probabilistic bioinformatic tools are unlikely to completely solve this problem, they can optimize the triaging of candidate variants by identifying the empirical bioinformatic signatures of pathogenicity—properties found to be significantly enriched among variants that have been described to be clinically relevant (Petrovski et al. 2013; Zhu et al. 2015).

Recently, we found that pathogenic missense variants preferentially reside in the missense-intolerant subdomain and exon structures of known disease genes (Gussow et al. 2016). Although investigators frequently browse the neighboring protein-coding sequence of a novel missense variant during variant interpretation, in this study we adopted MTR sliding windows to demonstrate how those considerations can be formalized without requiring knowledge of biological structures within a gene. We showed that this valuable information identifies the important regions of genes, where clinically relevant missense variants are more likely to be found.

The resolution permitted by currently available standing variation data limits the window size that can be applied in calculating MTR estimates. For the current ExAC v1 and ExAC v2 samples, a window of 31 codons captured on average 11 and 16 distinct variants per window, respectively. Overall, a greater number of MTR sliding windows 4641 (35%) of the 13,386 windows achieved a FDR < 0.05 when using the ExAC v2 sample compared to ExAC v1, where this was achieved for 3157 (24%) of the windows (Supplemental Data S3). As larger reference cohorts emerge, more windows will have the resolution required to achieve significance, and there will also be opportunities to reduce window sizes. Reduced window sizes will enable better identification of the critical boundaries and will also allow these measures to be sensitive to smaller critical regions, where 31 codons might currently be insensitive. Most importantly, with larger reference cohorts, more sophisticated approaches to detecting signatures of purifying selection, such as the RVIS framework (Petrovski et al. 2013; Gussow et al. 2016), will become more amenable to small window applications and potentially expand into the noncoding sequence (Petrovski et al. 2015). Although it is some time away (Zou et al. 2016), eventually when enough exomes have been sampled from a diverse range of ancestral populations, we can expect the germline allelic frequency at every position will begin to reflect a nucleotide-level intolerance estimate.

Like previous illustrations using our regression-based RVIS (Petrovski et al. 2013; Zhu et al. 2015), we expect the optimal use of this regional-based missense intolerance information will be in combination with variant-level predictions of deleteriousness. In this study, we evaluated the predictive utility of 20 widely utilized variant-level tools. Understanding where in a specific gene's score distribution a novel missense variant resides and which tools best capture pathogenicity for a given disease gene have been practical limitations in relying on bioinformatic tools. These considerations are important given the growing number of missense prediction tools available (Cooper and Shendure 2011; Goldstein et al. 2013). Evaluating the predictive utility of 20 variant-level tools in a gene-customized framework allowed us to empirically determine the informative tools; for six highly informed epilepsy genes the data also supported integrating information across two or more tools. For both *KCNQ2* and *SCN8A*, a gene-specific integrated model achieved superior predictive accuracy compared to its individual components when applied to independent test data. For the remaining four genes, the model predicted the test data as well as the top performing feature. We did, however, observe stochasticity in the top performing feature for a gene based on differing samples, whereas the gene-specific model consistently performed as well or better than the top performing feature. This stochasticity is best demonstrated by the multiple applications of the *SCN2A* model. The top performing feature on the training data was FATHMM (AUC = 0.87), on the original test data it was PolyPhen-2 (AUC = 0.83), and on the novel test data it was VEST 3.0 (AUC = 0.87). Yet, in all three data sets, the multivariate gene-specific model did just as well or better than the top performing component (training data AUC = 0.93; original test data AUC = 0.83; novel test data AUC = 0.88).

This framework has much broader relevance as the same problems arise for many disease genes. Our initial focus on epilepsy is motivated by the fact that epilepsy has a large disease burden, has a large effect on quality of life, and its genetic architecture has proved to be highly suited to sequencing approaches, with a large number of distinct causal variants identified in a growing set of epilepsy-related genes (Epi4K Consortium and Epilepsy Phenome/Genome Project 2013, 2017; EuroEPINOMICS-RES Consortium et al. 2014). In the current study, we present results of applying a gene-customized framework on 11 genes with epilepsy association, although we intend to expand this work to all human disease genes. As the catalogs of pathogenic variants increase in size, we will retrain and revalidate this approach based on additional variants found among evaluated genes.

Although we do not expect empirical-based estimates of regional purifying selection to outperform existing bioinformatic tools, it is clear that these signals do contribute independent previously uncaptured information to the prediction of pathogenic variants. Given this demonstrable utility, quantification of regional missense intolerance can be expected to improve over time. The current results for the most informed epilepsy genes motivates further evaluation of the GPP scores generated by gene-specific models as alternatives to relying on individual tools in a generalized way. Or, at the very least, our findings indicate the individual tools that have been empirically shown to have, and not have, predictive utility in a given epilepsy gene.

## Methods

### Epilepsy gene transcript selection

To ensure that every variant in this study was based on the same epilepsy gene transcript, we focused on the uniprot canonical transcript (http://www.uniprot.org/) (The UniProt Consortium 2017). We appreciate that true pathogenic variants may affect alternate exons not captured by the canonical transcript; however, to ensure consistency we focused on a single transcript per epilepsy gene throughout this paper:

- *SCN1A*: [uniprot P35498-1; ENST00000303395; CCDS54413.1; NM_001165963.1 aka NM_001202435.1]
- *KCNQ2*: [uniprot O43526-1; ENST00000359125; CCDS13520.1; NM_172107.3]
- *SCN2A*: [uniprot Q99250-1; ENST00000283256; CCDS33314.1; NM_001040142.1 aka NM_021007.2]
- *SCN8A*: [uniprot Q9UQD0-1; ENST00000354534; CCDS44891.1; NM_014191.3]
- *STXBP1*: [uniprot P61764-1; ENST00000373299; CCDS35146.1; NM_001032221.3]
- *SLC2A1*: [uniprot P11166-1; ENST00000426263; CCDS477.1; NM_006516.2]
- *GRIN2A*: [uniprot Q12879-1; ENST00000396573; CCDS10539.1; NM_000833.4 aka NM_001134407.2]
- *LGI1*: [uniprot O95970-1; ENST00000371418; CCDS7431.1; NM_005097.3]
- *KCNT1*: [uniprot Q5JUK3-3; ENST00000371757; CCDS35175.2; NM_020822.2]
- *CDKL5*: [uniprot O76039-1; ENST00000379989; CCDS14186.1; NM_001037343.1 aka NM_003159.2]
- *PCDH19*: [uniprot Q8TAB3-1; ENST00000373034; CCDS55462.1; NM_001184880.1]

### Patient-ascertained pathogenic variant collections

We focused on 11 dominant epilepsy genes (EpiPM Consortium 2015) each reporting at least 20 epilepsy-associated “pathogenic” missense variants among the combination of ClinVar (Landrum et al. 2016) (ftp://ftp.ncbi.nlm.nih.gov/pub/clinvar/; accessed May 1, 2016) and HGMD (Stenson et al. 2003) (hgmd2016.3) databases (Supplemental Table S1).

Pathogenic variants were selected on the basis of the following conditions: for ClinVar, we required the classification to be “Pathogenic,” “Likely Pathogenic,” or “Likely pathogenic; Pathogenic”; for HGMD, we required the classification to be “DM.” For missense variants reported in ClinVar and HGMD, both had to have consensus pathogenicity claims as described above. Epilepsy association was defined by keyword matching ClinVar and HGMD phenotypes with at least one of the following keywords: seizure, epilepsy, convulsion, Gastaut, spasm, glucose (for *SLC2A1*), Ohtahara, west syndrome, encephalopathy, or Dravet. ClinVar “N/A” or “not provided” were permitted following review of the text in the corresponding ClinVar report.

For the resulting 1043 variants, two researchers independently screened the literature associated with the corresponding variant (or the clinical notes left within the ClinVar page) to identify variants reported with sufficient segregation support. For the purposes of this study, sufficient segregation support was qualified as having met one of the following two criteria:

1. There was written evidence that the specific variant arose de novo (germline or somatic) in the family; or
2. The variant was accompanied by pedigree support showing that the variant segregated among all (and more than three) affected carriers that were genotyped, and was not present in more than one of the genotyped unaffected carriers in the pedigree.

Across the 1043 missense variants deposited in the ClinVar/HGMD variant databases (Supplemental Data S1), 606 were found to have the segregation support described above (Supplemental Table S1). The intent of this task was to prune our list of case-ascertained pathogenic-reported variants to the subset that we consider enriched for clinically relevant missense variants, based on the level of segregation evidence accompanying the claims of pathogenicity in literature or via ClinVar (Supplemental Data S1).

### Population control variants

To sample from presumed benign missense variation, we used two large samples of human standing variation with the ExAC v1 reference sample representing a subset of the ExAC v2 and gnomAD reference sample (Lek et al. 2016). We systematically sampled missense variants passing the quality control checks imposed by the data set creators and defined three mutually exclusive groups of control variants:

Control Group 1: *N* = 1517 singleton missense variants from the ExAC v1 sample (i.e., only one allele observed among up to 121,412 chromosomes).
Control Group 2: *N* = 1377 singleton missense variants from the combined ExAC v2 and gnomAD sample. Since ExAC v2 includes ExAC v1 samples, we excluded all variants reported at any frequency in ExAC v1.
Control Group 3: *N* = 1066 rare (nonsingleton) missense variants among ExAC v2 and gnomAD with a MAF < 0.05% across each of the seven ExAC v2 ancestry groups, excluding missense variants contained in Controls Groups 1 or 2.

We focused on singleton and rare missense variants to best match our control variants to the site frequency spectrum (SFS) commonly observed for epilepsy patient–ascertained variants (Epi4K Consortium and Epilepsy Phenome/Genome 2017). Moreover, focusing on the lowest end of the available SFS helps ensure that the variants we used as controls are unlikely to have contributed to the training of the various pre-ExAC bioinformatic tools that we evaluated. As with the case variants, we acknowledge that some of these control-ascertained variants might contribute to epilepsy risk; however, we expect such a false negative rate to be low, as also supported by earlier epilepsy sequencing studies (Epi4K Consortium and Epilepsy Phenome/Genome 2017).

### The Missense Tolerance Ratio (MTR)

To illustrate the landscape of missense tolerance, we used aggregated variant data from two publicly available samples of human standing variation to highlight regions within genes that reflect preferential depletion of missense variation given the total variation observed. The first data set ExAC v1 (release 0.3.1) represents a sample of 60,706 unrelated individuals. The second data set is the recently released ExAC v2 and gnomAD combined data (version 2.0), representing a sample of 138,632 unrelated individuals (Lek et al. 2016).

The *d*_N_/*d*_S_ score was introduced to enable detection of selective evolutionary pressure in interspecies comparisons (Kimura 1977). The *d*_N_/*d*_S_ is like an odds ratio, and by construction, its estimates can be imprecise when *d*_N_ or *d*_S_ are small, which was usually not the case in the historical context of interspecies comparisons over whole genes. To accommodate the skewed distribution near *d*_N_/*d*_S_ = 0, the log of *d*_N_/*d*_S_ is often adopted. Here, we assessed missense depletion using a sliding window approach in which the observed counts of missense (*D*_n_) and synonymous (*D*_s_) variants can be limited by resolution in a window. In this context, we found that log(*d*_N_/*d*_S_) becomes volatile near zero and is undefined if either *D*_n_ or *D*_s_ is zero. For this reason, we adopted a modified formulation that uses similar elements and is intuitive in its own right, yet simpler than log(*d*_N_/*d*_S_).

A 31-codon (i.e., 93 protein-coding nucleotides) sliding window was applied to the protein-coding sequence of human genes to estimate the Missense Tolerance Ratio (MTR), per window:

$$\mathrm{MTR}=\frac{\;[\mathrm{missens}e_{\left[\mathrm{obs}\right]}/(\mathrm{missens}e_{\left[\mathrm{obs}\right]}+\mathrm{synonymou}s_{\left[\mathrm{obs}\right]})]}{\;[\mathrm{missens}e_{\left[\;\mathrm{pos}\right]}/(\mathrm{missens}e_{\left[\;\mathrm{pos}\right]}+\mathrm{synonymou}s_{\left[\;\mathrm{pos}\right]})]},$$

where the numerator of MTR presents the observed proportion in a given window of missense variants among the missense and synonymous variants combined. We scaled this by the more stable denominator, which is the same proportion but computed not from the observed variants, but from the set of all possible missense and synonymous variants in the window. We found that this formulation remains highly correlated with log(*d*_N_/*d*_S_) (Supplemental Fig. S11) while avoiding problems when either *D*_n_ = 0 or *D*_s_ = 0.

The expected proportion of missense variants in a given protein-coding window was calculated by annotating all possible variants with VEP (Ensembl GRCh37 release 85, July 2016) and assuming all events were equally possible. We focused on the protein-coding missense and synonymous single-nucleotide variants (SNVs) within the isoforms defined above, under section Epilepsy gene transcript selection. The observed proportion of missense variation for the same protein-coding window was calculated by focusing on the missense and SNVs judged to pass the quality criteria assigned by the database creators of ExAC and based on annotations from the same isoforms as described in Epilepsy gene transcript selection. Thus, for any given 31-codon sliding window, we calculated the proportion of observed missense variants reported in the ExAC public databases given the total sum of observed missense and synonymous variants reported in that window. A summary of the observed variation among the combined ExAC v2 and gnomAD database is provided for each of the 11 epilepsy genes (Supplemental Table S3).

By construction, each codon's MTR estimate represents the preferential depletion of missense variants at the index codon and the 15 codons before and after the index codon. The first and last 15 codons of a transcript reflect smaller window sizes (Supplemental Data S3). Importantly, a sliding window approach was adopted to allow our estimates to be entirely independent of existing biological boundaries, such as exons, conserved domains, and functional domains. The window size of 31 codons captured a median of 10 variants per window (mean 10.6 ± 5.6 variants) based on the ExAC v1 release 0.3.1 data. This window size subsequently captured a median of 15 variants per window (mean 16.1 ± 7.3 variants) based on the larger combined ExAC v2 and gnomAD sample (release 2.0). As expected, the number of variants per window when comparing the ExAC v1 and the ExAC v2 databases was highly correlated (Pearson's *r* = 0.88) (Supplemental Data S3).

Although the MTR is not sensitive to variability in sequencing coverage, regions with lower coverage should be interpreted with caution given the reduced sample contributing to those corresponding MTR window estimates. For this reason, we also plotted the proportion of the ExAC samples that reported at least 10-fold coverage across each codon (Supplemental Data S3). We also adopted a Binomial exact test to test for a deviation from MTR = 1 at each sliding window and further adjusted for the study-wide false discovery rate (FDR) by using the Benjamini-Hochberg procedure (Supplemental Data S3). Our MTR viewer is publicly available and currently supports Ensembl v85 transcripts (http://mtr-viewer.mdhs.unimelb.edu.au). The website accepts as input either individual transcript IDs or displays the canonical transcript when a HGNC gene symbol is provided.

The MTR landscapes of human-lineage purifying selection can be biologically interesting for at least three additional reasons: (1) they highlight that even within known functional domains there is variability in missense intolerance; (2) we observed divergence of intolerant regions among highly homologous *SCN1A*, *SCN2A* and *SCN8A* genes (Fig. 1 G–I), suggesting those could be regions within the homologs that are important to their distinct functional roles in the central nervous system and opening up new scientific questions about the functional and clinical importance of these regions; and (3) conversely, we observed that many of the troughs across the landscapes of these three sodium channel genes overlap, reinforcing those critical functions as being maintained by purifying selection across the gene family.

We also compared MTR to known subregional intolerance scores across the 11 genes and found low correlation with subRVIS (Pearson's *r*^*2*^ of 0.008 [CDD] and 0.009 [exon]) (Gussow et al. 2016) and the preprint obs_exp constraint estimates (Pearson's *r*^*2*^ of 0.153) (Samocha et al. 2017). In doing this, we also found that the 11 epilepsy genes comprised more distinct MTR estimates (*n* = 7708), compared to these other boundary-based regional intolerance estimates: subRVIS-exon (*n* = 33), subRVIS-CDD (*n* = 34), and preprint obs_exp regional constraint score (*n* = 24 distinct units, including three genes with a nonvariable estimate). Supplemental Figure S12 comparisons illustrate extra information that MTR estimates capture, highlighting that even within known functional domains, exonic units, or other large divisions, there is important variation in missense intolerance.

### Annotating the 88,865 possible missense variants

We generated all possible missense variants that could affect the canonical transcripts (as defined earlier) of the 11 studied epilepsy genes. This resulted in 88,865 possible missense variants that we subsequently annotated using 31 various bioinformatic tools based on the following three variant annotation platforms:

- PolyPhen-2 (http://genetics.bwh.harvard.edu/pph2/) was used to derive predictions from both HumDiv and HumVar PolyPhen-2 models focused on the Consensus Coding Sequence (CCDS) transcripts.
- The *Ensembl* Variant Effect Predictor (VEP; accessed December 2016) was used to annotate missense variants with 24 bioinformatic tools specific to the canonical transcripts. To standardize all scores, we adopted the rankscore annotations from the dbNSFP database version 3.2c (https://drive.google.com/file/d/0B60wROKy6OqcUl9NbkFRdVZlQzQ/view).
- Finally, we used the Combined Annotation Dependent Depletion (CADD v1.3; http://cadd.gs.washington.edu/score) platform to extract five additional features for the missense variants: GC and CpG (percent in a window of ±75 bp), Primate-based PhastCons and PhyloP scores, and the Grantham score.

We could not annotate the X Chromosome genes *CDKL5* and *PCDH19* for five features (Eigen-PC-raw_rankscore, Eigen-raw_ rankscore, H1-hESC_fitCons_score_rankscore, HUVEC_fitCons_ score_rankscore, and integrated_fitCons_score_rankscore), so we only evaluated these five features for the autosomal genes. After excluding these five features for the two X Chromosome genes, we had missing data for 2276 (0.08%) of all 2,683,040 annotations (Supplemental Data S4). We imputed these missing annotations by taking the median score of the successfully annotated missense variants within the corresponding gene.

### Identifying highly correlated in silico features

We used the nearZeroVar function within the R caret package to identify features achieving near-zero variance. We defined near-zero variance using a freqCut of 80/20 (i.e., 4 as the cutoff for the permitted ratio of the most common value to the second most common value in the feature) and uniqueCut of 5 (i.e., the cutoff for the percentage of distinct values available out of the total 74,510 autosomal missense variants). Through this step, we found that the phastCons20way_mammalian_rankscore (freqCut = 5.2% and percent unique = 1.2%) and phastCons100way_vertebrate_ rankscore (freqCut = 75.3% and percent unique = 1.1%) did not meet the two requirements and were not included in feature importance evaluations.

We also generated a correlation matrix using the 31 features (Supplemental Fig. S4) and adopted the FindCorrelation function from the R caret package to identify features with high pairwise Pearson's *r* correlations, defined as an absolute value >0.75 (default). We also set the FindCorrelation exact parameter to equal true. Thus, when a pairwise correlation with an absolute value >0.75 was identified, the function selected which feature to remove by considering the mean of the absolute values of all pairwise correlations for both features, and it removed the feature with the larger mean absolute correlation across the matrix. Adopting the exact = true ensures that this function reevaluated the average absolute correlations at each step; thus, the average absolute mean did not consider earlier features already eliminated due to high correlation. Through this step, we identified and removed the following nine features from subsequent feature importance evaluations: CpG (>|0.75| with CG content); PP2_Hvar (>|0.75| with PP2_Hdiv, Condel and Eigen_rs); Condel (>|0.75| with PP2_Hvar, CADD and Eigen_rs); EigenPC_rs (>|0.75| with CADD, Eigen_rs and phyloP_ver_rs); Eigen_rs (>|0.75| with PP2_Hdiv, PP2_Hvar, Condel, CADD, EigenPC_rs and MutationAssessor_rs); MetaLR_rs (>|0.75| with FATHMM_rs and MetaSVM_rs); MetaSVM_rs (>|0.75| with FATHMM_rs and MetaLR_rs); phyloP_ver_rs (>|0.75| with EigenPC_rs and fathmm_MKL_rs); and integrate_fitCons_rs (>|0.75| with H1_hESC_fitCons_rs and HUVEC_fitCons_rs).

Subsequently we included the MTR estimate from the ExAC v1 sample to the correlation matrix to address an interesting question about whether any existing bioinformatic tools correlated with the novel empirical estimates of regional missense depletion in standing variation data (Supplemental Data S1; Supplemental Fig. S4).

### Using the Boruta algorithm to assess feature importance

In the previous steps, we removed two features due to near-zero variance and an additional nine due to high correlations (Pearson's |*r*| > 0.75). We then adopted the Boruta algorithm (R package Boruta) for random forest classifiers to evaluate which of the 20 remaining bioinformatic tools are predictive of pathogenicity in a given gene (Kursa and Rudnicki 2010). The Boruta algorithm adopts an “all-relevant” feature importance assessment using a robust permutation-based approach to identify features that are in some circumstances relevant to the classification outcome of interest, rather than attempting to achieve a minimal subset of features. Boruta judges importance by a feature's ability to outperform randomized instances of all the studied true features (referred to as shadow features). Shadow features are obtained for all 20 bioinformatic features by randomly shuffling each original feature's values across the observations, repeatedly. Informative features are then defined as features with a random forest *Z*-score distribution above that of the highest performing randomized feature (i.e., max shadow feature) (Kursa and Rudnicki 2010). The *Z*-scores reflected the mean decrease accuracy measure in R's randomForest function. Within the R package, we set our seed to be 15; to increase our confidence, we set our maxRuns to represent 1000 random forest runs (an order of magnitude greater than the default setting) and used the R randomForest default settings of ntree = 500 and mtry = 4. Thus, for a given gene, only the features that consistently achieve higher importance scores (*Z*-scores) than the *Z*-score distribution from the best-performing (max) shadow feature across all the random forest runs were selected as informative (Fig. 4).

We sought to minimize circularity in our feature evaluations by relying only on ExAC v1 and ExAC v2 singleton and rare (MAF < 0.05%) variants that would not have had major contribution to the training sets of these features. Taking the most consistently top ranked feature VEST 3.0, in its training it adopted missense variants with a minor allele frequency >1% among the Exome Sequencing Project (ESP6500) and 47,000 HGMD pathogenic missense variants (Carter et al. 2013). Although our study design sought to alleviate concerns about the total impact of circularity affecting feature evaluations, this effect remains an important consideration (Grimm et al. 2015).

### Deriving a gene-specific prediction of pathogenicity (GPP) model

To assess whether integrating multiple sources of information can provide an improved prediction of missense variant pathogenicity for epilepsy genes, we generated logistic regression models, one for each gene, to distinguish the 606 qualified pathogenic variants (y = 1) from the ExAC v1 Group 1 control missense variants (y = 0).

For the seven epilepsy genes of which the ExAC v1 MTR achieved *P* < 0.0045 (Table 1), we included the ExAC v1 MTR as the initial predictor. We then added that gene's top ranking feature (Supplemental Table S5) and computed the Akaike information criterion (AIC) for the model, *AIC*_*i*_. The model grew by including the next ‘highly informative’ feature, *AIC*_*i*+1_, until the addition of additional features no longer significantly reduced information loss (*P* > 0.05) compared to the previous model (Supplemental Table S7). Given the currently available samples of case and control missense variants, the final model included only one feature for genes *GRIN2A, KCNT1, LGI1, SLC2A1*, and *STXBP1*. For the remaining six epilepsy genes, we fit the following logistic regression model:

$$logit[Pr(Y=1)]=\beta_{0}+\;\beta_{1}X_{1}+\cdots +\beta_{i}X_{i},$$

where β_0_ is the intercept coefficient, and β_*i*_ is the logistic regression coefficient for the corresponding feature score, *X*_*i*_. The multivariate logistic regression models for the six epilepsy genes are provided in Supplemental Table S8.

We also generated another set of pathogenicity-predicted probabilities by substituting the ExAC v1 MTR estimates with the more informed ExAC v2 MTR estimates in the logistic models (Supplemental Data S4). Comparing the two models, we observed high correlation between the predicted probabilities of the resulting 60,121 scored missense variants (Pearson's *r* = 0.94; *P* < 1 × 10^−16^) (Supplemental Data S4).

## Competing interest statement

S.F.B. discloses payments from UCB Pharma, Novartis Pharmaceuticals, Sanofi-Aventis, and Jansen Cilag for lectures and educational presentations, and a patent for *SCN1A* testing held by Bionomics, Inc. and licensed to various diagnostic companies. S.P. serves on the advisory board and is an equity holder of Pairnomix.

## Supplementary Material

Supplemental Material
