# Supplementary material for: Optimizing genomic medicine in epilepsy through a gene-customized approach to missense variant interpretation
Source: Genome Res. 2017 Oct;27(10):1715–29. doi: 10.1101/gr.226589.117 (PMC5630035; doi:10.1101/gr.226589.117)
Supplement: Supplemental Material [file supp_27_10_1715__index.html]

Optimizing genomic medicine in epilepsy through a gene-customized approach to missense variant interpretation — Supplemental Material 

# Optimizing genomic medicine in epilepsy through a gene-customized approach to missense variant interpretation

## Supplemental Material

- Supplemental\_Fig\_S2.pdf
- Supplemental\_Fig\_S3.pdf
- Supplemental\_Fig\_S5.pdf
- Supplemental\_Fig\_S6.pdf
- Supplemental\_Fig\_S10.pdf
- Supplemental\_Data\_S1.xlsx
- Supplemental\_Data\_S2.xlsx
- Supplemental\_Data\_S3.xlsx
- Supplemental\_Data\_S4.zip
- Supplemental\_Appendix.pdf
