# Supplementary figures and images for: Optimizing genomic medicine in epilepsy through a gene-customized approach to missense variant interpretation
Source: Genome Res. 2017 Oct;27(10):1715–29. doi: 10.1101/gr.226589.117 (PMC5630035; doi:10.1101/gr.226589.117)

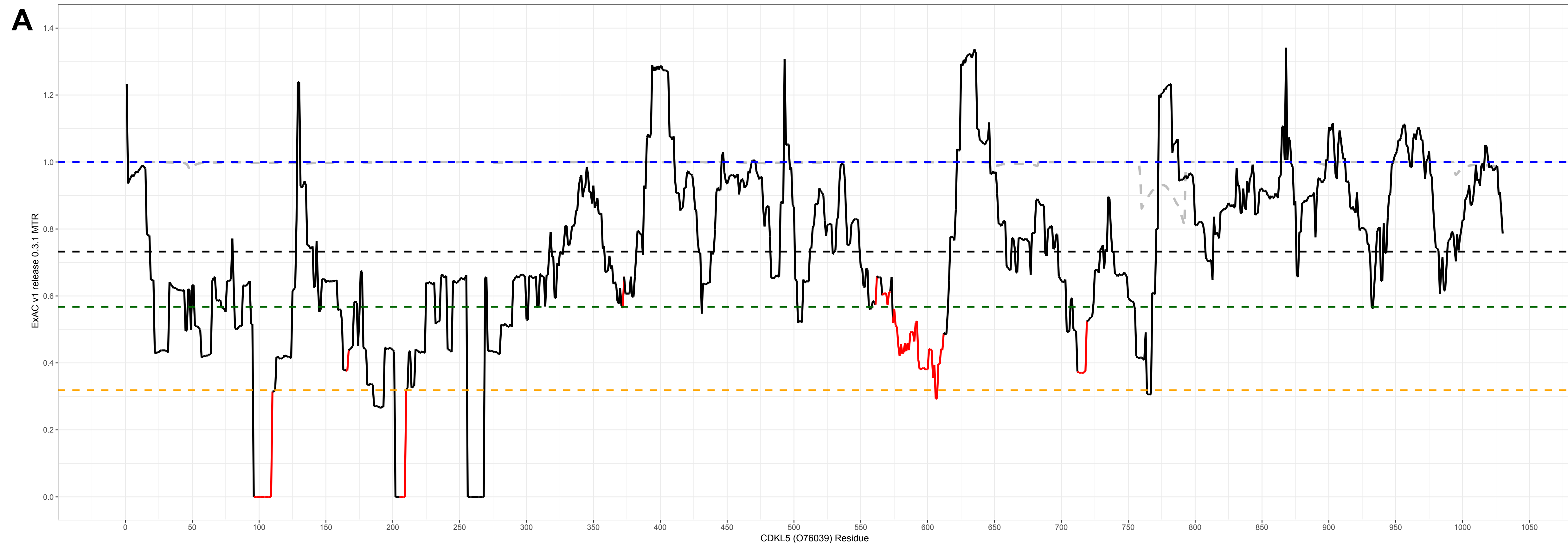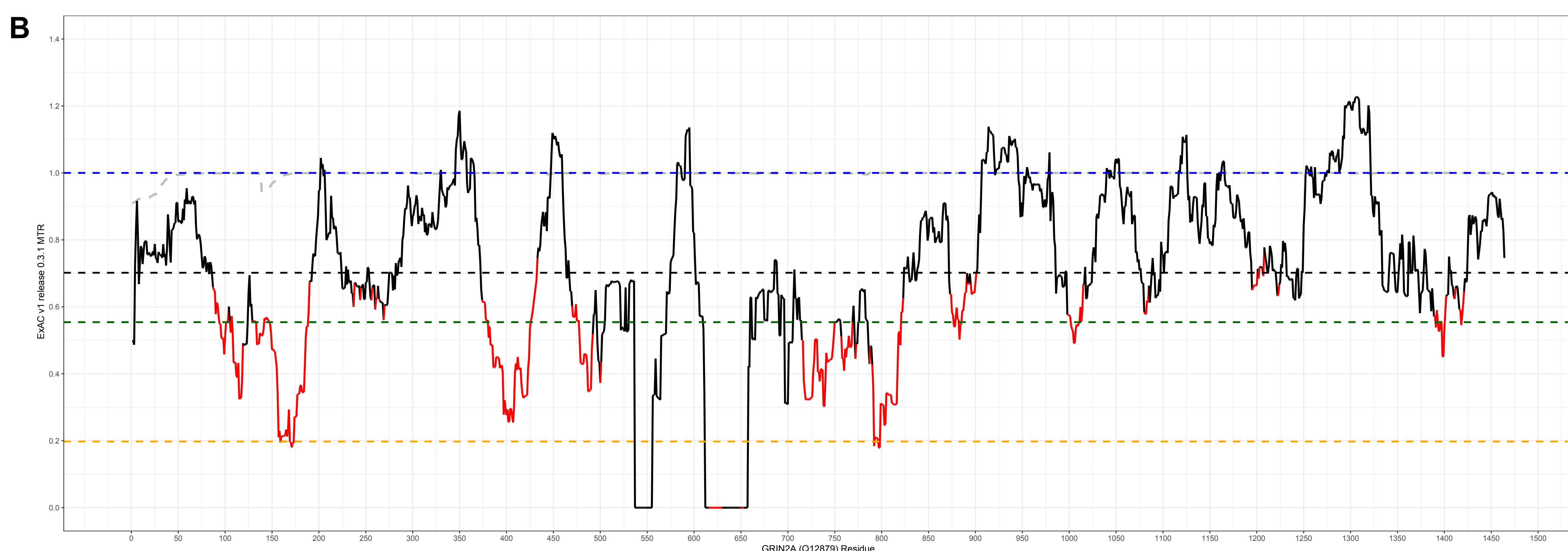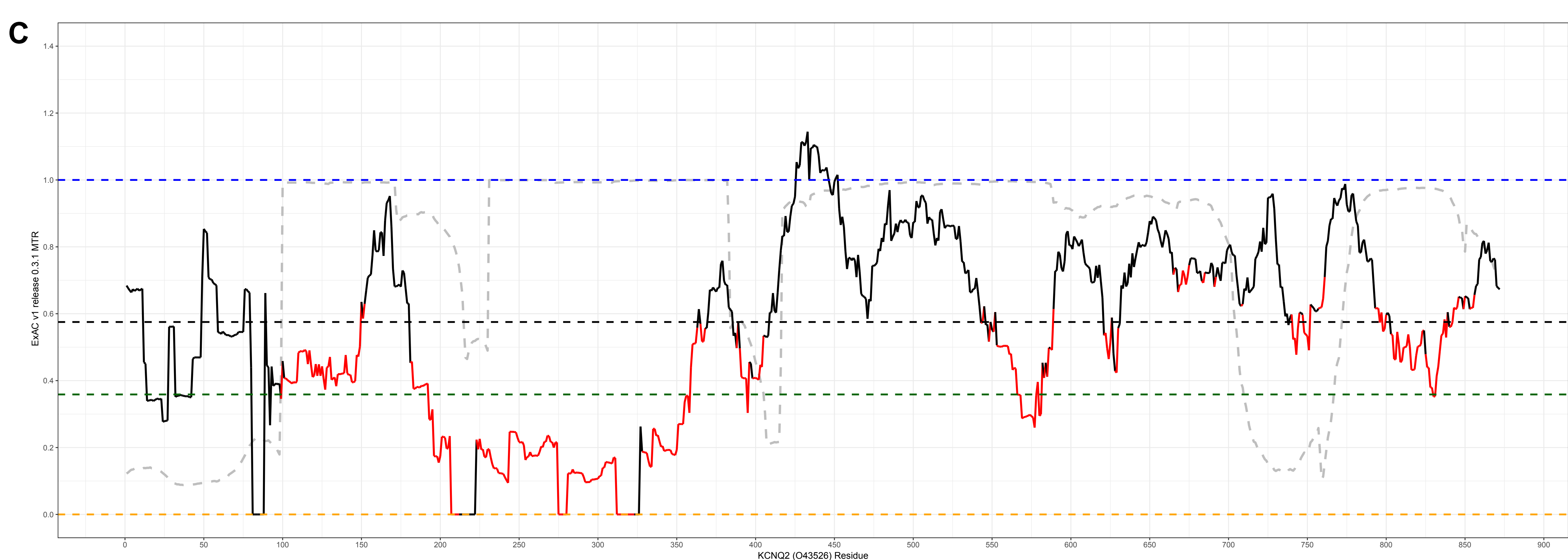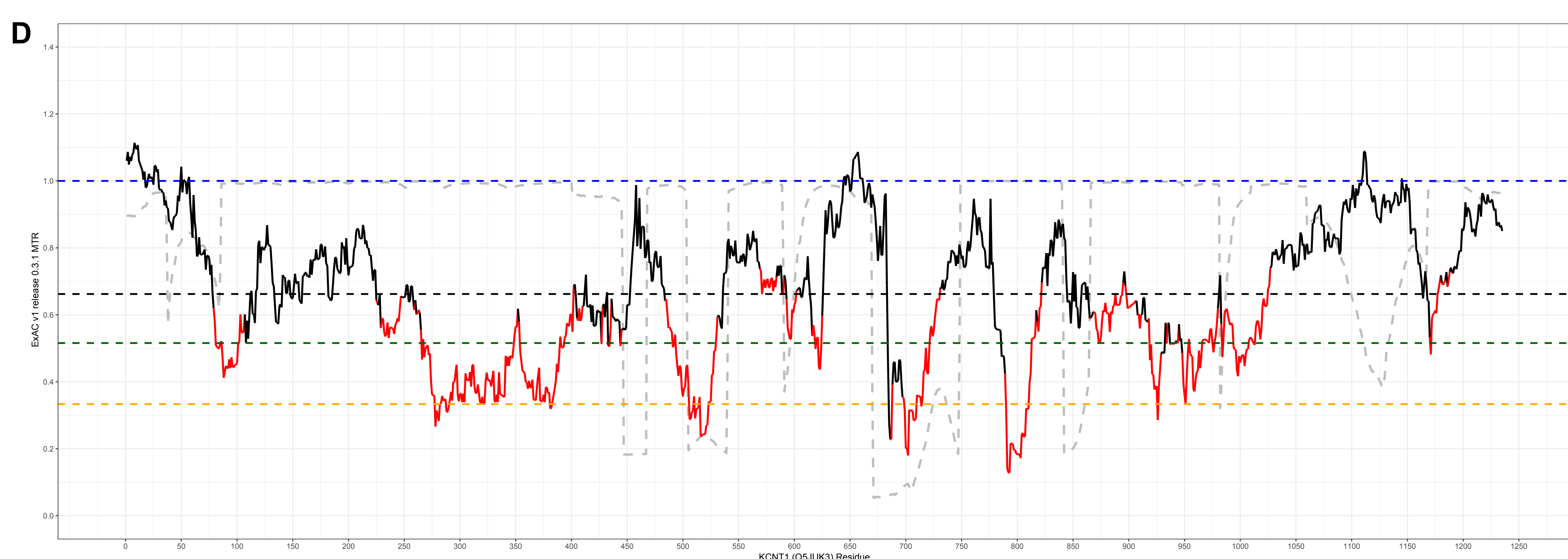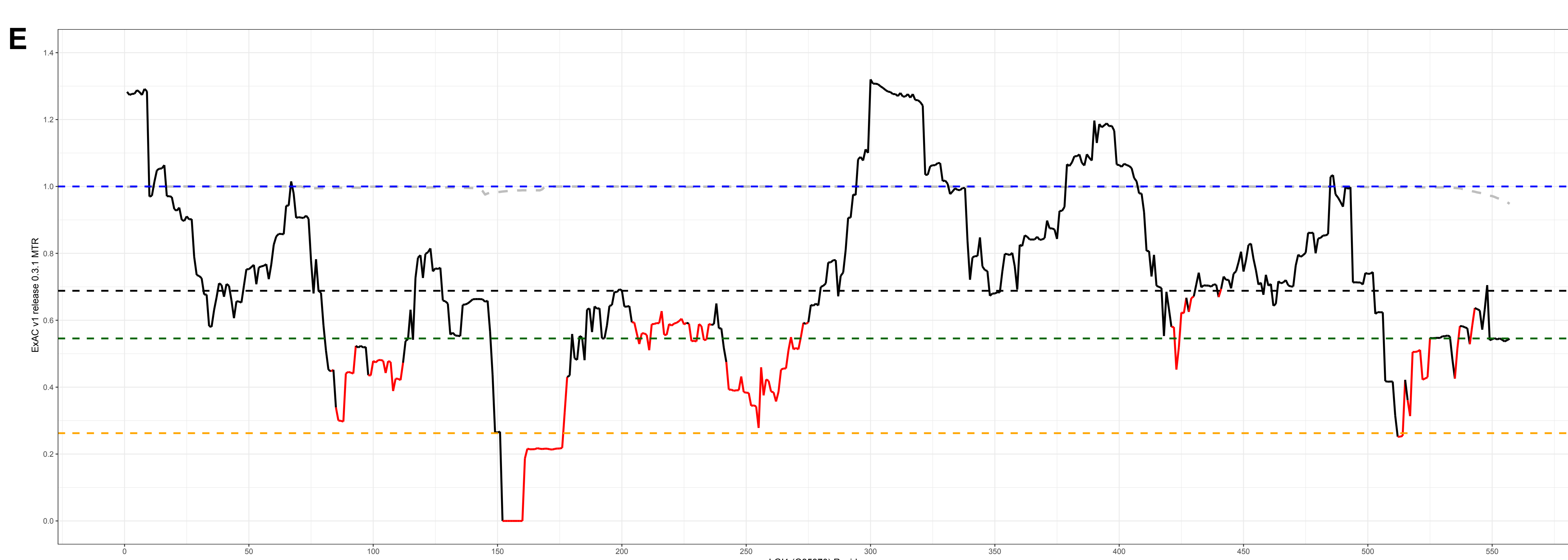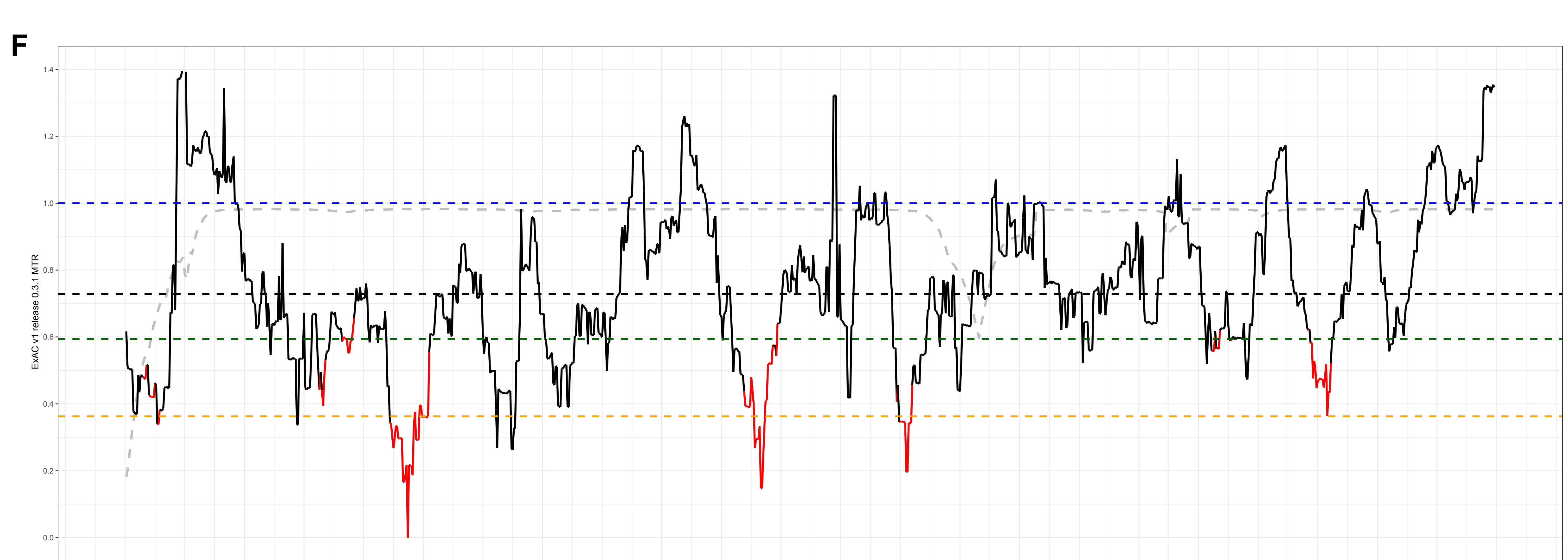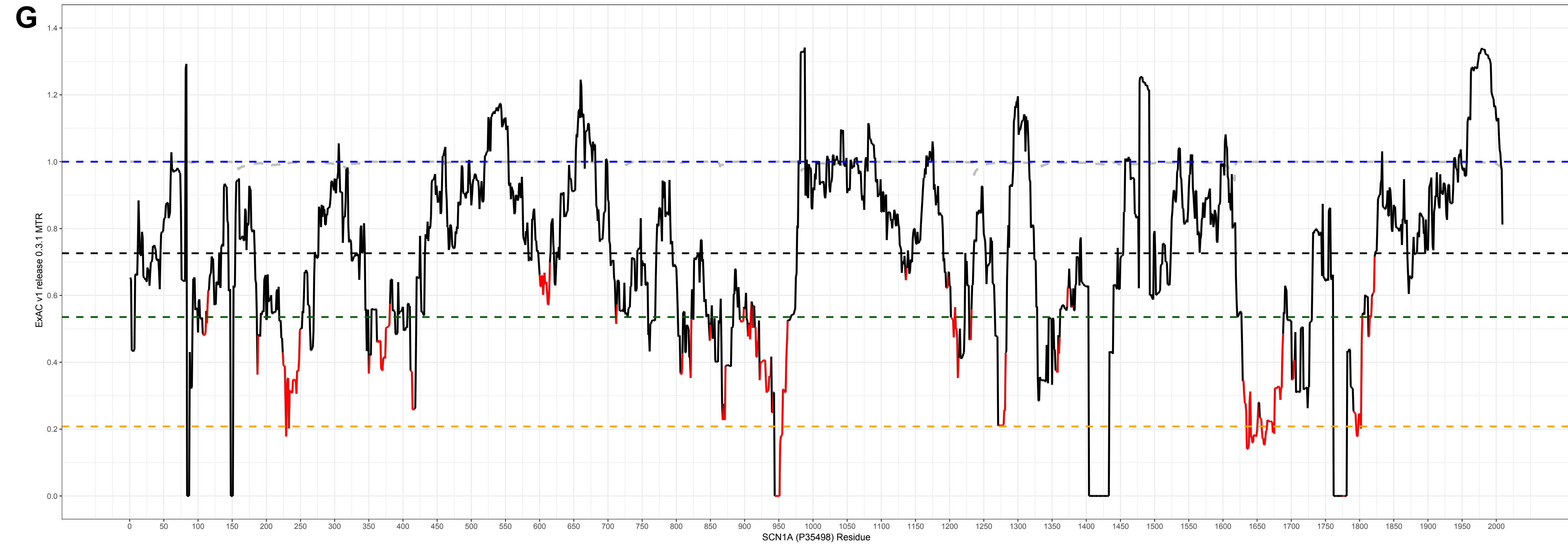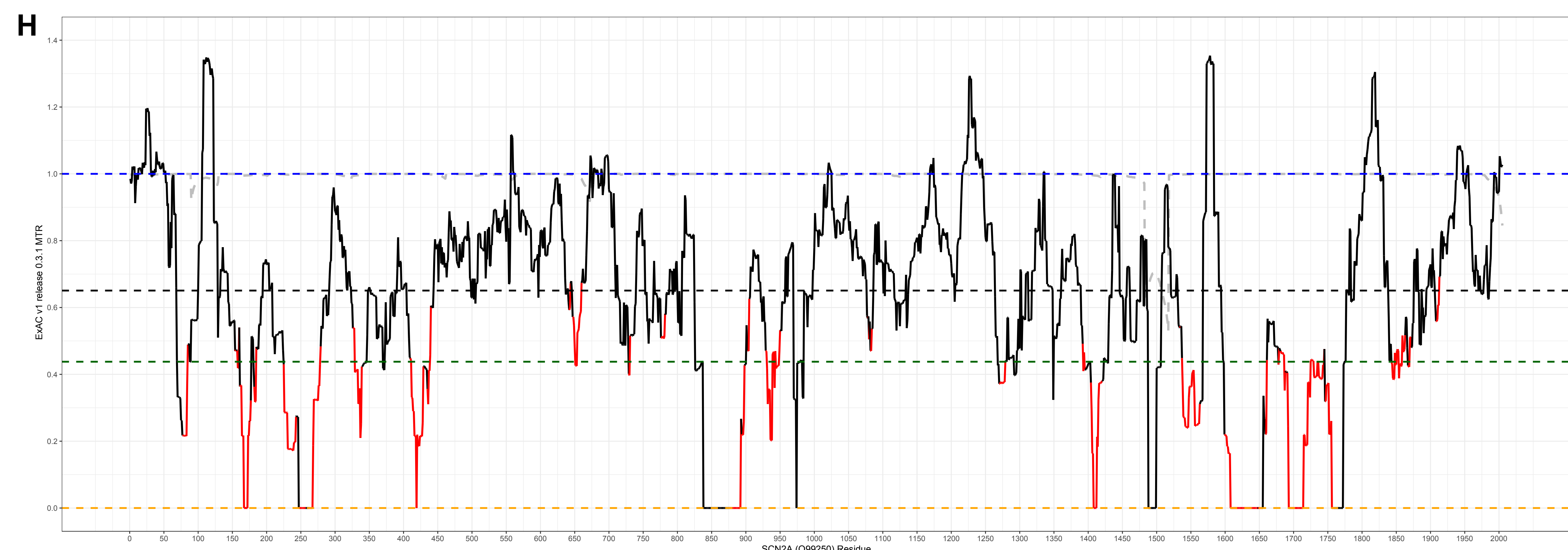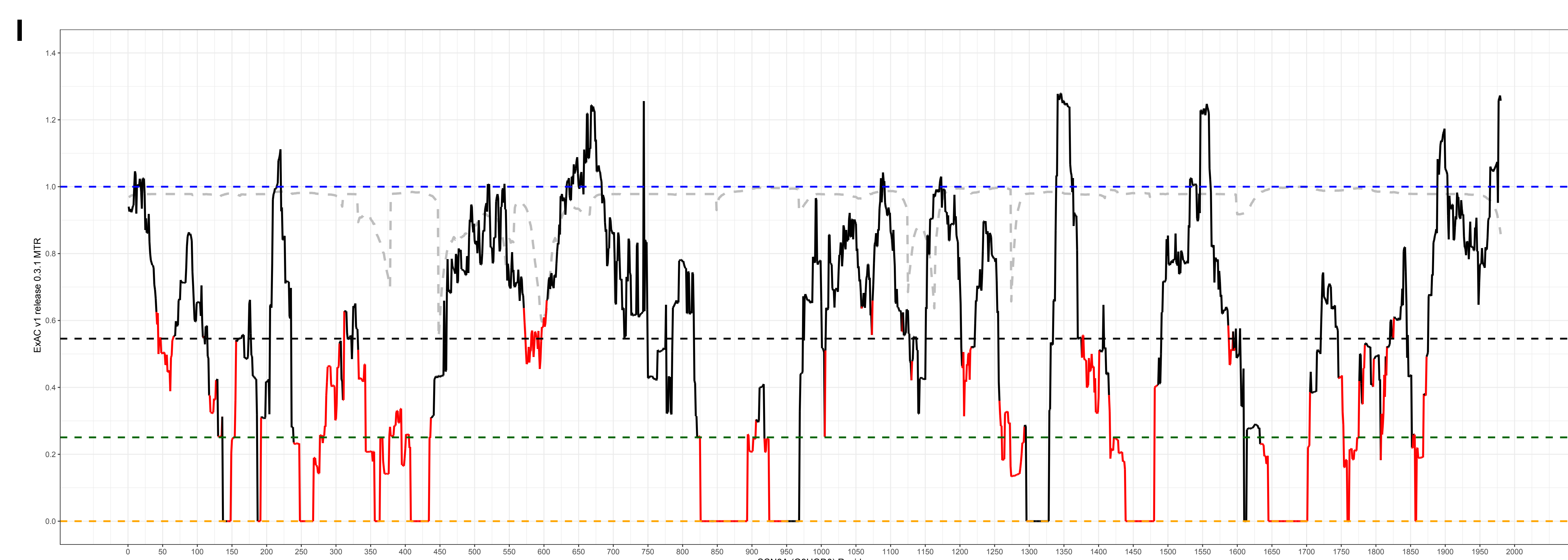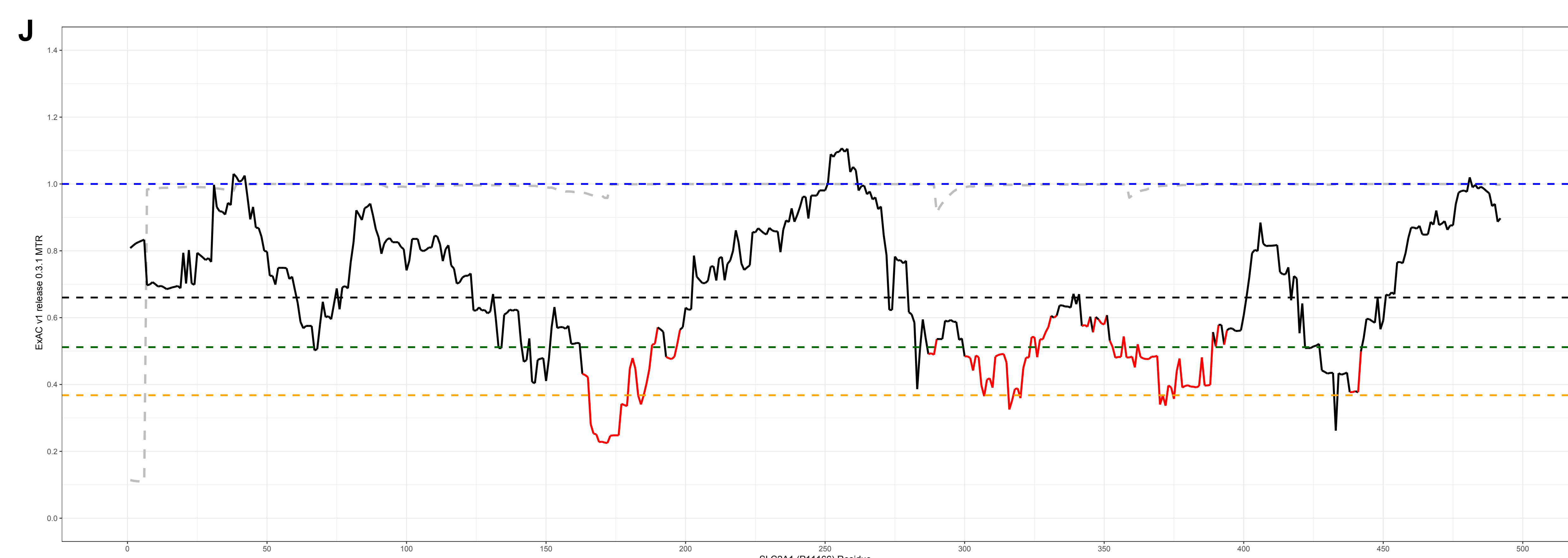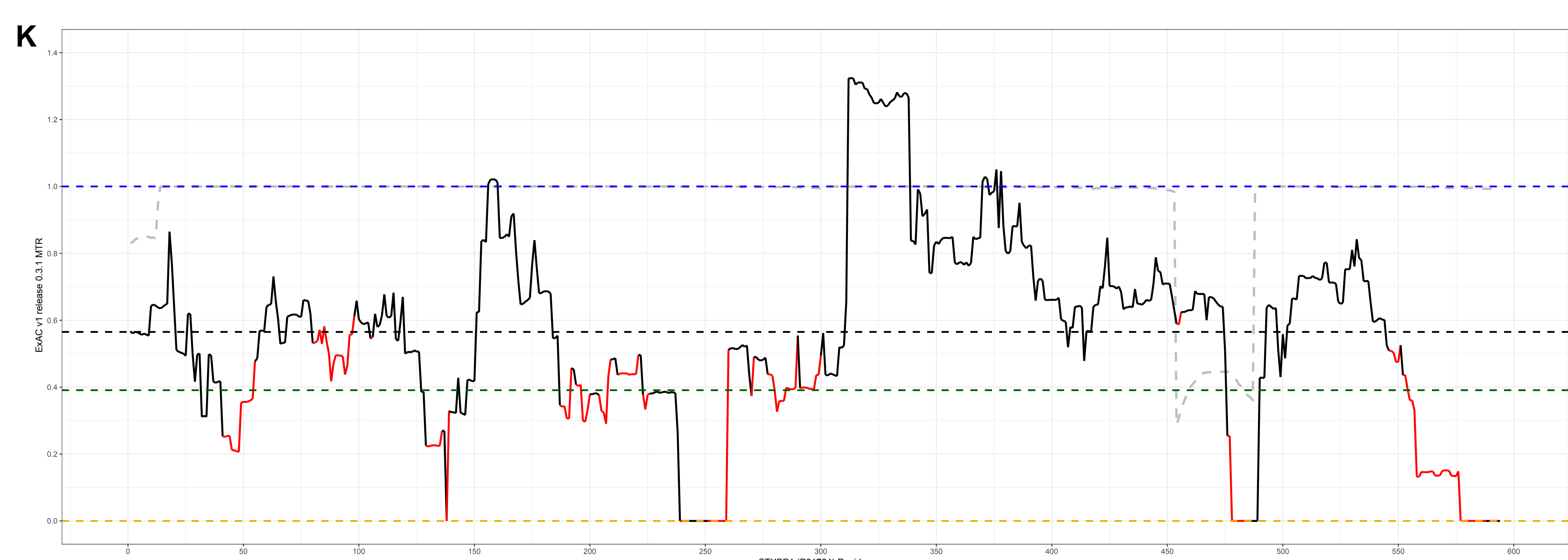

Supplement: Supplemental Material [file supp_gr.226589.117_Supplemental_Fig_S2.pdf]

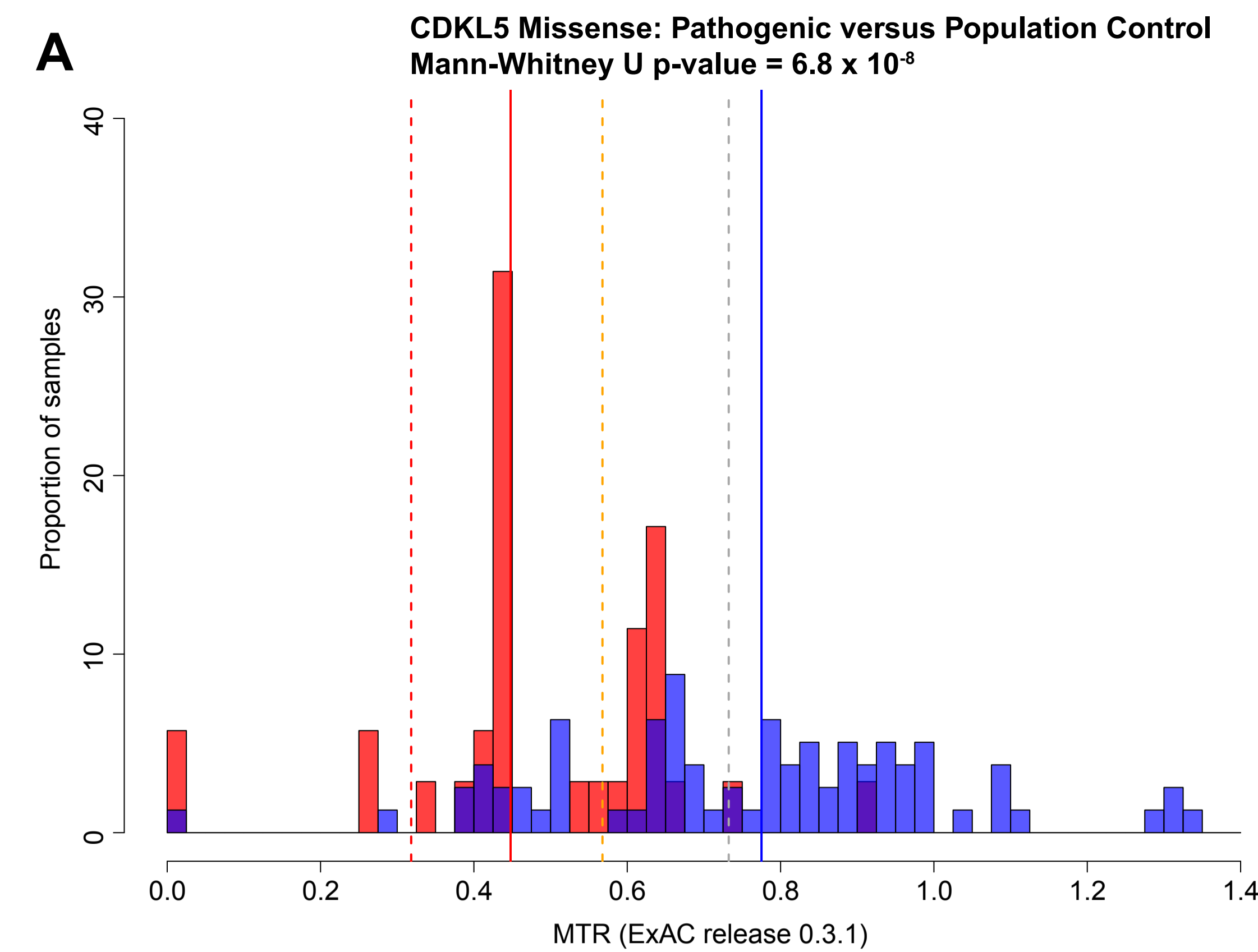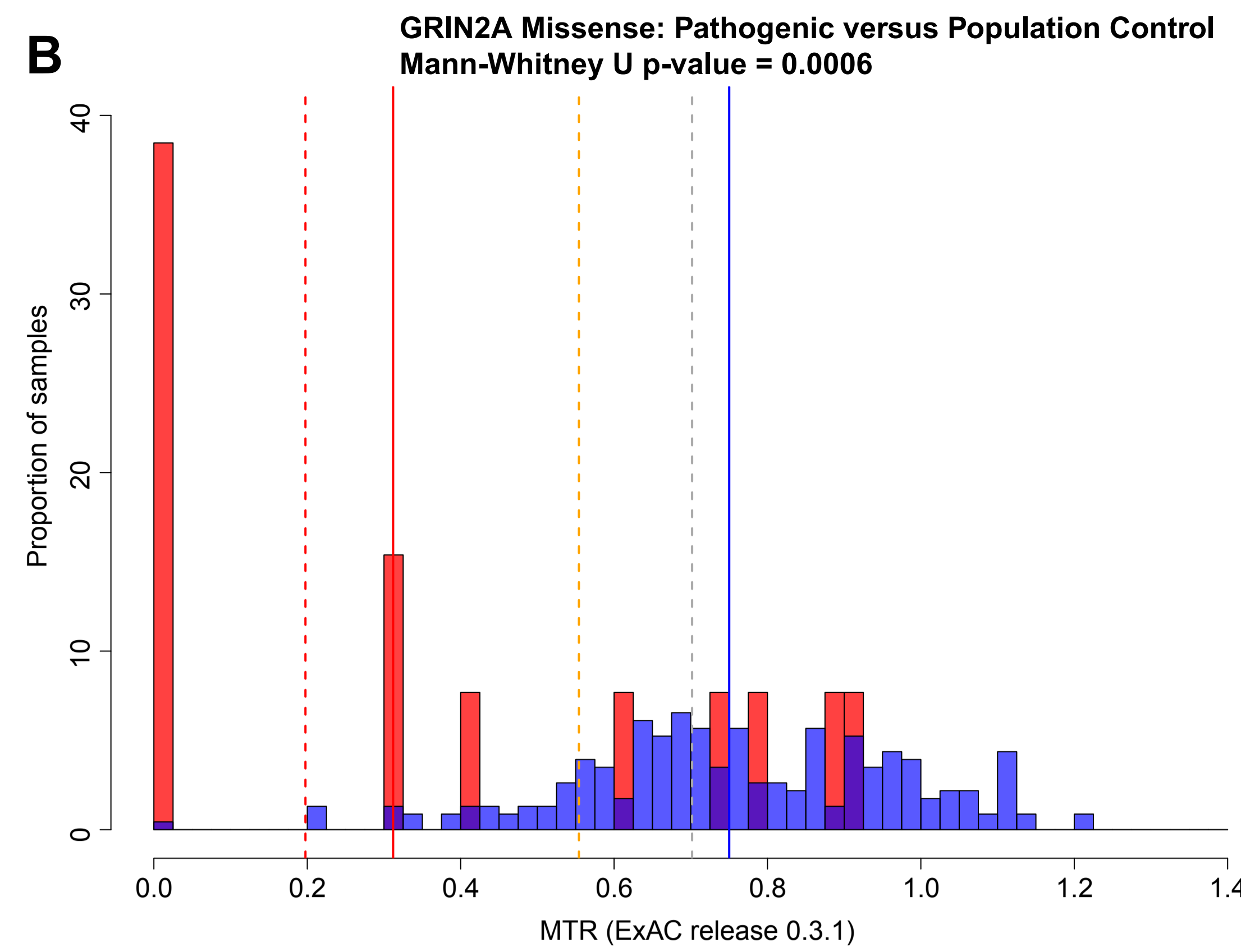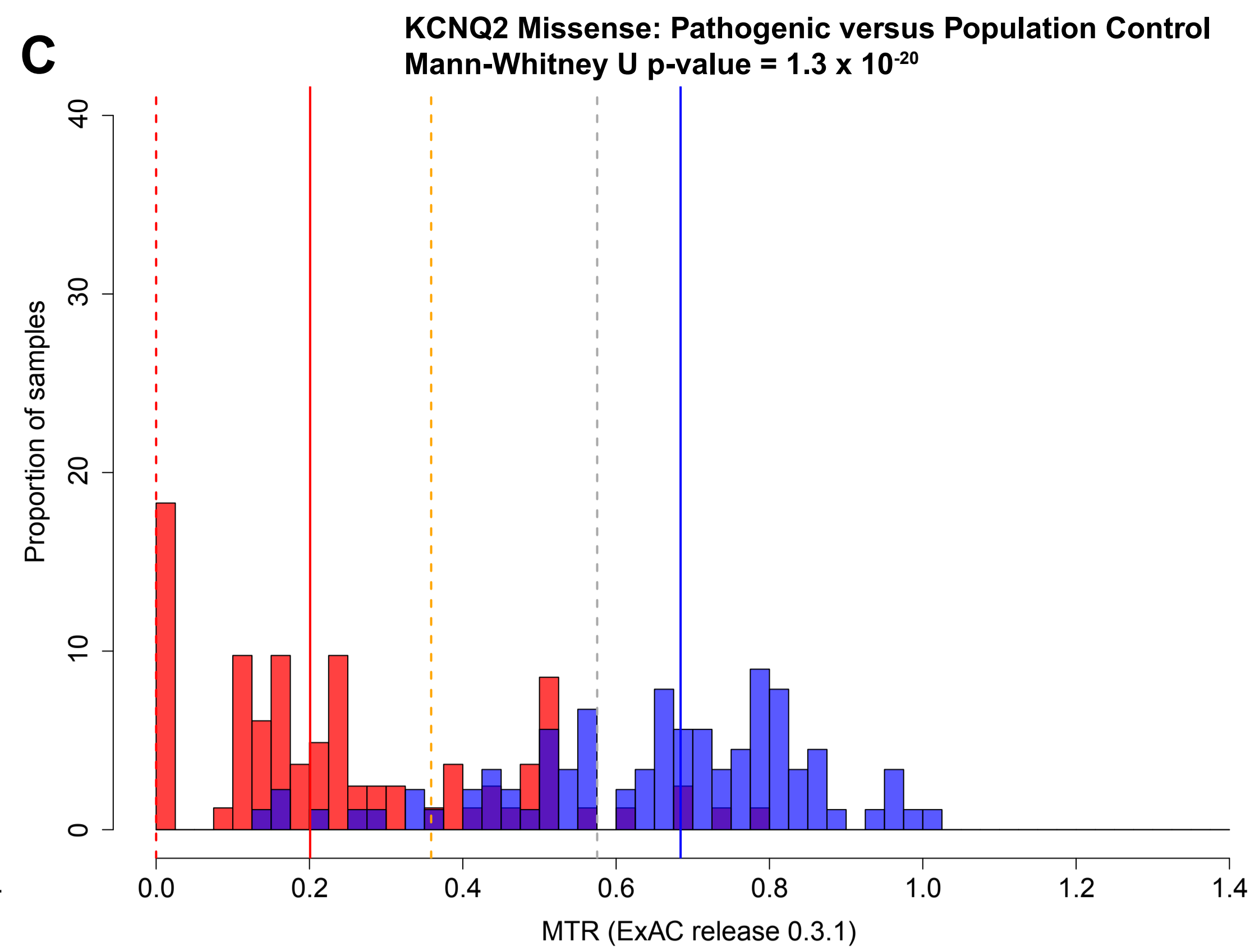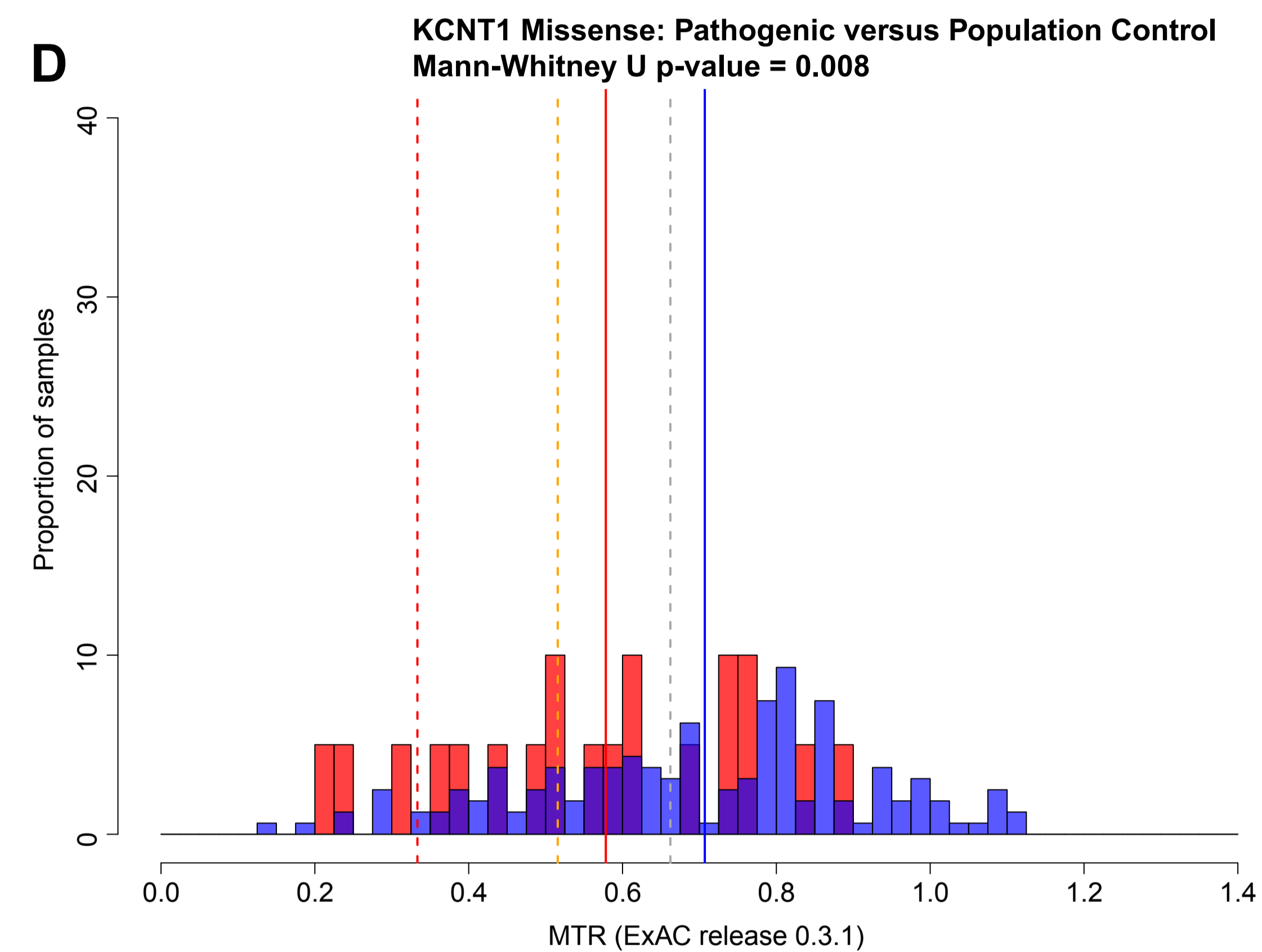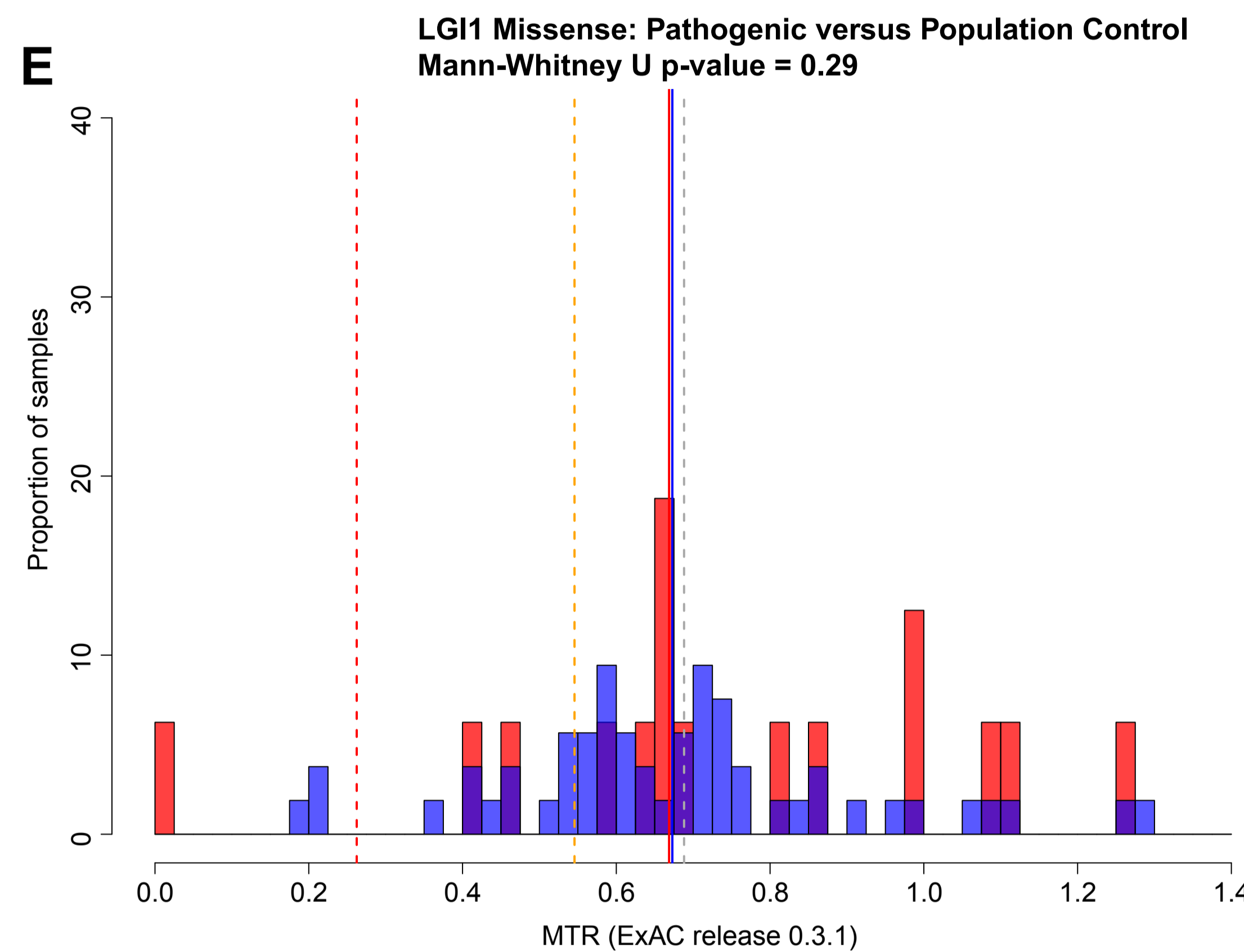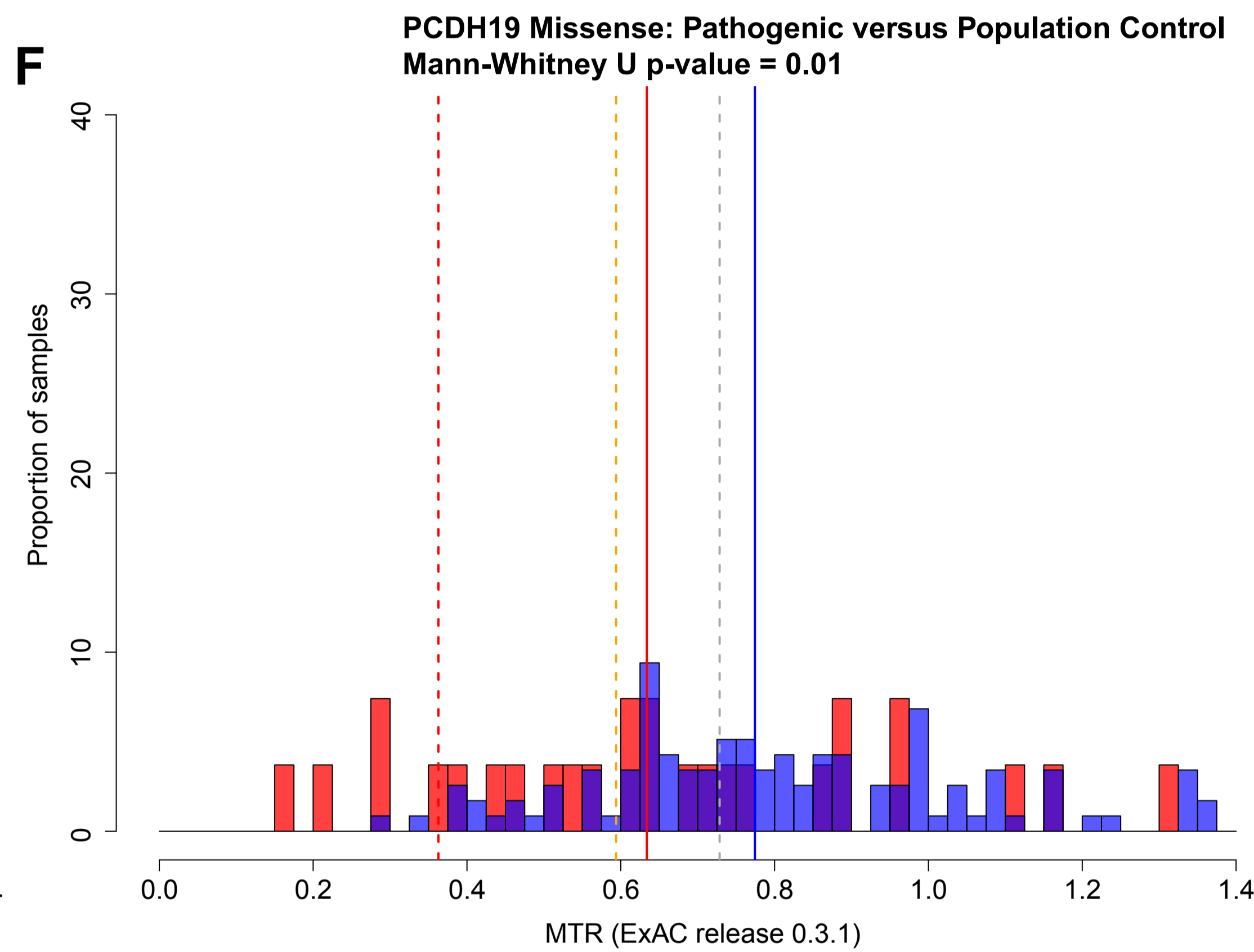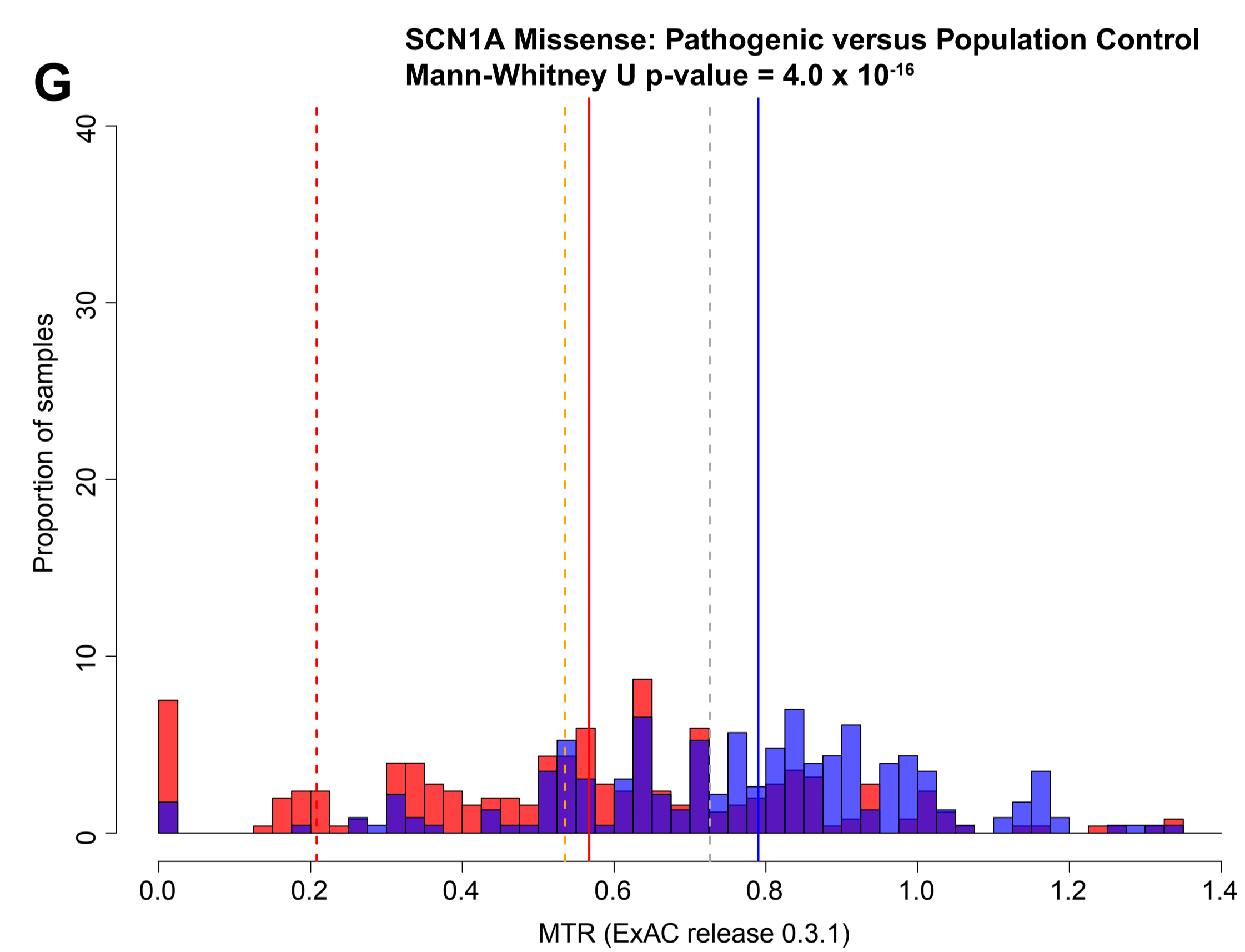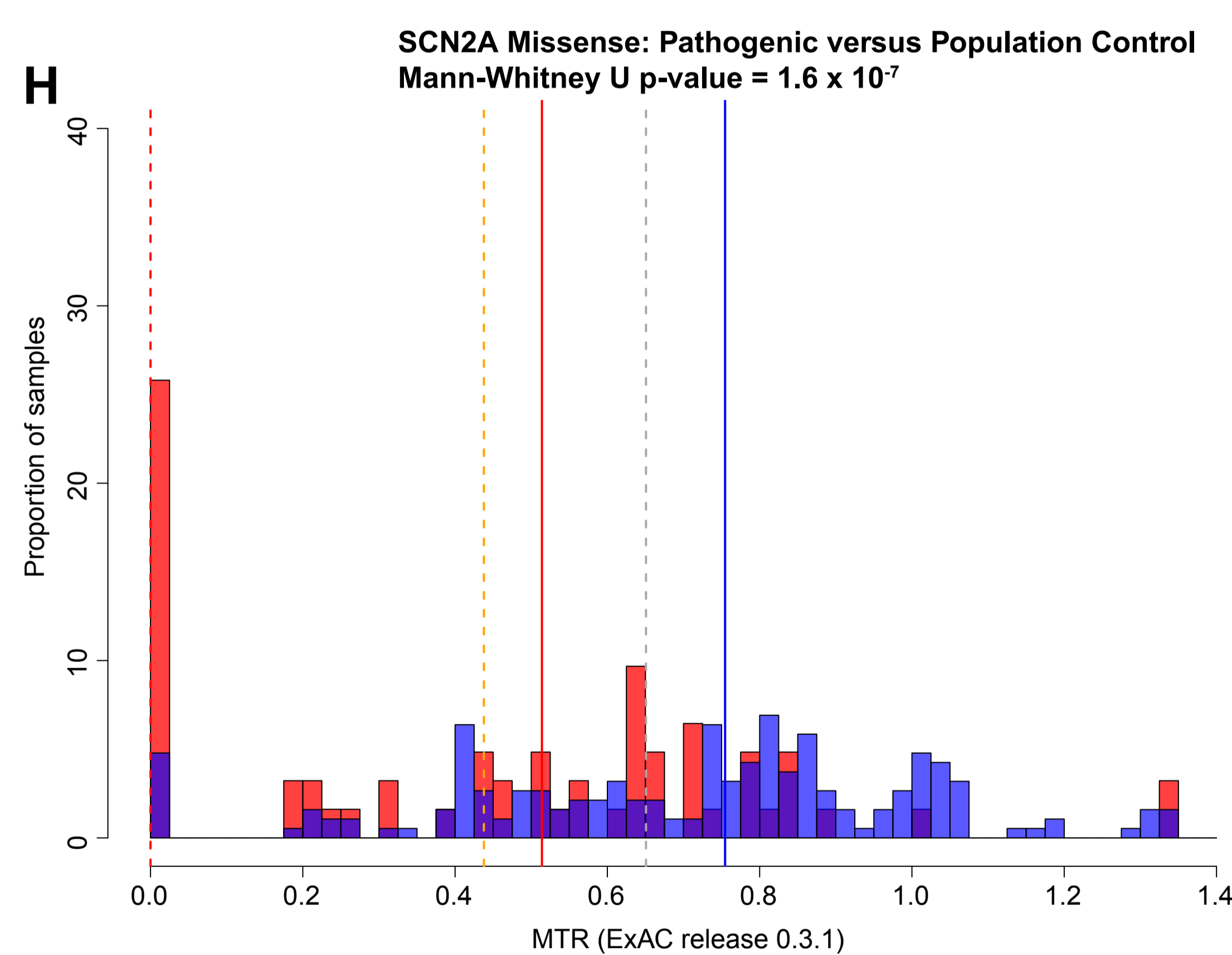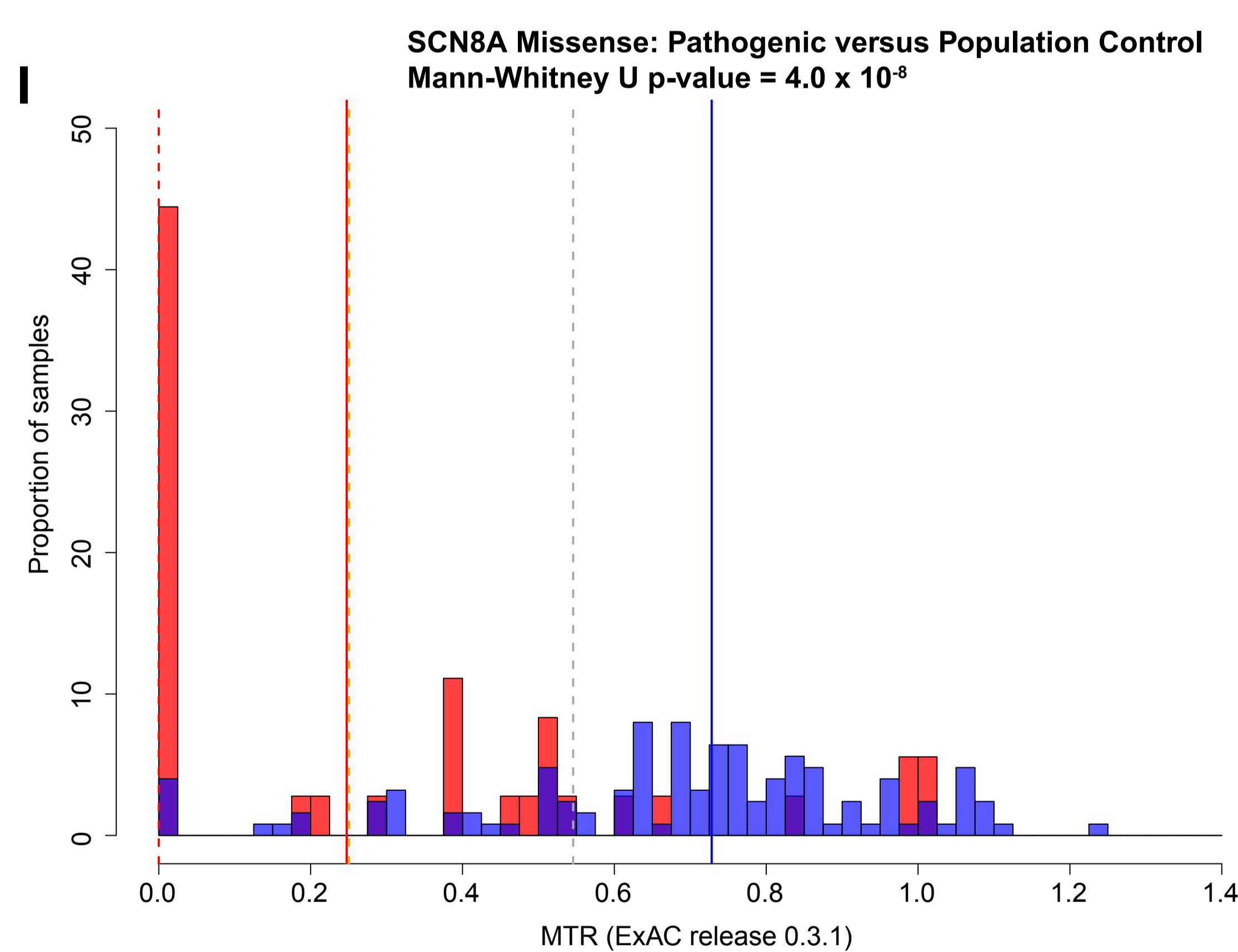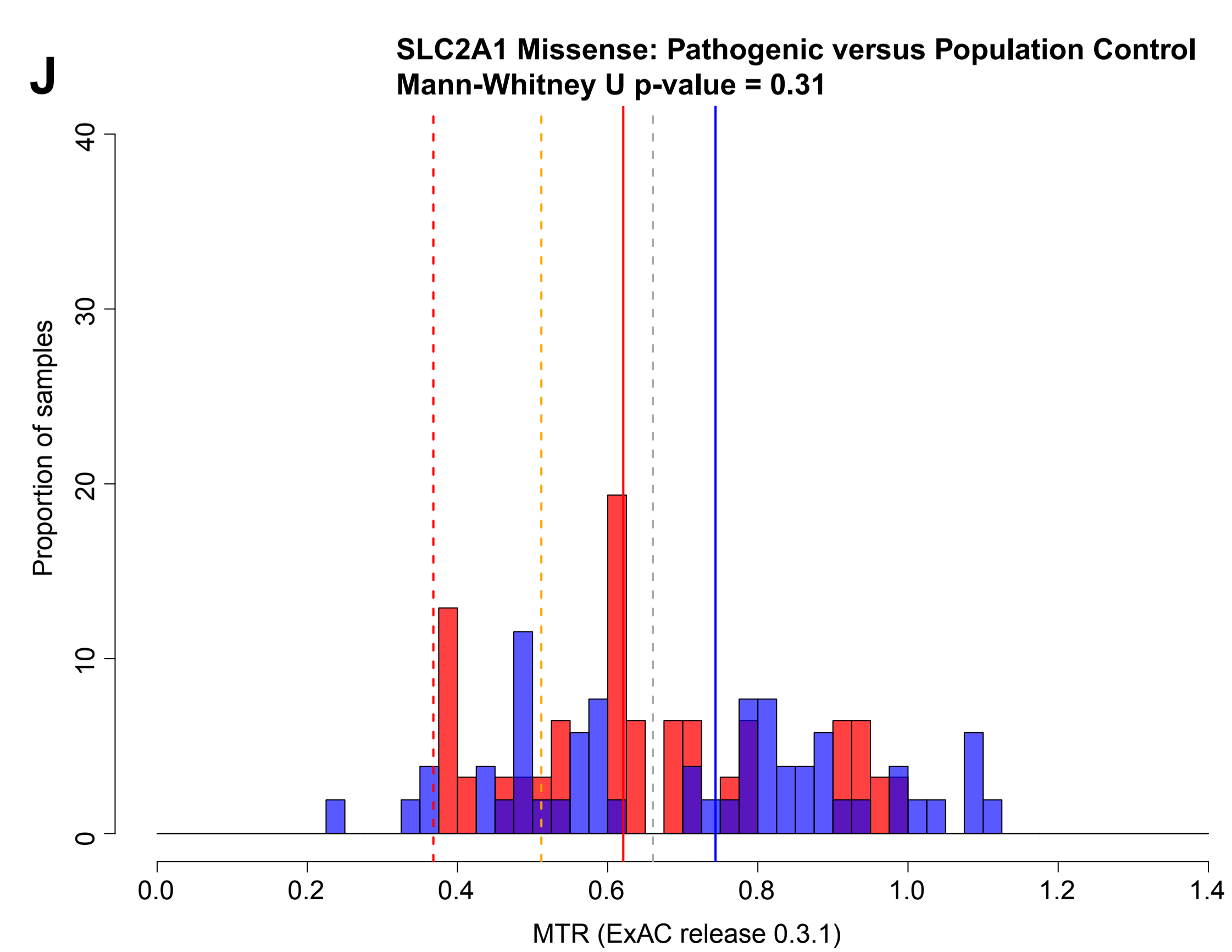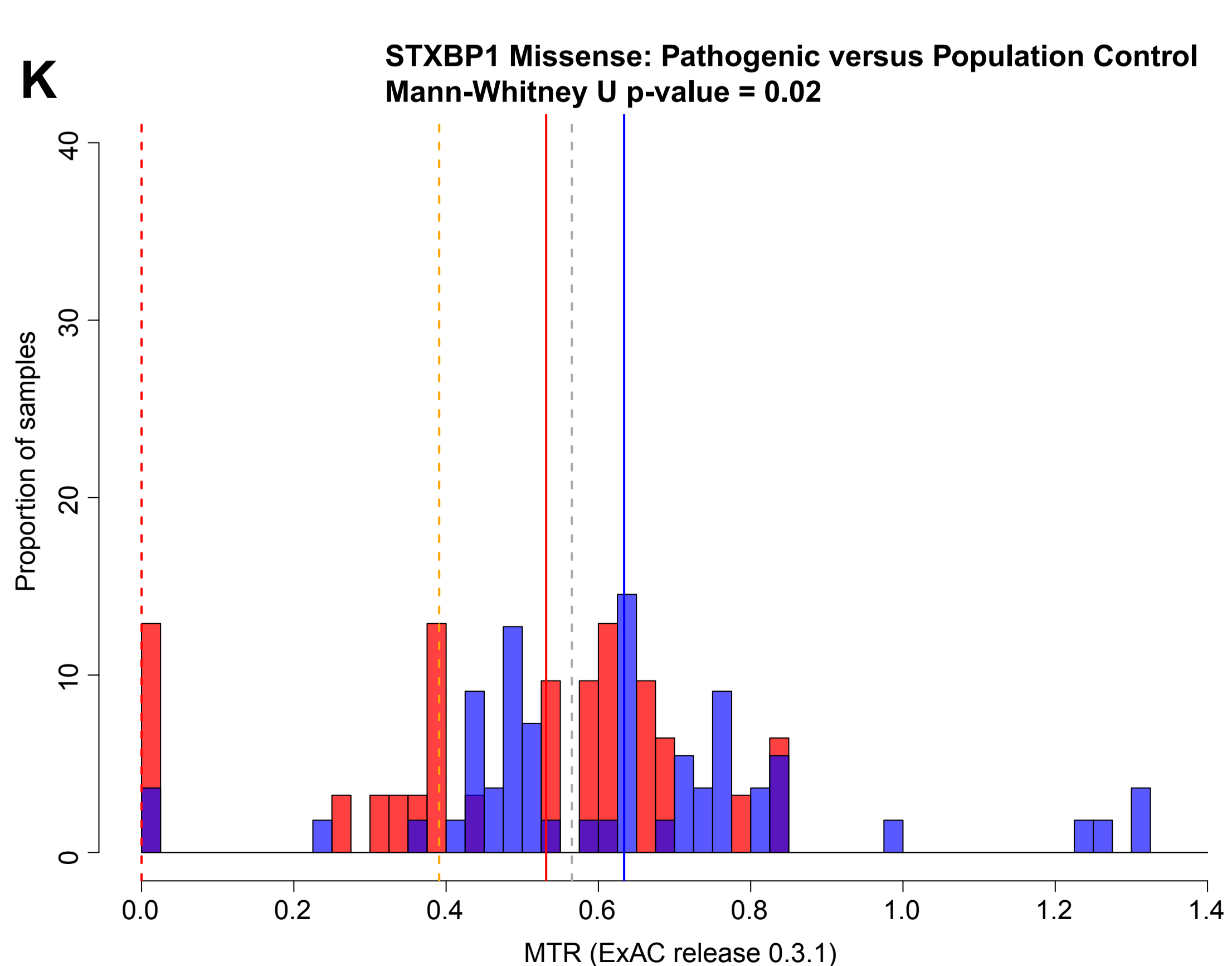

Supplement: Supplemental Material [file supp_gr.226589.117_Supplemental_Fig_S3.pdf]

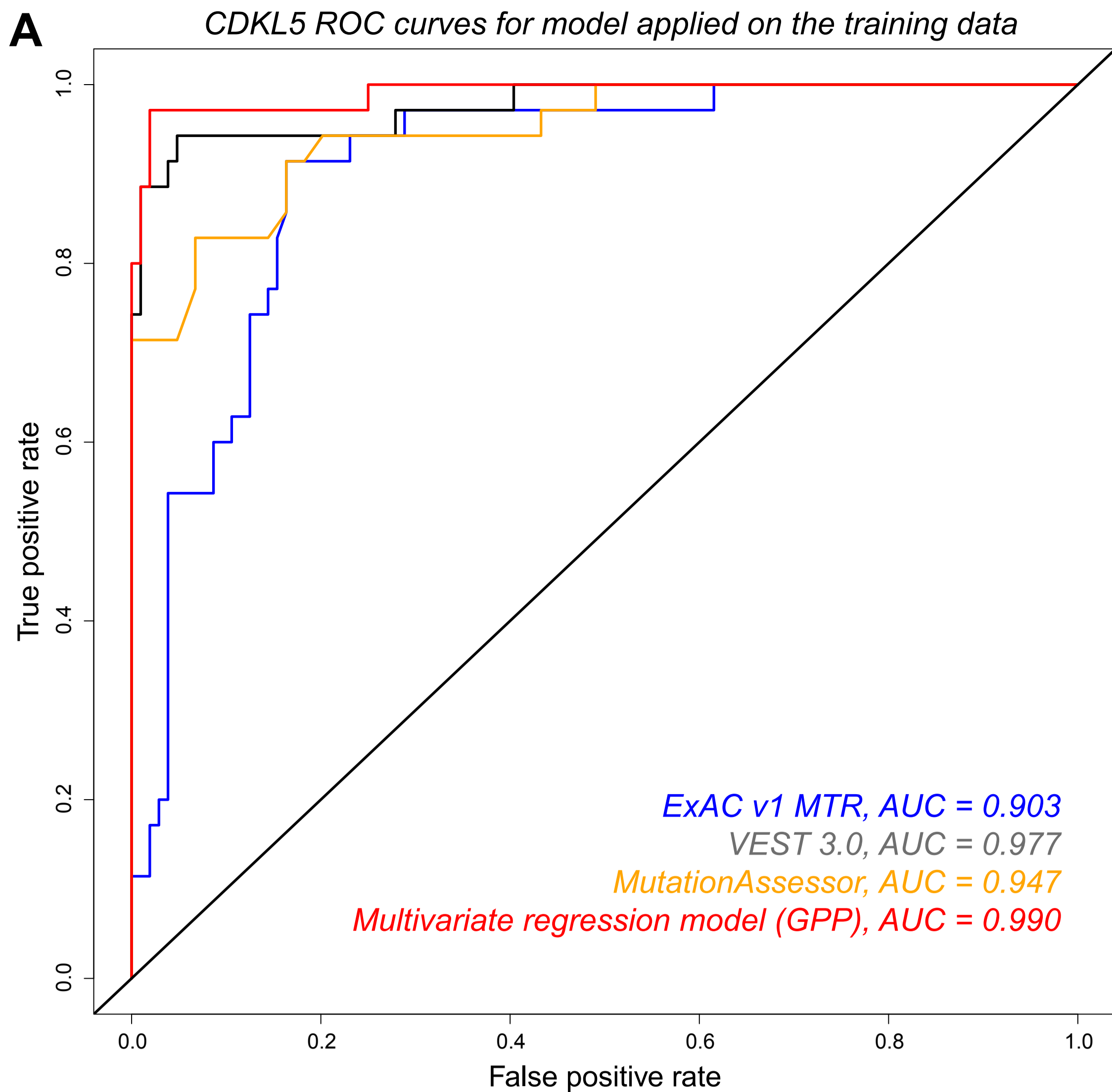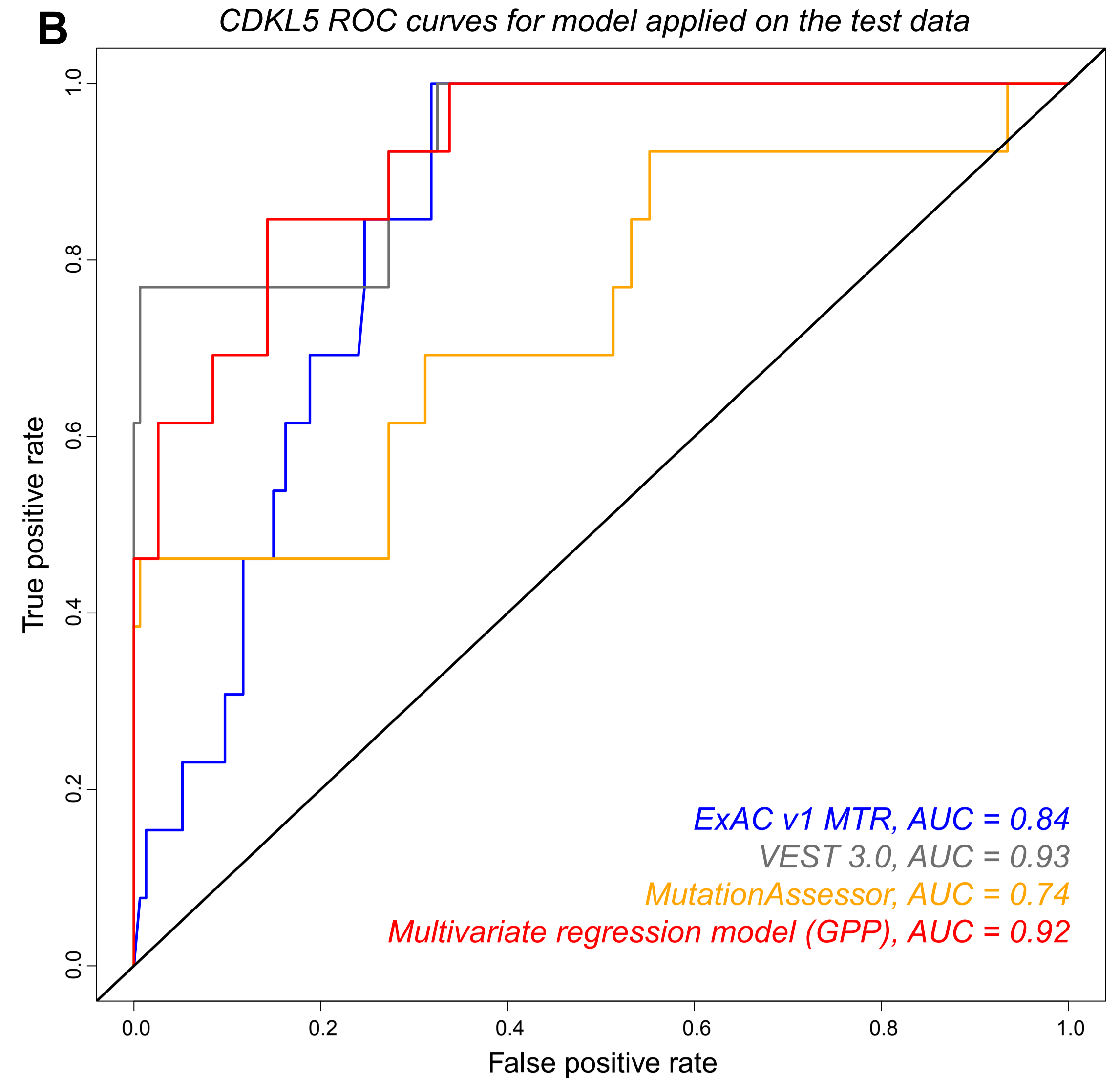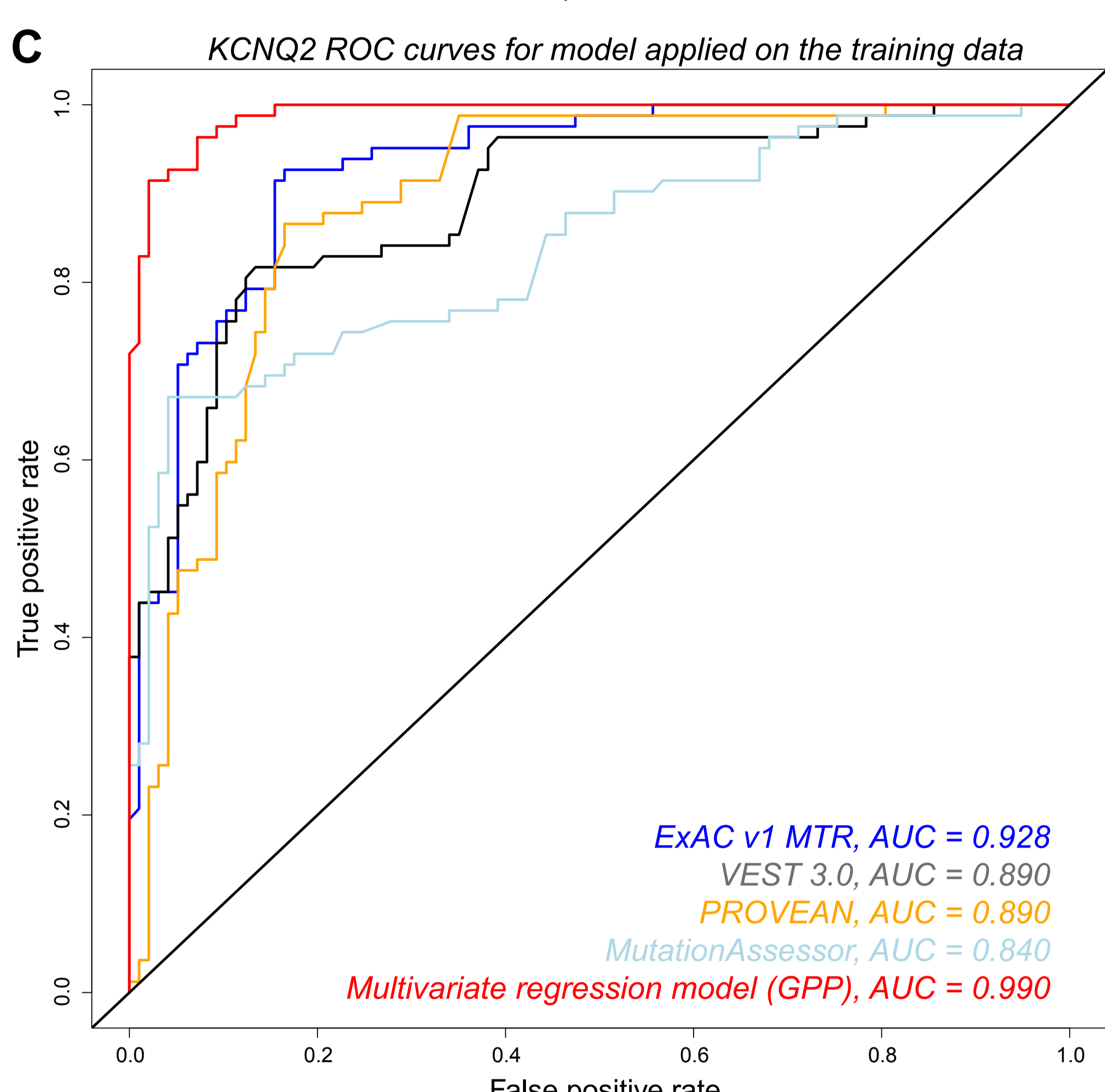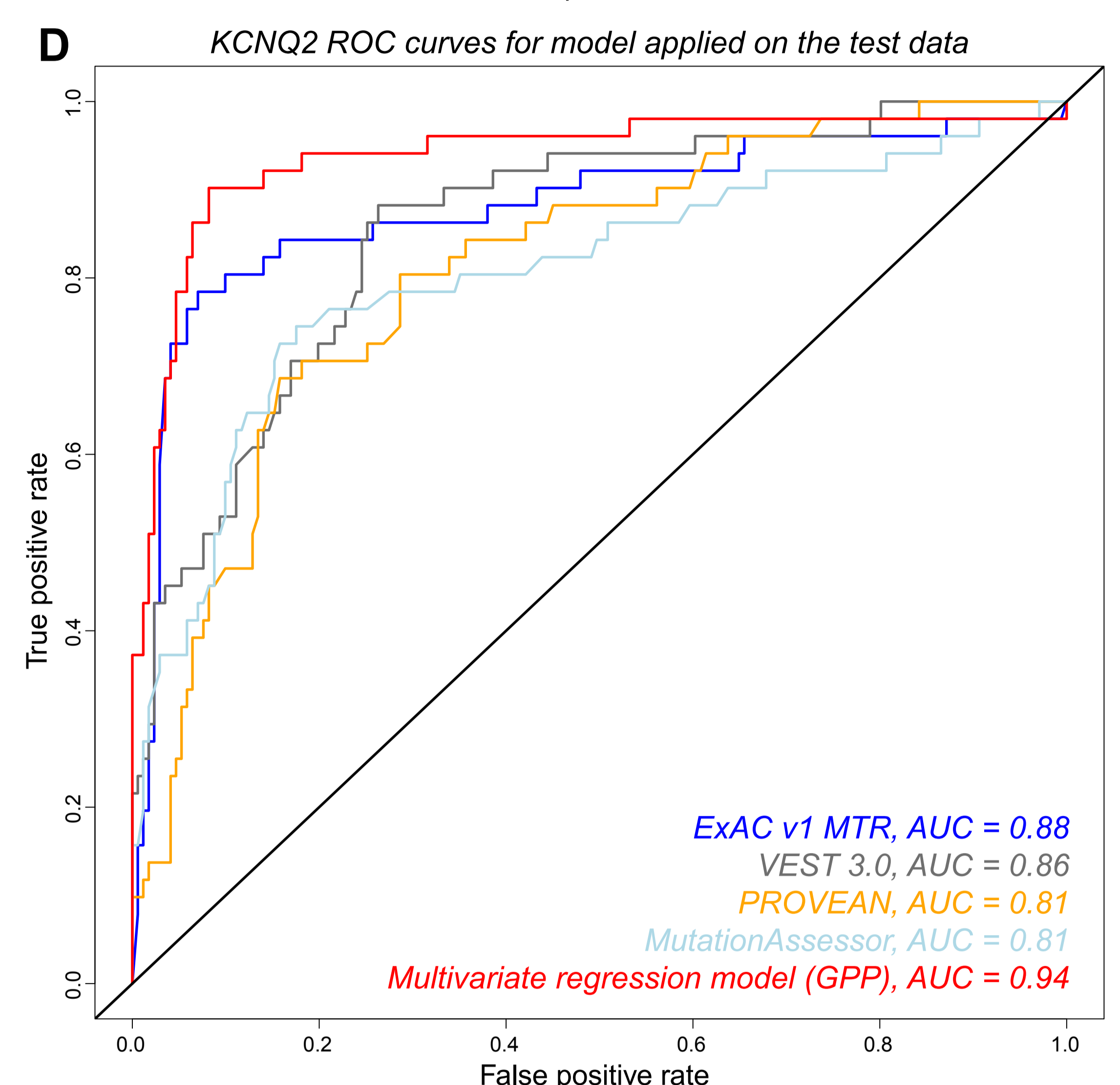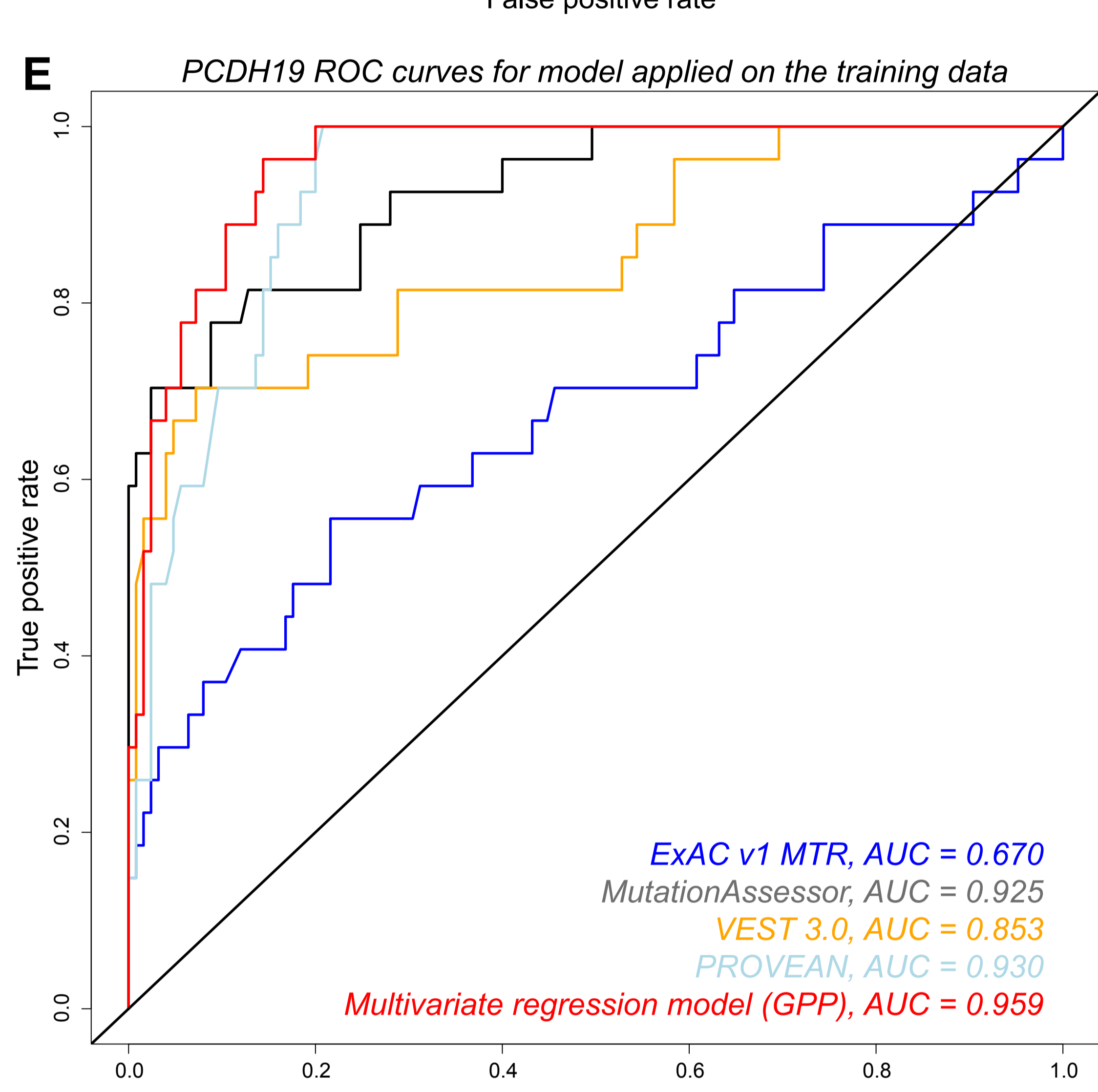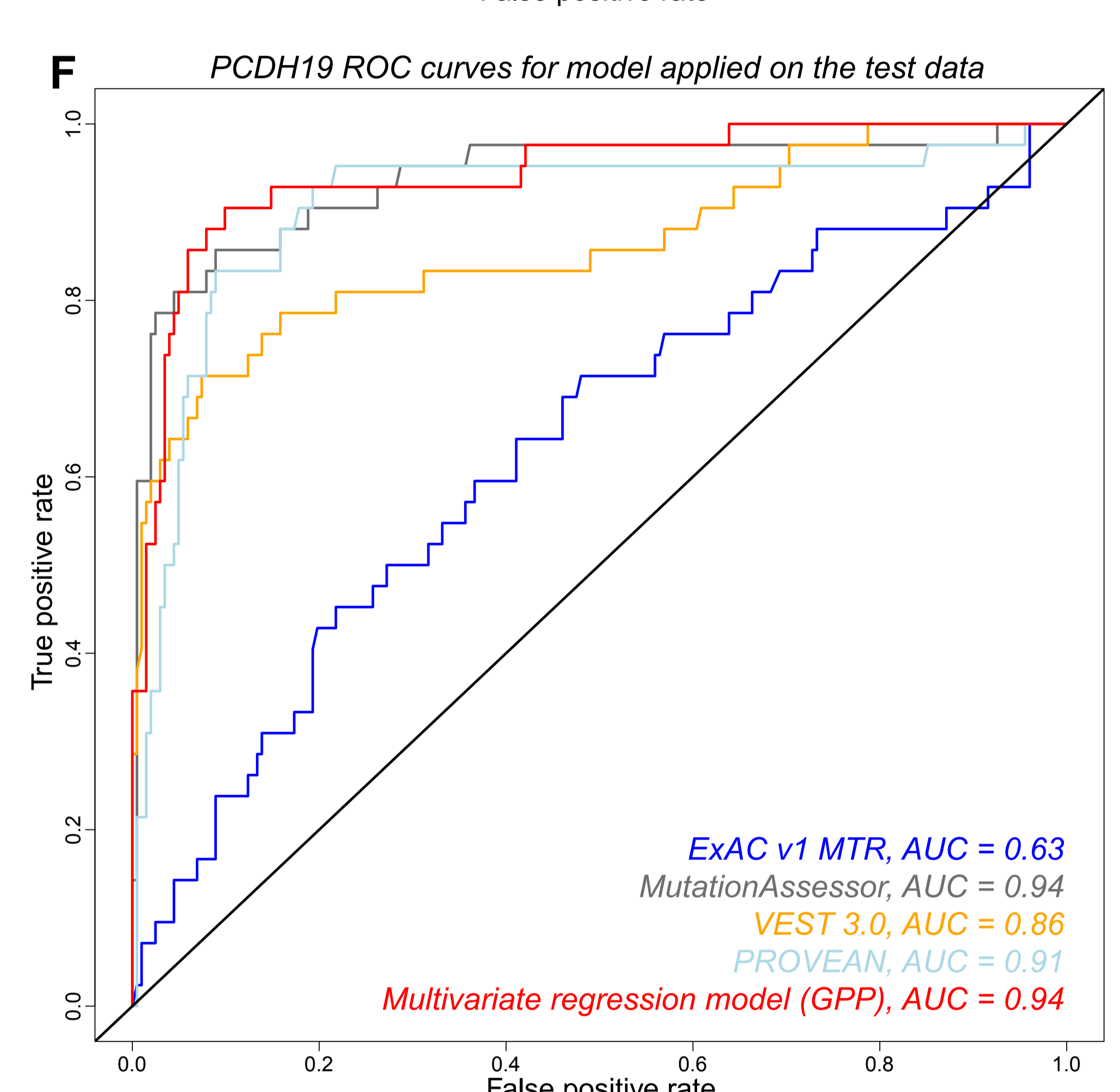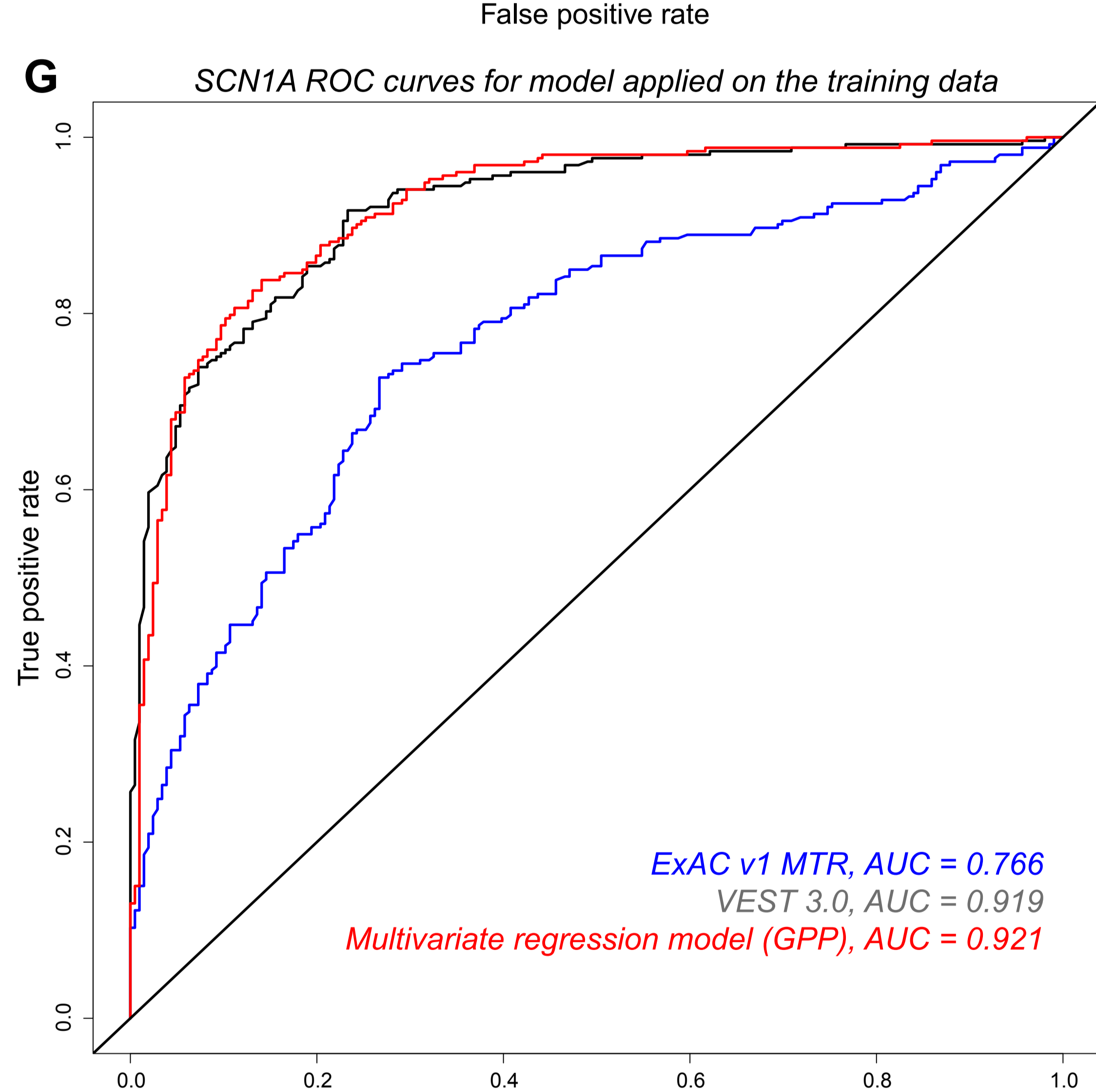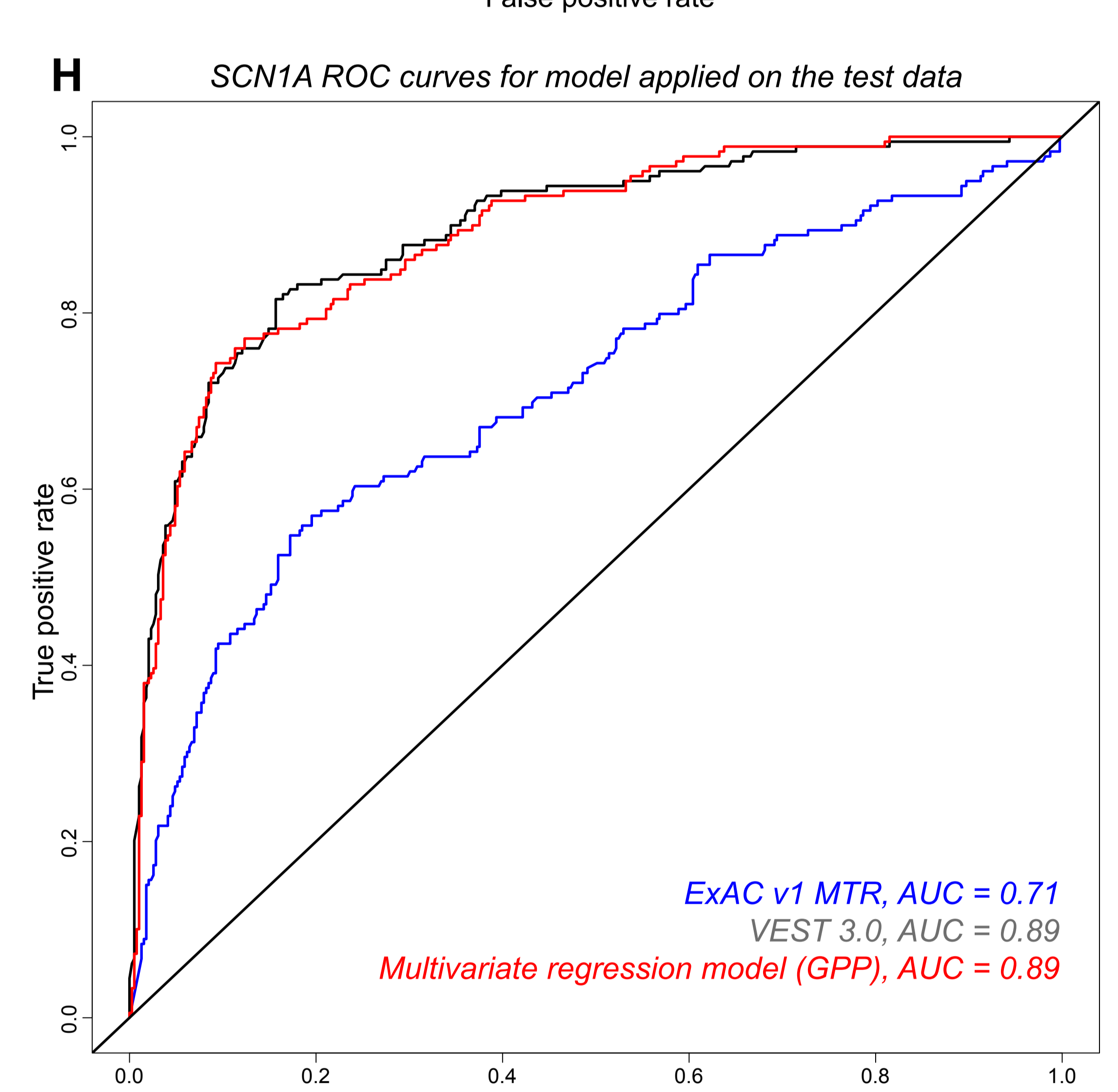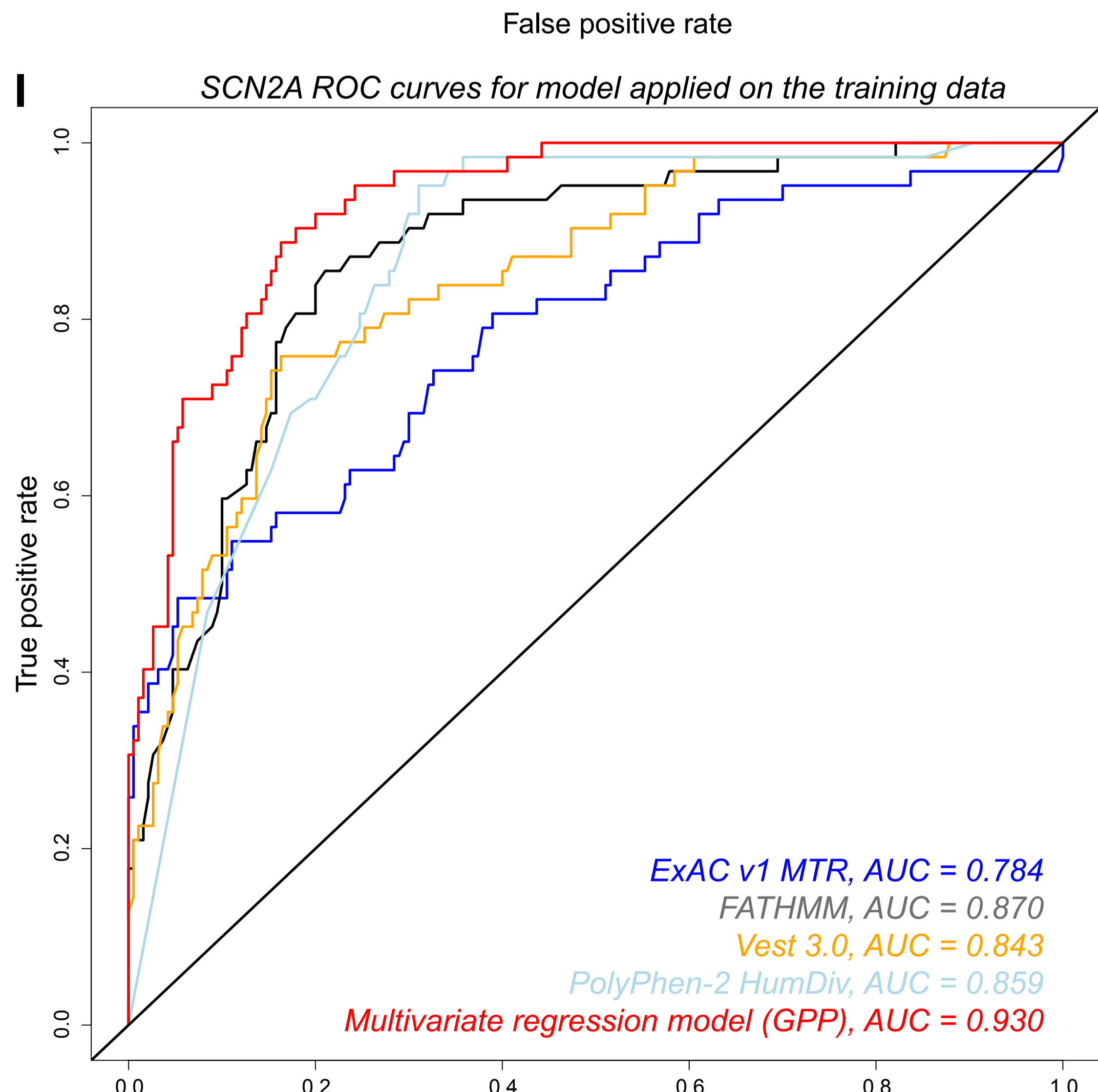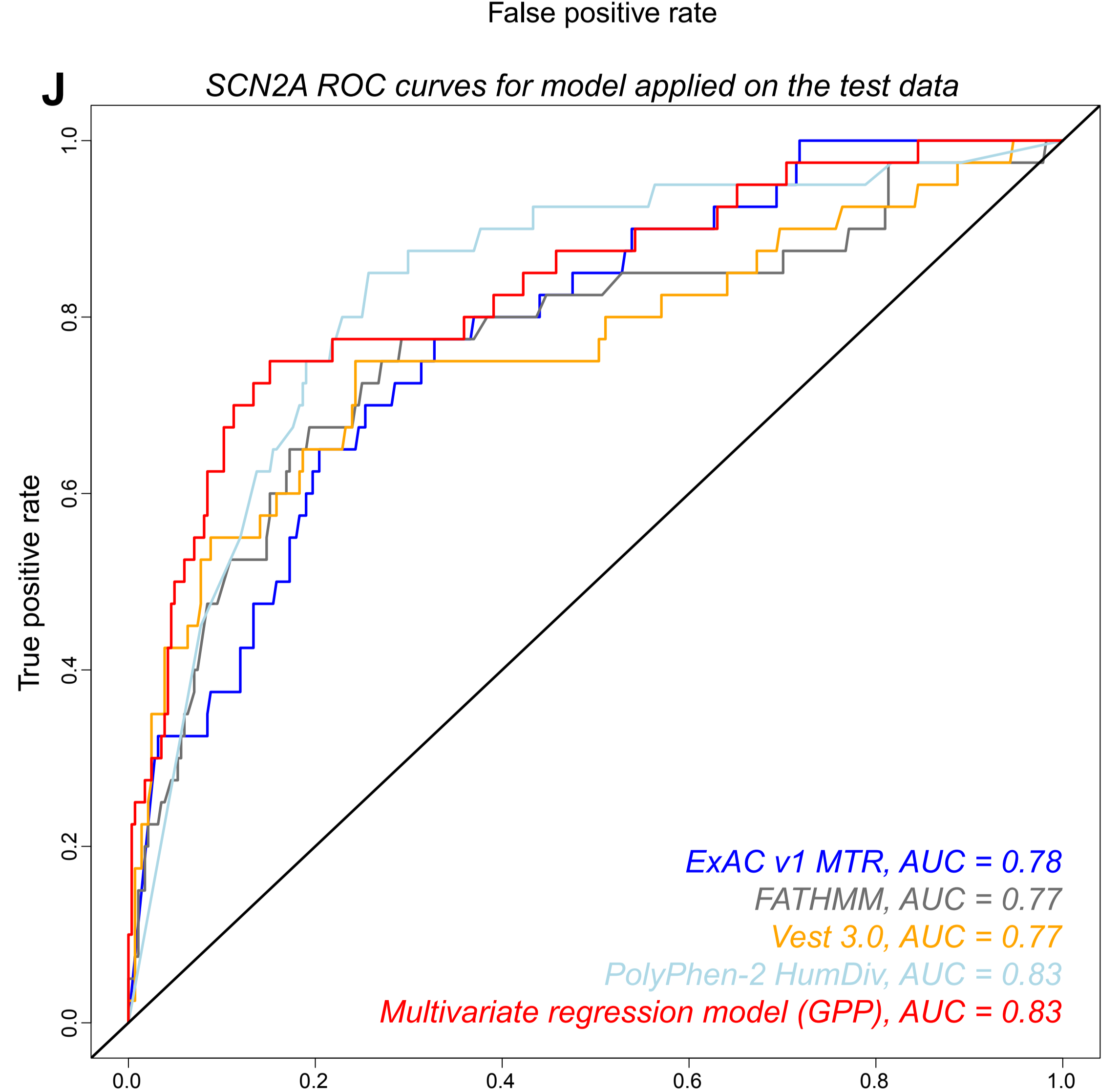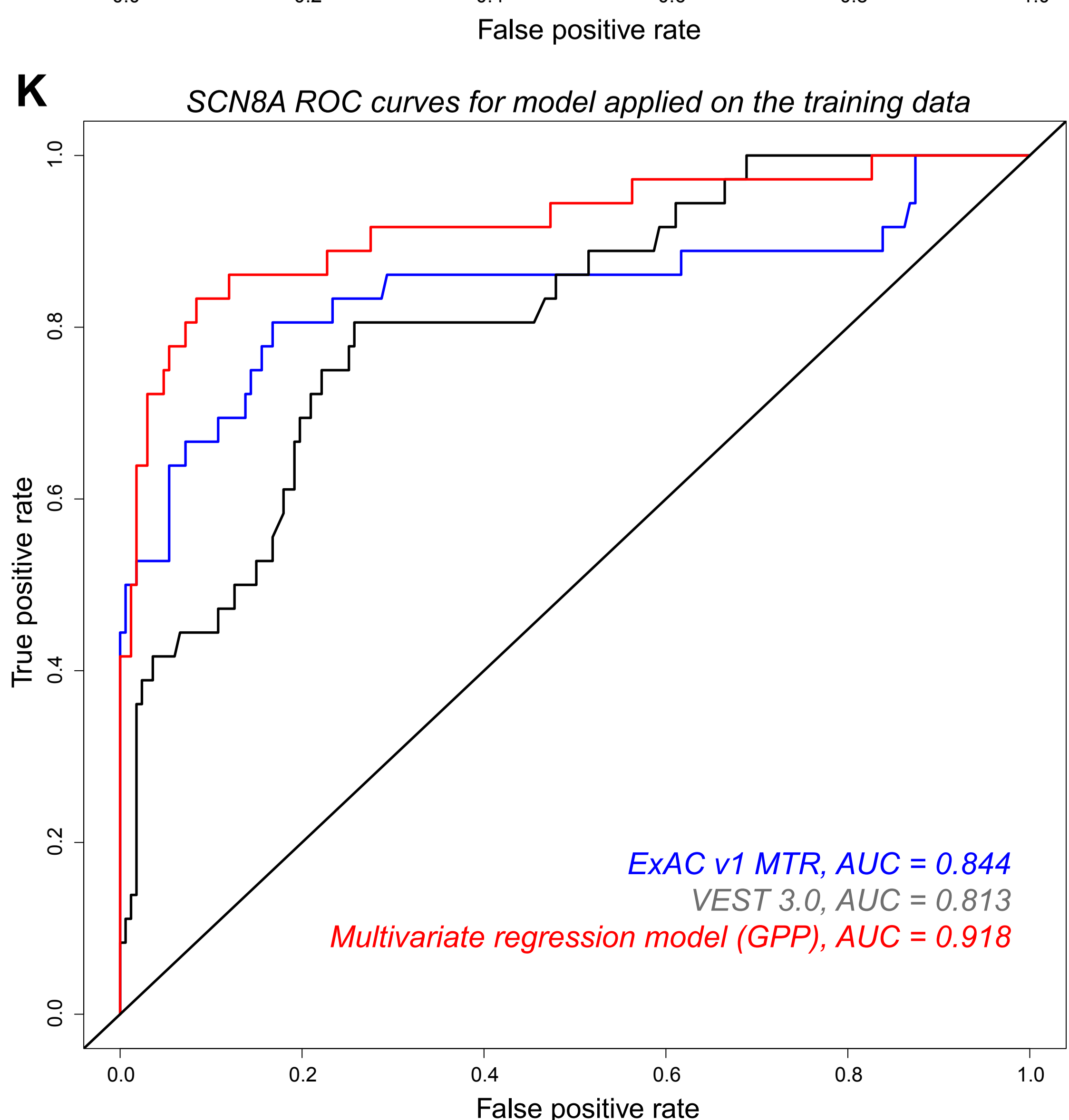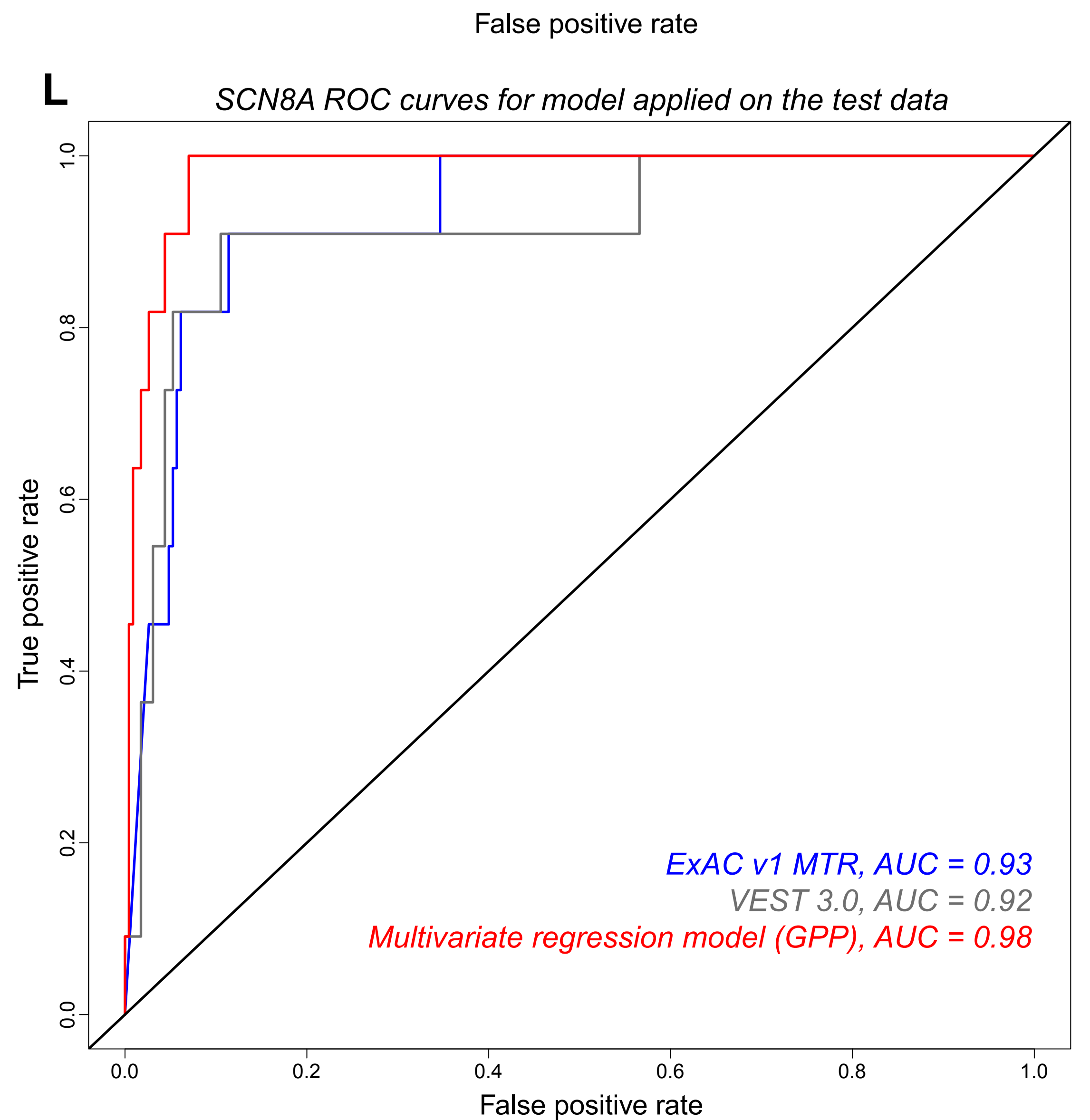

Supplement: Supplemental Material [file supp_gr.226589.117_Supplemental_Fig_S6.pdf]
