# Supplementary material for: Optimizing genomic medicine in epilepsy through a gene-customized approach to missense variant interpretation
Source: Genome Res. 2017 Oct;27(10):1715–29. doi: 10.1101/gr.226589.117 (PMC5630035; doi:10.1101/gr.226589.117)
Supplement: Supplemental Material [file supp_gr.226589.117_Supplemental_Fig_S5.pdf]

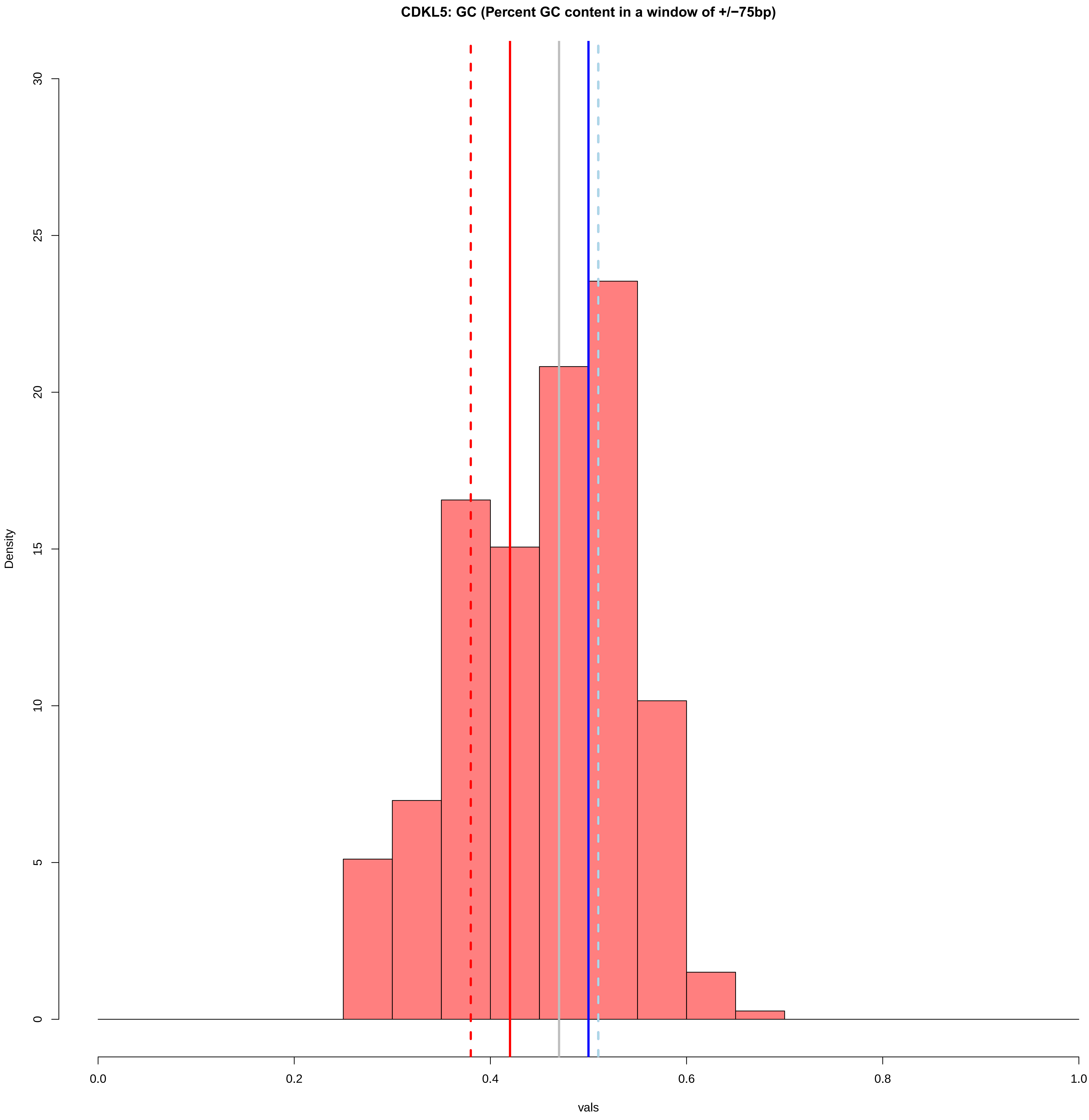
CDKL5: GC (Percent GC content in a window of +/−75bp)
Density
0
5
10
15
20
25
30
0.0
0.2
0.4
0.6
0.8
1.0
vals

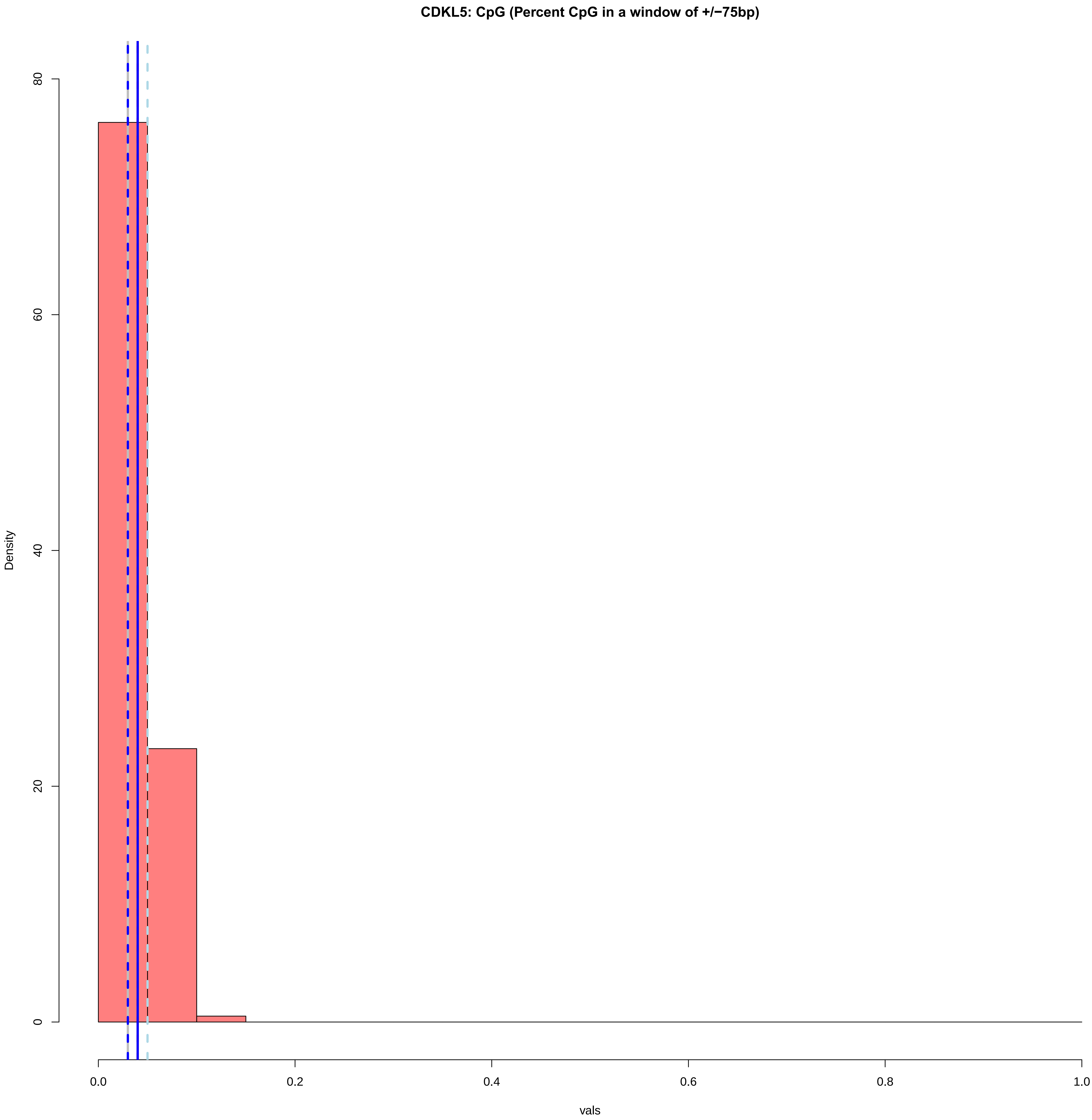
CDKL5: CpG (Percent CpG in a window of +/−75bp)
Density
0
20
40
60
80
0.0
0.2
0.4
0.6
0.8
1.0
vals

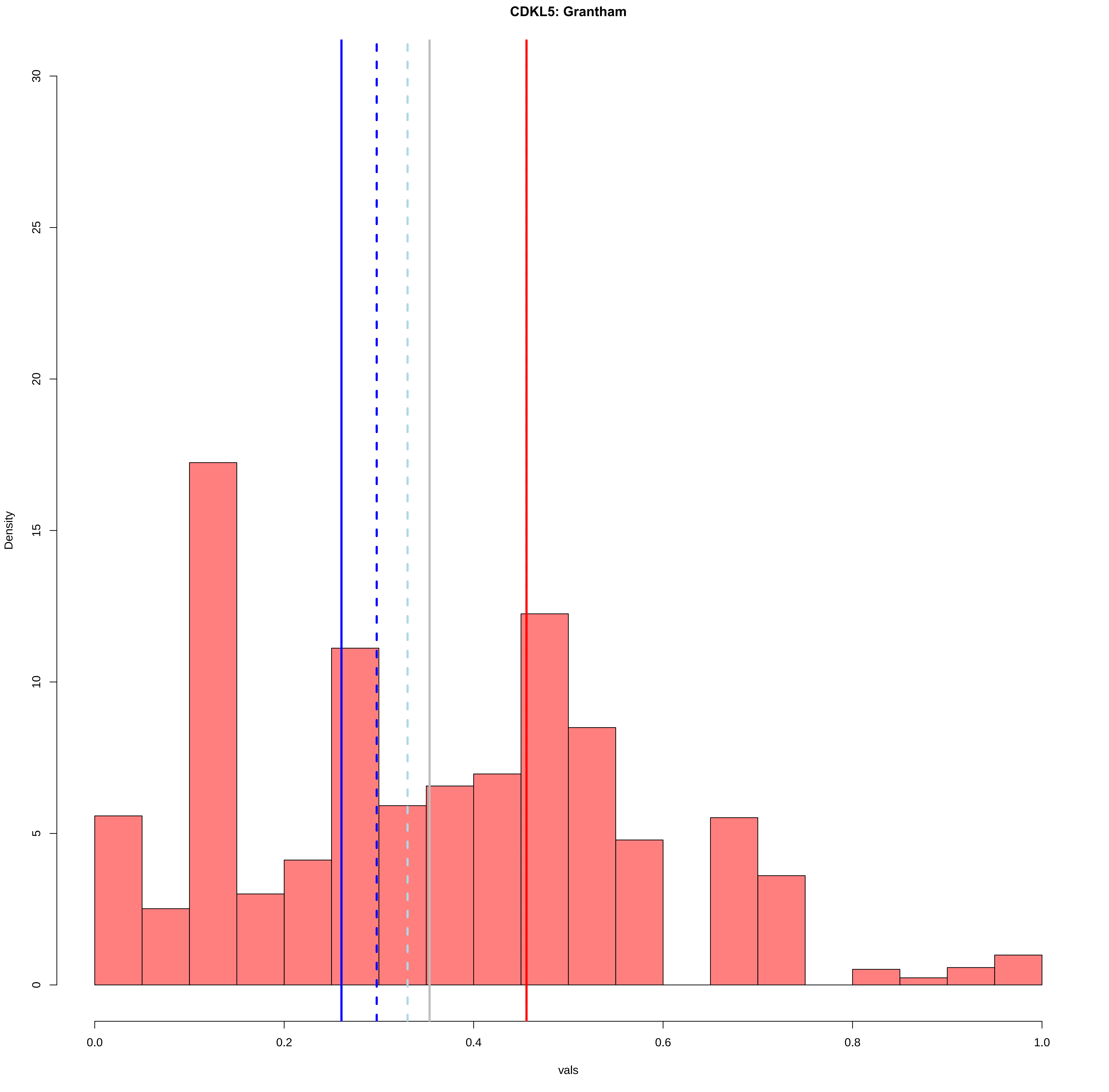
CDKL5: Grantham
Density
vals
0
5
10
15
20
25
30
0.0
0.2
0.4
0.6
0.8
1.0

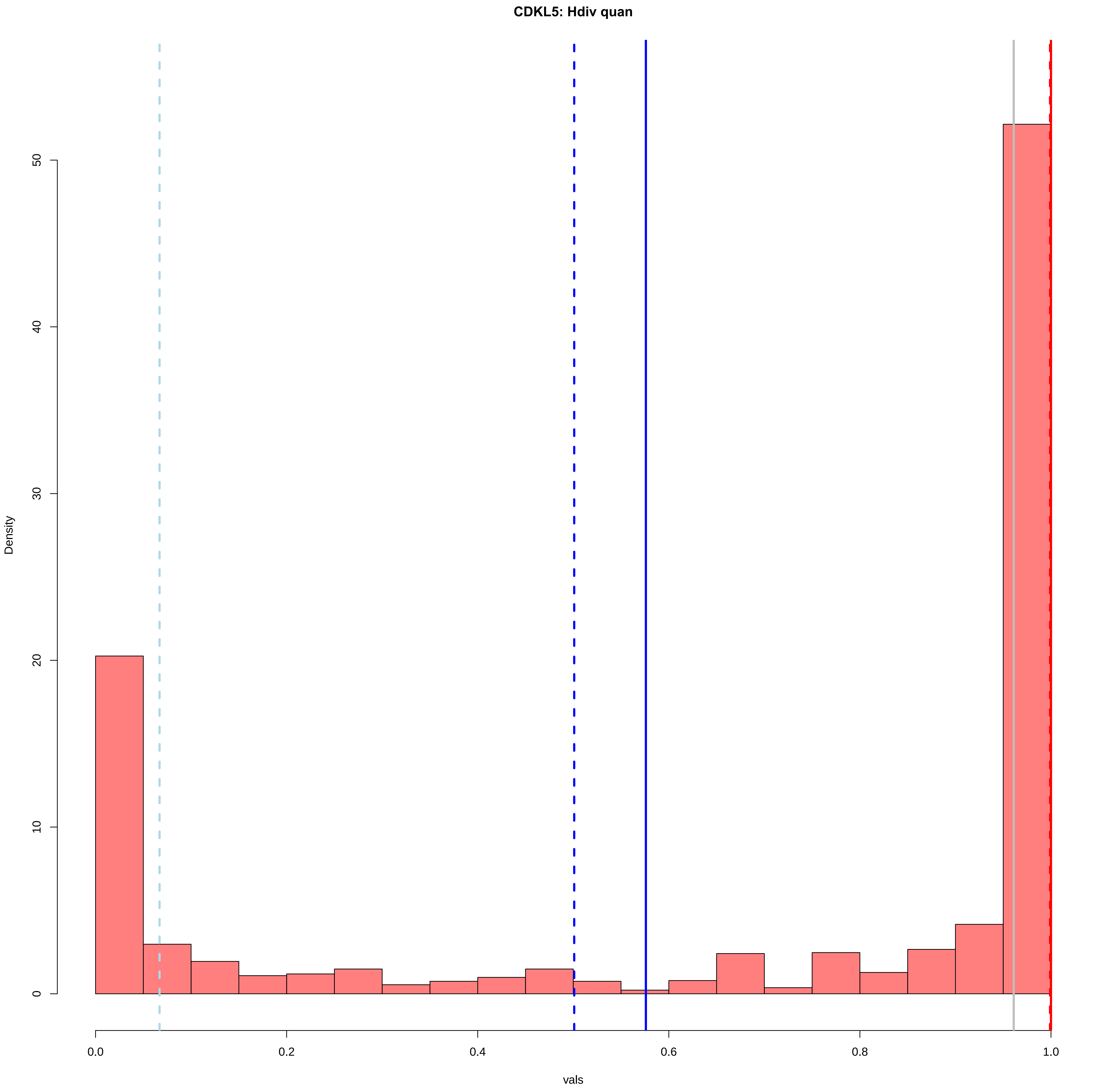
CDKL5: Hdiv quan
Density
vals
0
10
20
30
40
50
0.0
0.2
0.4
0.6
0.8
1.0

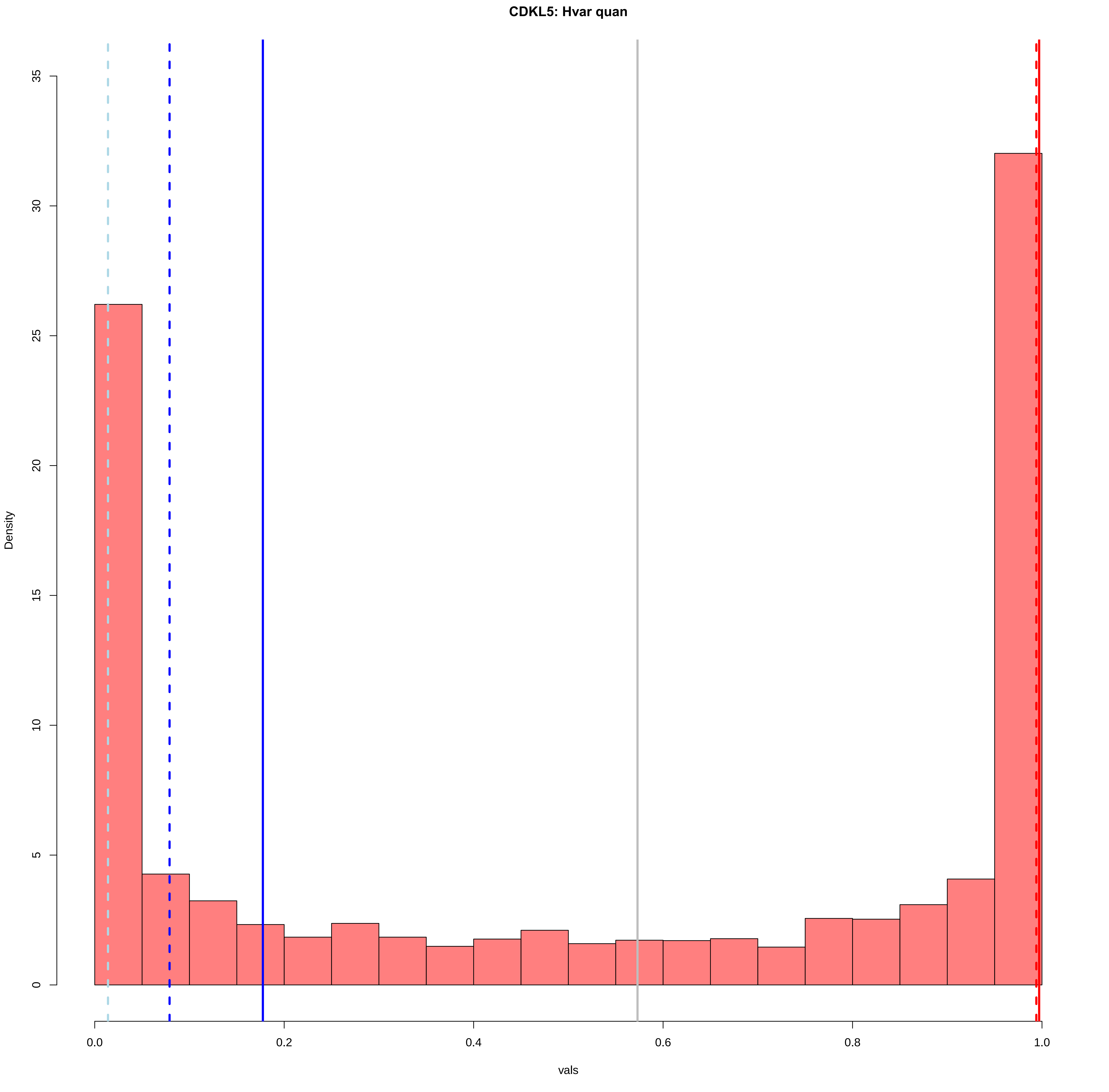

CDKL5: Hvar quan
Density
vals
0
5
10
15
20
25
30
35
0.0
0.2
0.4
0.6
0.8
1.0

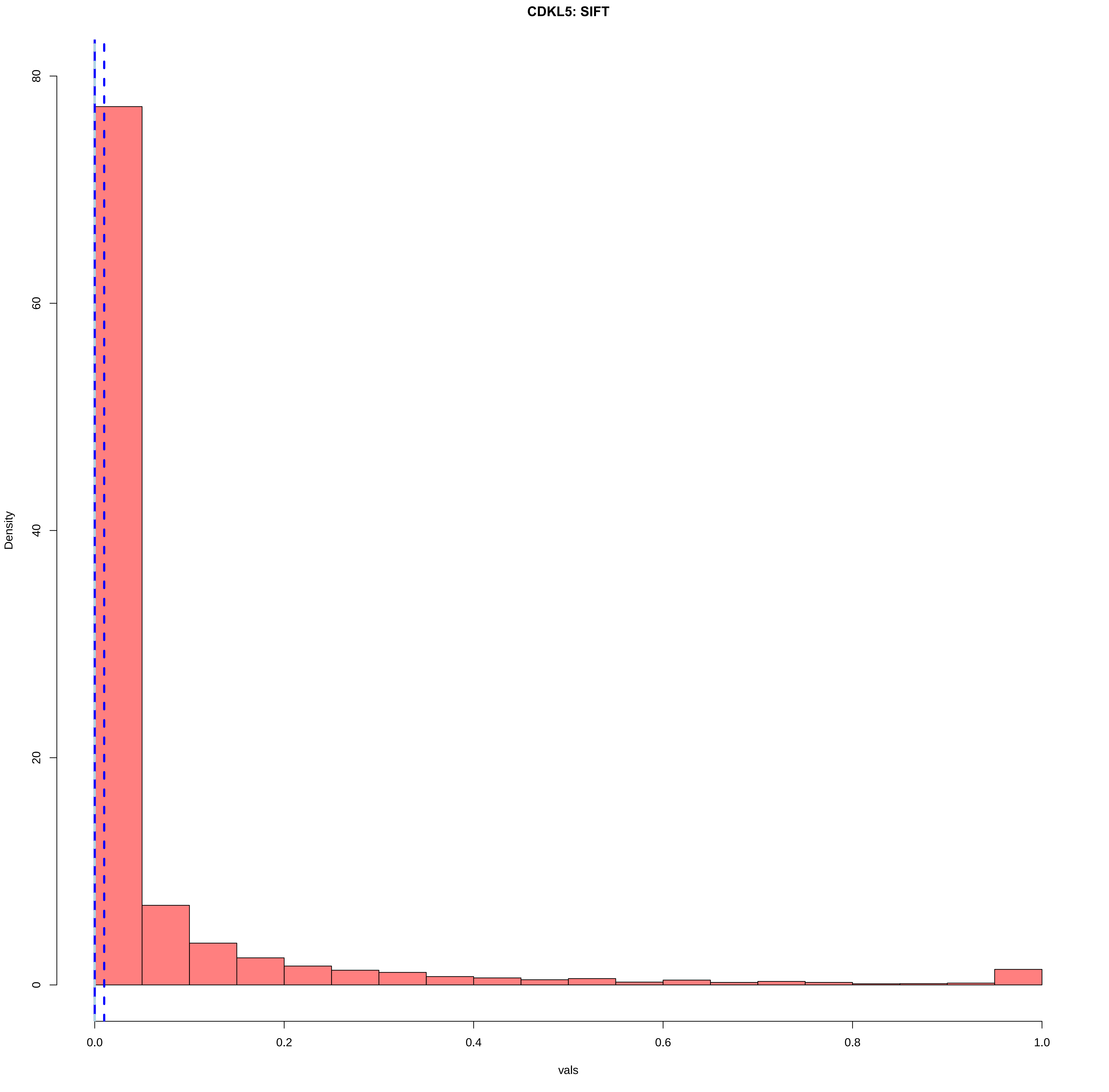
CDKL5: SIFT
Density
vals
0
20
40
60
80
0.0
0.2
0.4
0.6
0.8
1.0

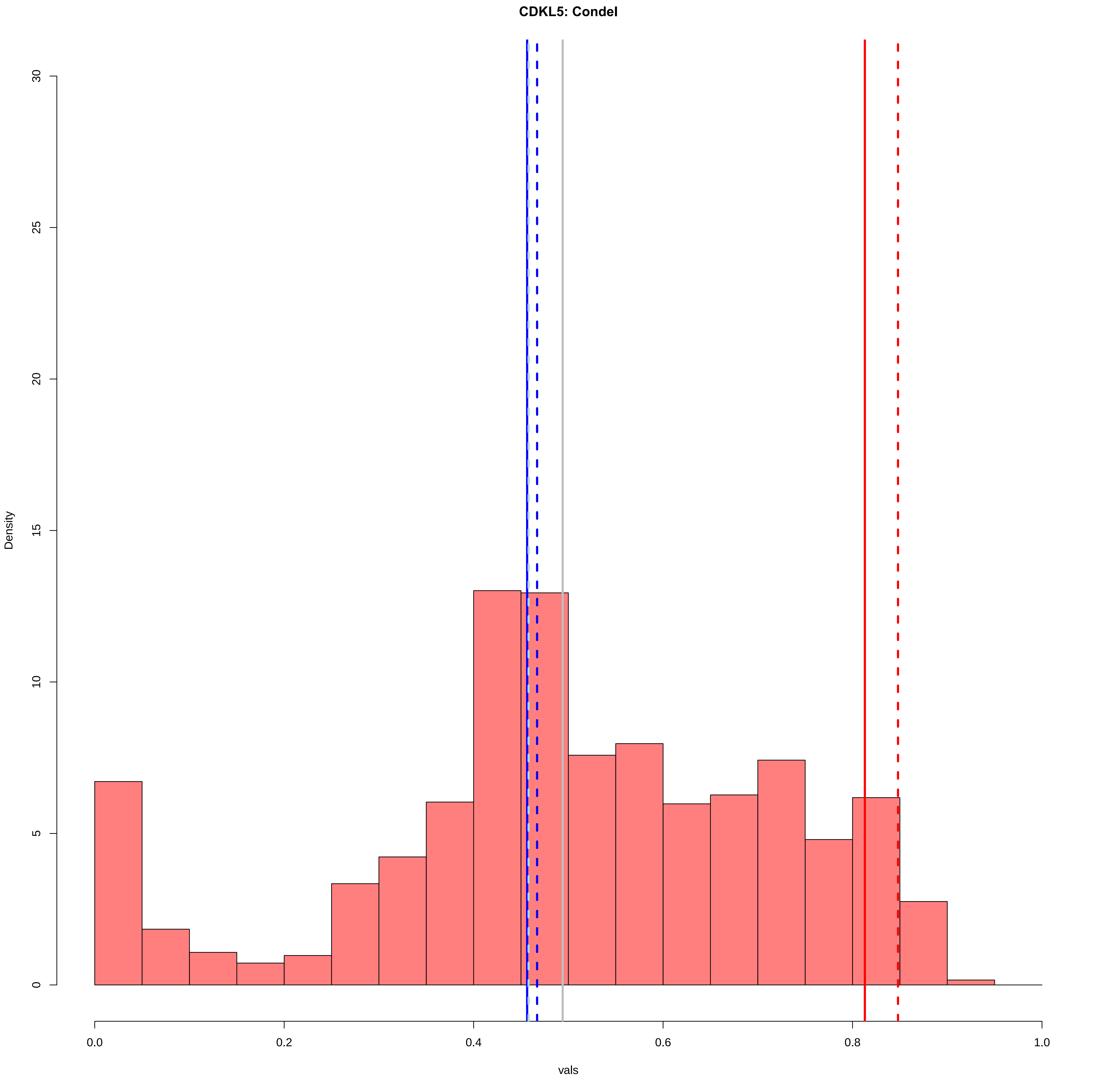
CDKL5: Condel
Density
vals
0
5
10
15
20
25
30
0.0
0.2
0.4
0.6
0.8
1.0

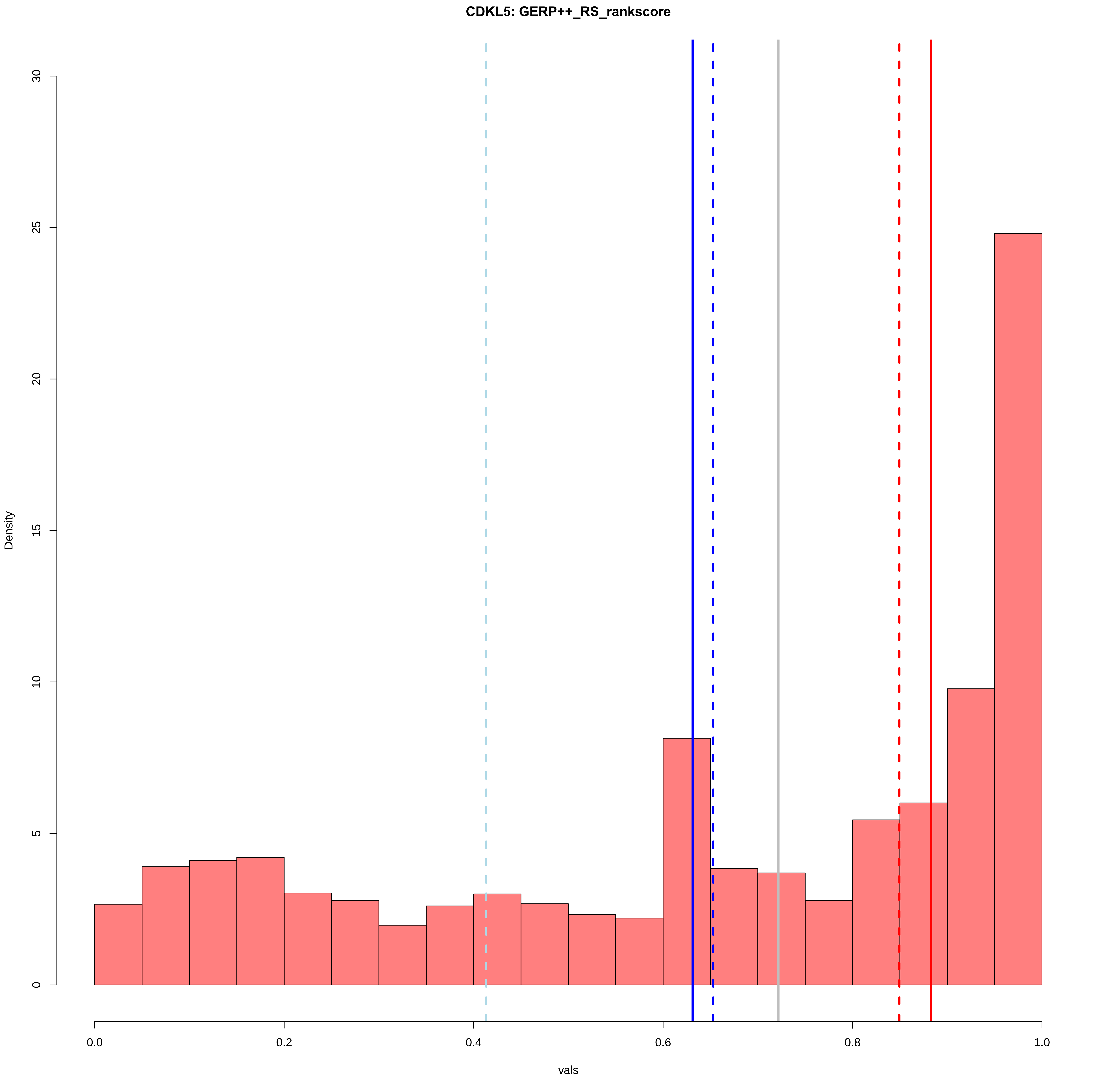

CDKL5: GERP++_RS_rankscore
Density
vals
0
5
10
15
20
25
30
0.0
0.2
0.4
0.6
0.8
1.0

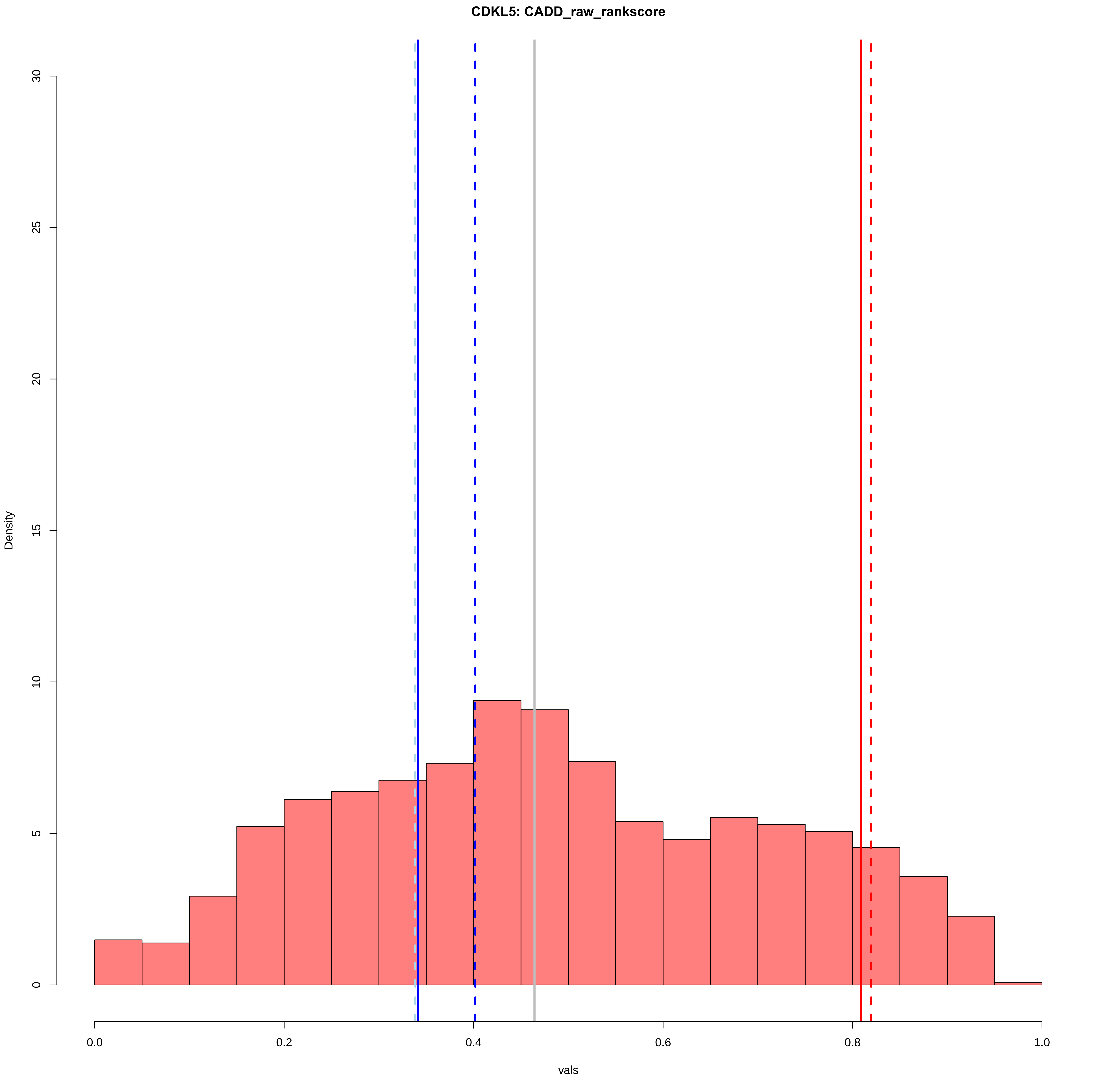
CDKL5: CADD_raw_rankscore
Density
vals
0
5
10
15
20
25
30
0.0
0.2
0.4
0.6
0.8
1.0

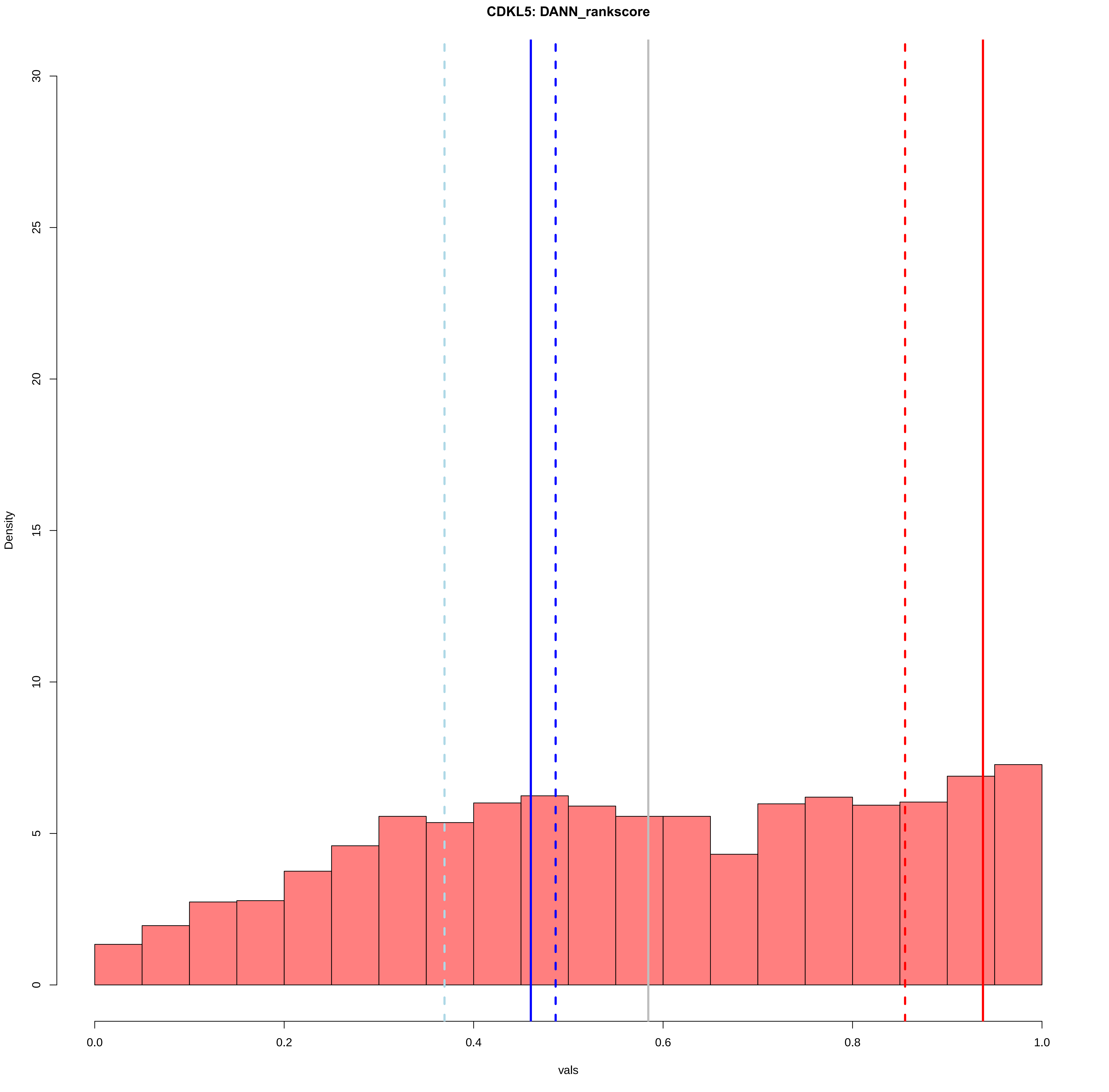

CDKL5: DANN_rankscore
Density
vals
0
5
10
15
20
25
30
0.0
0.2
0.4
0.6
0.8
1.0

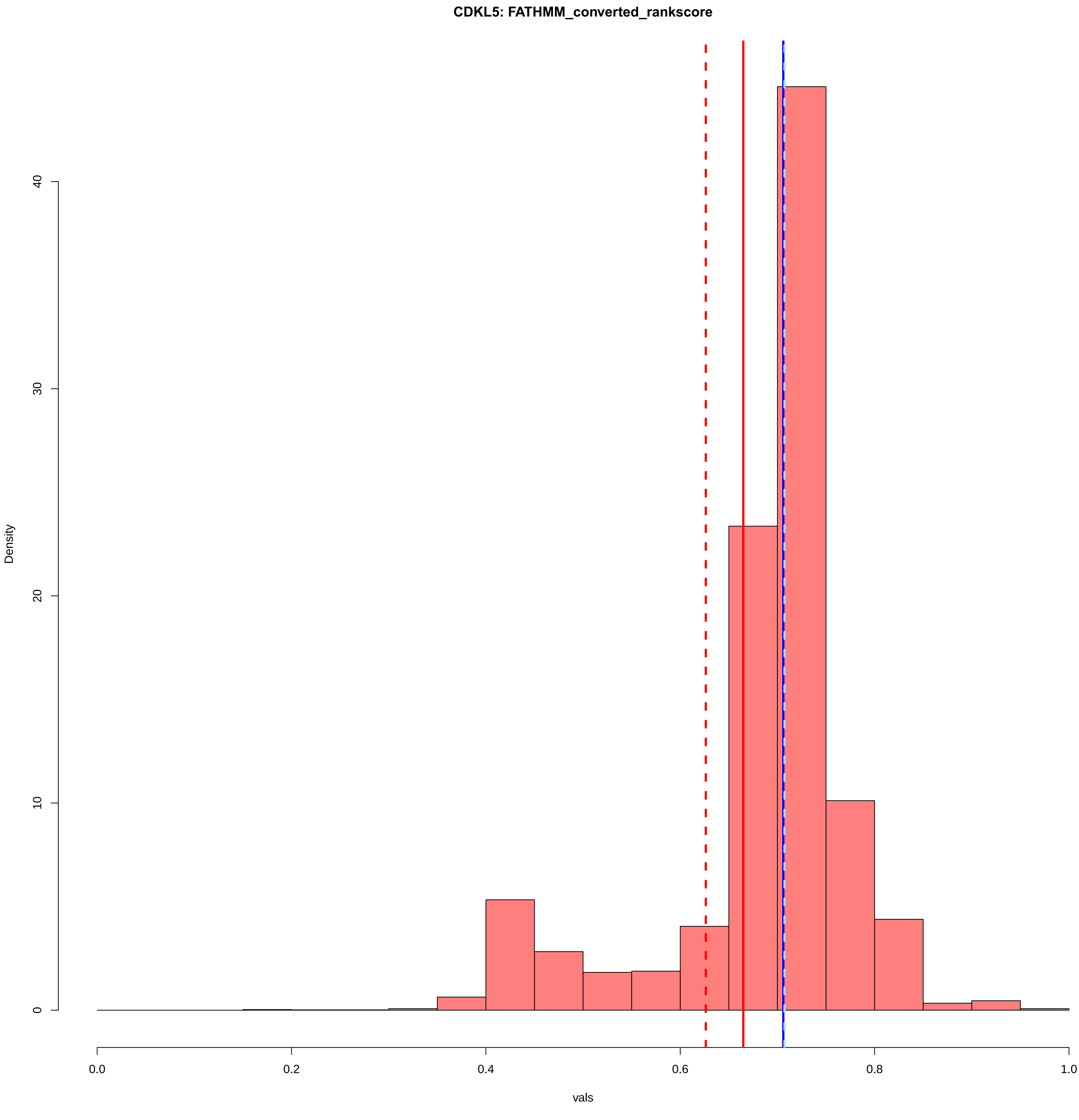

CDKL5: FATHMM_converted_rankscore
Density
vals
0
10
20
30
40
0.0
0.2
0.4
0.6
0.8
1.0

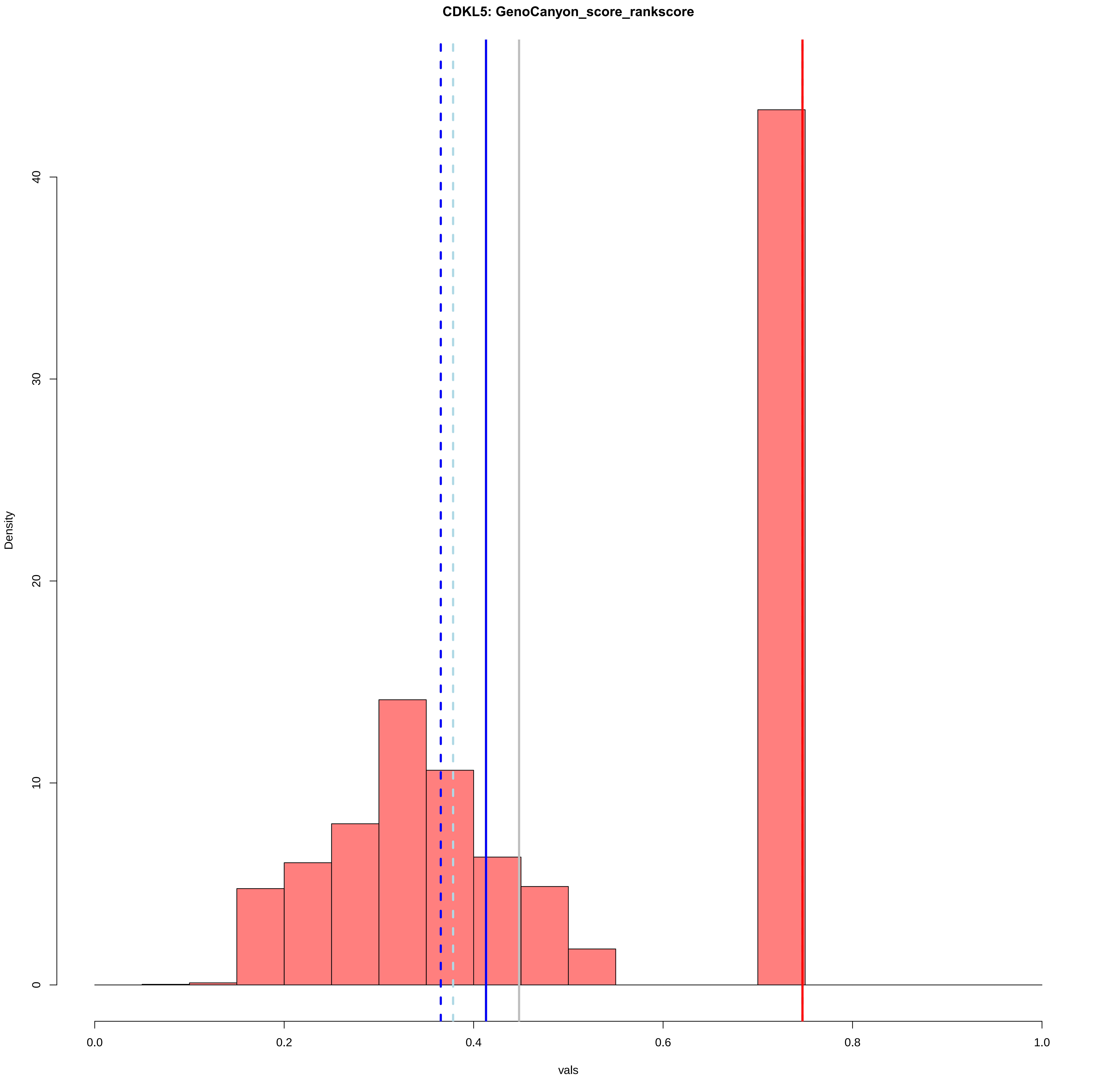
CDKL5: GenoCanyon_score_rankscore
Density
0
10
20
30
40
0.0
0.2
0.4
0.6
0.8
1.0
vals

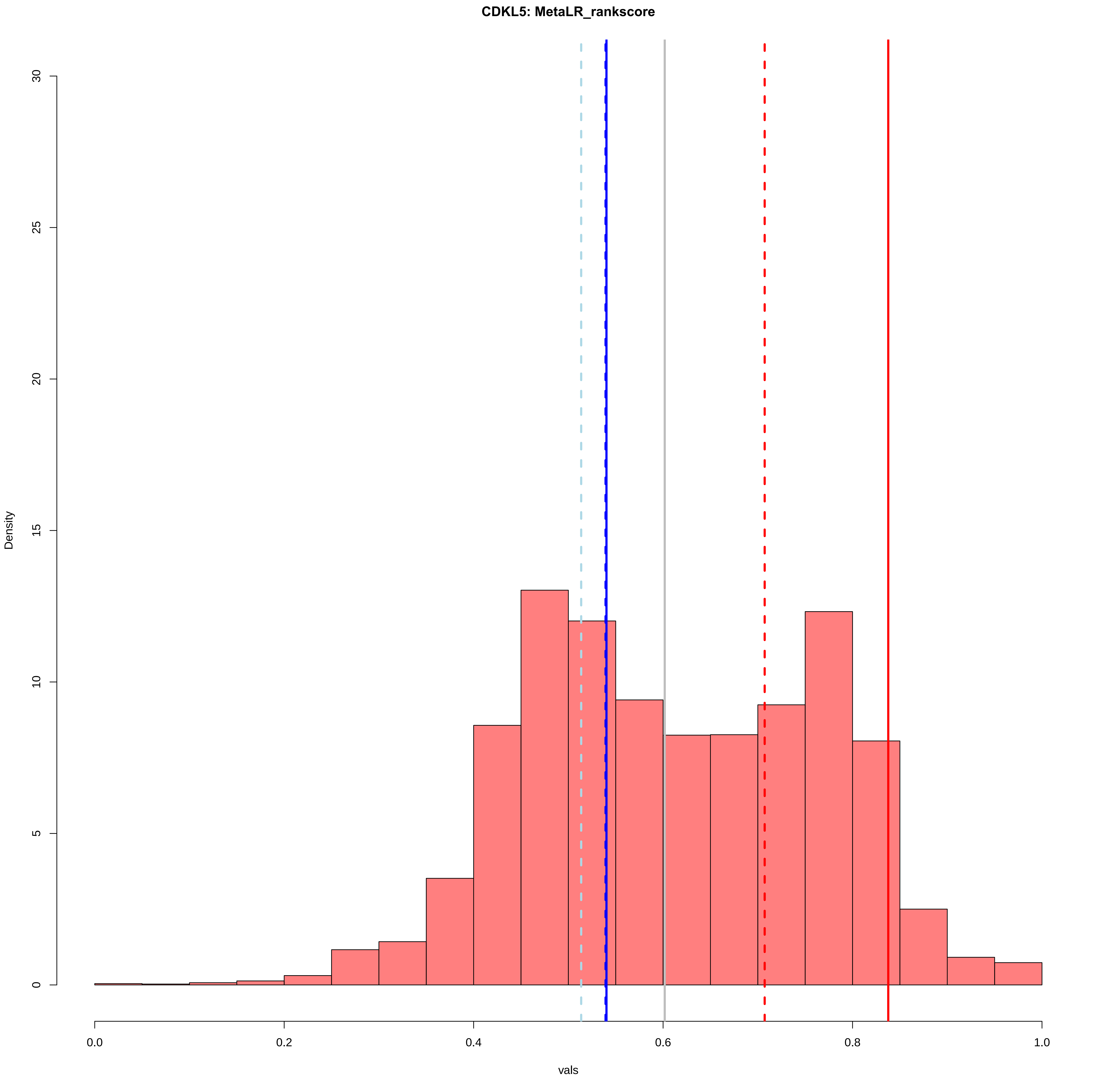
CDKL5: MetaLR_rankscore
Density
vals
0
5
10
15
20
25
30
0.0
0.2
0.4
0.6
0.8
1.0

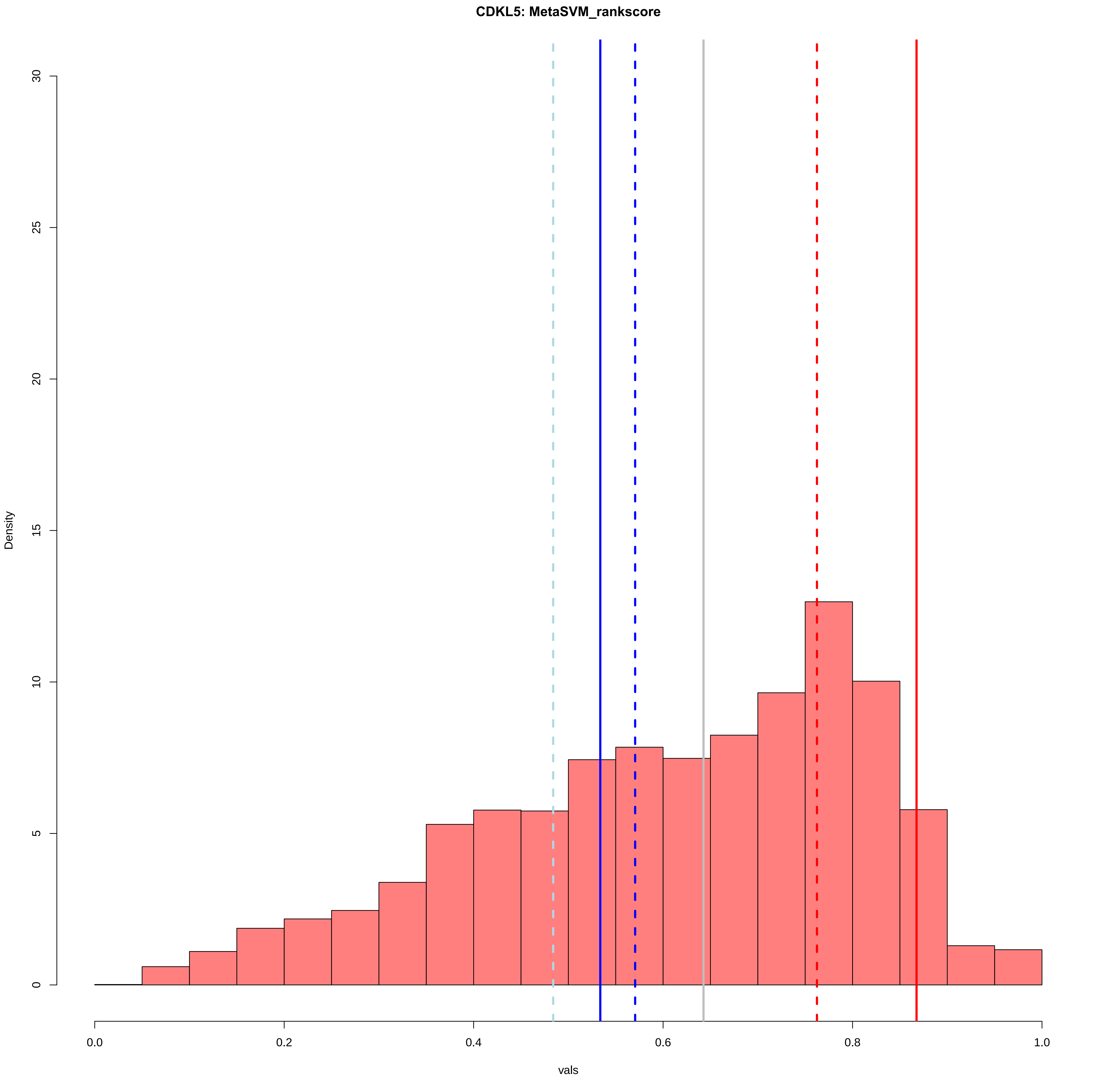

CDKL5: MetaSVM_rankscore
Density
0
5
10
15
20
25
30
0.0
0.2
0.4
0.6
0.8
1.0
vals

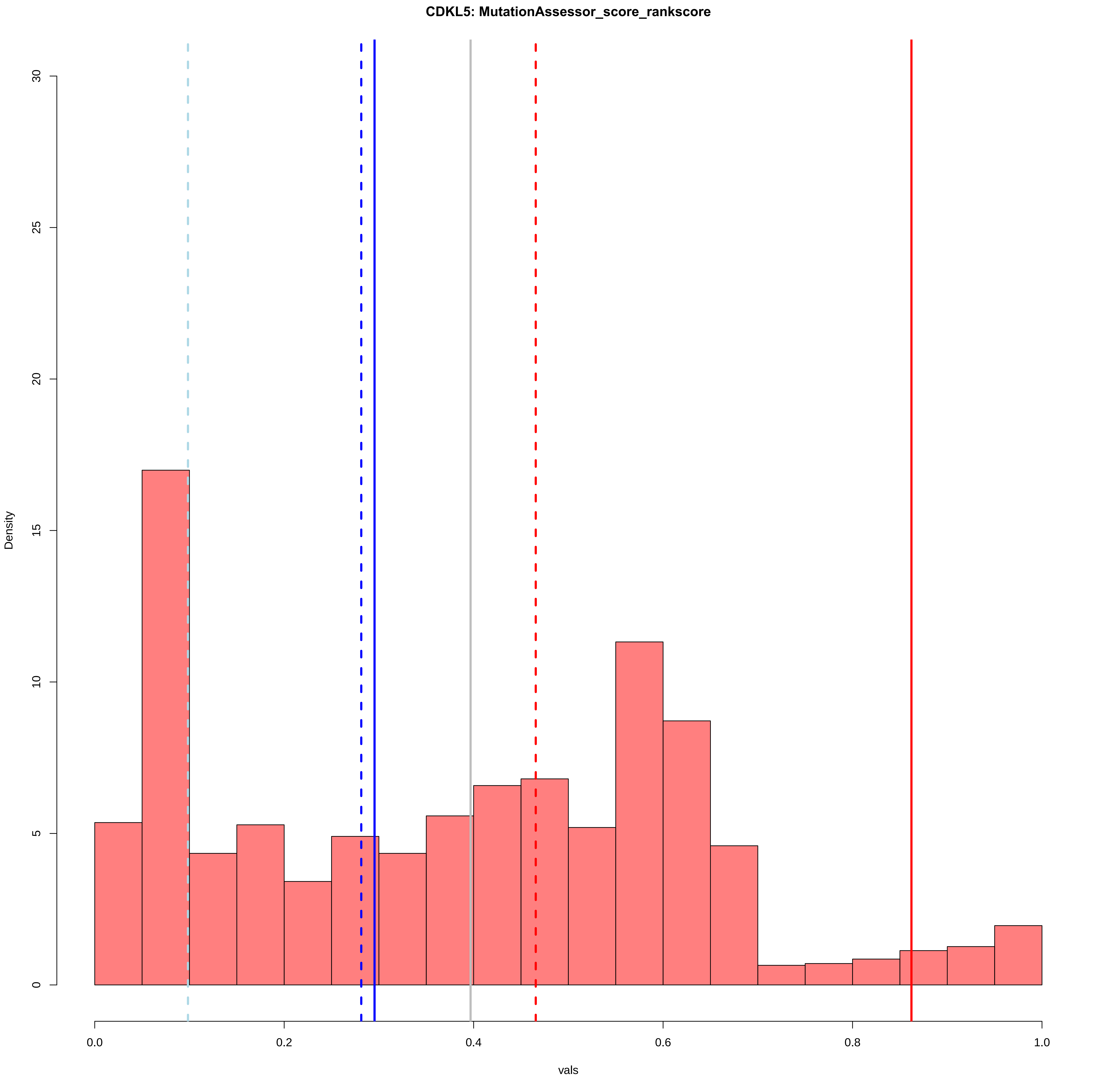

CDKL5: MutationAssessor_score_rankscore
Density
vals
0
5
10
15
20
25
30
0.0
0.2
0.4
0.6
0.8
1.0

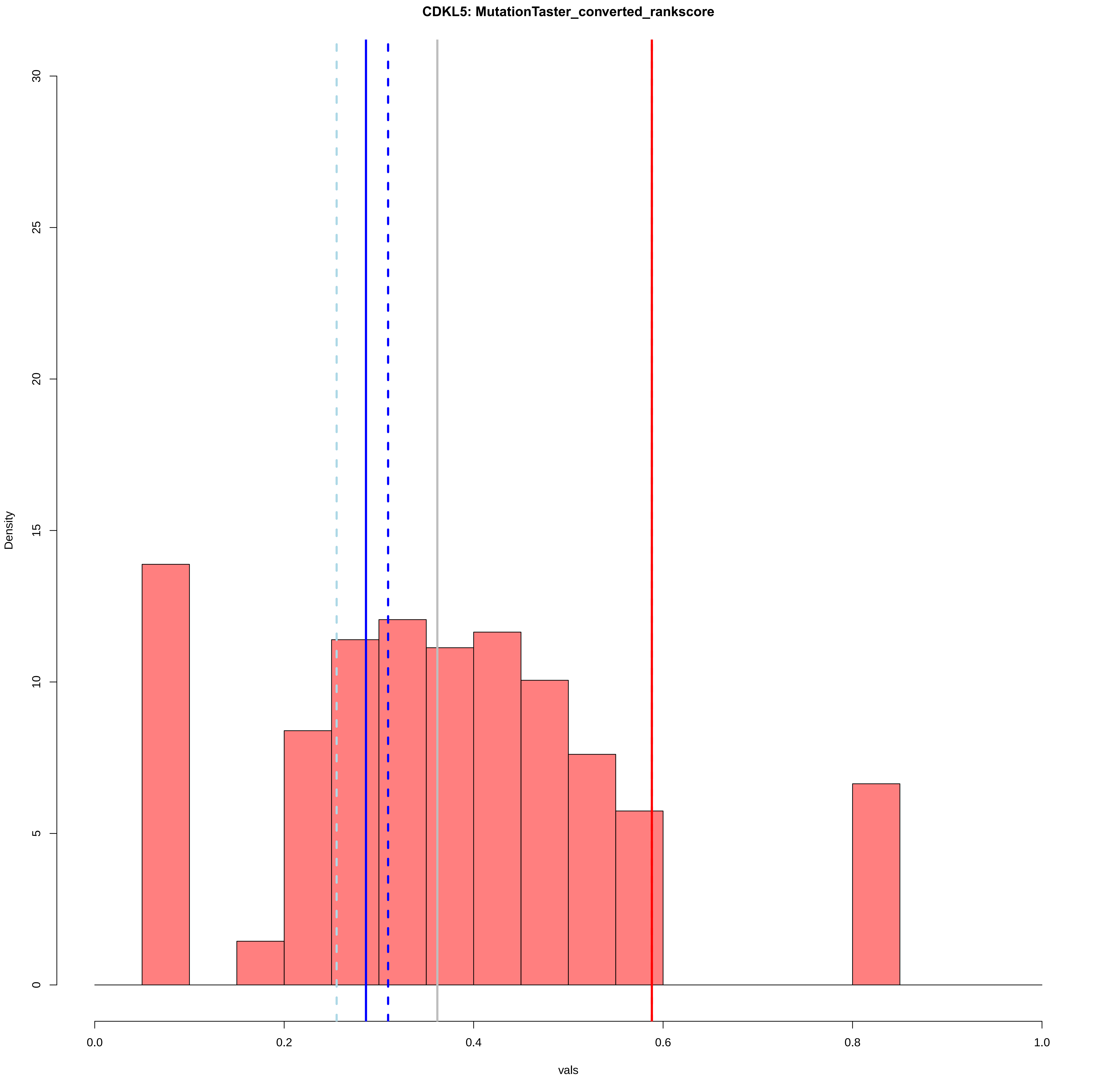
CDKL5: MutationTaster_converted_rankscore
Density
vals
0
5
10
15
20
25
30
0.0
0.2
0.4
0.6
0.8
1.0

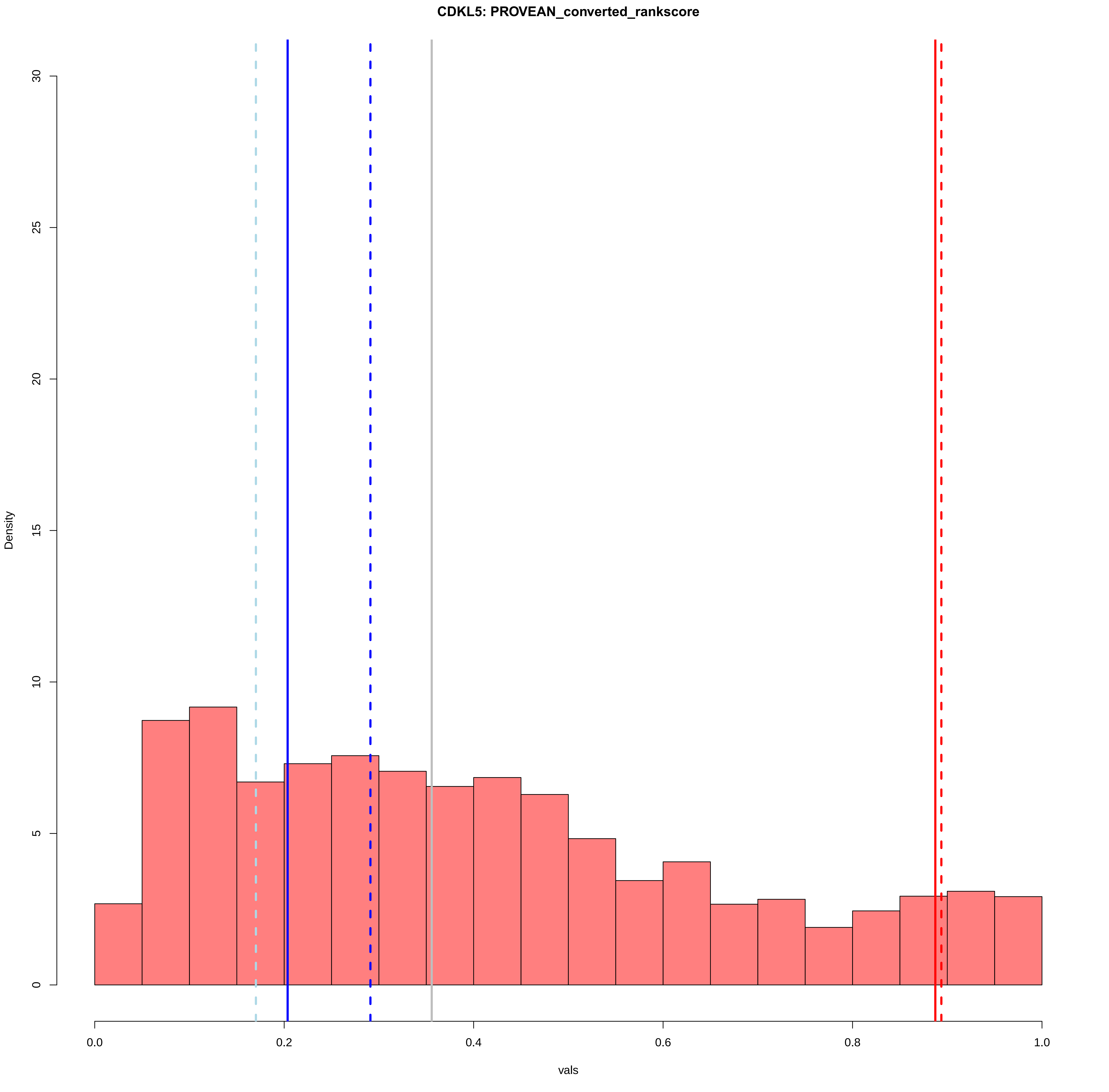
CDKL5: PROVEAN_converted_rankscore
Density
0
5
10
15
20
25
30
0.0
0.2
0.4
0.6
0.8
1.0
vals

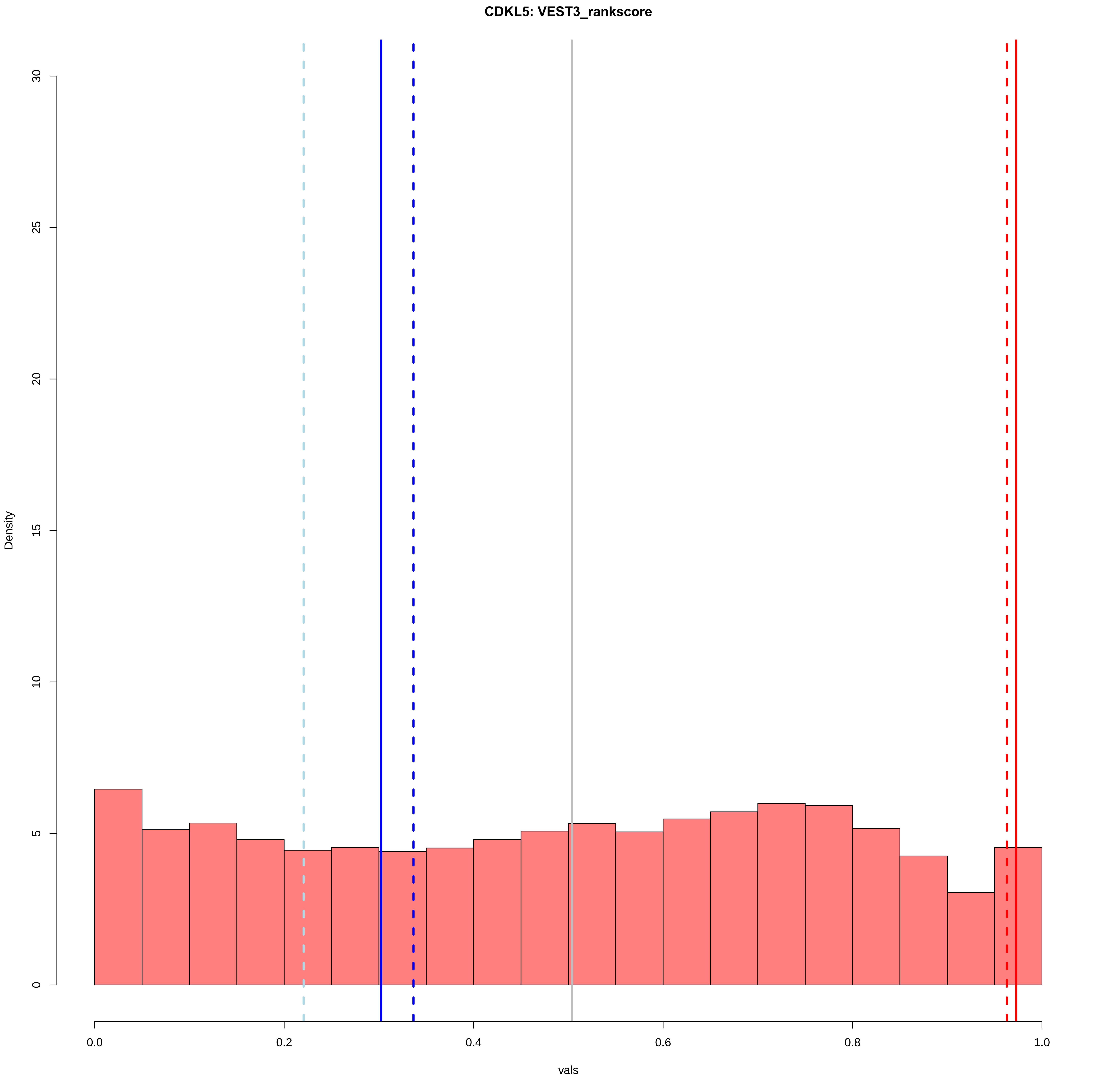
CDKL5: VEST3_rankscore
Density
vals
0
5
10
15
20
25
30
0.0
0.2
0.4
0.6
0.8
1.0

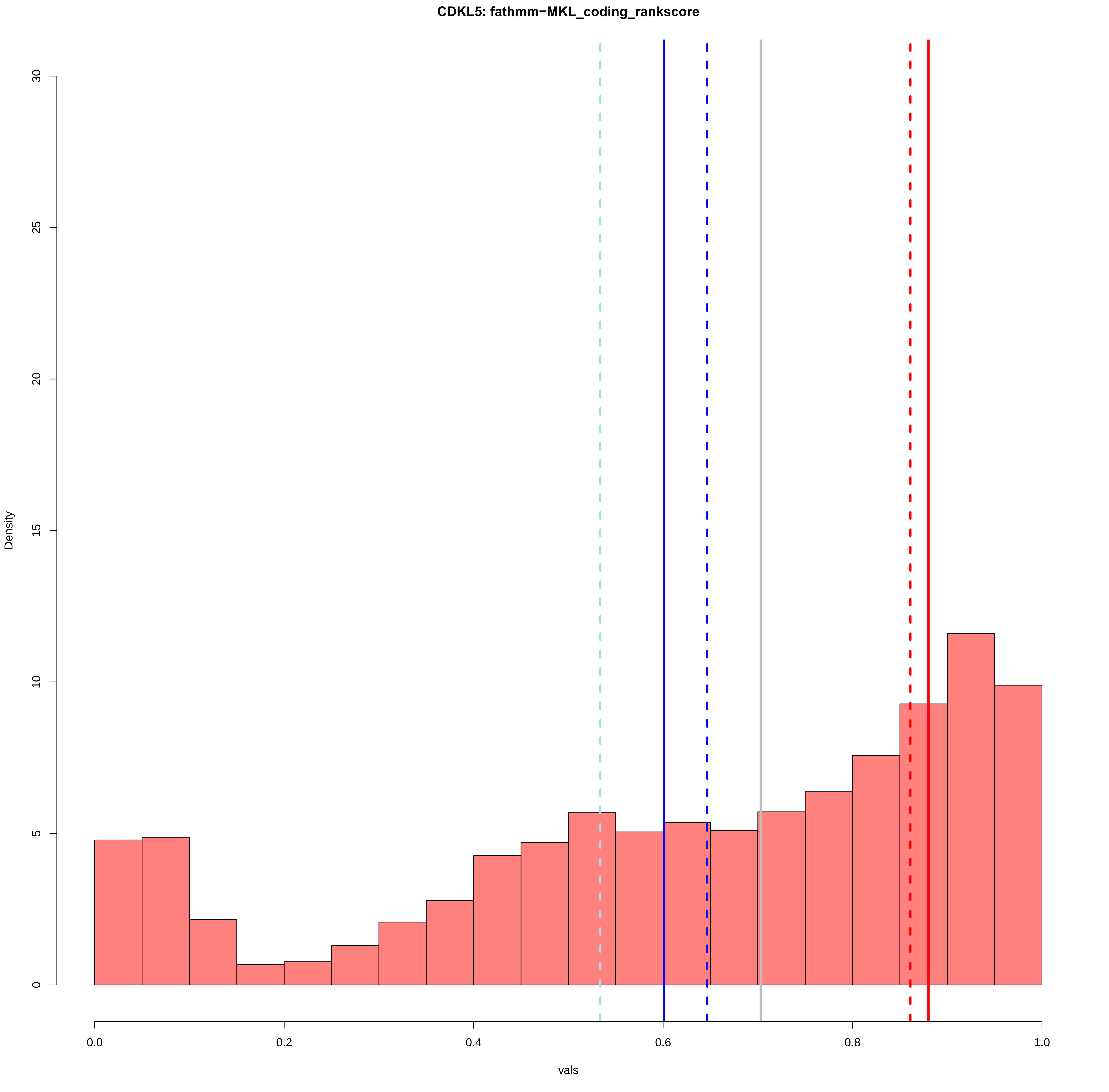

CDKL5: fathmm−MKL_coding_rankscore
Density
vals
0
5
10
15
20
25
30
0.0
0.2
0.4
0.6
0.8
1.0

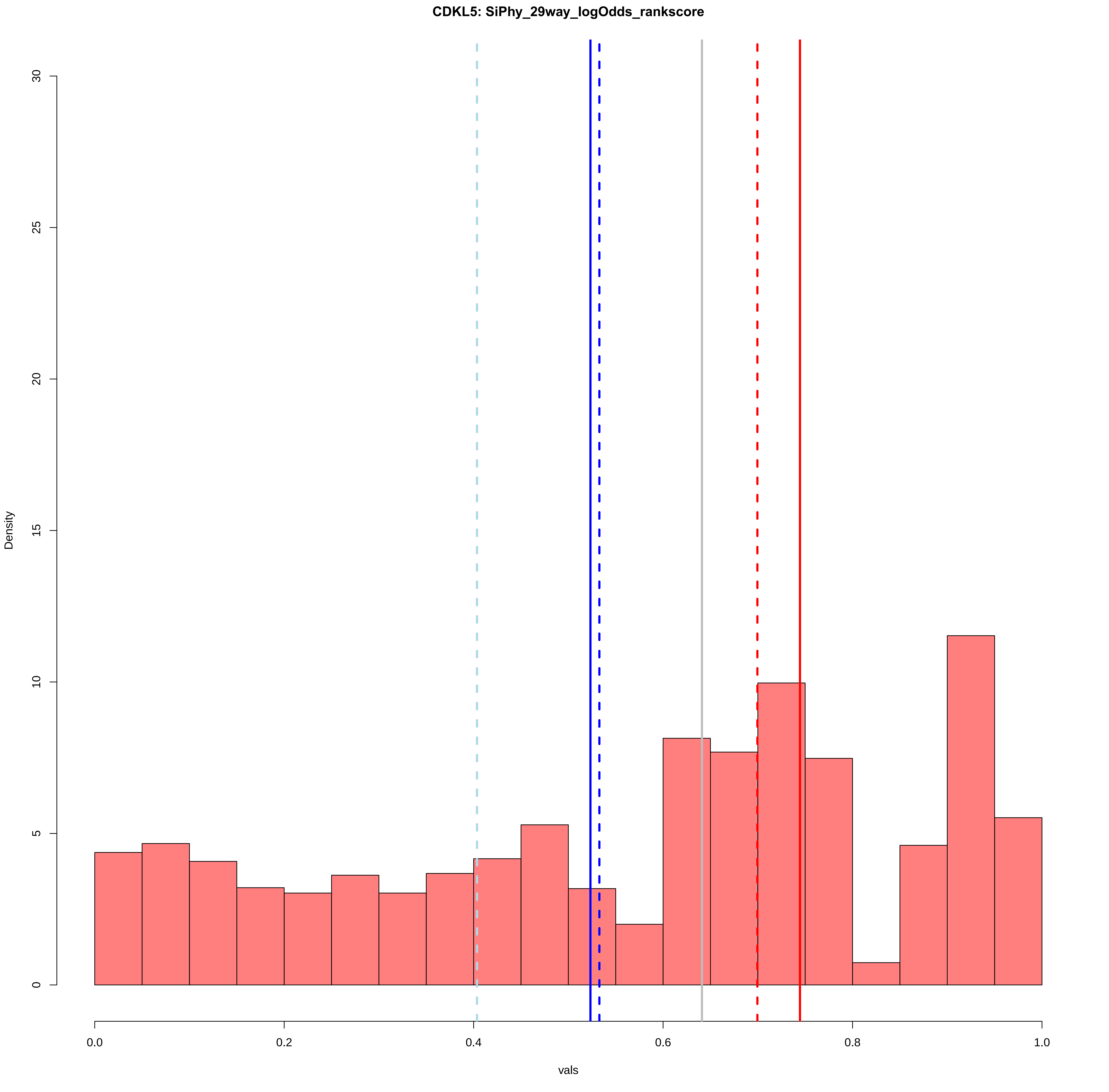
CDKL5: SiPhy_29way_logOdds_rankscore
Density
vals
0
5
10
15
20
25
30
0.0
0.2
0.4
0.6
0.8
1.0

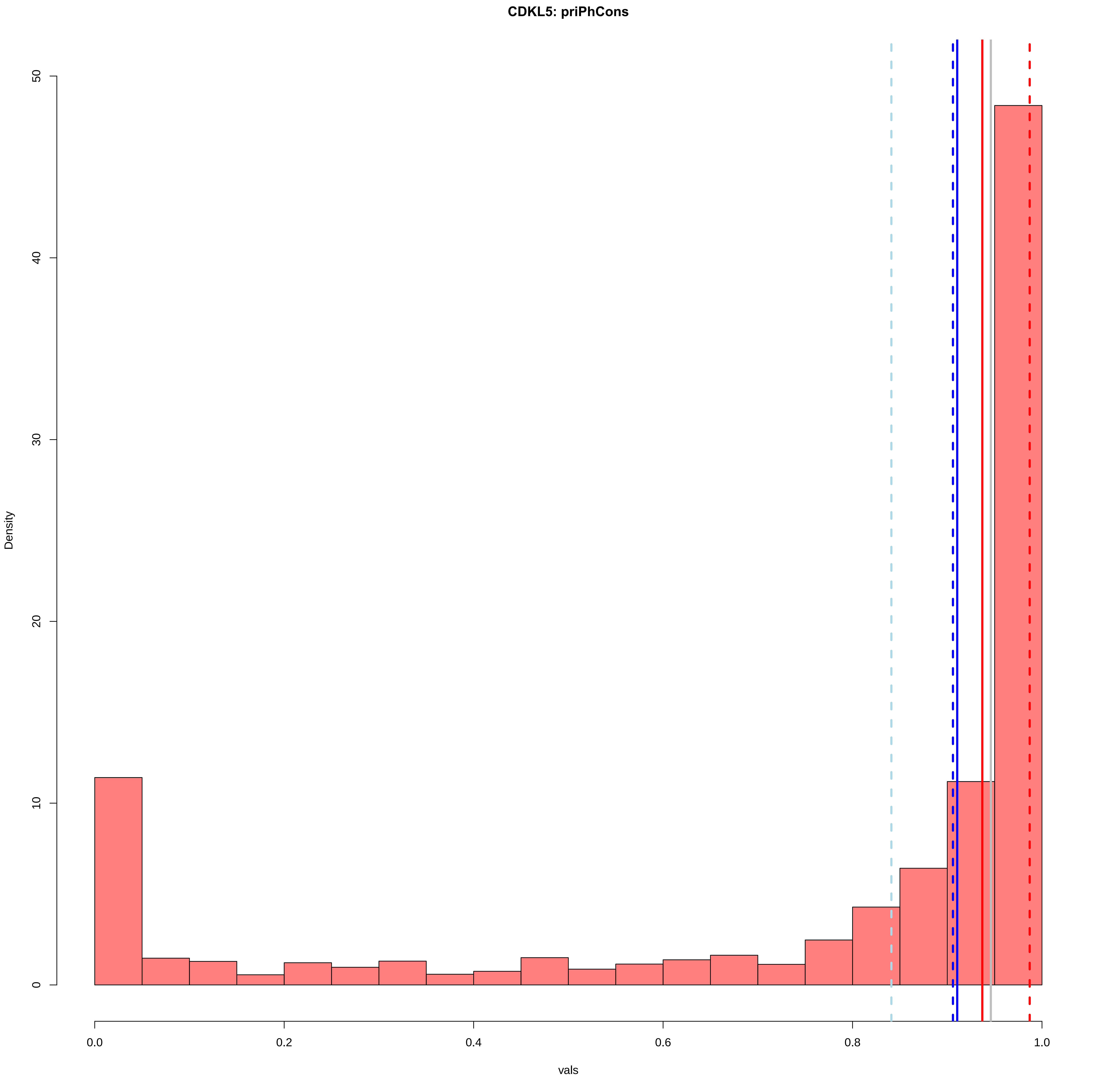
CDKL5: priPhCons
Density
vals
0
10
20
30
40
50
0.0
0.2
0.4
0.6
0.8
1.0

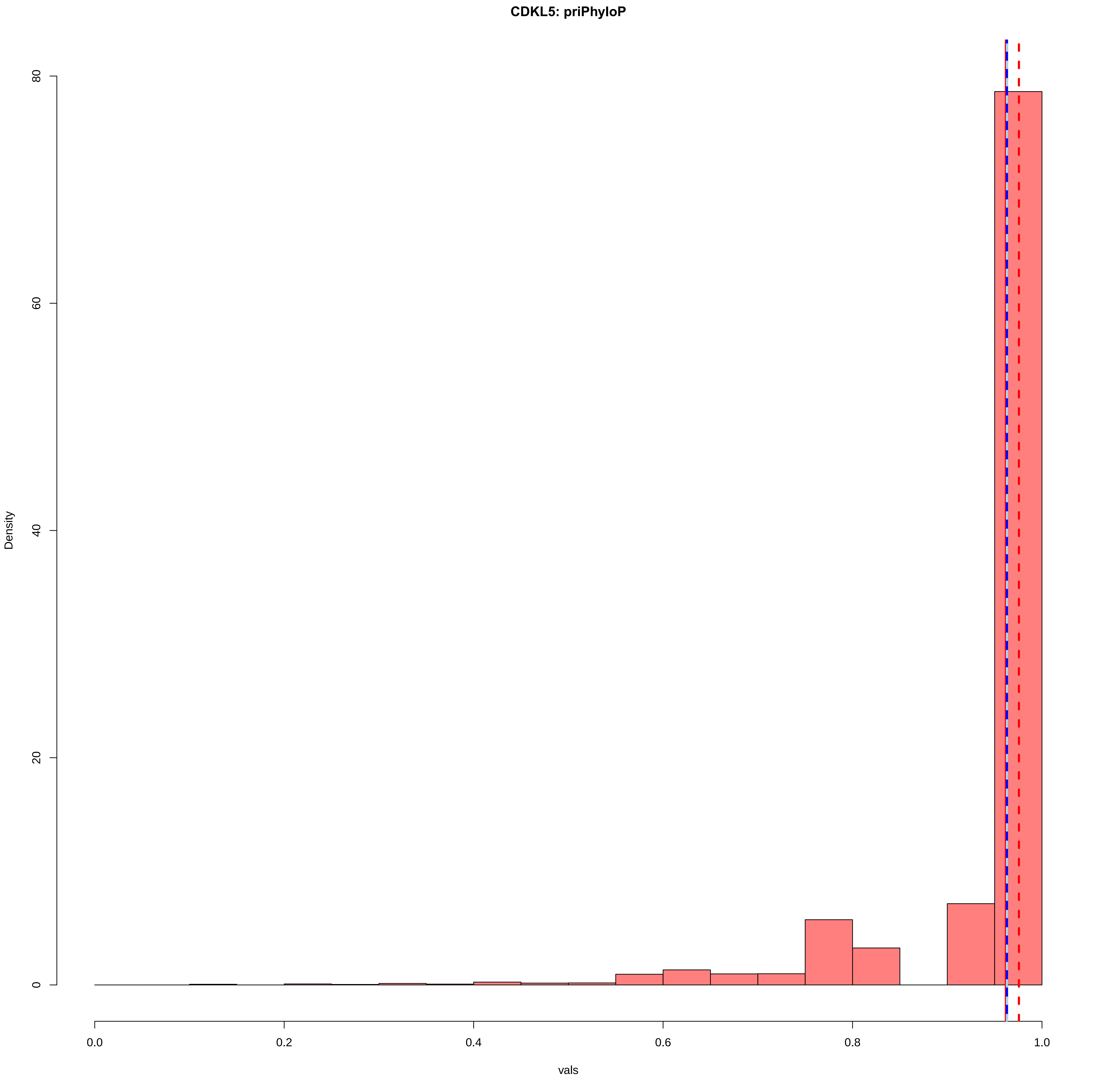

CDKL5: priPhyloP
Density
vals
0
20
40
60
80
0.0
0.2
0.4
0.6
0.8
1.0

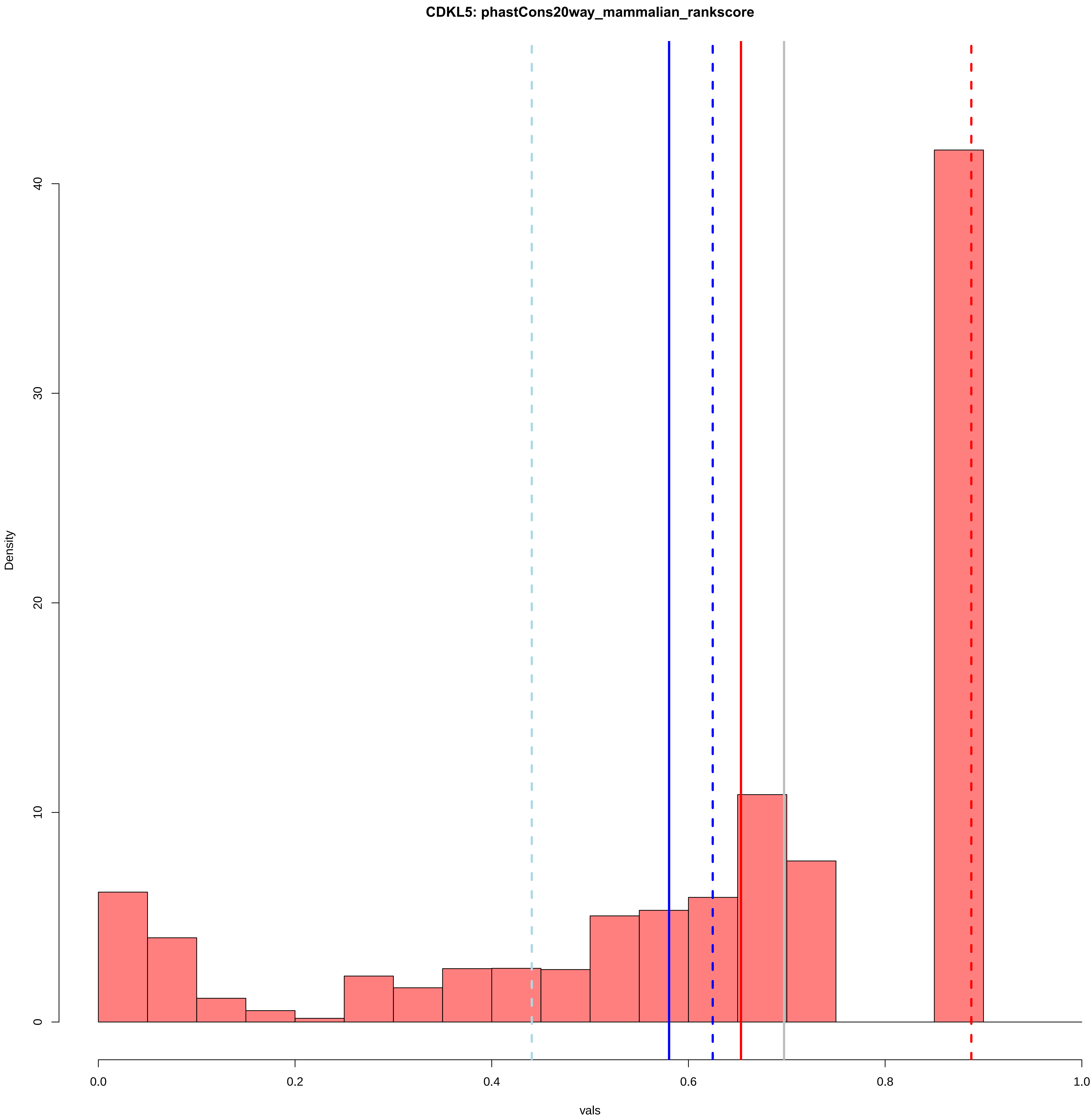

CDKL5: phastCons20way_mammalian_rankscore
Density
vals
0
10
20
30
40
0.0
0.2
0.4
0.6
0.8
1.0

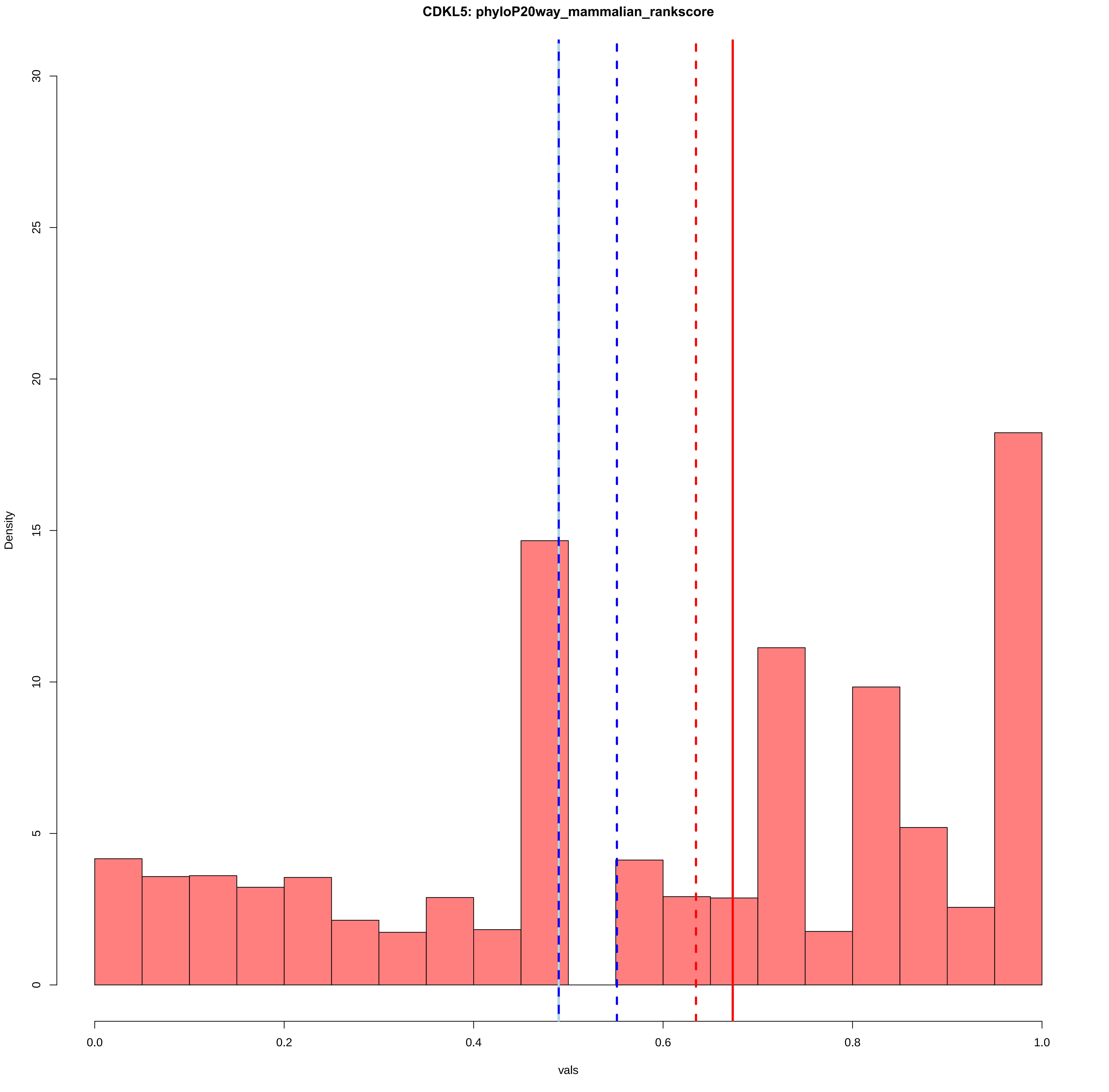
CDKL5: phyloP20way_mammalian_rankscore
Density
vals
0
5
10
15
20
25
30
0.0
0.2
0.4
0.6
0.8
1.0

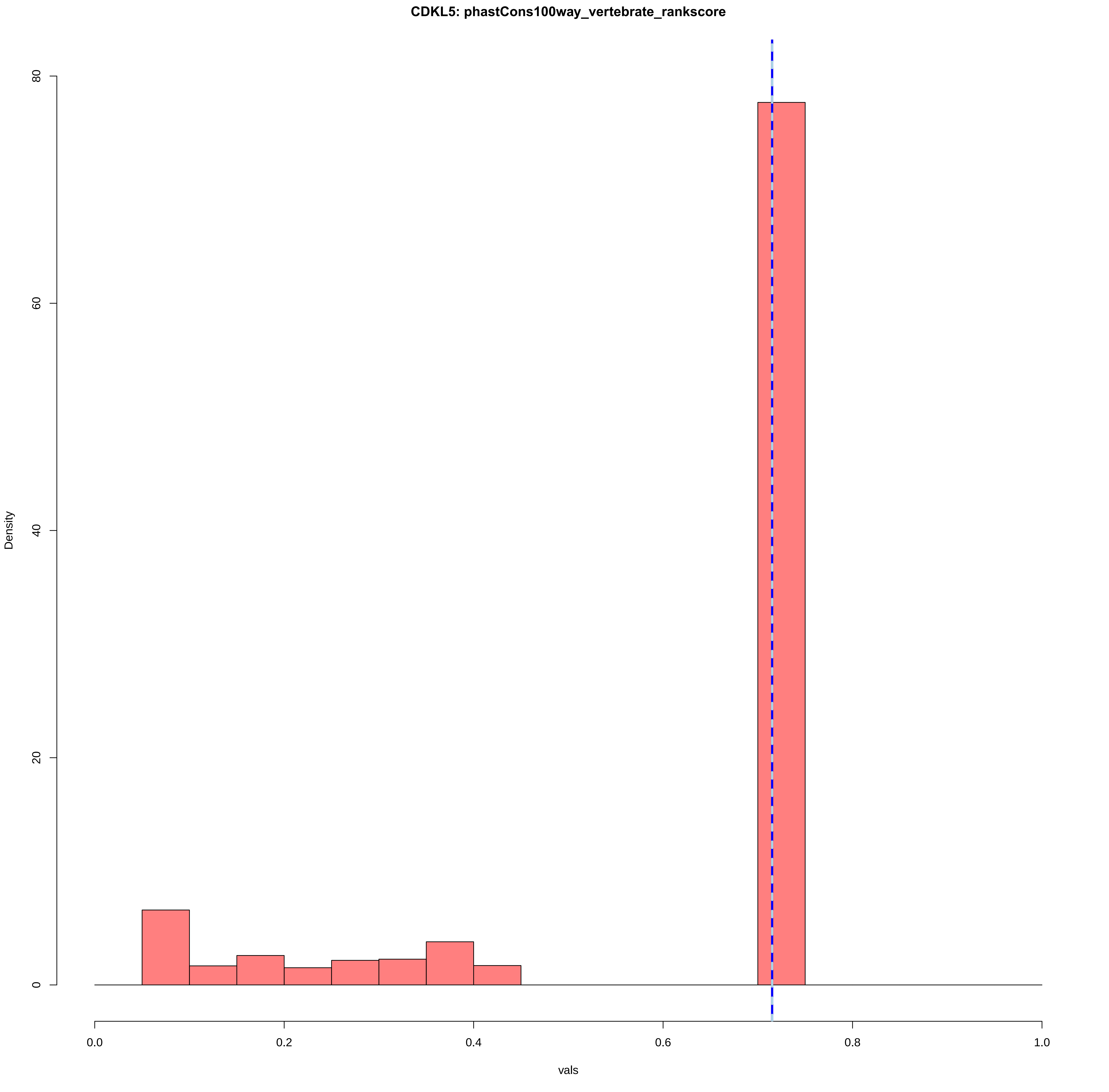
CDKL5: phastCons100way_vertebrate_rankscore
Density
0
20
40
60
80
0.0
0.2
0.4
0.6
0.8
1.0
vals

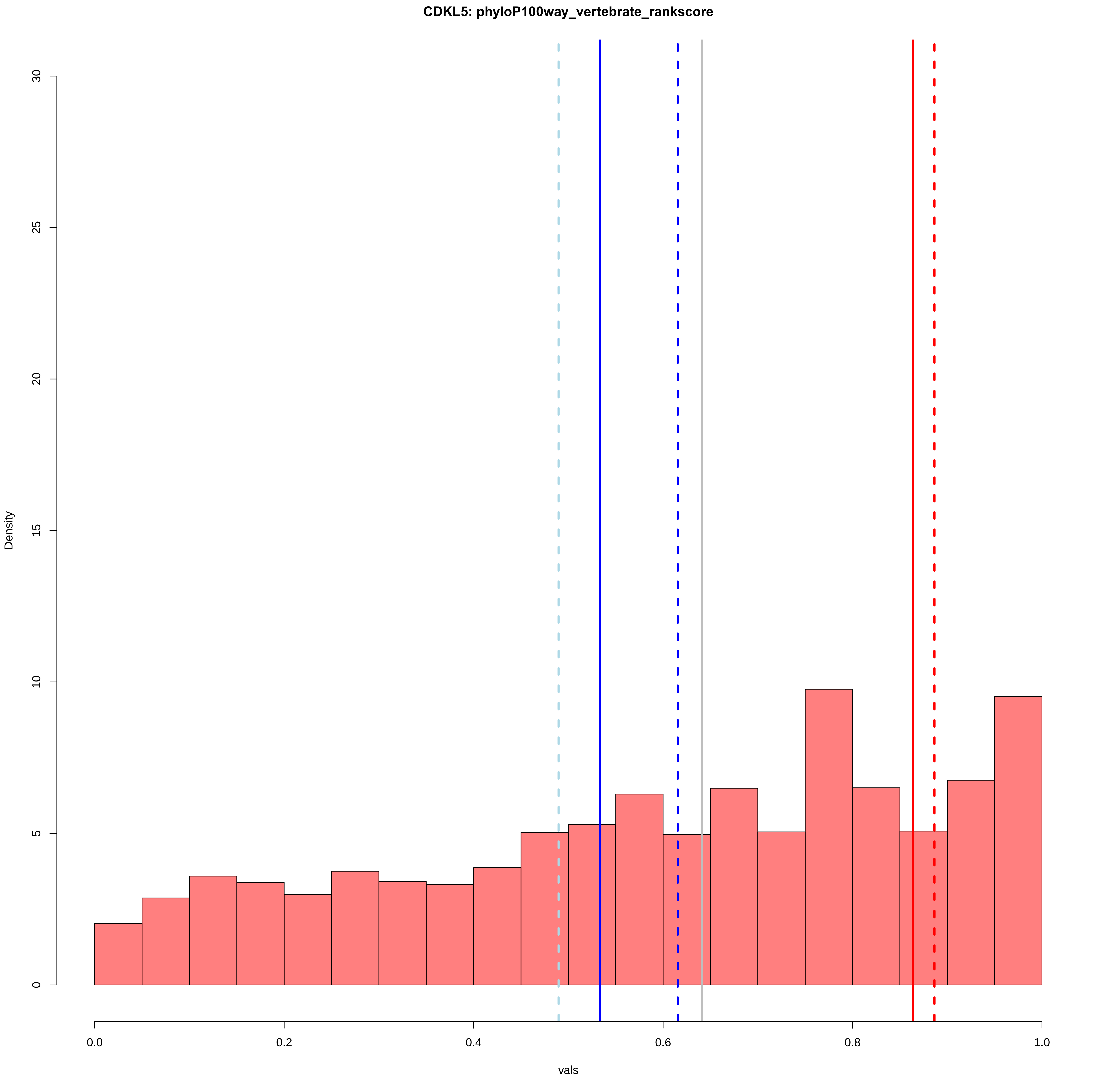

CDKL5: phyloP100way_vertebrate_rankscore
Density
vals
0
5
10
15
20
25
30
0.0
0.2
0.4
0.6
0.8
1.0

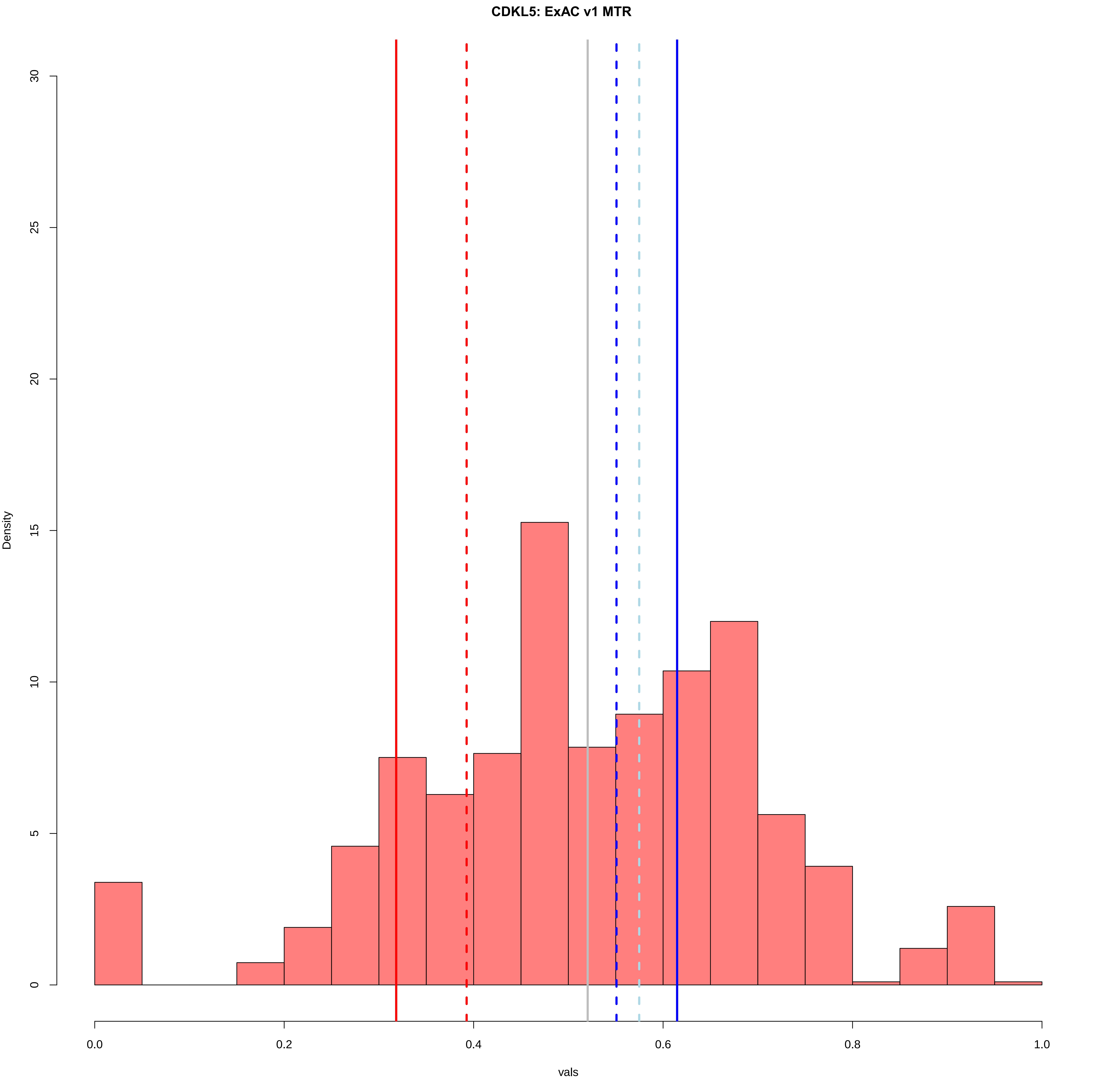

CDKL5: ExAC v1 MTR
Density
vals
0
5
10
15
20
25
30
0.0
0.2
0.4
0.6
0.8
1.0

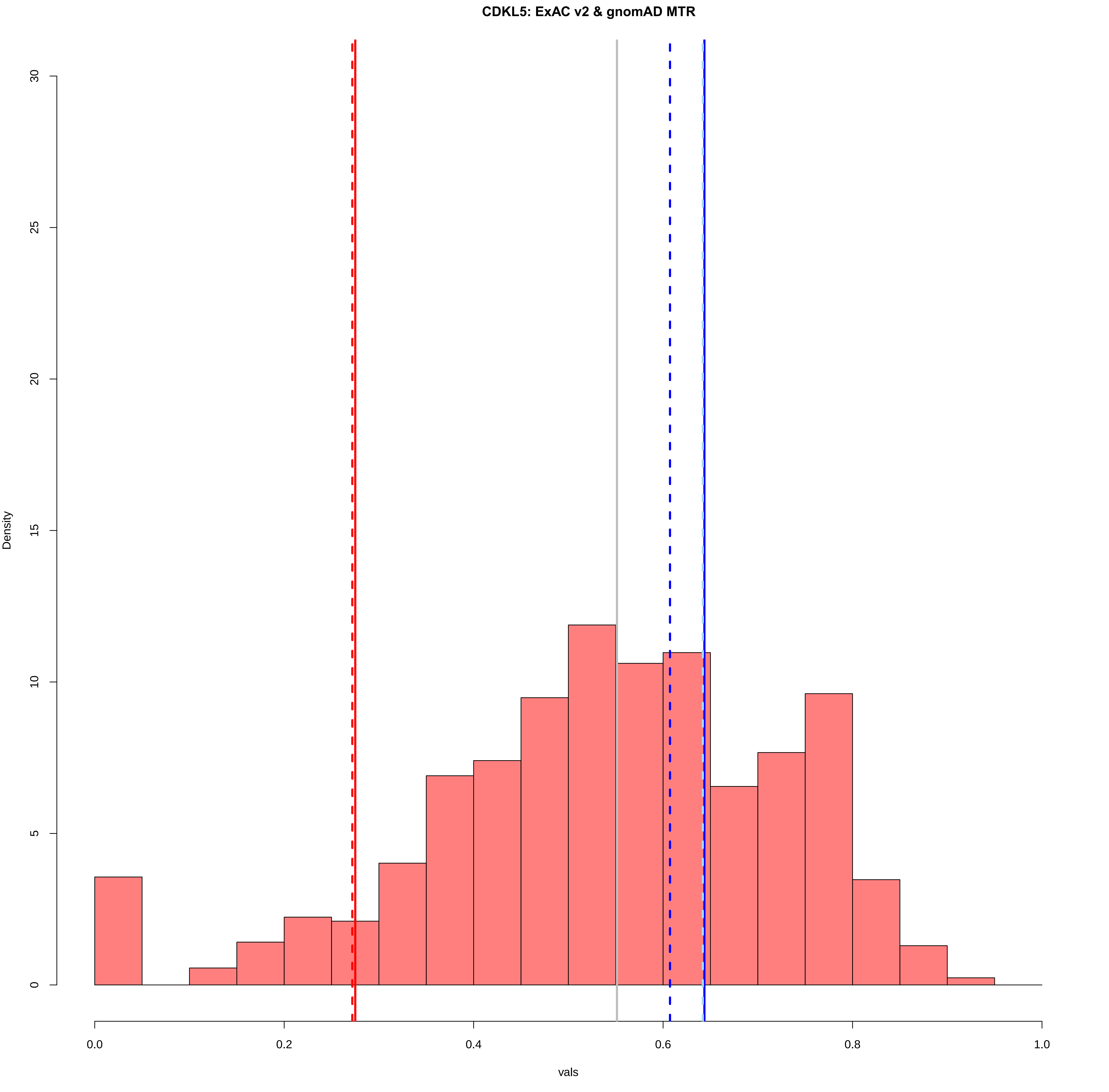

CDKL5: ExAC v2 & gnomAD MTR
Density
vals
0
5
10
15
20
25
30
0.0
0.2
0.4
0.6
0.8
1.0

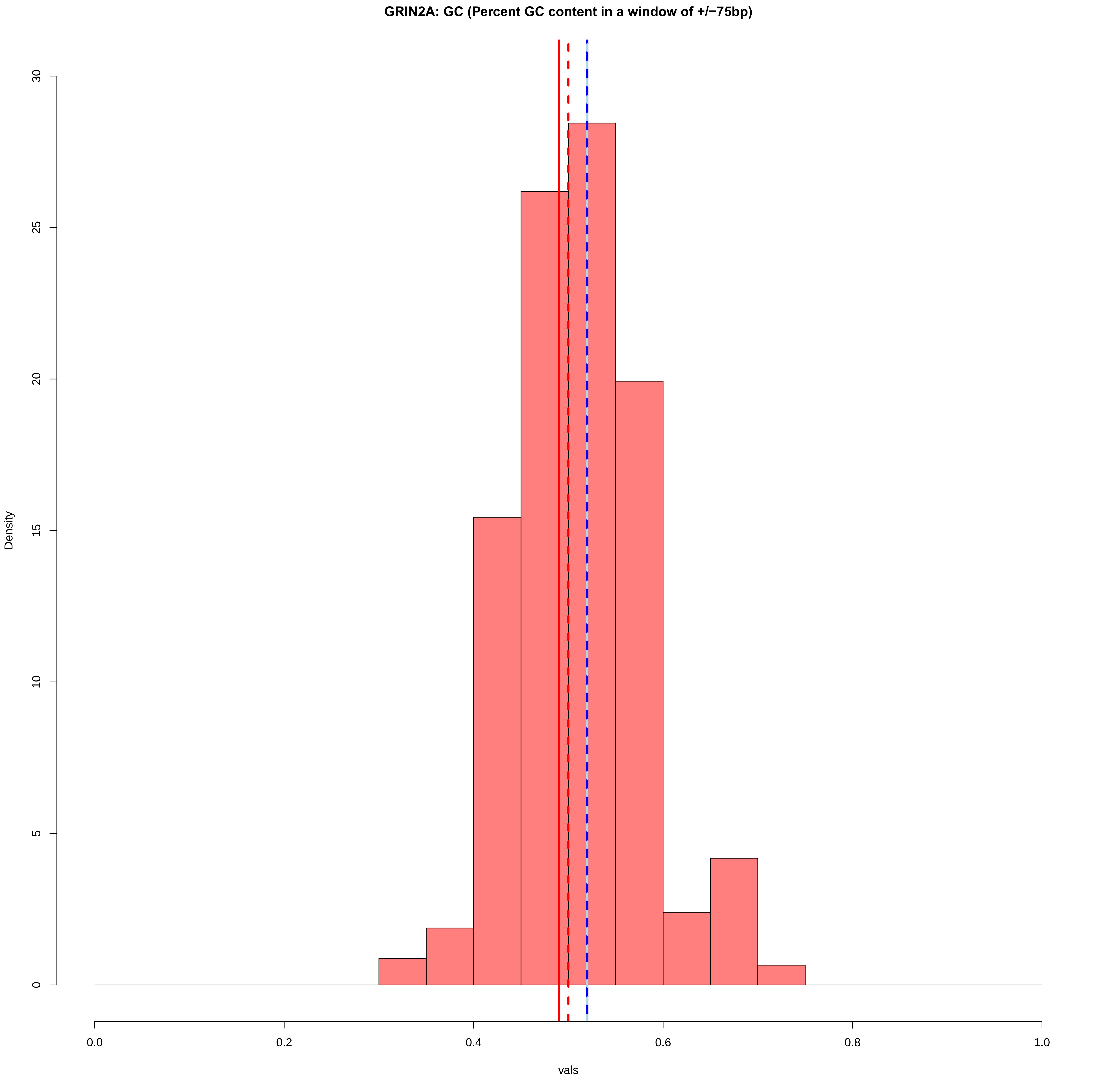

GRIN2A: GC (Percent GC content in a window of +/−75bp)
Density
vals
0
5
10
15
20
25
30
0.0
0.2
0.4
0.6
0.8
1.0

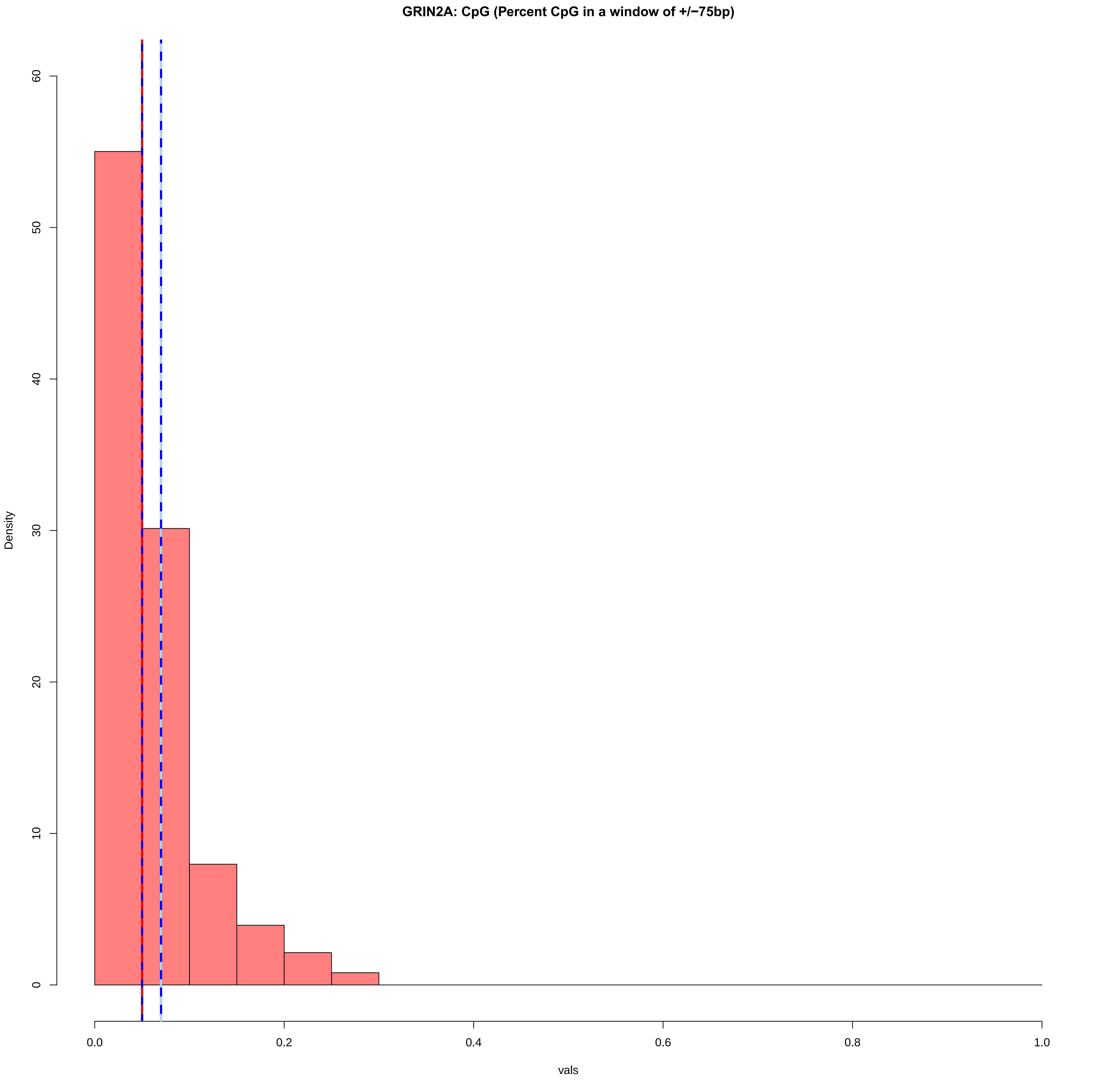
GRIN2A: CpG (Percent CpG in a window of +/−75bp)
Density
vals
0
10
20
30
40
50
60
0.0
0.2
0.4
0.6
0.8
1.0

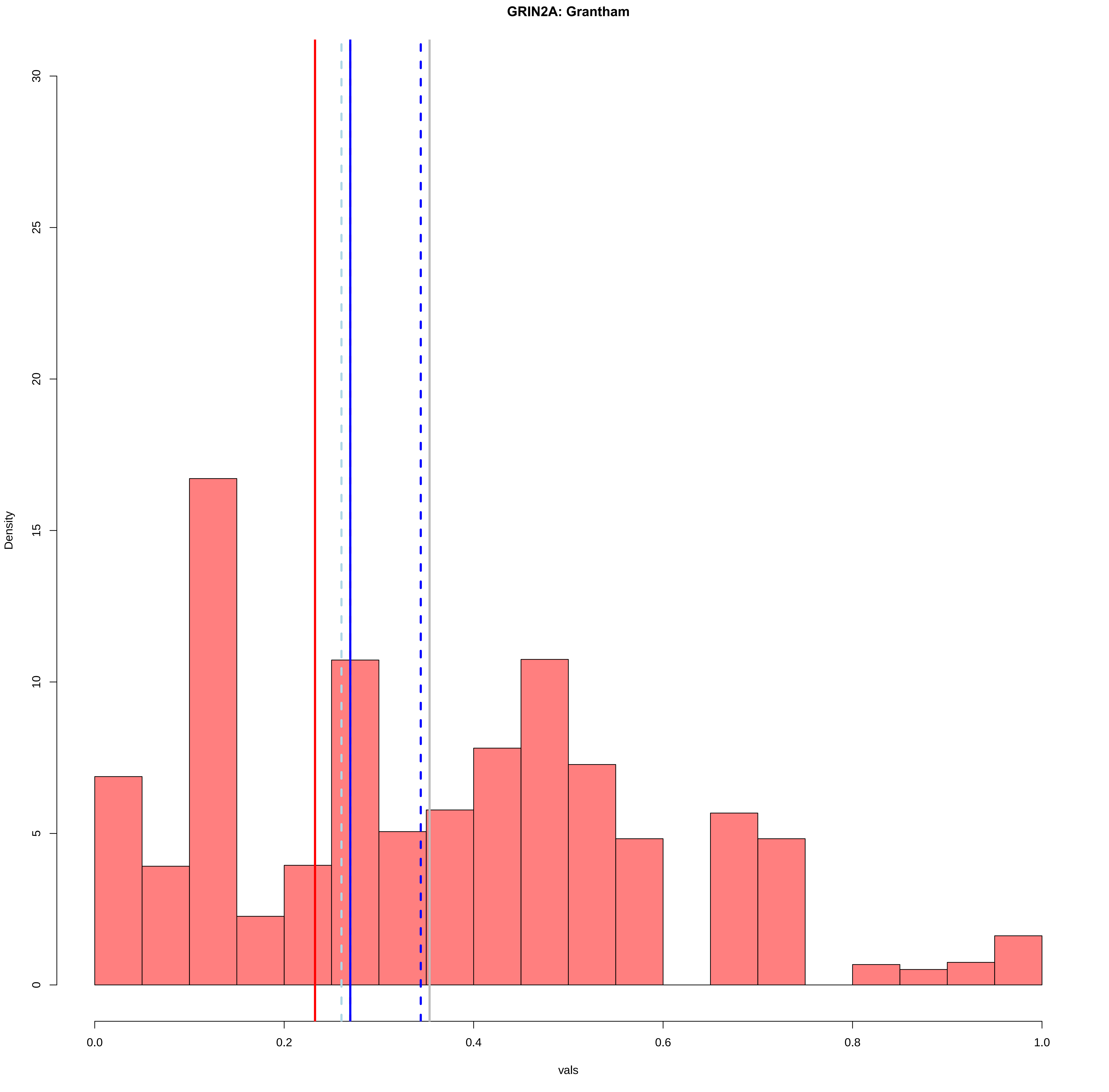
GRIN2A: Grantham
Density
vals
0
5
10
15
20
25
30
0.0
0.2
0.4
0.6
0.8
1.0

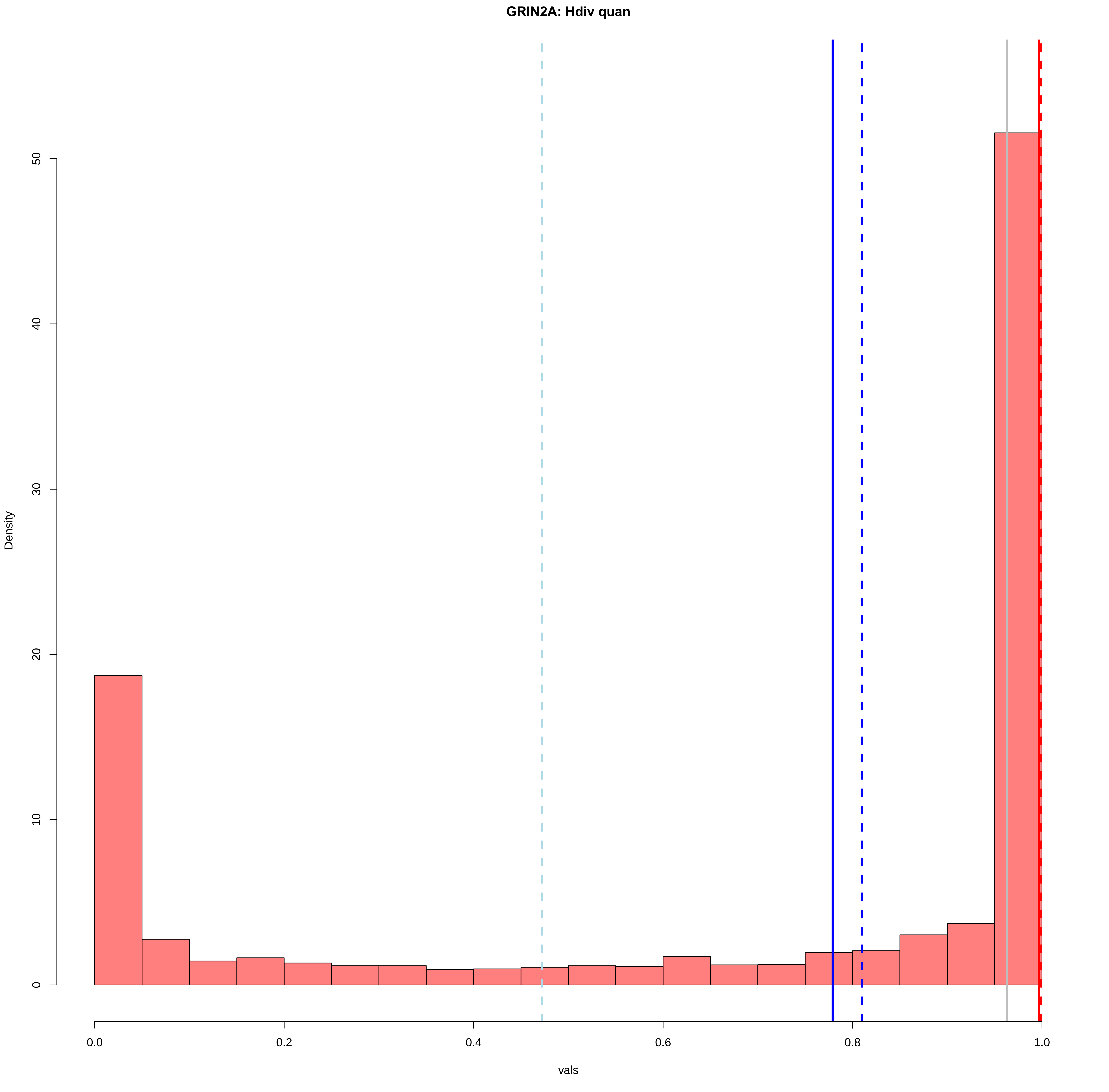

GRIN2A: Hdiv quan
Density
vals
0
10
20
30
40
50
0.0
0.2
0.4
0.6
0.8
1.0

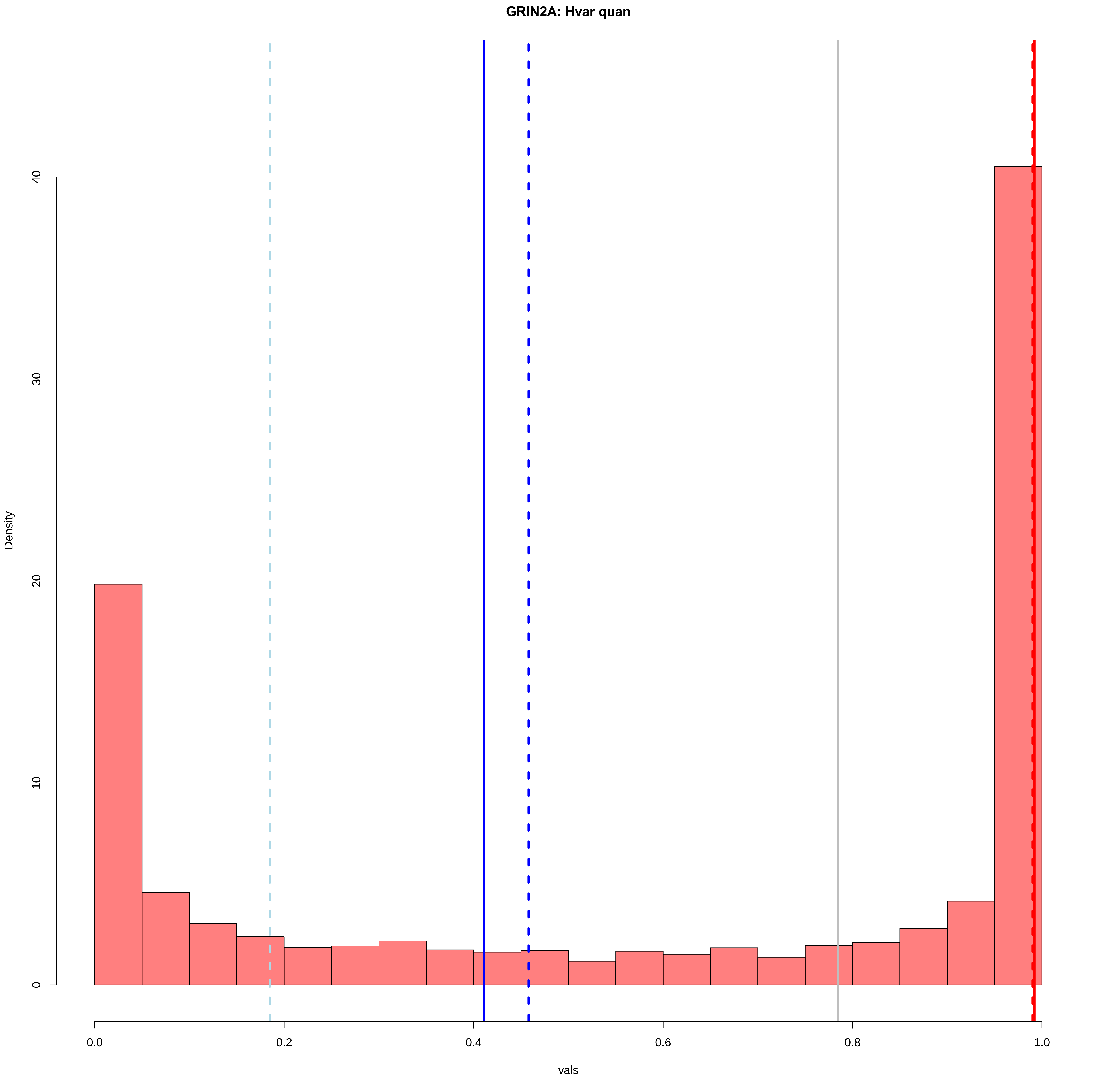
GRIN2A: Hvar quan
Density
vals
0
10
20
30
40
0.0
0.2
0.4
0.6
0.8
1.0

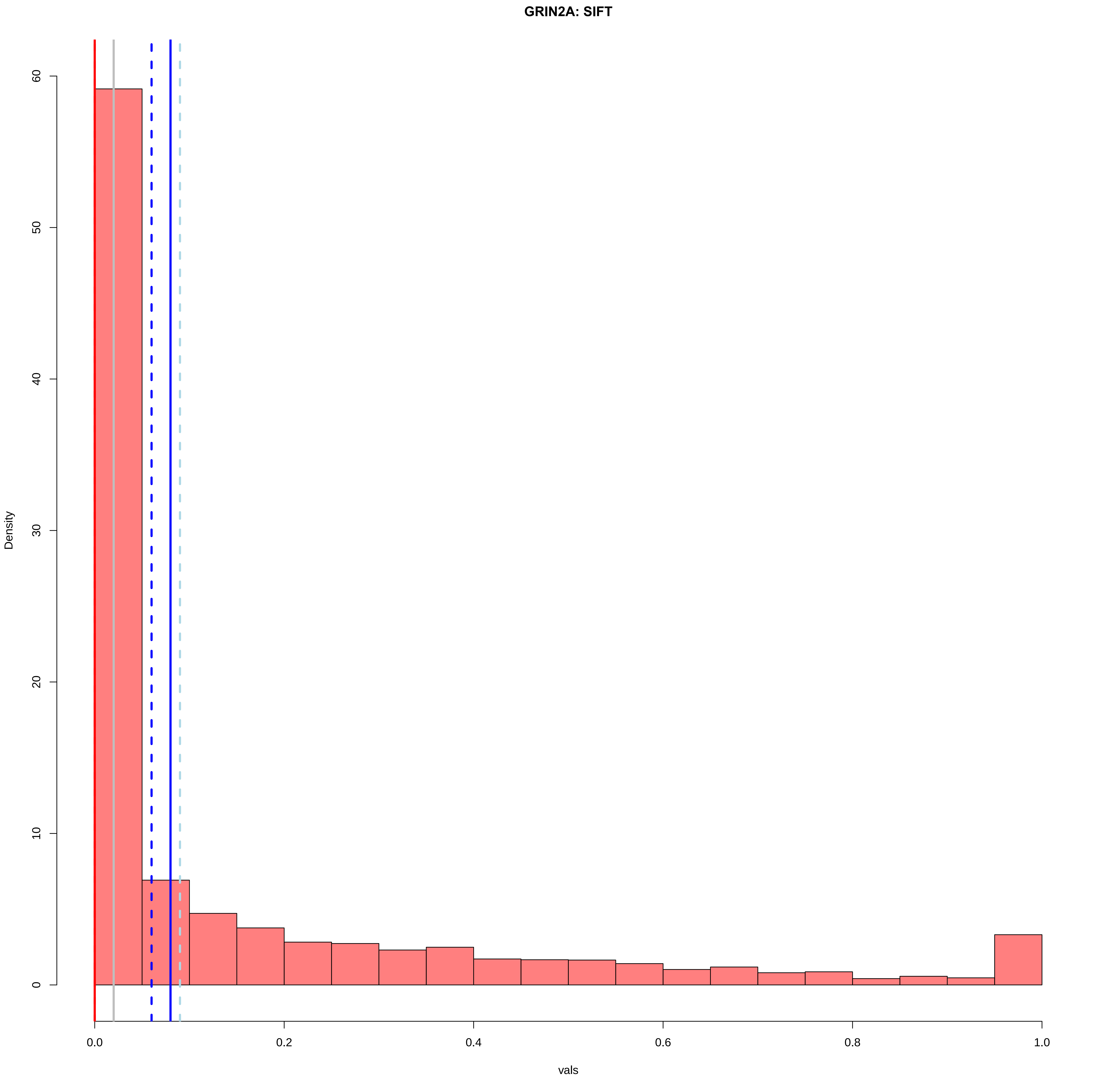

GRIN2A: SIFT
Density
vals
0
10
20
30
40
50
60
0.0
0.2
0.4
0.6
0.8
1.0

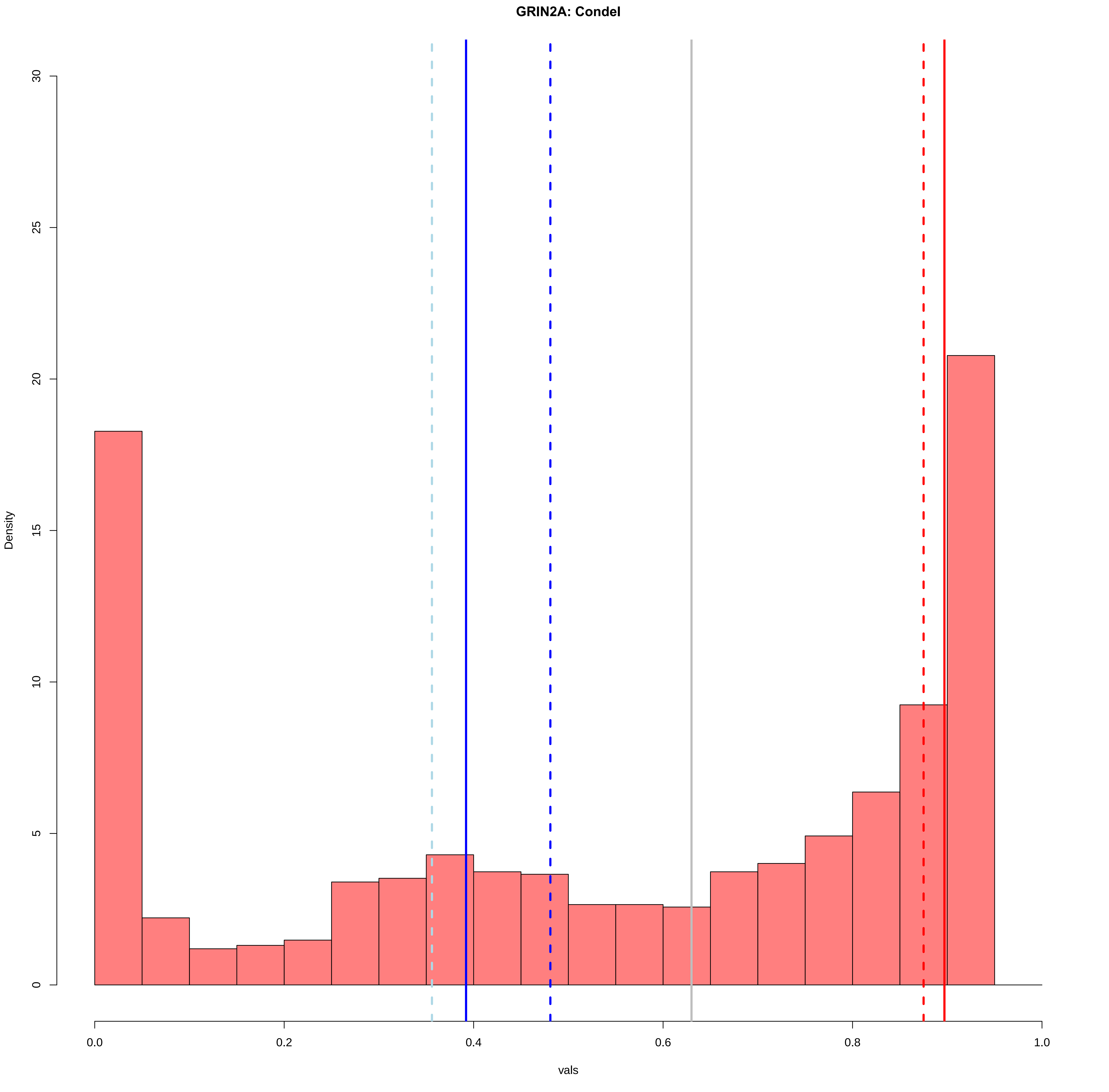

GRIN2A: Condel
Density
vals
0
5
10
15
20
25
30
0.0
0.2
0.4
0.6
0.8
1.0

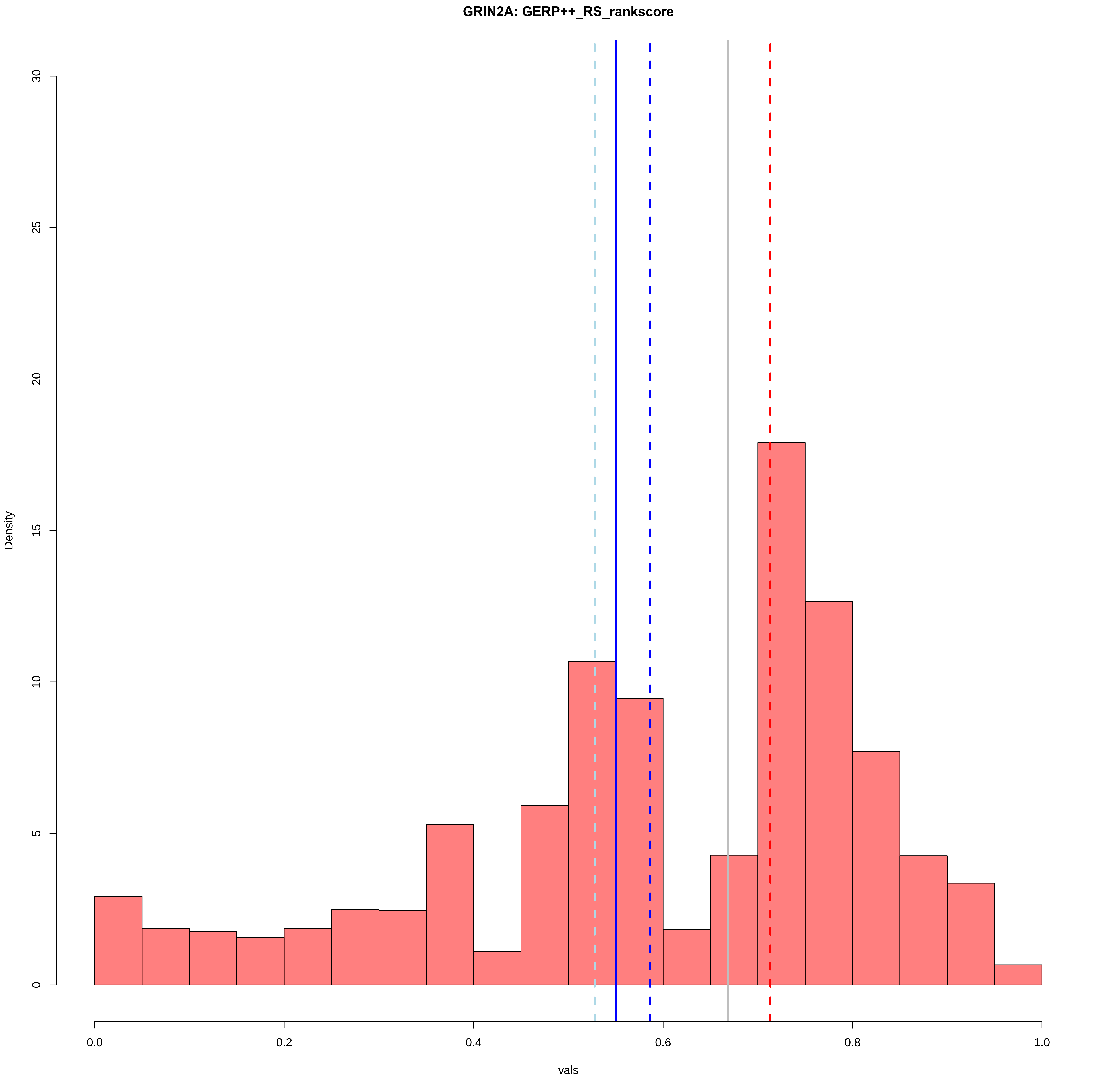
GRIN2A: GERP++_RS_rankscore
Density
vals
0
5
10
15
20
25
30
0.0
0.2
0.4
0.6
0.8
1.0

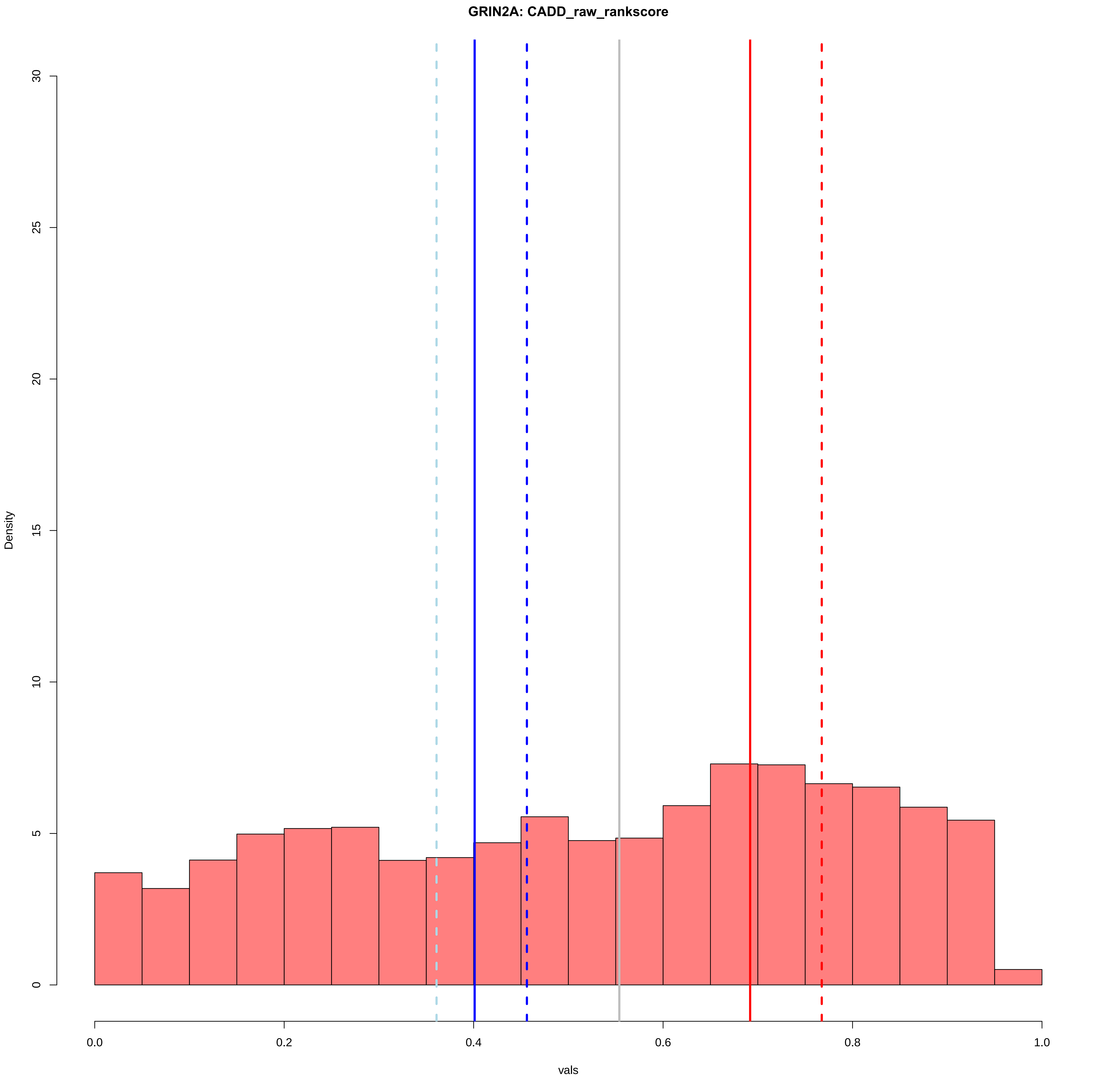
GRIN2A: CADD_raw_rankscore
Density
vals
0
5
10
15
20
25
30
0.0
0.2
0.4
0.6
0.8
1.0

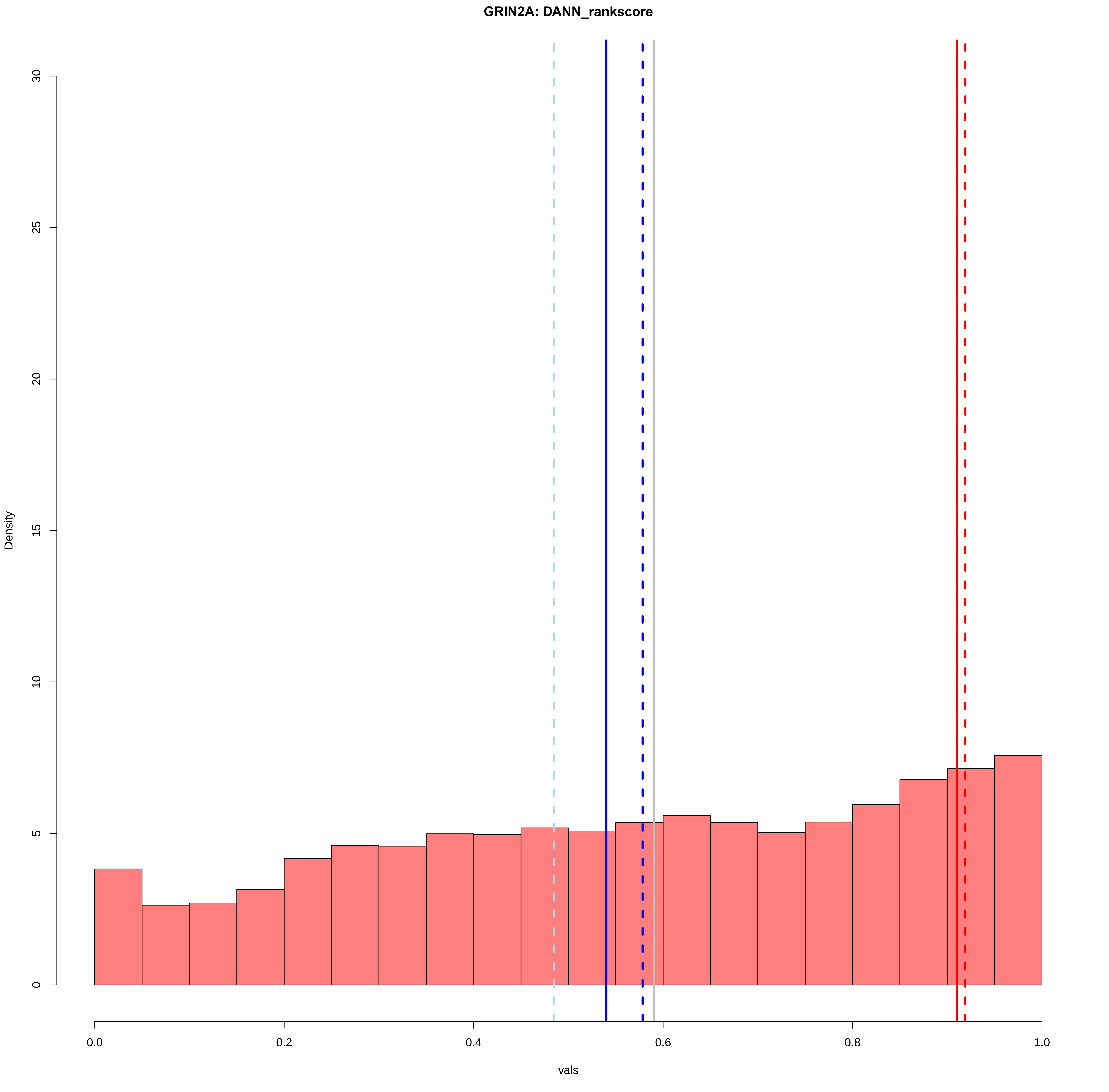

GRIN2A: DANN_rankscore
Density
vals
0
5
10
15
20
25
30
0.0
0.2
0.4
0.6
0.8
1.0

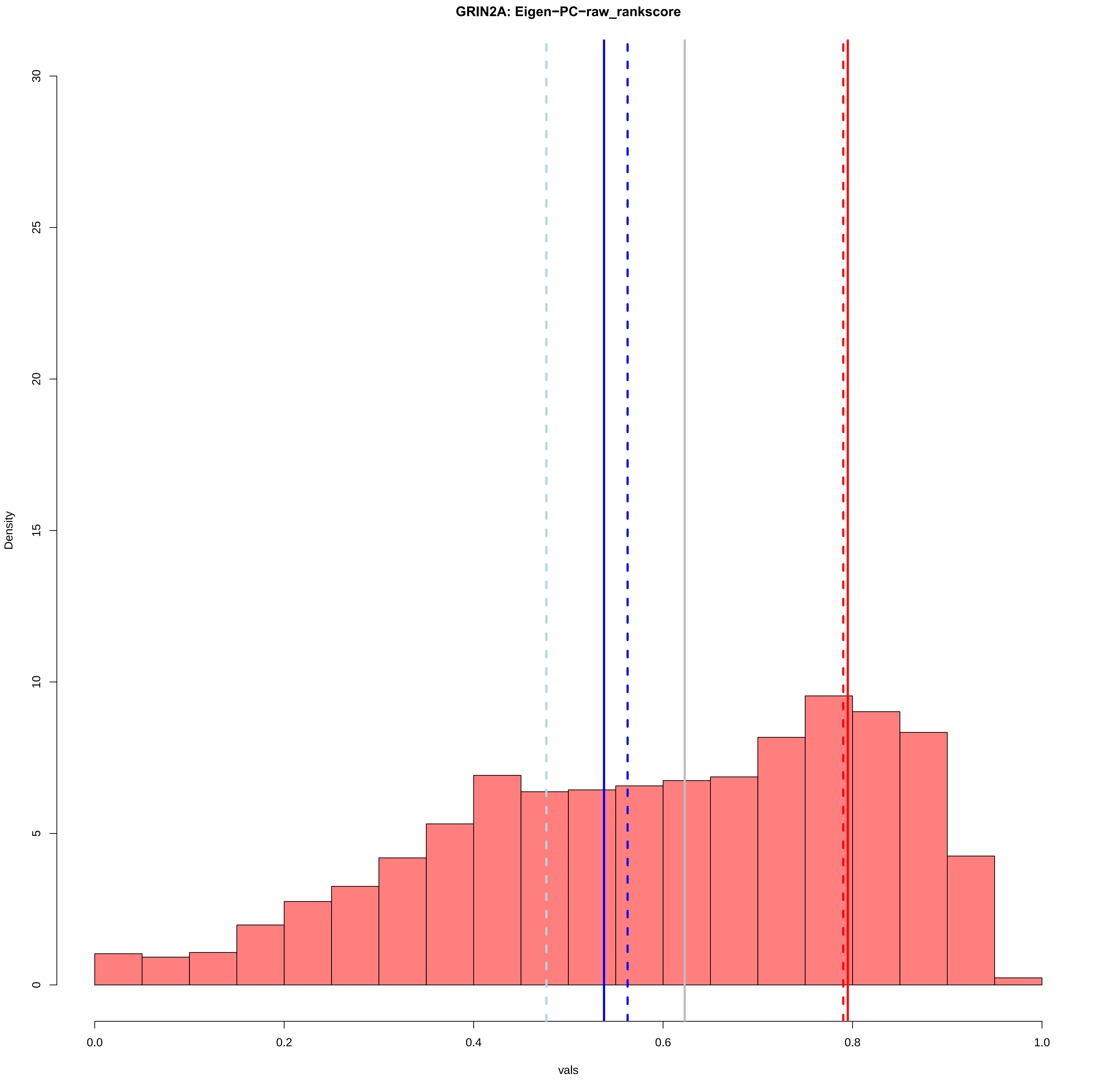

GRIN2A: Eigen−PC−raw_rankscore
Density
vals
0
5
10
15
20
25
30
0.0
0.2
0.4
0.6
0.8
1.0

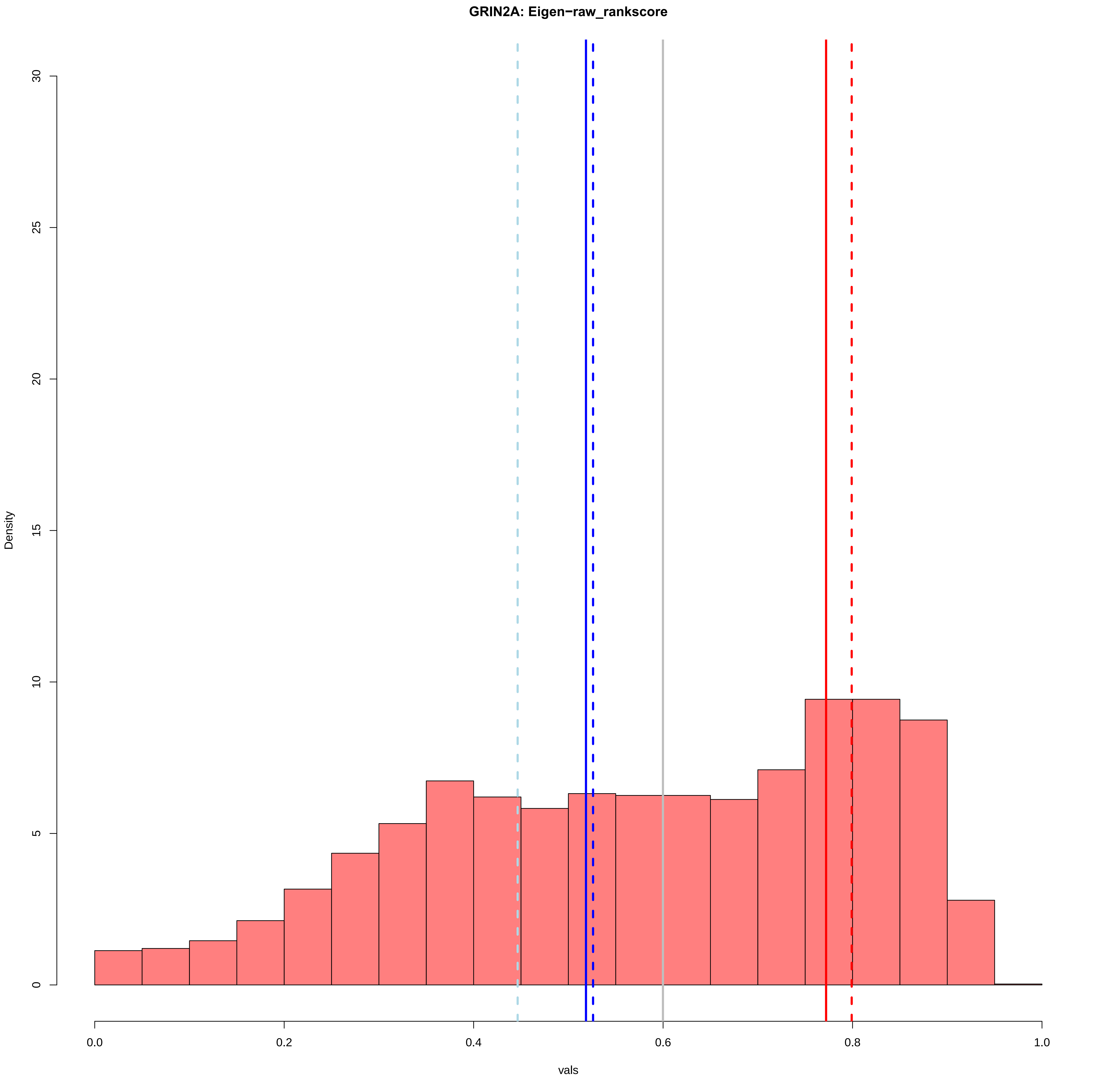

GRIN2A: Eigen−raw_rankscore
Density
vals
0
5
10
15
20
25
30
0.0
0.2
0.4
0.6
0.8
1.0

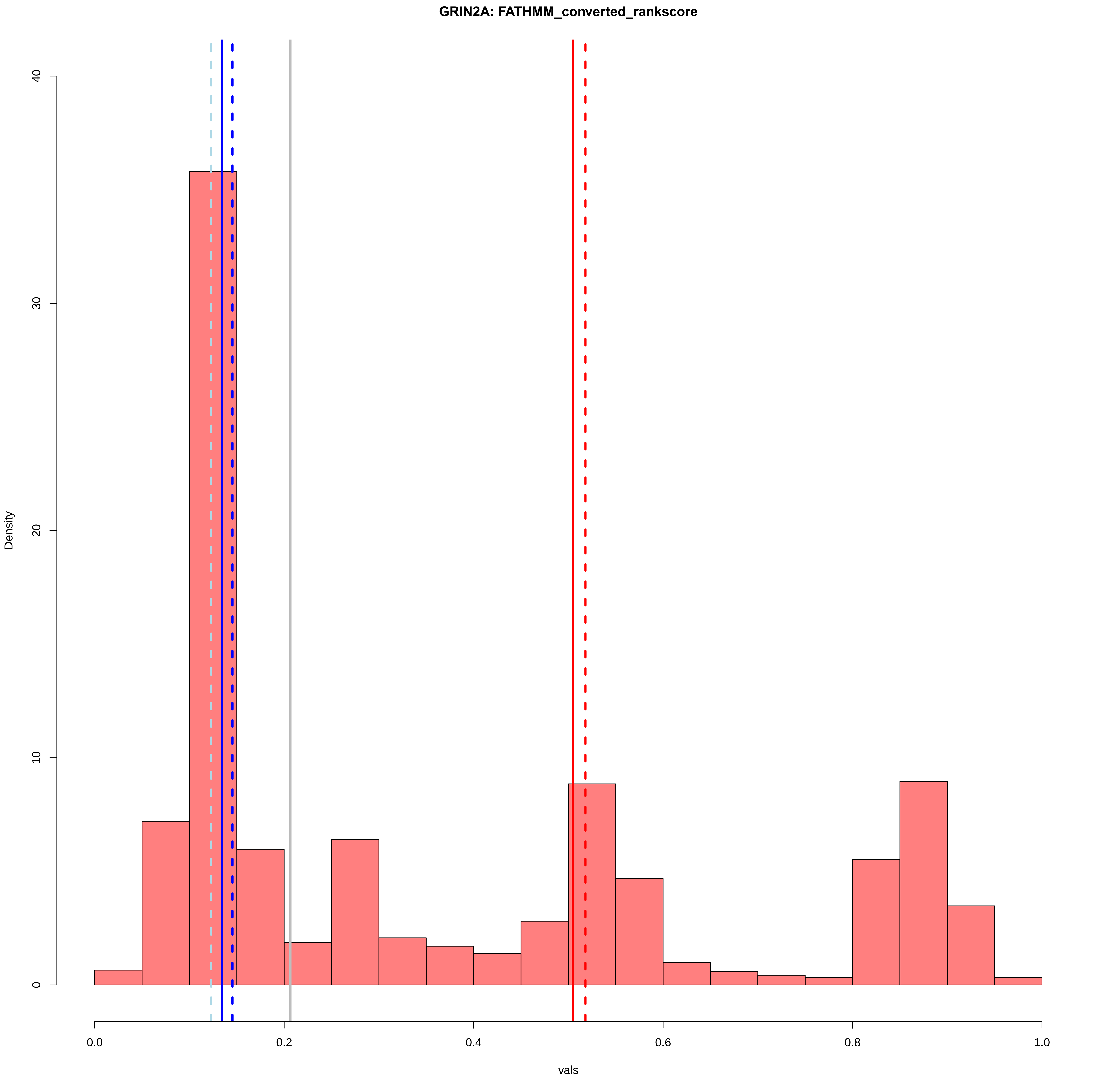

GRIN2A: FATHMM_converted_rankscore
Density
vals
0
10
20
30
40
0.0
0.2
0.4
0.6
0.8
1.0

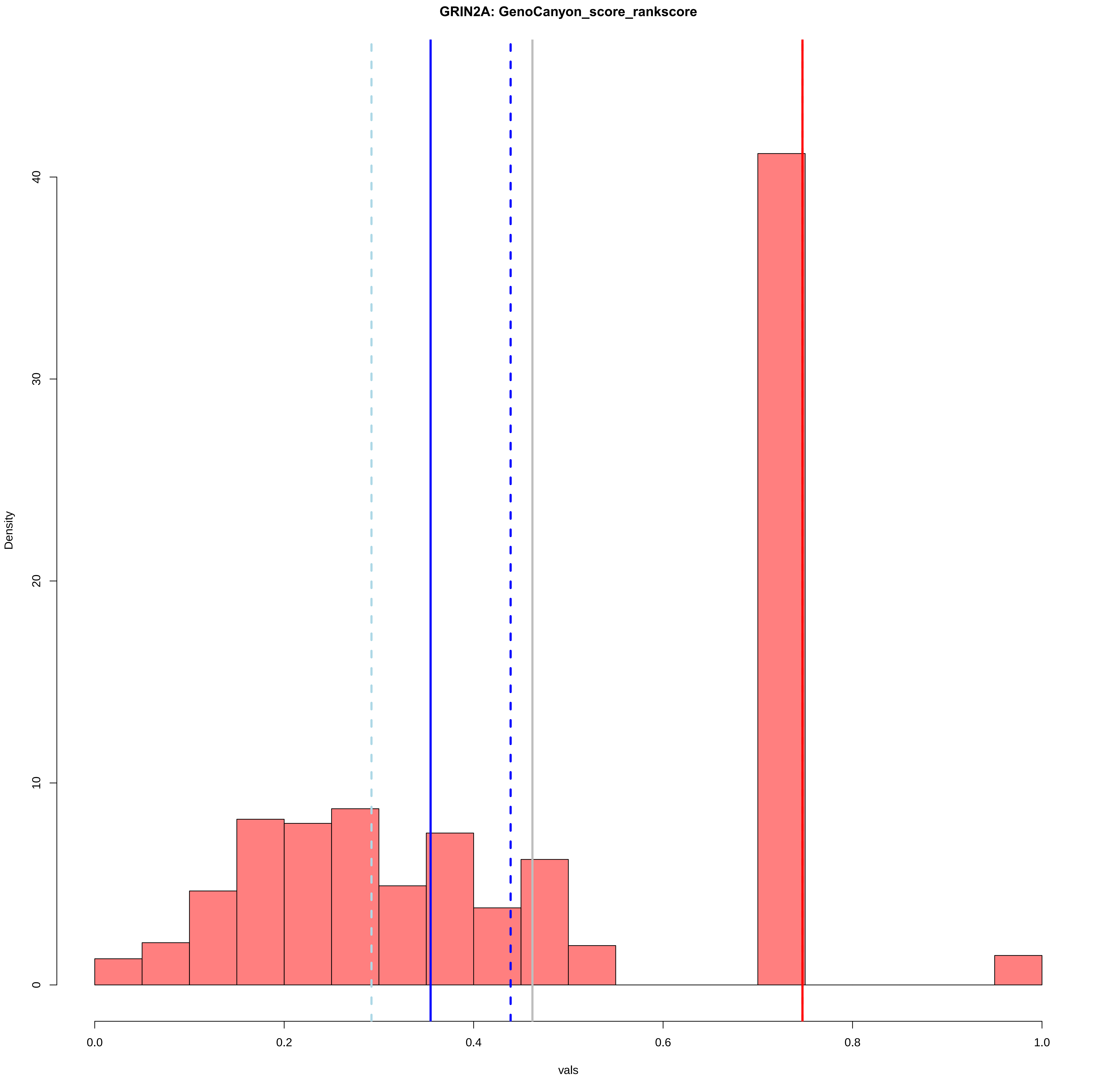

GRIN2A: GenoCanyon_score_rankscore
Density
0
10
20
30
40
0.0
0.2
0.4
0.6
0.8
1.0
vals

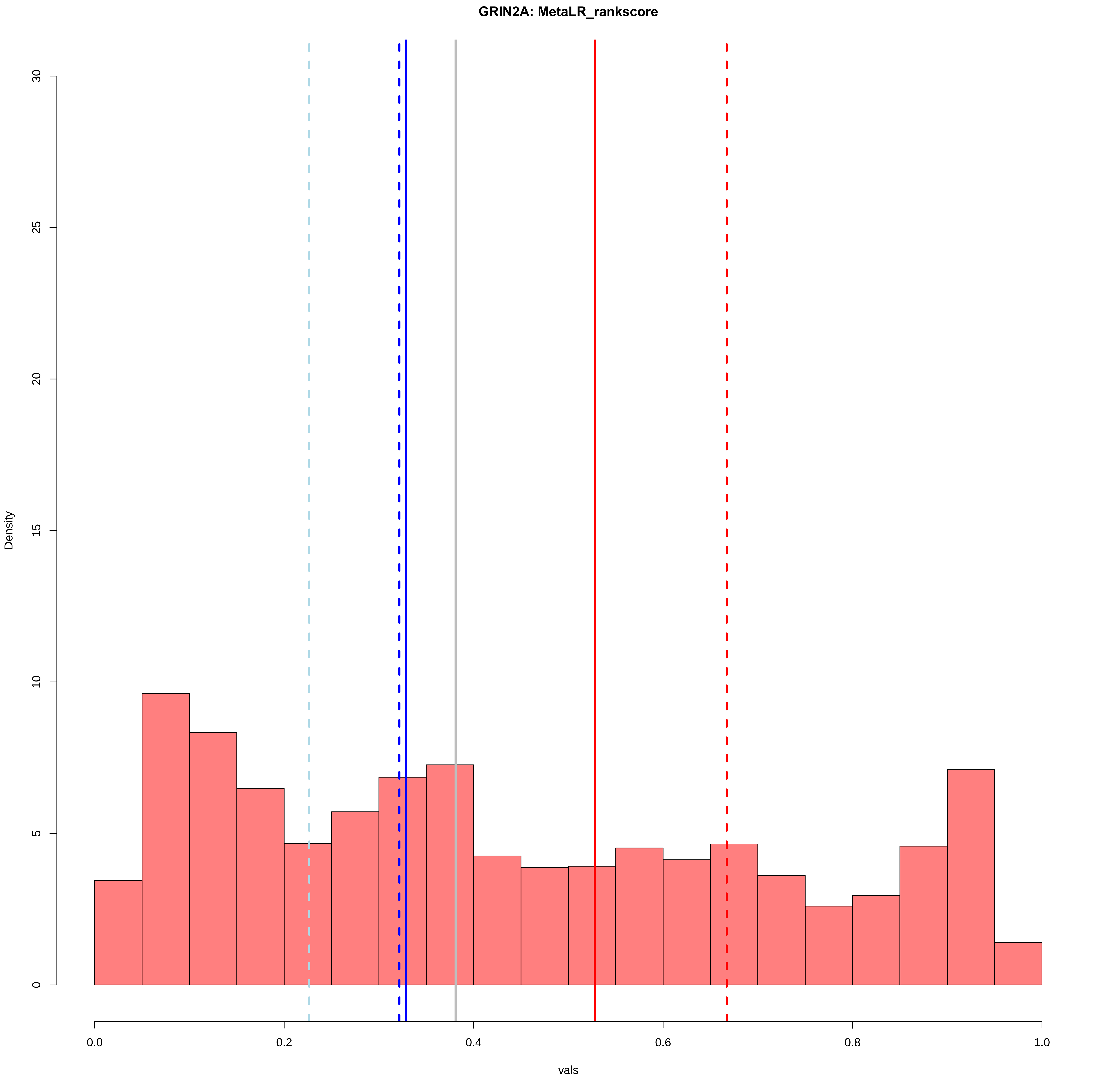

GRIN2A: MetaLR_rankscore
Density
0
5
10
15
20
25
30
0.0
0.2
0.4
0.6
0.8
1.0
vals

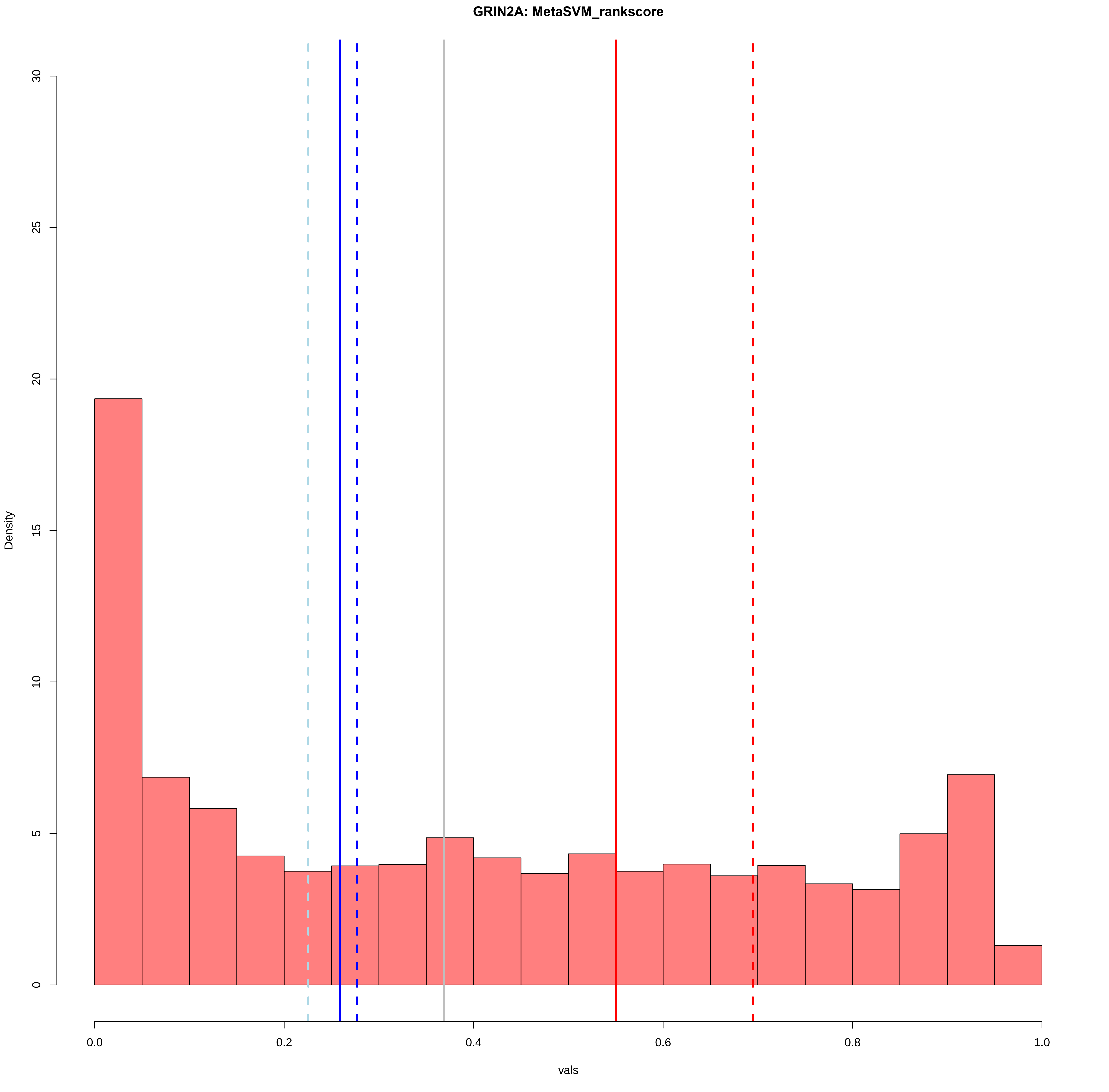

GRIN2A: MetaSVM_rankscore
Density
vals
0
5
10
15
20
25
30
0.0
0.2
0.4
0.6
0.8
1.0

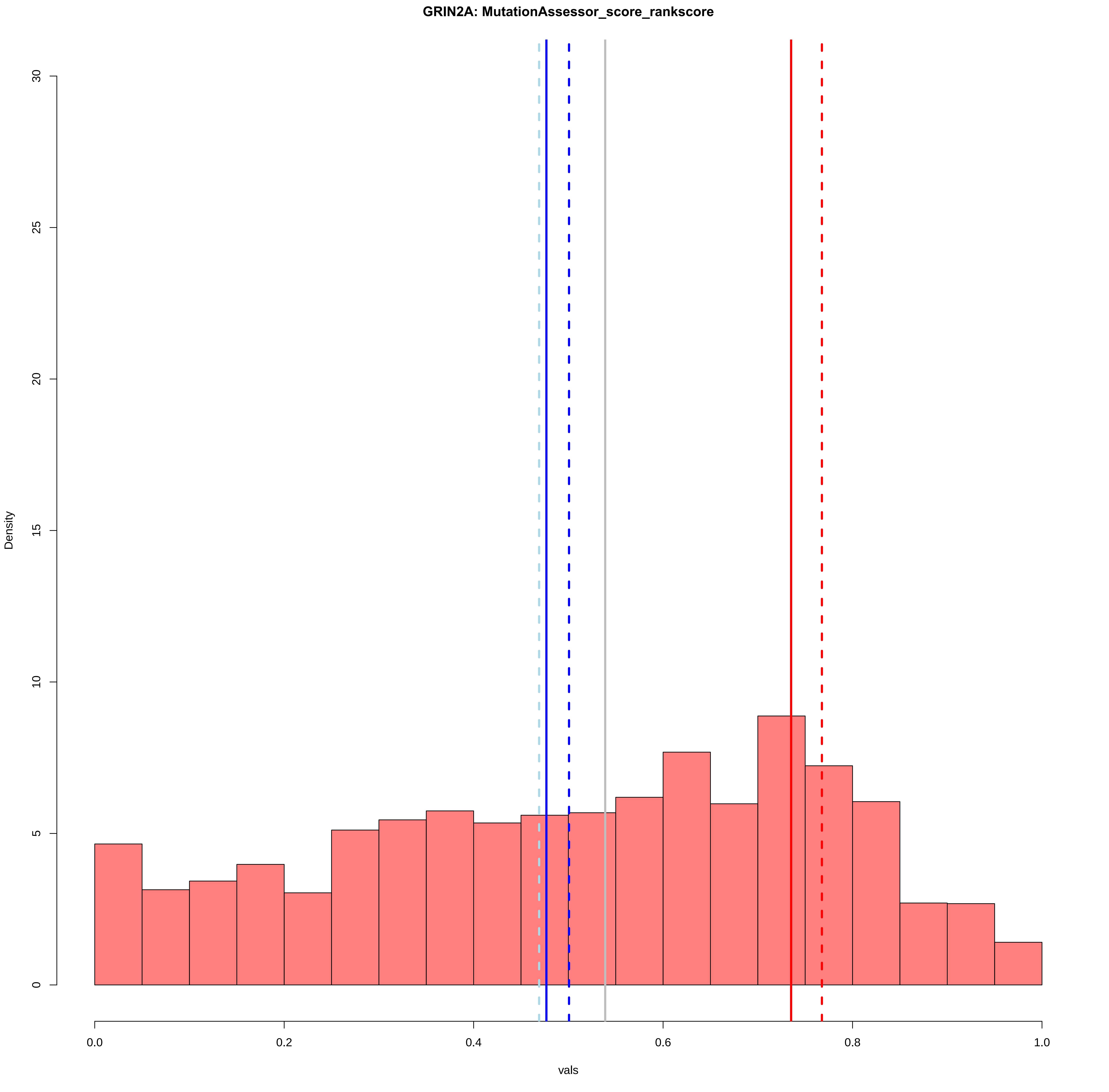

GRIN2A: MutationAssessor_score_rankscore
Density
0
5
10
15
20
25
30
0.0
0.2
0.4
0.6
0.8
1.0
vals

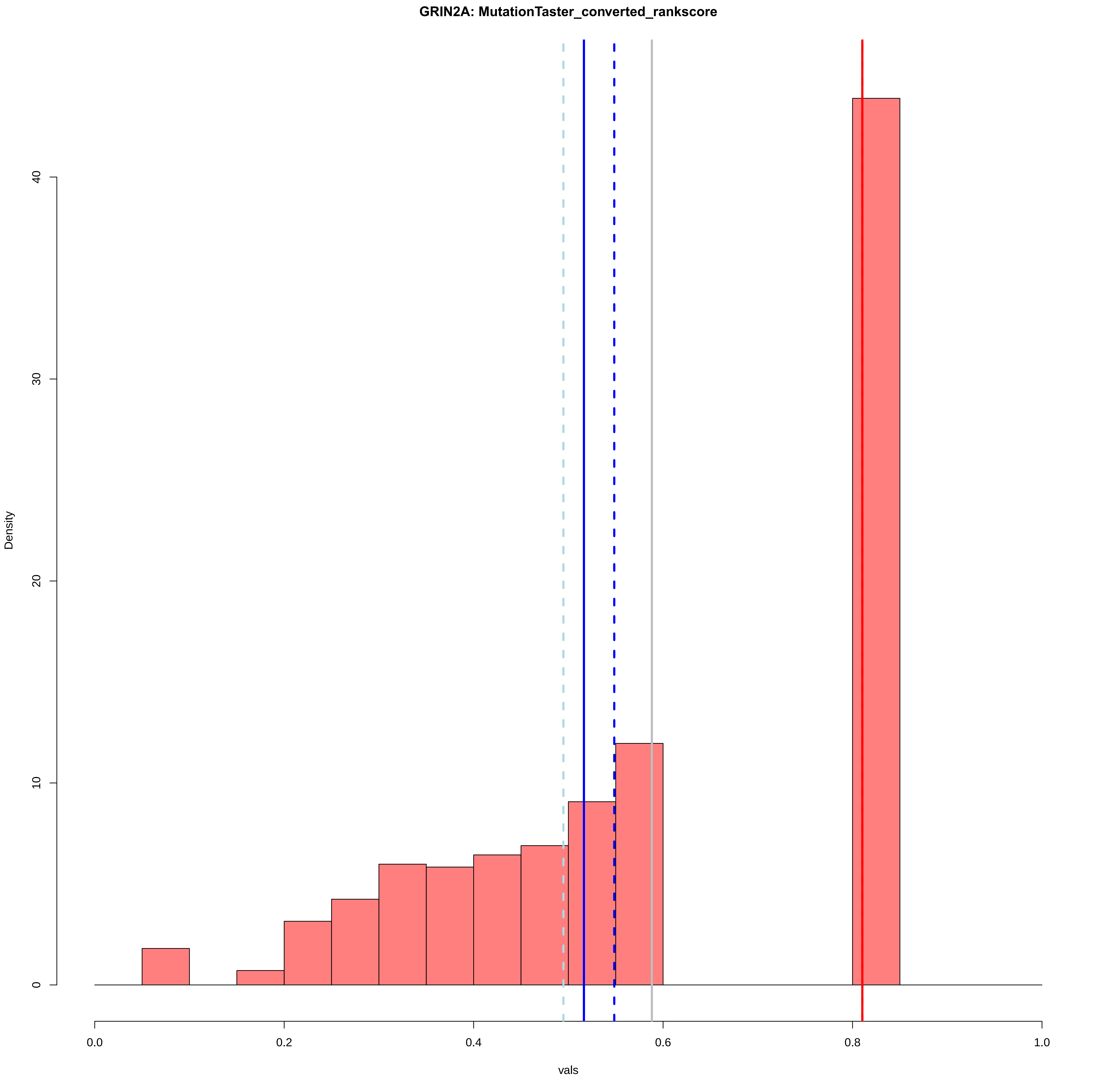
GRIN2A: MutationTaster_converted_rankscore
Density
vals
0
10
20
30
40
0.0
0.2
0.4
0.6
0.8
1.0

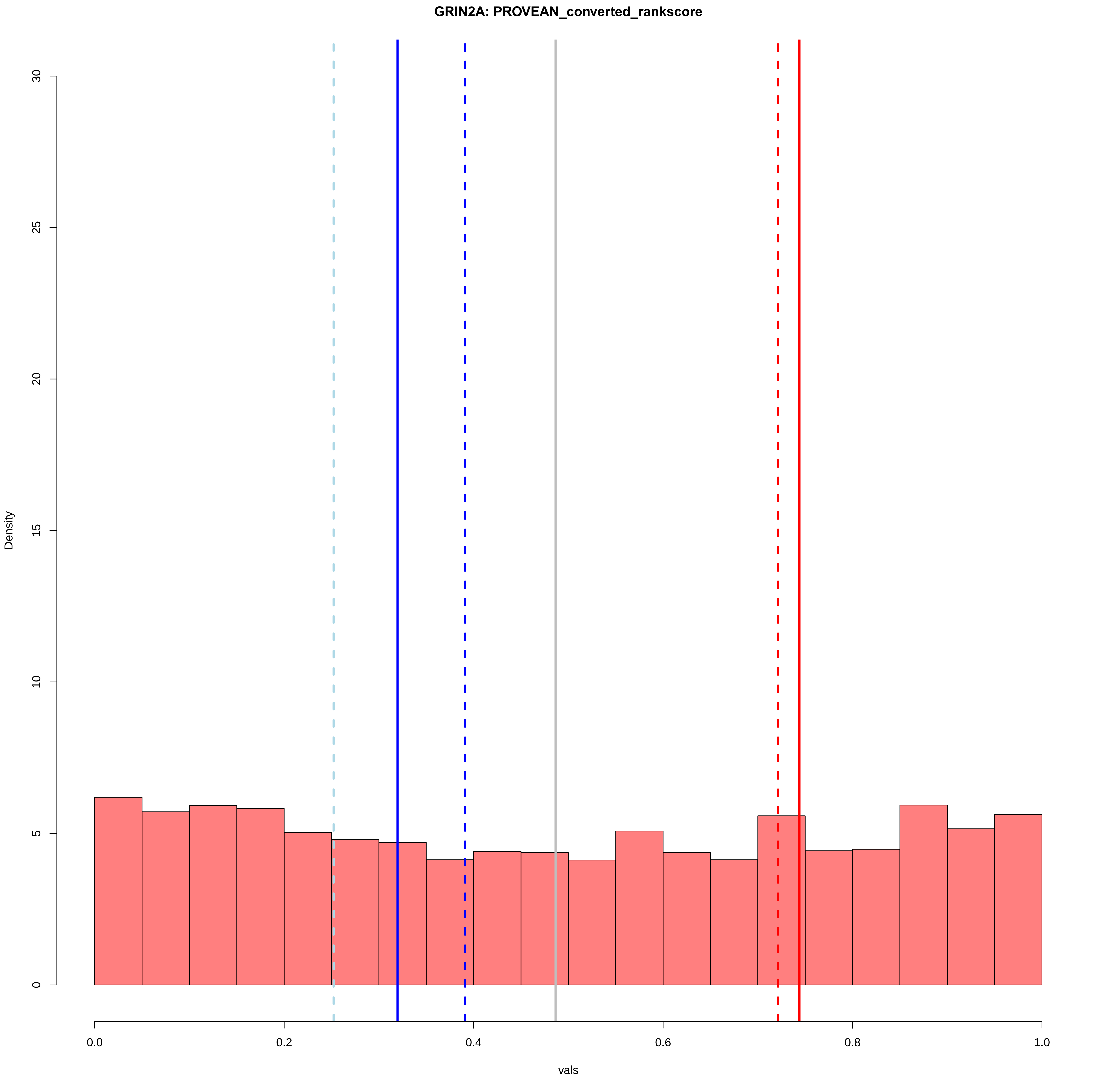
GRIN2A: PROVEAN_converted_rankscore
Density
vals
0
5
10
15
20
25
30
0.0
0.2
0.4
0.6
0.8
1.0

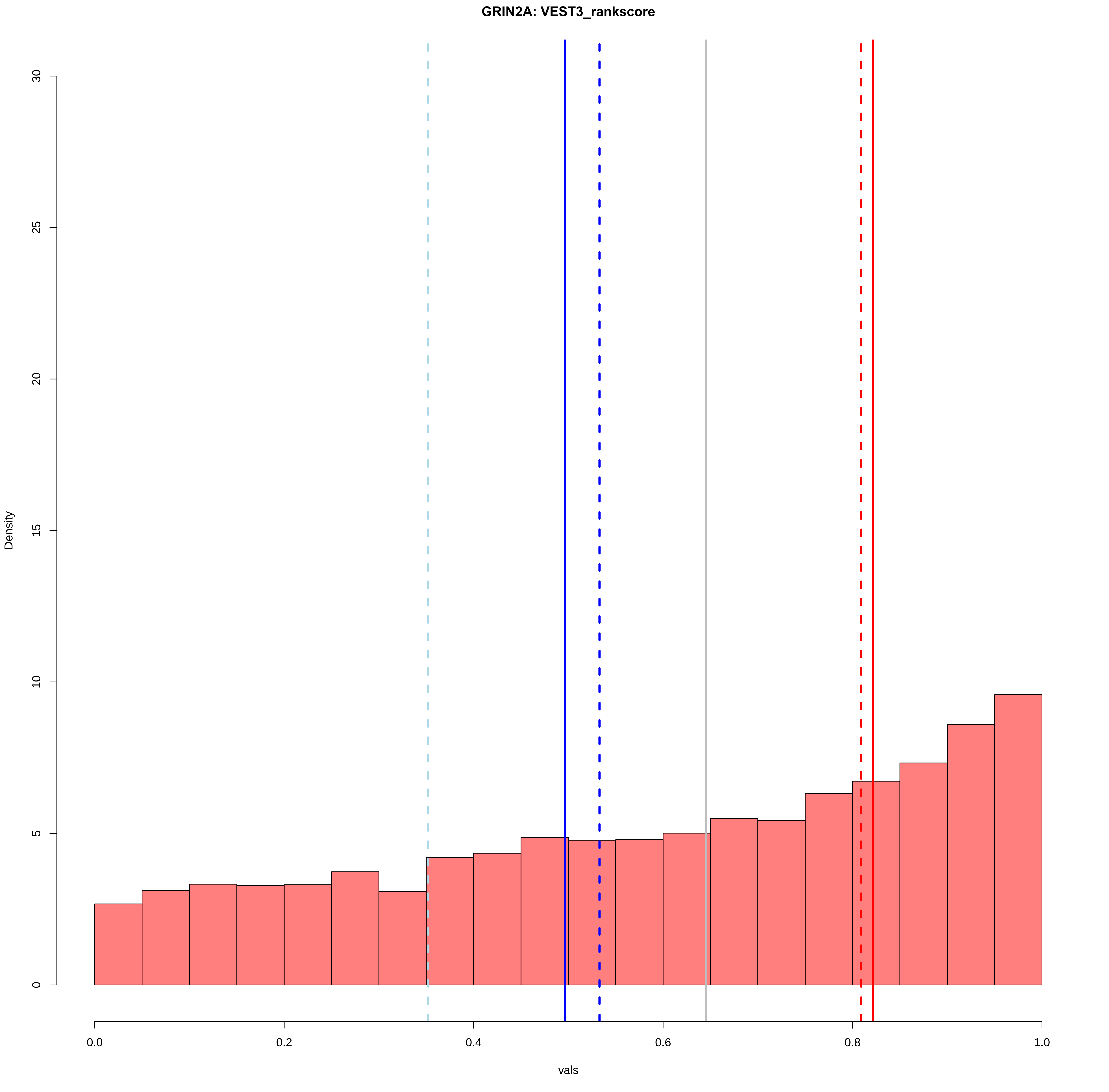

GRIN2A: VEST3_rankscore
Density
vals
0
5
10
15
20
25
30
0.0
0.2
0.4
0.6
0.8
1.0

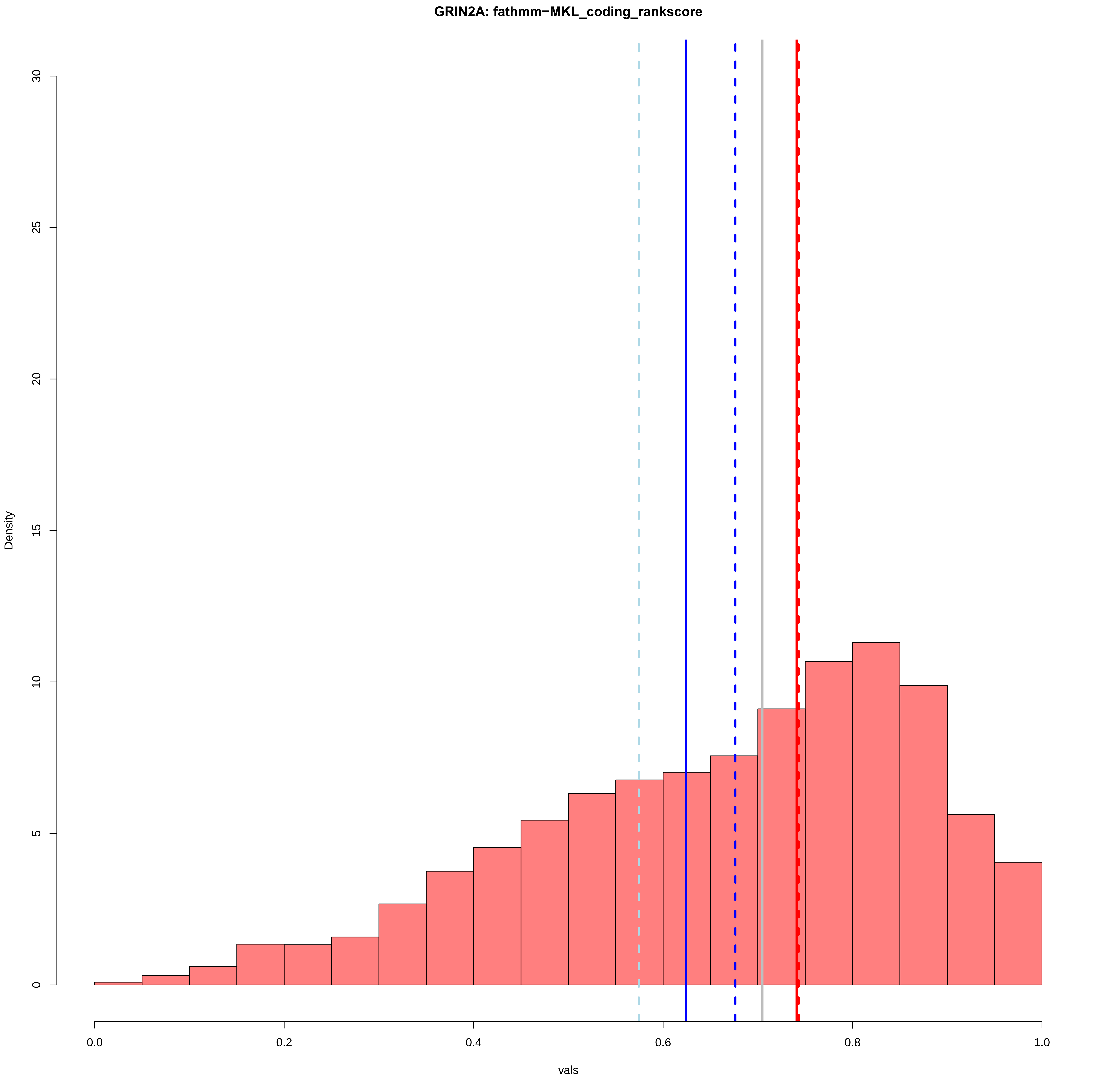

GRIN2A: fathmm−MKL_coding_rankscore
Density
vals
0
5
10
15
20
25
30
0.0
0.2
0.4
0.6
0.8
1.0

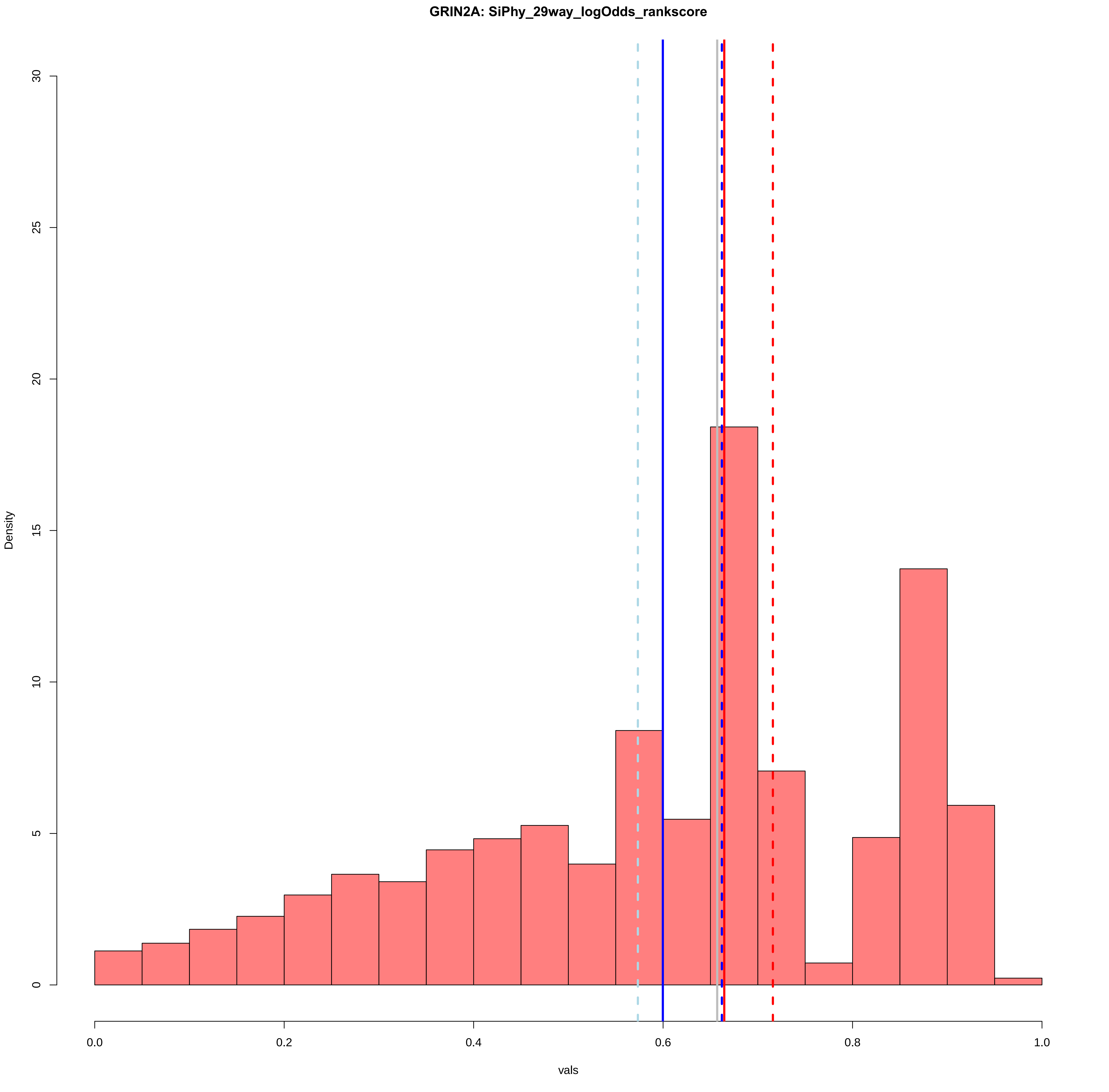

GRIN2A: SiPhy_29way_logOdds_rankscore
Density
vals
0
5
10
15
20
25
30
0.0
0.2
0.4
0.6
0.8
1.0

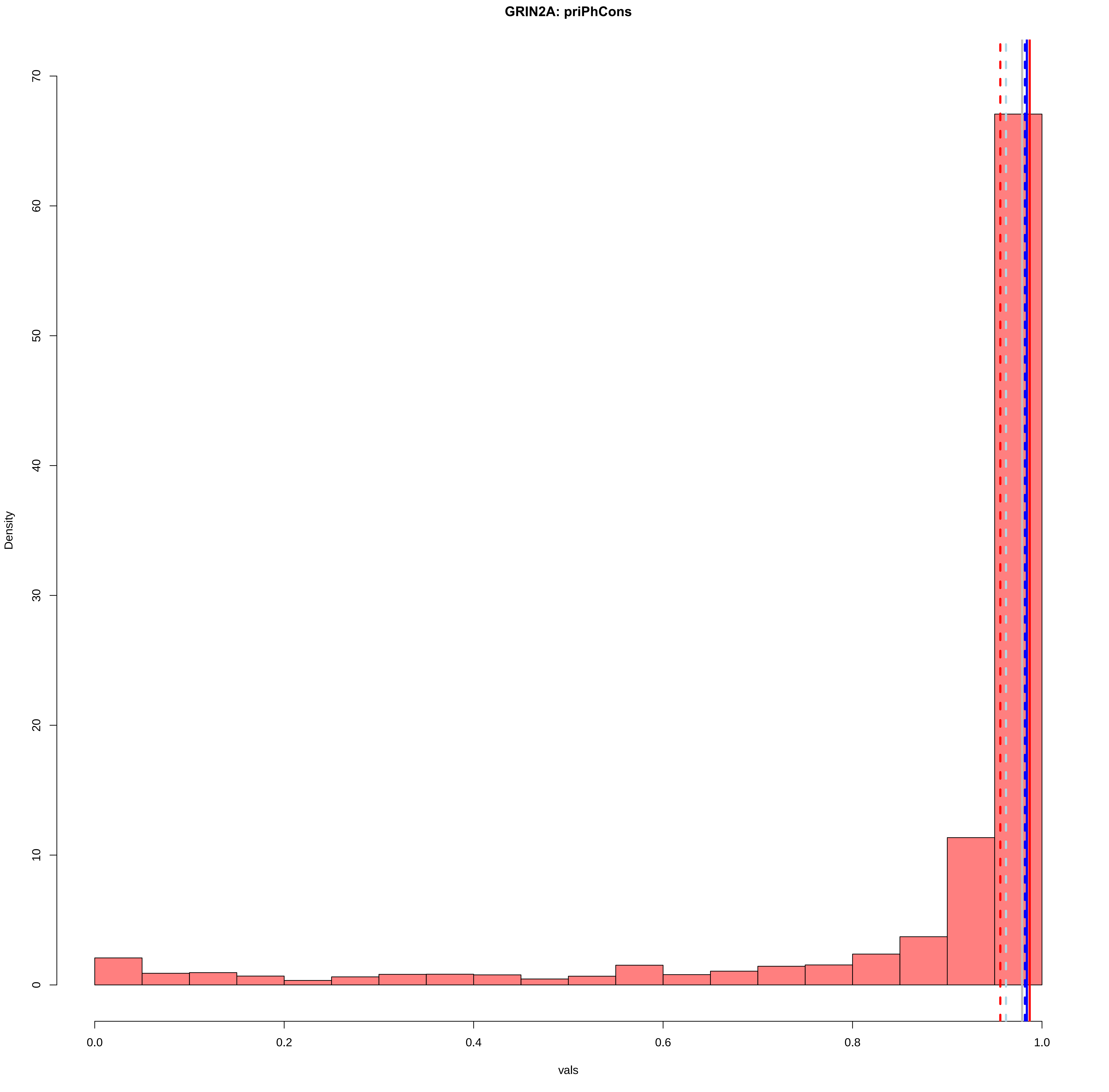

GRIN2A: priPhCons
Density
vals
0
10
20
30
40
50
60
70
0.0
0.2
0.4
0.6
0.8
1.0

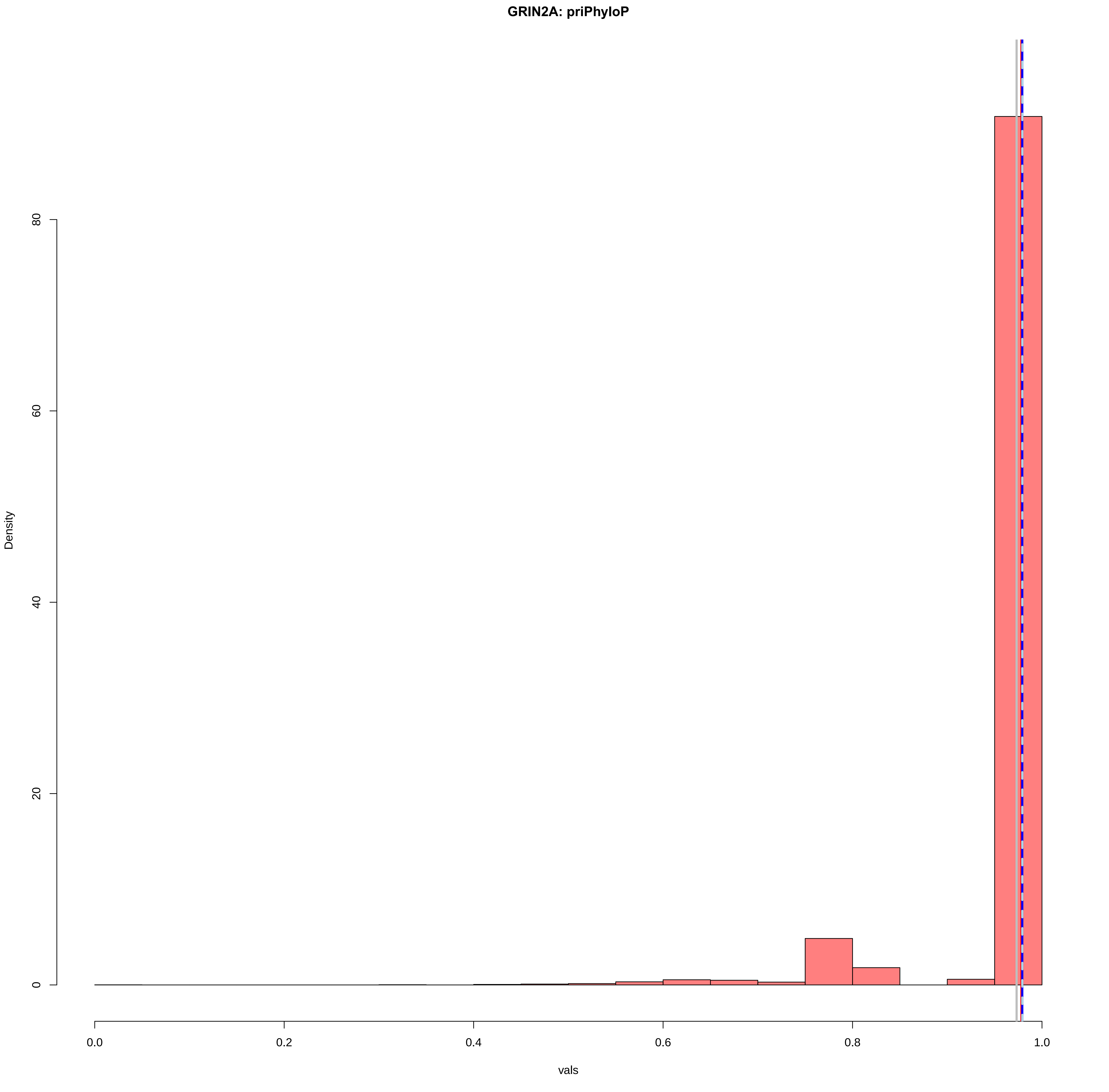

GRIN2A: priPhyloP
Density
vals
0
20
40
60
80
0.0
0.2
0.4
0.6
0.8
1.0

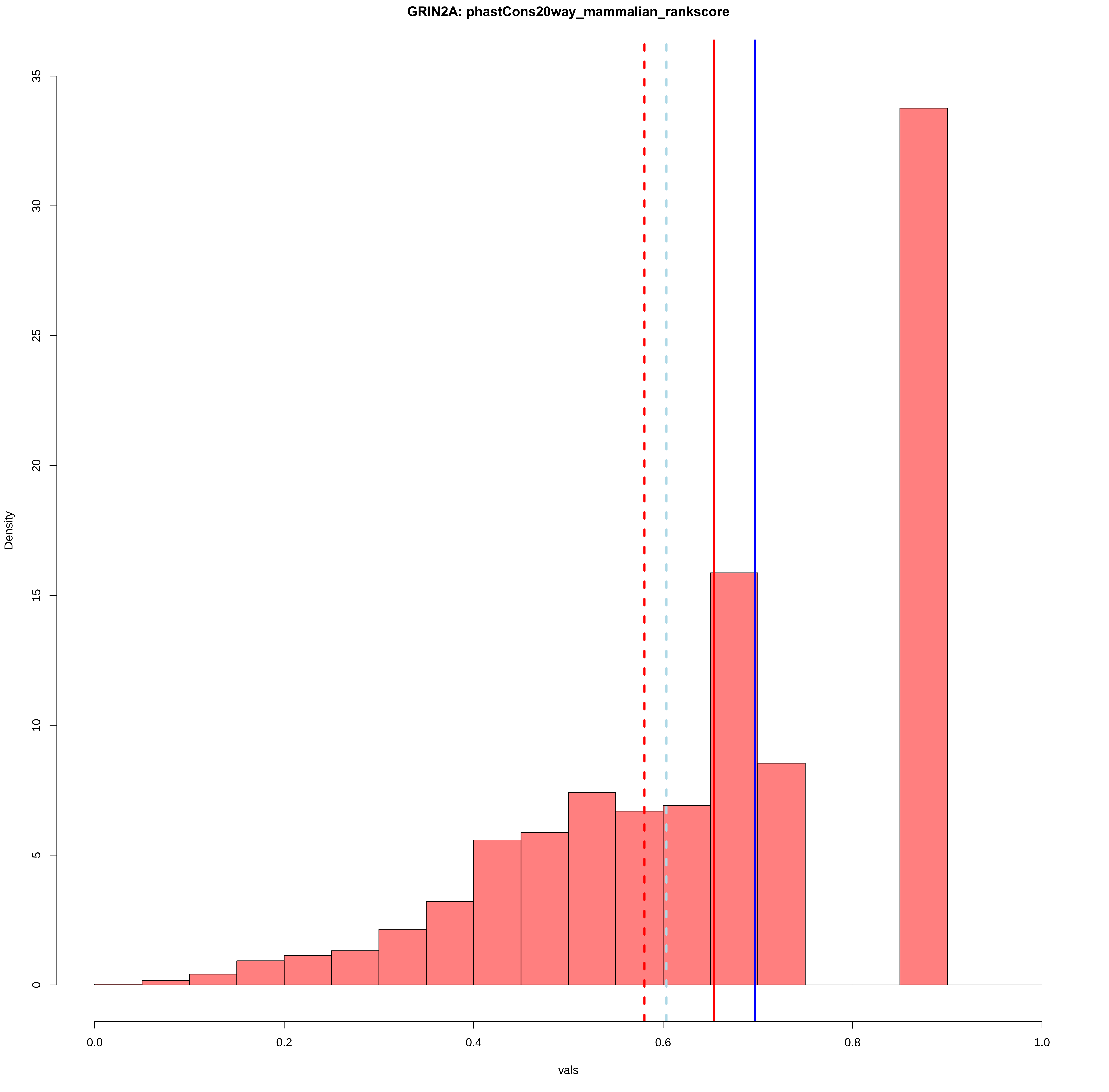

GRIN2A: phastCons20way_mammalian_rankscore
Density
0
5
10
15
20
25
30
35
0.0
0.2
0.4
0.6
0.8
1.0
vals

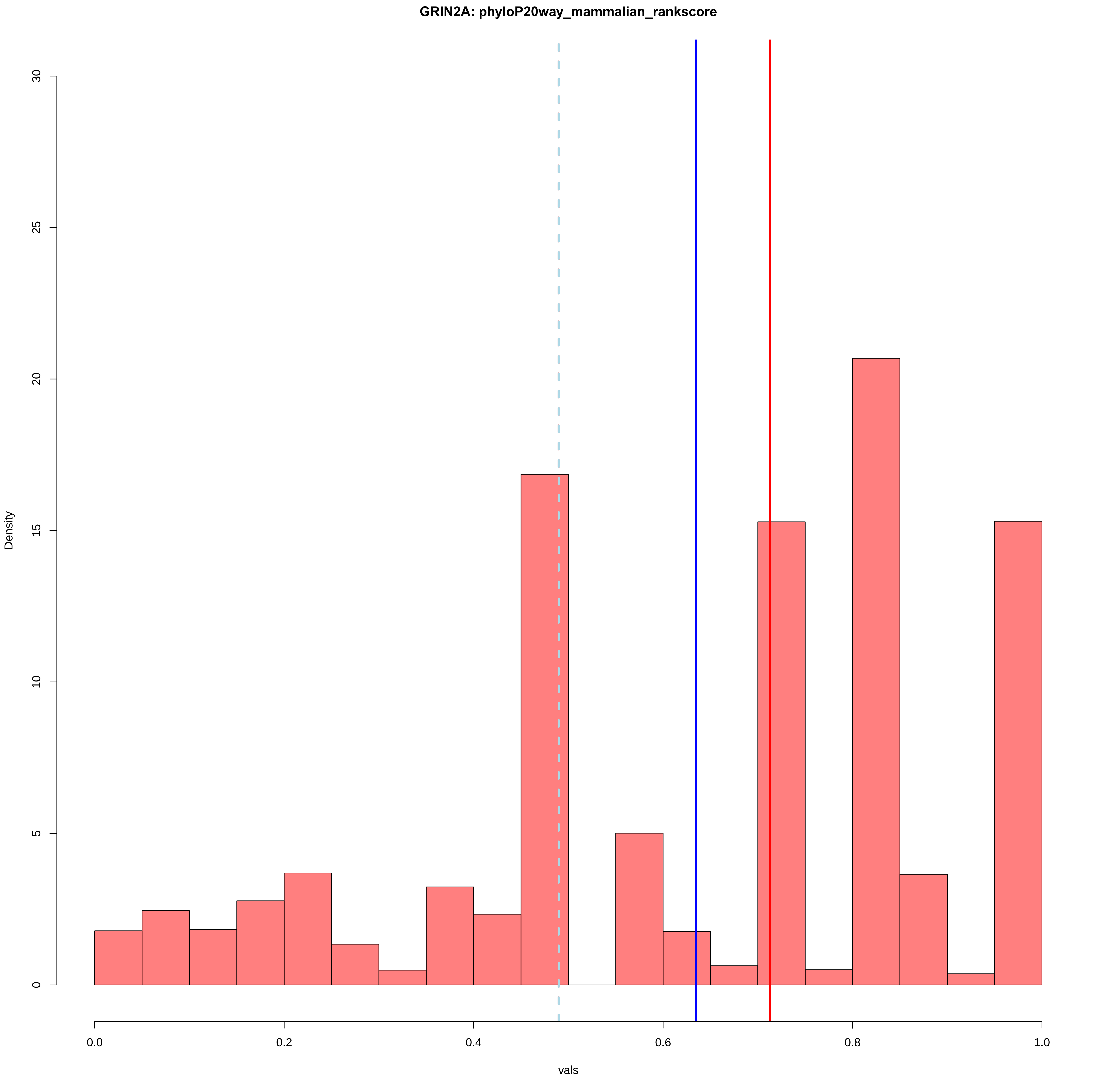
GRIN2A: phyloP20way_mammalian_rankscore
Density
vals
0
5
10
15
20
25
30
0.0
0.2
0.4
0.6
0.8
1.0

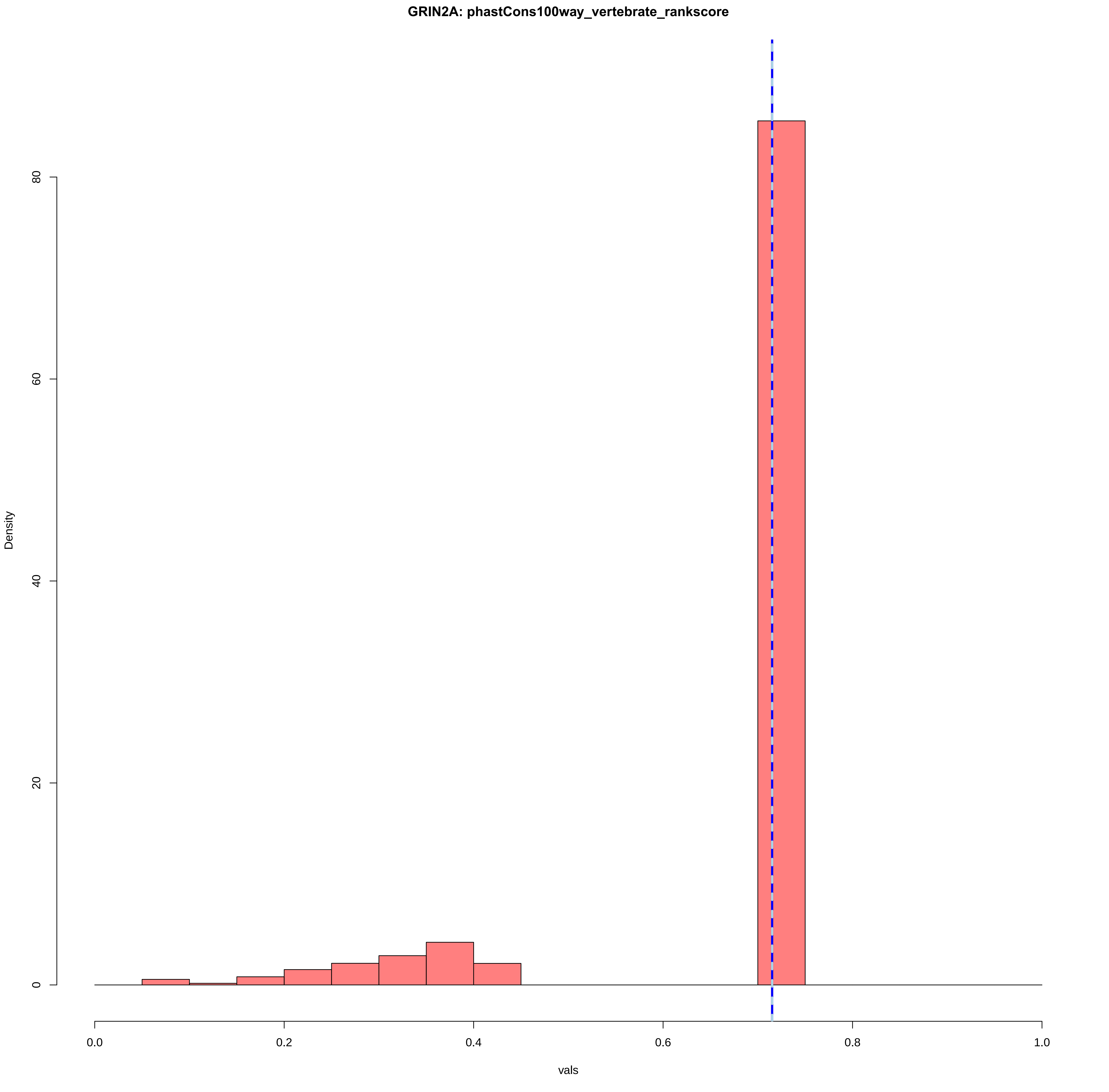
GRIN2A: phastCons100way_vertebrate_rankscore
Density
vals
0
20
40
60
80
0.0
0.2
0.4
0.6
0.8
1.0

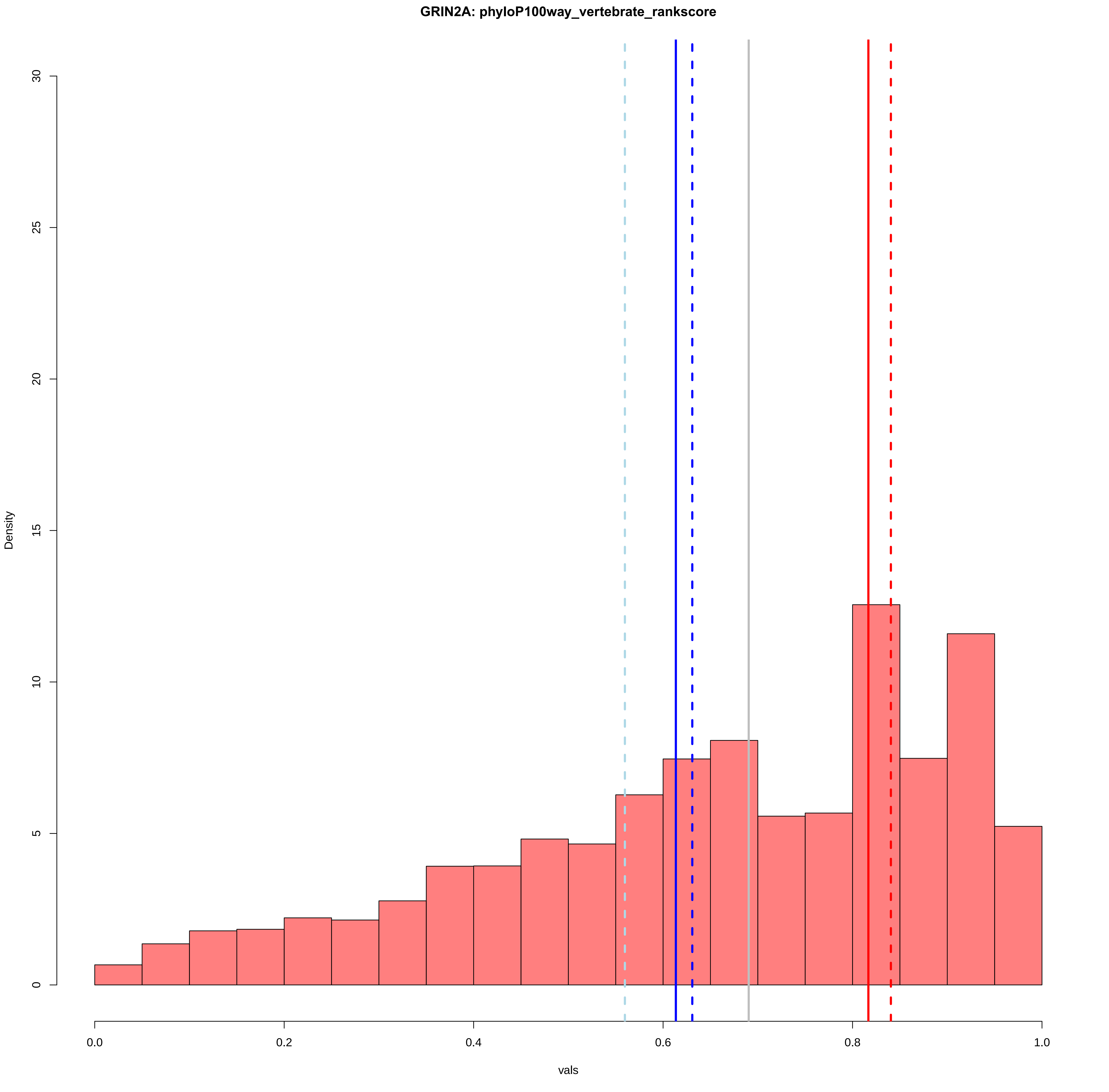

GRIN2A: phyloP100way_vertebrate_rankscore
Density
vals
0
5
10
15
20
25
30
0.0
0.2
0.4
0.6
0.8
1.0

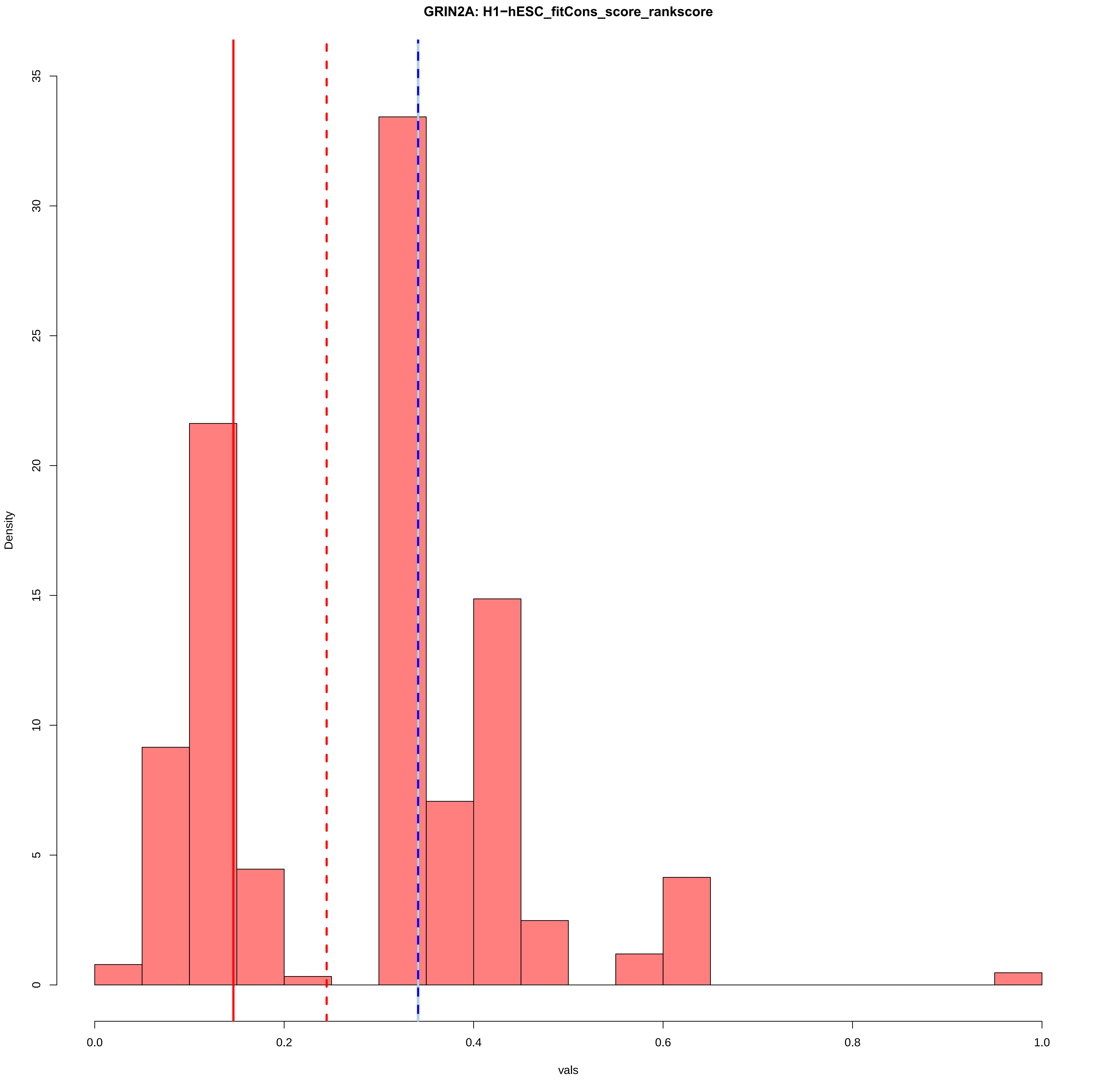
GRIN2A: H1−hESC_fitCons_score_rankscore
Density
0
5
10
15
20
25
30
35
0.0
0.2
0.4
0.6
0.8
1.0
vals

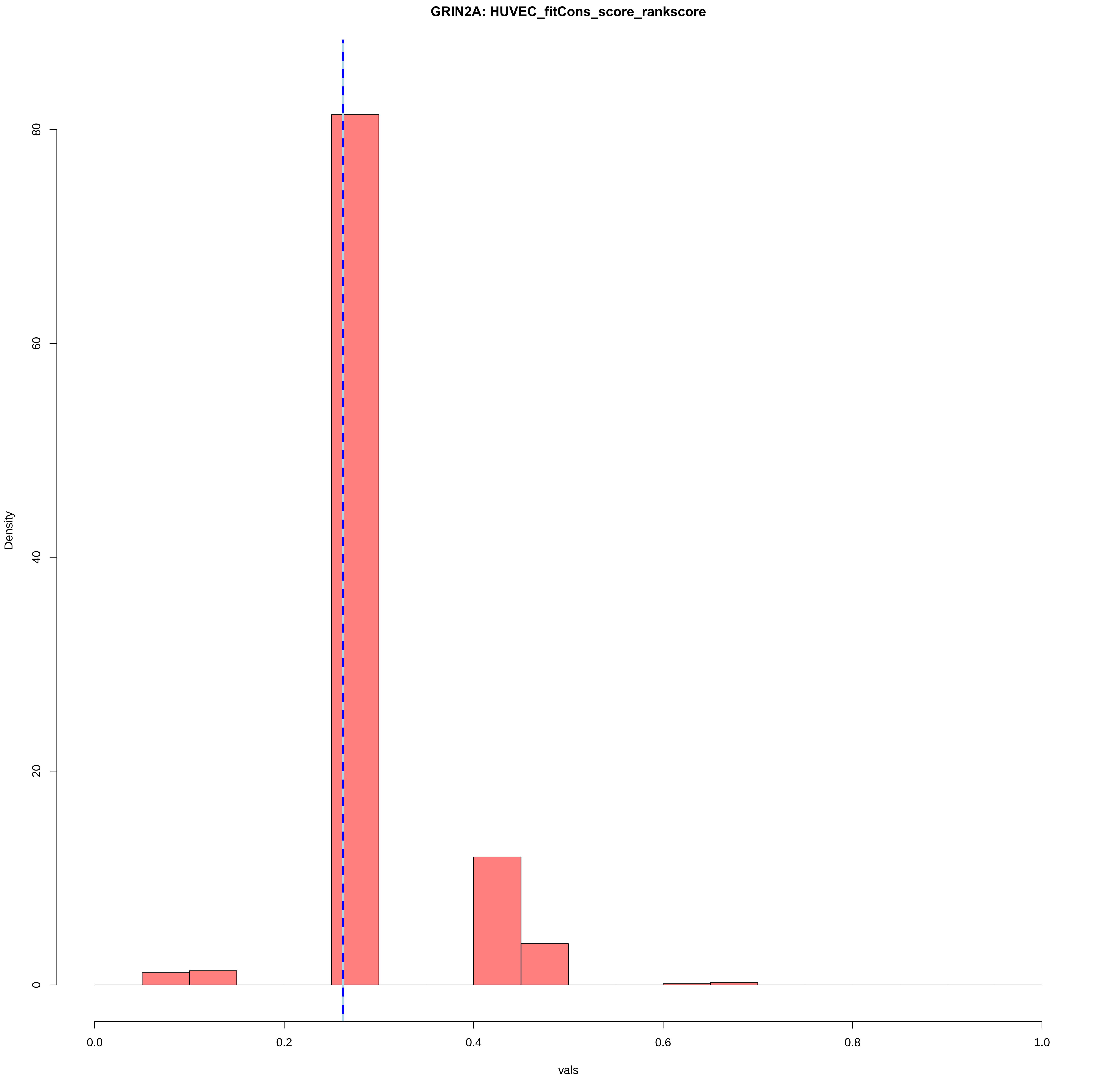
GRIN2A: HUVEC_fitCons_score_rankscore
Density
vals
0
20
40
60
80
0.0
0.2
0.4
0.6
0.8
1.0

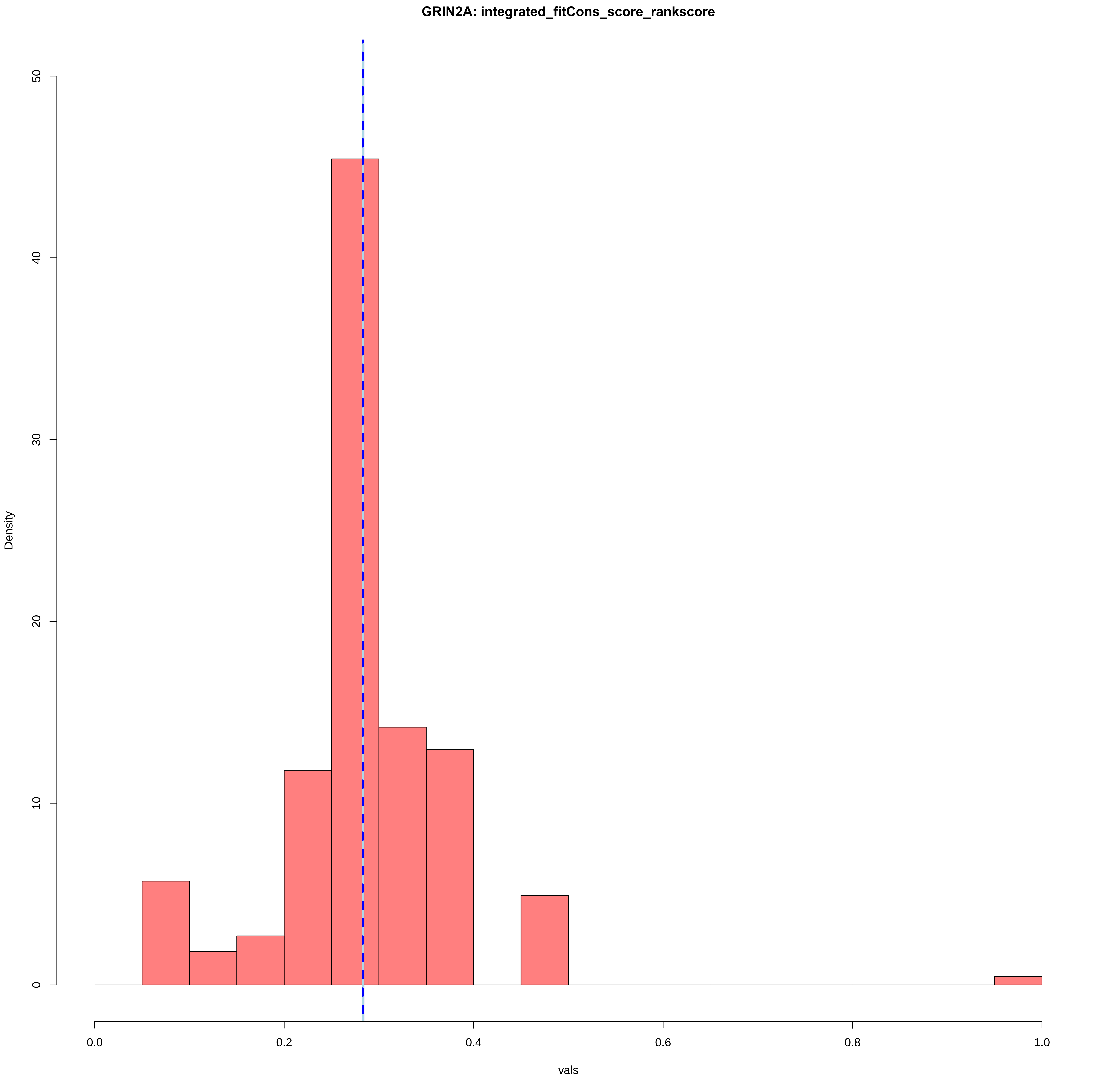
GRIN2A: integrated_fitCons_score_rankscore
Density
vals
0
10
20
30
40
50
0.0
0.2
0.4
0.6
0.8
1.0

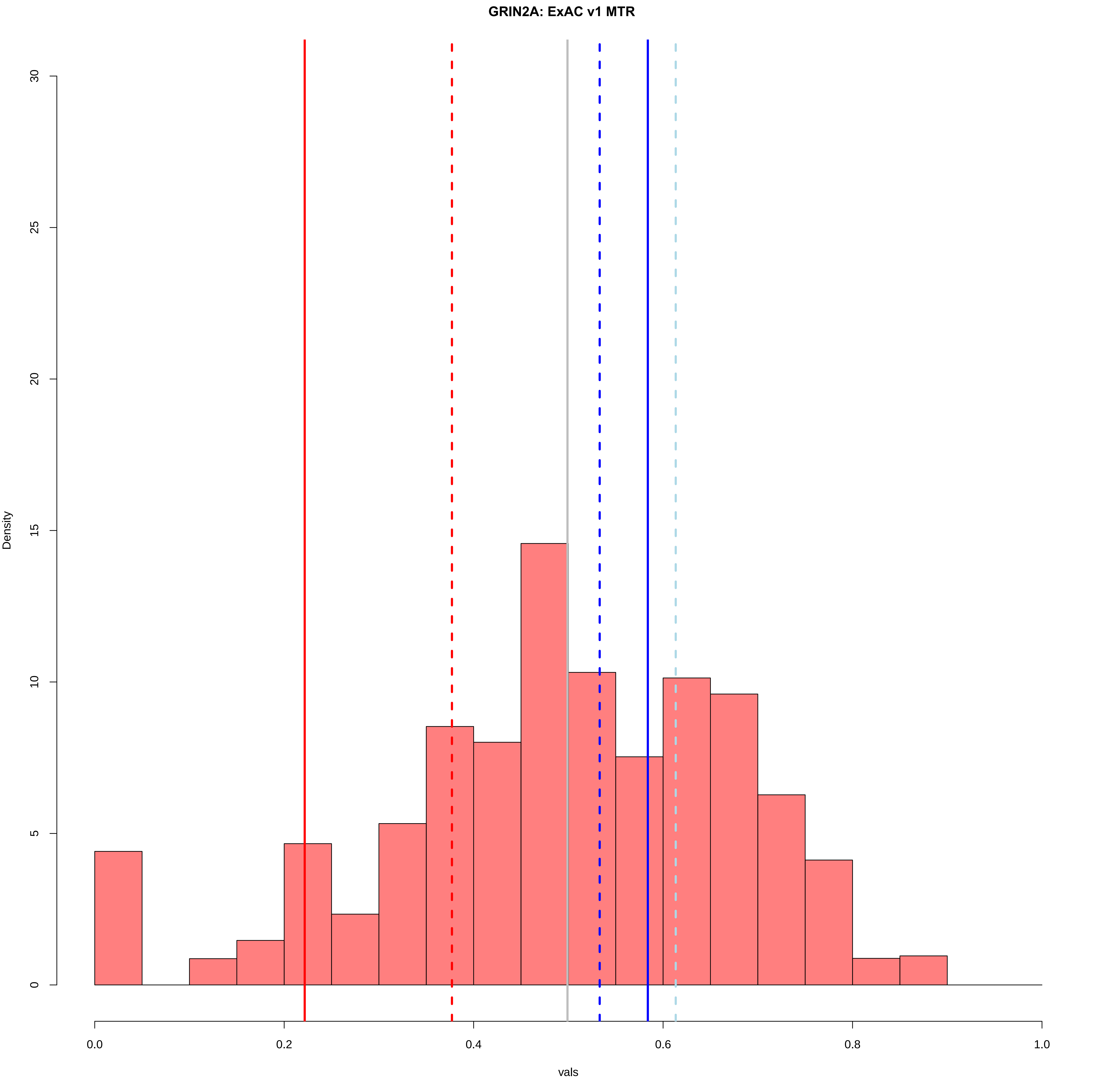
GRIN2A: ExAC v1 MTR
Density
vals
0
5
10
15
20
25
30
0.0
0.2
0.4
0.6
0.8
1.0

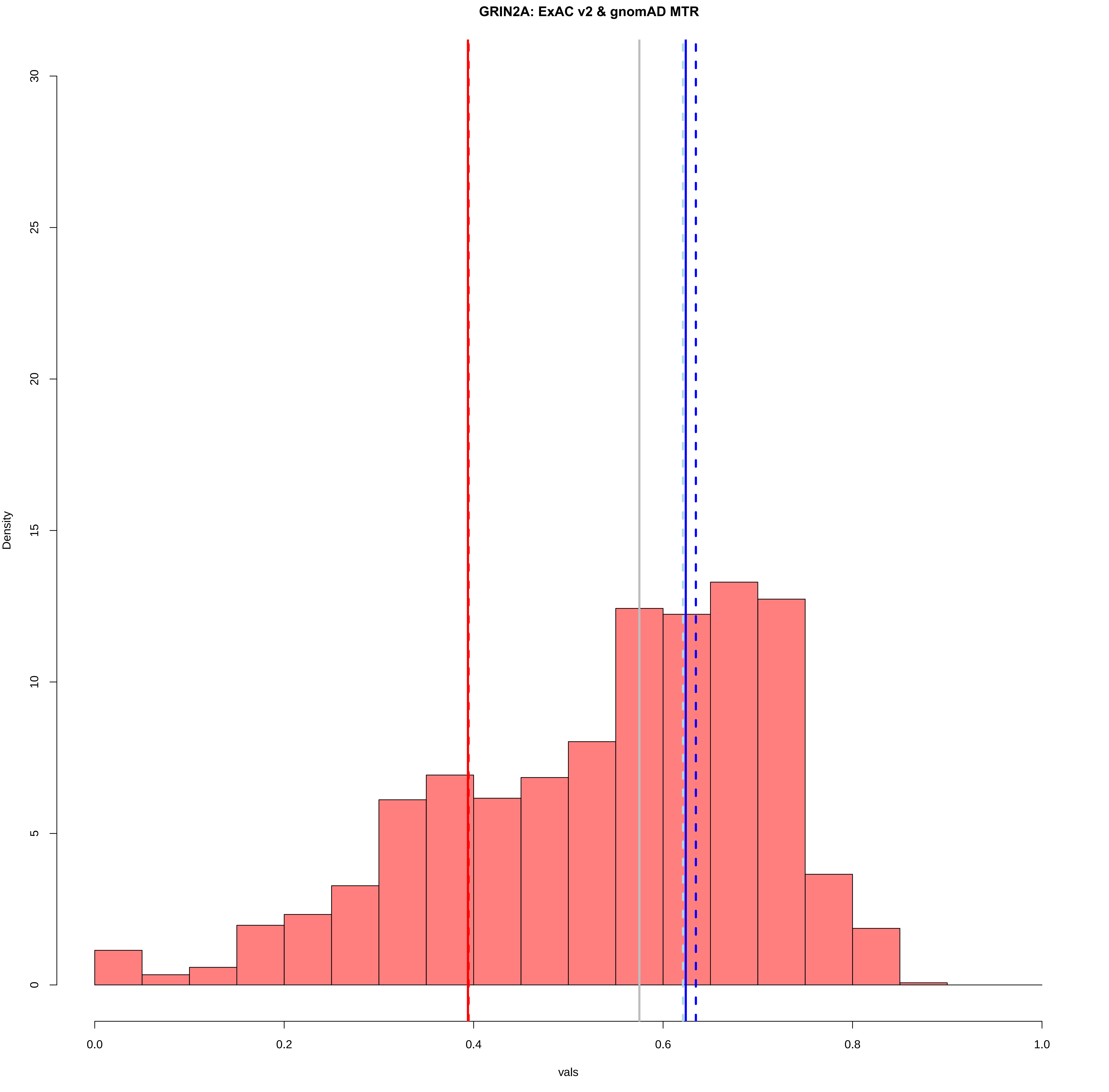
GRIN2A: ExAC v2 & gnomAD MTR
Density
vals
0
5
10
15
20
25
30
0.0
0.2
0.4
0.6
0.8
1.0

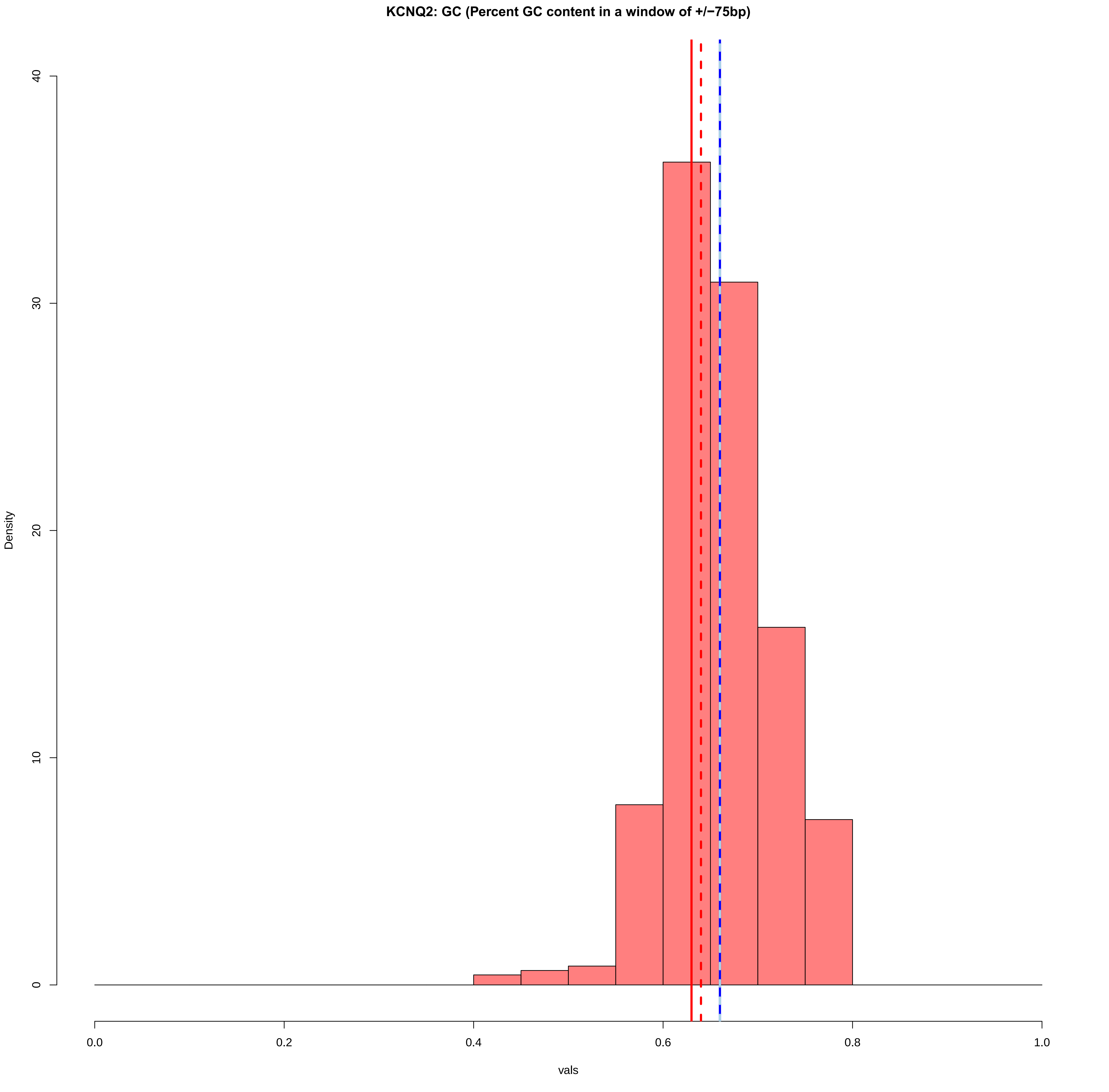
KCNQ2: GC (Percent GC content in a window of +/−75bp)
Density
vals
0
10
20
30
40
0.0
0.2
0.4
0.6
0.8
1.0

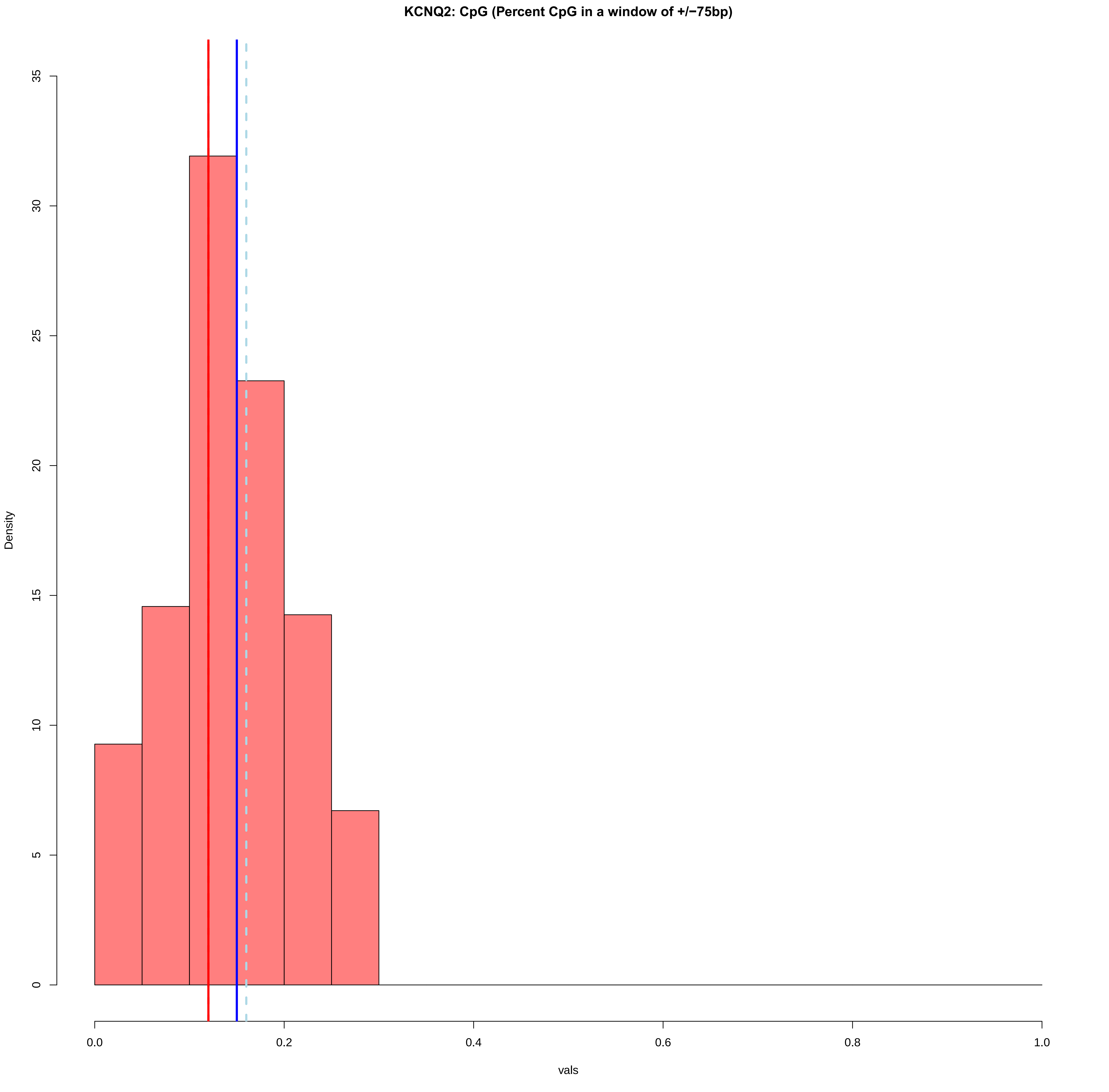
KCNQ2: CpG (Percent CpG in a window of +/−75bp)
Density
vals
0
5
10
15
20
25
30
35
0.0
0.2
0.4
0.6
0.8
1.0

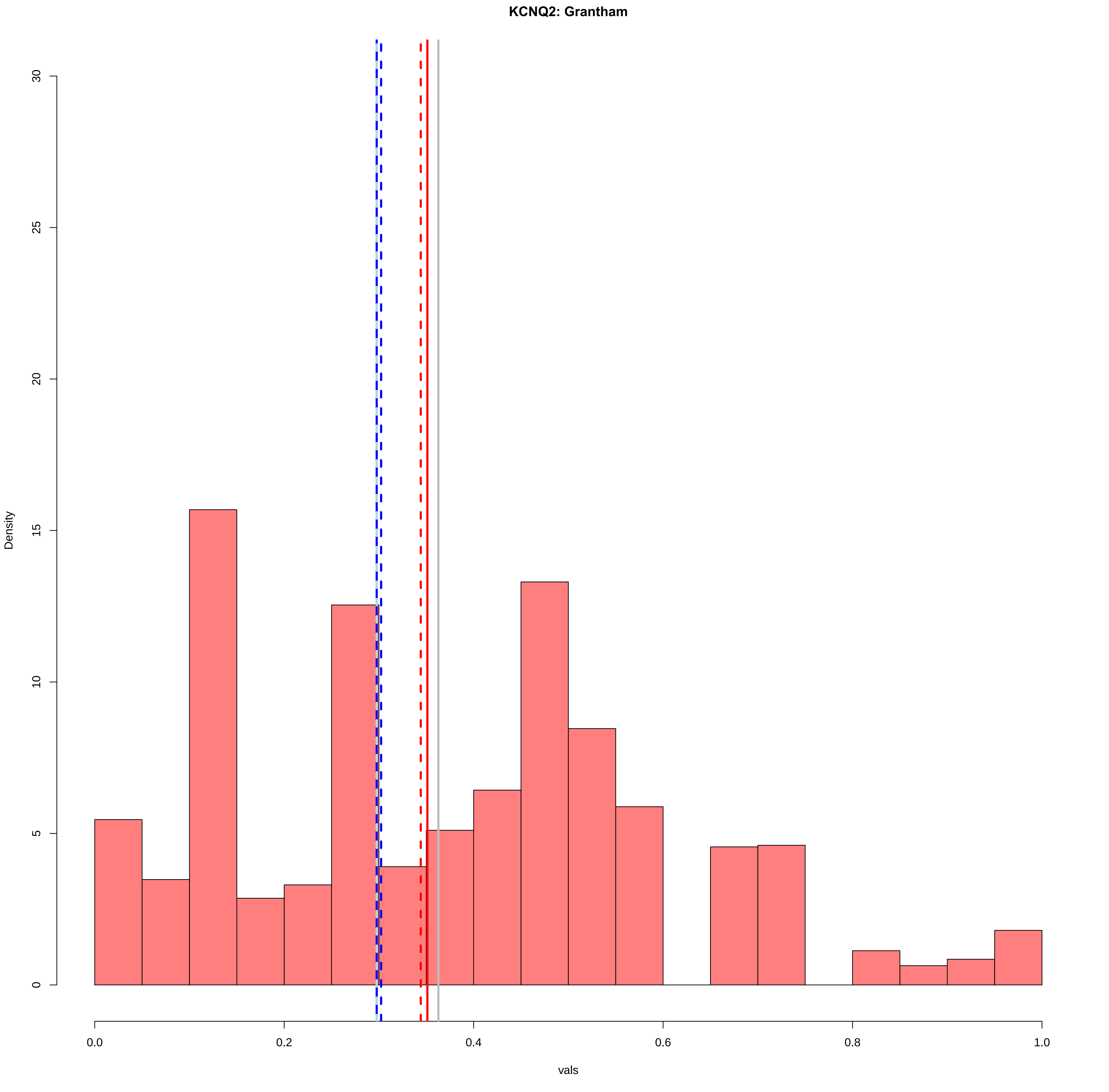
KCNQ2: Grantham
Density
vals
0
5
10
15
20
25
30
0.0
0.2
0.4
0.6
0.8
1.0

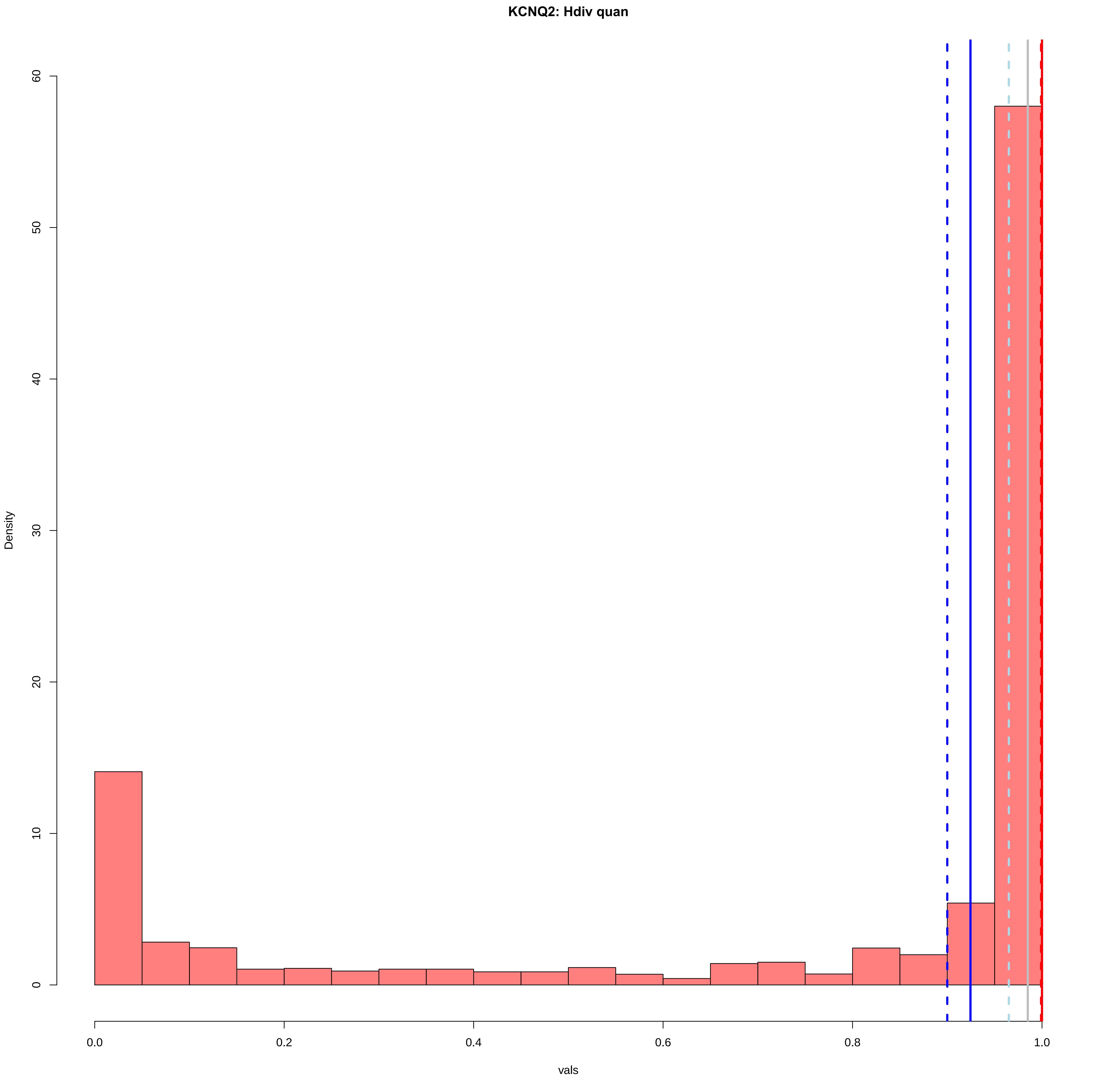
KCNQ2: Hdiv quan
Density
vals
0
10
20
30
40
50
60
0.0
0.2
0.4
0.6
0.8
1.0

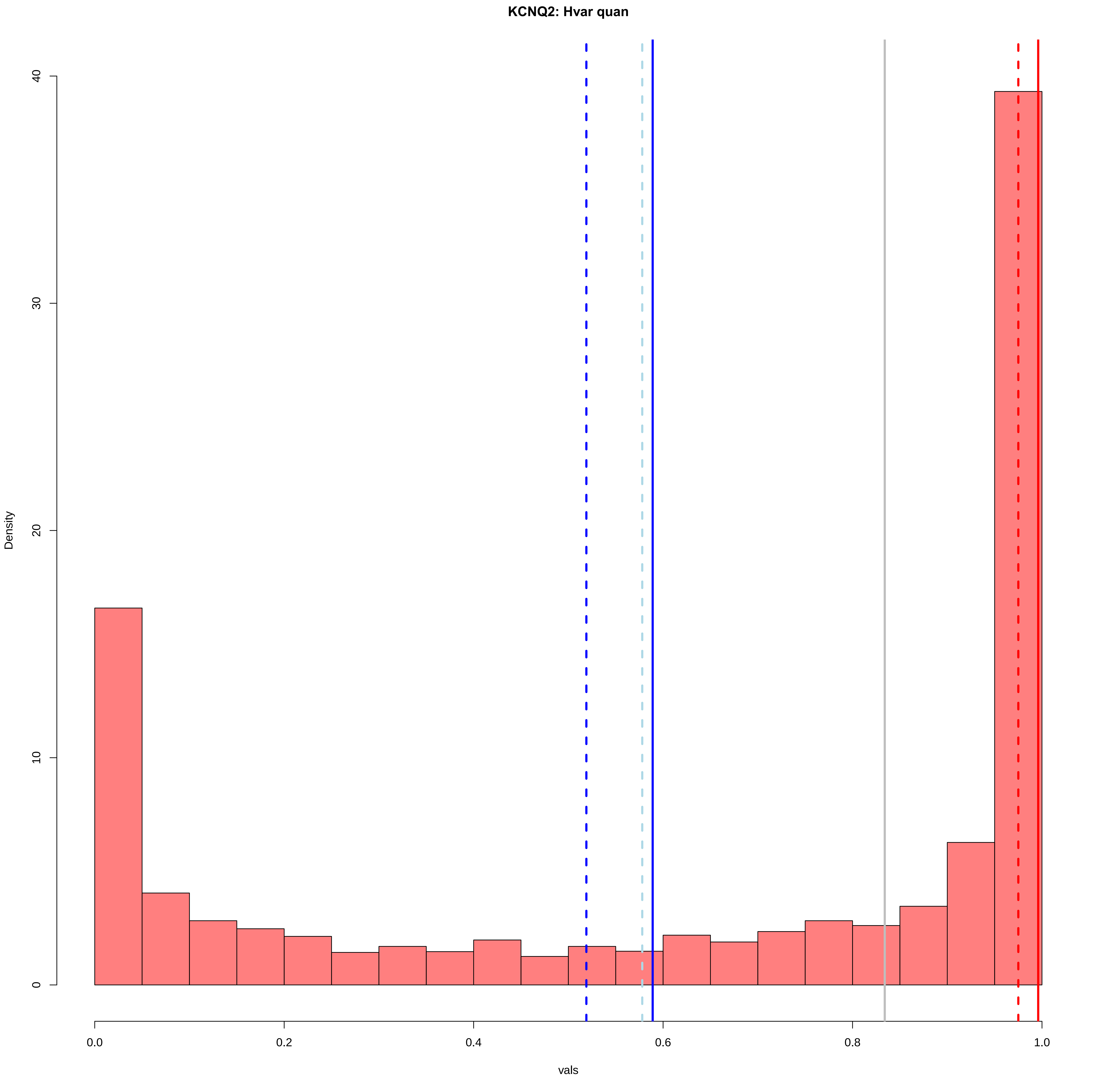
KCNQ2: Hvar quan
Density
40
30
20
10
0
0.0
0.2
0.4
0.6
0.8
1.0
vals

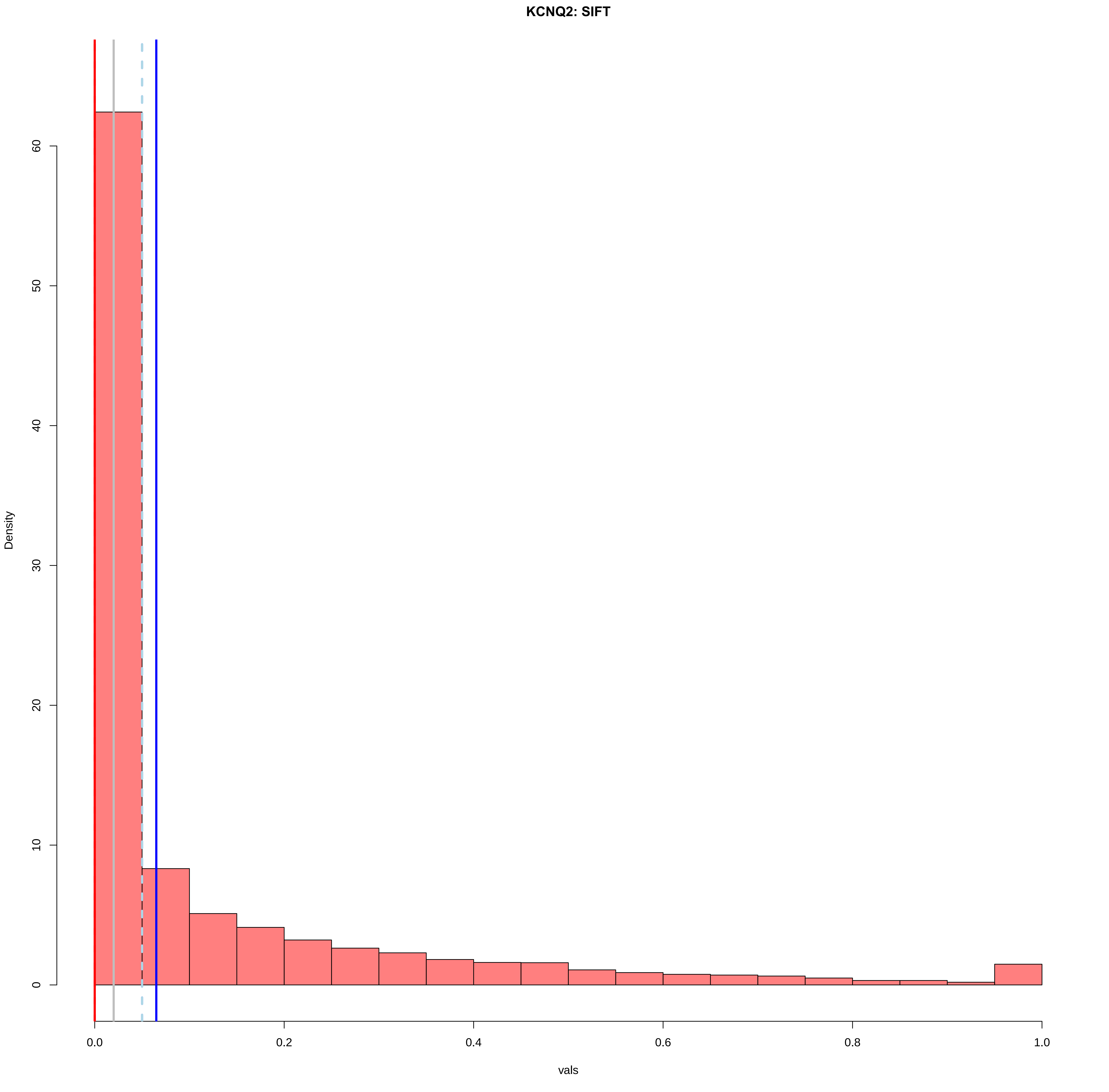
KCNQ2: SIFT
Density
vals
0
10
20
30
40
50
60
0.0
0.2
0.4
0.6
0.8
1.0

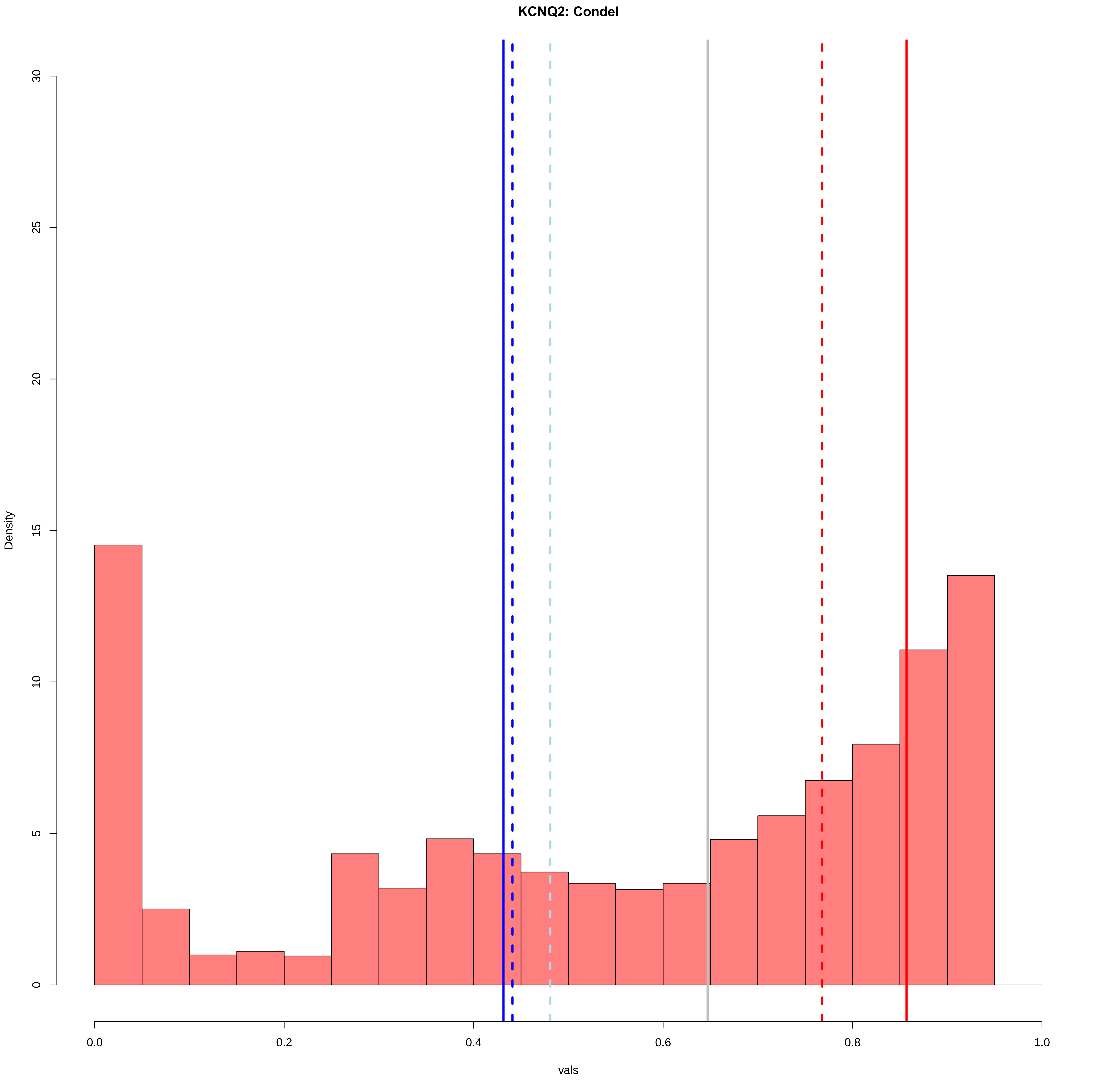
KCNQ2: Condel
Density
vals
0
5
10
15
20
25
30
0.0
0.2
0.4
0.6
0.8
1.0

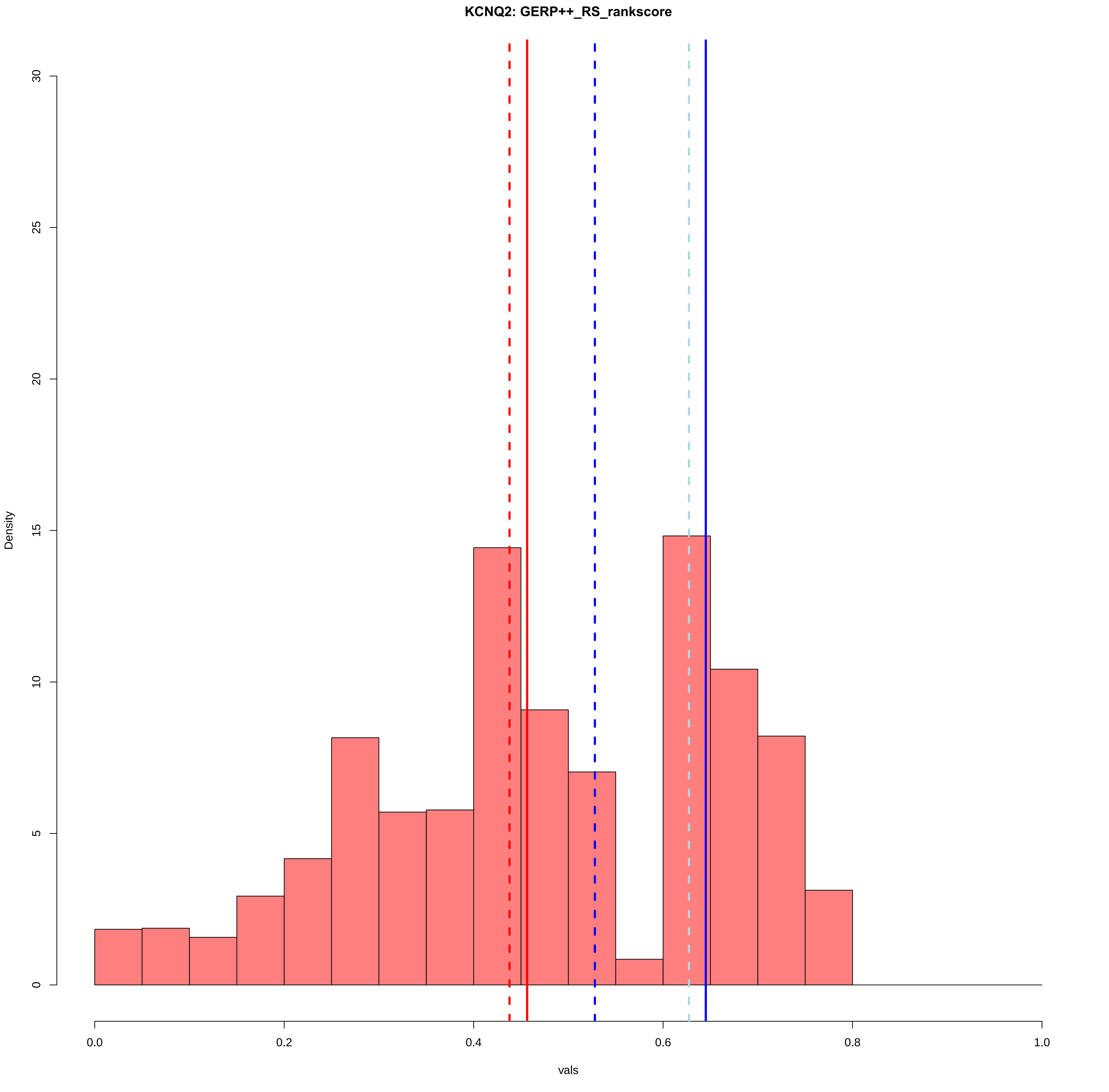
KCNQ2: GERP++_RS_rankscore
Density
vals
0
5
10
15
20
25
30
0.0
0.2
0.4
0.6
0.8
1.0

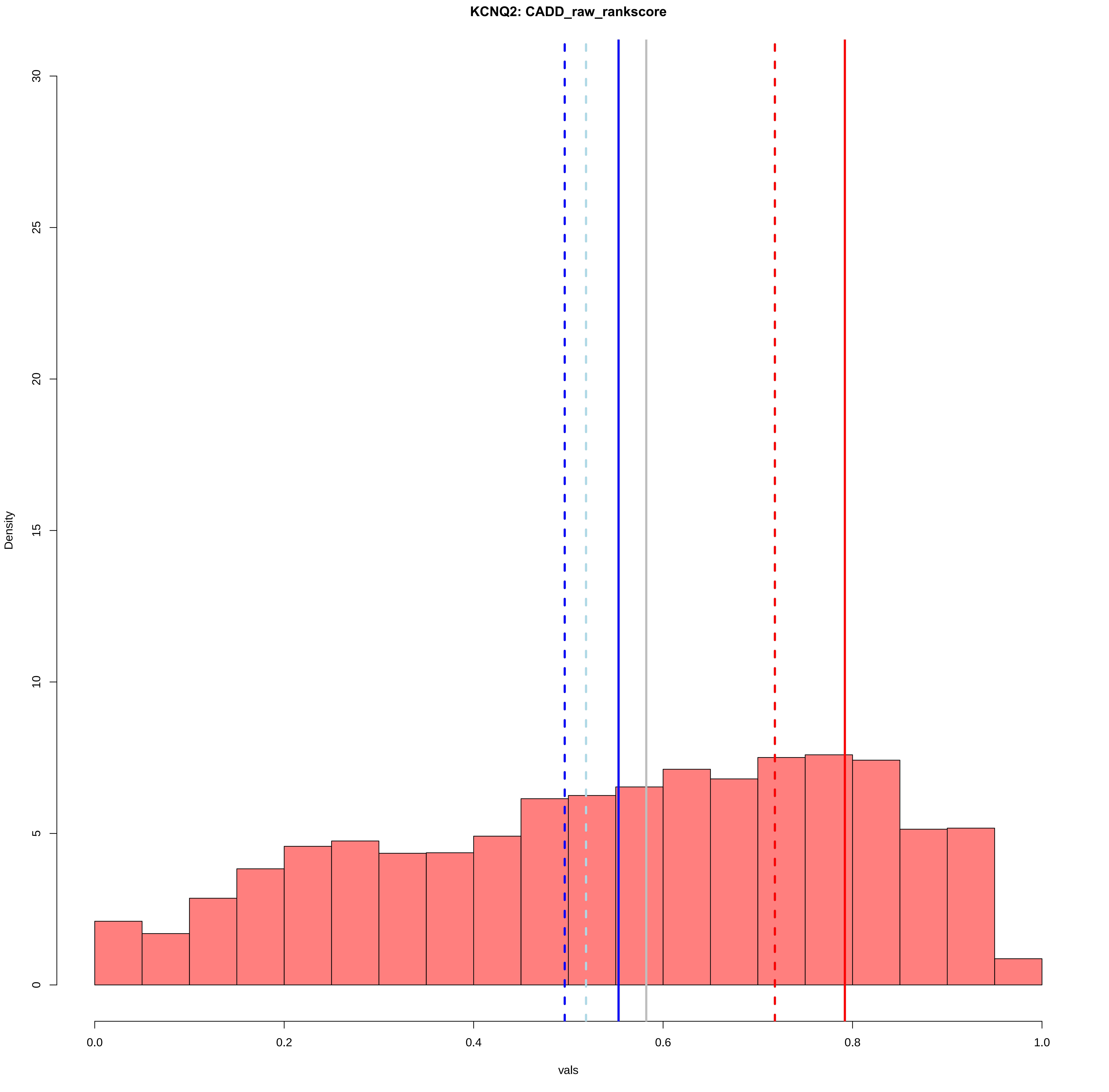

KCNQ2: CADD_raw_rankscore
Density
vals
0
5
10
15
20
25
30
0.0
0.2
0.4
0.6
0.8
1.0

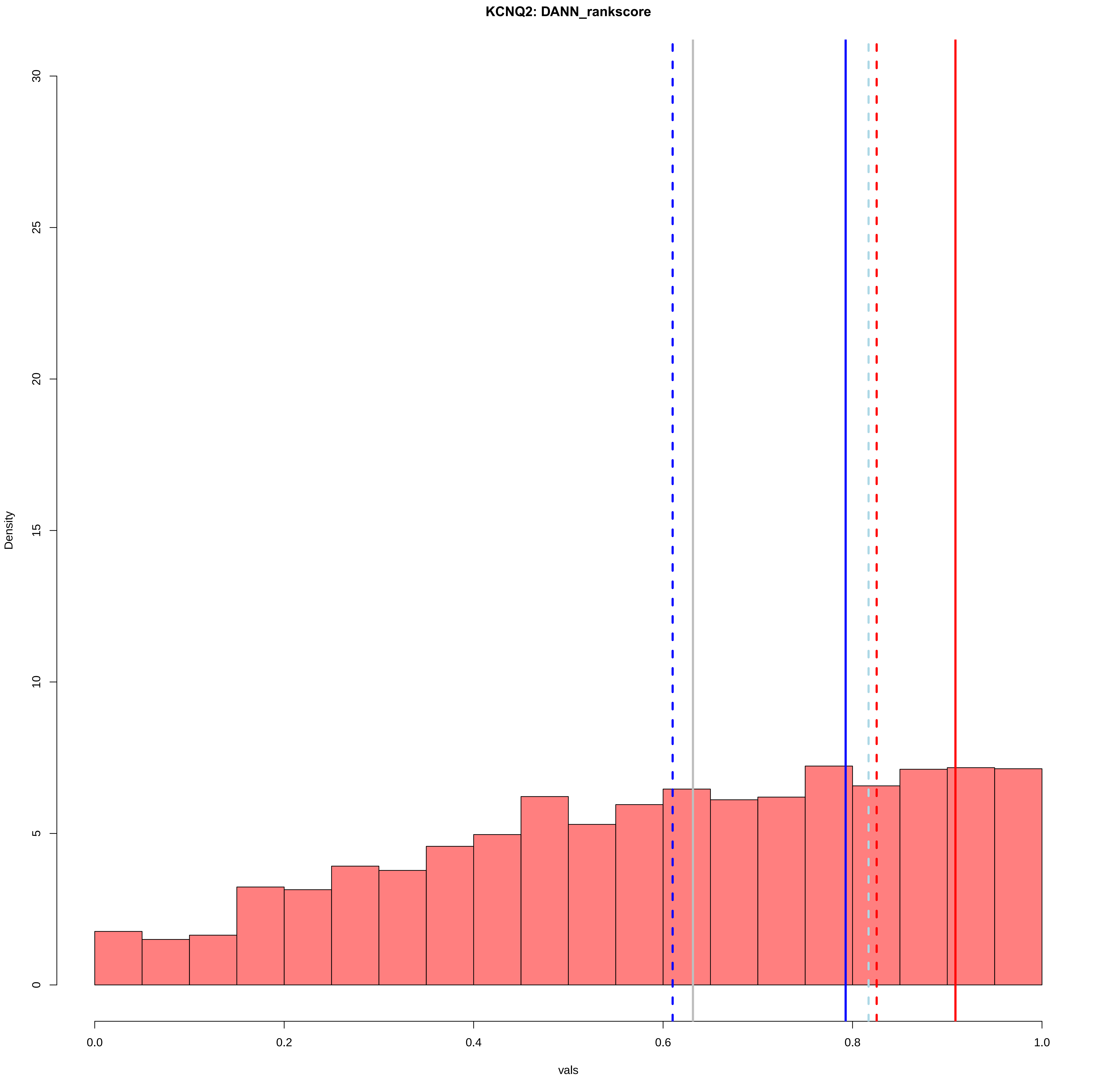

KCNQ2: DANN_rankscore
Density
vals
0
5
10
15
20
25
30
0.0
0.2
0.4
0.6
0.8
1.0

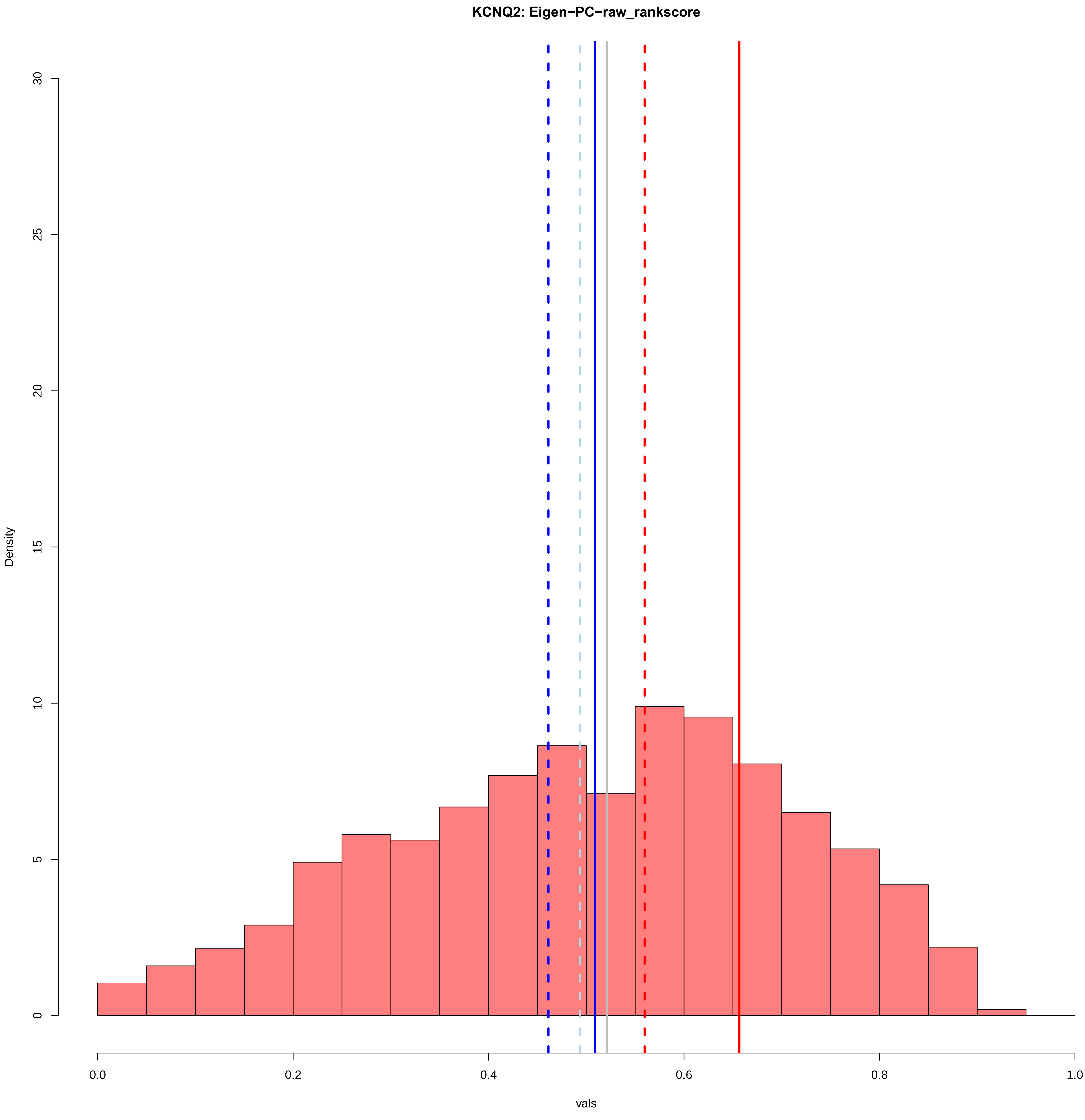

KCNQ2: Eigen−PC−raw_rankscore
Density
vals
0
5
10
15
20
25
30
0.0
0.2
0.4
0.6
0.8
1.0

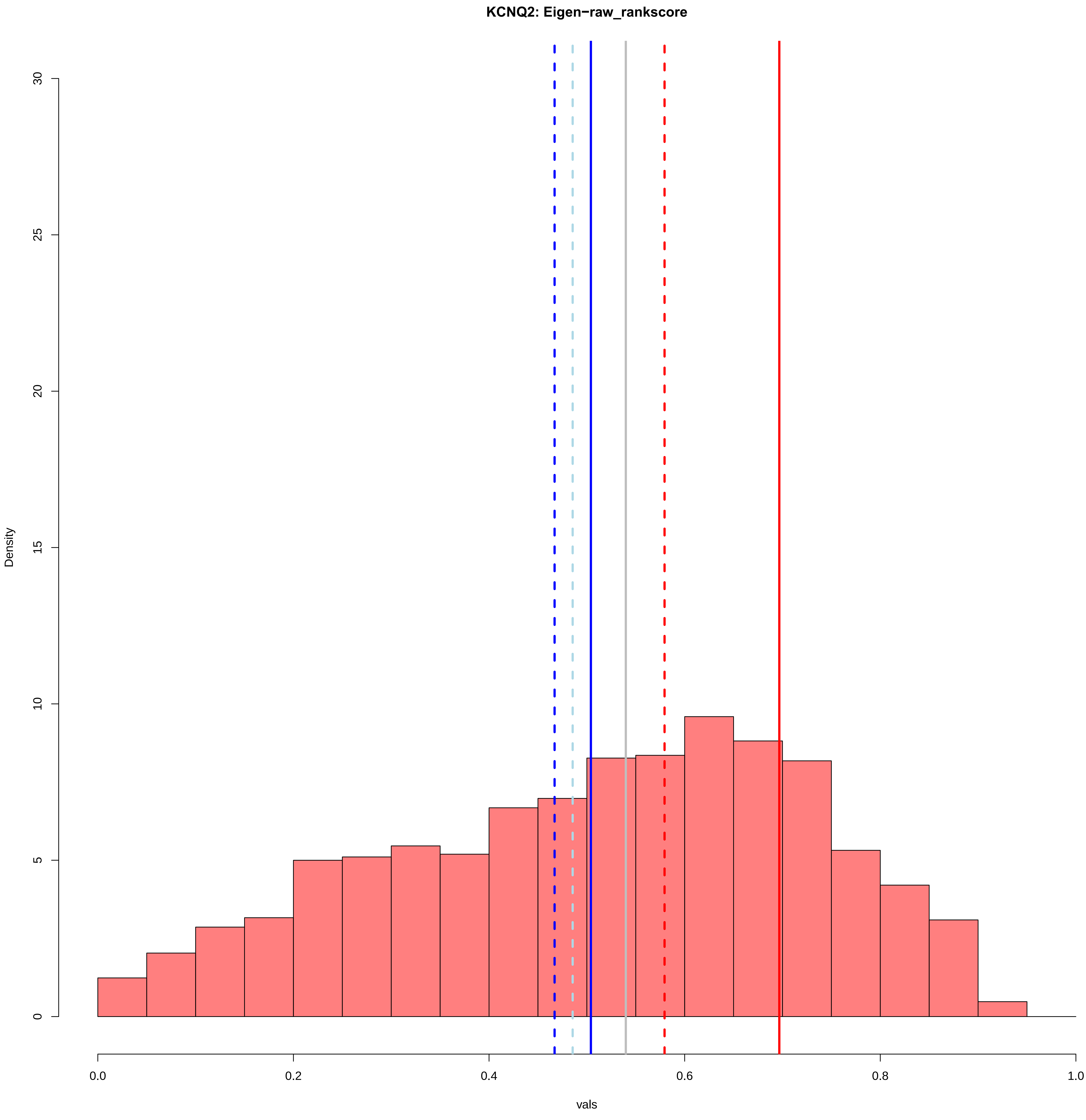
KCNQ2: Eigen−raw_rankscore
Density
vals
0
5
10
15
20
25
30
0.0
0.2
0.4
0.6
0.8
1.0

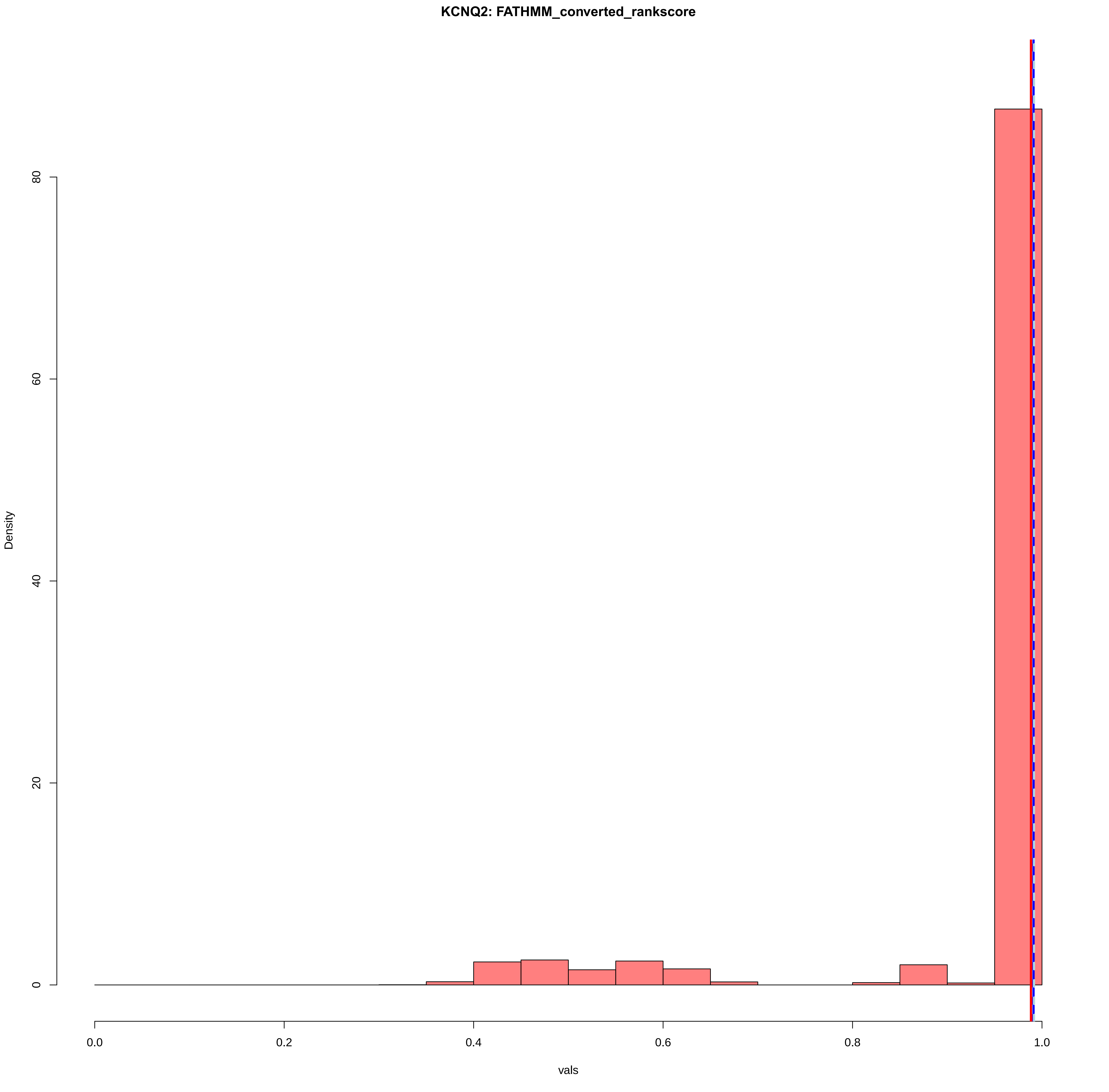
KCNQ2: FATHMM_converted_rankscore
Density
vals
0
20
40
60
80
0.0
0.2
0.4
0.6
0.8
1.0

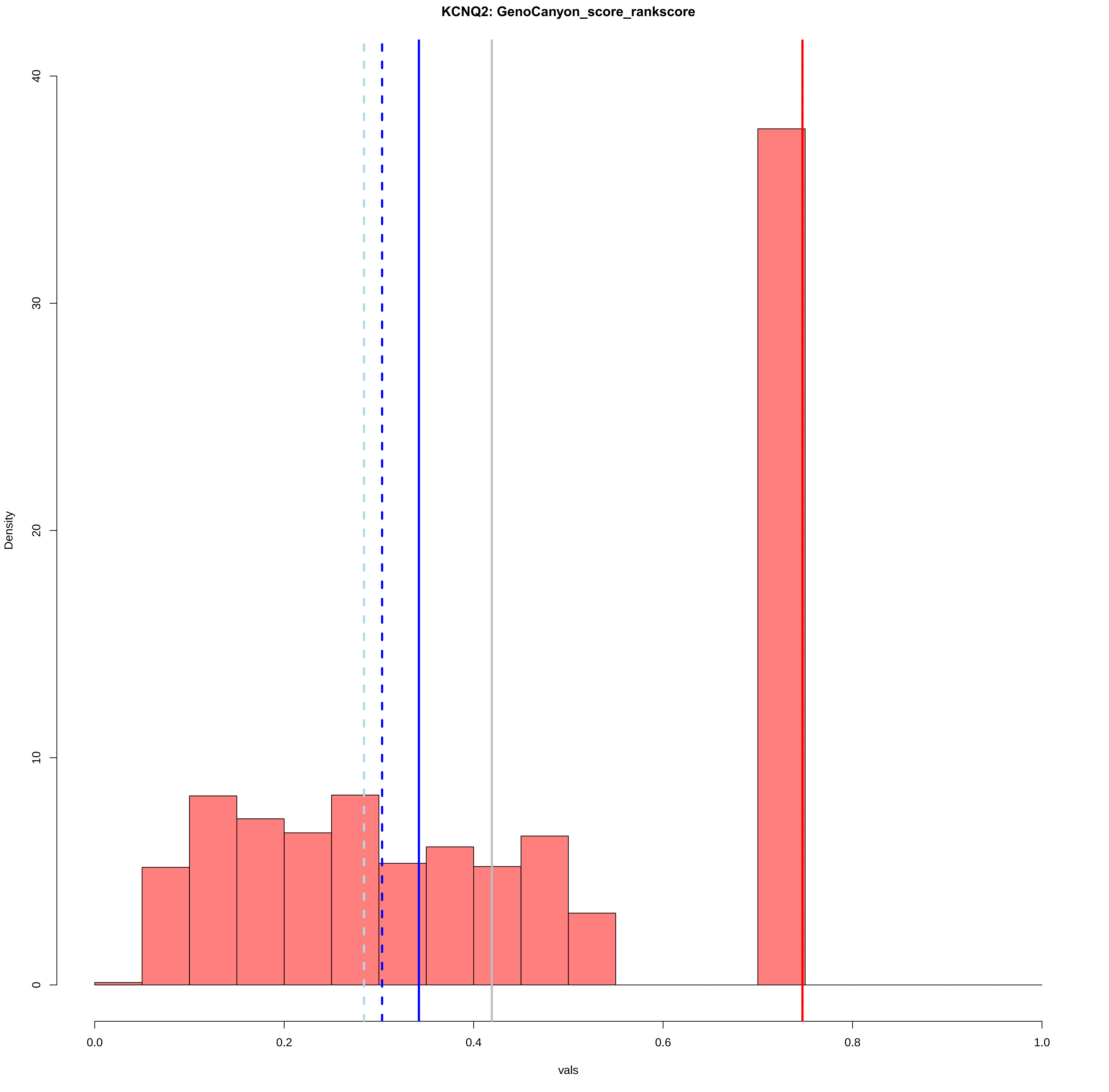
KCNQ2: GenoCanyon_score_rankscore
Density
vals
0
10
20
30
40
0.0
0.2
0.4
0.6
0.8
1.0

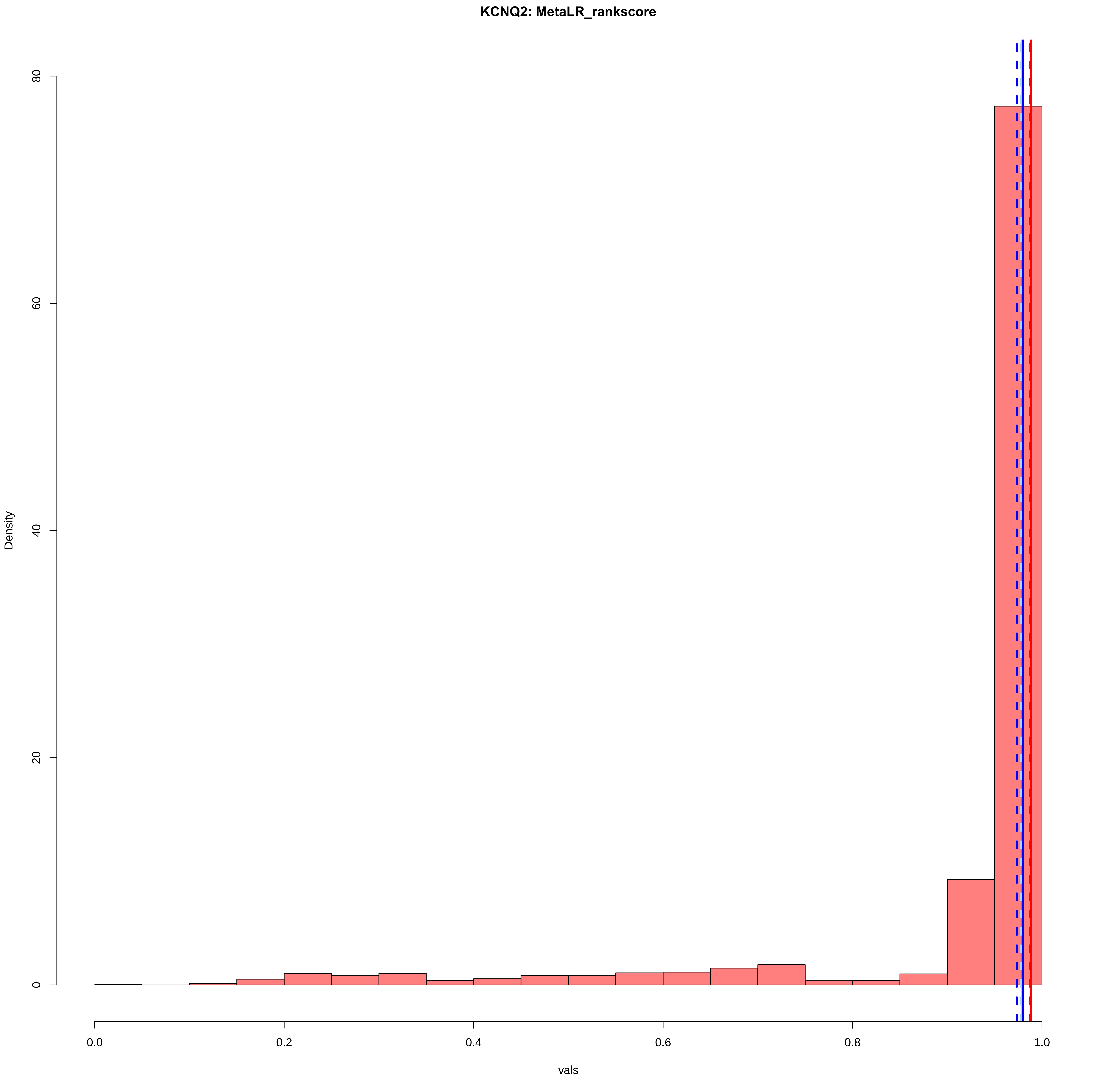
KCNQ2: MetaLR_rankscore
Density
0
20
40
60
80
0.0
0.2
0.4
0.6
0.8
1.0
vals

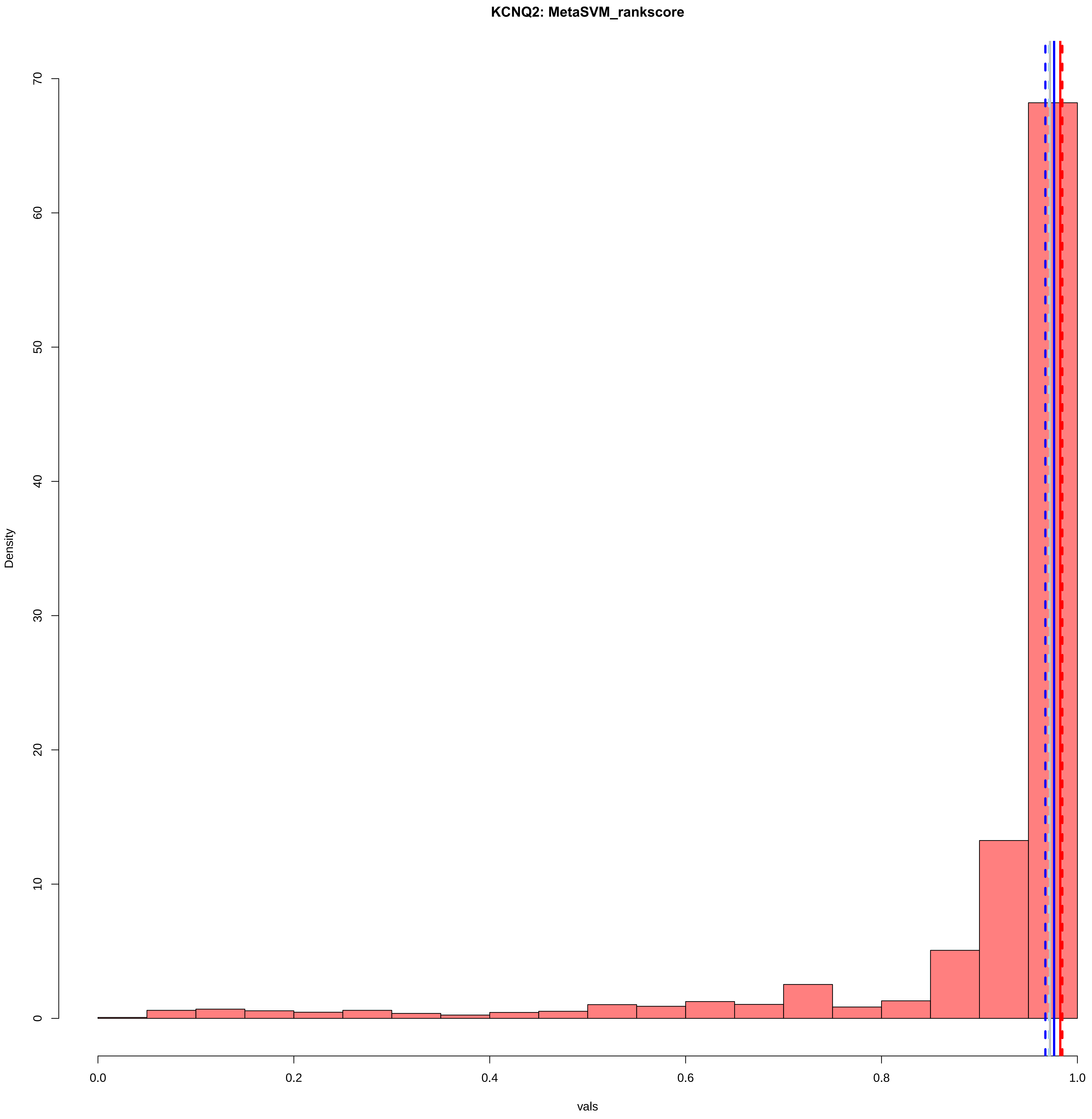

KCNQ2: MetaSVM_rankscore
Density
vals
0
10
20
30
40
50
60
70
0.0
0.2
0.4
0.6
0.8
1.0

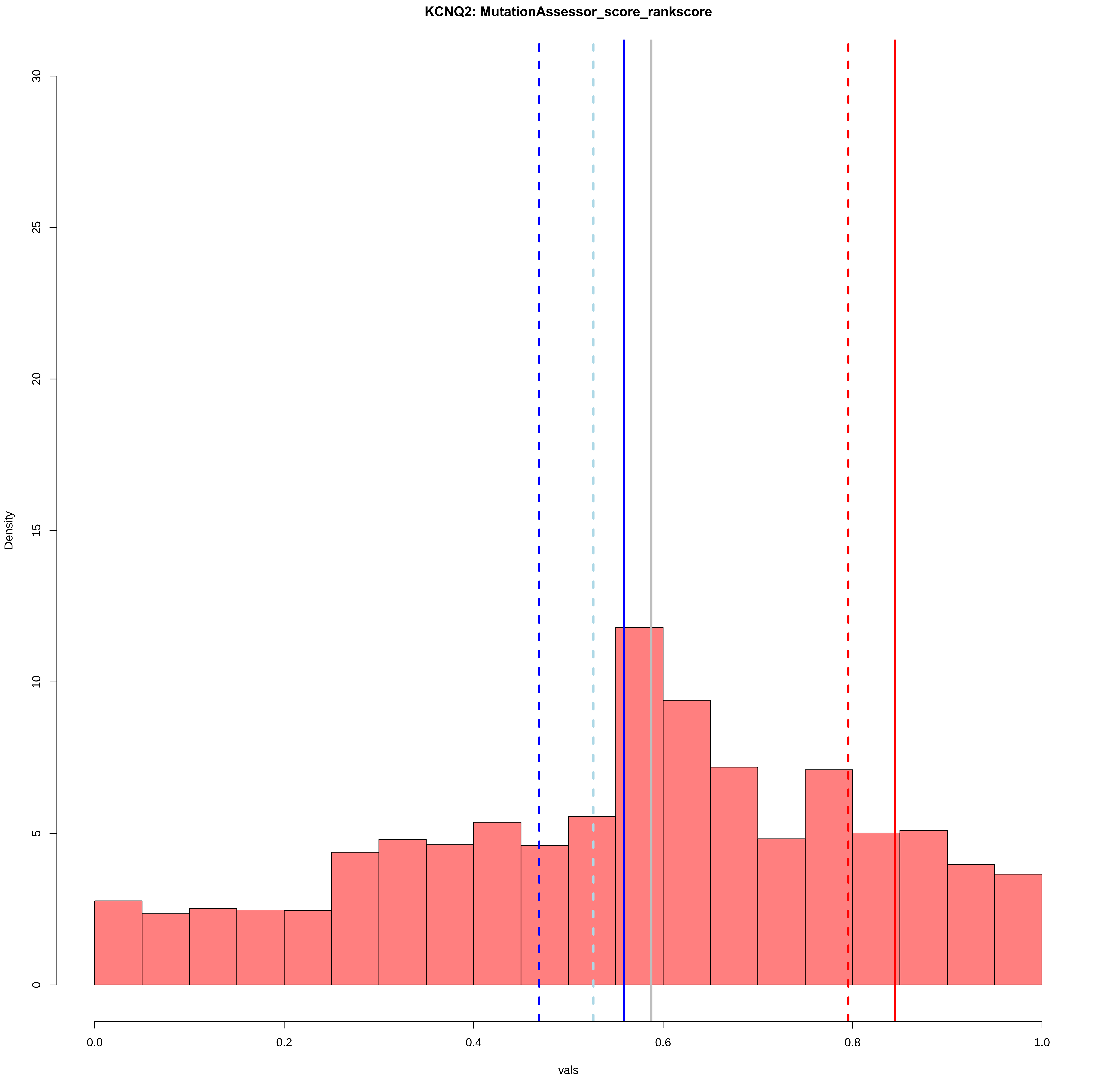

KCNQ2: MutationAssessor_score_rankscore
Density
vals
0
5
10
15
20
25
30
0.0
0.2
0.4
0.6
0.8
1.0

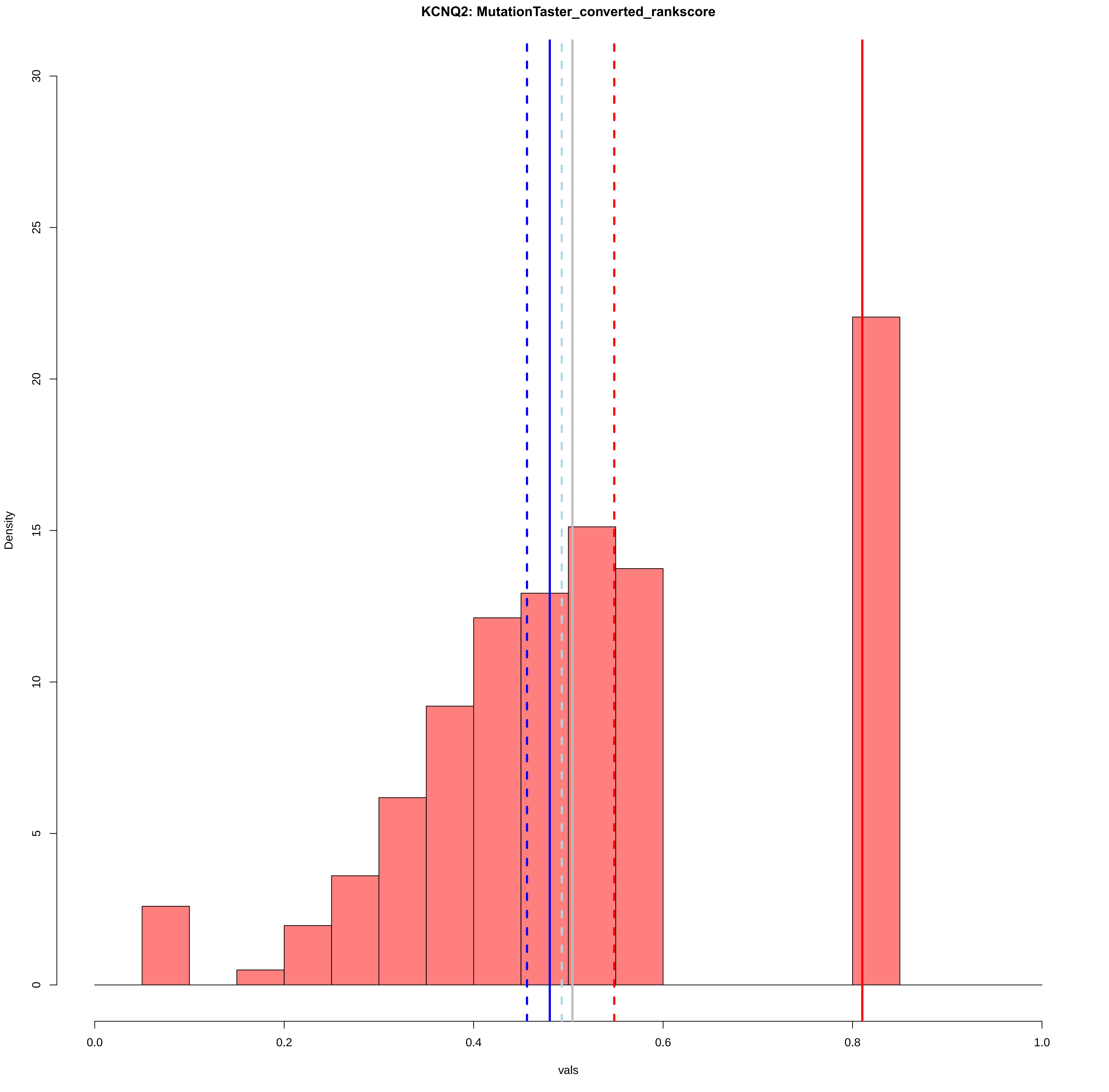
KCNQ2: MutationTaster_converted_rankscore
Density
vals
0
5
10
15
20
25
30
0.0
0.2
0.4
0.6
0.8
1.0

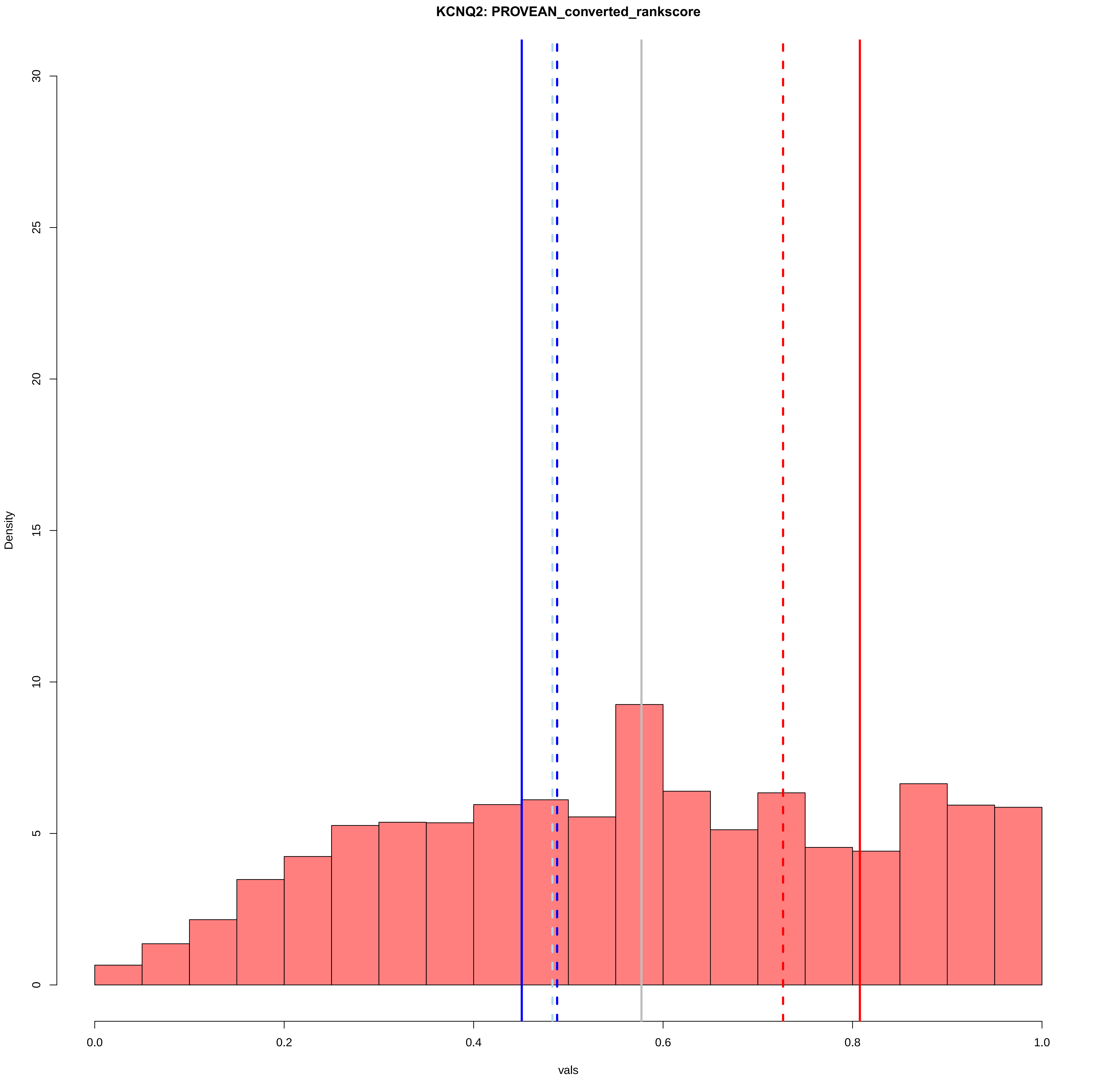
KCNQ2: PROVEAN_converted_rankscore
Density
vals
0
5
10
15
20
25
30
0.0
0.2
0.4
0.6
0.8
1.0

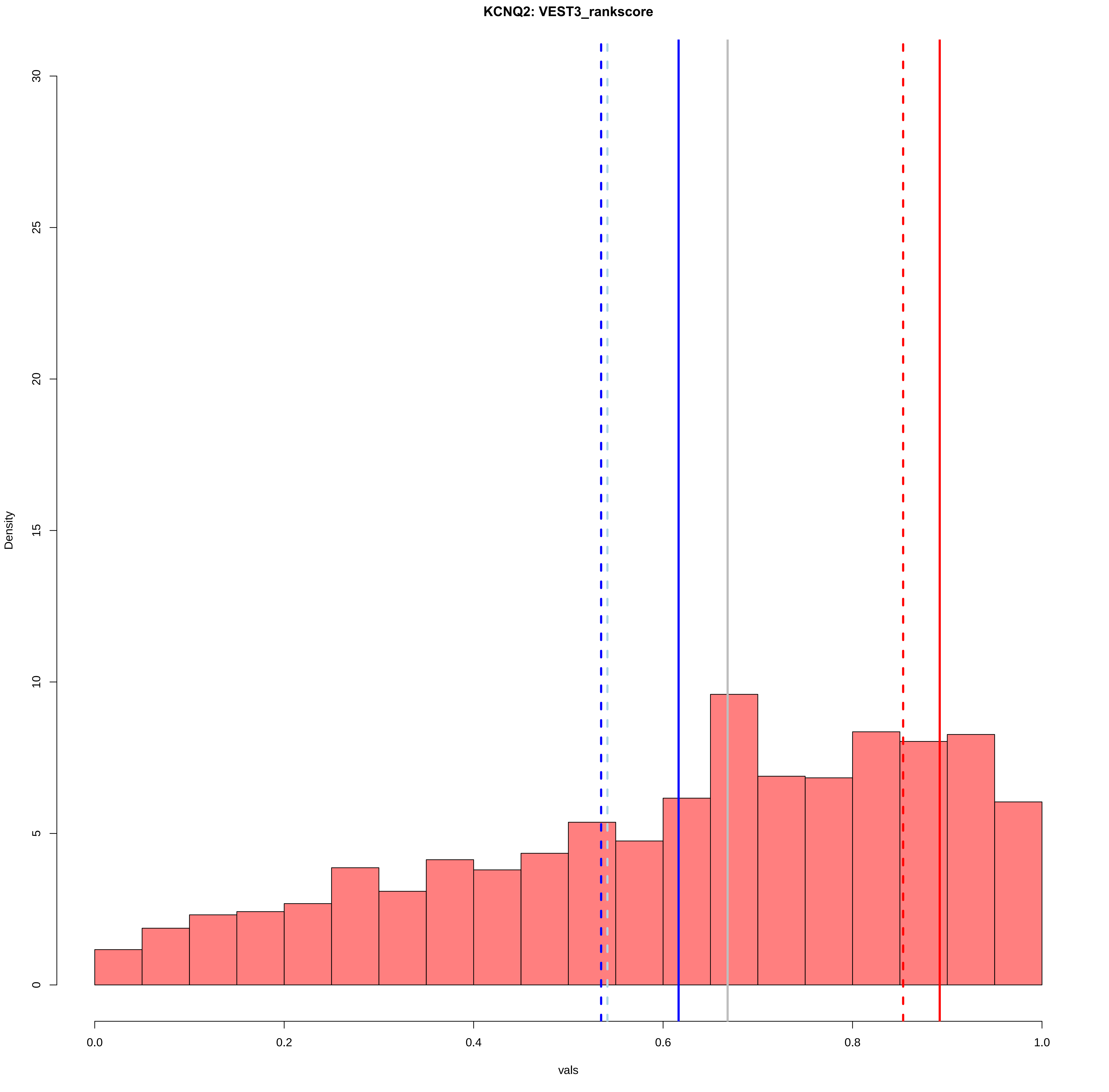

KCNQ2: VEST3_rankscore
Density
vals
0
5
10
15
20
25
30
0.0
0.2
0.4
0.6
0.8
1.0

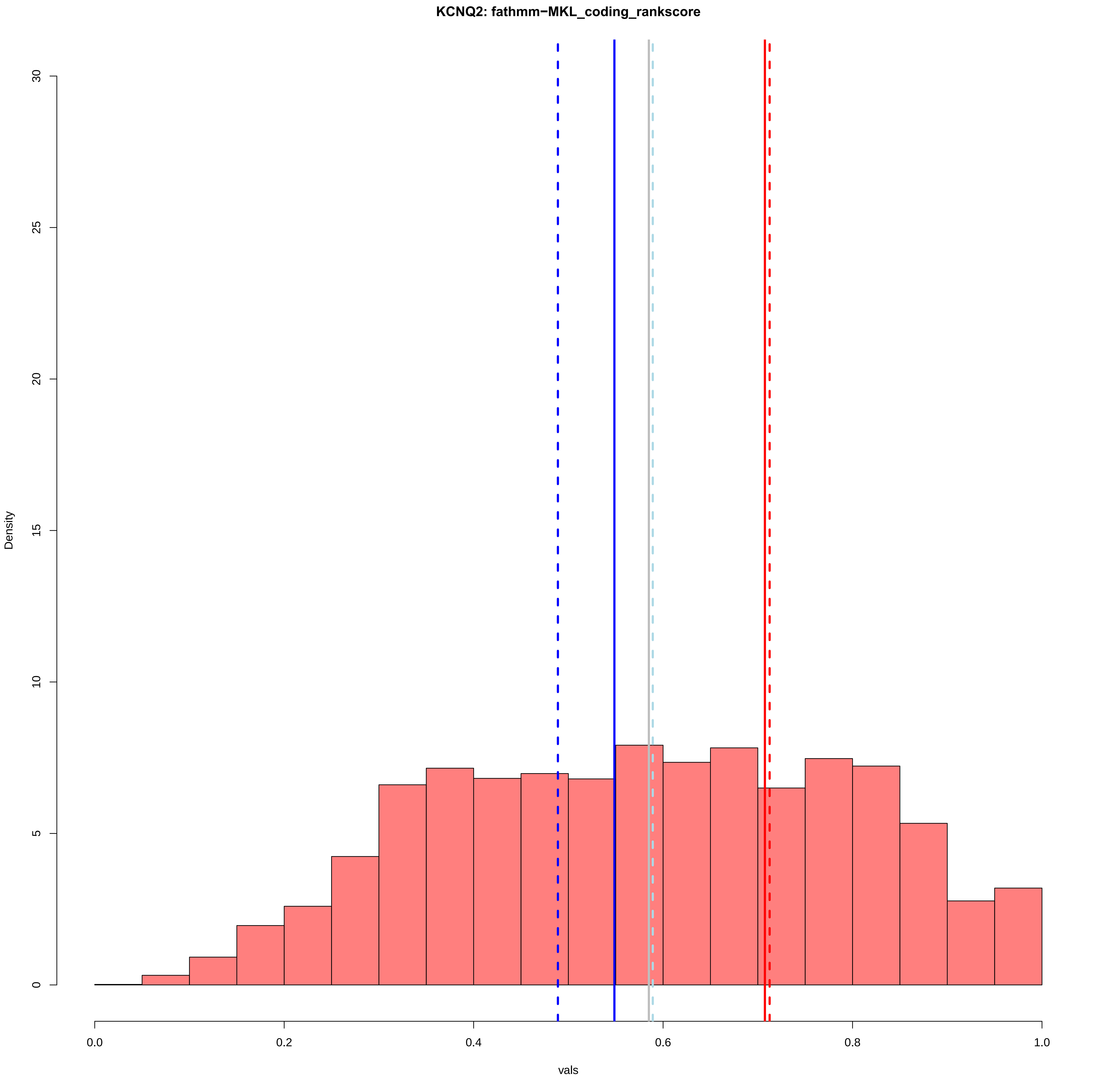

KCNQ2: fathmm−MKL_coding_rankscore
Density
vals
0
5
10
15
20
25
30
0.0
0.2
0.4
0.6
0.8
1.0

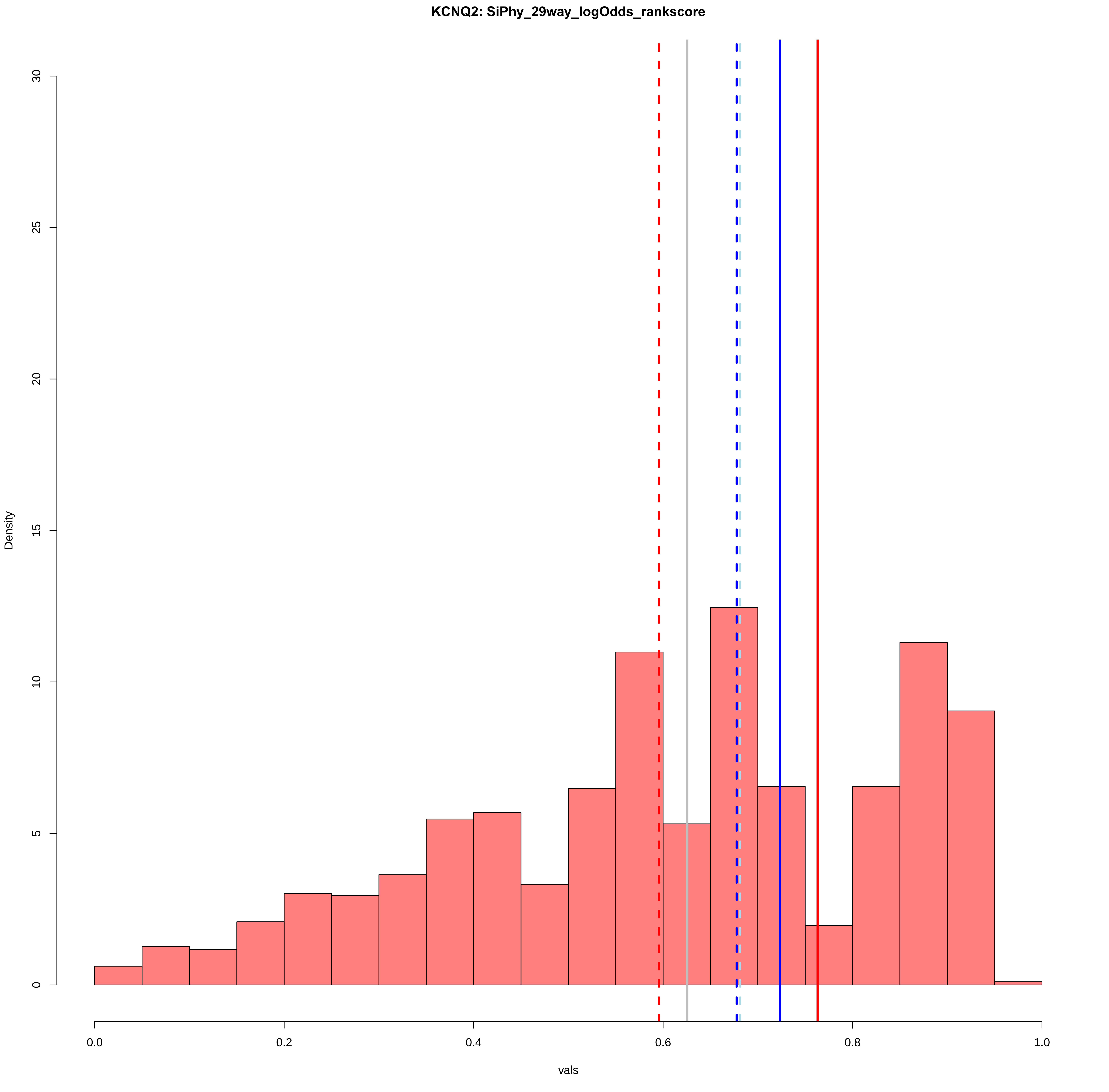

KCNQ2: SiPhy_29way_logOdds_rankscore
Density
vals
0
5
10
15
20
25
30
0.0
0.2
0.4
0.6
0.8
1.0

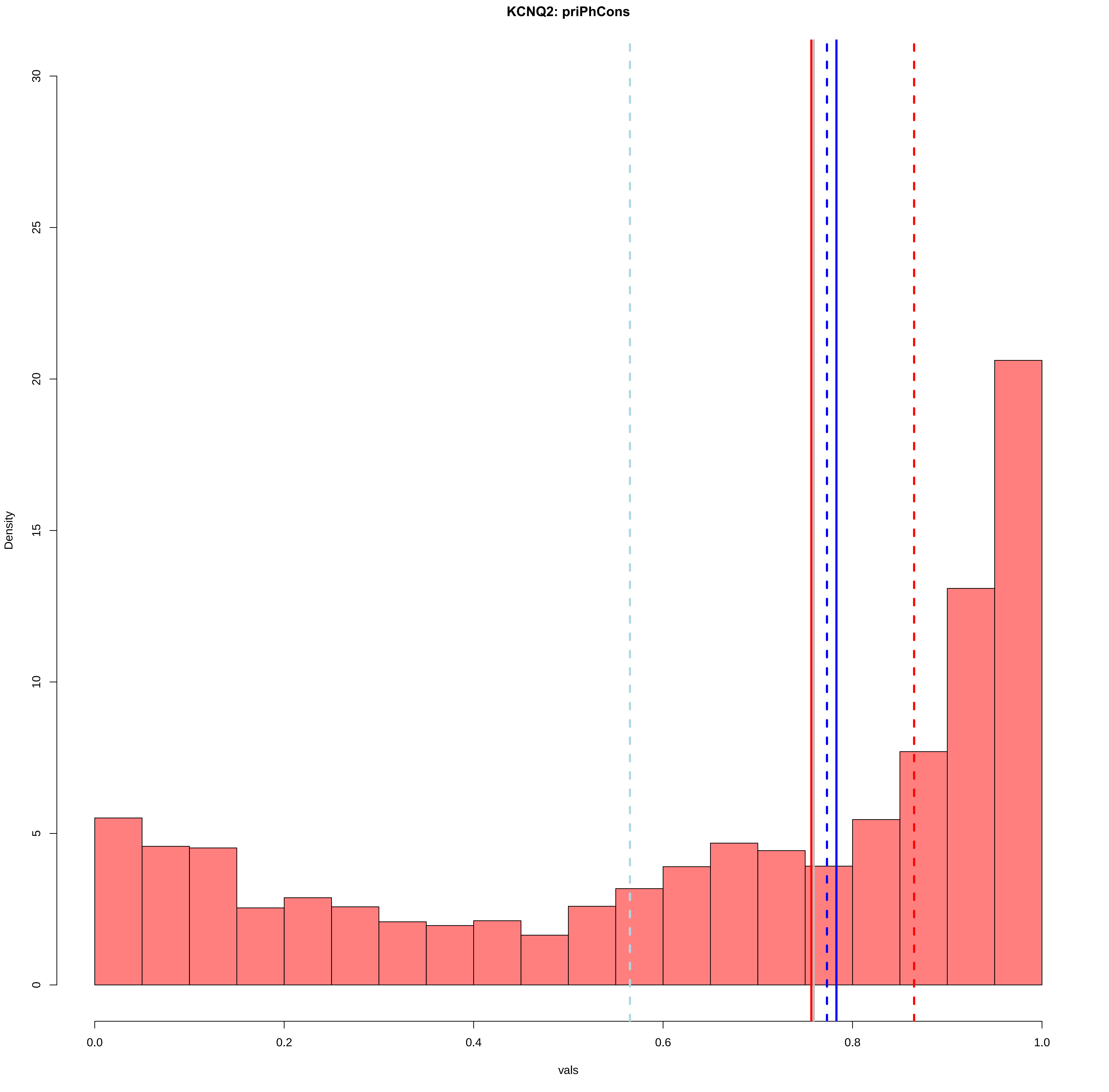
KCNQ2: priPhCons
Density
vals
0
5
10
15
20
25
30
0.0
0.2
0.4
0.6
0.8
1.0

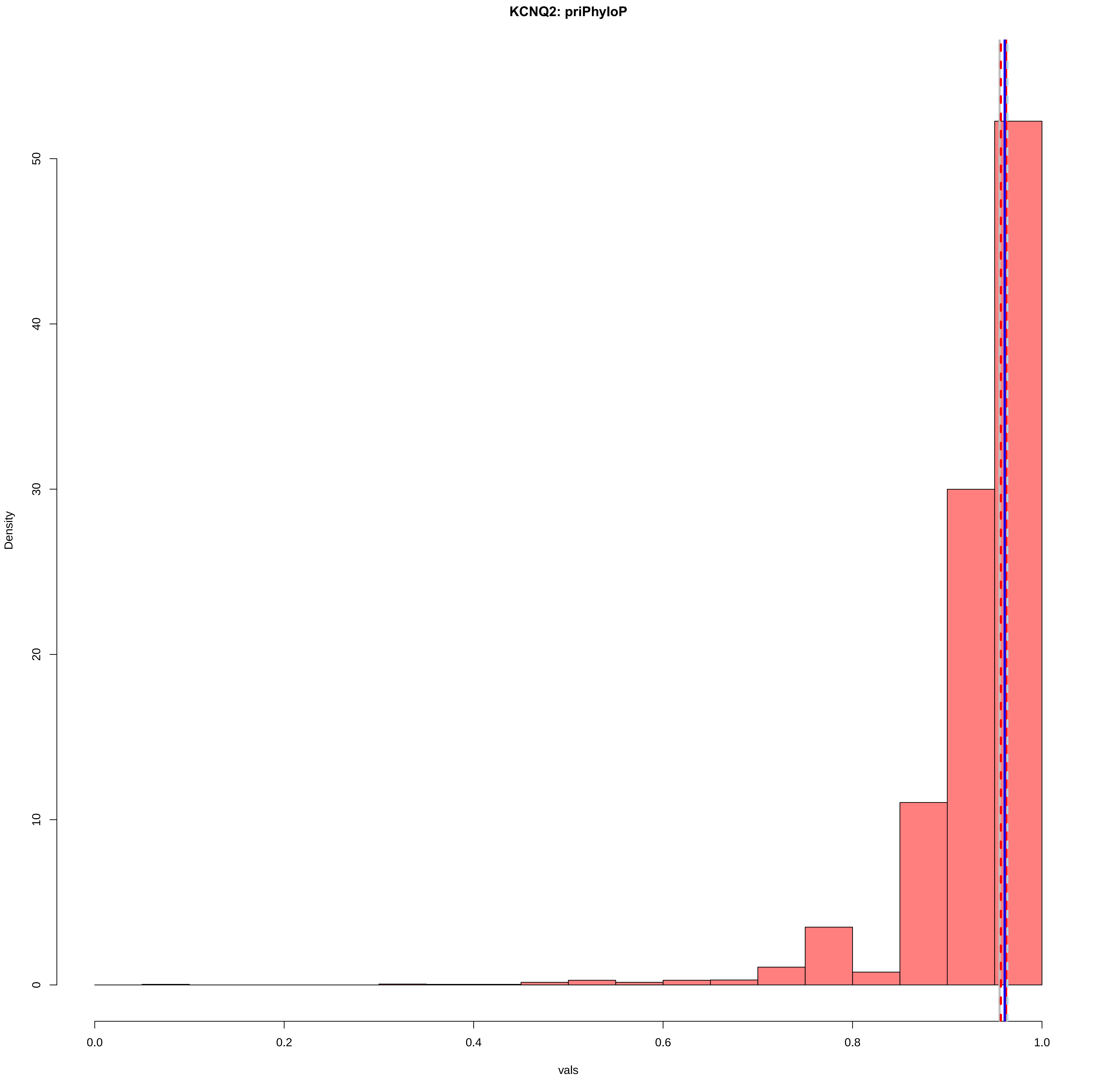
KCNQ2: priPhyloP
Density
vals
0
10
20
30
40
50
0.0
0.2
0.4
0.6
0.8
1.0

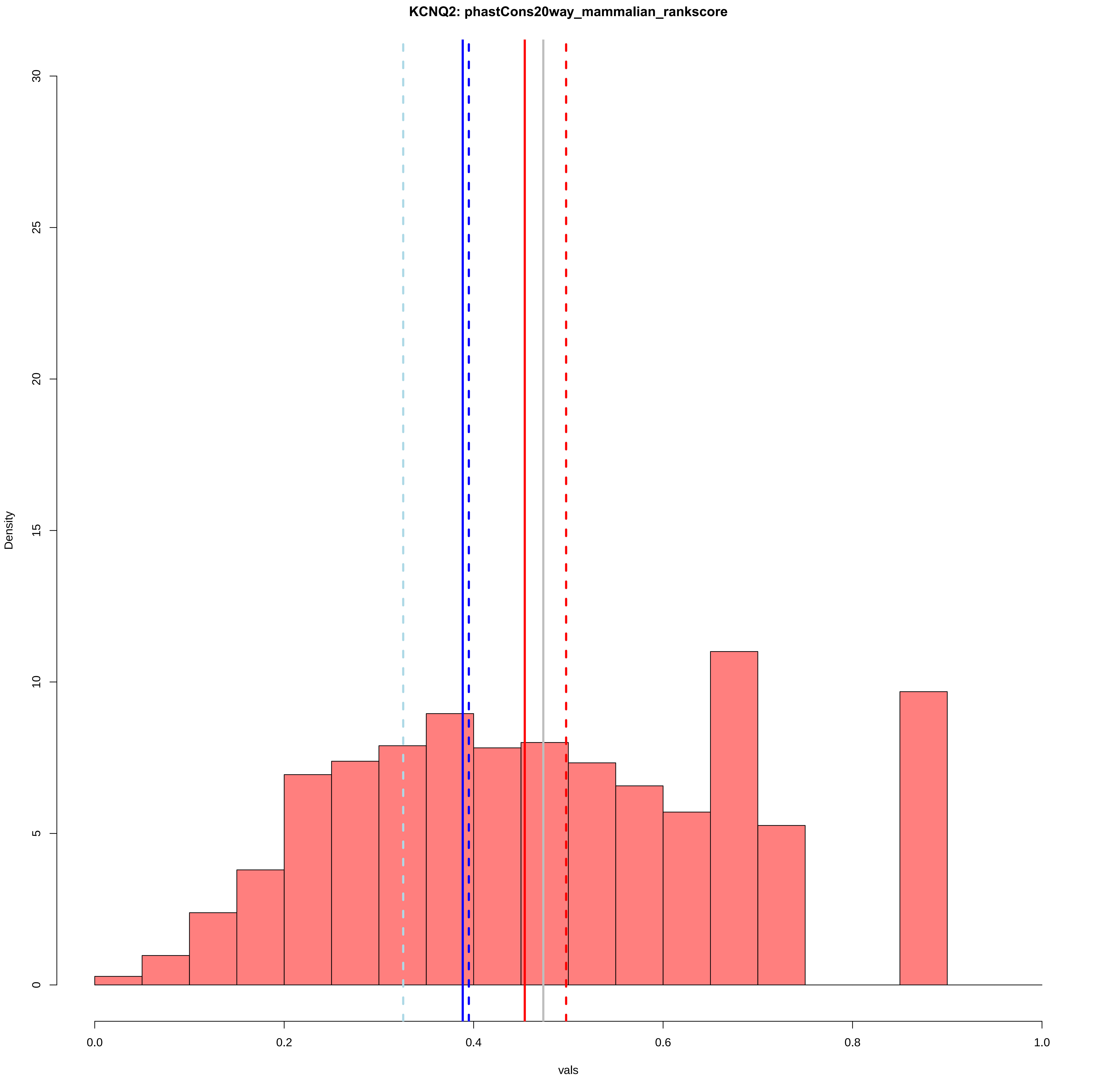
KCNQ2: phastCons20way_mammalian_rankscore
Density
vals
0
5
10
15
20
25
30
0.0
0.2
0.4
0.6
0.8
1.0

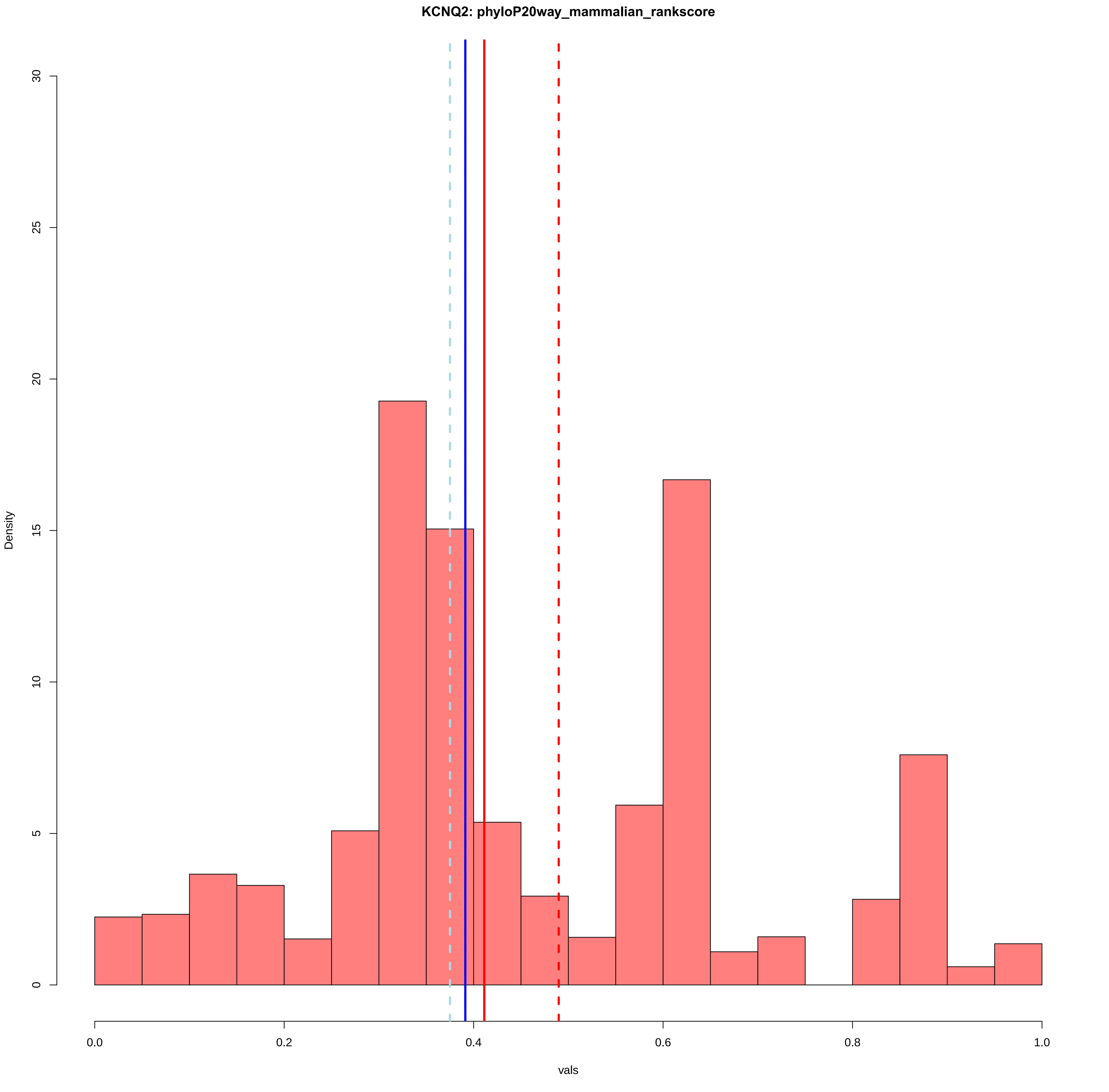
KCNQ2: phyloP20way_mammalian_rankscore
Density
vals
0
5
10
15
20
25
30
0.0
0.2
0.4
0.6
0.8
1.0

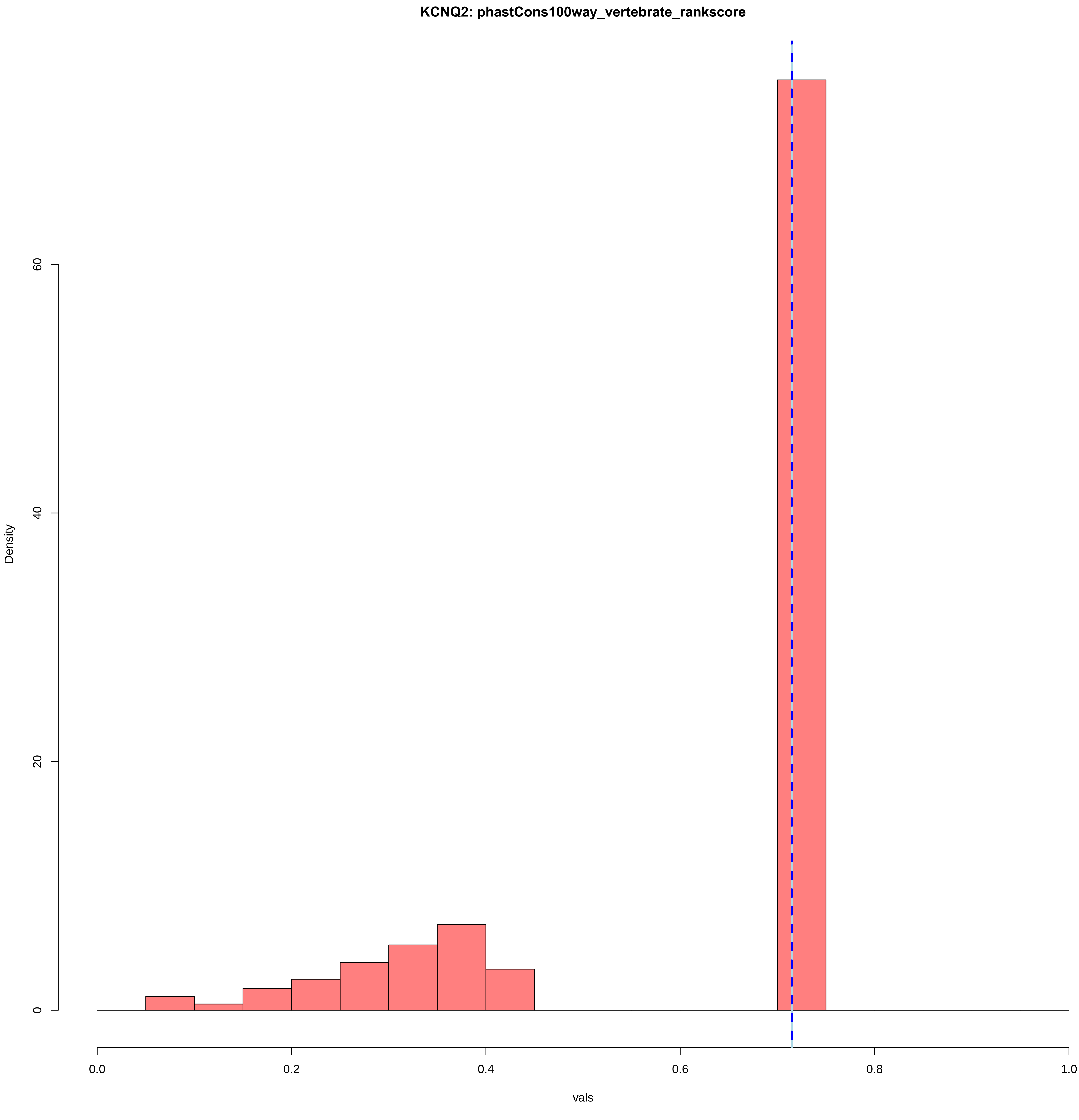
KCNQ2: phastCons100way_vertebrate_rankscore
Density
vals
0
20
40
60
0.0
0.2
0.4
0.6
0.8
1.0

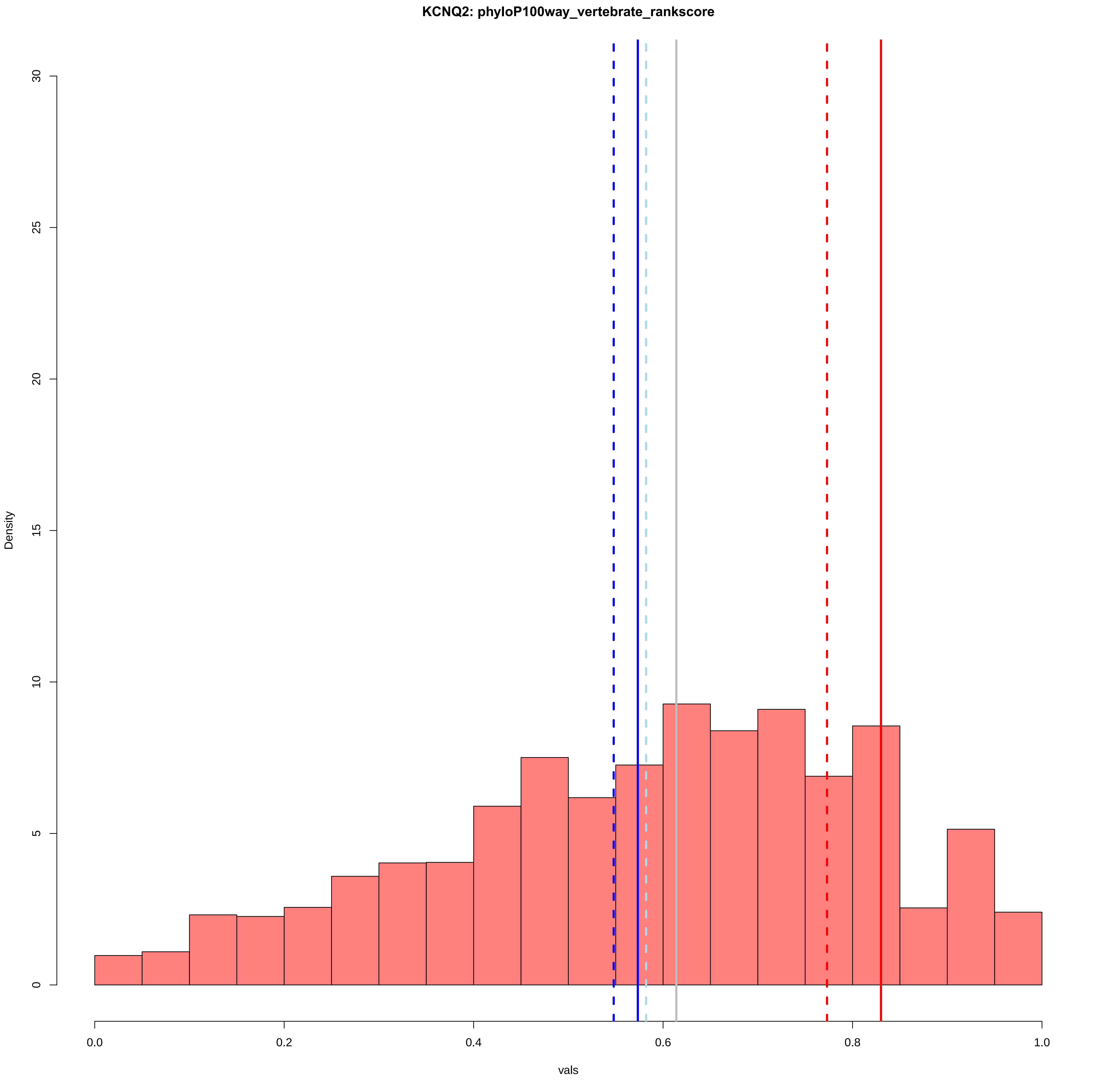
KCNQ2: phyloP100way_vertebrate_rankscore
Density
0
5
10
15
20
25
30
0.0
0.2
0.4
0.6
0.8
1.0
vals

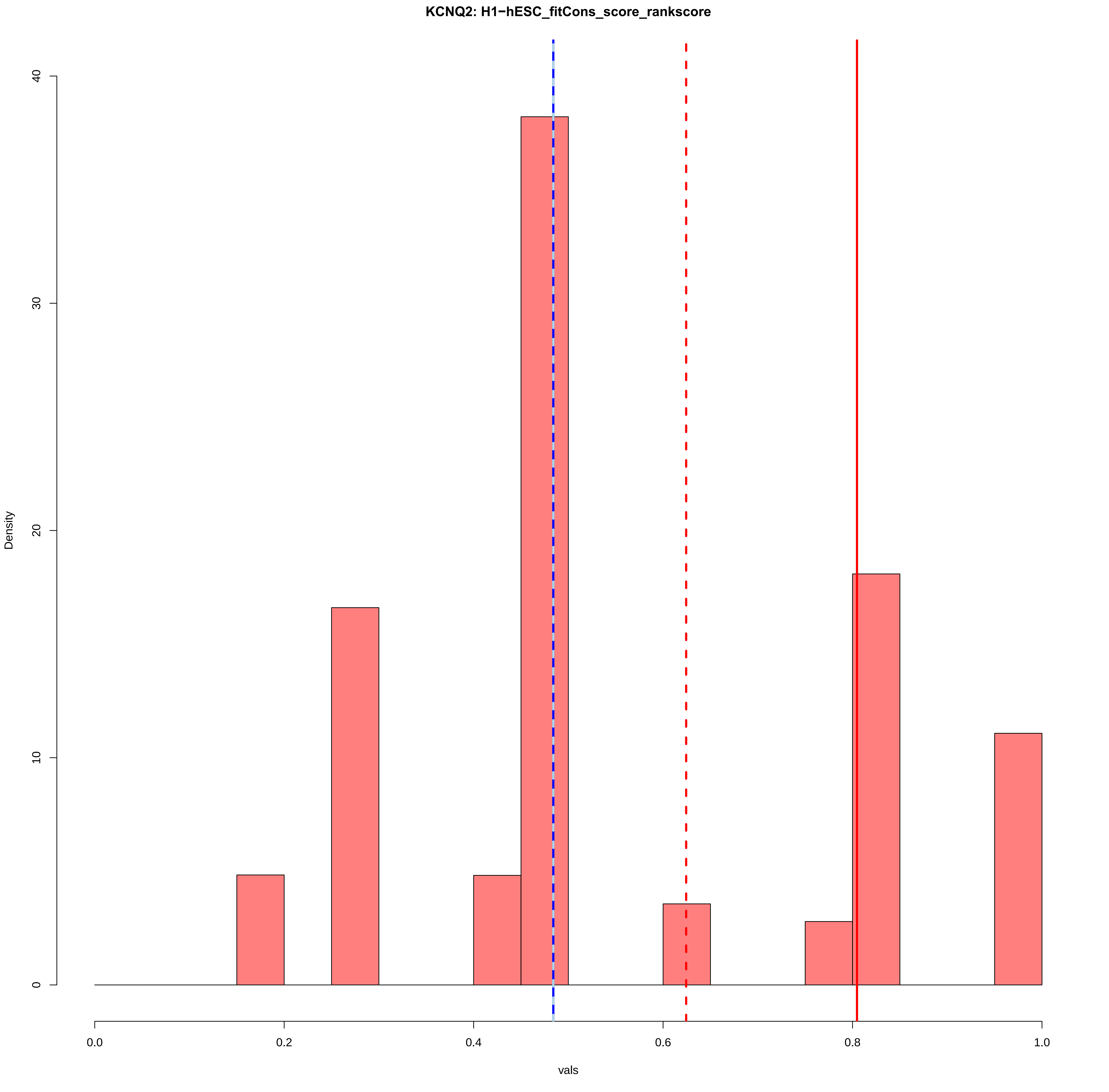
KCNQ2: H1−hESC_fitCons_score_rankscore
Density
0
10
20
30
40
0.0
0.2
0.4
0.6
0.8
1.0
vals

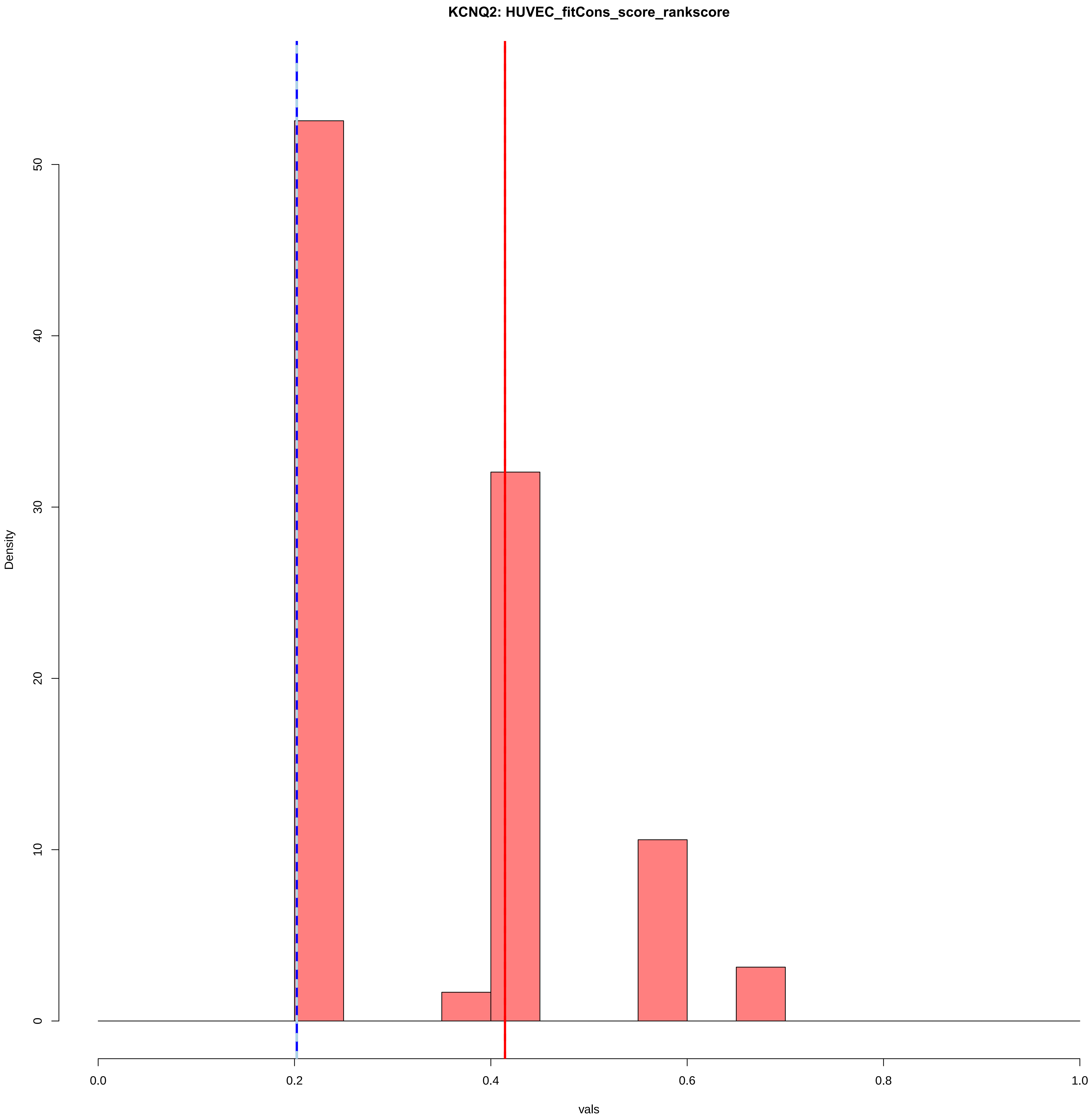
KCNQ2: HUVEC_fitCons_score_rankscore
Density
vals
0
10
20
30
40
50
0.0
0.2
0.4
0.6
0.8
1.0

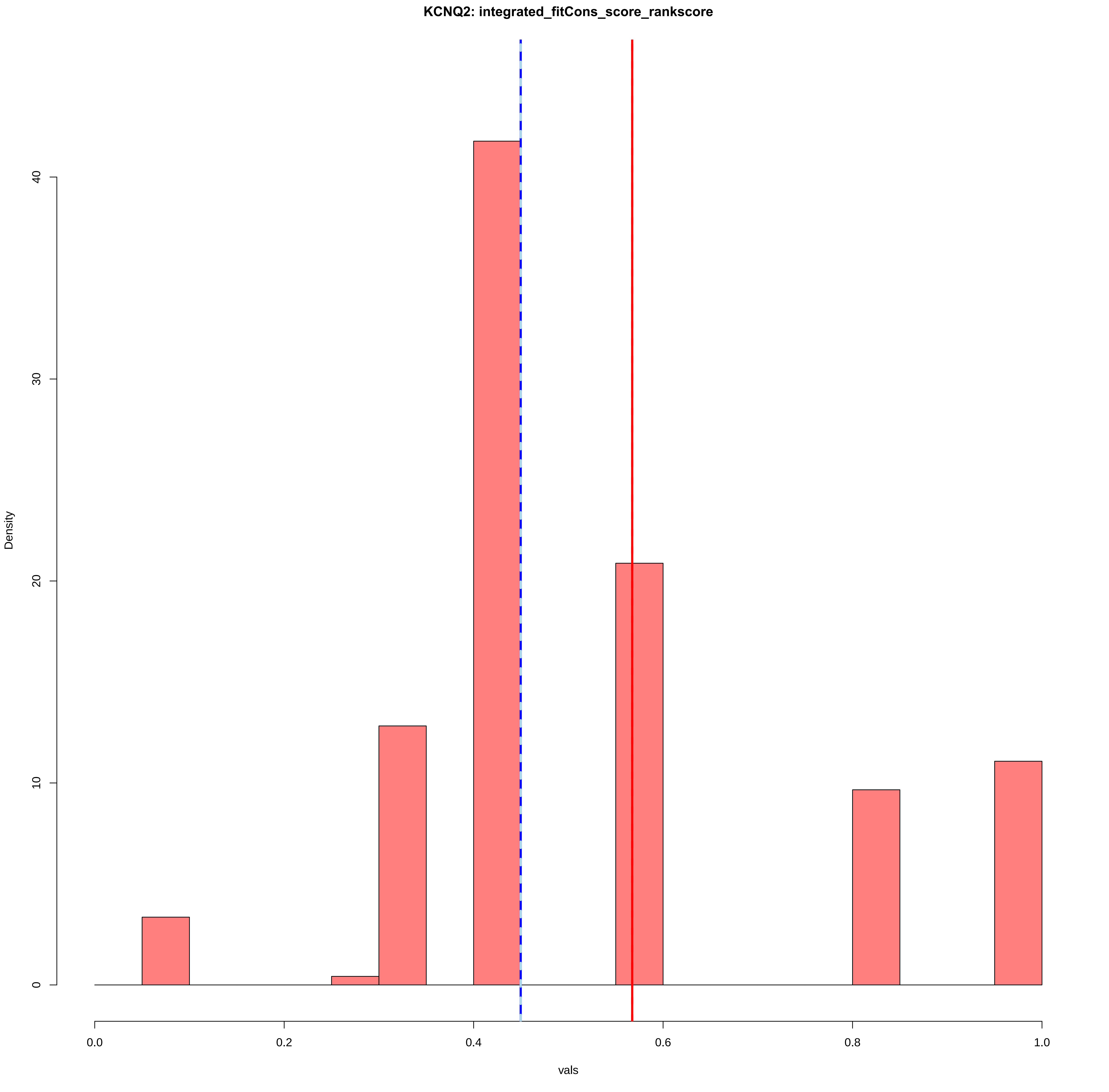

KCNQ2: integrated_fitCons_score_rankscore
Density
0
10
20
30
40
0.0
0.2
0.4
0.6
0.8
1.0
vals

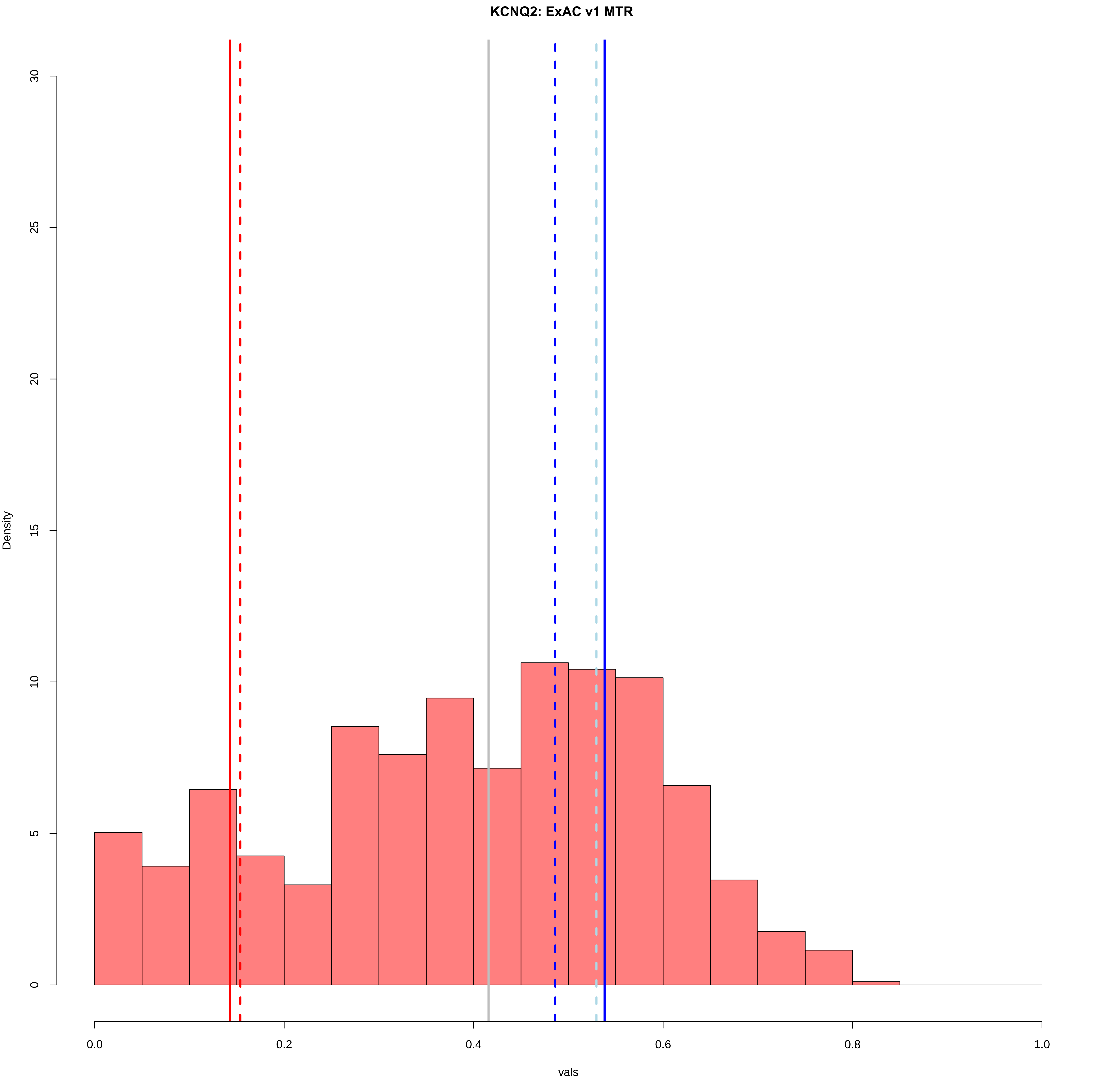
KCNQ2: ExAC v1 MTR
Density
vals
0
5
10
15
20
25
30
0.0
0.2
0.4
0.6
0.8
1.0

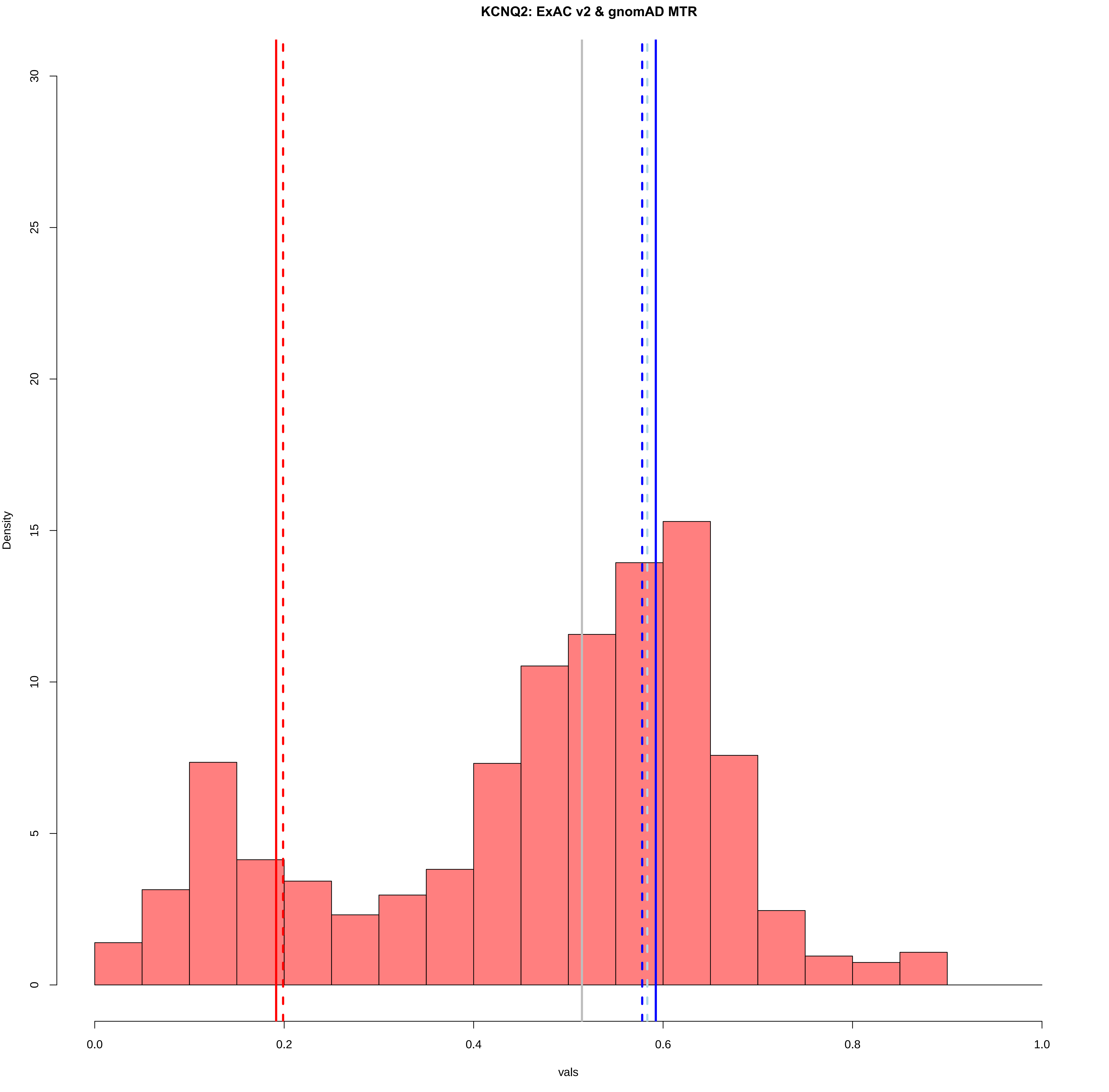
KCNQ2: ExAC v2 & gnomAD MTR
Density
vals
0
5
10
15
20
25
30
0.0
0.2
0.4
0.6
0.8
1.0

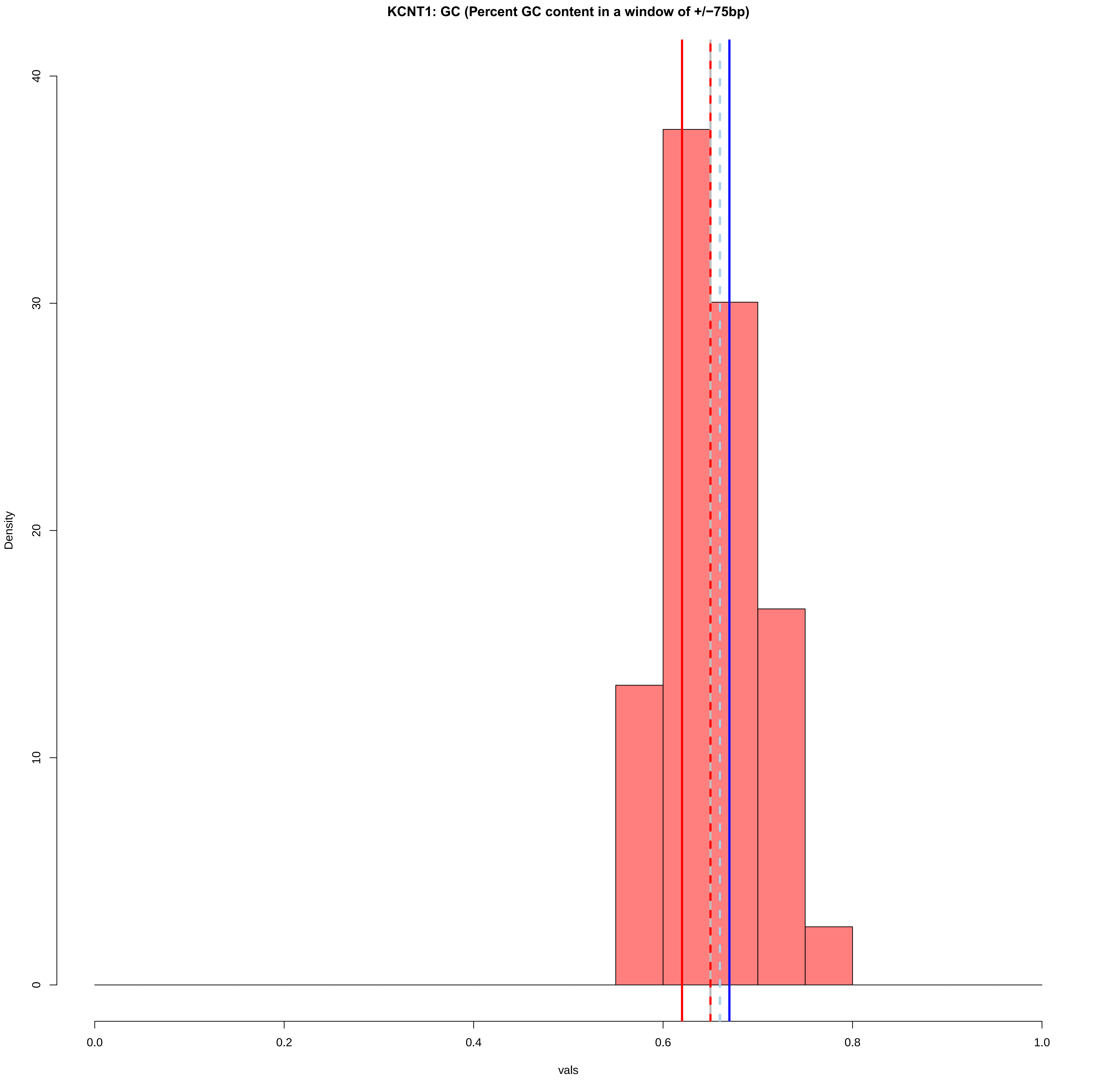
KCNT1: GC (Percent GC content in a window of +/−75bp)
Density
vals
0
10
20
30
40
0.0
0.2
0.4
0.6
0.8
1.0

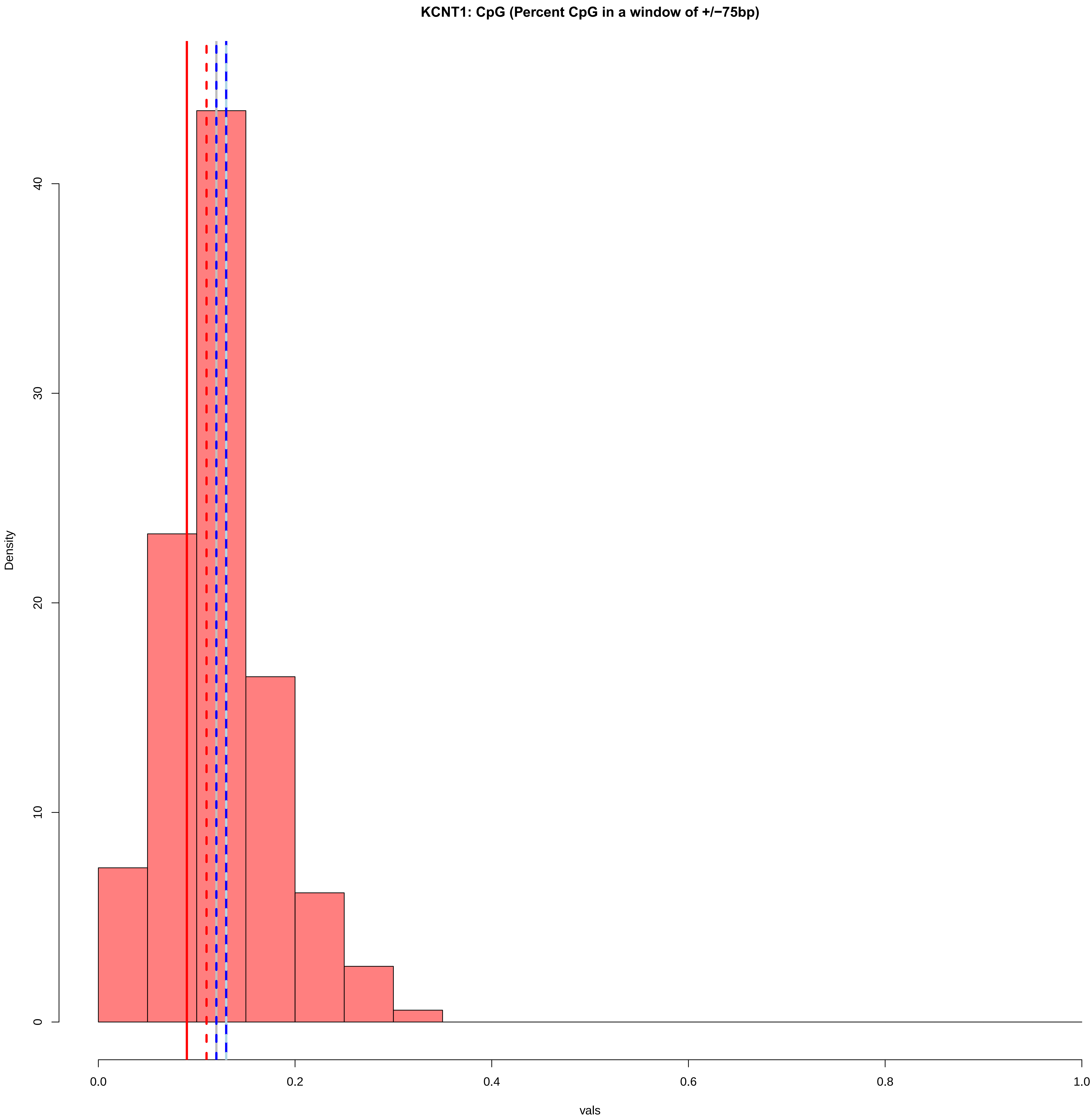

KCNT1: CpG (Percent CpG in a window of +/−75bp)
Density
0
10
20
30
40
0.0
0.2
0.4
0.6
0.8
1.0
vals

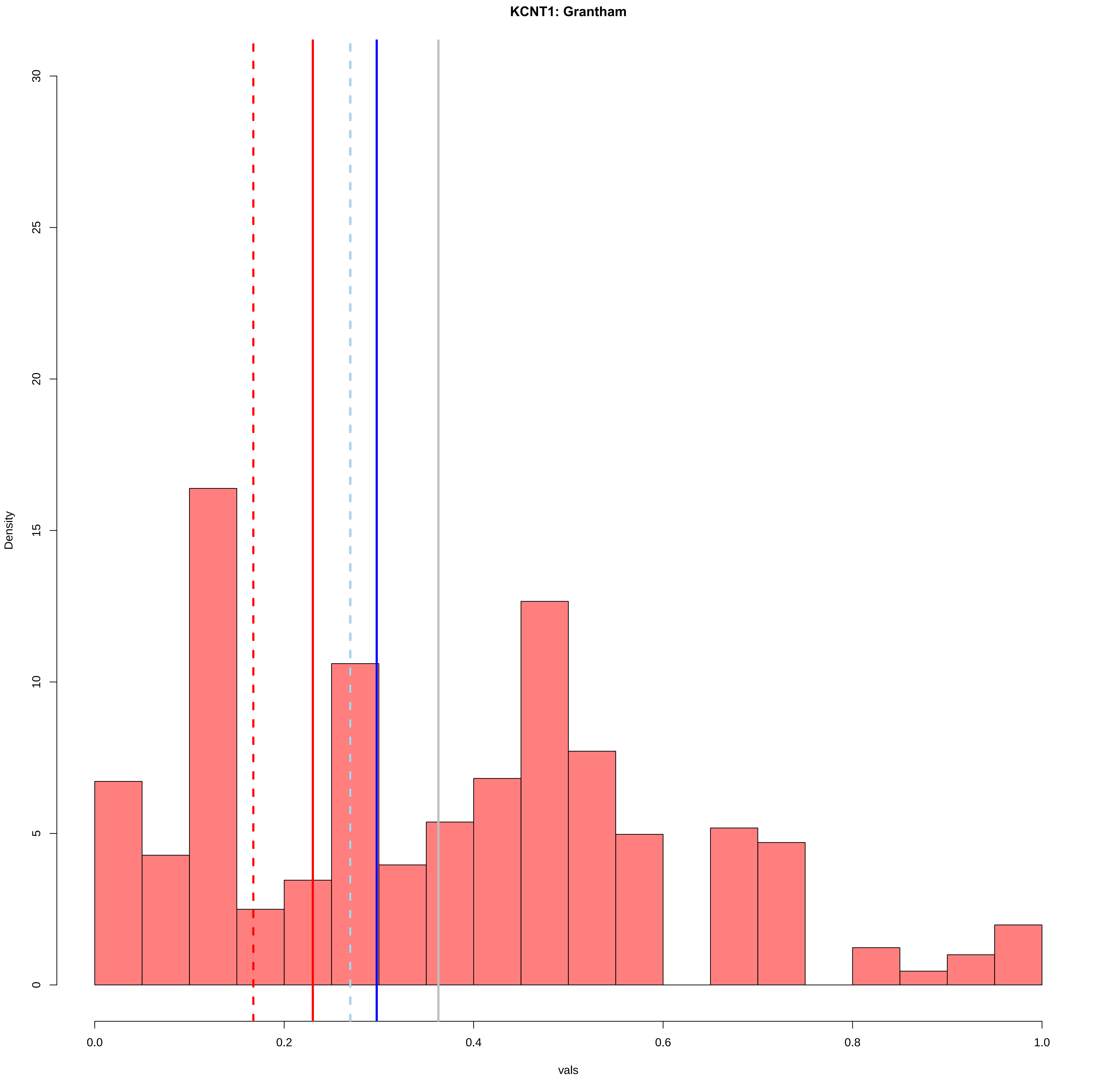

KCNT1: Grantham
Density
vals
0
5
10
15
20
25
30
0.0
0.2
0.4
0.6
0.8
1.0

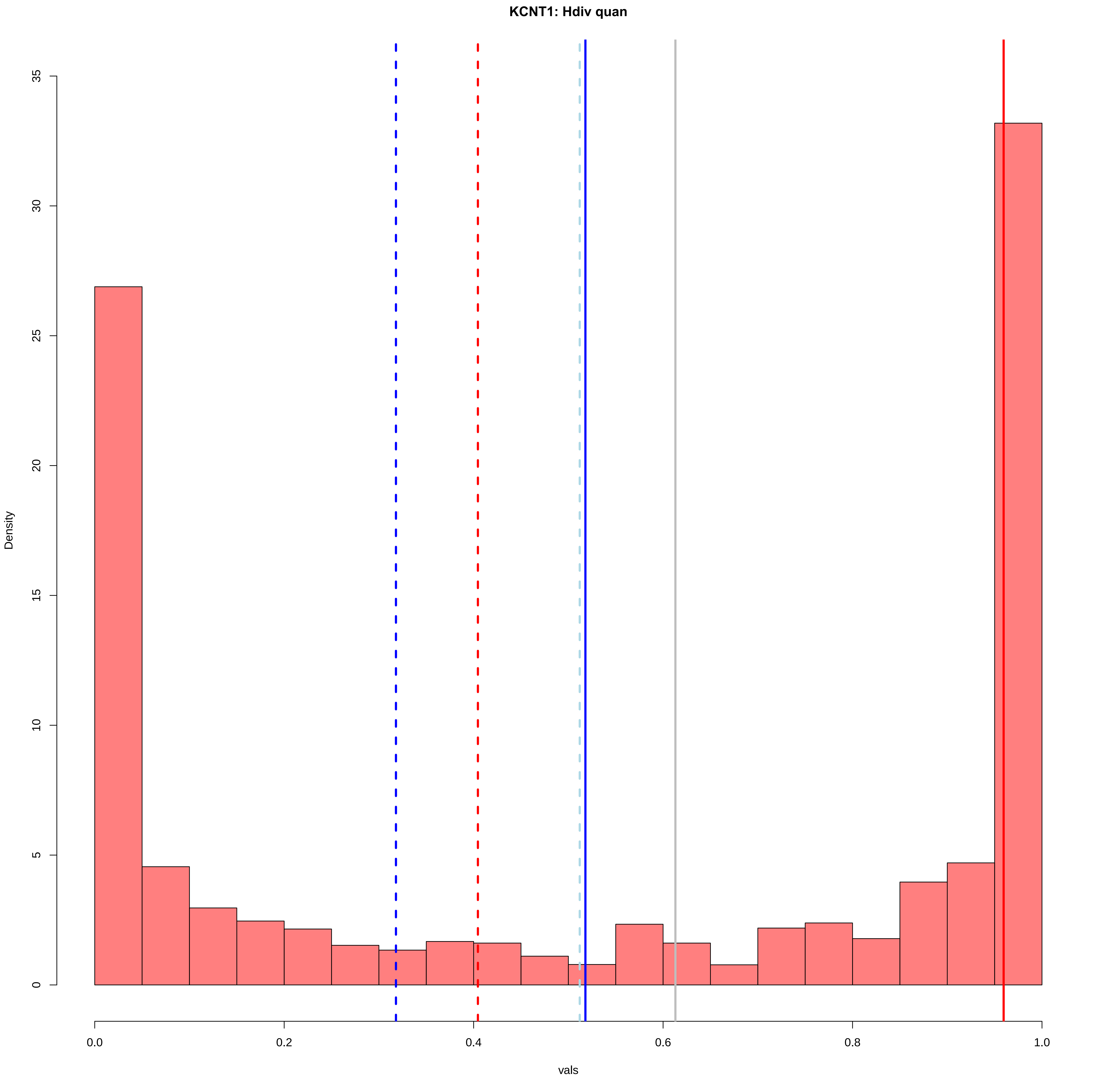
KCNT1: Hdiv quan
Density
vals
0.0
0.2
0.4
0.6
0.8
1.0
0
5
10
15
20
25
30
35

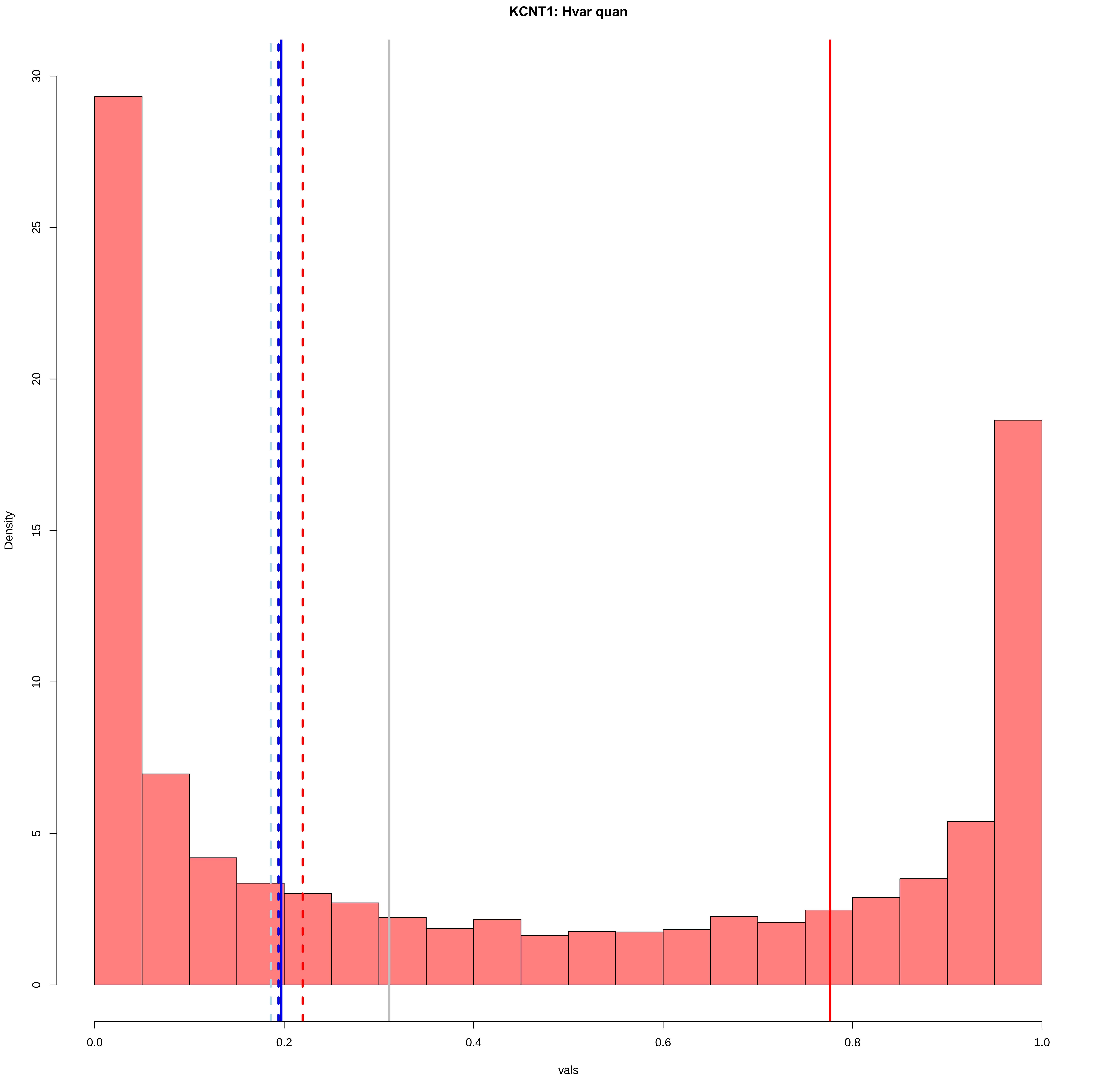

KCNT1: Hvar quan
Density
vals
0
5
10
15
20
25
30
0.0
0.2
0.4
0.6
0.8
1.0

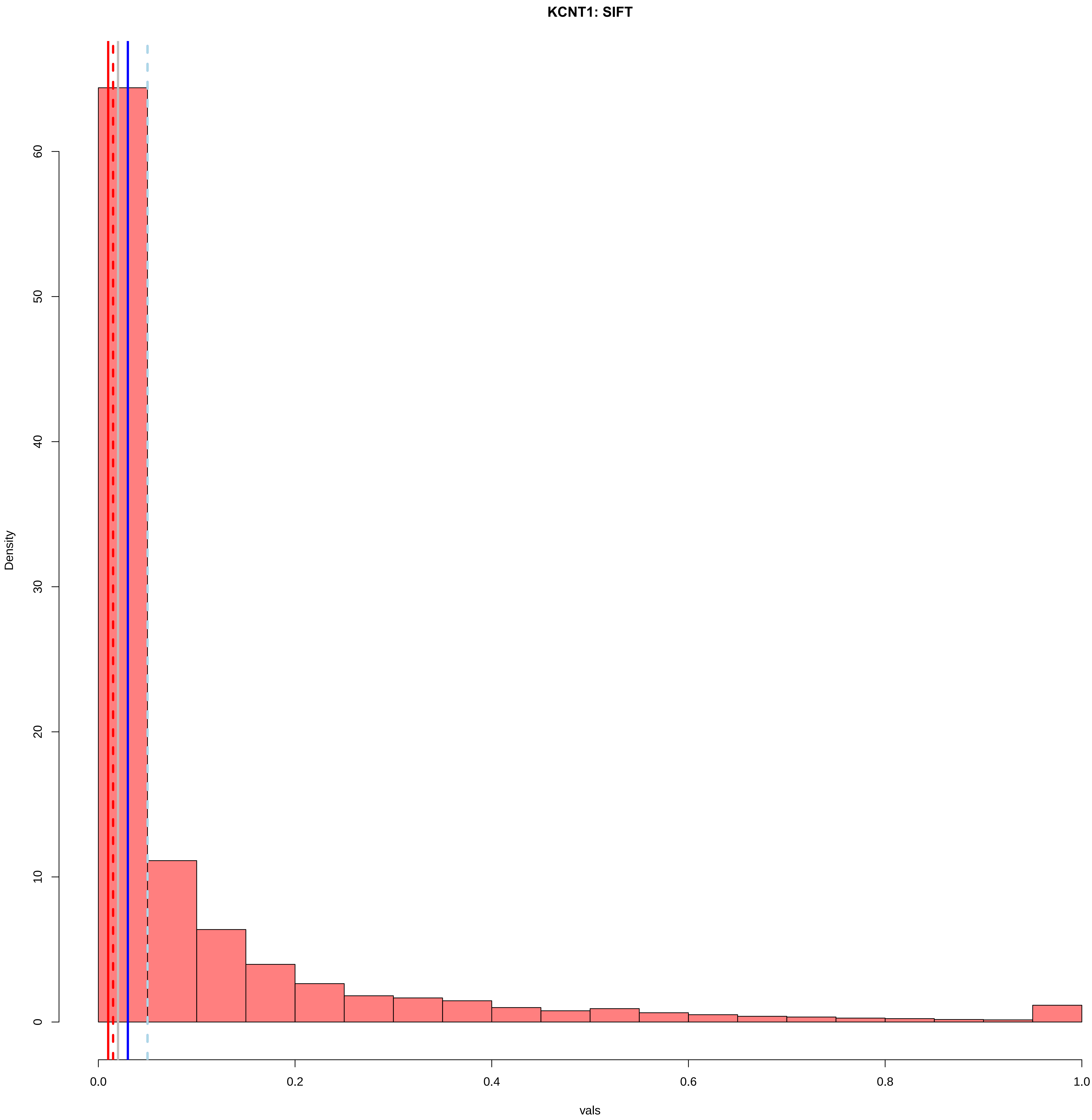
KCNT1: SIFT
Density
0
10
20
30
40
50
60
vals
0.0
0.2
0.4
0.6
0.8
1.0

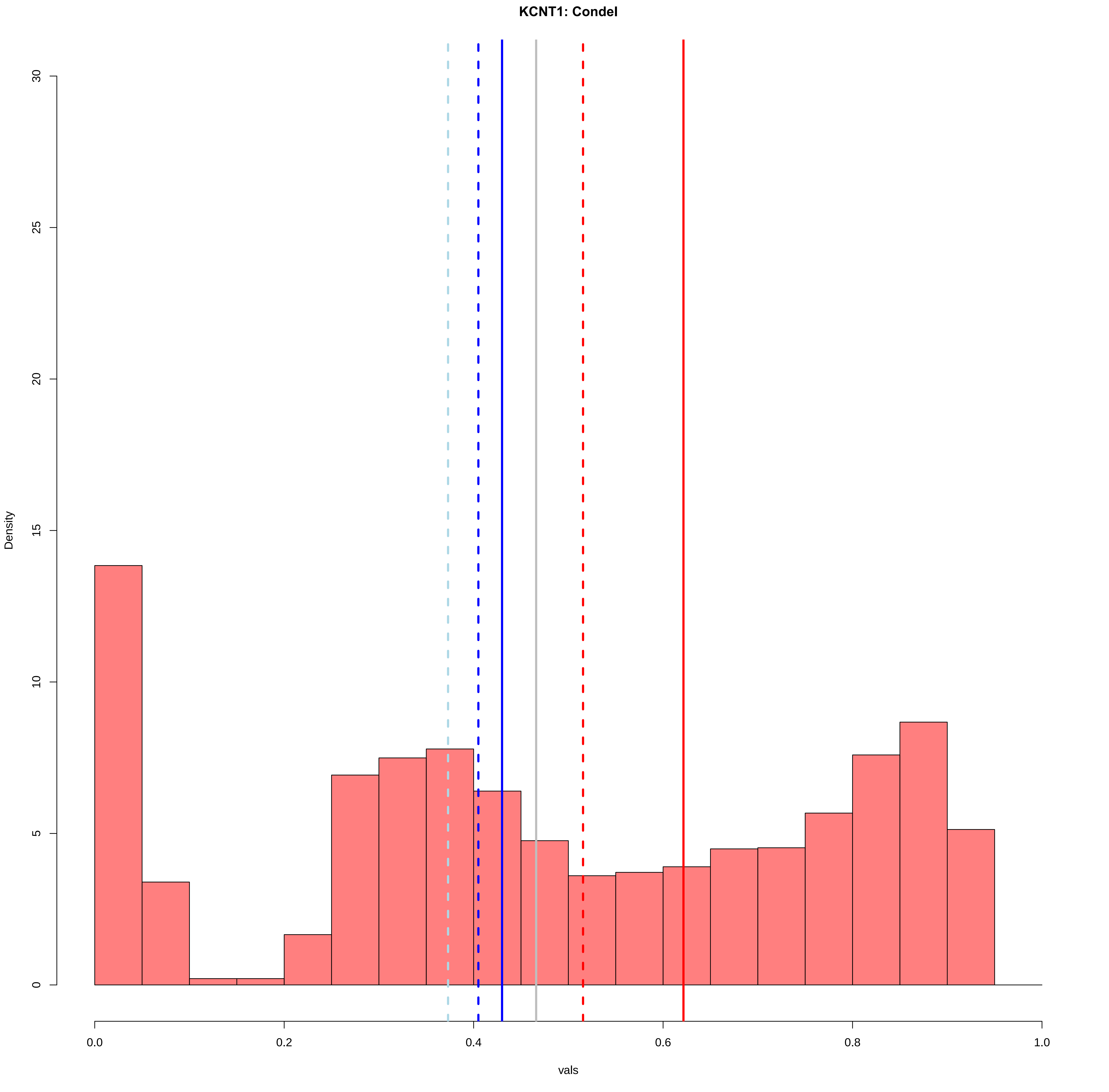
KCNT1: Condel
Density
vals
0
5
10
15
20
25
30
0.0
0.2
0.4
0.6
0.8
1.0

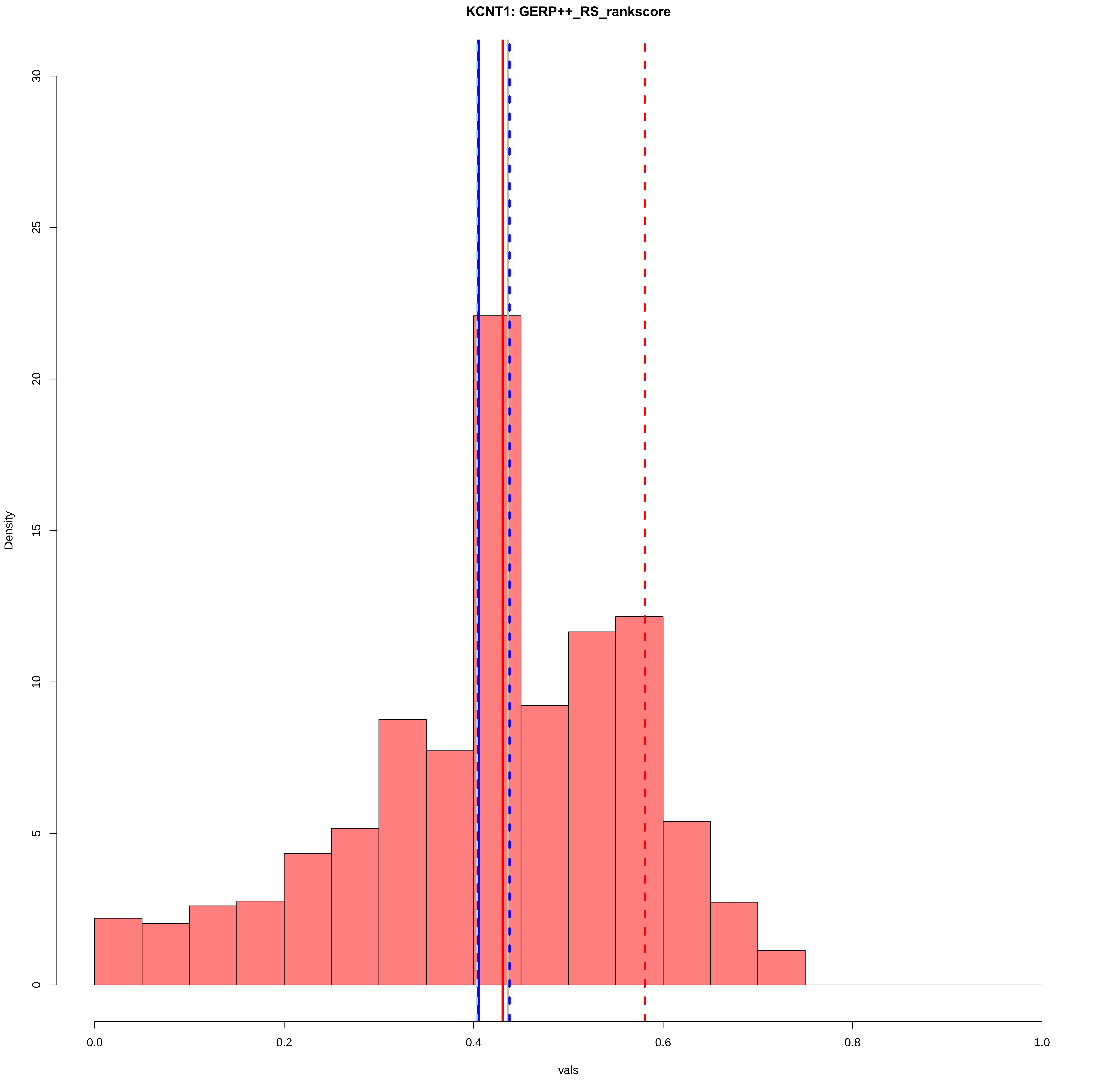
KCNT1: GERP++_RS_rankscore
Density
vals
0
5
10
15
20
25
30
0.0
0.2
0.4
0.6
0.8
1.0

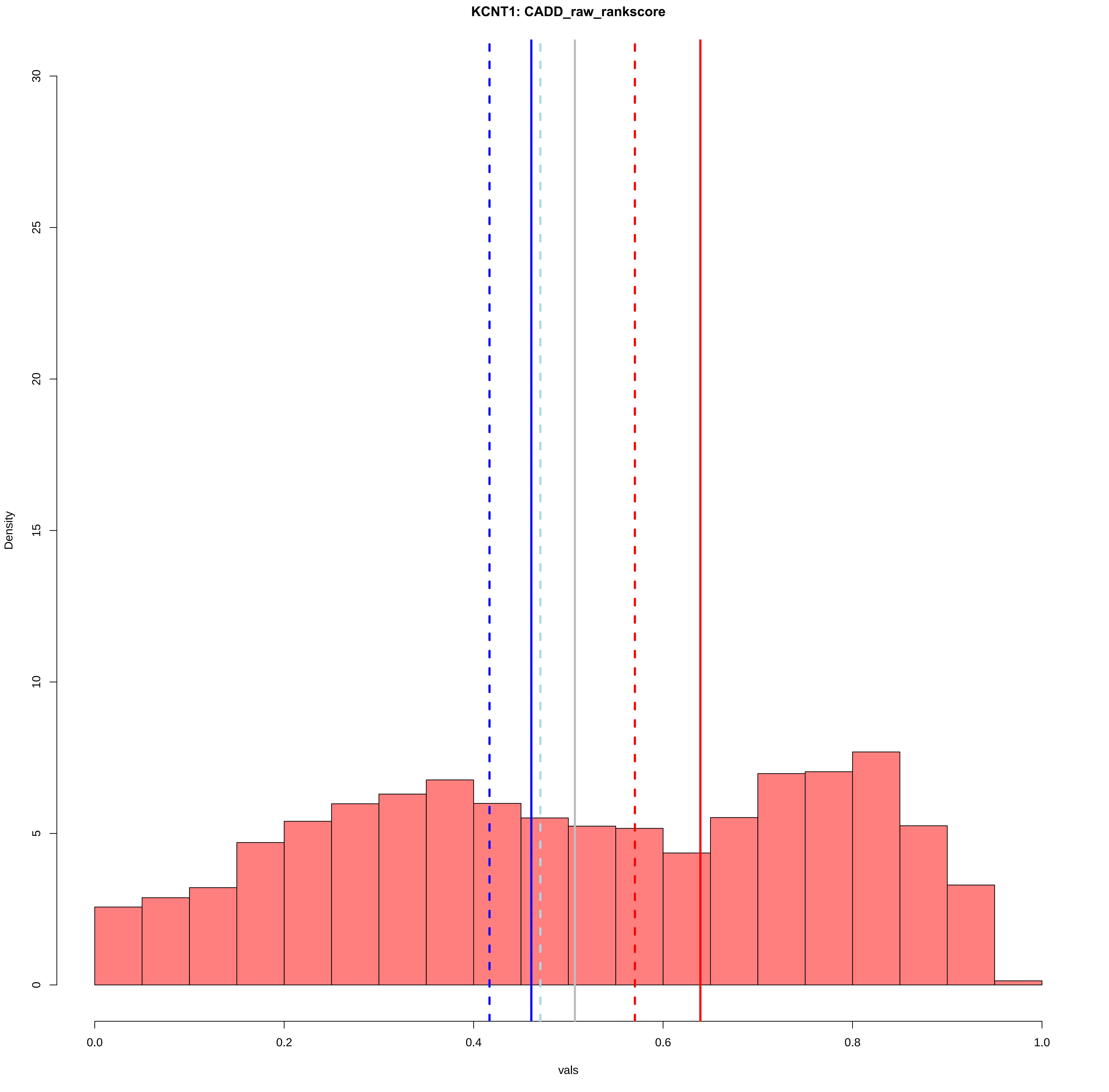

KCNT1: CADD_raw_rankscore
Density
0
5
10
15
20
25
30
0.0
0.2
0.4
0.6
0.8
1.0
vals

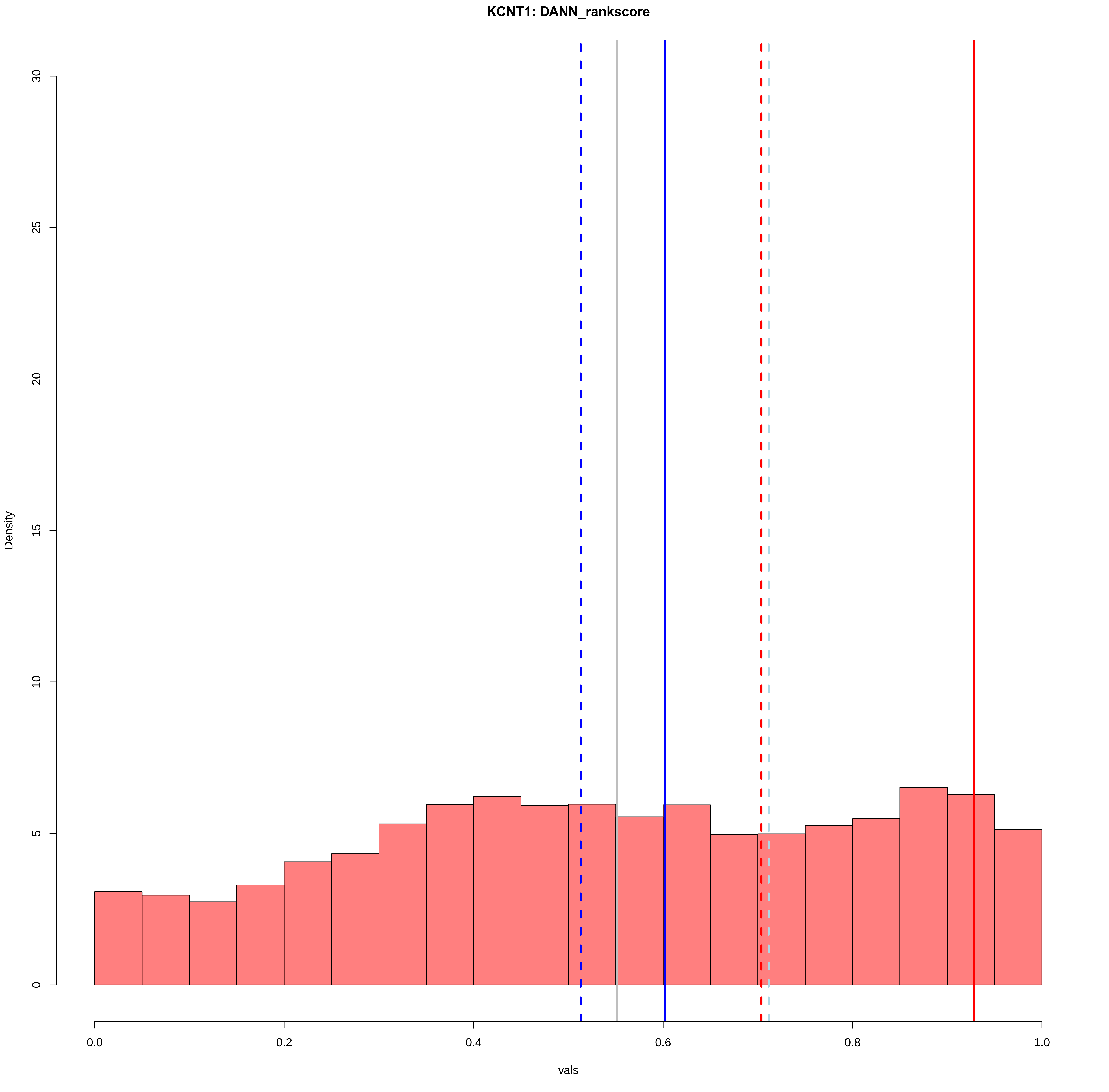
KCNT1: DANN_rankscore
Density
vals
0
5
10
15
20
25
30
0.0
0.2
0.4
0.6
0.8
1.0

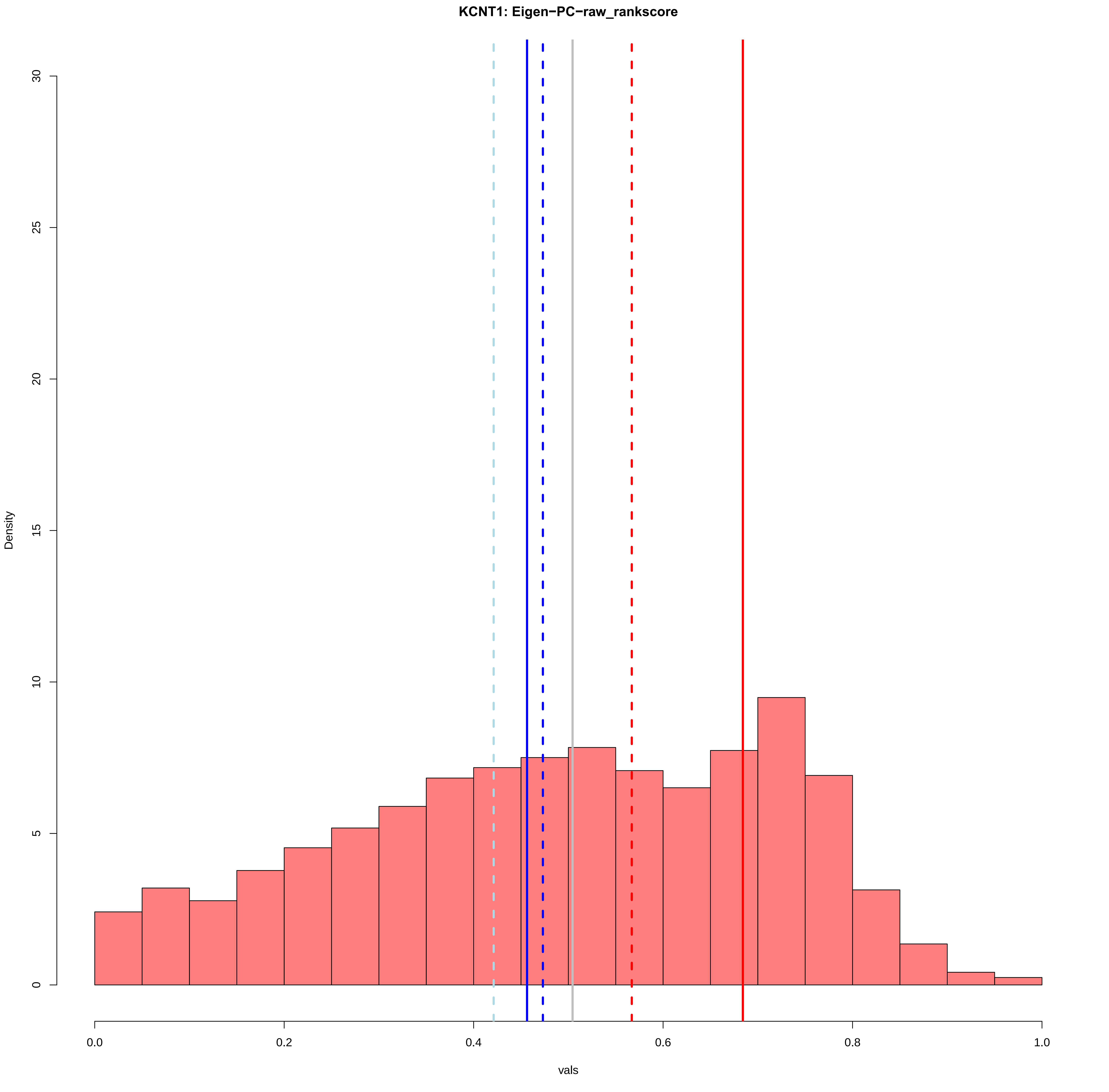
KCNT1: Eigen−PC−raw_rankscore
Density
vals
0
5
10
15
20
25
30
0.0
0.2
0.4
0.6
0.8
1.0

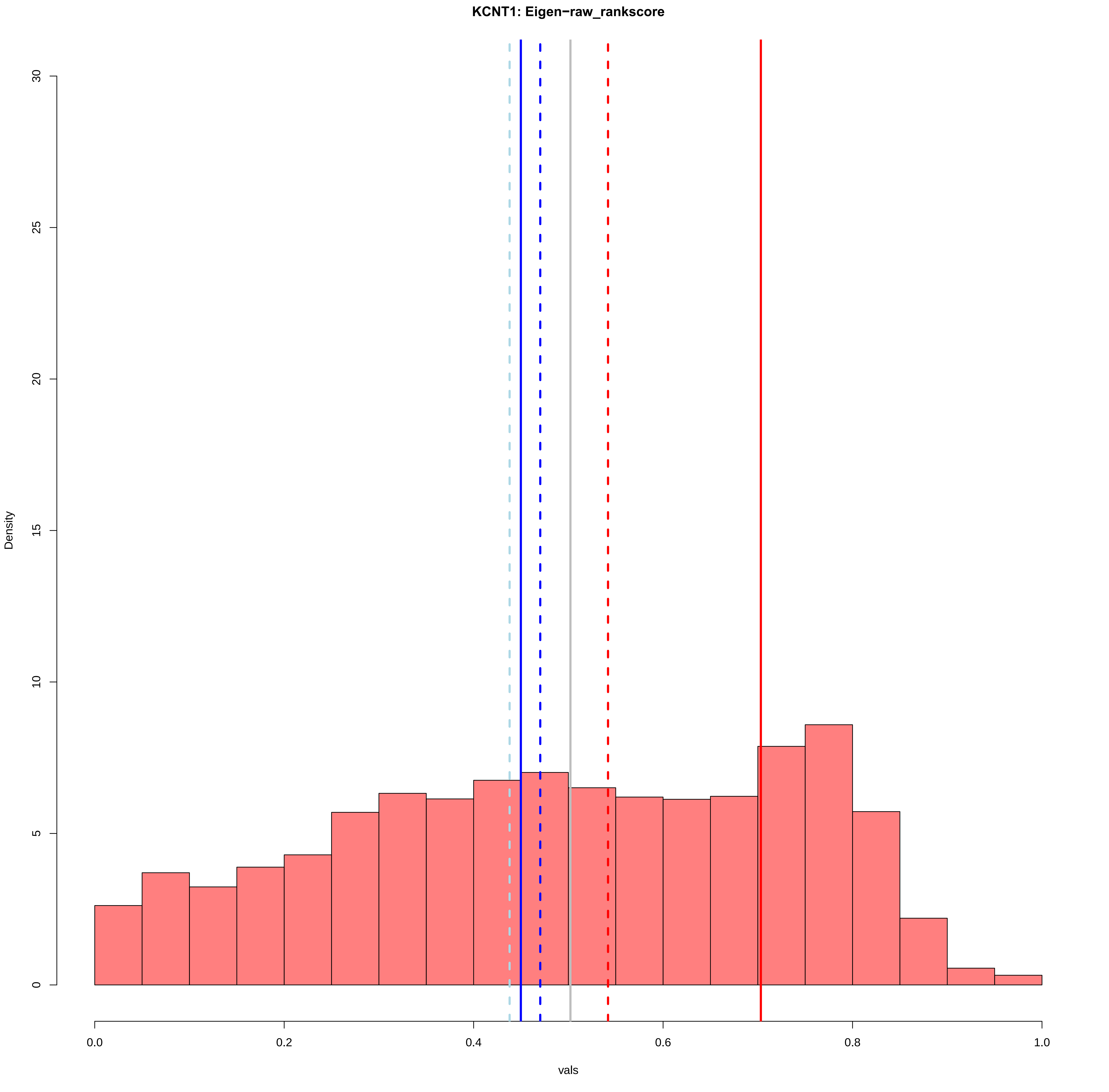
KCNT1: Eigen−raw_rankscore
Density
vals
0
5
10
15
20
25
30
0.0
0.2
0.4
0.6
0.8
1.0

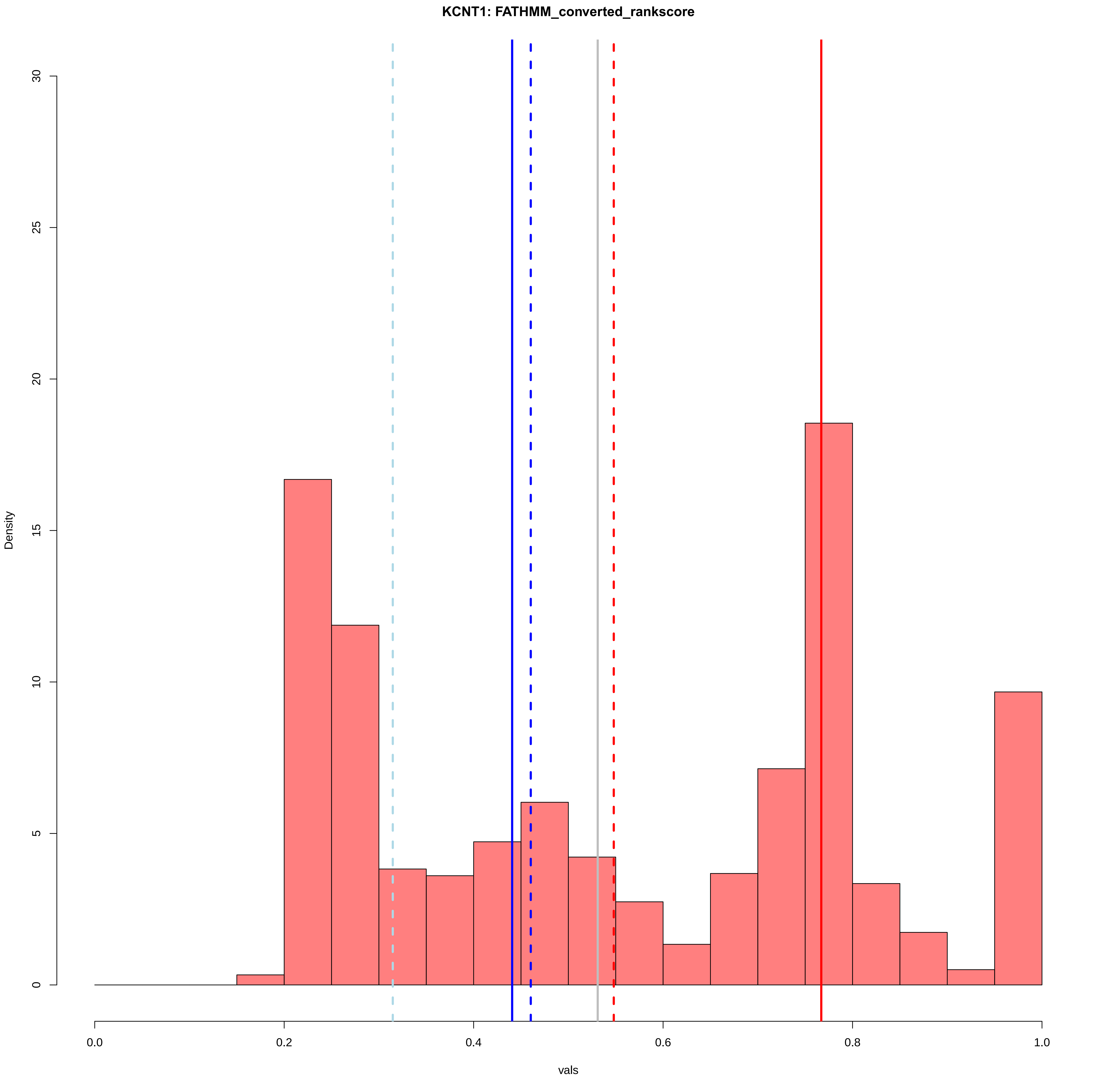
KCNT1: FATHMM_converted_rankscore
Density
vals
0
5
10
15
20
25
30
0.0
0.2
0.4
0.6
0.8
1.0

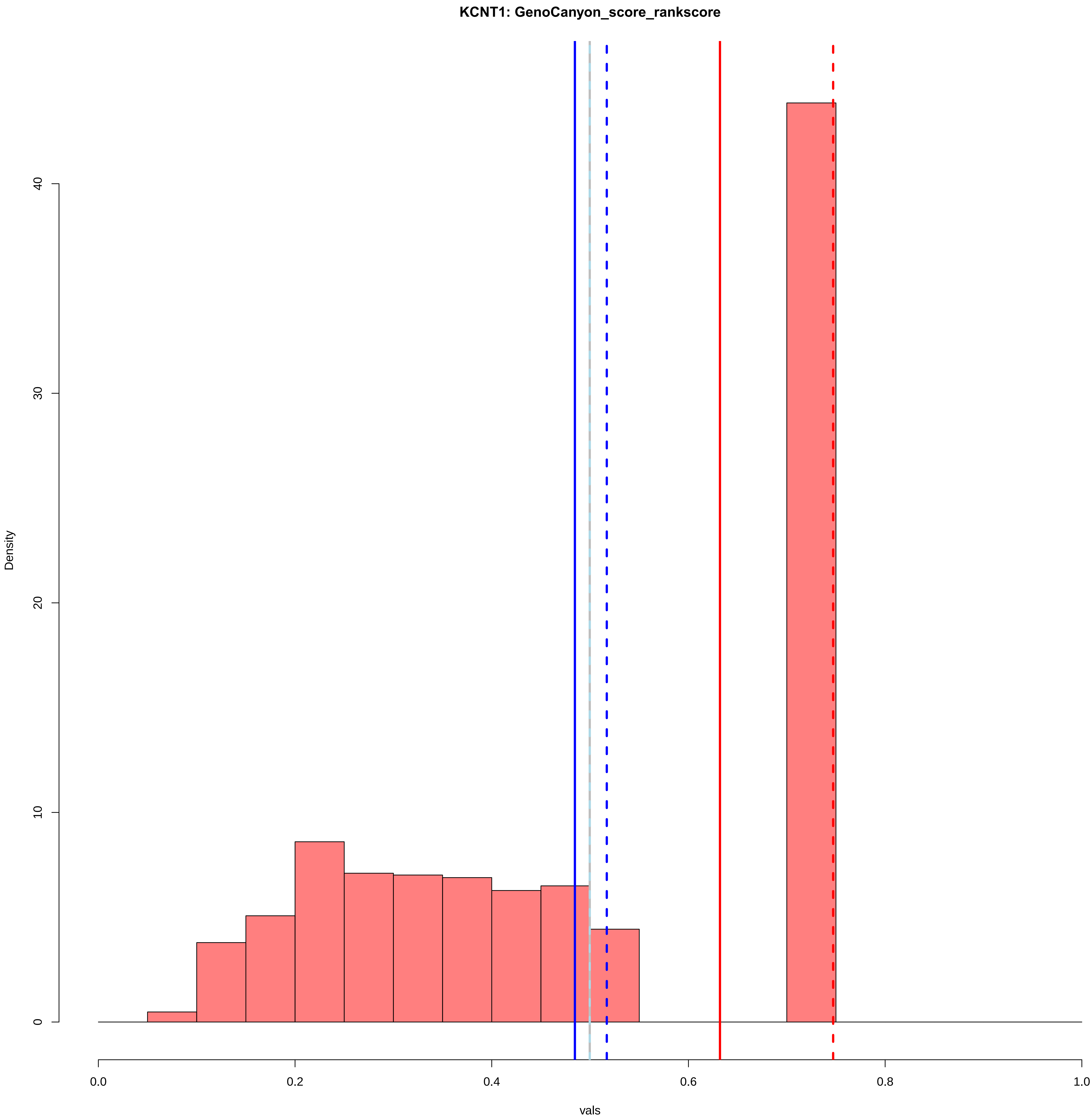
KCNT1: GenoCanyon_score_rankscore
Density
vals
0
10
20
30
40
0.0
0.2
0.4
0.6
0.8
1.0

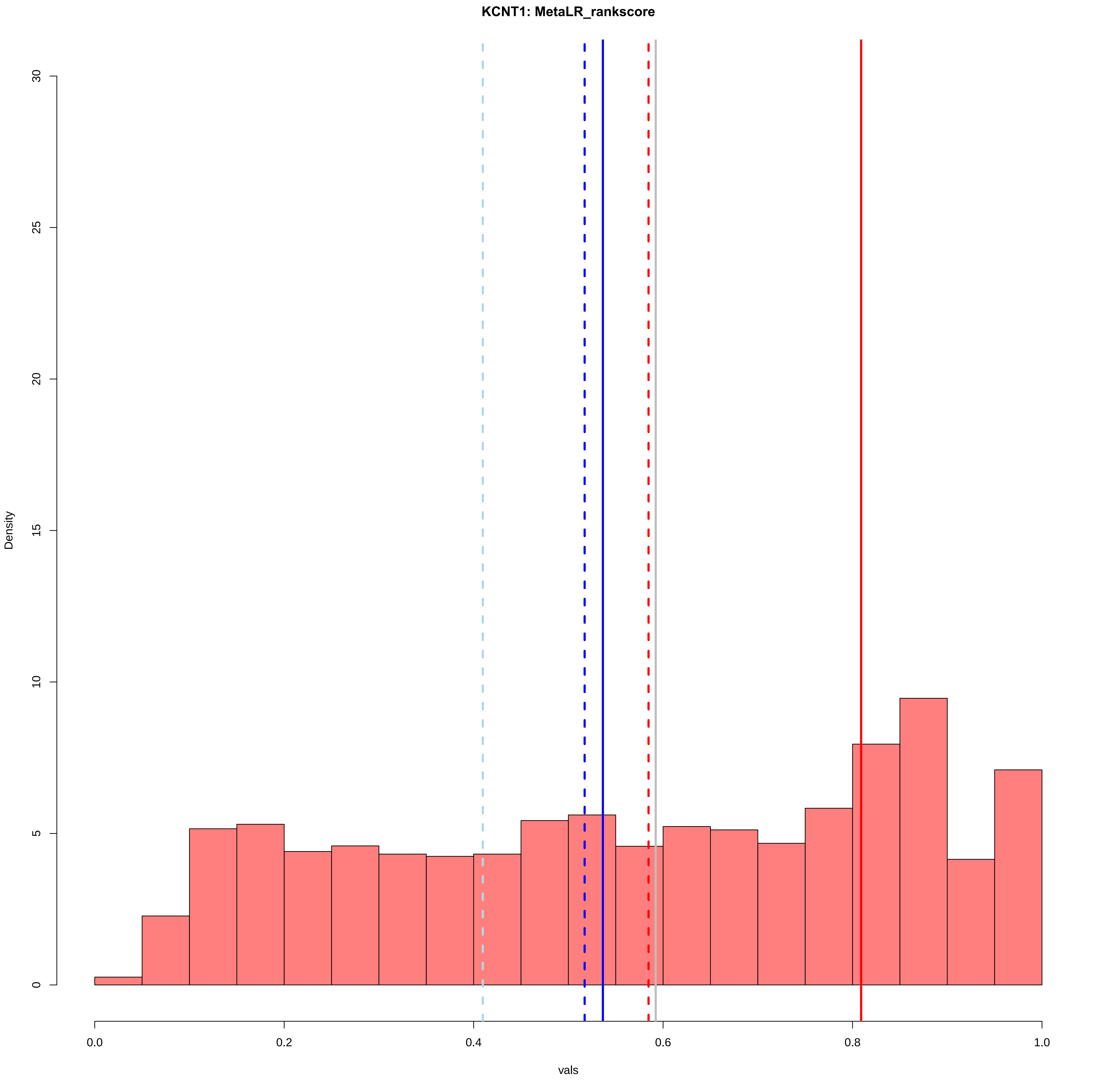

KCNT1: MetaLR_rankscore
Density
vals
0
5
10
15
20
25
30
0.0
0.2
0.4
0.6
0.8
1.0

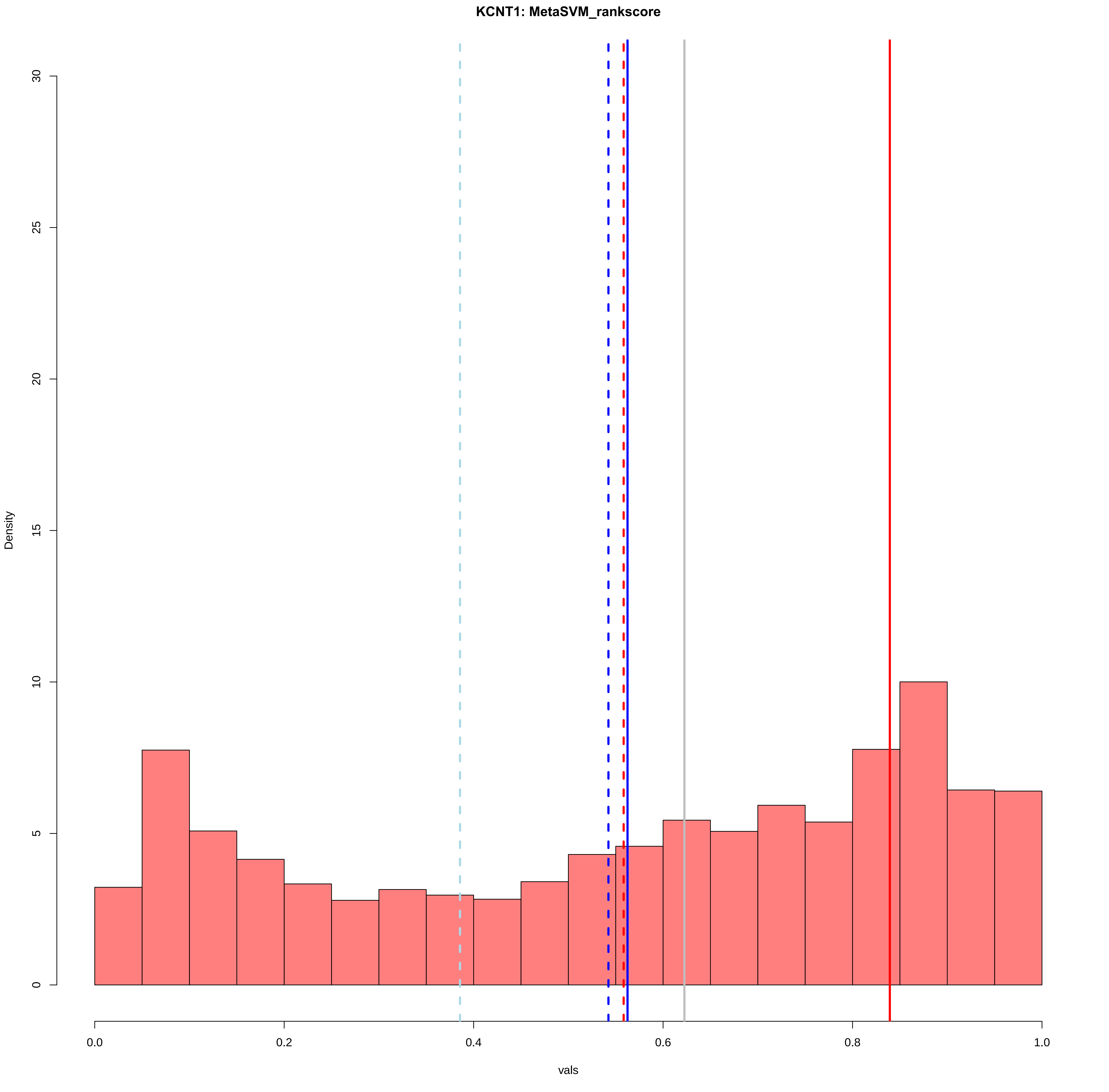
KCNT1: MetaSVM_rankscore
Density
vals
0
5
10
15
20
25
30
0.0
0.2
0.4
0.6
0.8
1.0

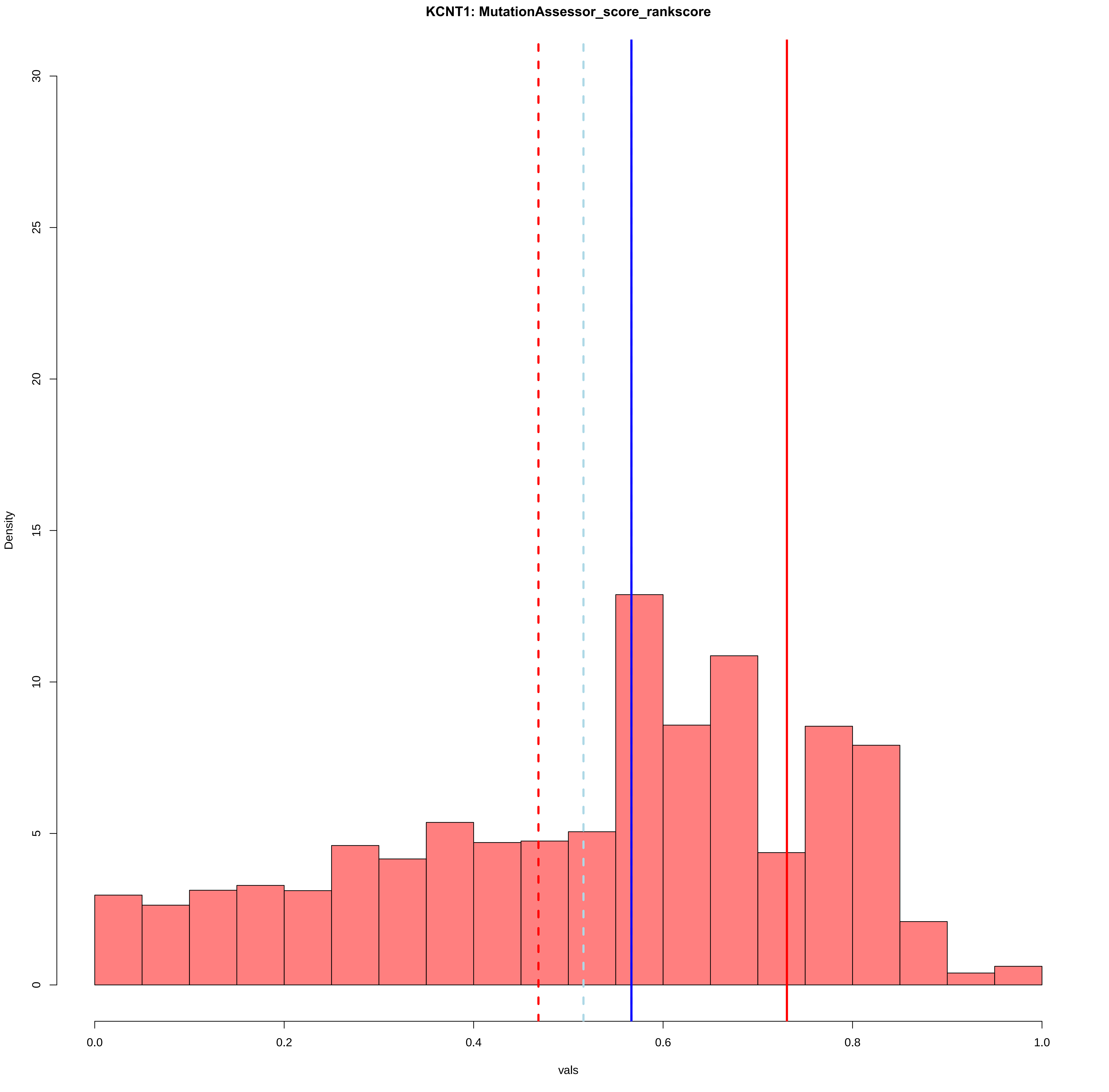
KCNT1: MutationAssessor_score_rankscore
Density
0
5
10
15
20
25
30
0.0
0.2
0.4
0.6
0.8
1.0
vals

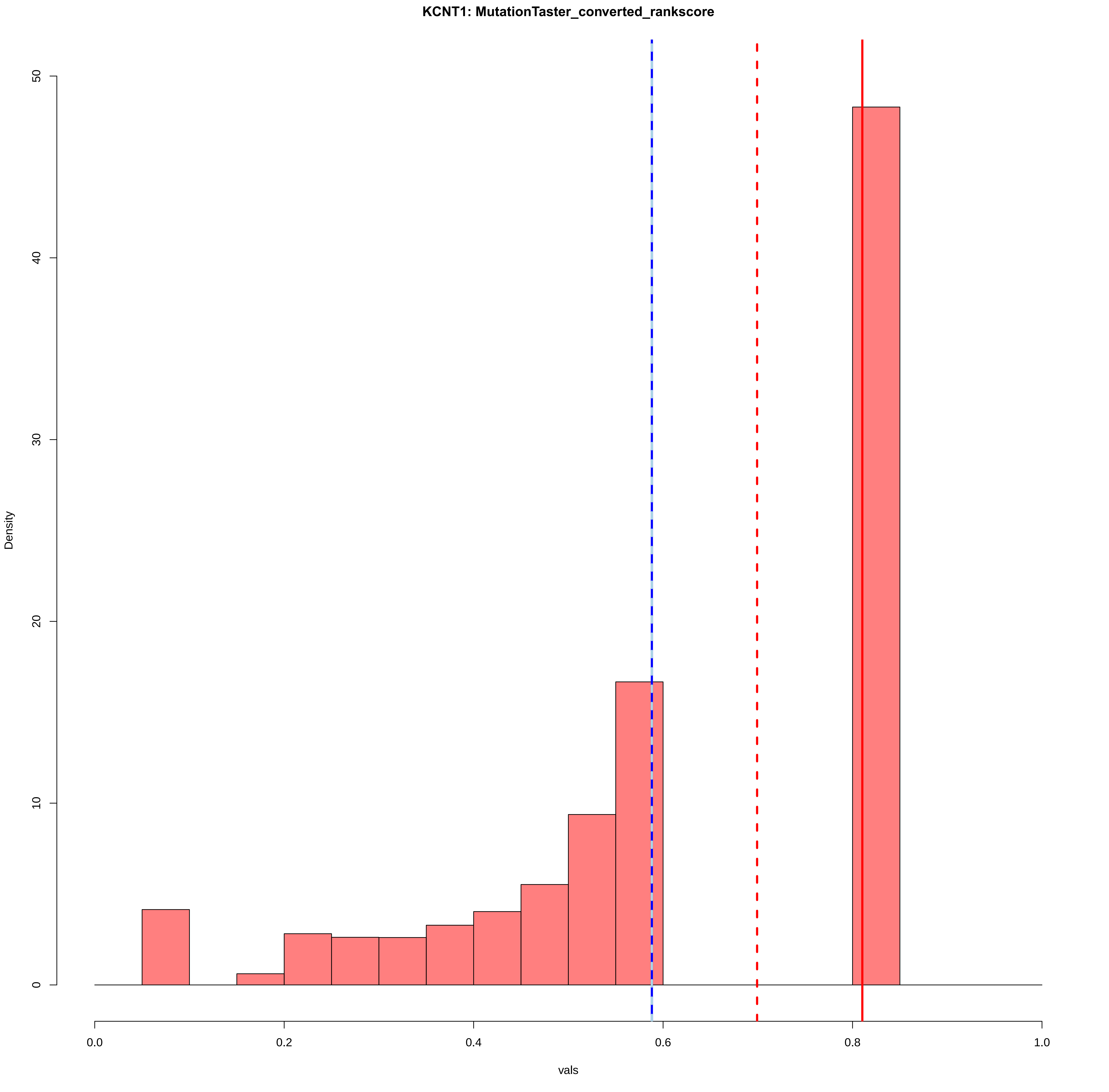
KCNT1: MutationTaster_converted_rankscore
Density
vals
0
10
20
30
40
50
0.0
0.2
0.4
0.6
0.8
1.0

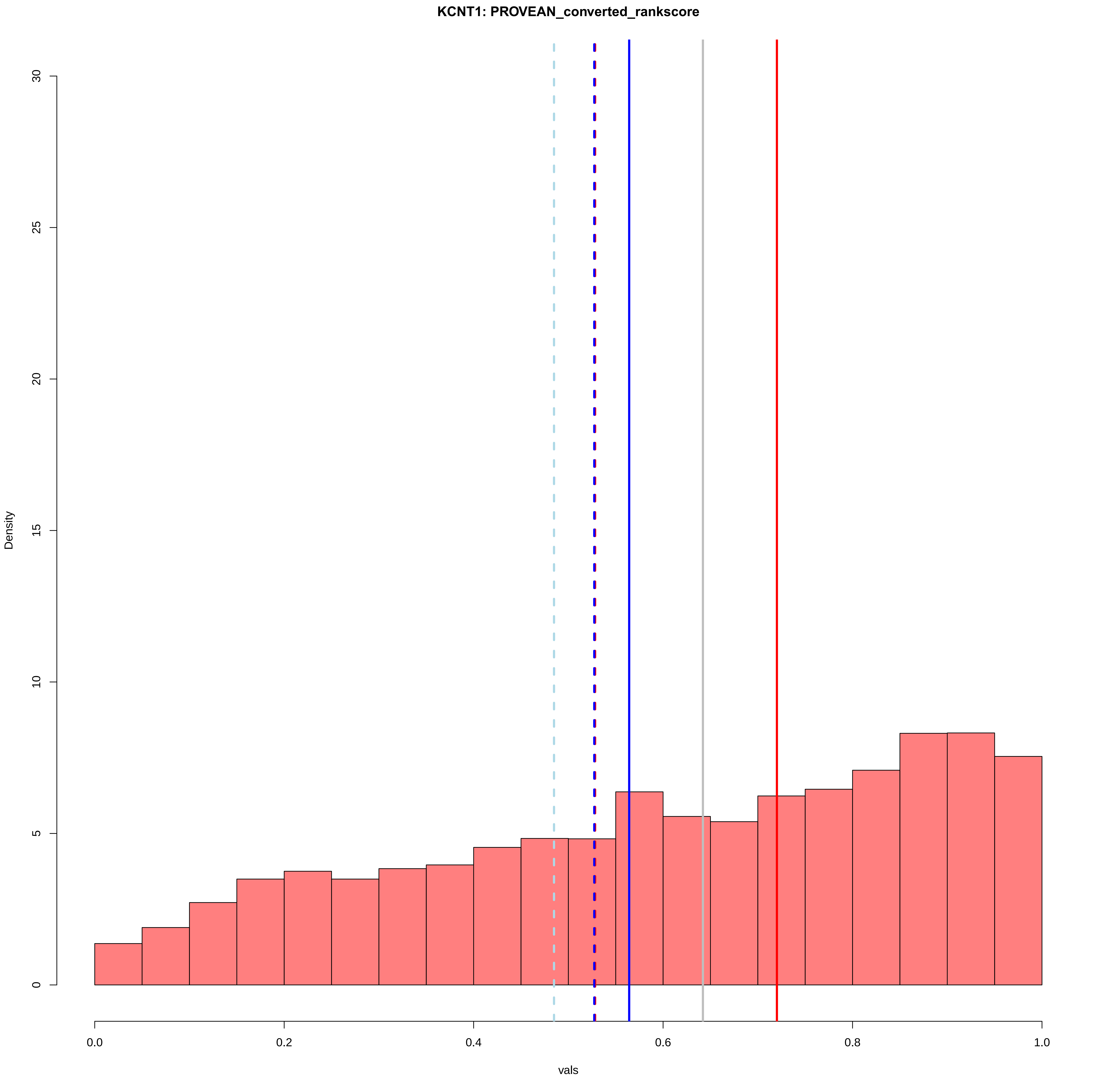
KCNT1: PROVEAN_converted_rankscore
Density
vals
0
5
10
15
20
25
30
0.0
0.2
0.4
0.6
0.8
1.0

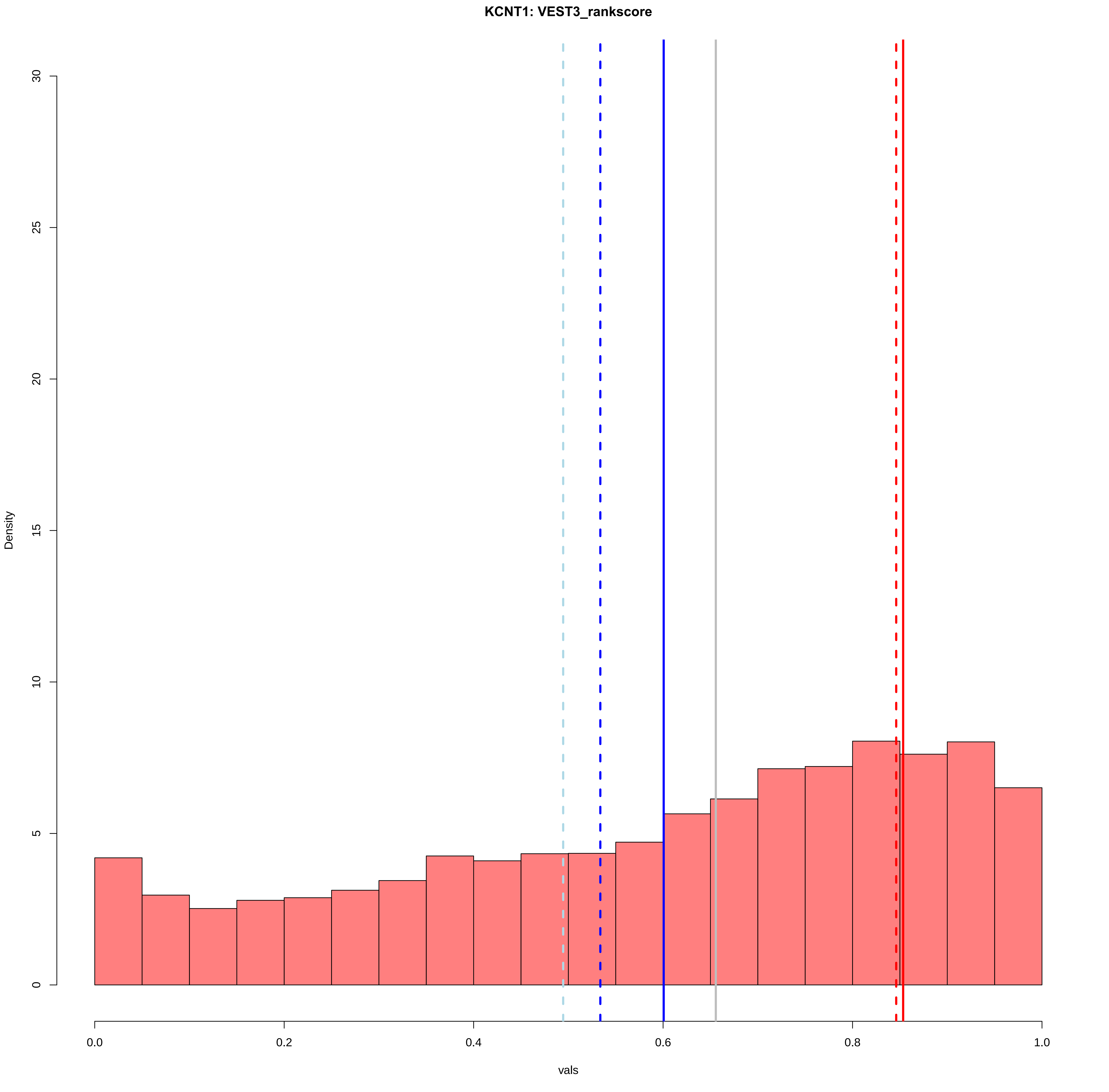

KCNT1: VEST3_rankscore
Density
vals
0
5
10
15
20
25
30
0.0
0.2
0.4
0.6
0.8
1.0

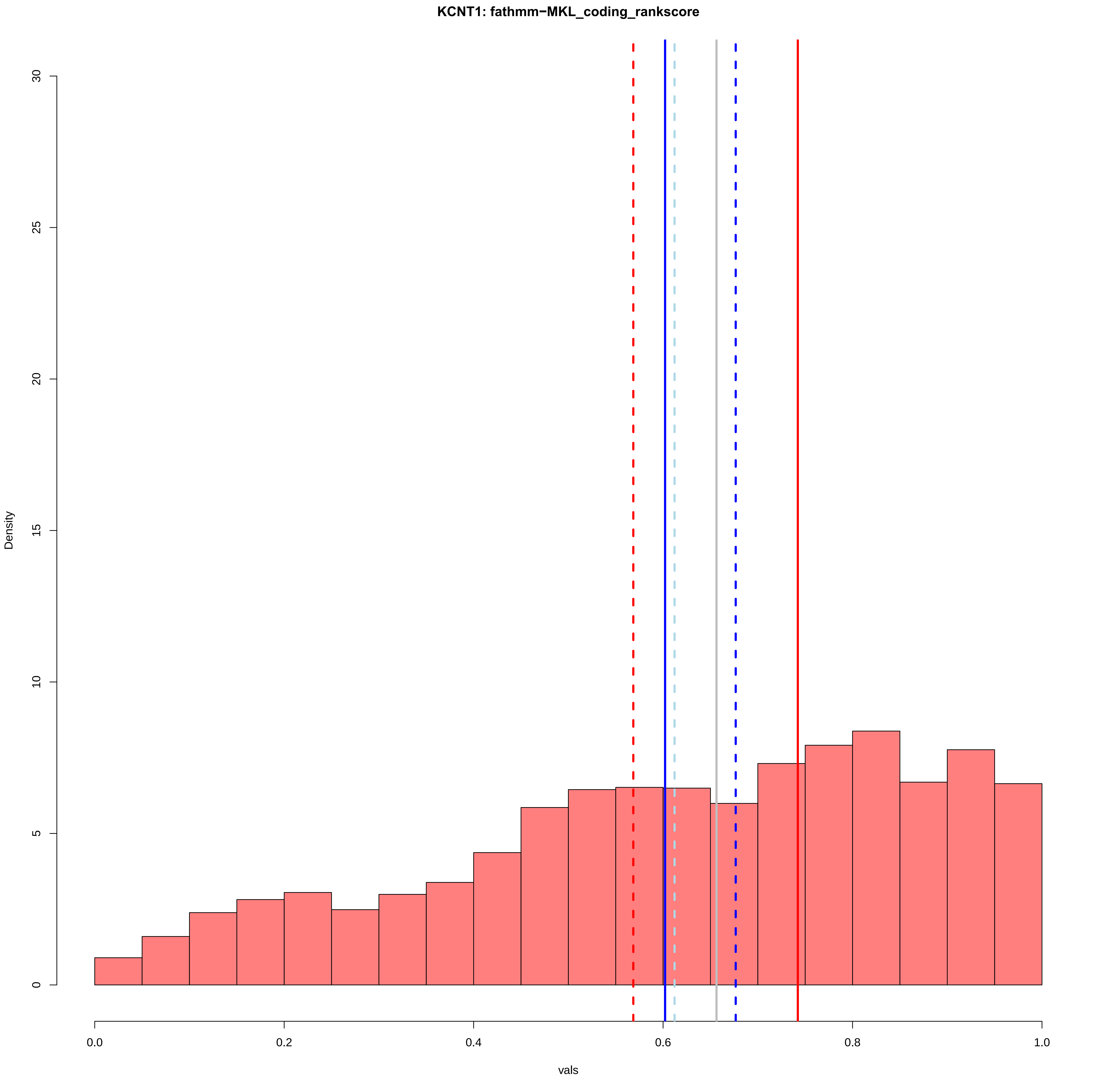

KCNT1: fathmm−MKL_coding_rankscore
Density
vals
0
5
10
15
20
25
30
0.0
0.2
0.4
0.6
0.8
1.0

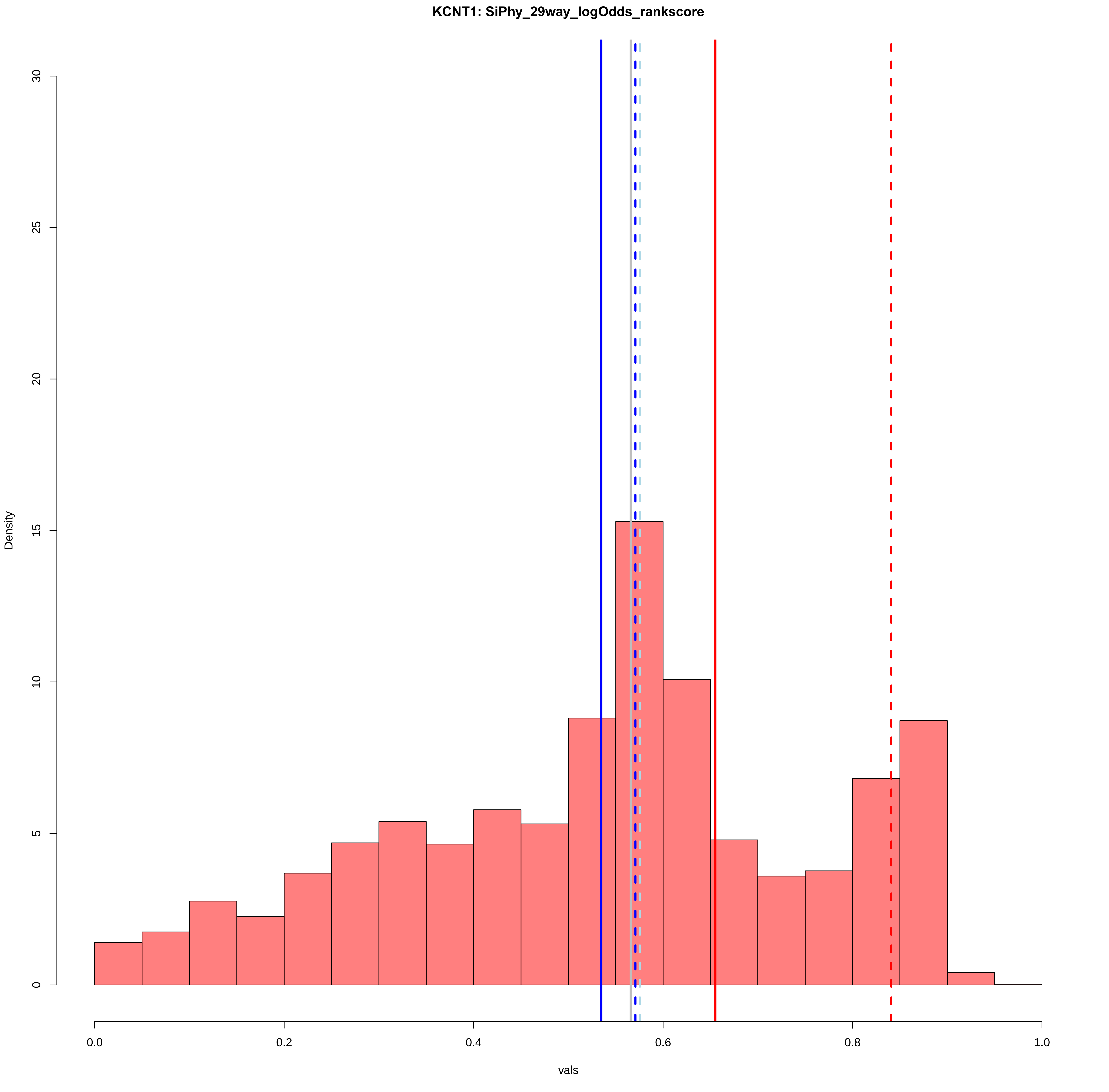

KCNT1: SiPhy_29way_logOdds_rankscore
Density
vals
0
5
10
15
20
25
30
0.0
0.2
0.4
0.6
0.8
1.0

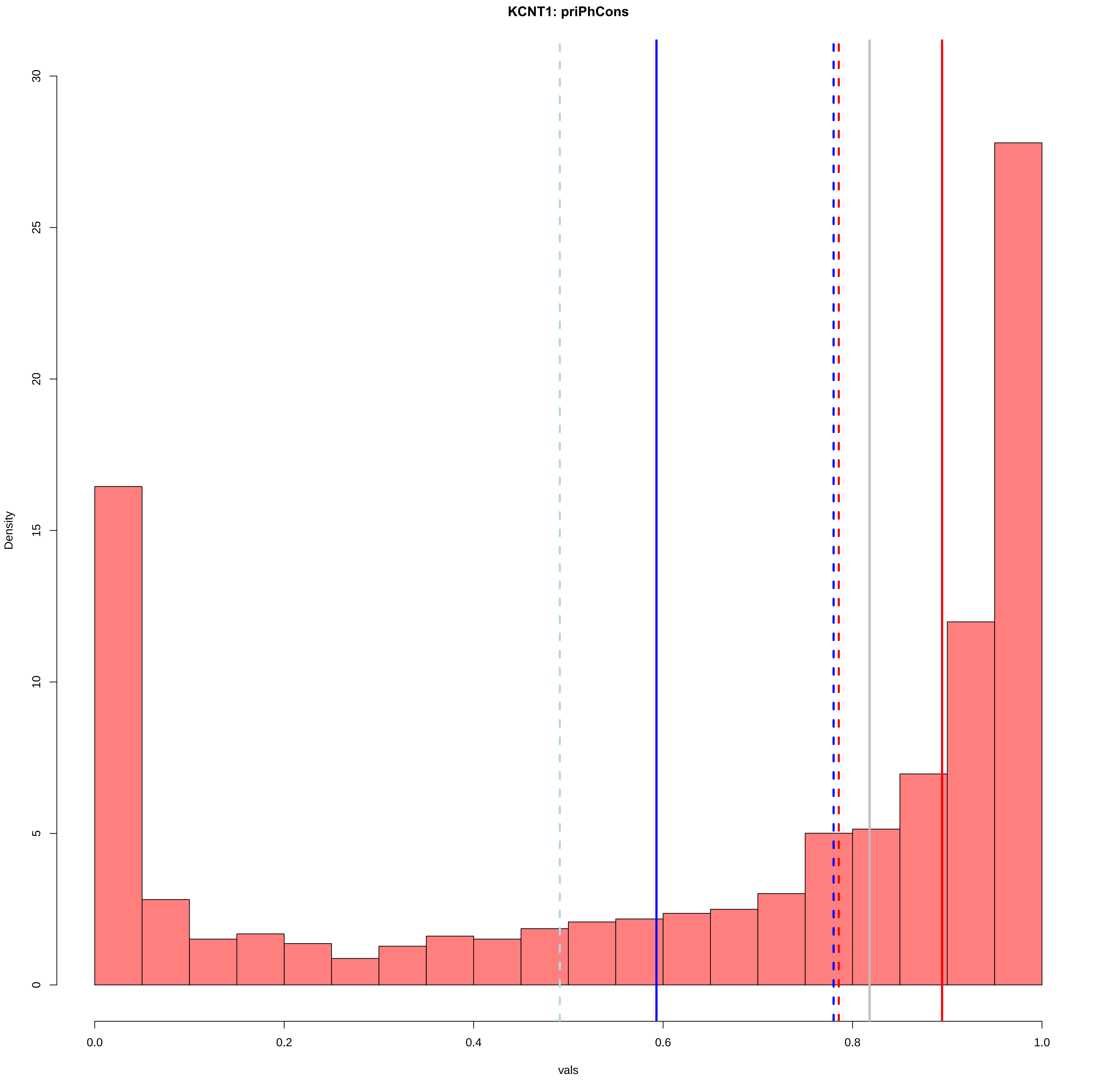
KCNT1: priPhCons
Density
vals
0
5
10
15
20
25
30
0.0
0.2
0.4
0.6
0.8
1.0

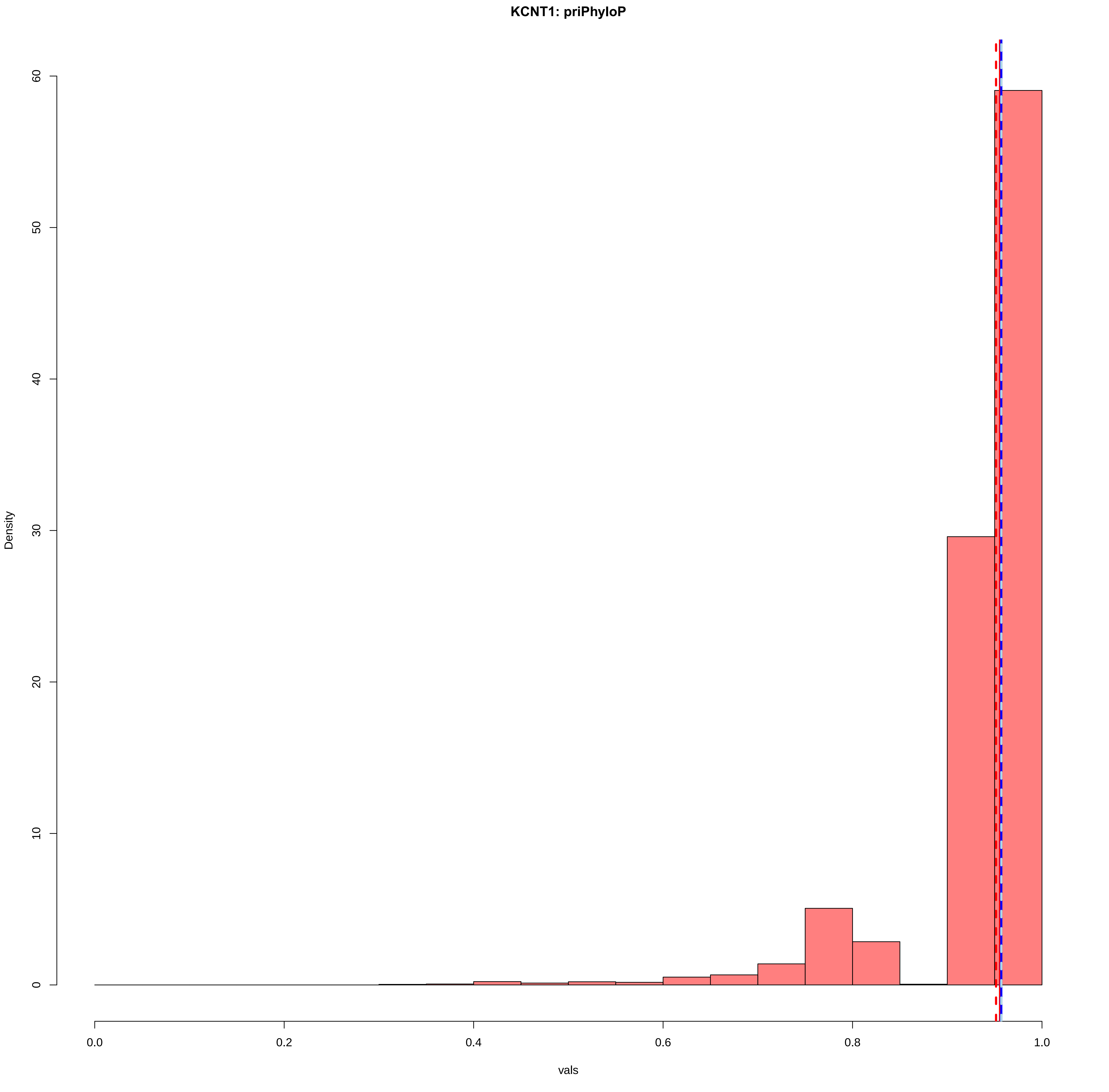

KCNT1: priPhyloP
Density
vals
0
10
20
30
40
50
60
0.0
0.2
0.4
0.6
0.8
1.0

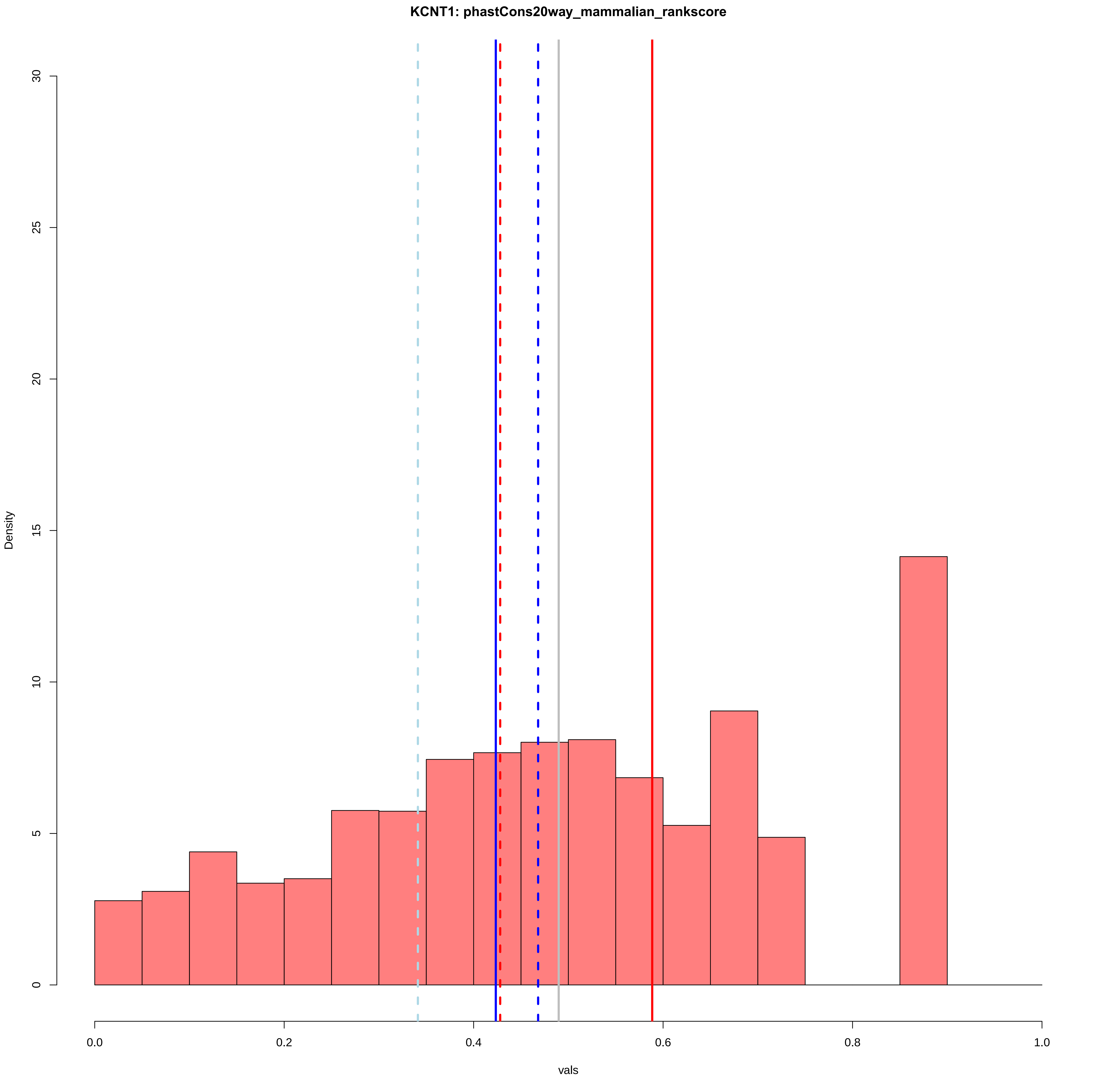

KCNT1: phastCons20way_mammalian_rankscore
Density
vals
0
5
10
15
20
25
30
0.0
0.2
0.4
0.6
0.8
1.0

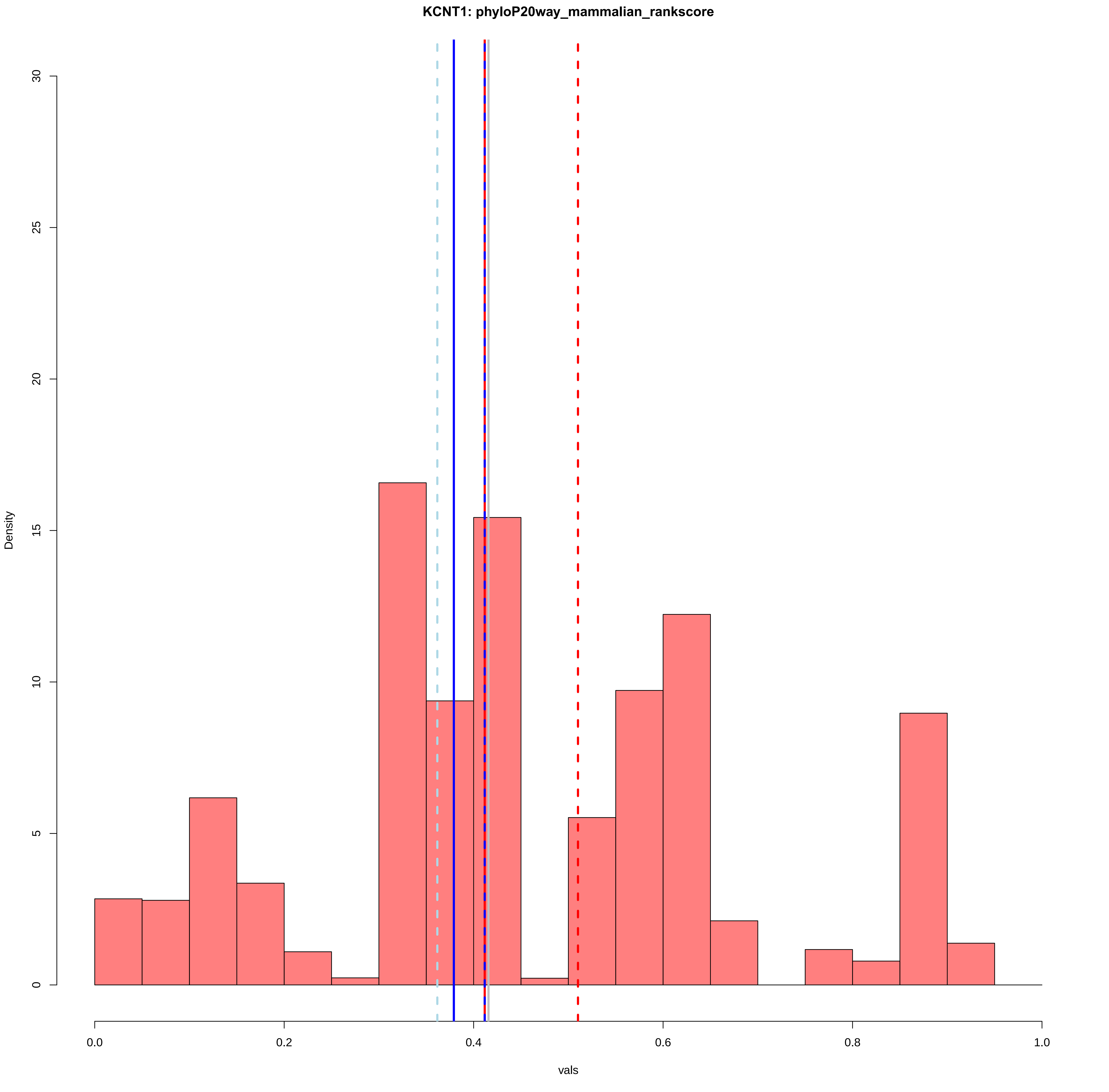
KCNT1: phyloP20way_mammalian_rankscore
Density
vals
0
5
10
15
20
25
30
0.0
0.2
0.4
0.6
0.8
1.0

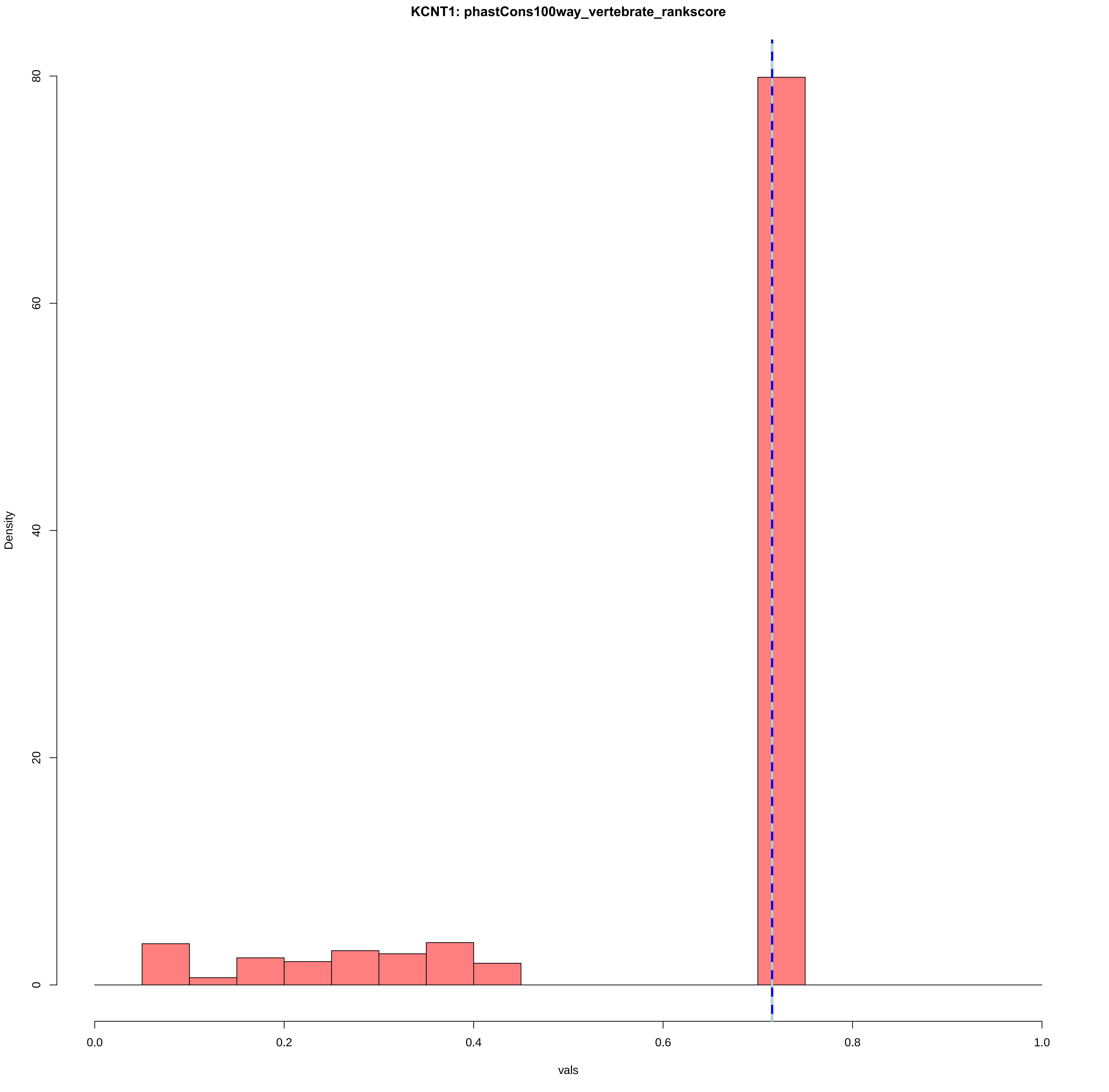
KCNT1: phastCons100way_vertebrate_rankscore
Density
vals
0
20
40
60
80
0.0
0.2
0.4
0.6
0.8
1.0

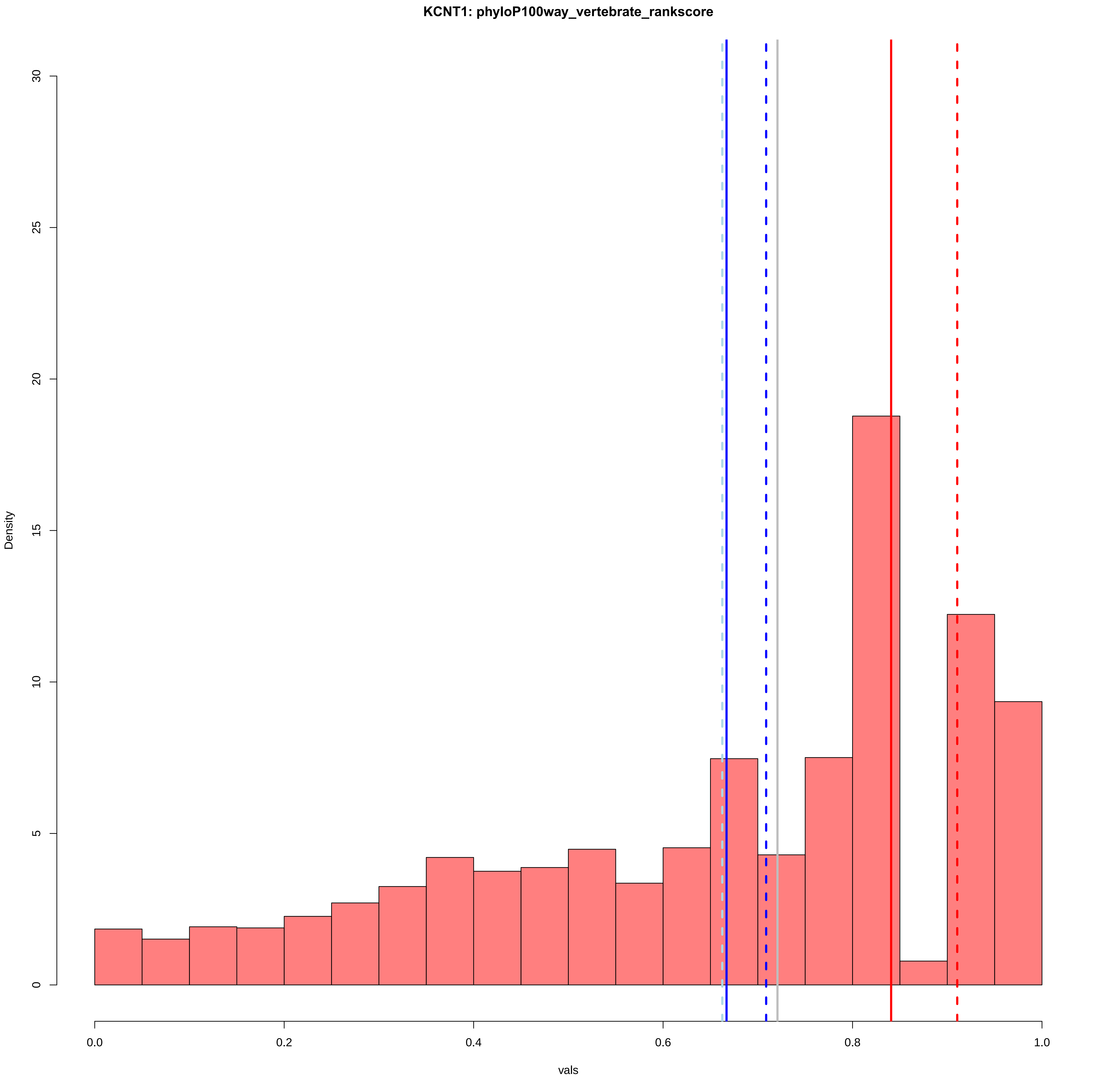

KCNT1: phyloP100way_vertebrate_rankscore
Density
vals
0
5
10
15
20
25
30
0.0
0.2
0.4
0.6
0.8
1.0

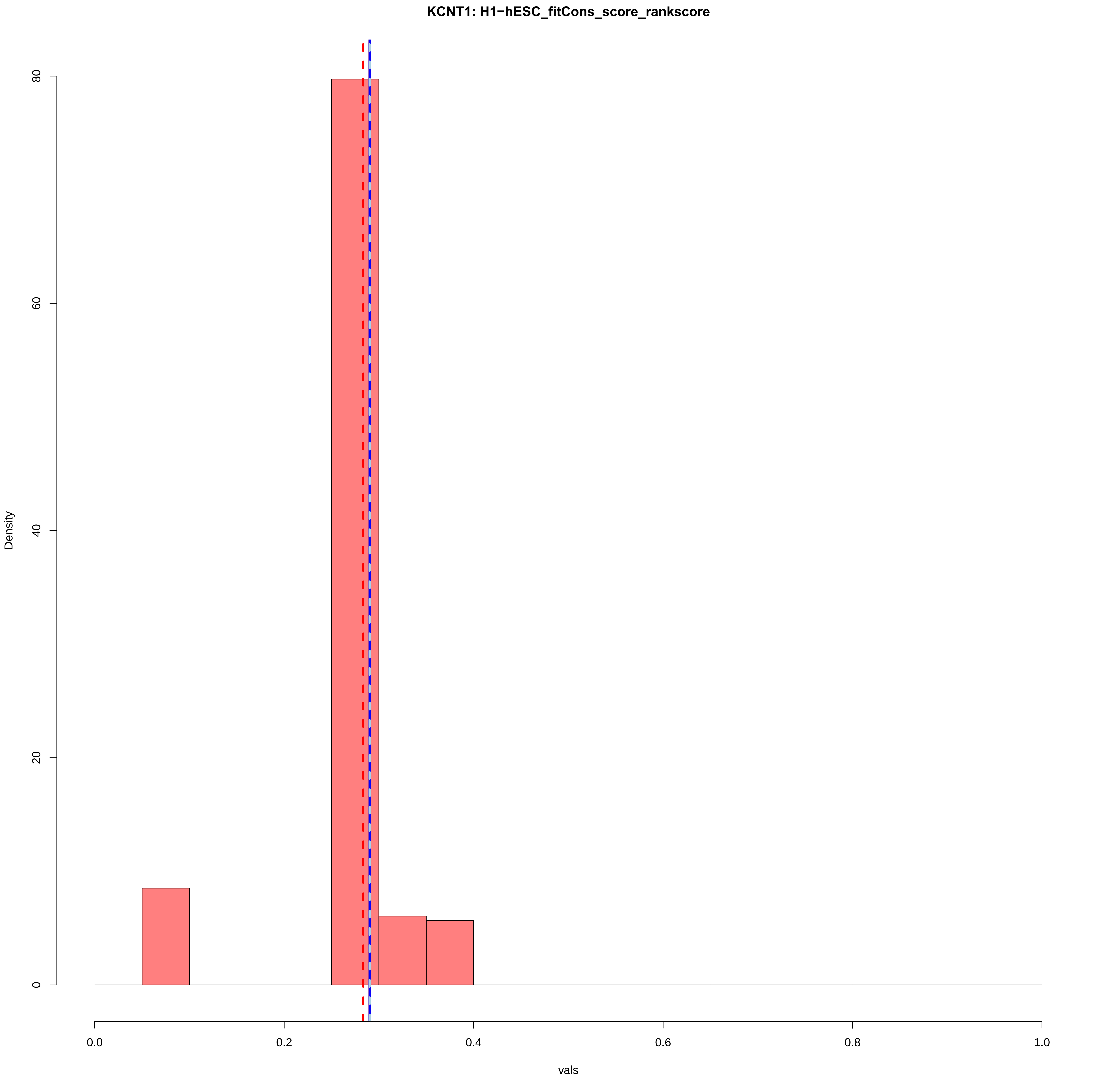
KCNT1: H1−hESC_fitCons_score_rankscore
Density
vals
0
20
40
60
80
0.0
0.2
0.4
0.6
0.8
1.0

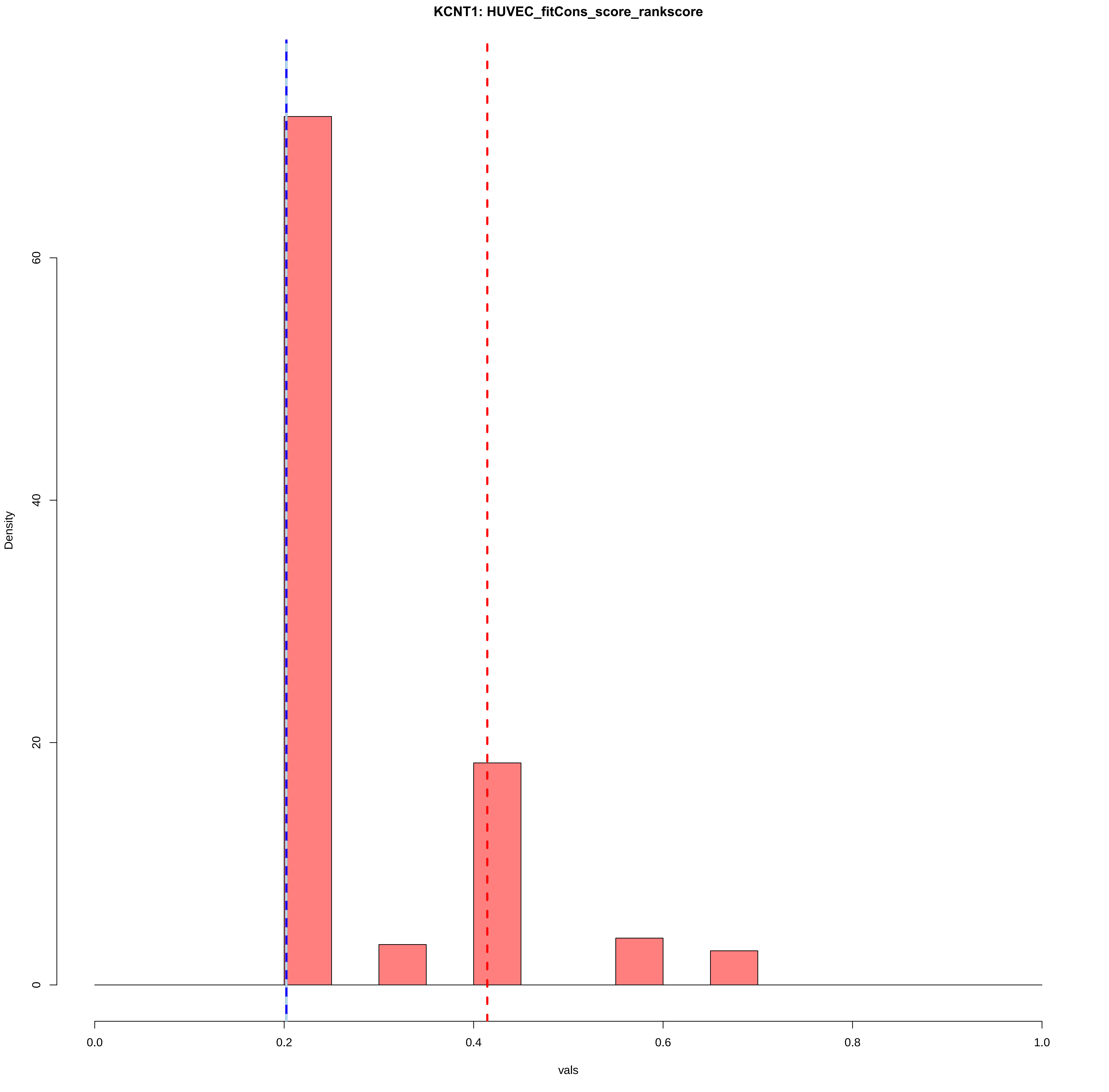

KCNT1: HUVEC_fitCons_score_rankscore
Density
0
20
40
60
0.0
0.2
0.4
0.6
0.8
1.0
vals

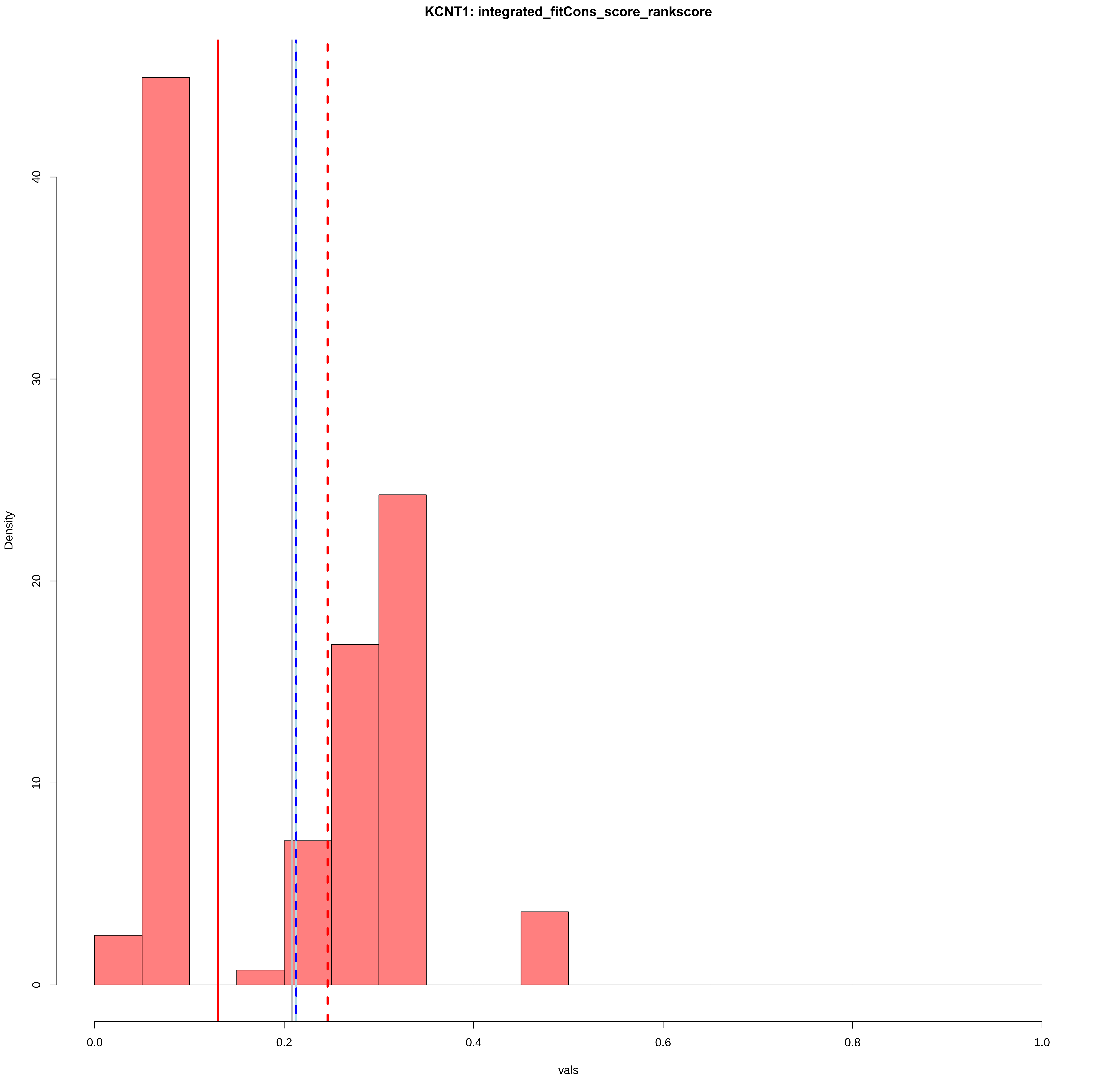
KCNT1: integrated_fitCons_score_rankscore
Density
0
10
20
30
40
0.0
0.2
0.4
0.6
0.8
1.0
vals

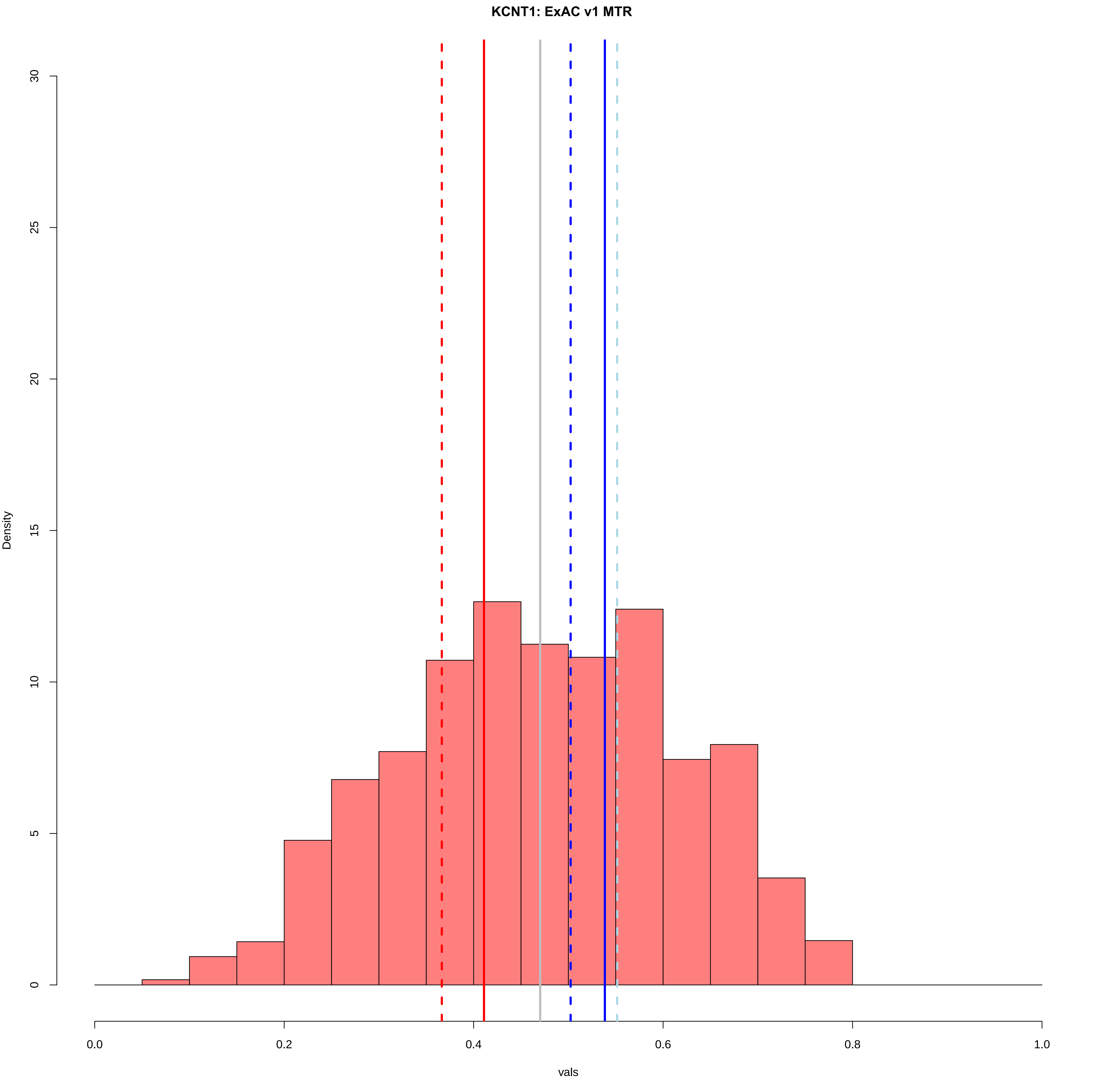

KCNT1: ExAC v1 MTR
Density
0
5
10
15
20
25
30
0.0
0.2
0.4
0.6
0.8
1.0
vals

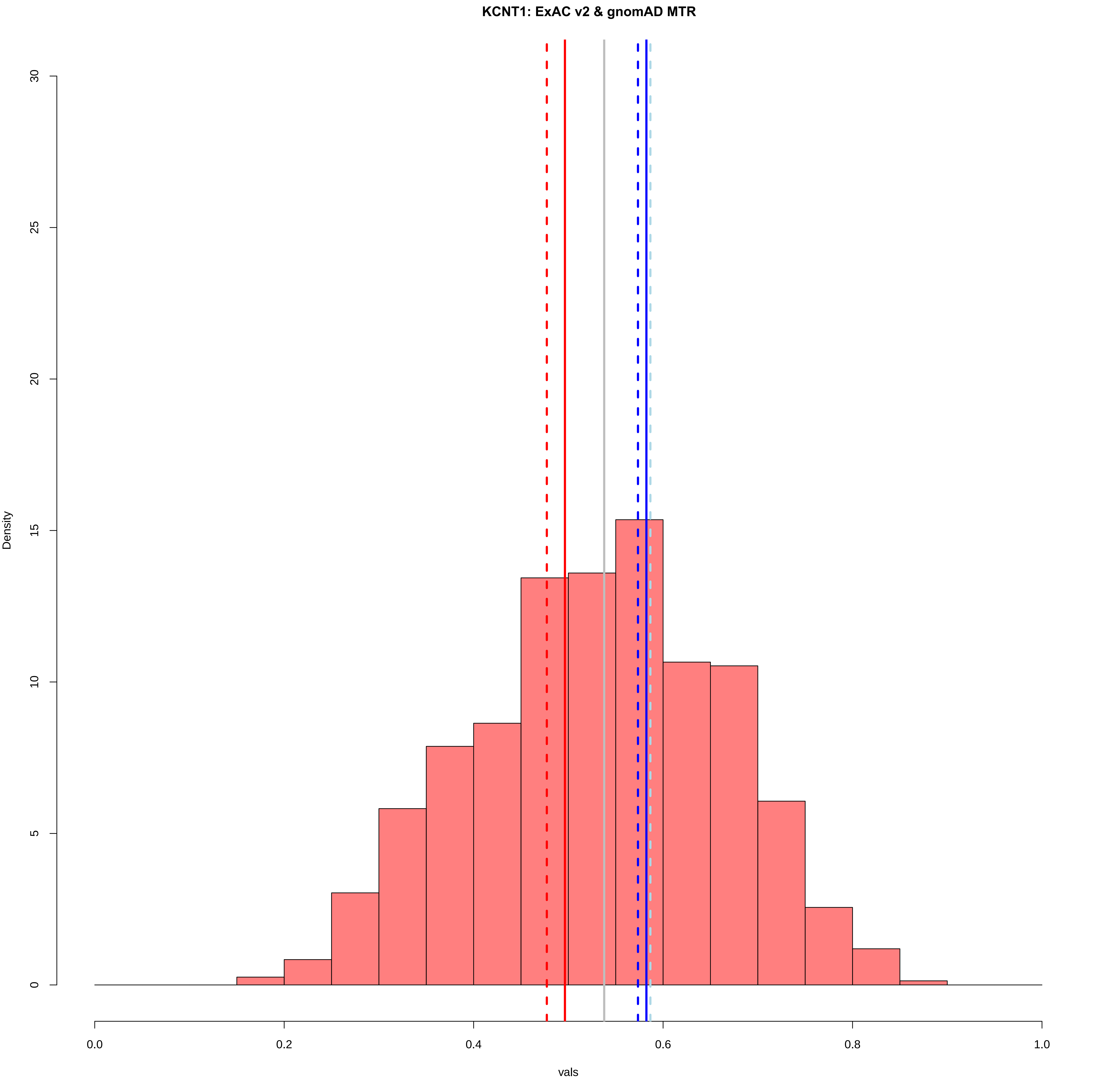

KCNT1: ExAC v2 & gnomAD MTR
Density
vals
0
5
10
15
20
25
30
0.0
0.2
0.4
0.6
0.8
1.0

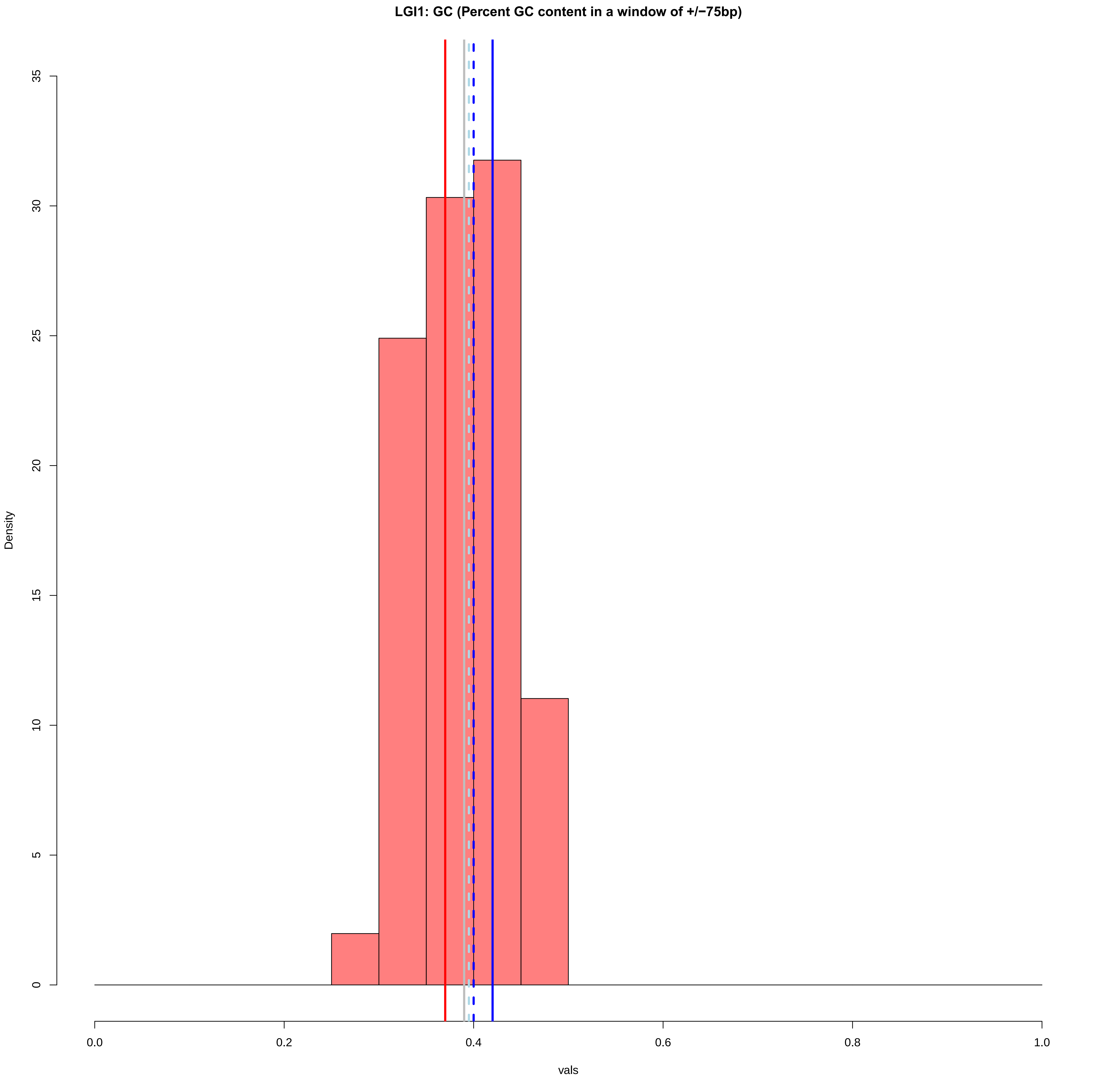

LGI1: GC (Percent GC content in a window of +/−75bp)
Density
0
5
10
15
20
25
30
35
0.0
0.2
0.4
0.6
0.8
1.0
vals

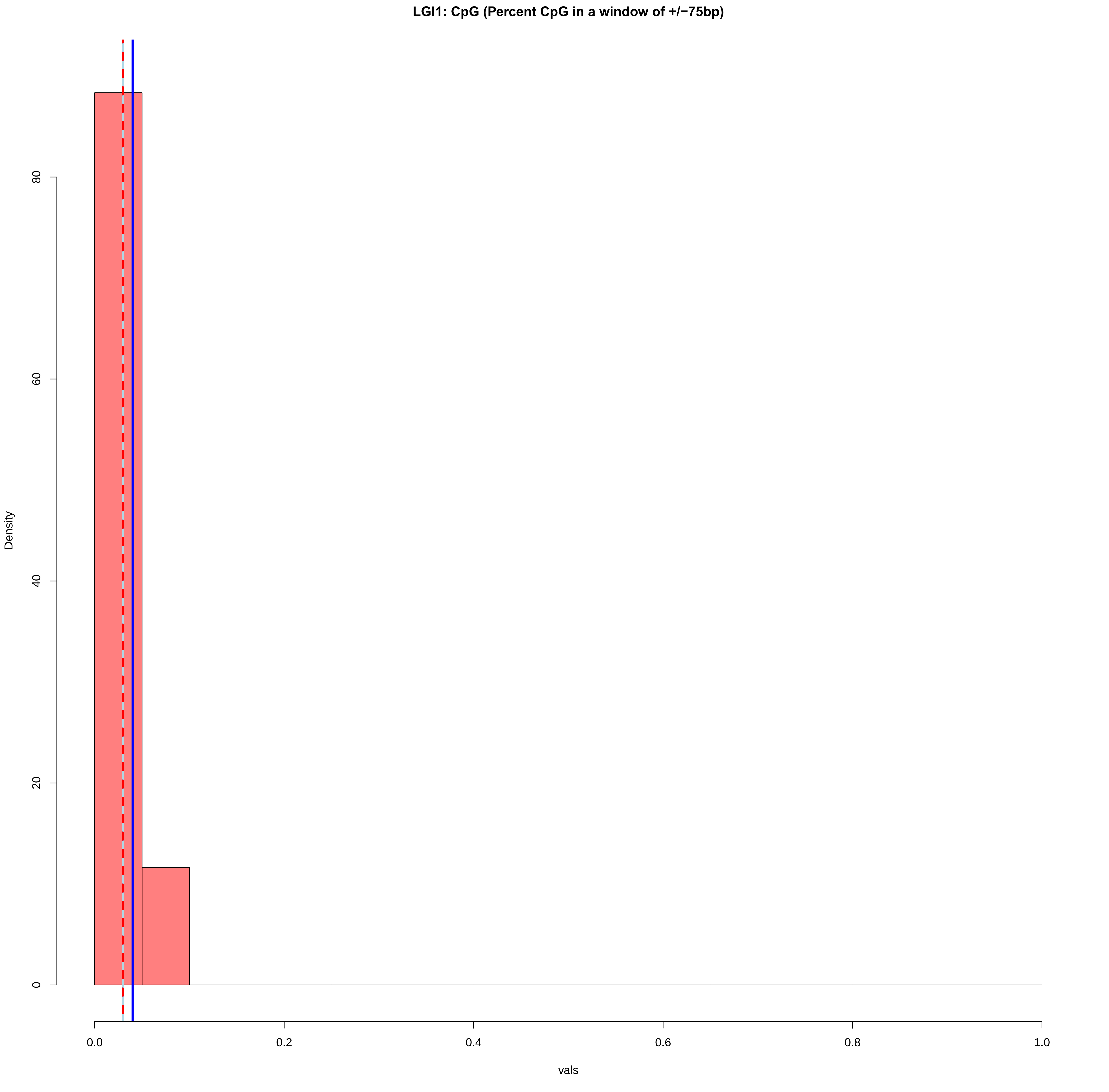
LGI1: CpG (Percent CpG in a window of +/−75bp)
Density
0
20
40
60
80
0.0
0.2
0.4
0.6
0.8
1.0
vals

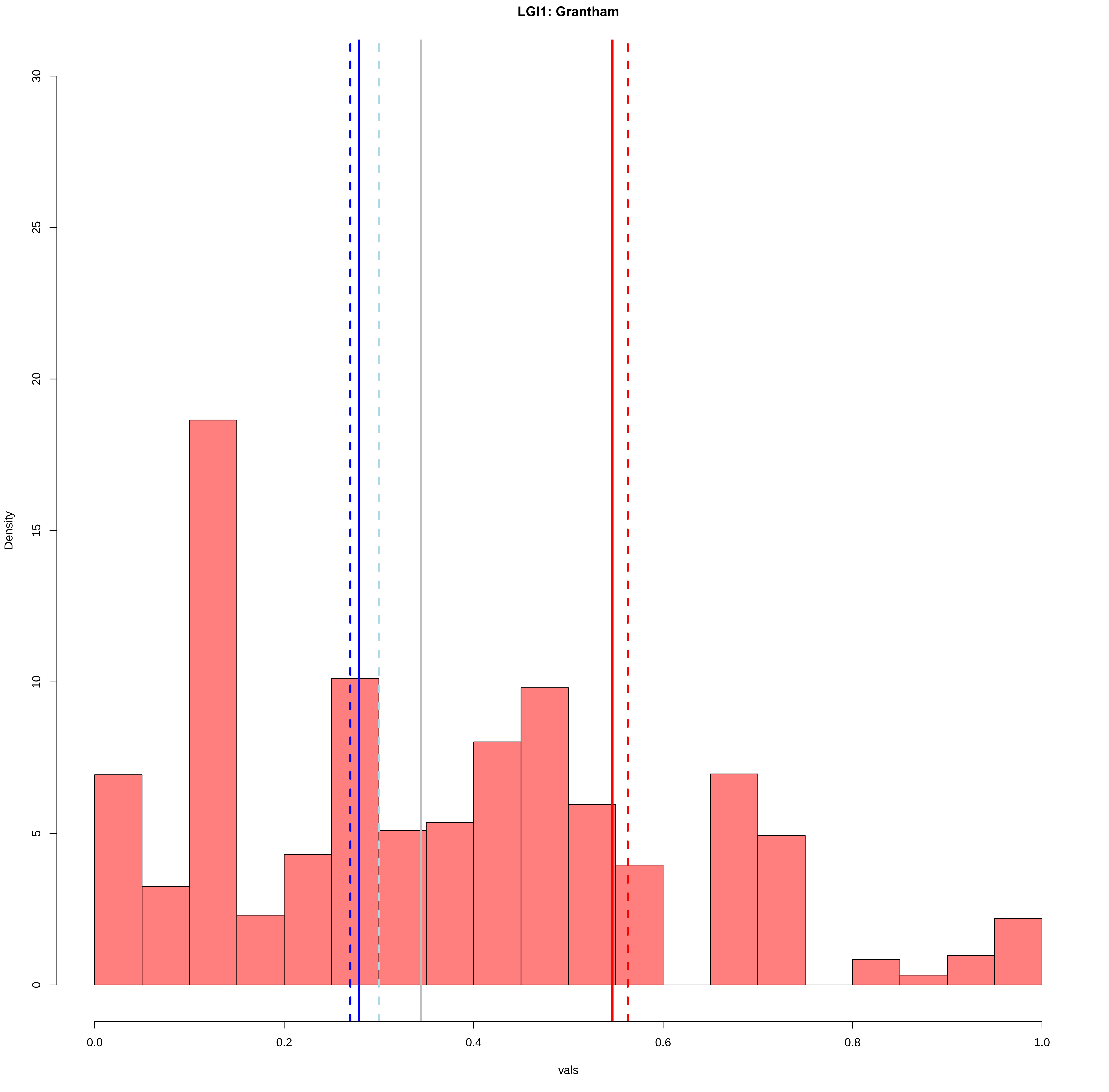
LGI1: Grantham
Density
vals
0
5
10
15
20
25
30
0.0
0.2
0.4
0.6
0.8
1.0

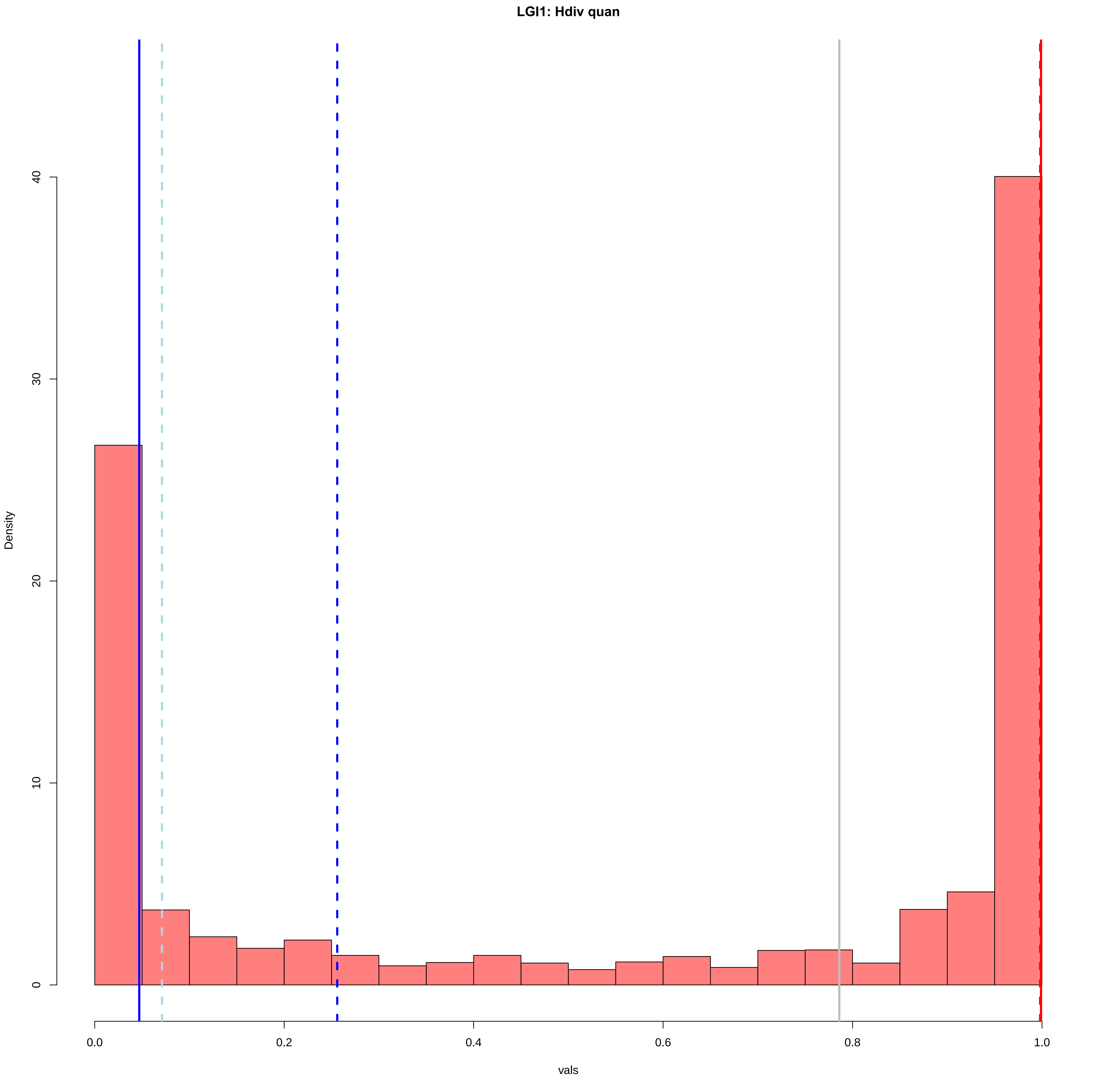

LGI1: Hdiv quan
Density
0
10
20
30
40
0.0
0.2
0.4
0.6
0.8
1.0
vals

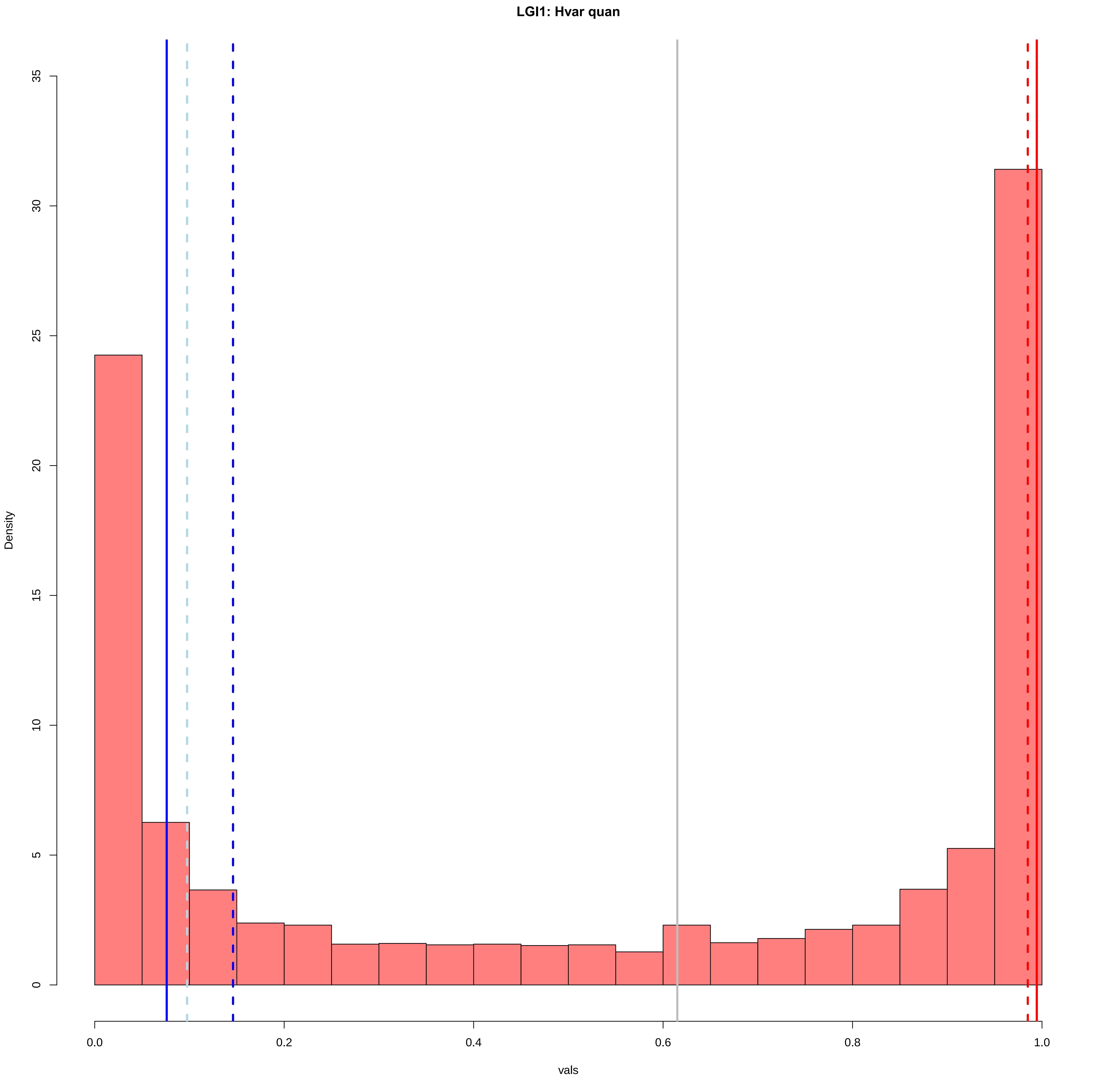
LGI1: Hvar quan
Density
vals
0
5
10
15
20
25
30
35
0.0
0.2
0.4
0.6
0.8
1.0

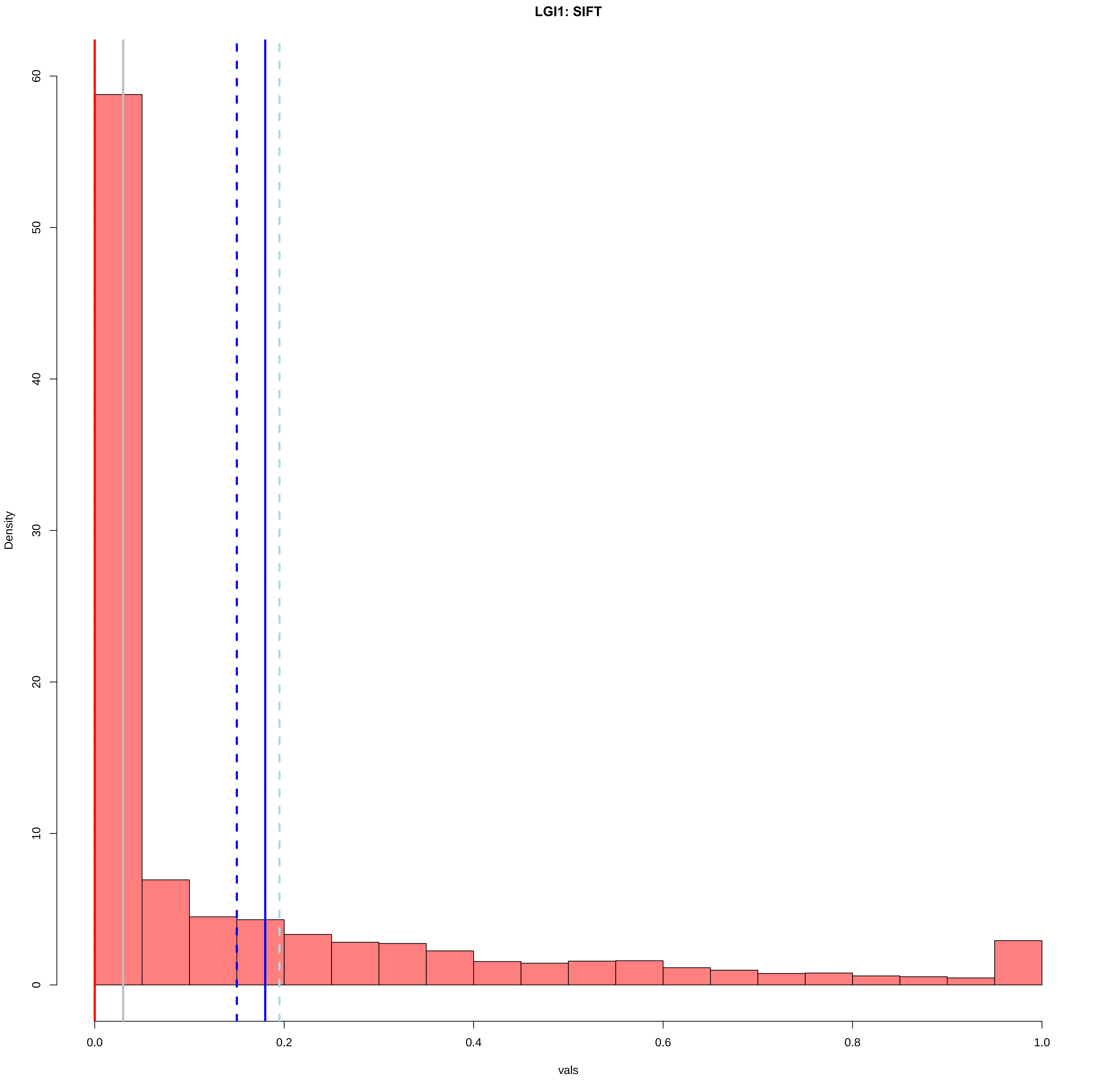

LGI1: SIFT
Density
0
10
20
30
40
50
60
0.0
0.2
0.4
0.6
0.8
1.0
vals

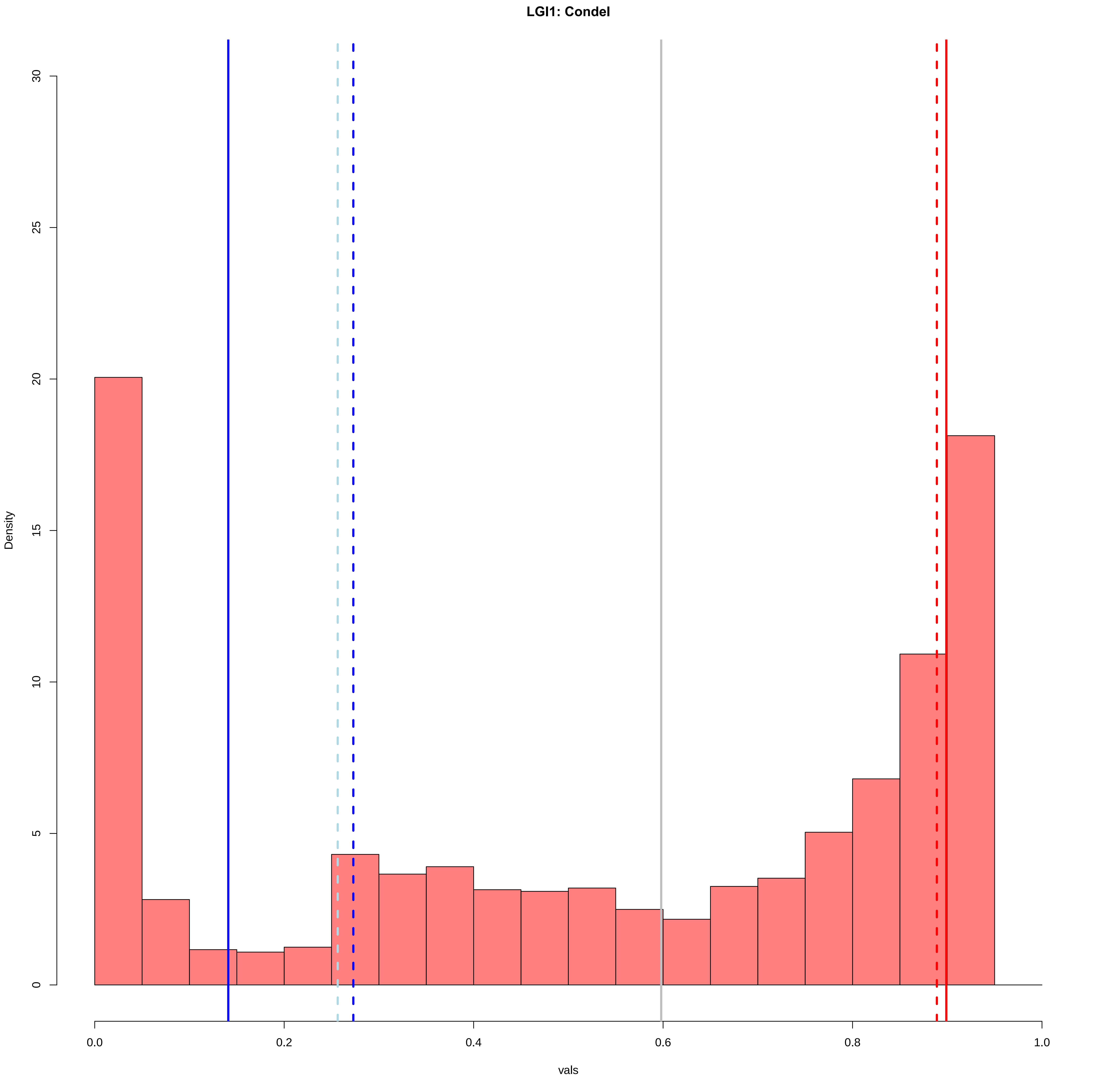

LGI1: Condel
Density
0
5
10
15
20
25
30
0.0
0.2
0.4
0.6
0.8
1.0
vals

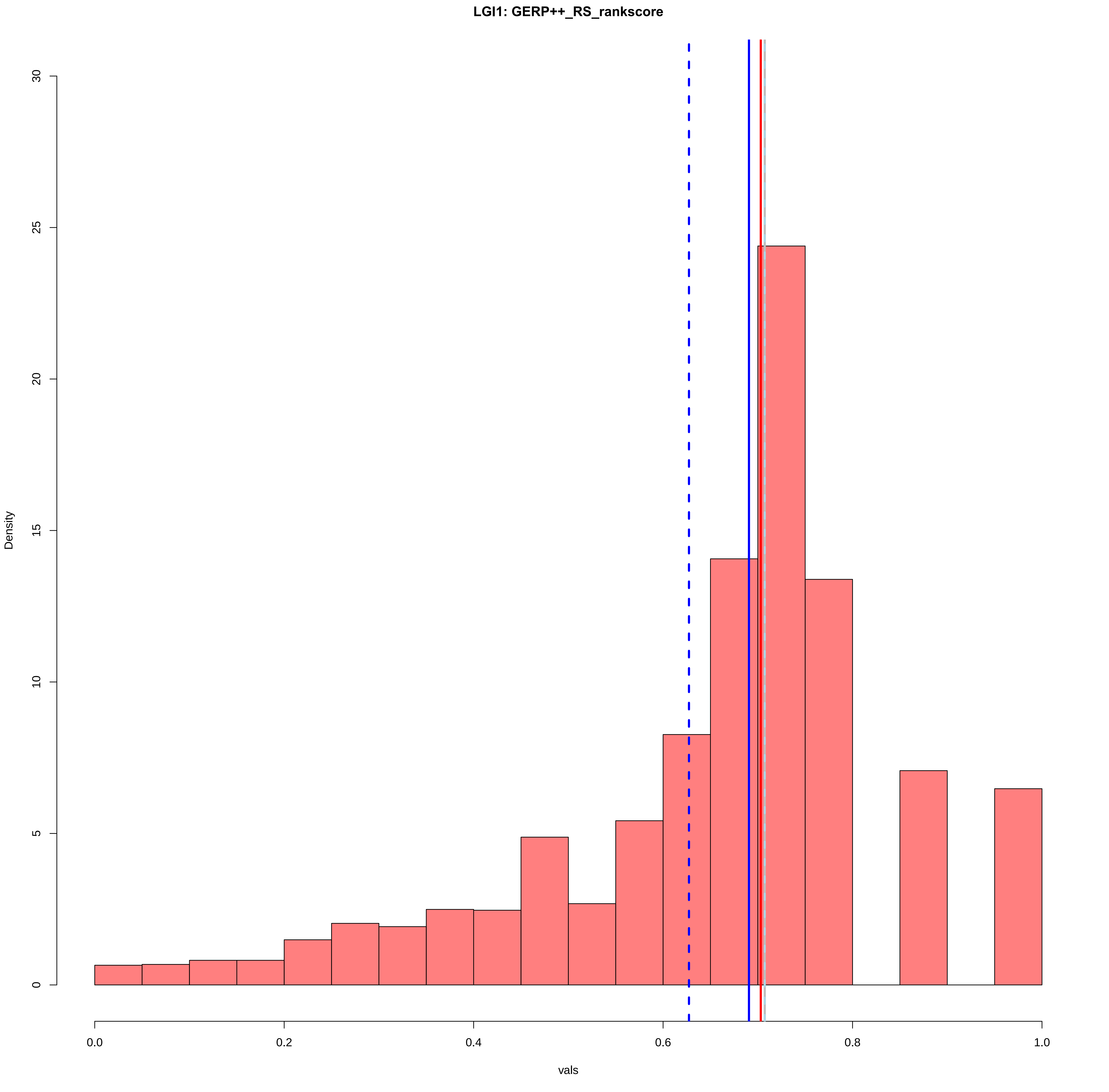
LGI1: GERP++_RS_rankscore
Density
vals
0
5
10
15
20
25
30
0.0
0.2
0.4
0.6
0.8
1.0

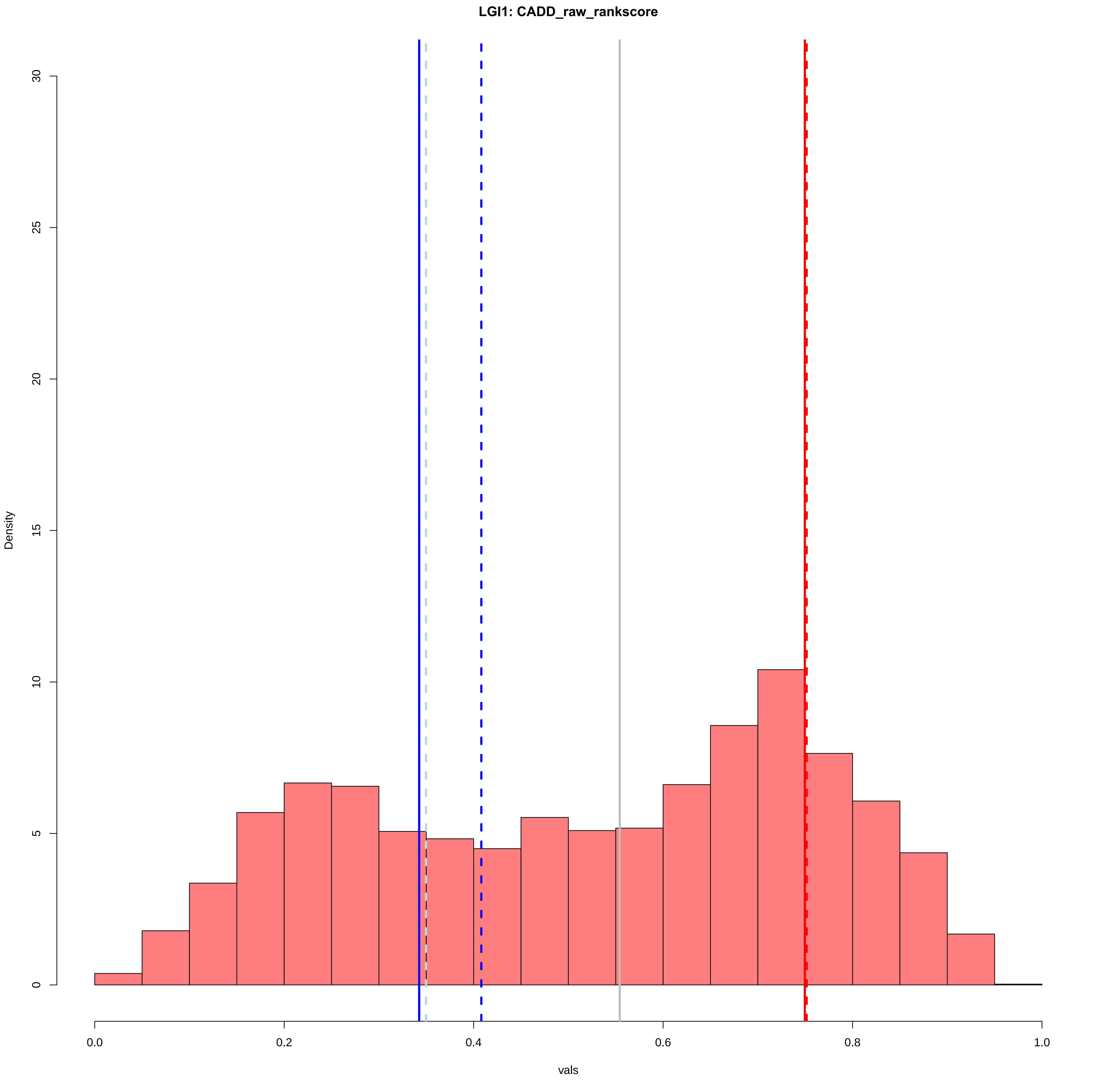

LGI1: CADD_raw_rankscore
Density
vals
0
5
10
15
20
25
30
0.0
0.2
0.4
0.6
0.8
1.0

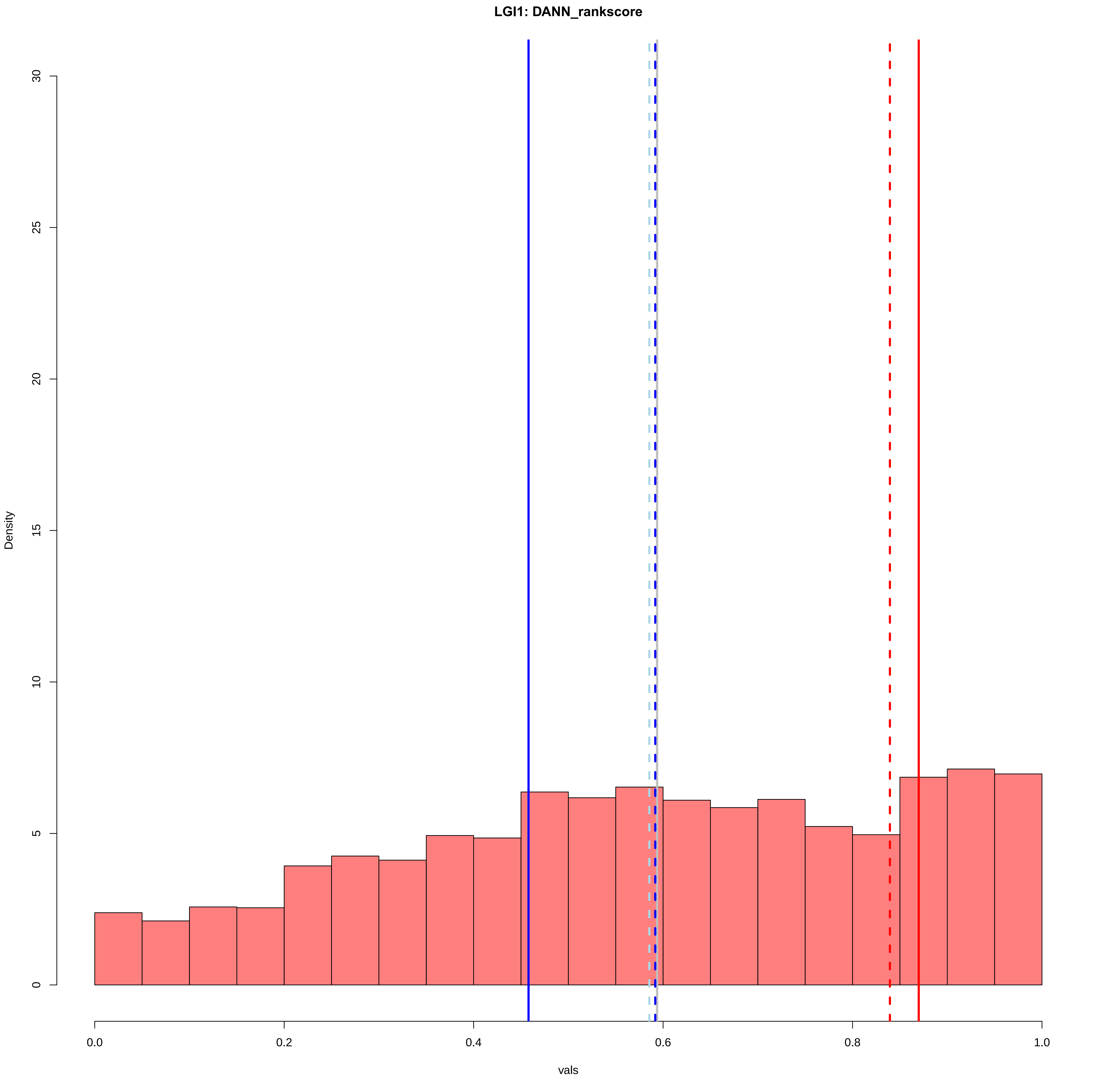

LGI1: DANN_rankscore
Density
vals
0
5
10
15
20
25
30
0.0
0.2
0.4
0.6
0.8
1.0

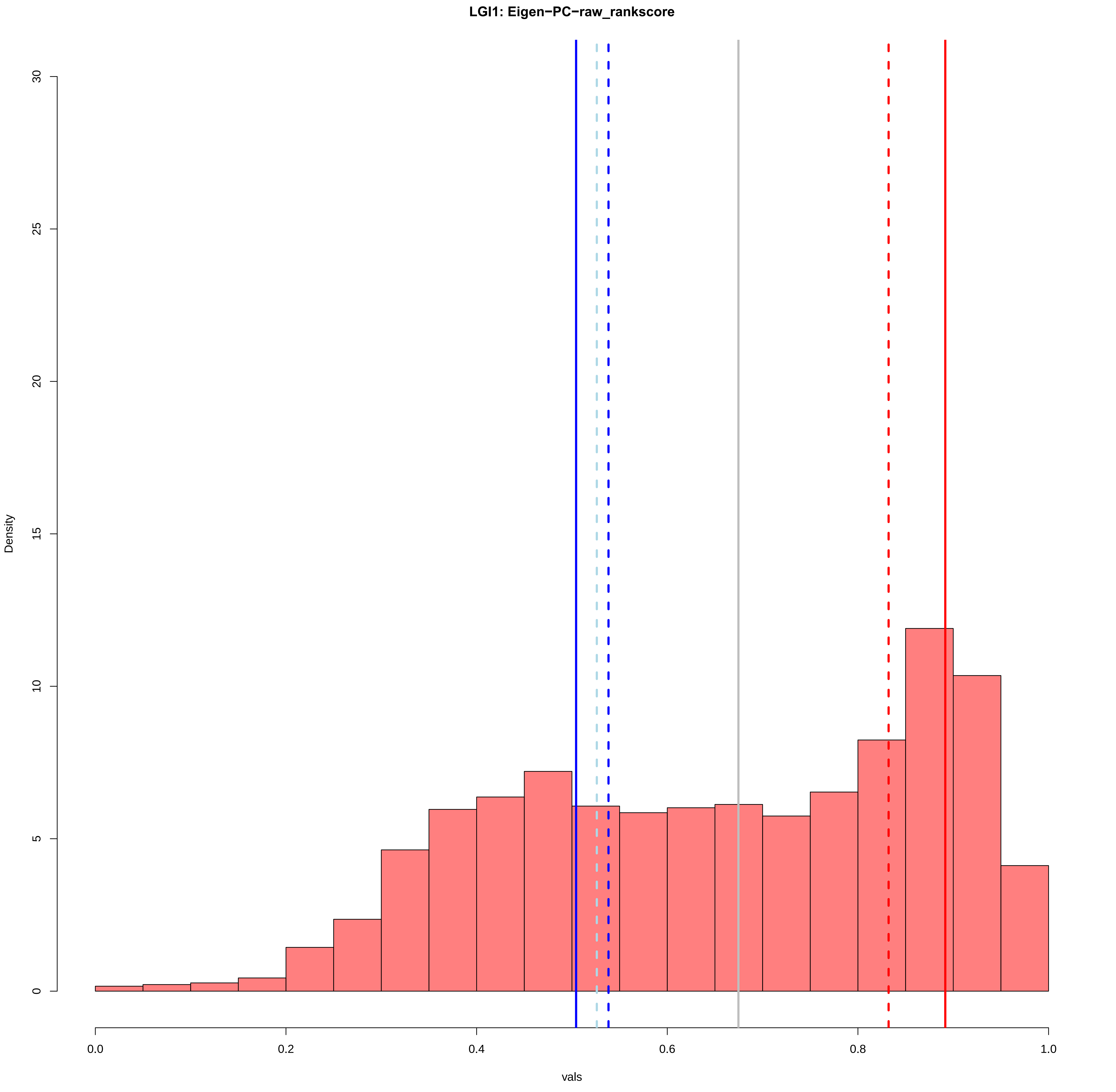

LGI1: Eigen−PC−raw_rankscore
Density
vals
0
5
10
15
20
25
30
0.0
0.2
0.4
0.6
0.8
1.0

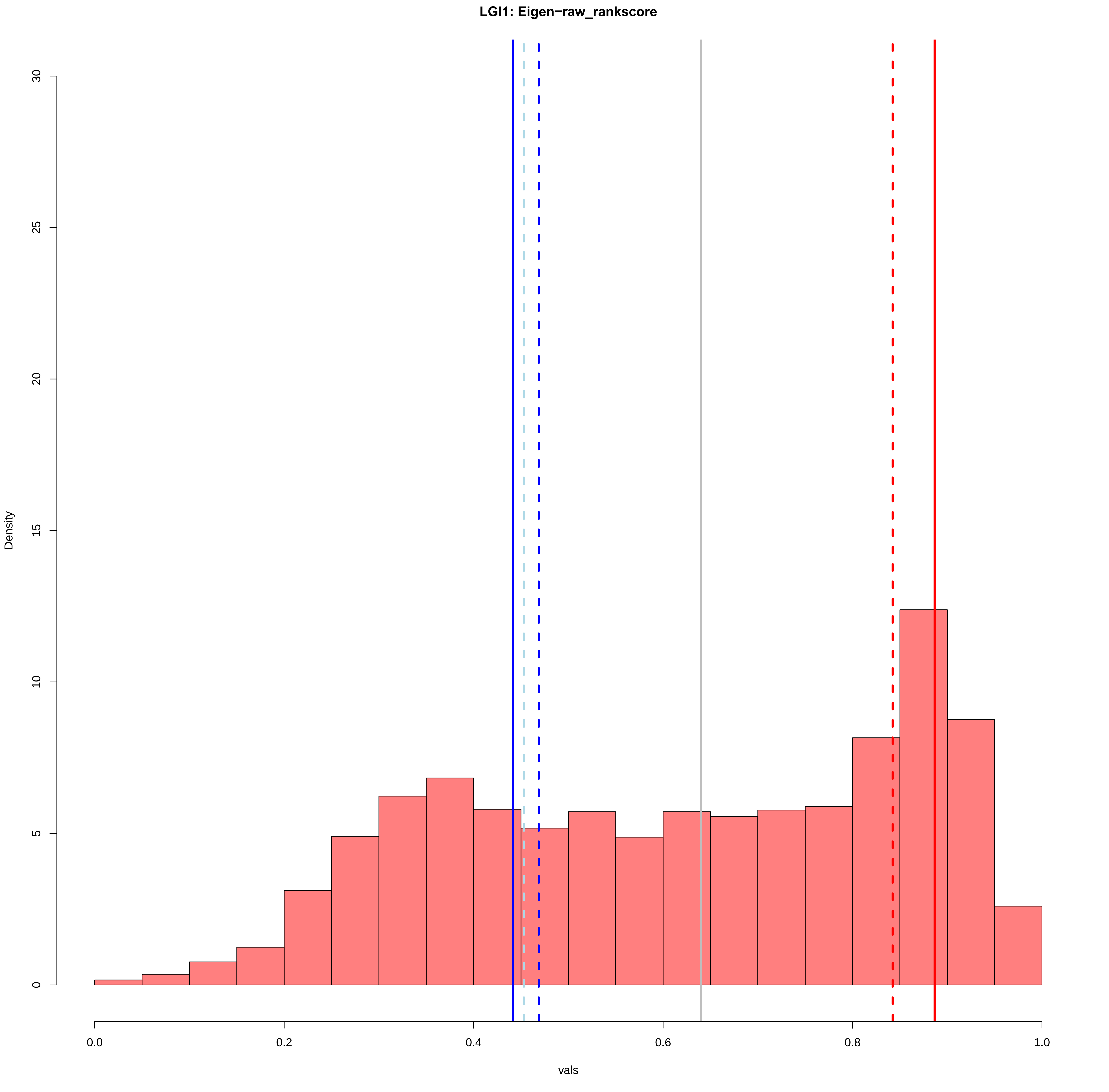
LGI1: Eigen−raw_rankscore
Density
vals
0
5
10
15
20
25
30
0.0
0.2
0.4
0.6
0.8
1.0

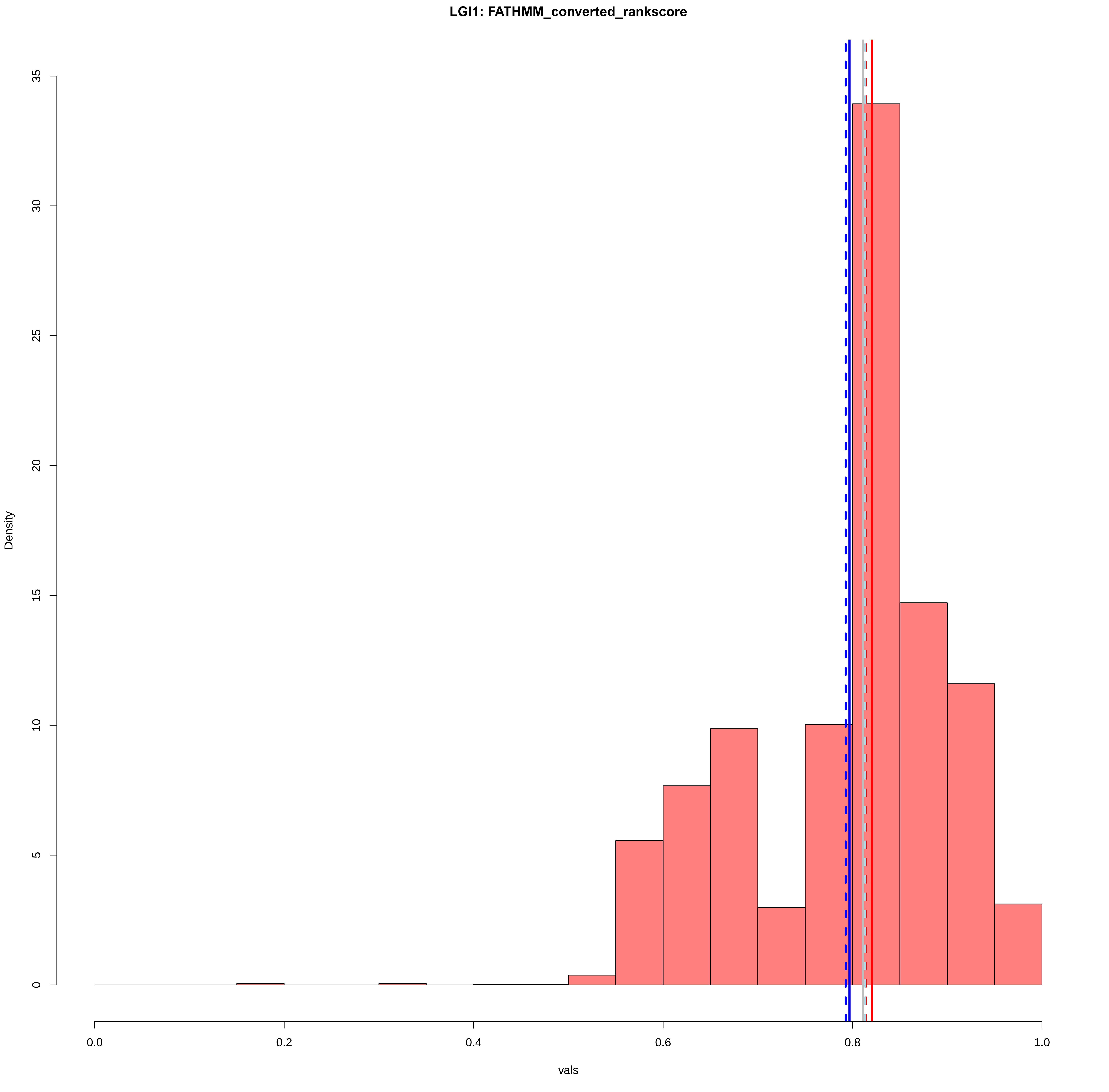
LGI1: FATHMM_converted_rankscore
Density
vals
0
5
10
15
20
25
30
35
0.0
0.2
0.4
0.6
0.8
1.0

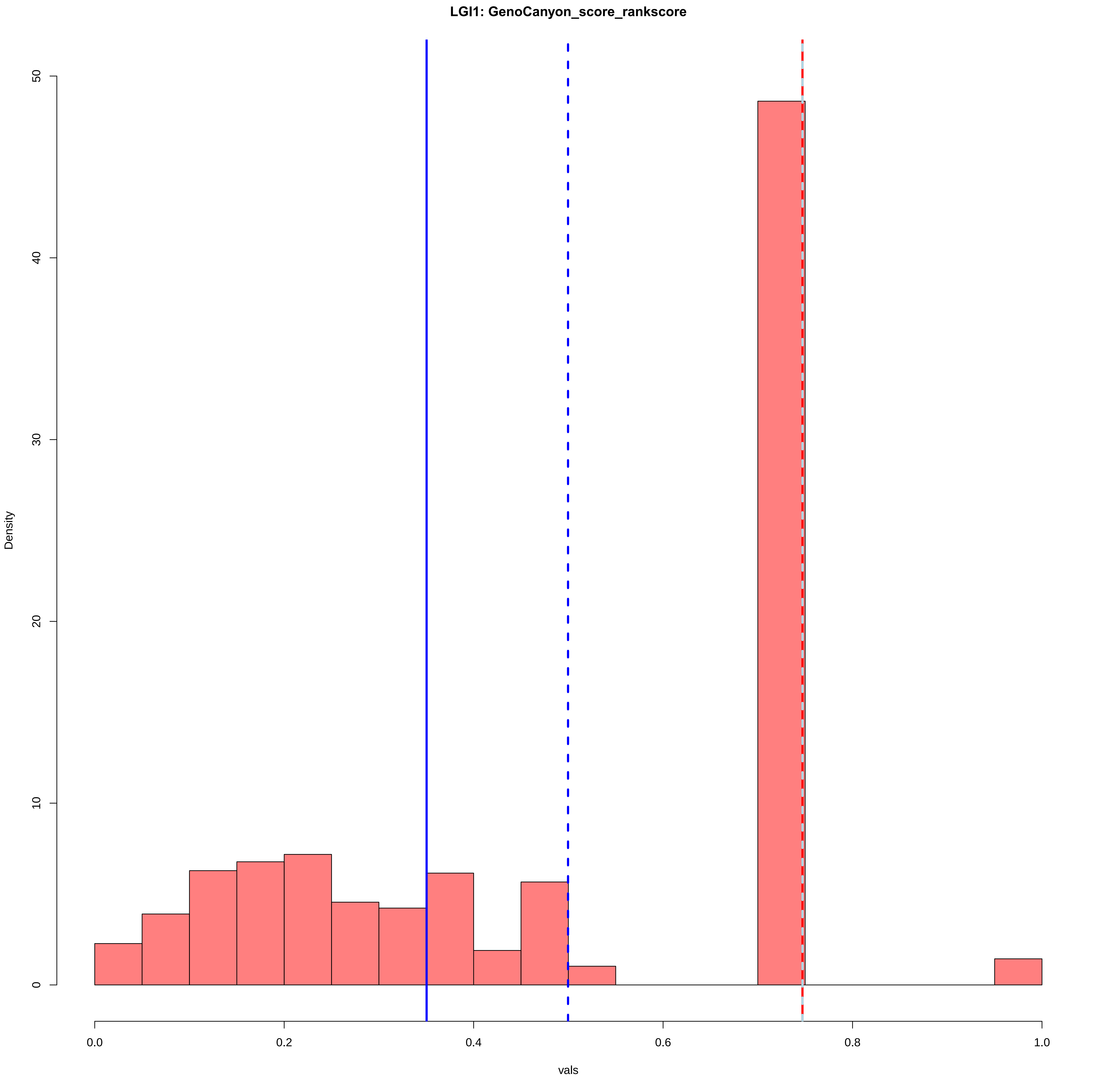
LGI1: GenoCanyon_score_rankscore
Density
vals
0
10
20
30
40
50
0.0
0.2
0.4
0.6
0.8
1.0

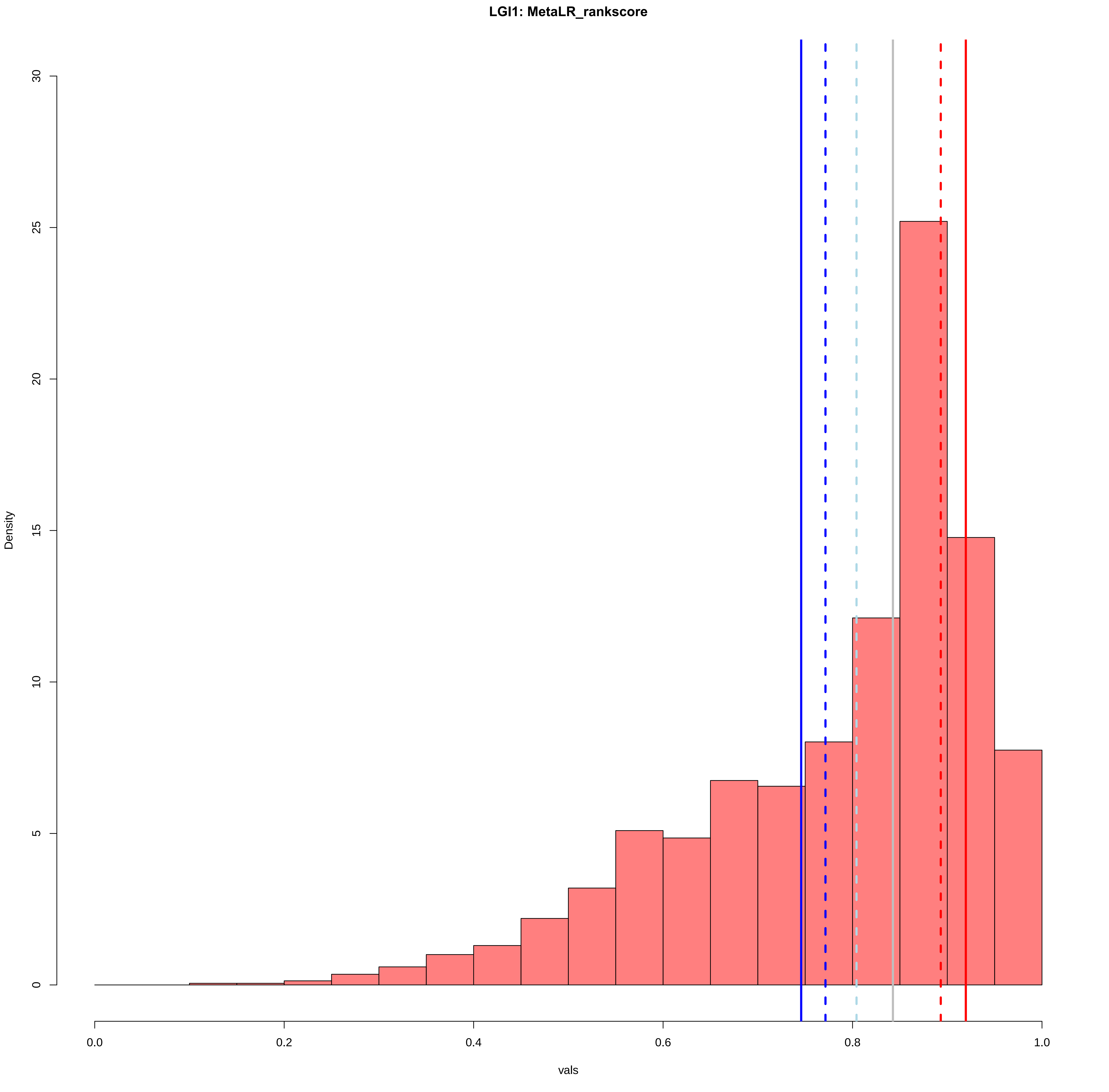
LGI1: MetaLR_rankscore
Density
vals
0
5
10
15
20
25
30
0.0
0.2
0.4
0.6
0.8
1.0

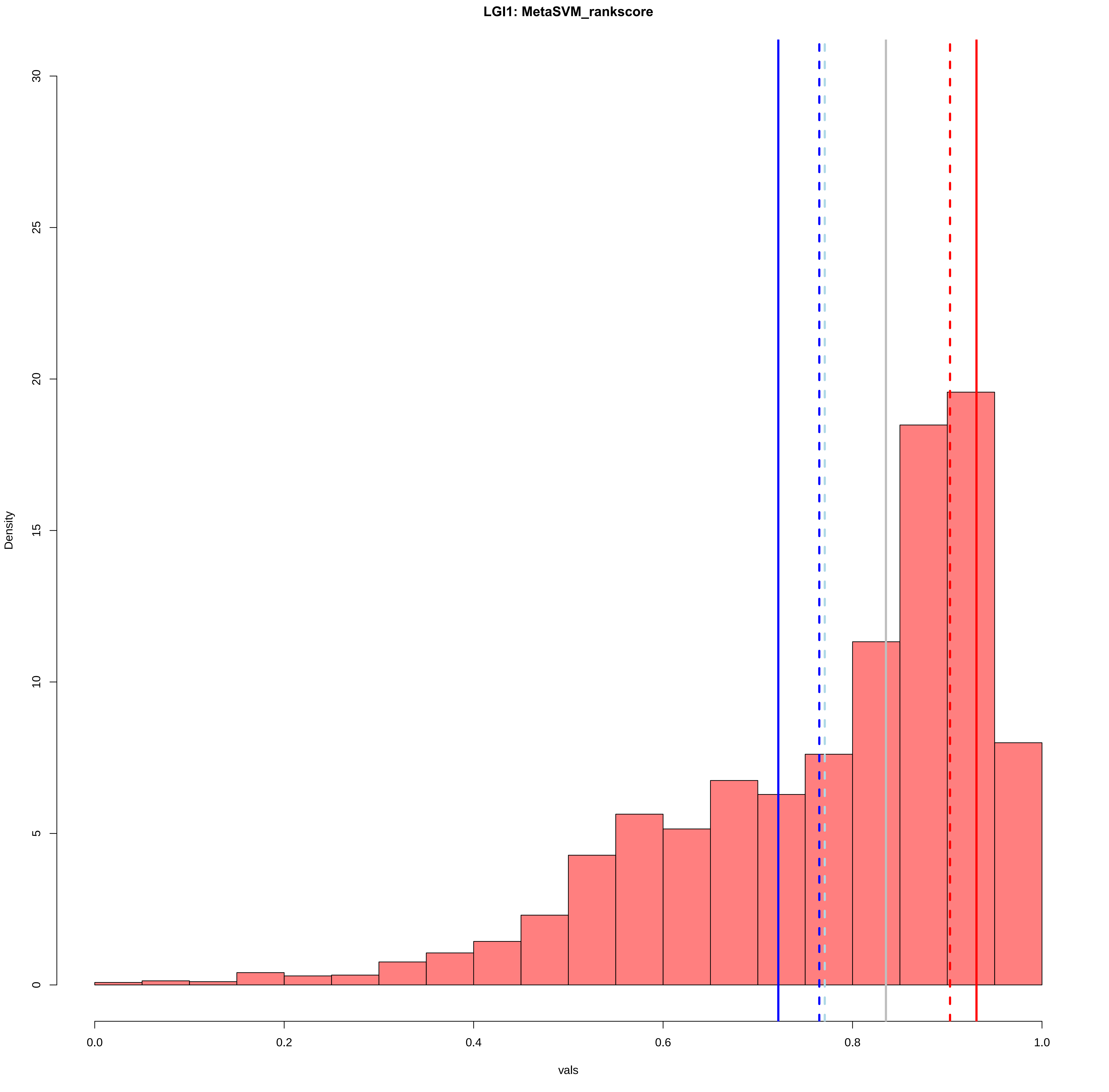

LGI1: MetaSVM_rankscore
Density
vals
0
5
10
15
20
25
30
0.0
0.2
0.4
0.6
0.8
1.0

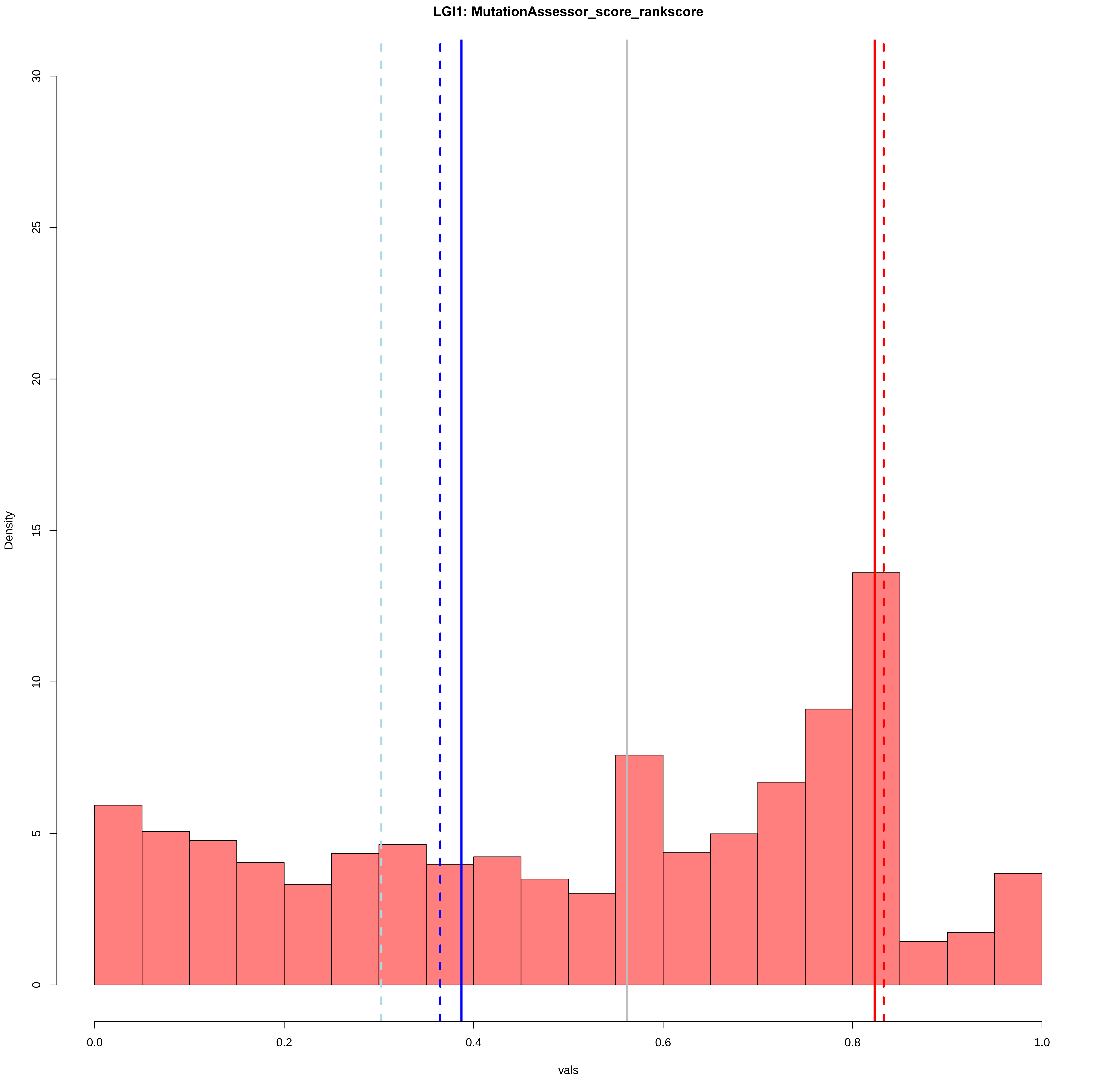

LGI1: MutationAssessor_score_rankscore
Density
vals
0
5
10
15
20
25
30
0.0
0.2
0.4
0.6
0.8
1.0

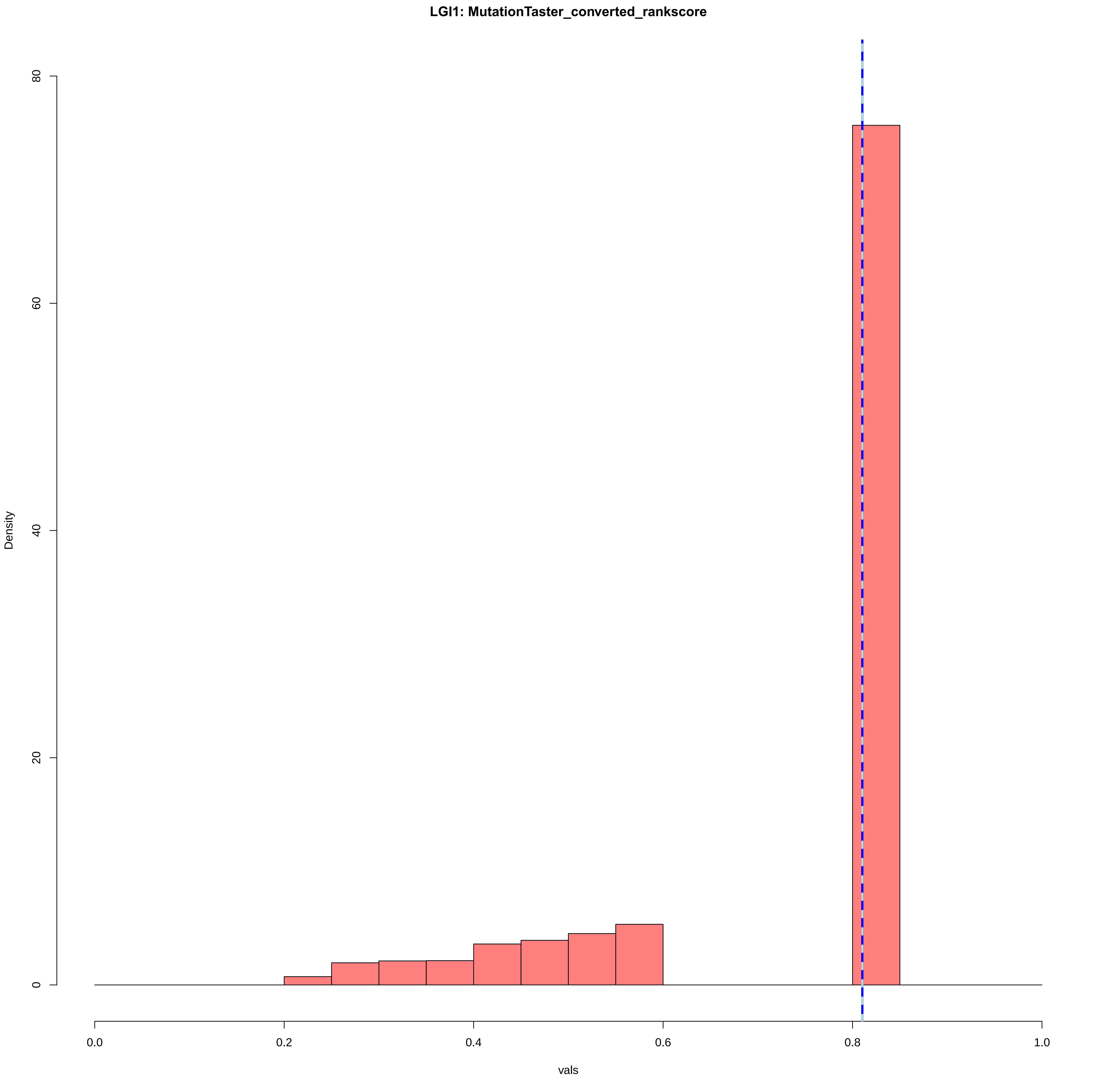
LGI1: MutationTaster_converted_rankscore
Density
0
20
40
60
80
0.0
0.2
0.4
0.6
0.8
1.0
vals

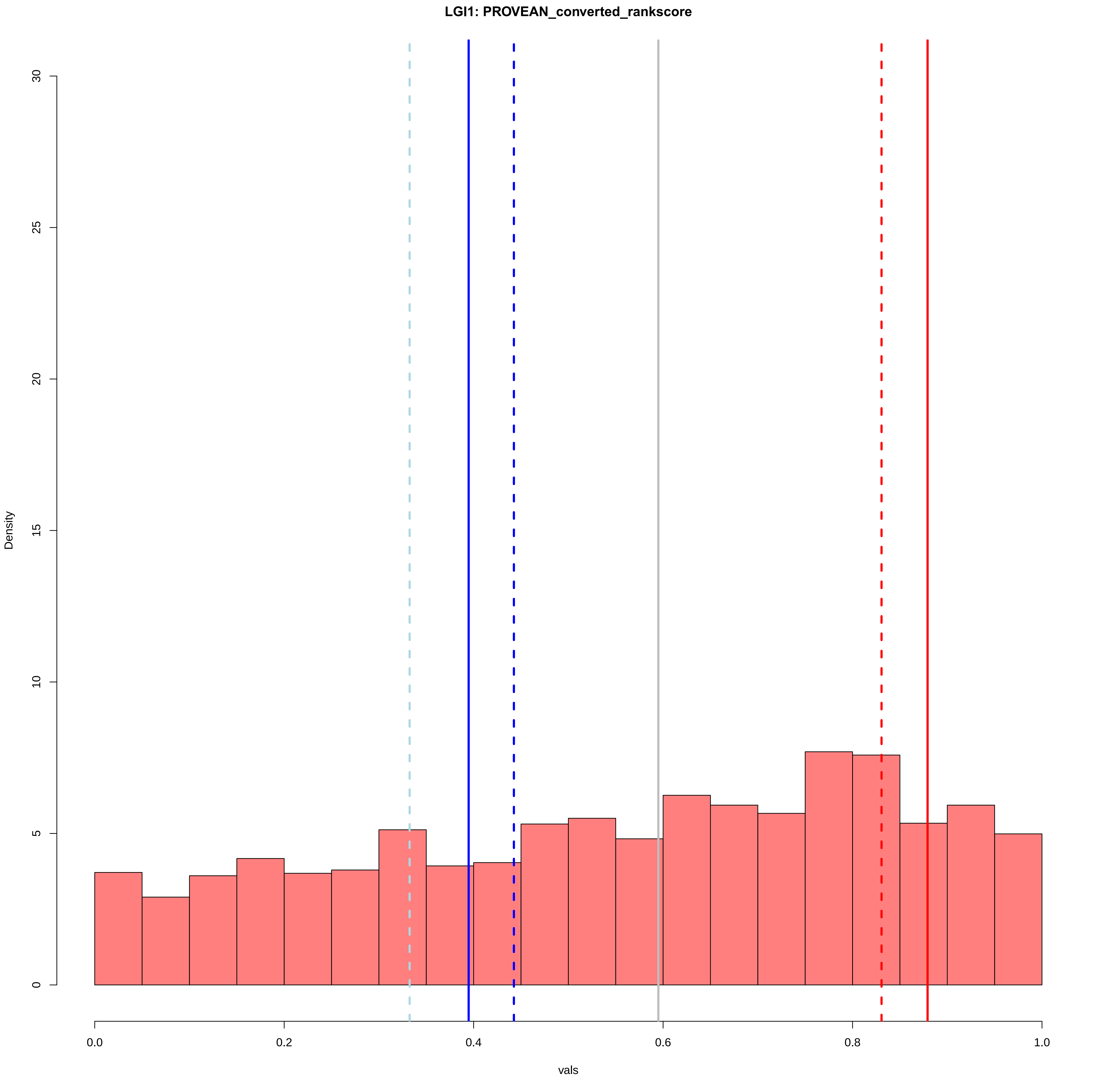
LGI1: PROVEAN_converted_rankscore
Density
vals
0
5
10
15
20
25
30
0.0
0.2
0.4
0.6
0.8
1.0

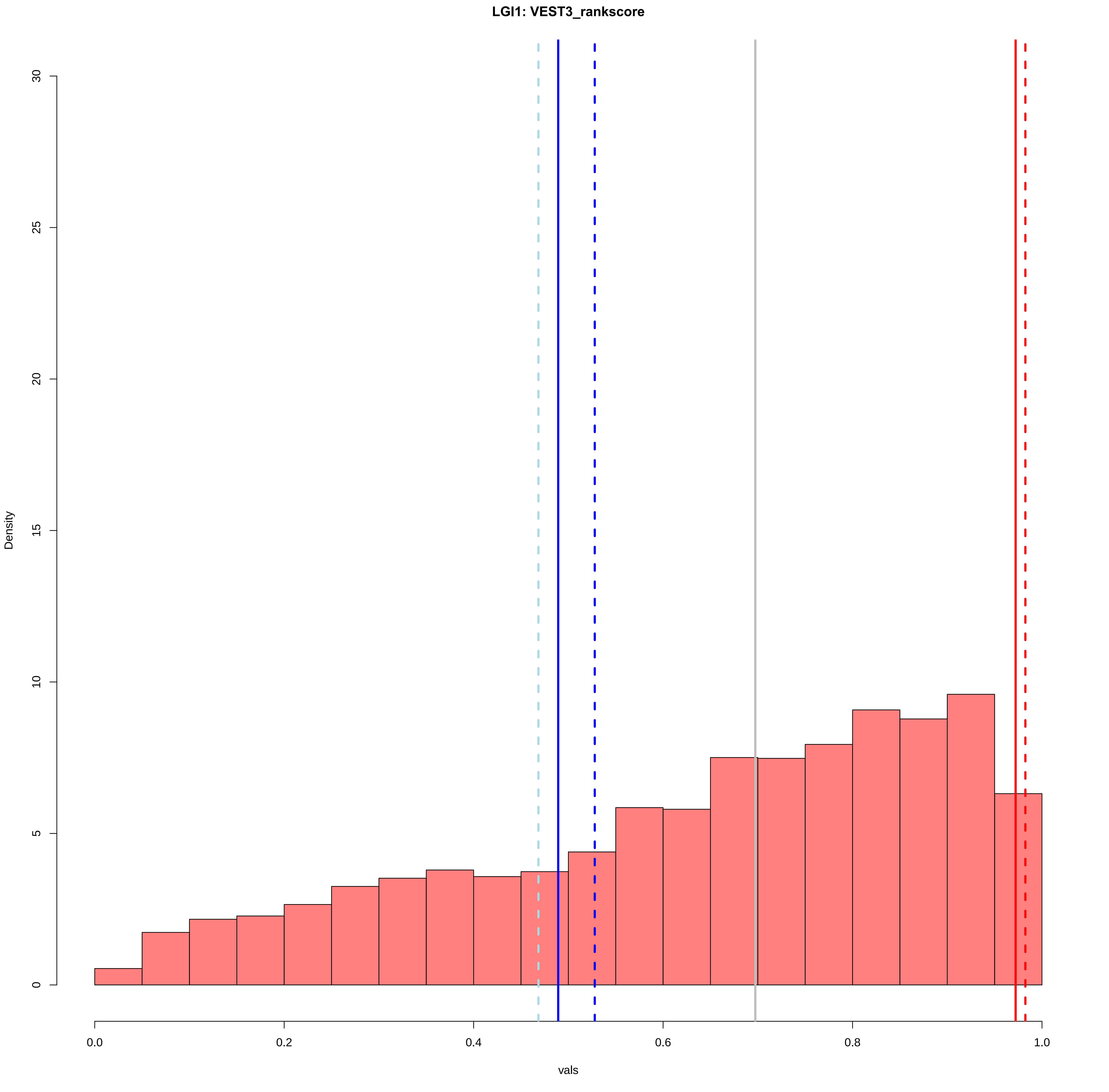
LGI1: VEST3_rankscore
Density
vals
0
5
10
15
20
25
30
0.0
0.2
0.4
0.6
0.8
1.0

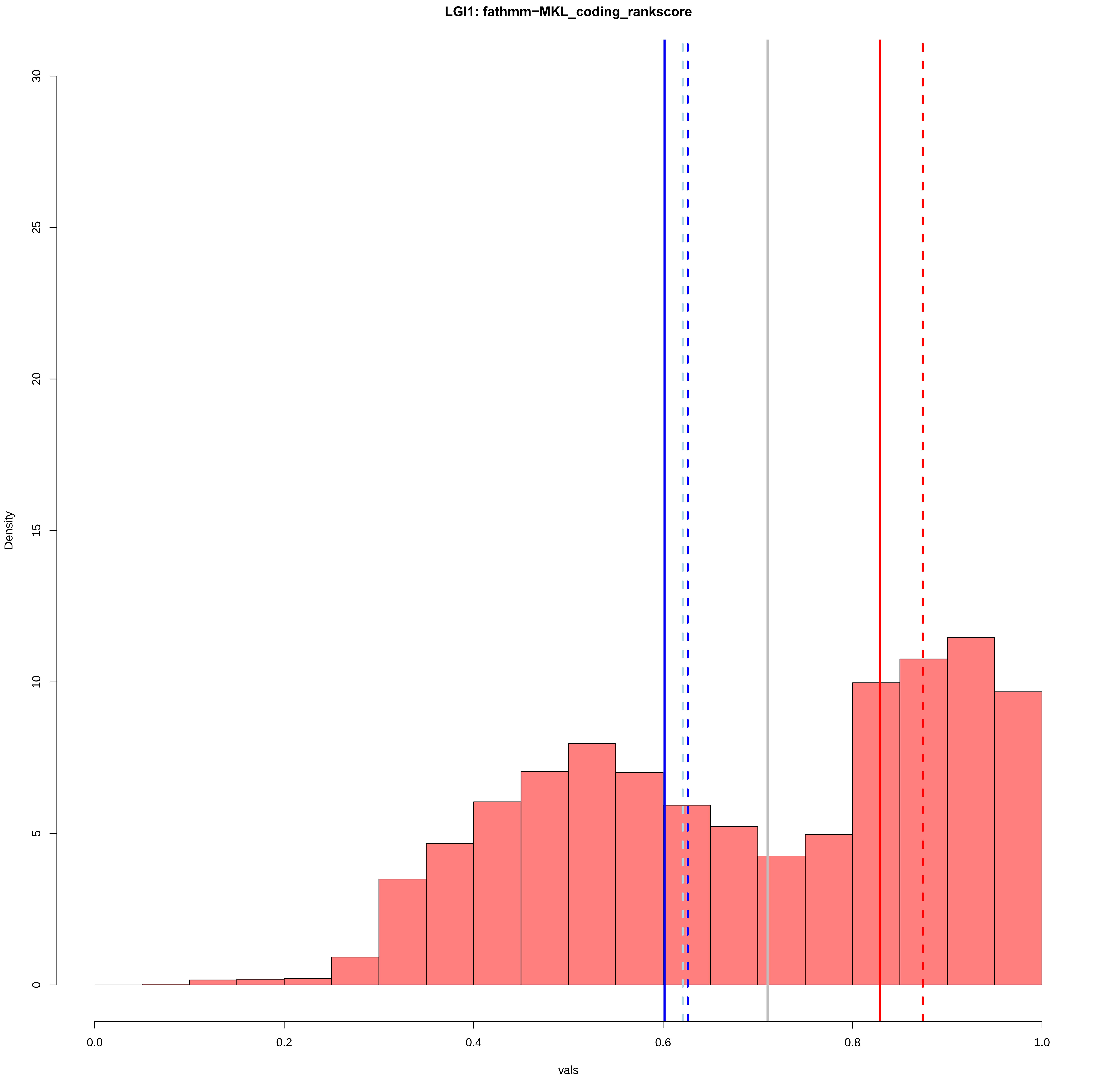

LGI1: fathmm−MKL_coding_rankscore
Density
vals
0
5
10
15
20
25
30
0.0
0.2
0.4
0.6
0.8
1.0

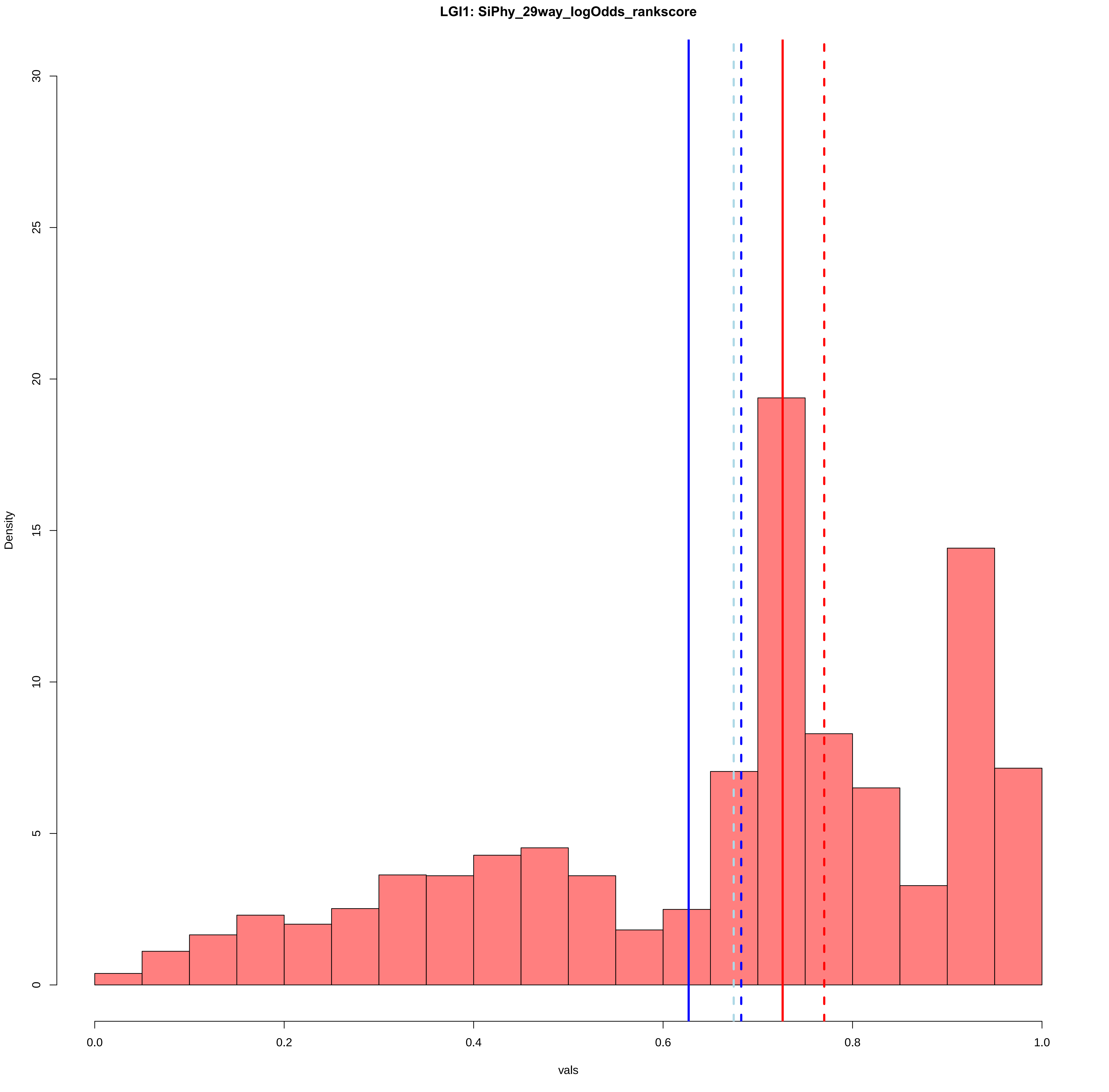

LGI1: SiPhy_29way_logOdds_rankscore
Density
vals
0
5
10
15
20
25
30
0.0
0.2
0.4
0.6
0.8
1.0

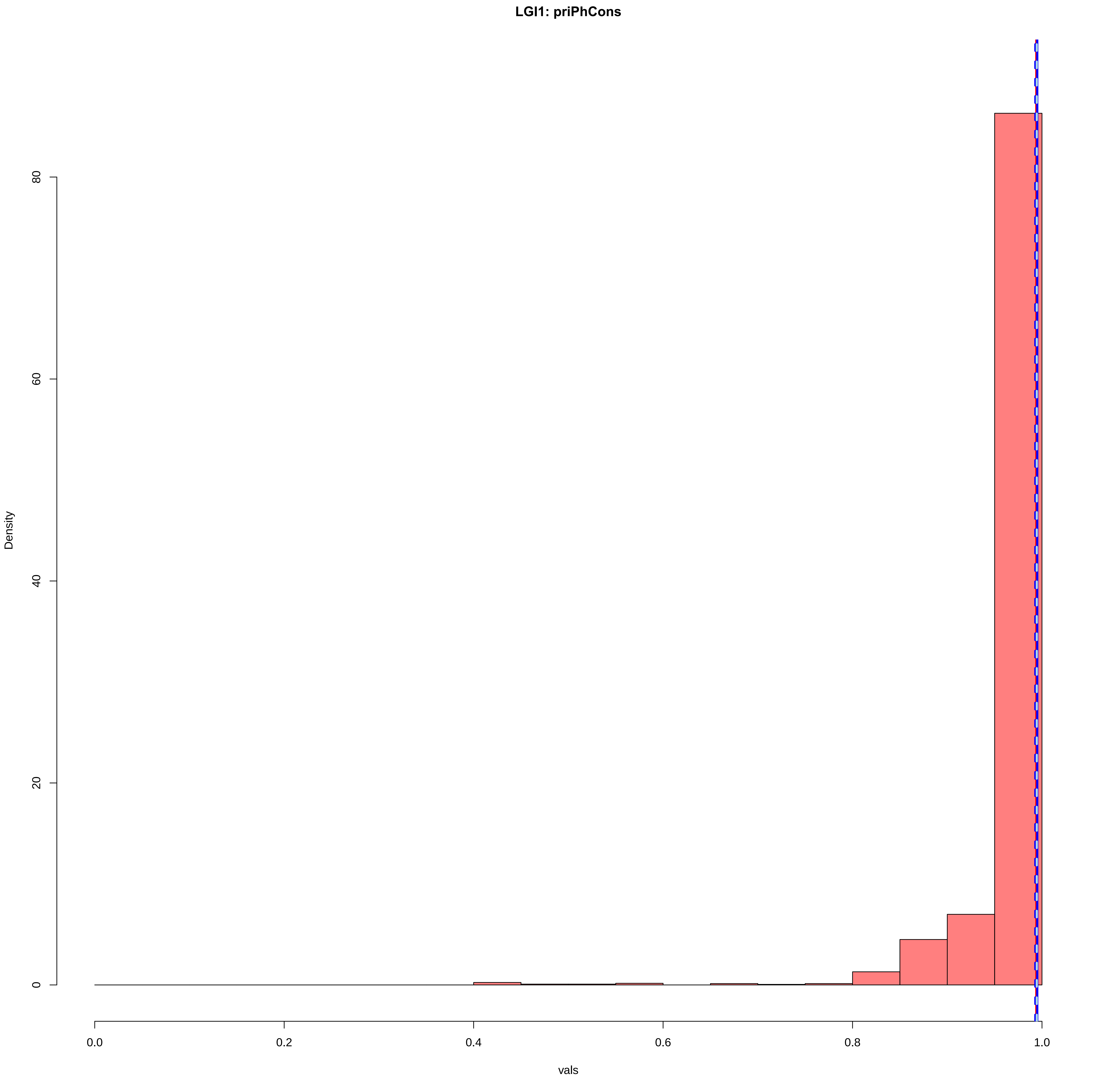
LGI1: priPhCons
Density
vals
0
20
40
60
80
0.0
0.2
0.4
0.6
0.8
1.0

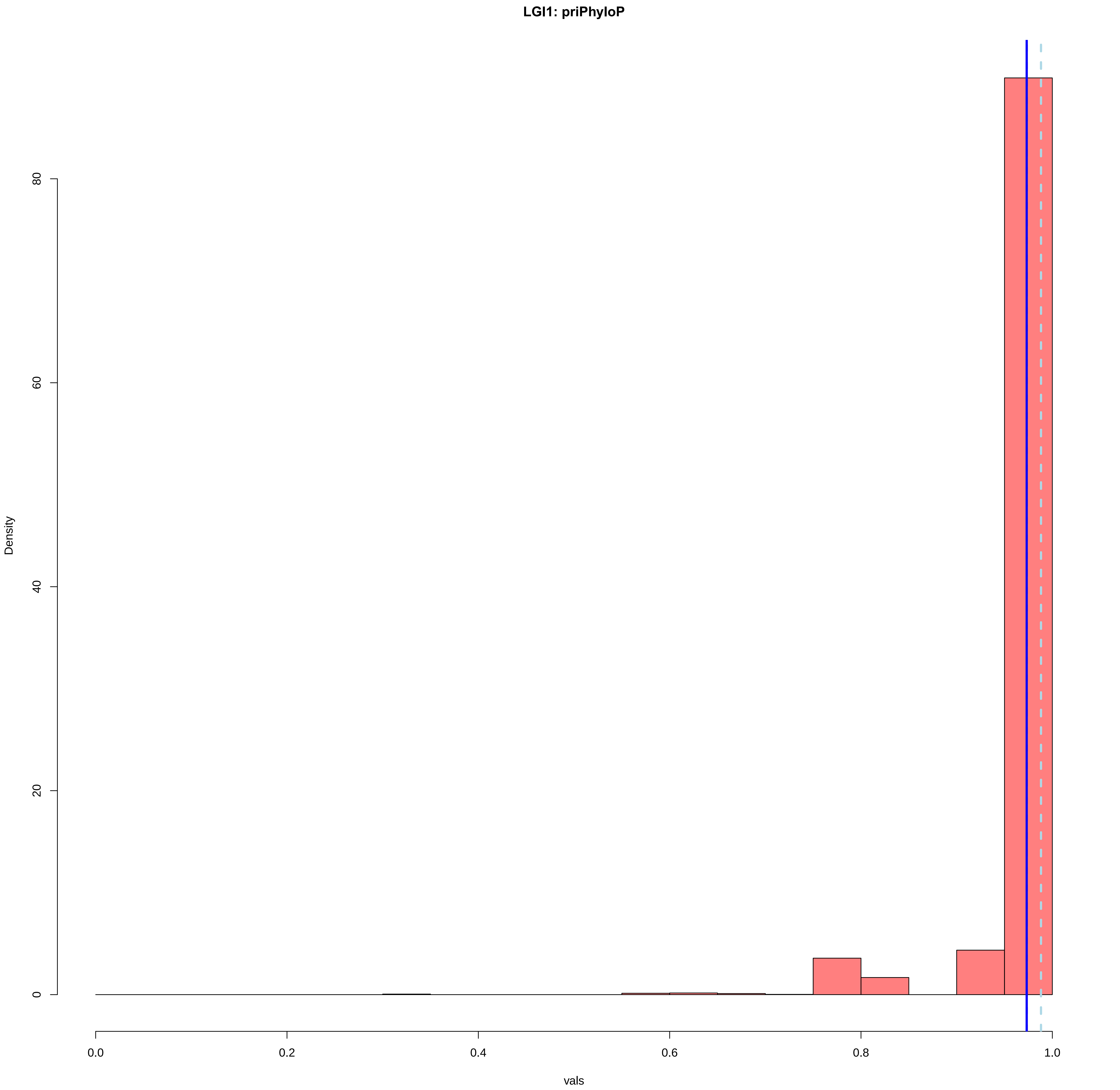
LGI1: priPhyloP
Density
0
20
40
60
80
0.0
0.2
0.4
0.6
0.8
1.0
vals

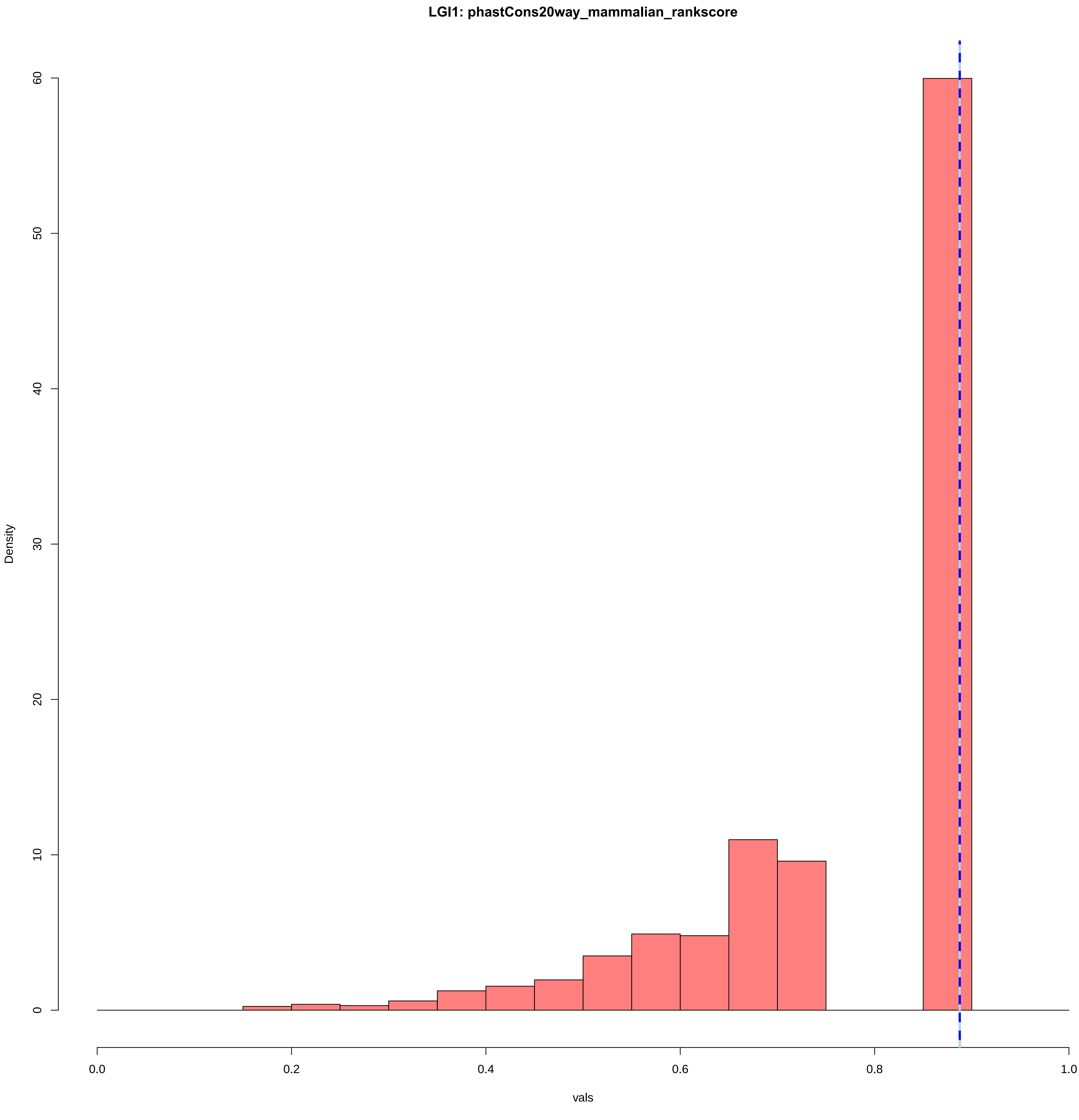
LGI1: phastCons20way_mammalian_rankscore
Density
vals
0
10
20
30
40
50
60
0.0
0.2
0.4
0.6
0.8
1.0

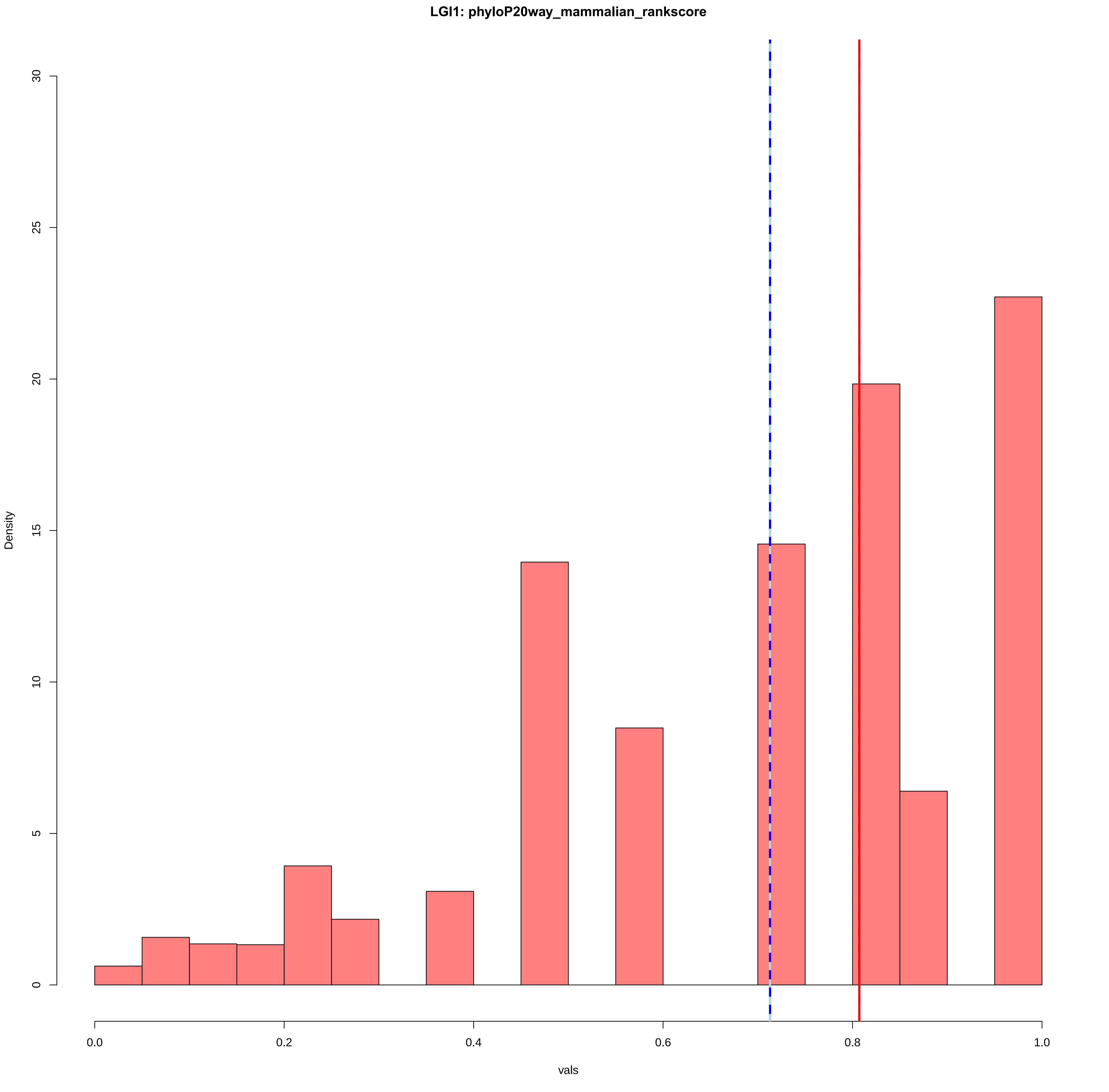
LGI1: phyloP20way_mammalian_rankscore
Density
vals
0
5
10
15
20
25
30
0.0
0.2
0.4
0.6
0.8
1.0

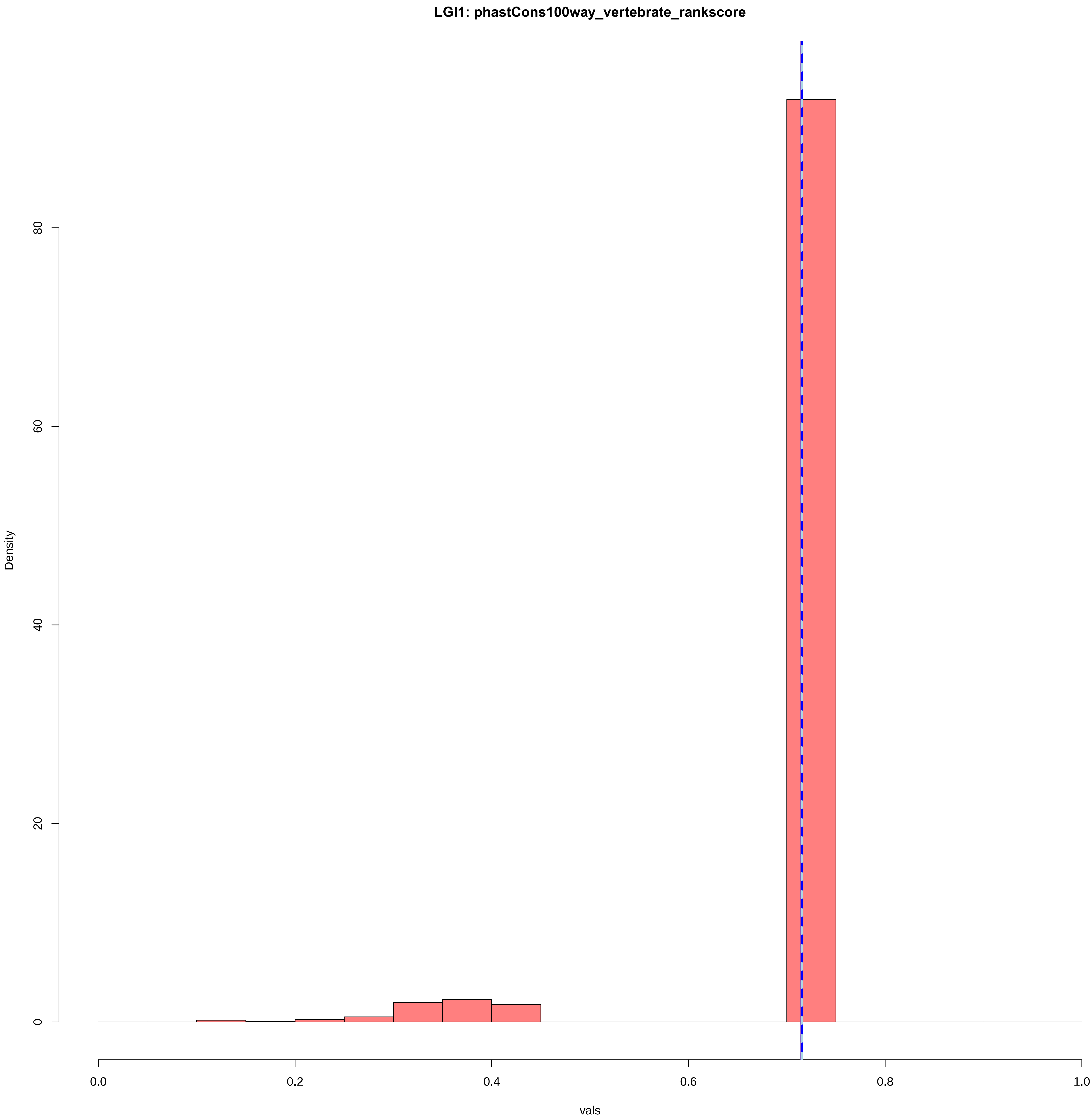

LGI1: phastCons100way_vertebrate_rankscore
Density
0
20
40
60
80
0.0
0.2
0.4
0.6
0.8
1.0
vals

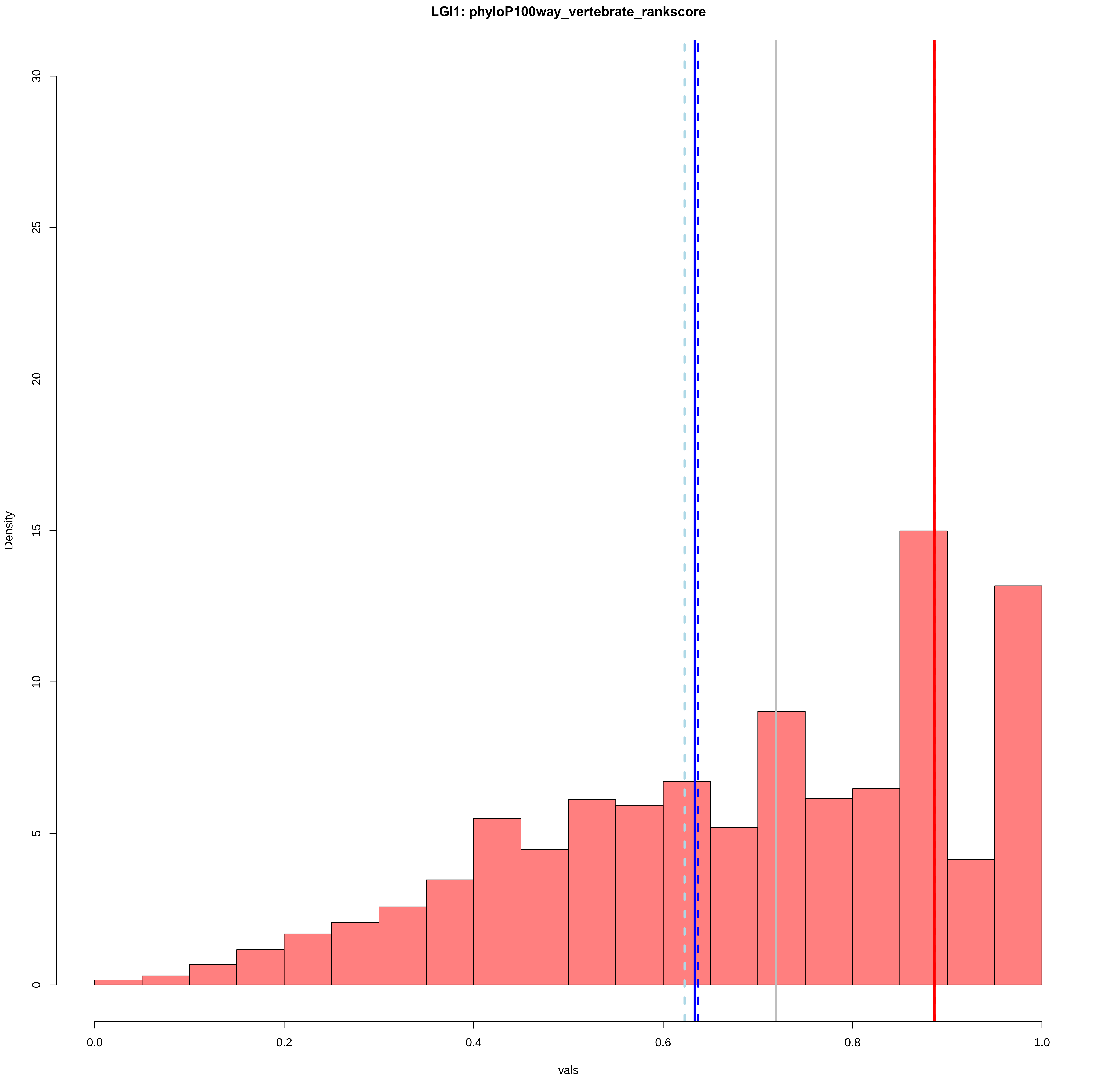

LGI1: phyloP100way_vertebrate_rankscore
Density
vals
0
5
10
15
20
25
30
0.0
0.2
0.4
0.6
0.8
1.0

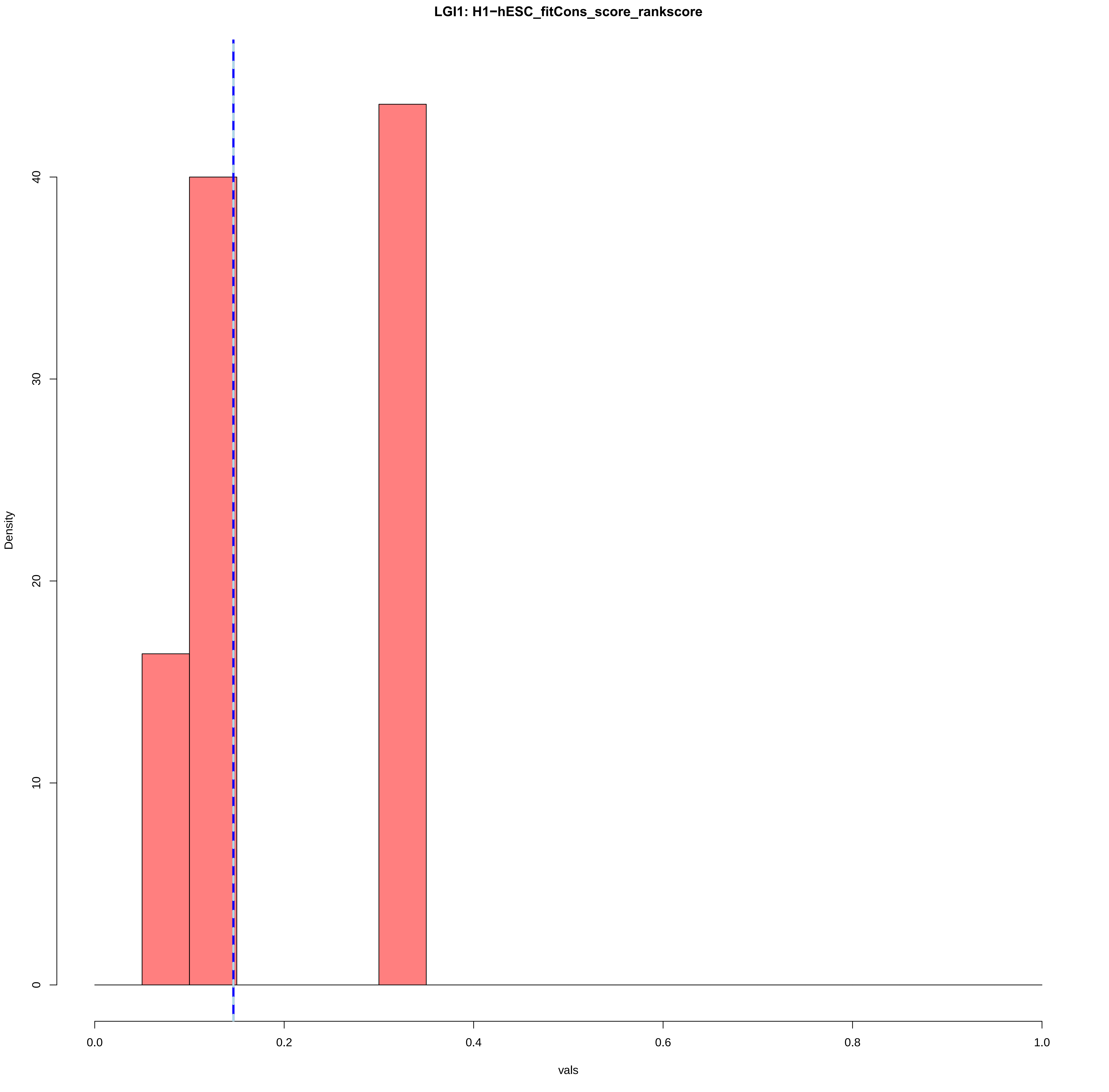
LGI1: H1−hESC_fitCons_score_rankscore
Density
vals
0
10
20
30
40
0.0
0.2
0.4
0.6
0.8
1.0

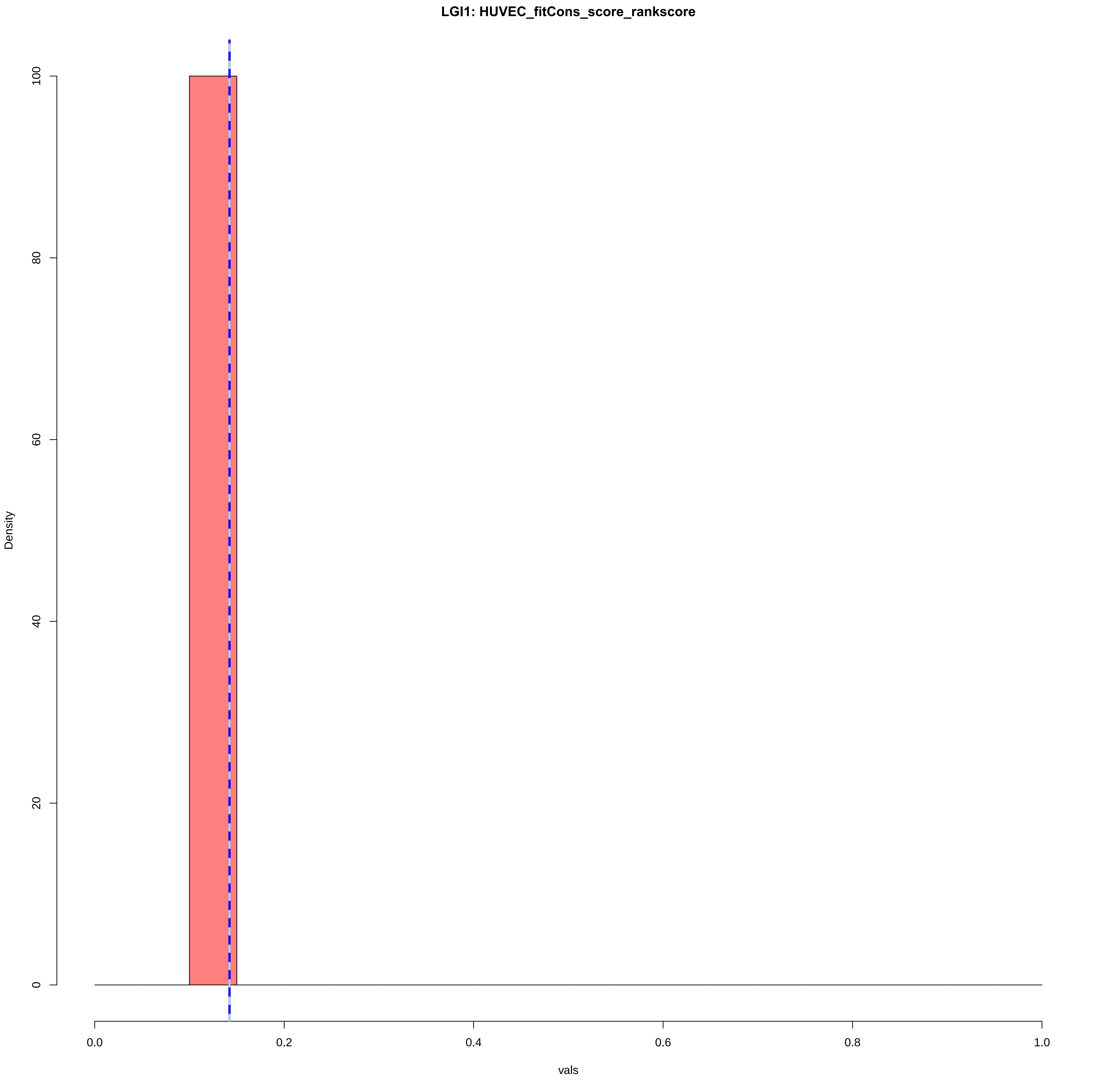
LGI1: HUVEC_fitCons_score_rankscore
Density
vals
0
20
40
60
80
100
0.0
0.2
0.4
0.6
0.8
1.0

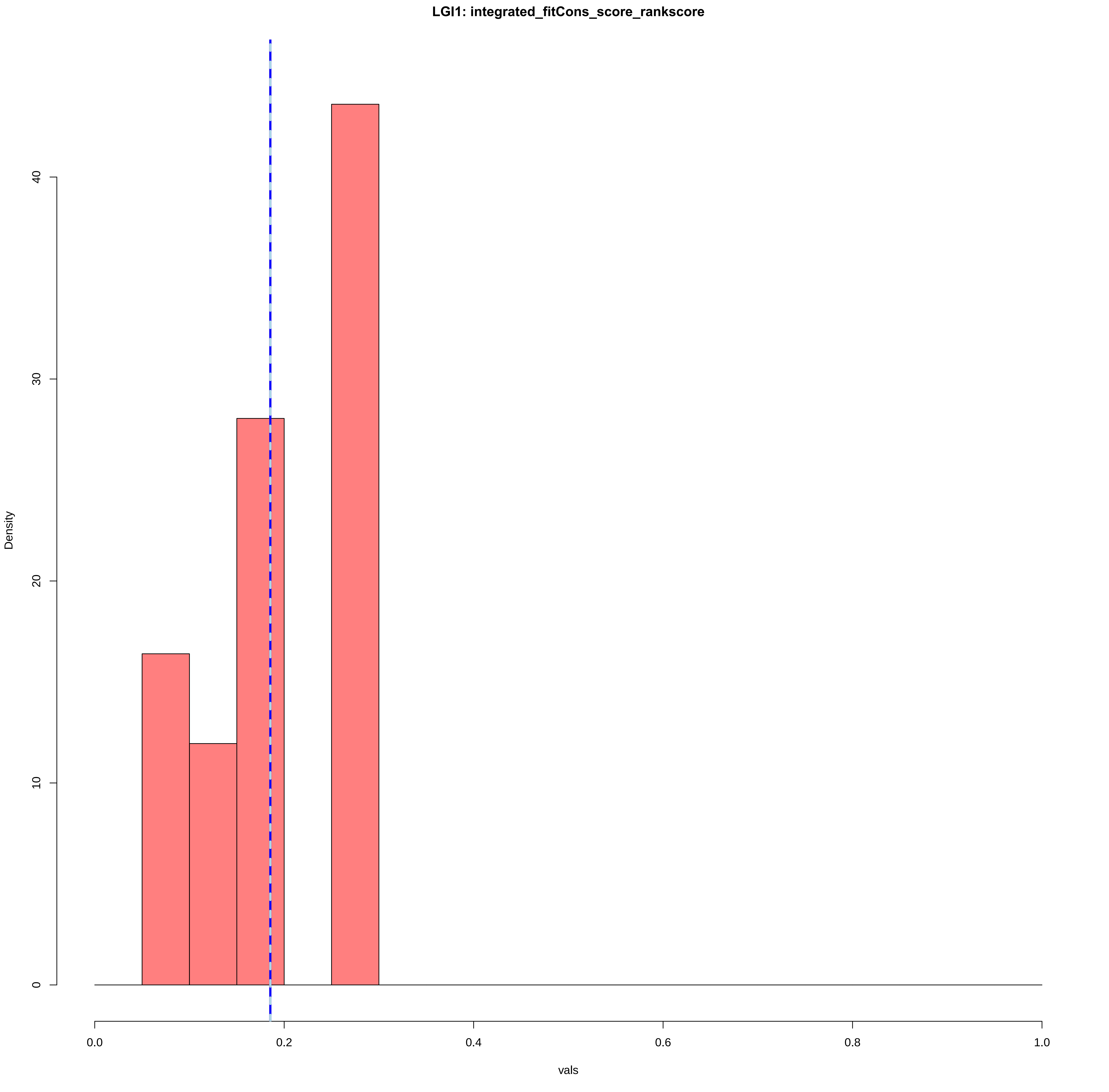
LGI1: integrated_fitCons_score_rankscore
Density
vals
0
10
20
30
40
0.0
0.2
0.4
0.6
0.8
1.0

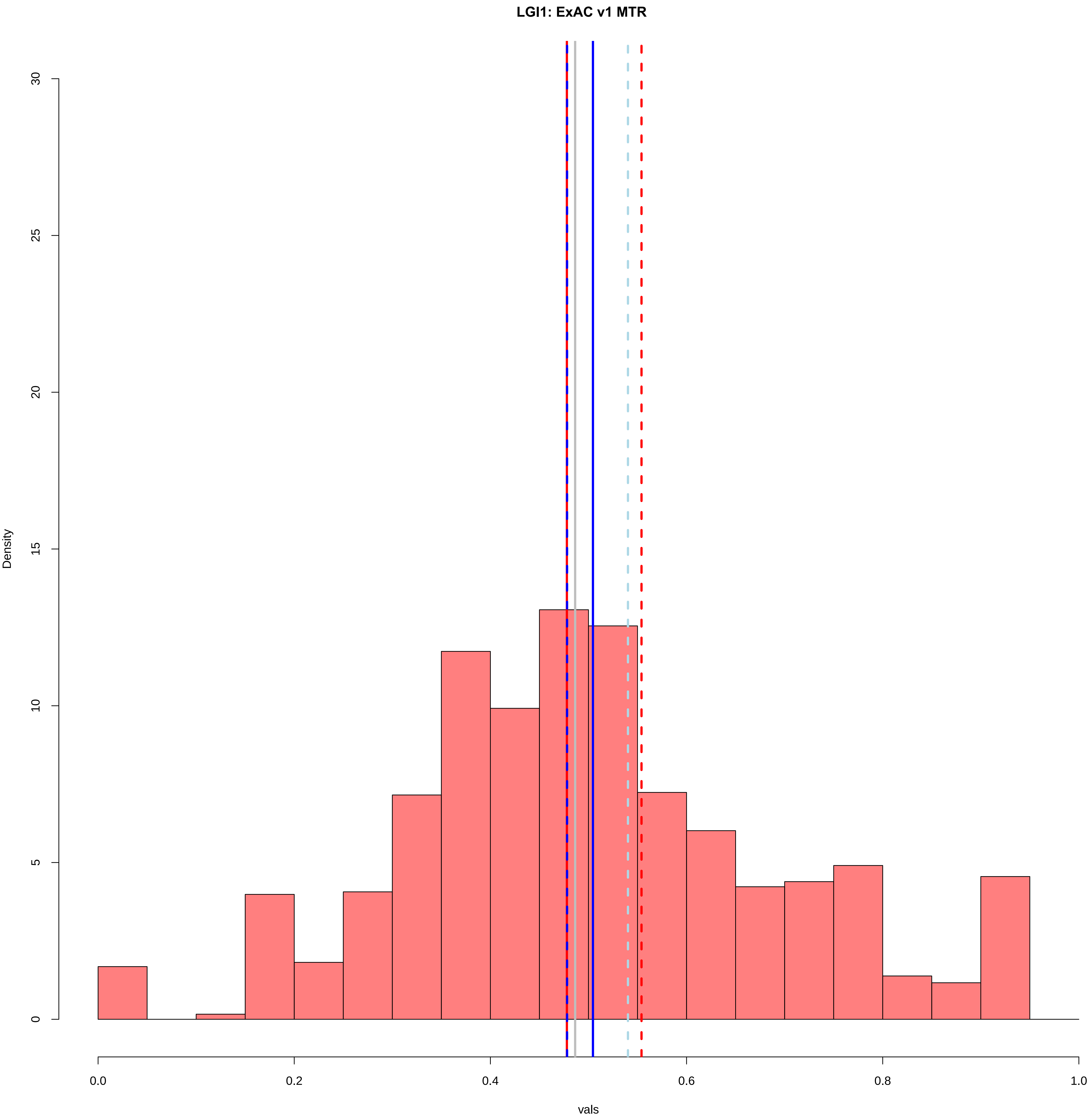

LGI1: ExAC v1 MTR
Density
vals
0
5
10
15
20
25
30
0.0
0.2
0.4
0.6
0.8
1.0

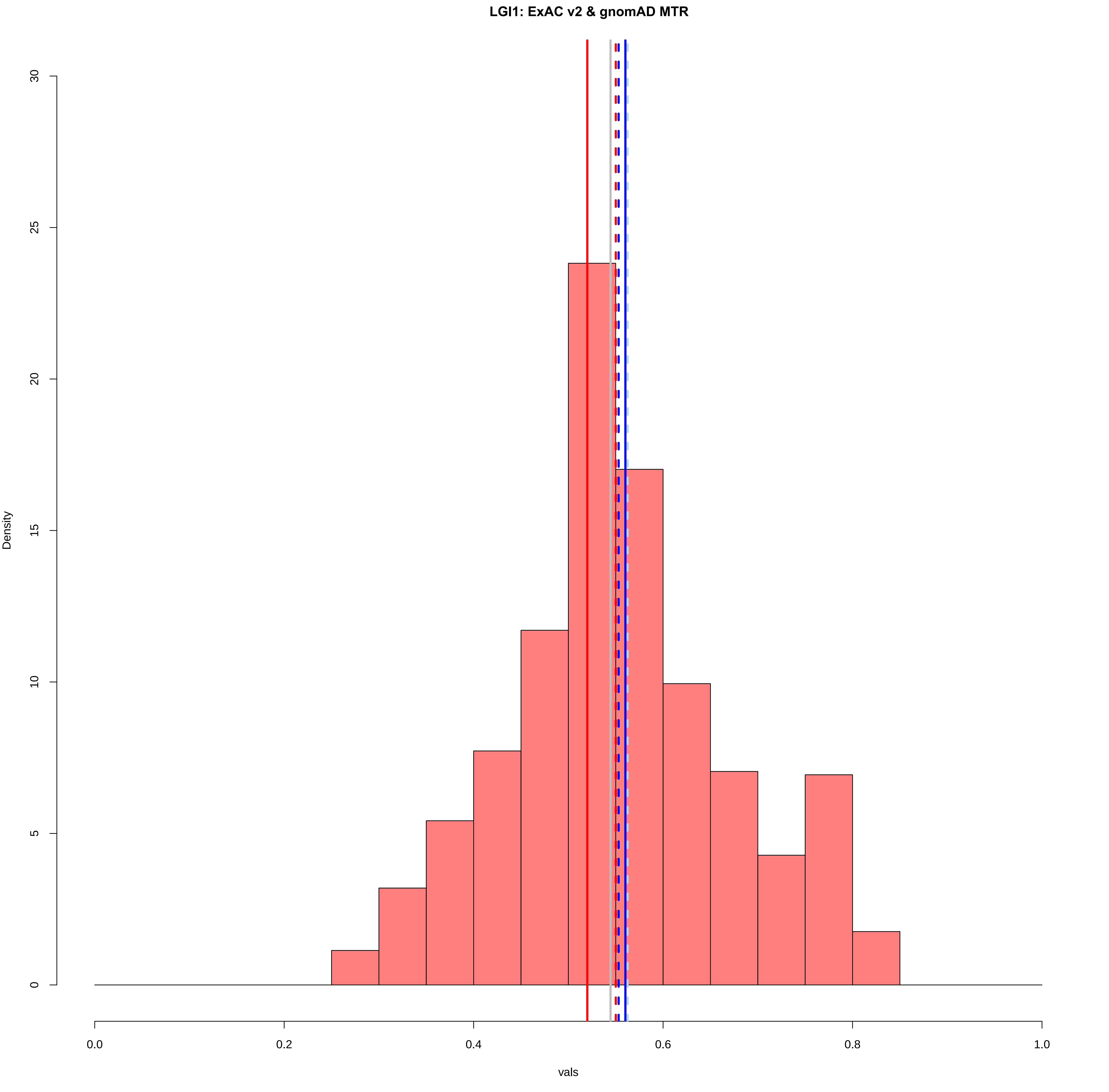

LGI1: ExAC v2 & gnomAD MTR
Density
vals
0
5
10
15
20
25
30
0.0
0.2
0.4
0.6
0.8
1.0

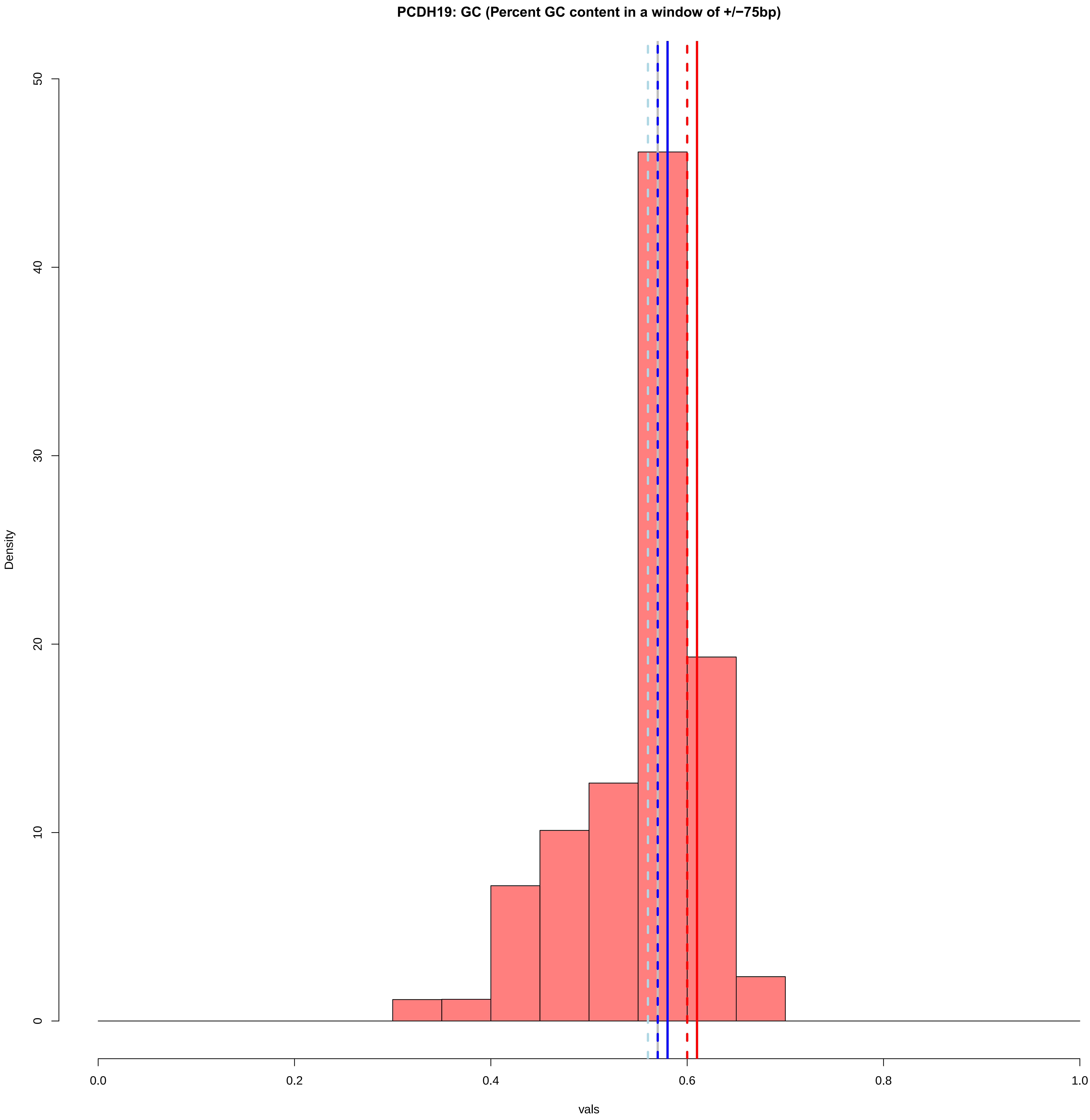
PCDH19: GC (Percent GC content in a window of +/−75bp)
Density
0
10
20
30
40
50
0.0
0.2
0.4
0.6
0.8
1.0
vals

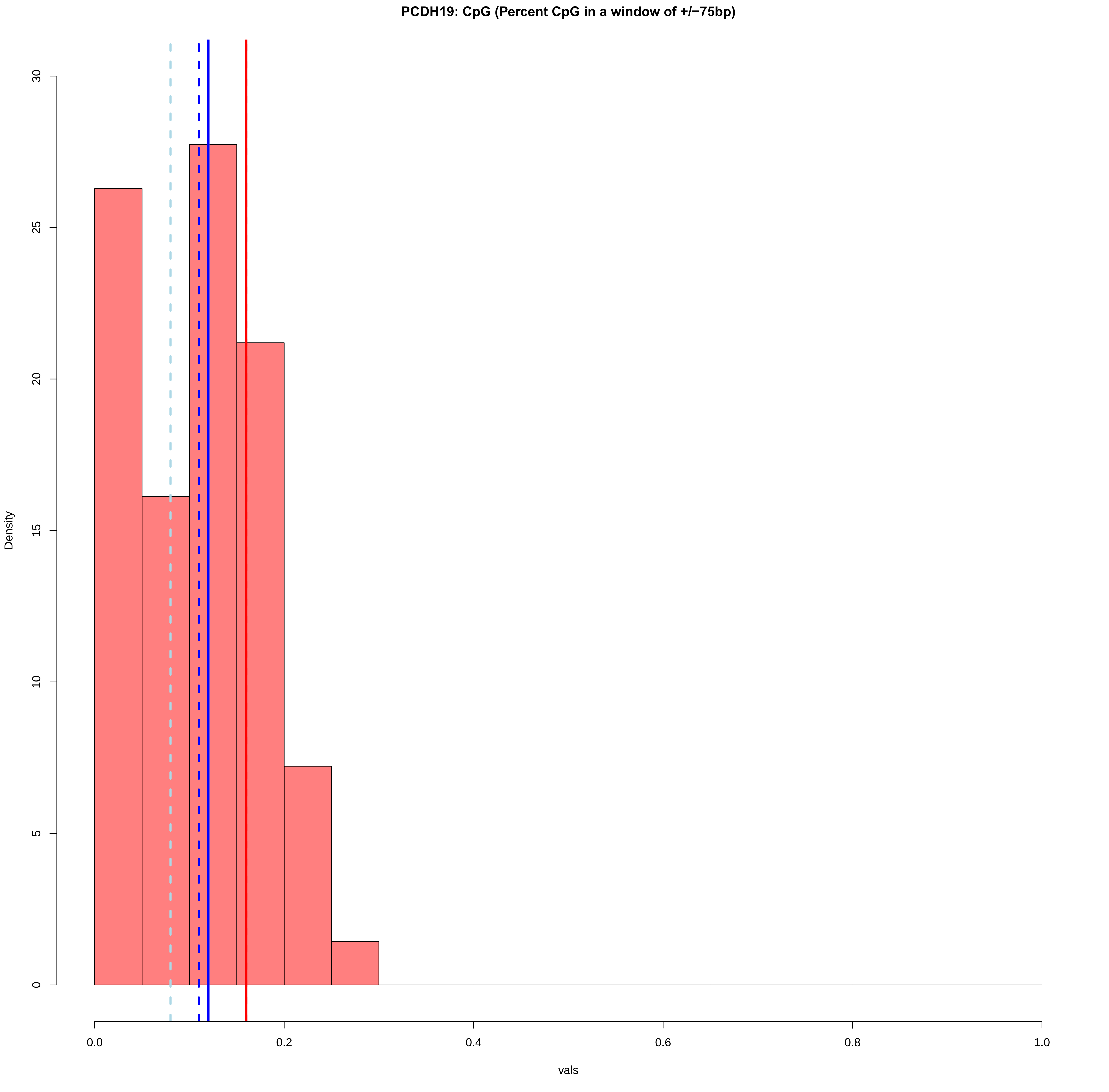
PCDH19: CpG (Percent CpG in a window of +/−75bp)
Density
vals
0
5
10
15
20
25
30
0.0
0.2
0.4
0.6
0.8
1.0

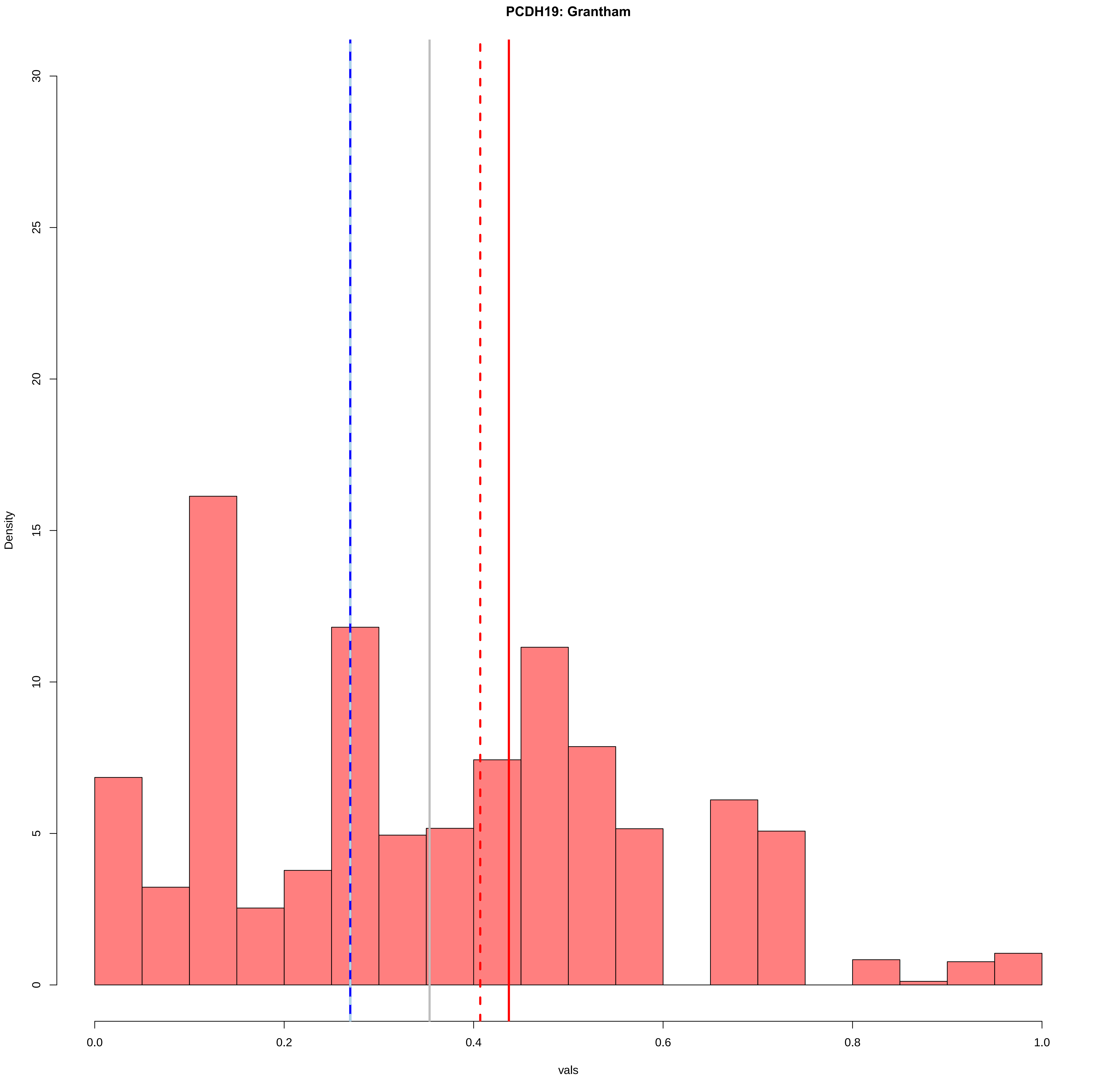
PCDH19: Grantham
Density
vals
0
5
10
15
20
25
30
0.0
0.2
0.4
0.6
0.8
1.0

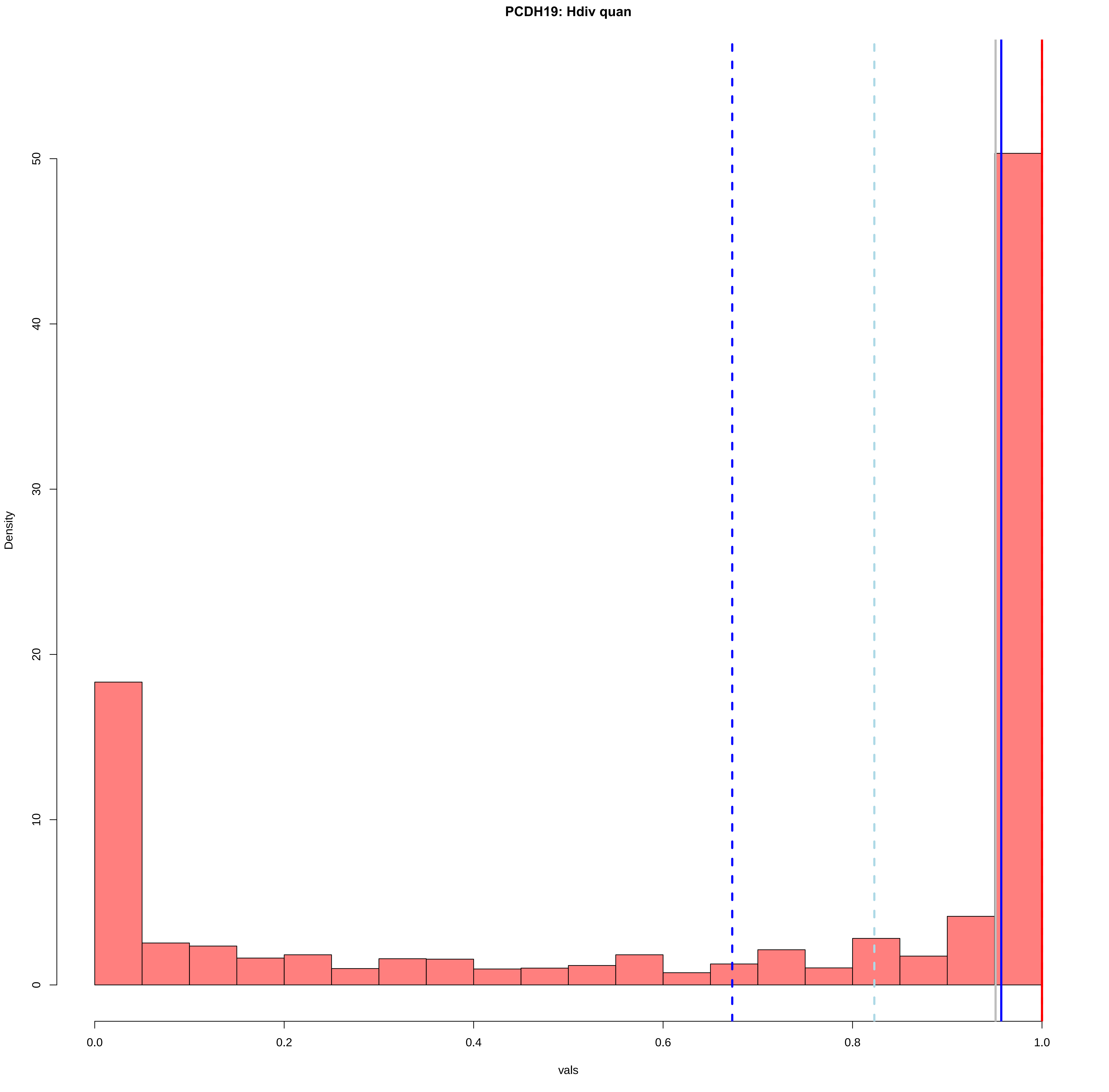

PCDH19: Hdiv quan
Density
0
10
20
30
40
50
0.0
0.2
0.4
0.6
0.8
1.0
vals

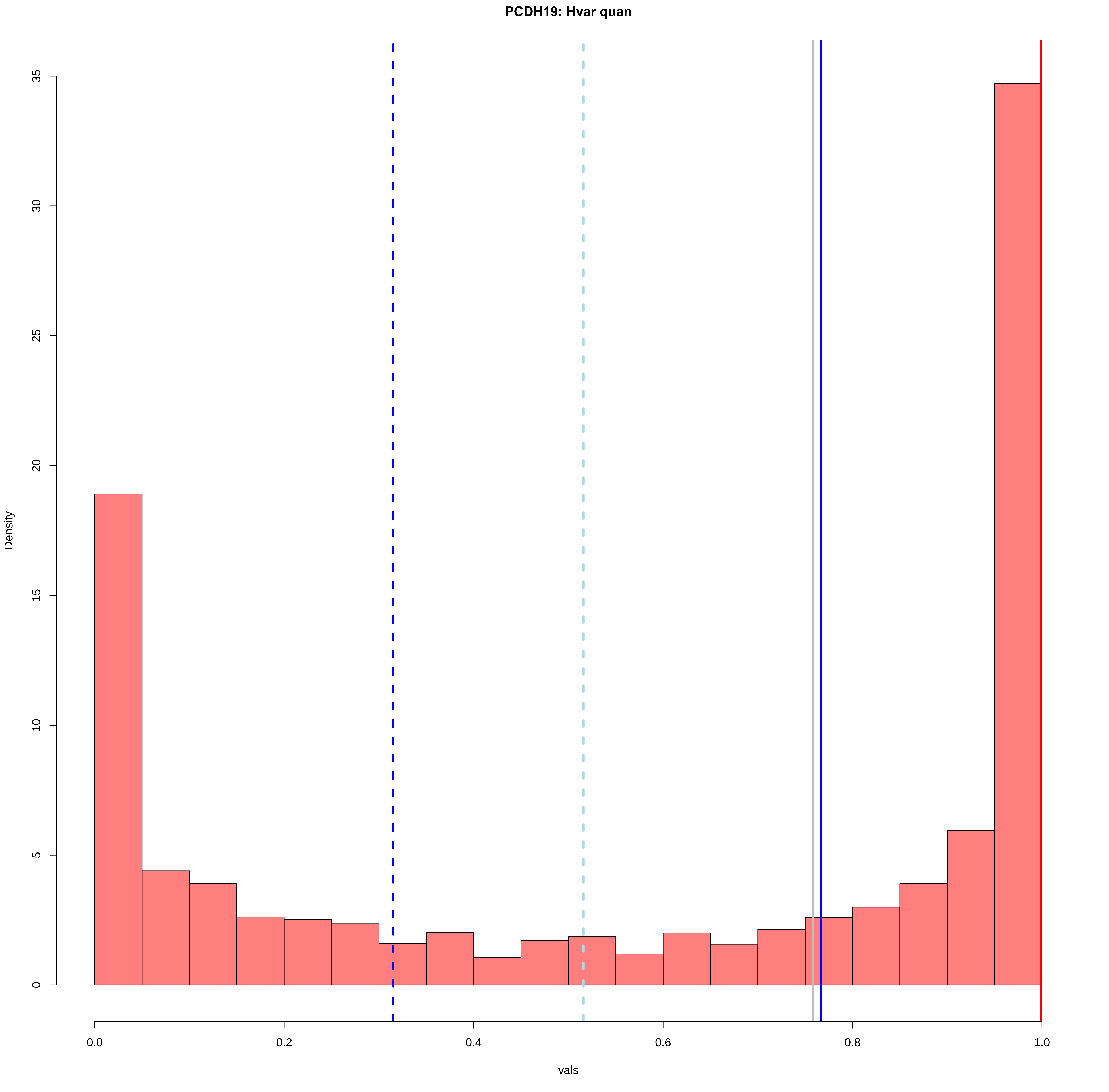
PCDH19: Hvar quan
Density
vals
0
5
10
15
20
25
30
35
0.0
0.2
0.4
0.6
0.8
1.0

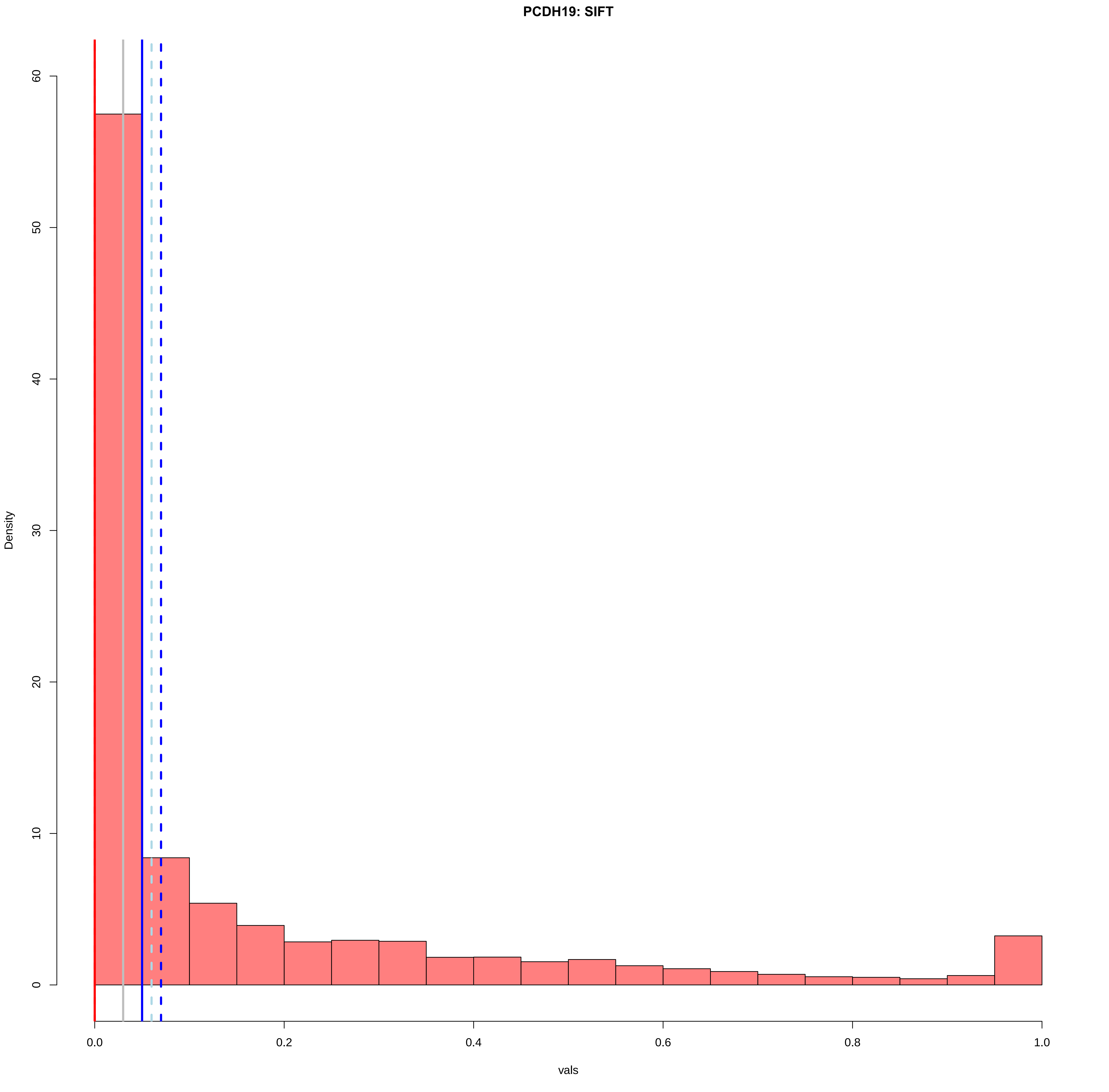

PCDH19: SIFT
Density
vals
0
10
20
30
40
50
60
0.0
0.2
0.4
0.6
0.8
1.0

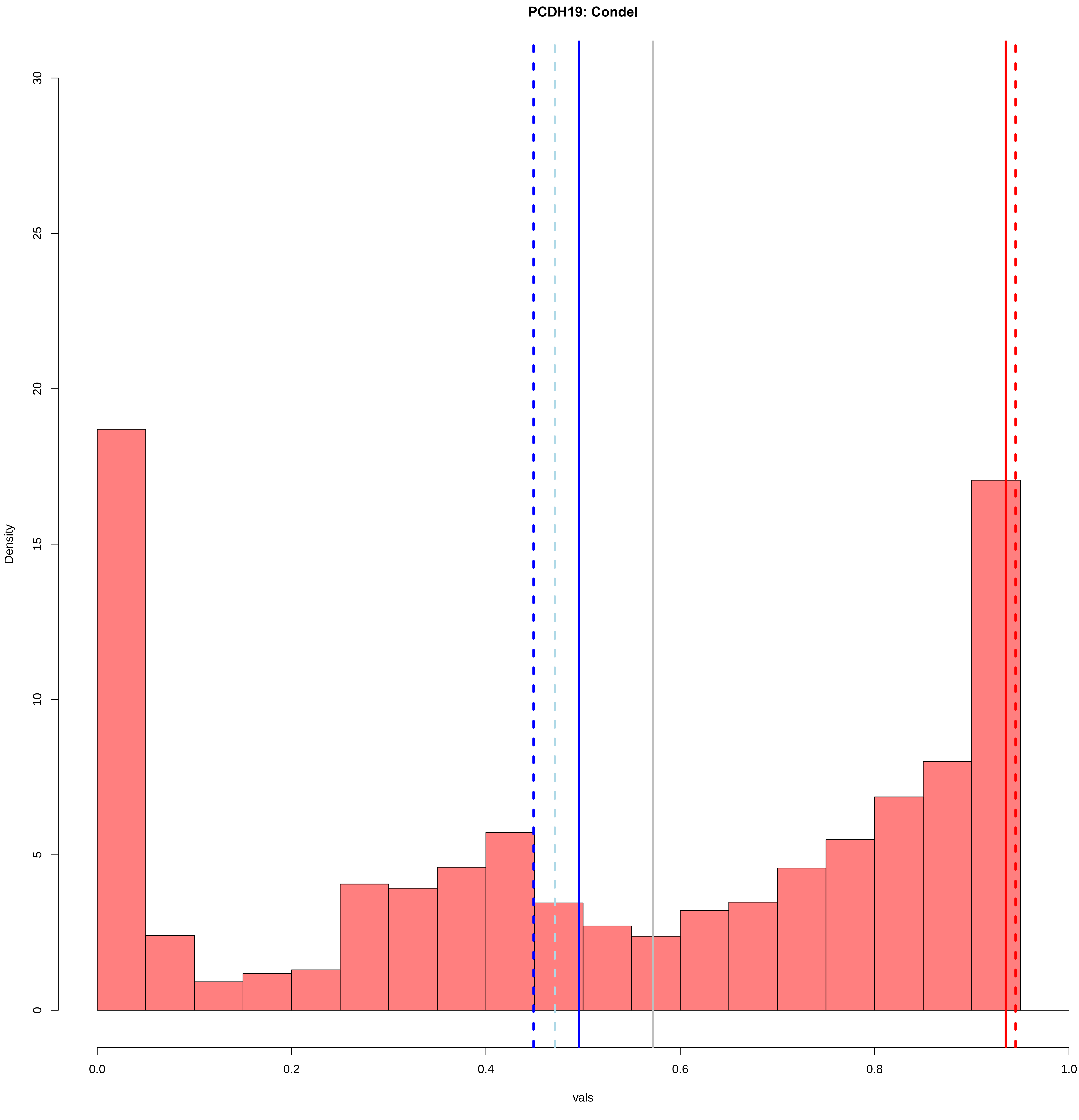

PCDH19: Condel
Density
vals
0
5
10
15
20
25
30
0.0
0.2
0.4
0.6
0.8
1.0

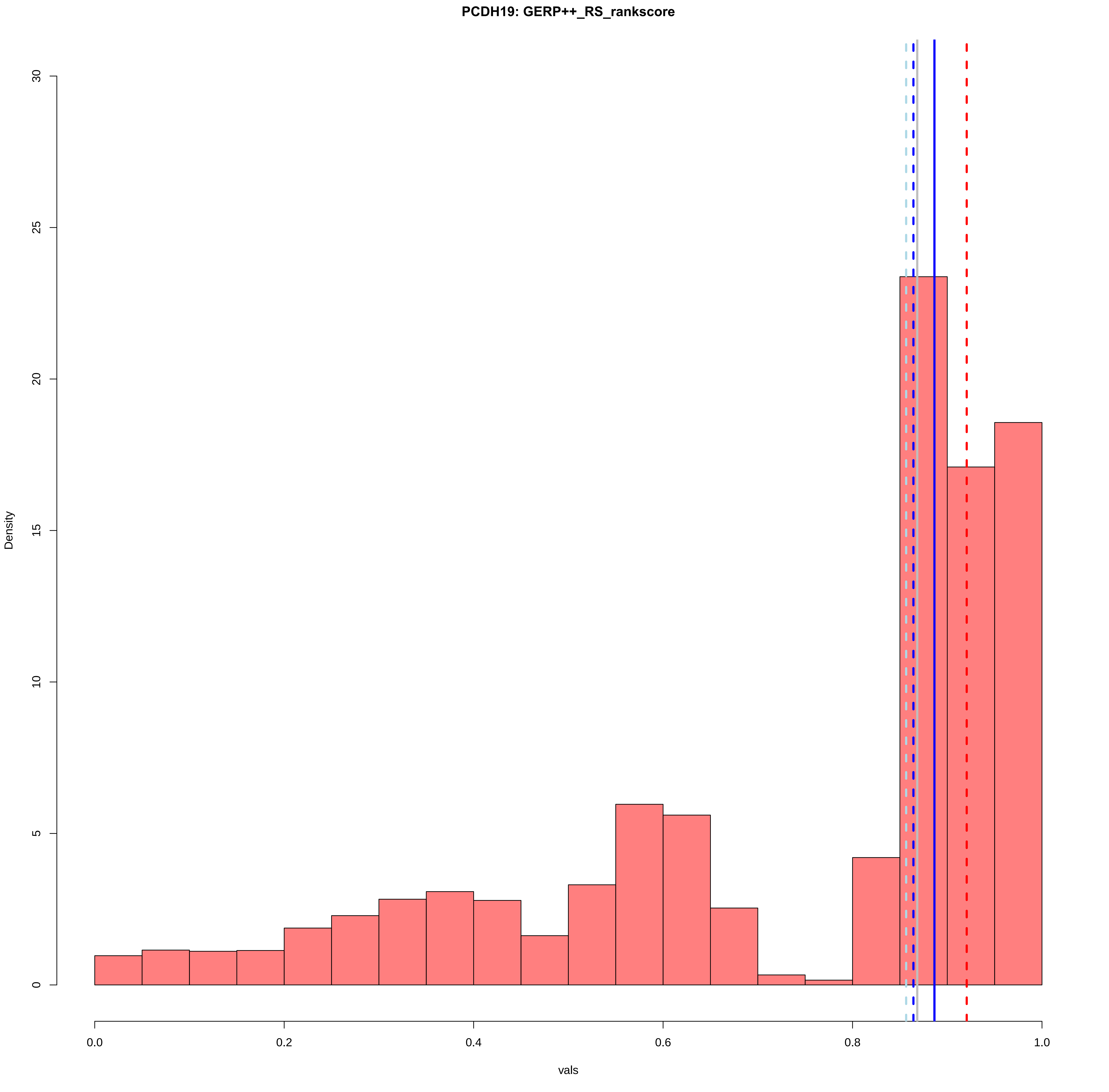

PCDH19: GERP++_RS_rankscore
Density
vals
0
5
10
15
20
25
30
0.0
0.2
0.4
0.6
0.8
1.0

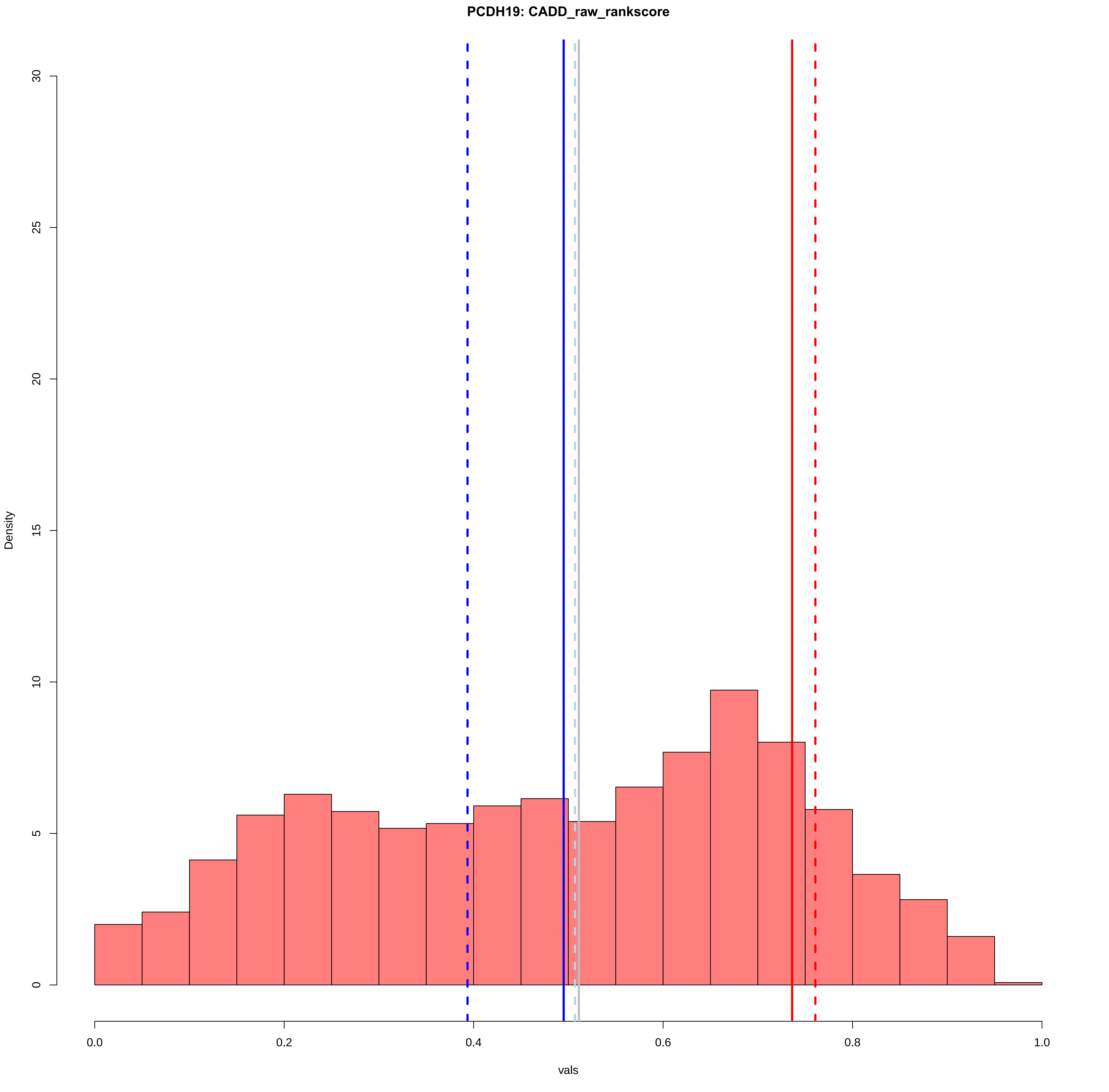
PCDH19: CADD_raw_rankscore
Density
vals
0
5
10
15
20
25
30
0.0
0.2
0.4
0.6
0.8
1.0

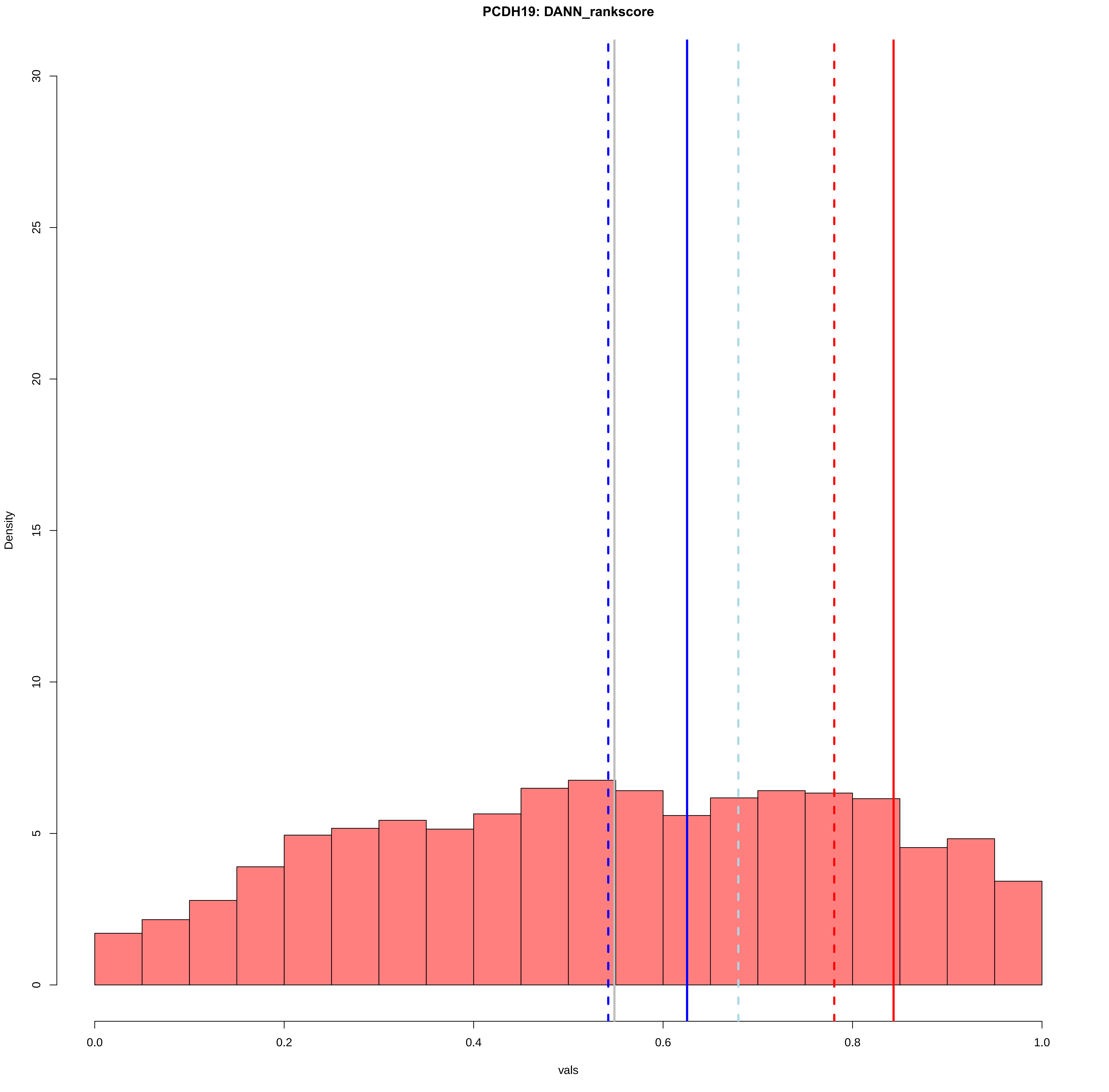

PCDH19: DANN_rankscore
Density
vals
0
5
10
15
20
25
30
0.0
0.2
0.4
0.6
0.8
1.0

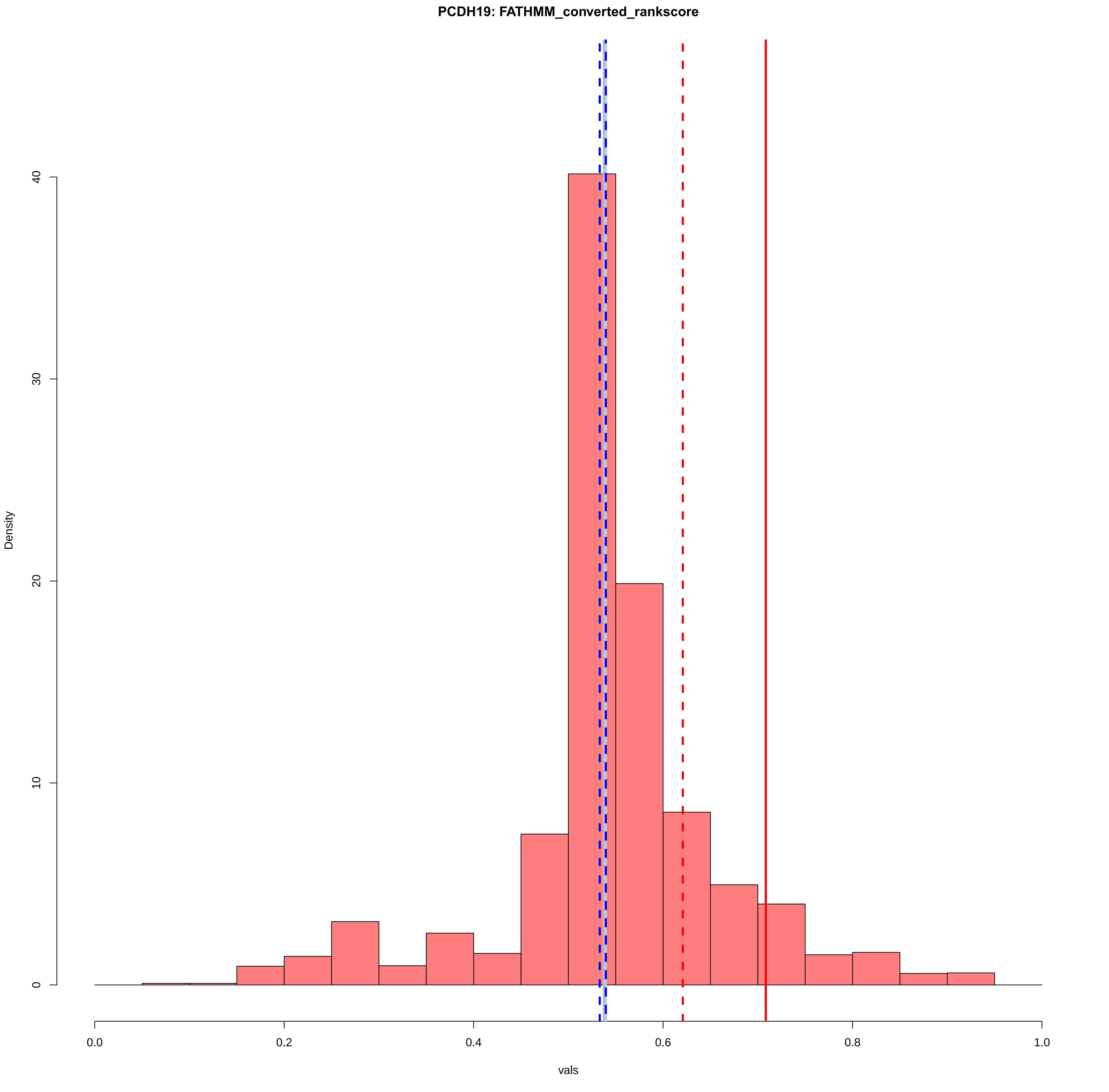
PCDH19: FATHMM_converted_rankscore
Density
vals
0
10
20
30
40
0.0
0.2
0.4
0.6
0.8
1.0

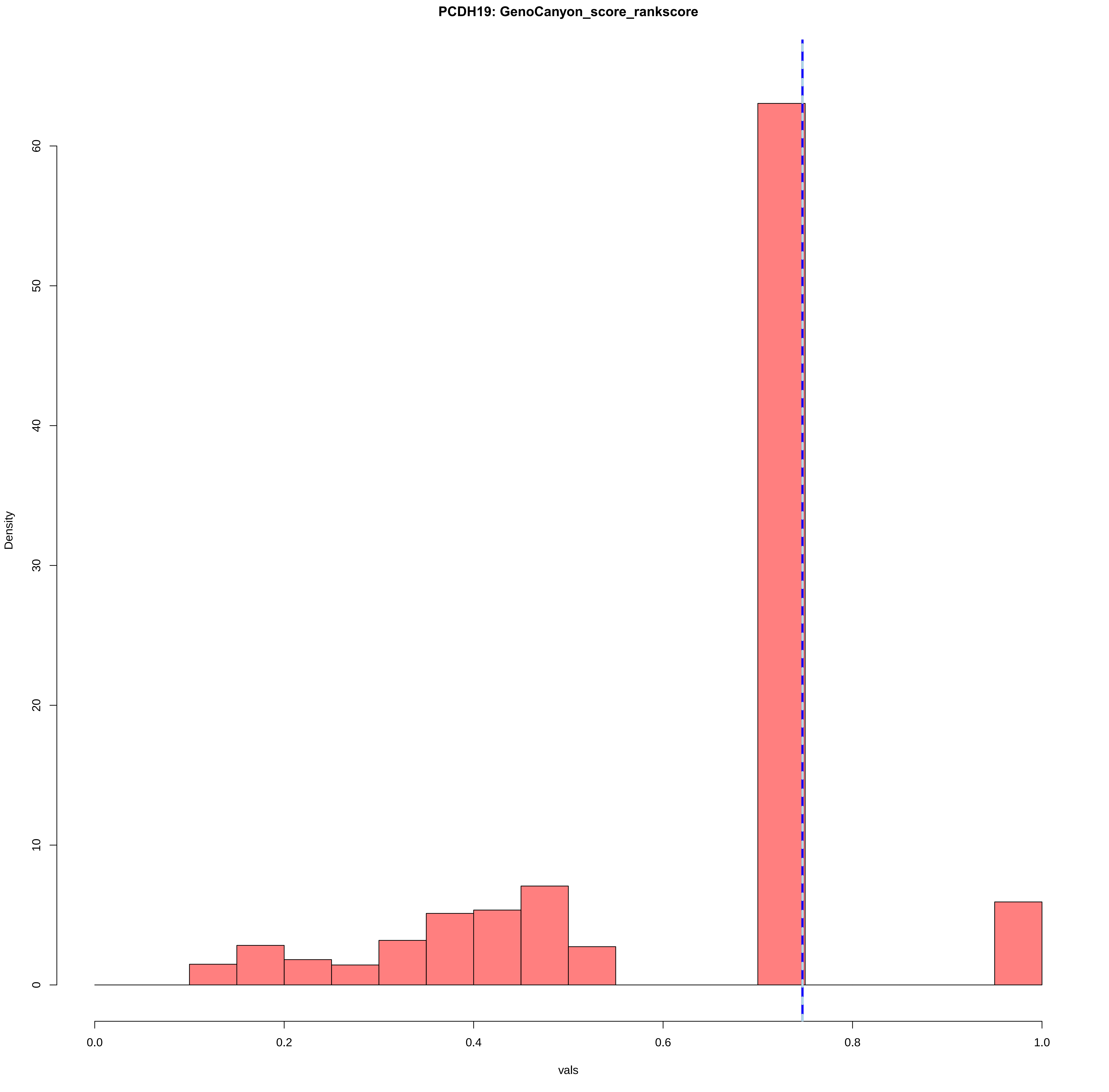
PCDH19: GenoCanyon_score_rankscore
Density
vals
0
10
20
30
40
50
60
0.0
0.2
0.4
0.6
0.8
1.0

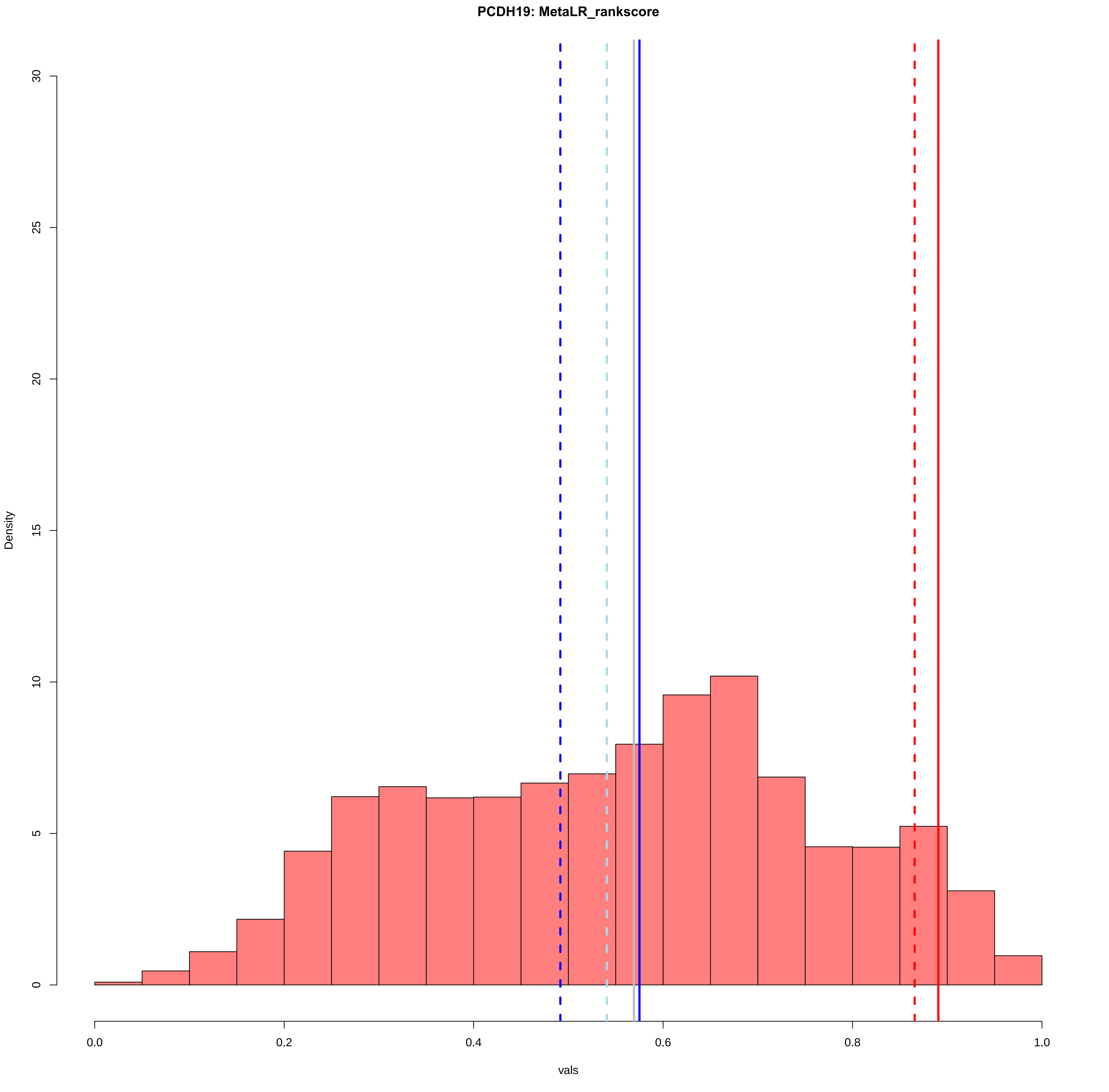

PCDH19: MetaLR_rankscore
Density
vals
0
5
10
15
20
25
30
0.0
0.2
0.4
0.6
0.8
1.0

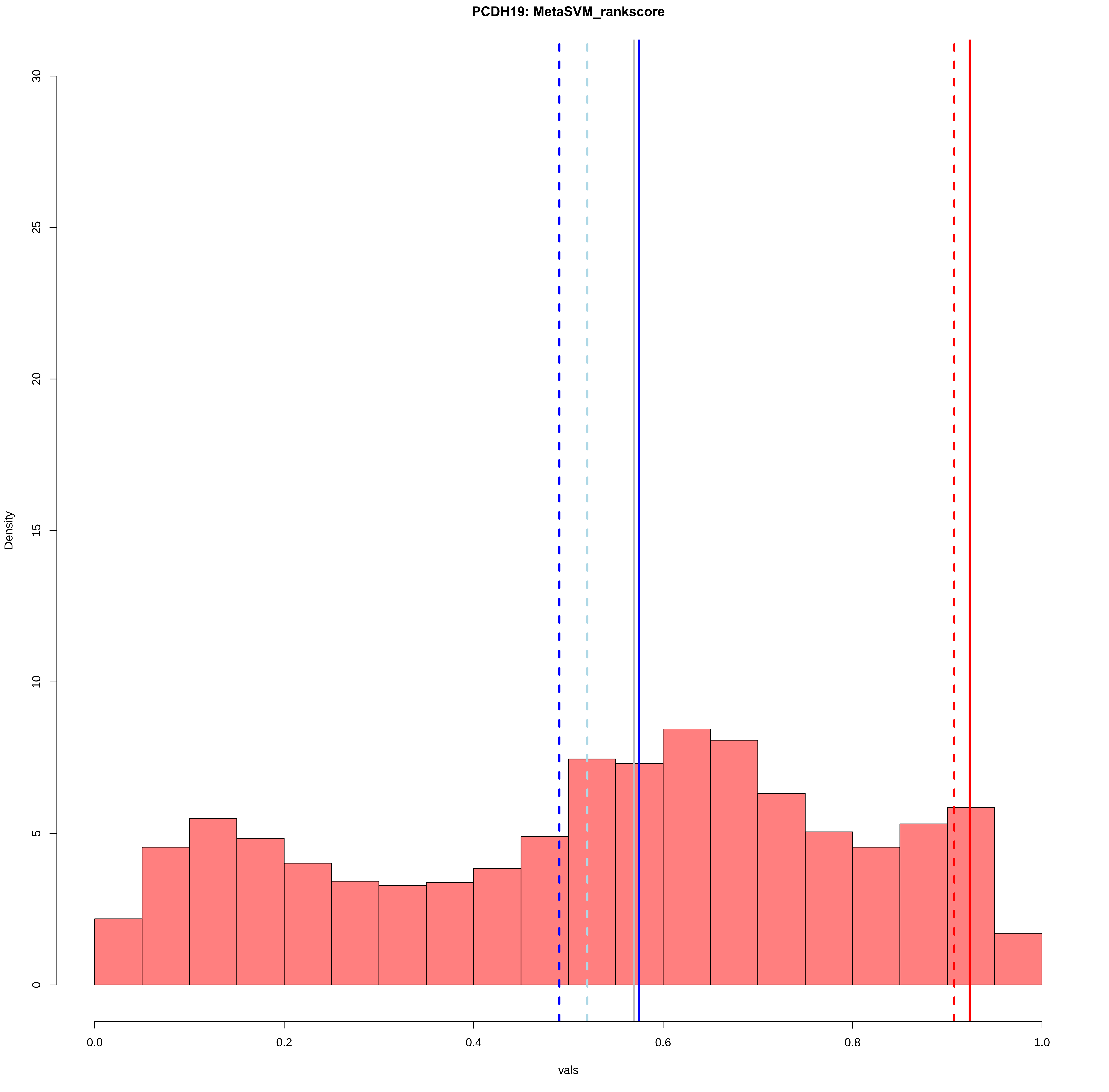

PCDH19: MetaSVM_rankscore
Density
0
5
10
15
20
25
30
0.0
0.2
0.4
0.6
0.8
1.0
vals

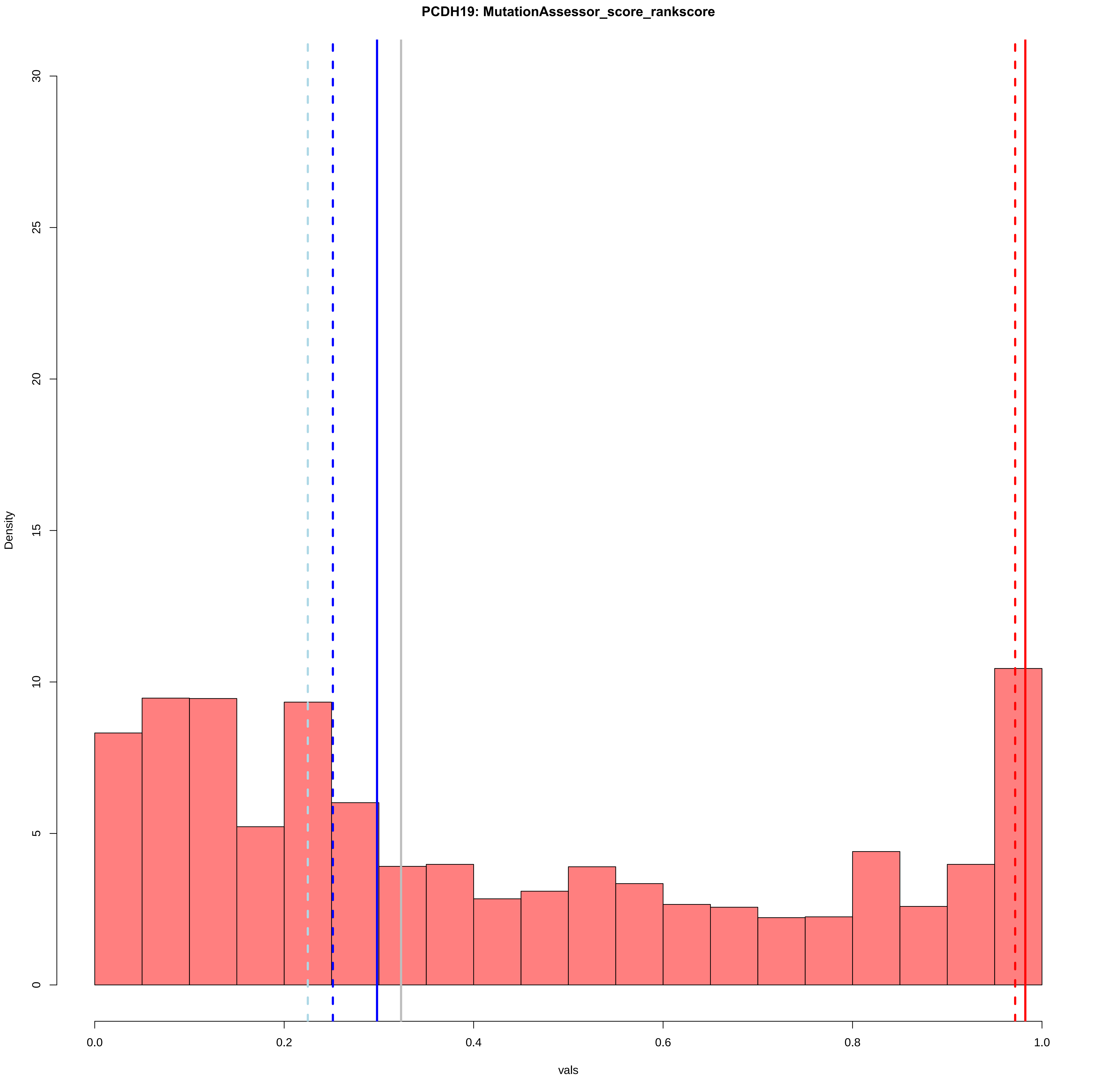
PCDH19: MutationAssessor_score_rankscore
Density
vals
0
5
10
15
20
25
30
0.0
0.2
0.4
0.6
0.8
1.0

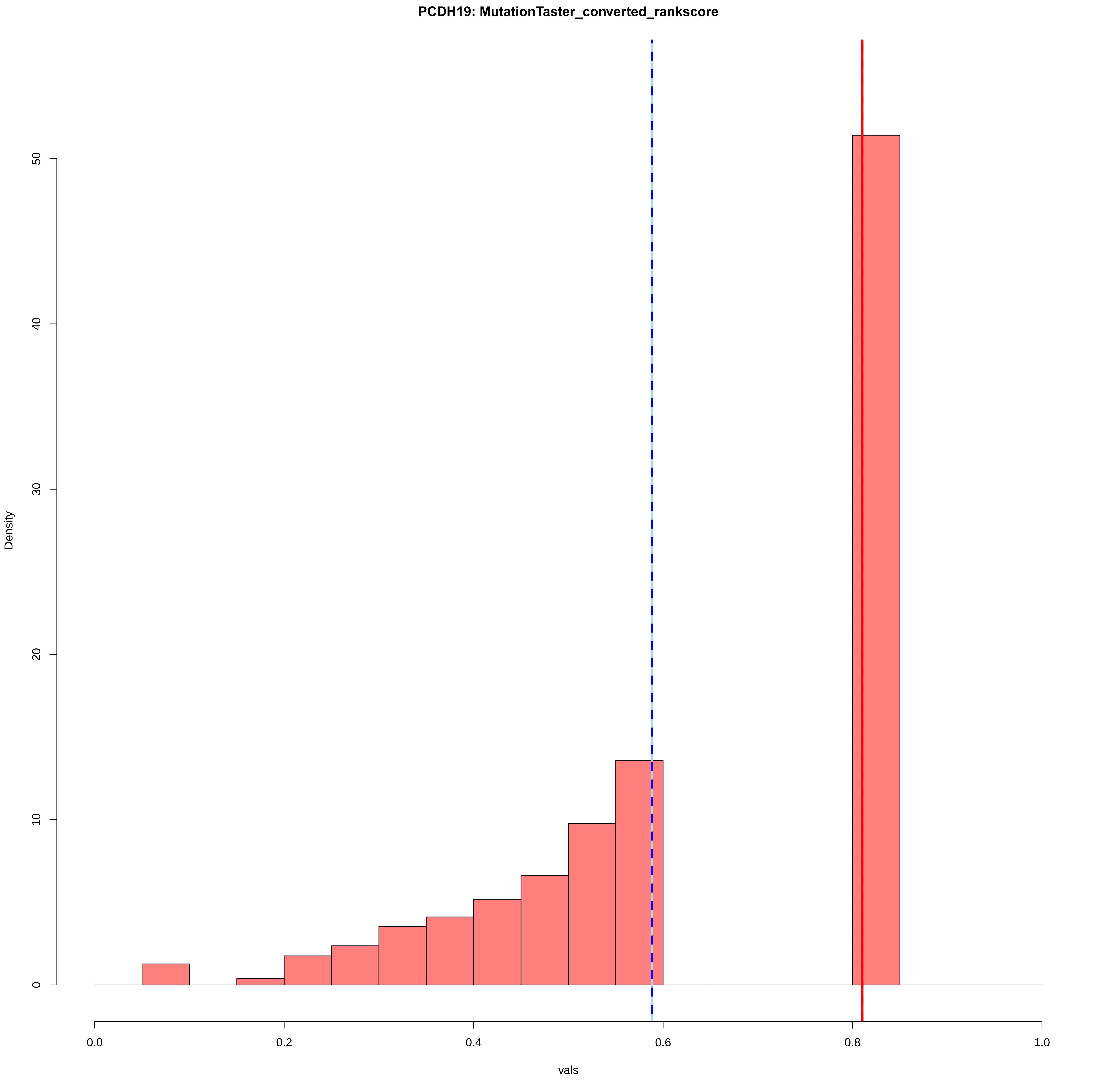

PCDH19: MutationTaster_converted_rankscore
Density
0
10
20
30
40
50
0.0
0.2
0.4
0.6
0.8
1.0
vals

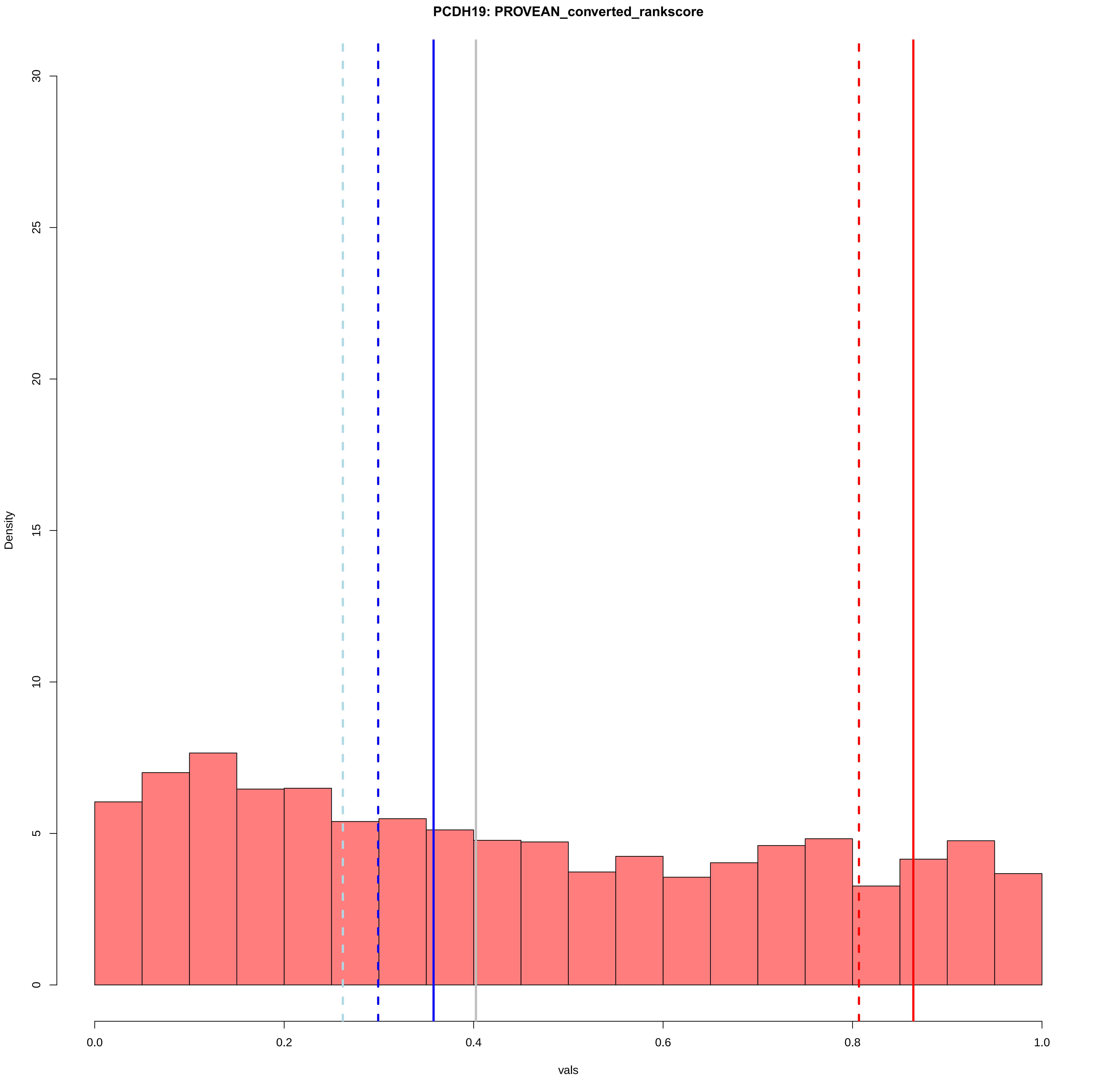

PCDH19: PROVEAN_converted_rankscore
Density
0
5
10
15
20
25
30
0.0
0.2
0.4
0.6
0.8
1.0
vals

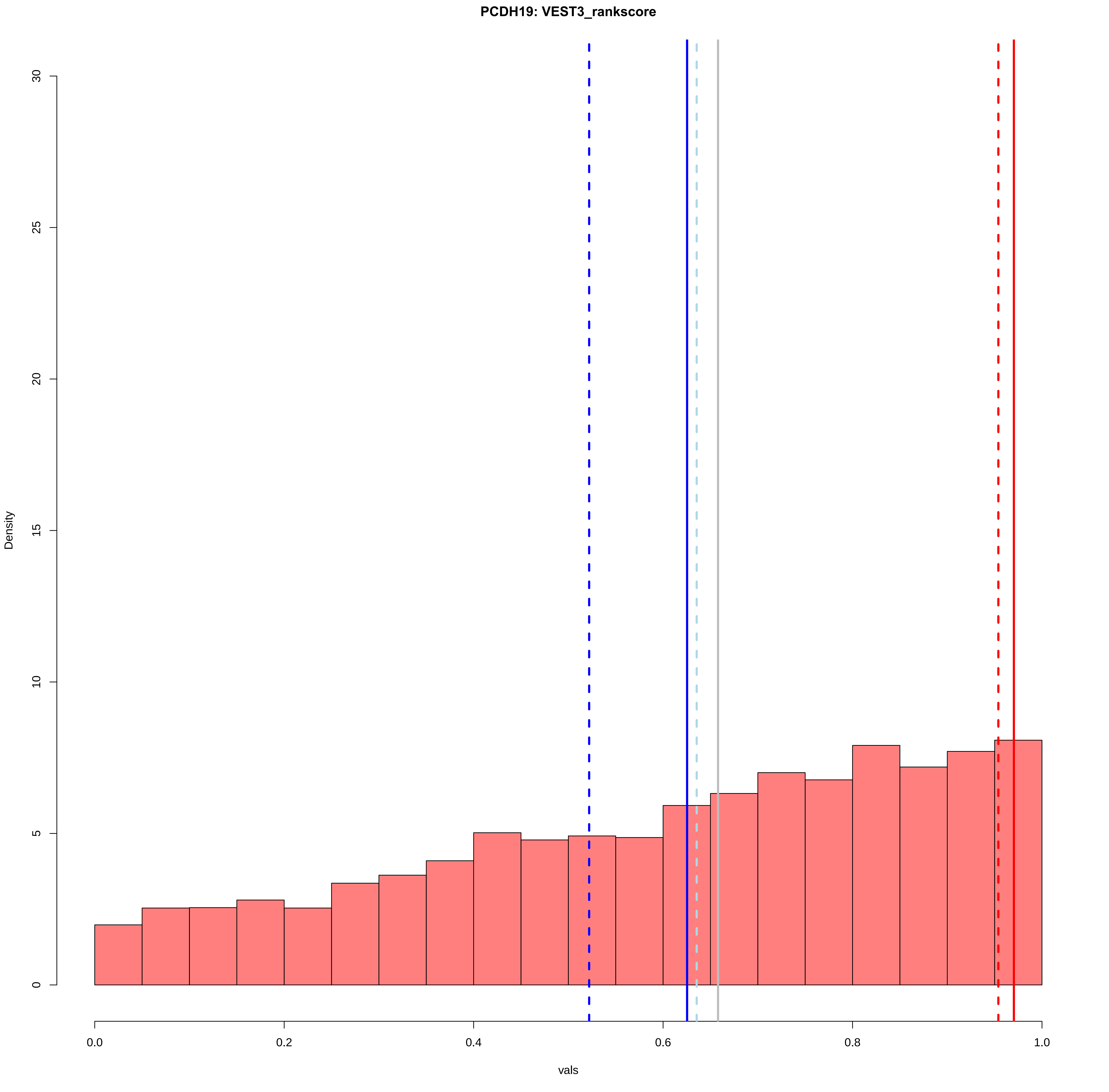

PCDH19: VEST3_rankscore
Density
vals
0
5
10
15
20
25
30
0.0
0.2
0.4
0.6
0.8
1.0

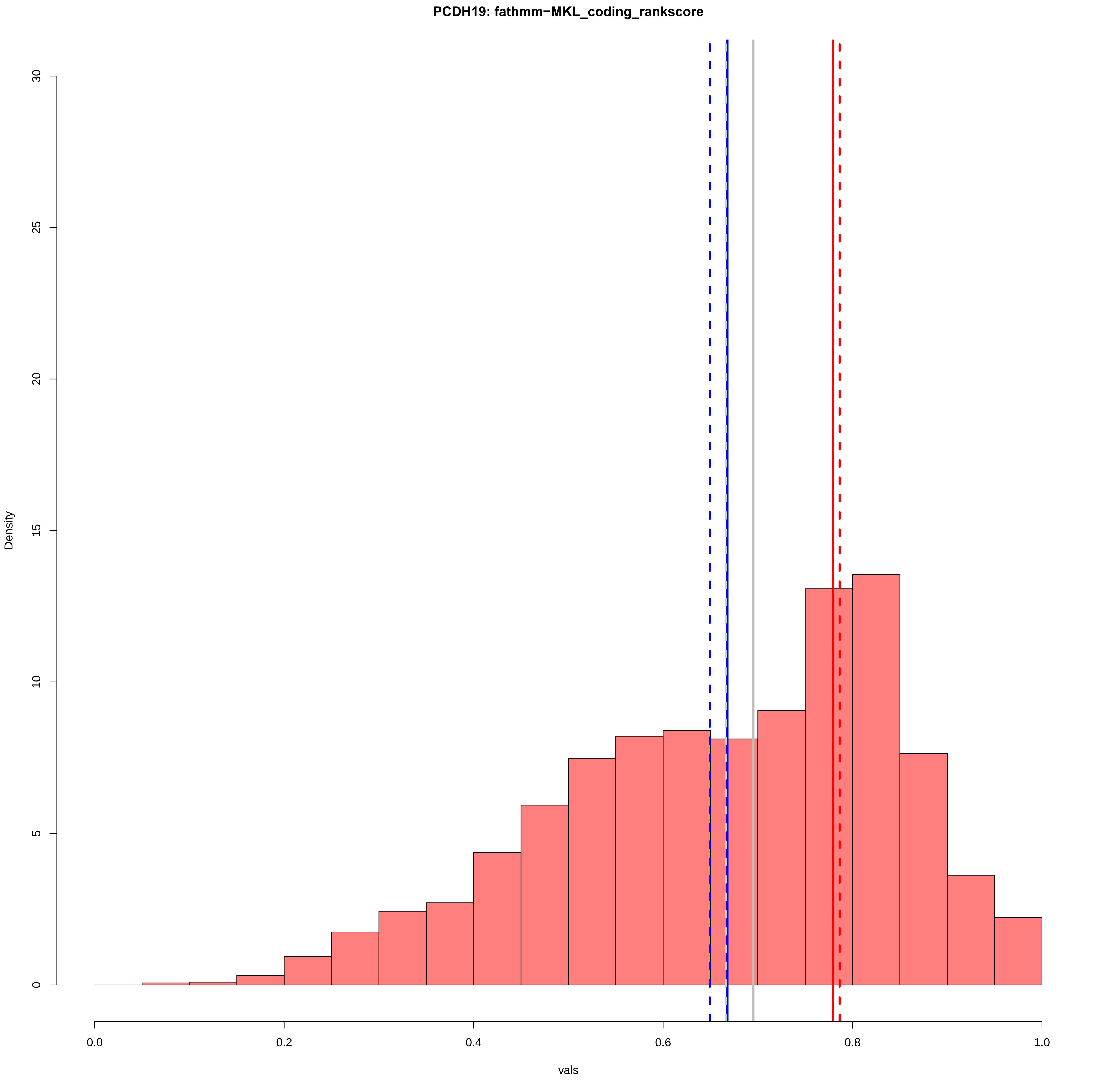

PCDH19: fathmm−MKL_coding_rankscore
Density
vals
0
5
10
15
20
25
30
0.0
0.2
0.4
0.6
0.8
1.0

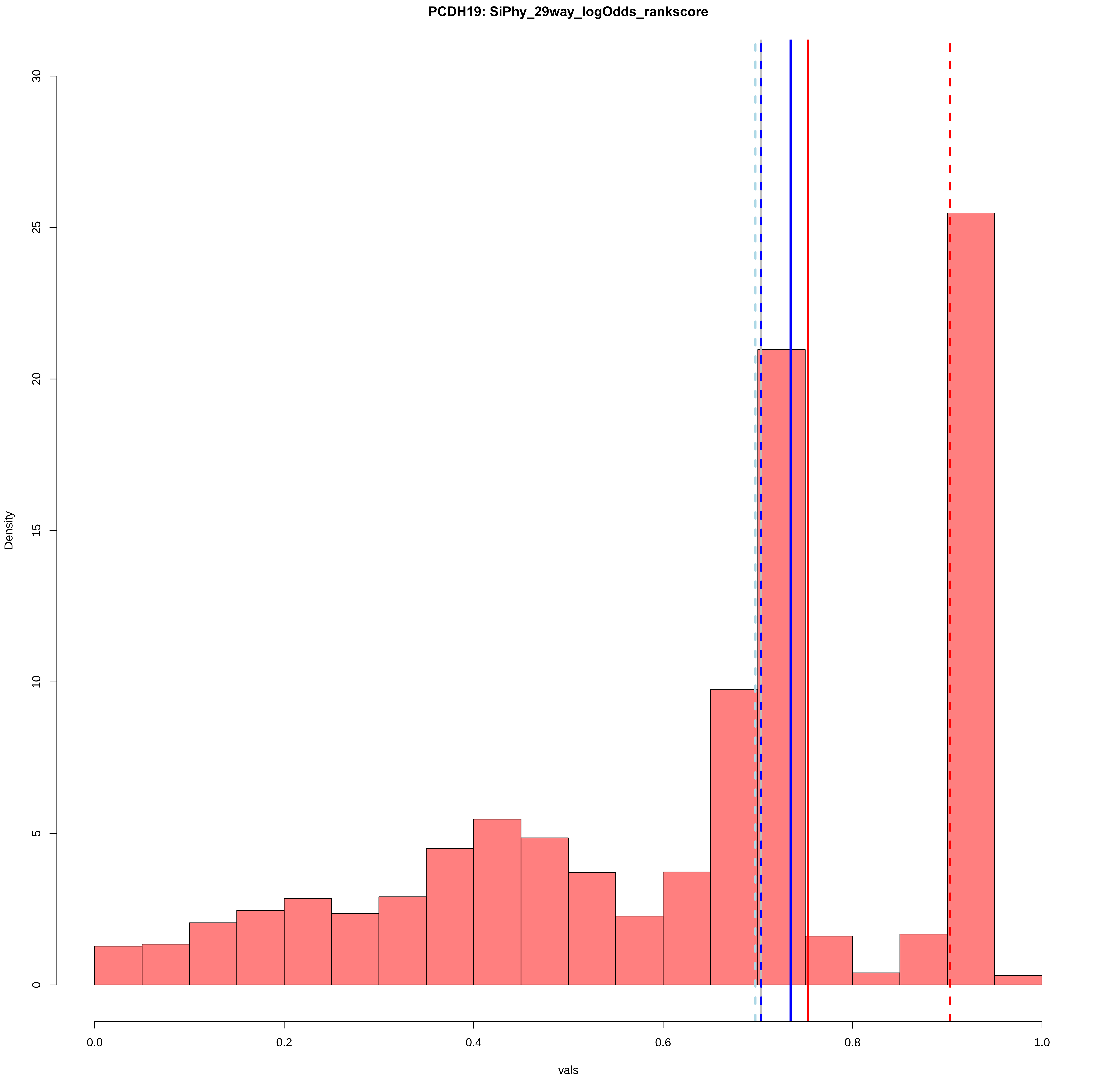
PCDH19: SiPhy_29way_logOdds_rankscore
Density
vals
0
5
10
15
20
25
30
0.0
0.2
0.4
0.6
0.8
1.0

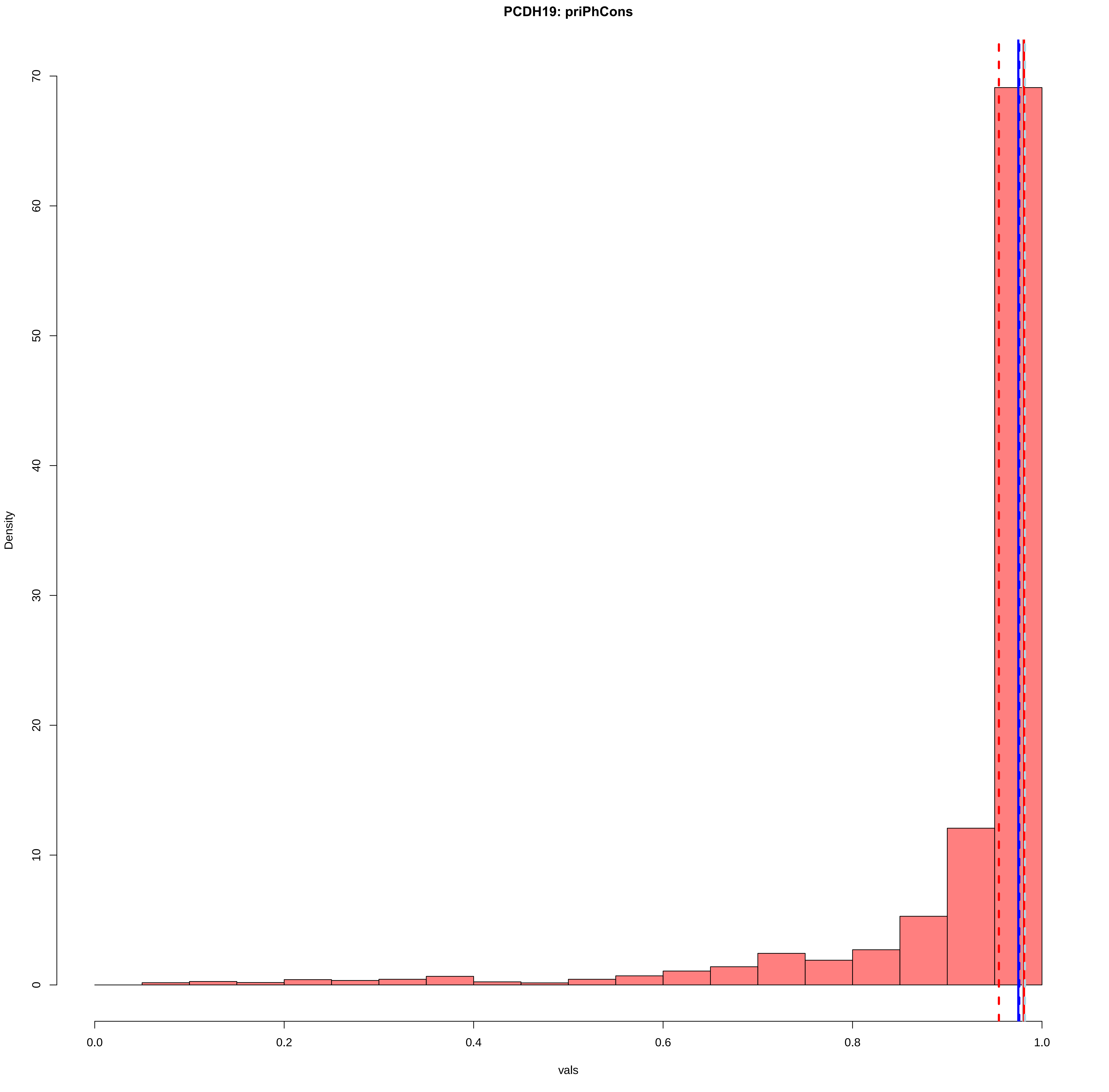
PCDH19: priPhCons
Density
vals
0
10
20
30
40
50
60
70
0.0
0.2
0.4
0.6
0.8
1.0

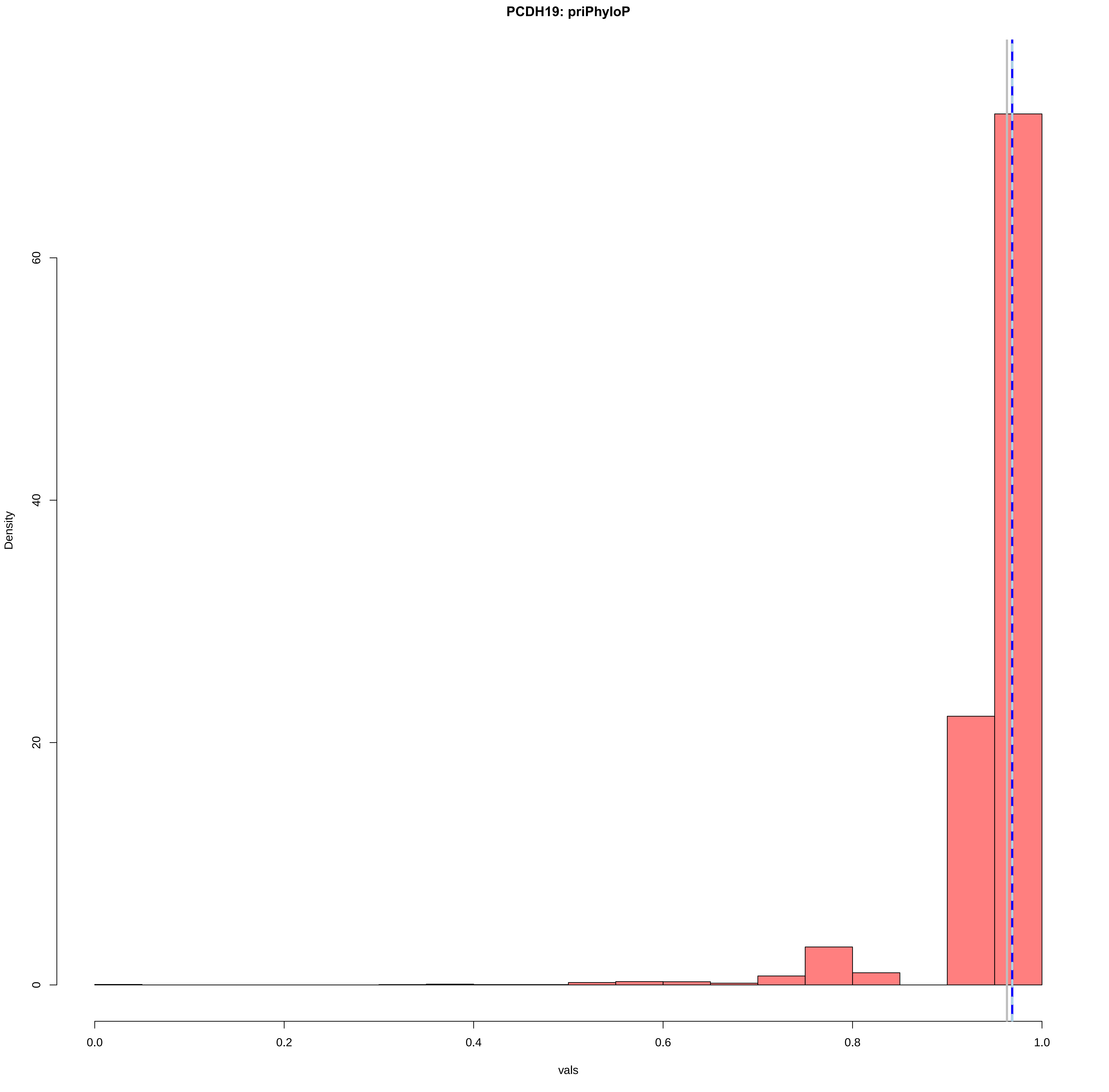
PCDH19: priPhyloP
Density
0
20
40
60
0.0
0.2
0.4
0.6
0.8
1.0
vals

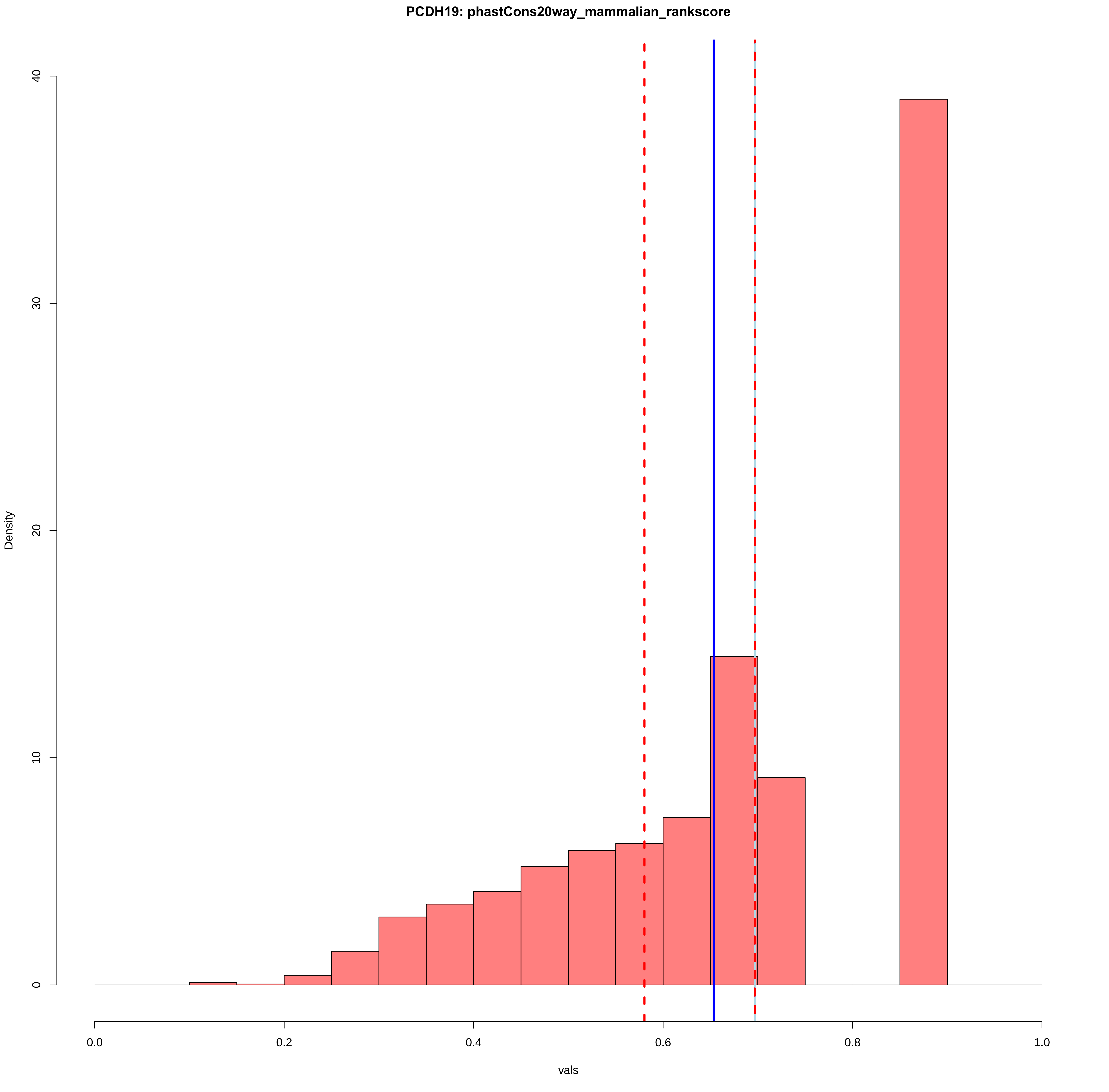
PCDH19: phastCons20way_mammalian_rankscore
Density
vals
0
10
20
30
40
0.0
0.2
0.4
0.6
0.8
1.0

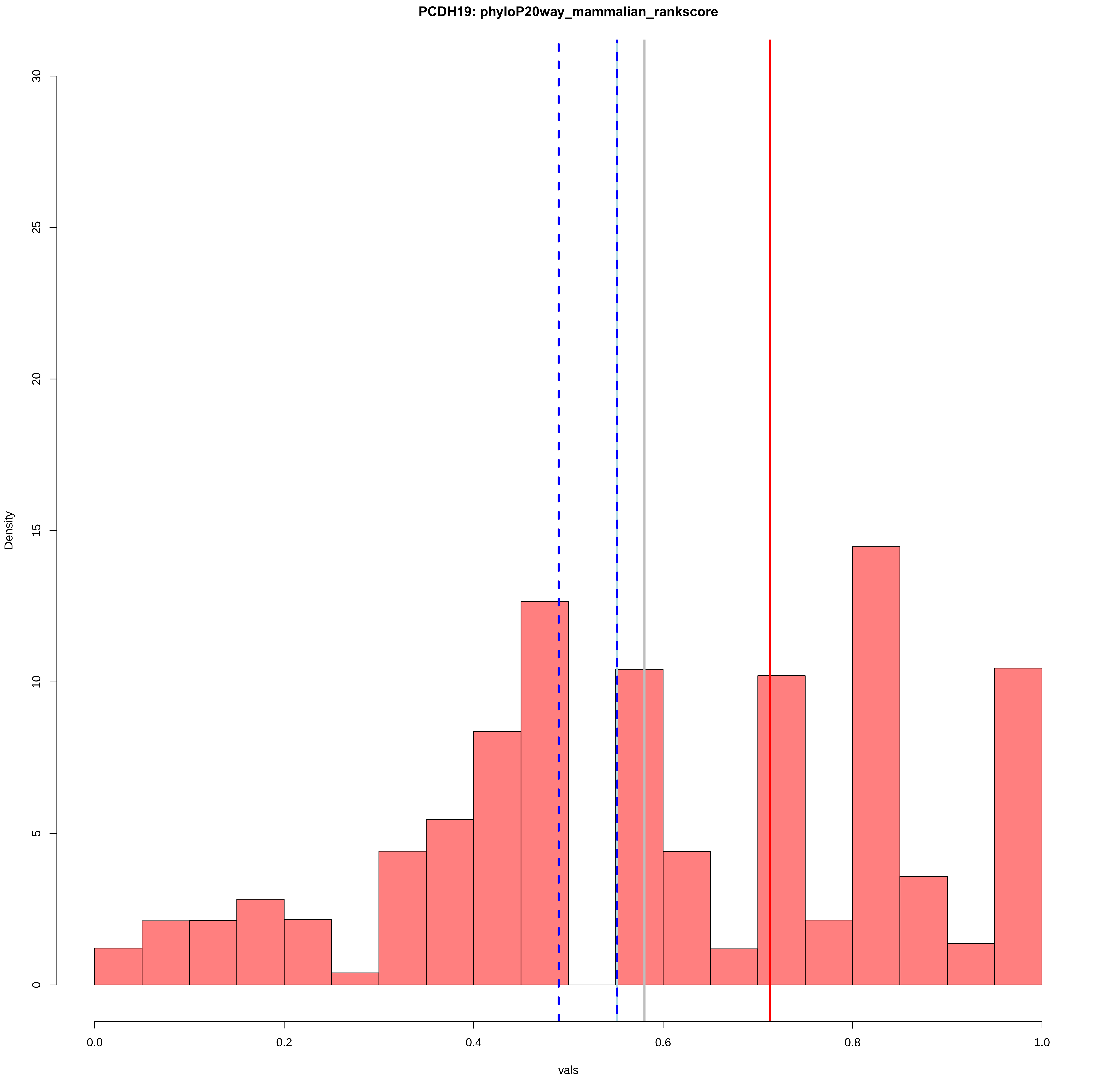

PCDH19: phyloP20way_mammalian_rankscore
Density
vals
0
5
10
15
20
25
30
0.0
0.2
0.4
0.6
0.8
1.0

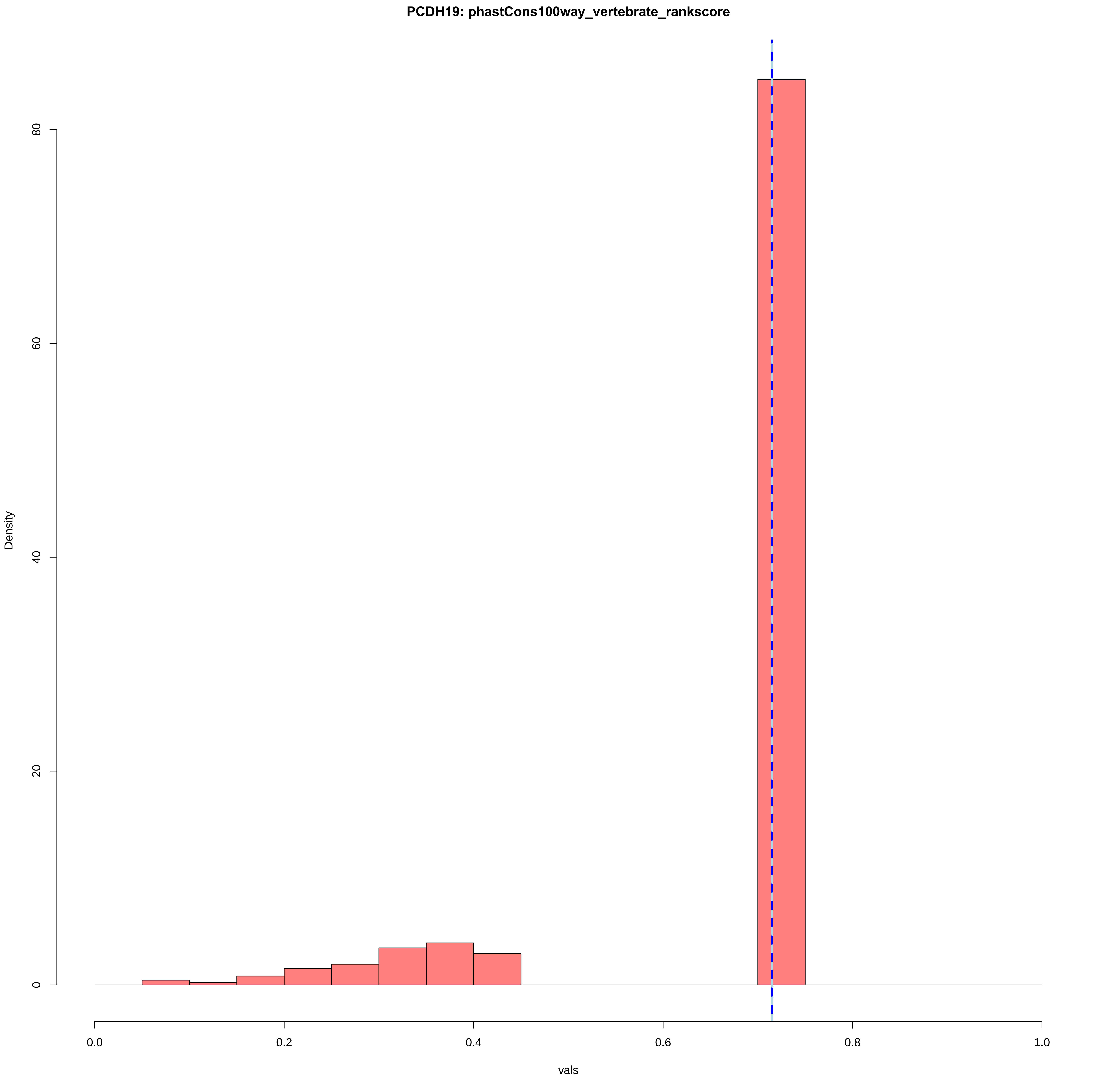
PCDH19: phastCons100way_vertebrate_rankscore
Density
vals
0
20
40
60
80
0.0
0.2
0.4
0.6
0.8
1.0

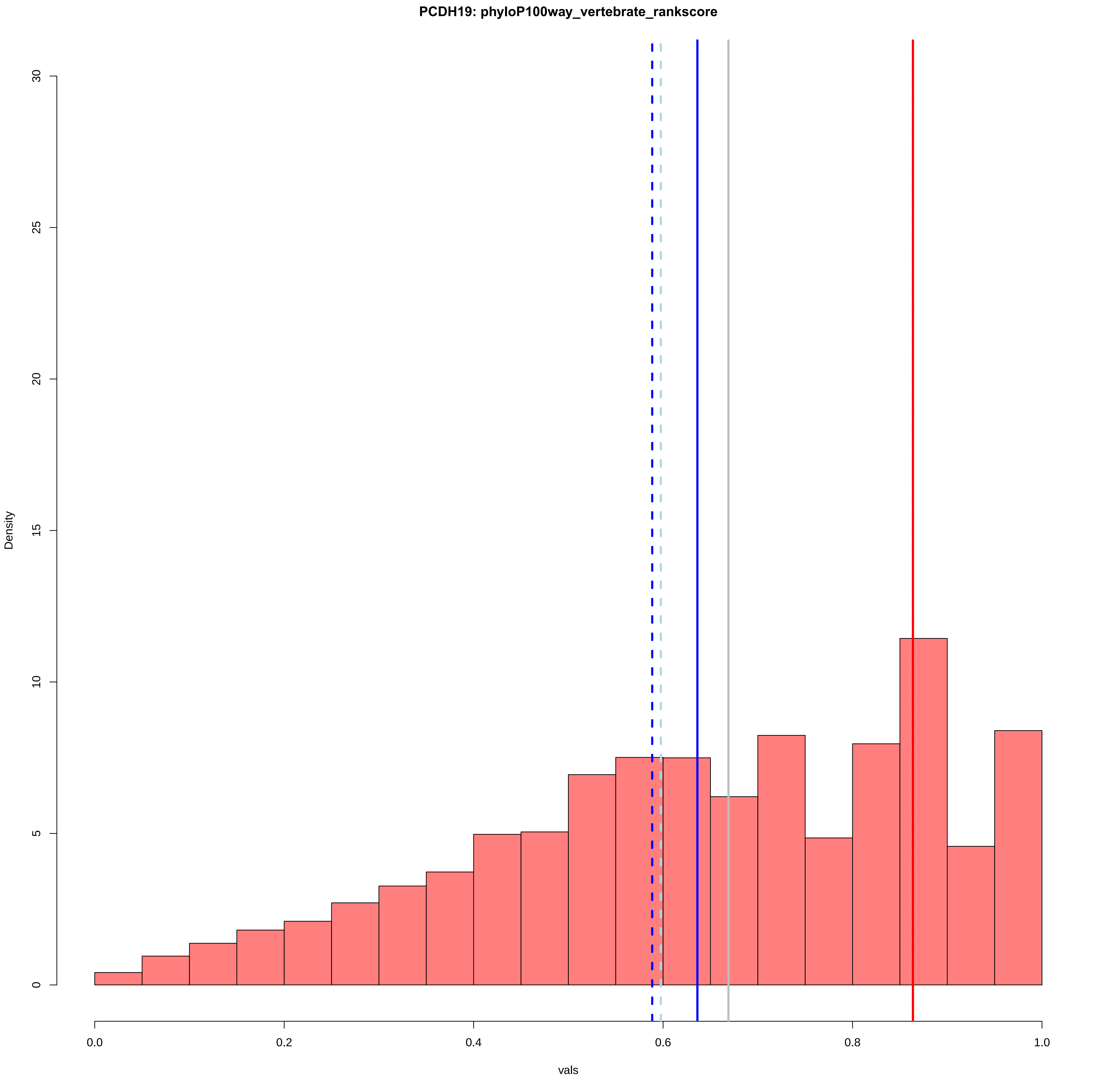

PCDH19: phyloP100way_vertebrate_rankscore
Density
0
5
10
15
20
25
30
0.0
0.2
0.4
0.6
0.8
1.0
vals

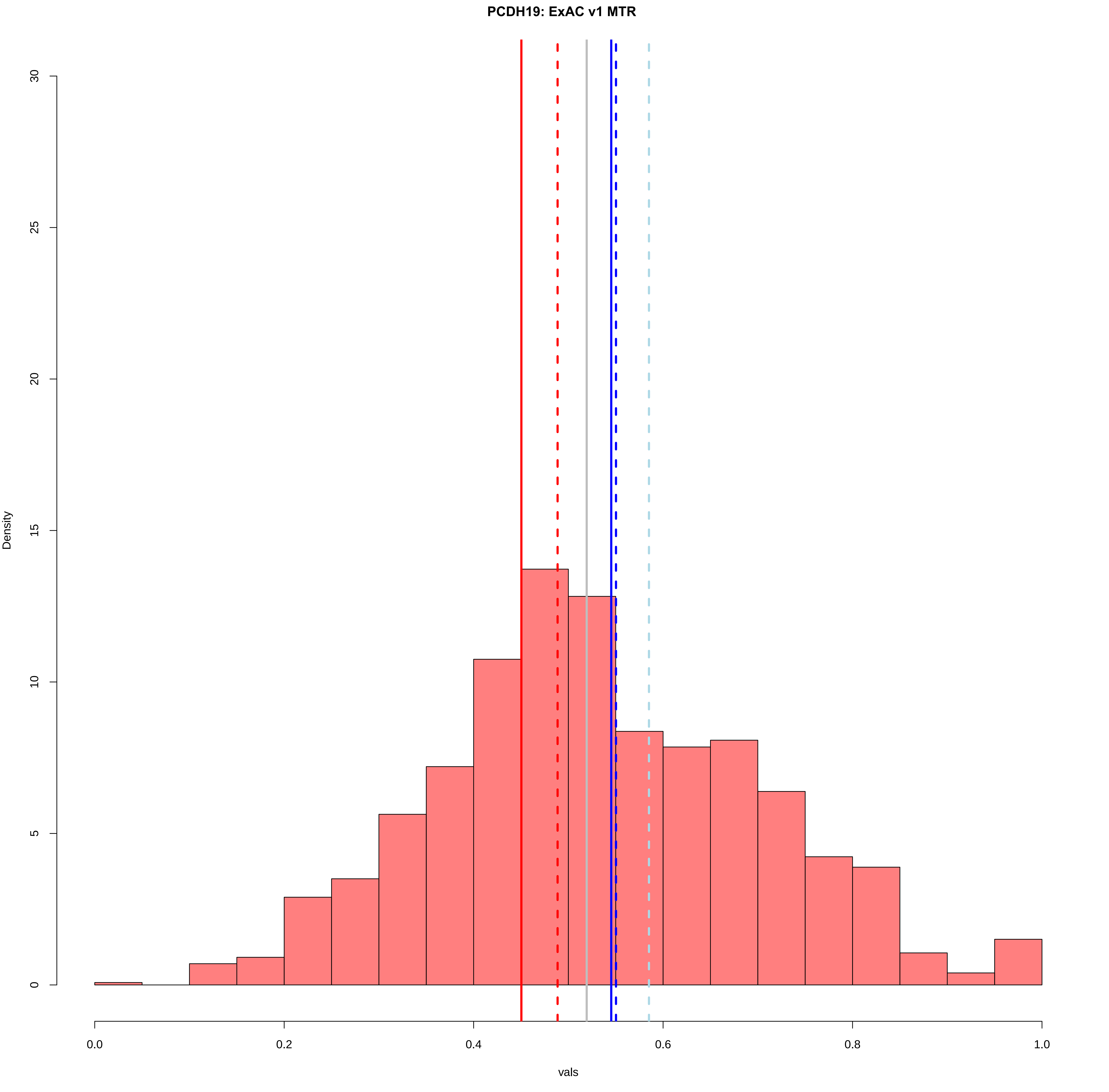
PCDH19: ExAC v1 MTR
Density
vals
0
5
10
15
20
25
30
0.0
0.2
0.4
0.6
0.8
1.0

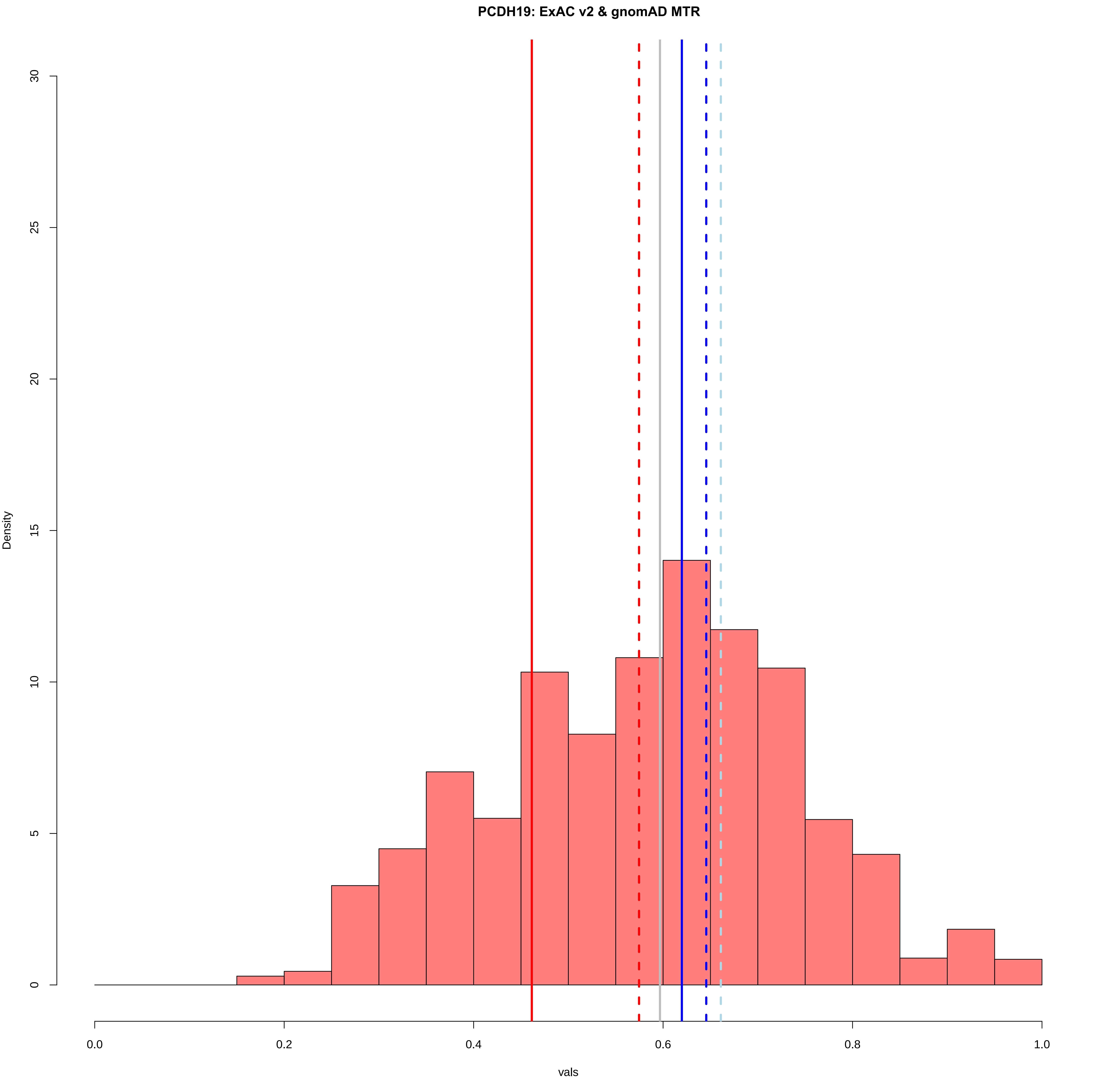
PCDH19: ExAC v2 & gnomAD MTR
Density
vals
0
5
10
15
20
25
30
0.0
0.2
0.4
0.6
0.8
1.0

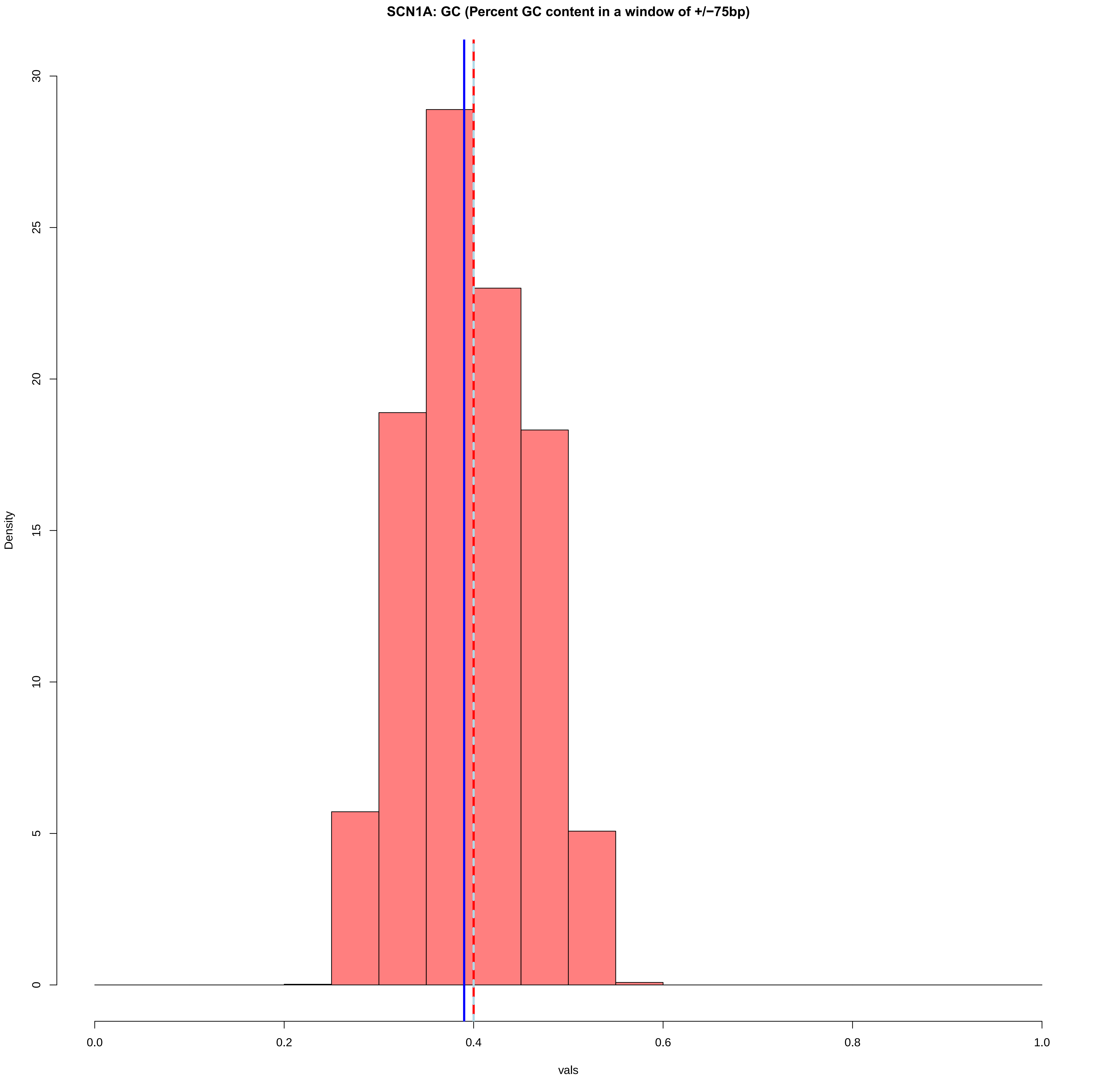

SCN1A: GC (Percent GC content in a window of +/−75bp)
Density
0
5
10
15
20
25
30
0.0
0.2
0.4
0.6
0.8
1.0
vals

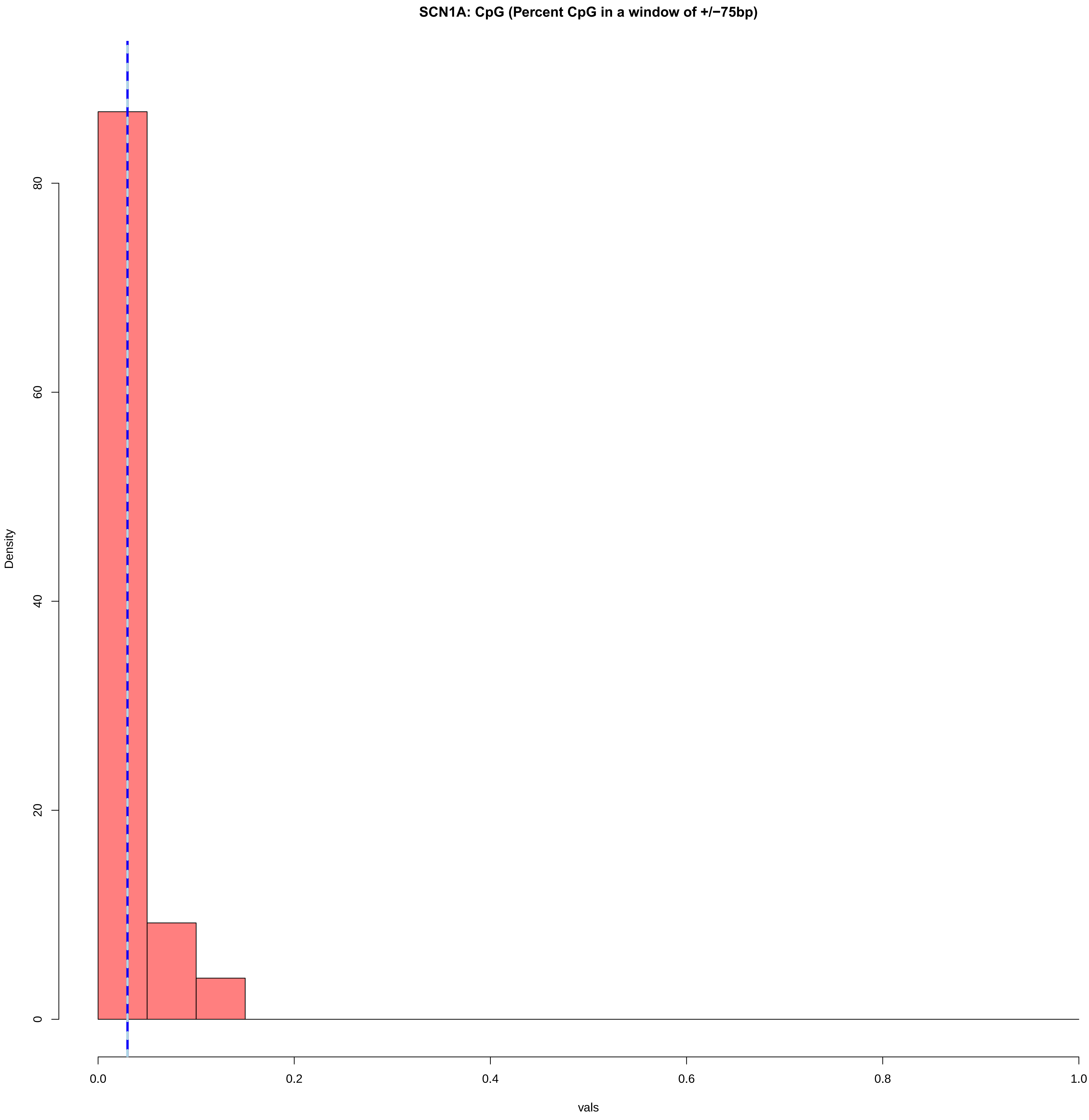
SCN1A: CpG (Percent CpG in a window of +/−75bp)
Density
0
20
40
60
80
vals
0.0
0.2
0.4
0.6
0.8
1.0

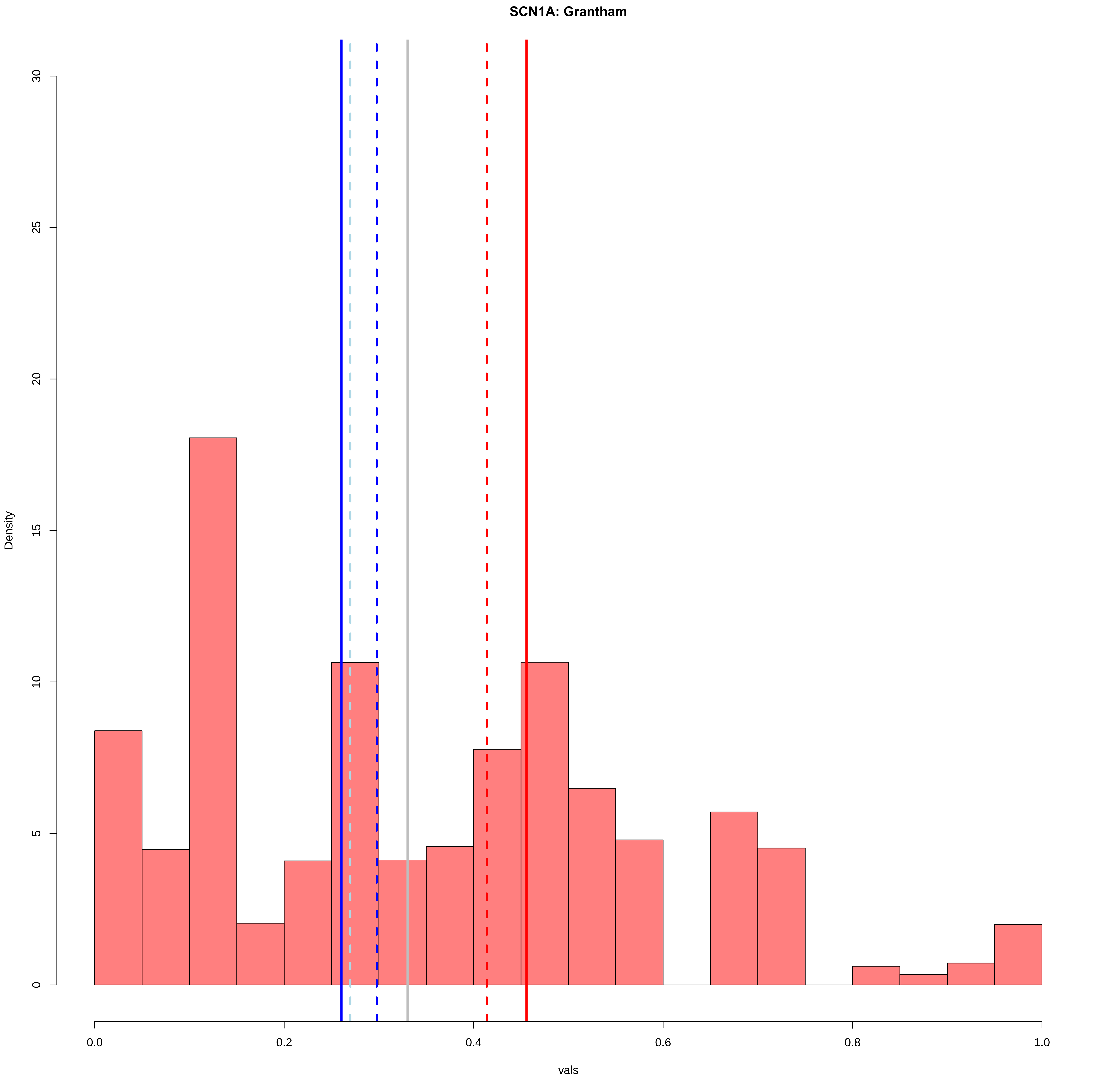
SCN1A: Grantham
Density
vals
0
5
10
15
20
25
30
0.0
0.2
0.4
0.6
0.8
1.0

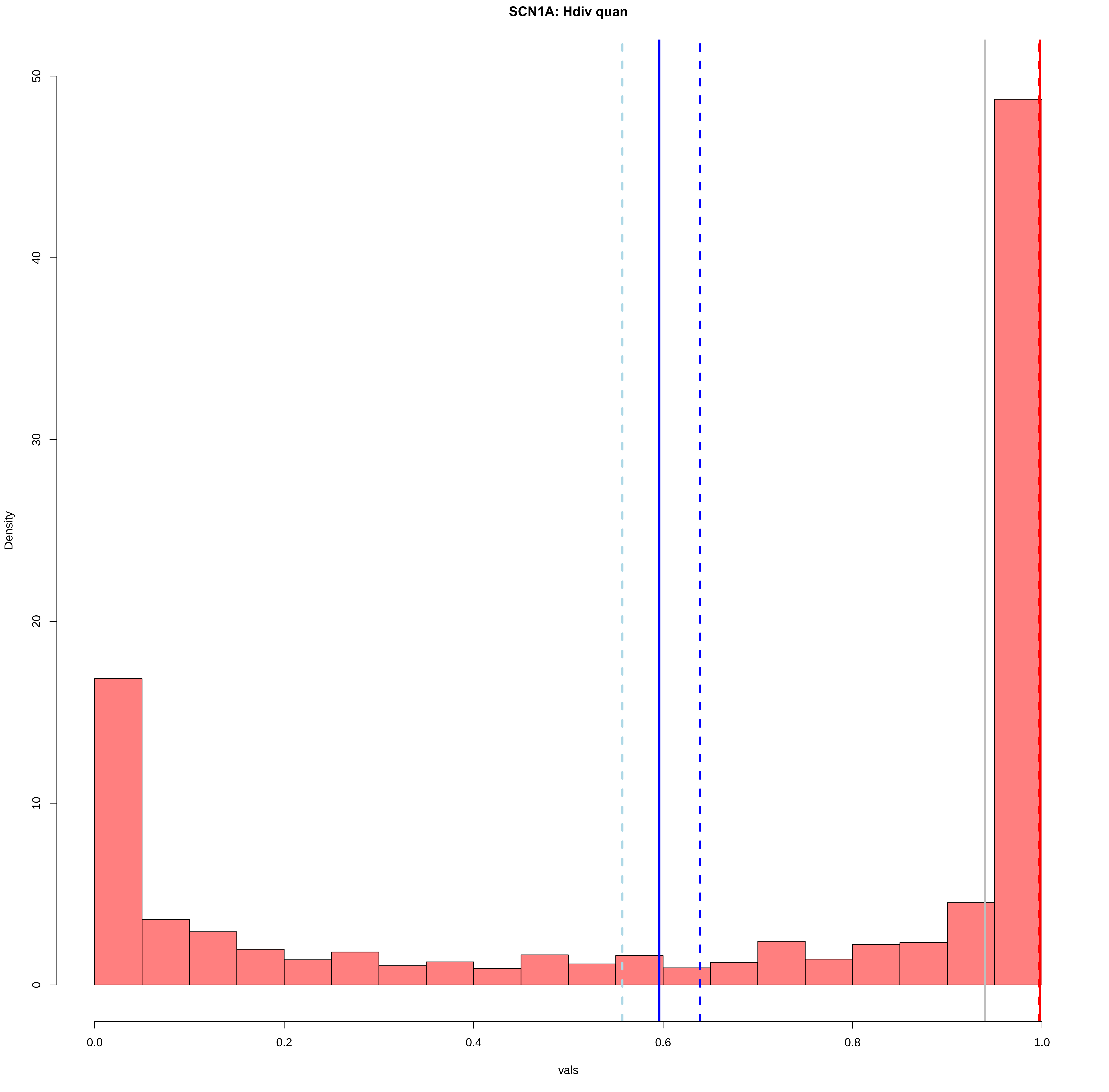
SCN1A: Hdiv quan
Density
vals
0
10
20
30
40
50
0.0
0.2
0.4
0.6
0.8
1.0

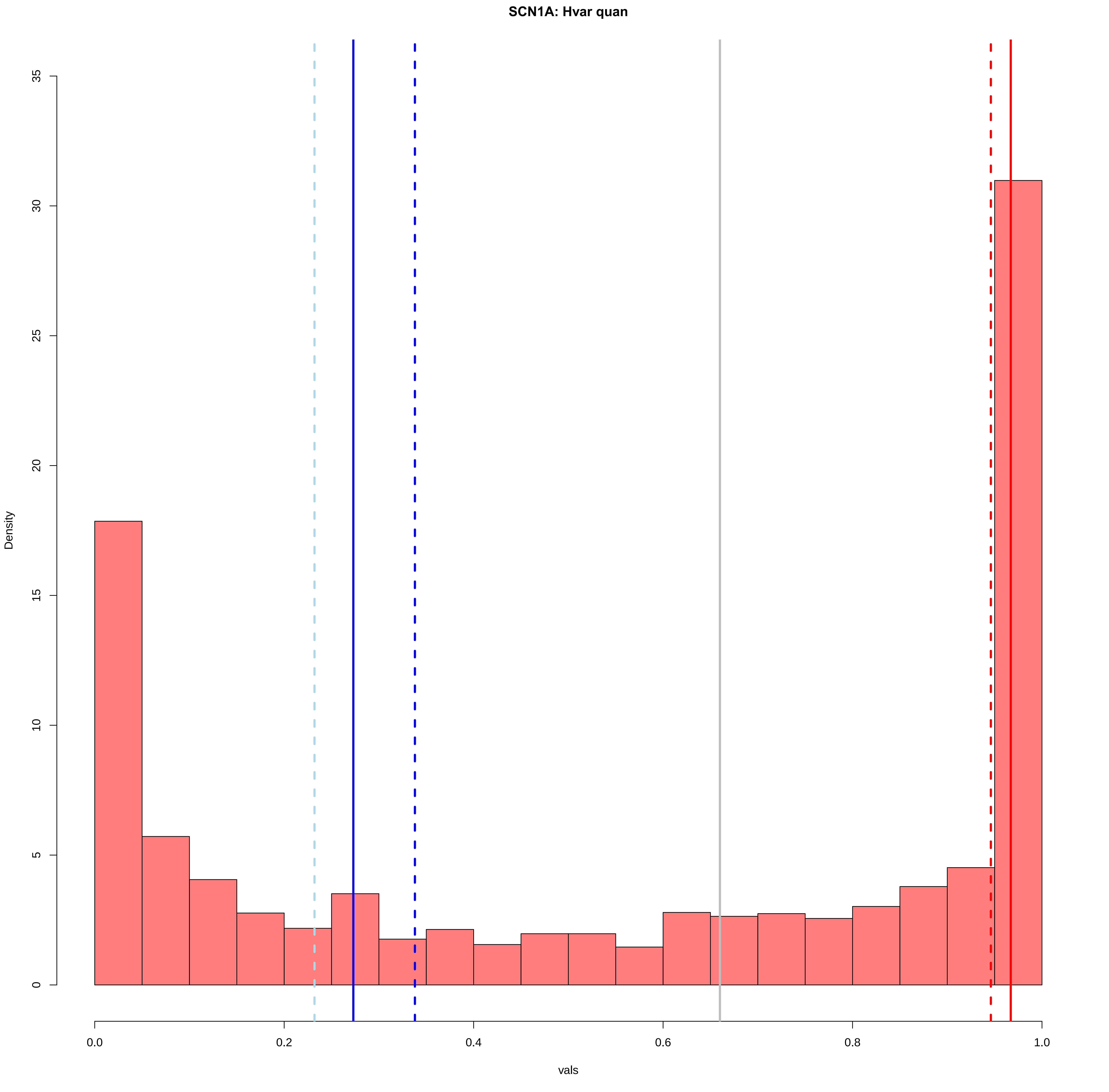

SCN1A: Hvar quan
Density
0
5
10
15
20
25
30
35
0.0
0.2
0.4
0.6
0.8
1.0
vals

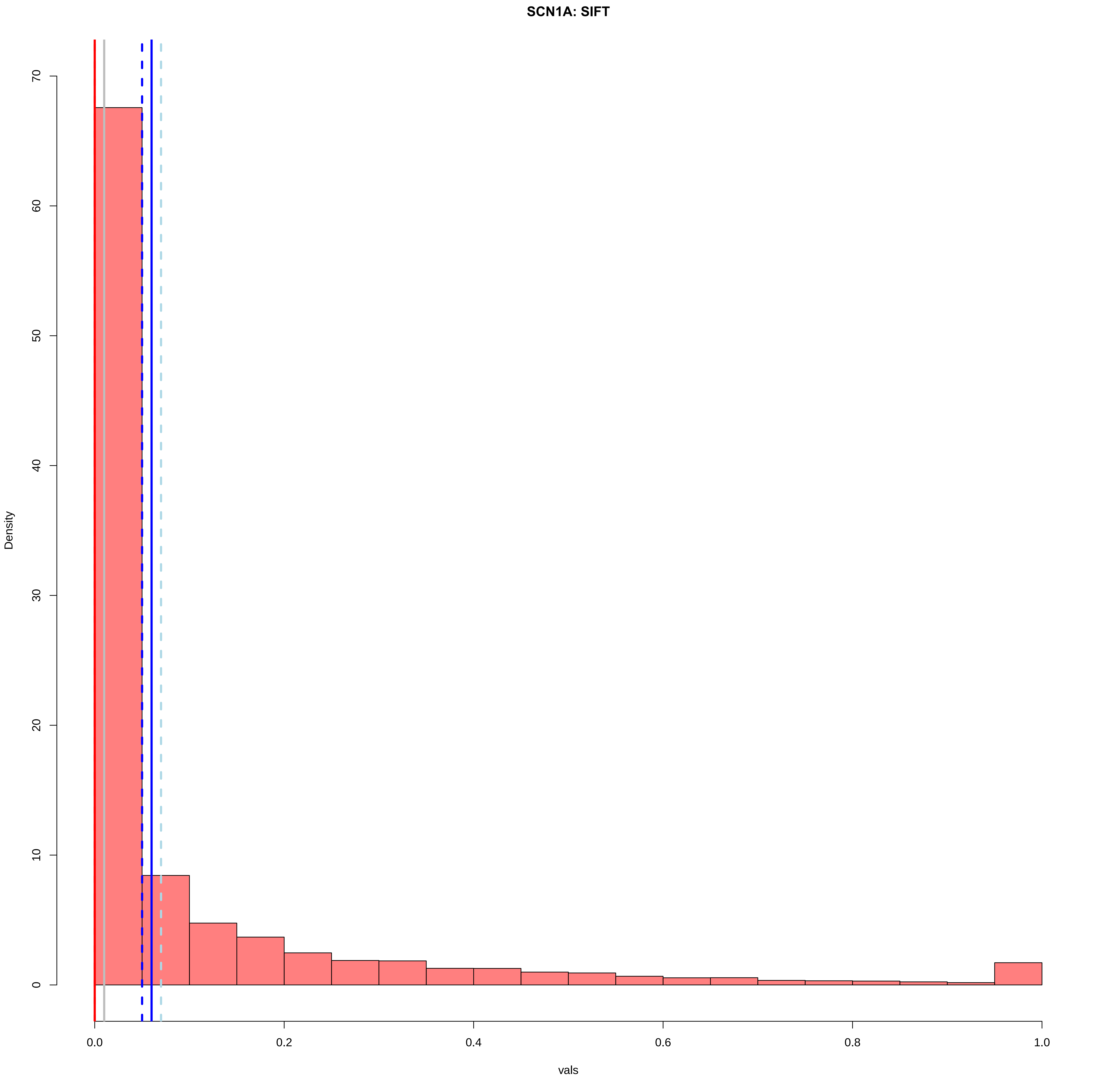
SCN1A: SIFT
Density
vals
0
10
20
30
40
50
60
70
0.0
0.2
0.4
0.6
0.8
1.0

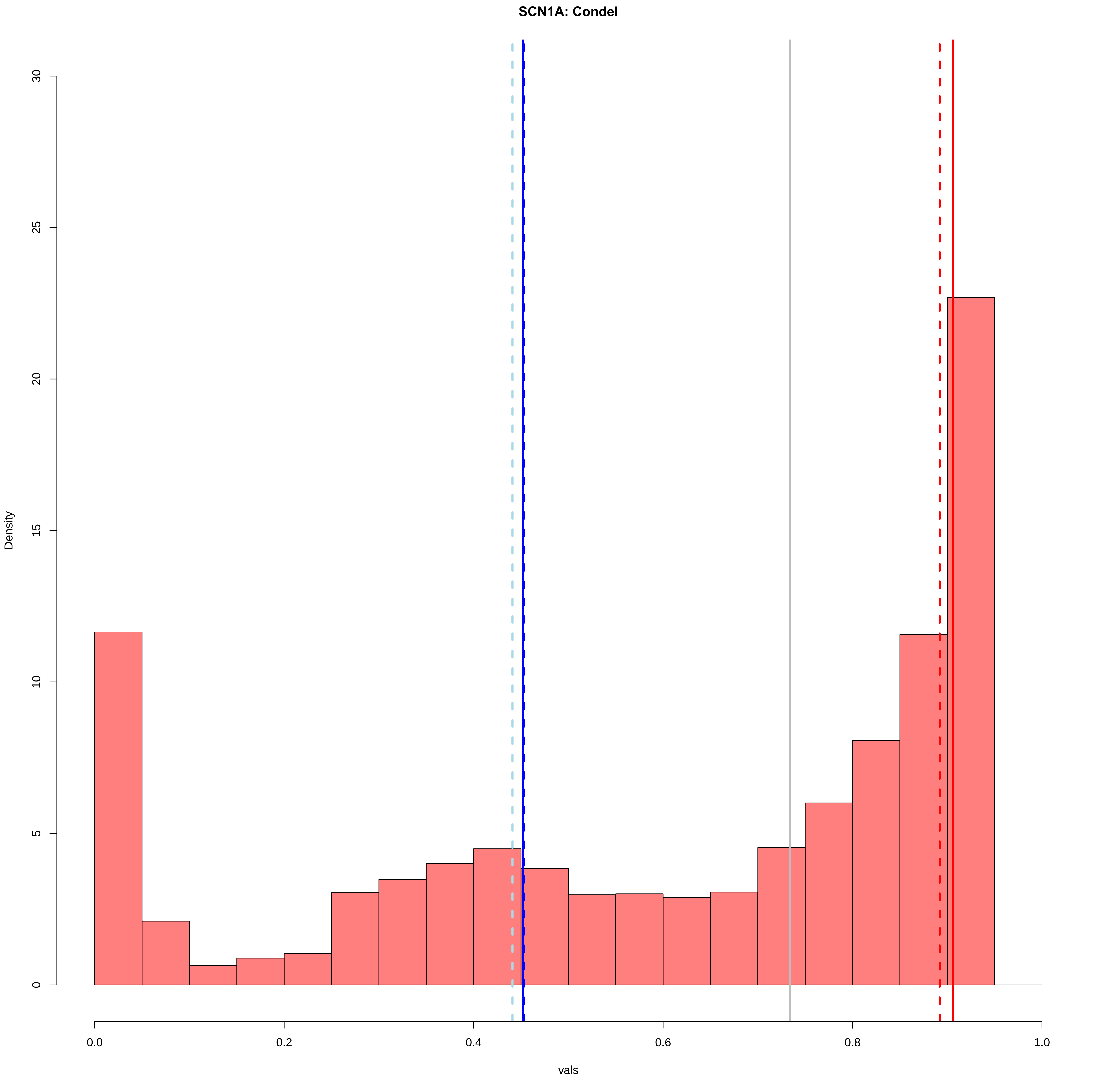
SCN1A: Condel
Density
vals
0
5
10
15
20
25
30
0.0
0.2
0.4
0.6
0.8
1.0

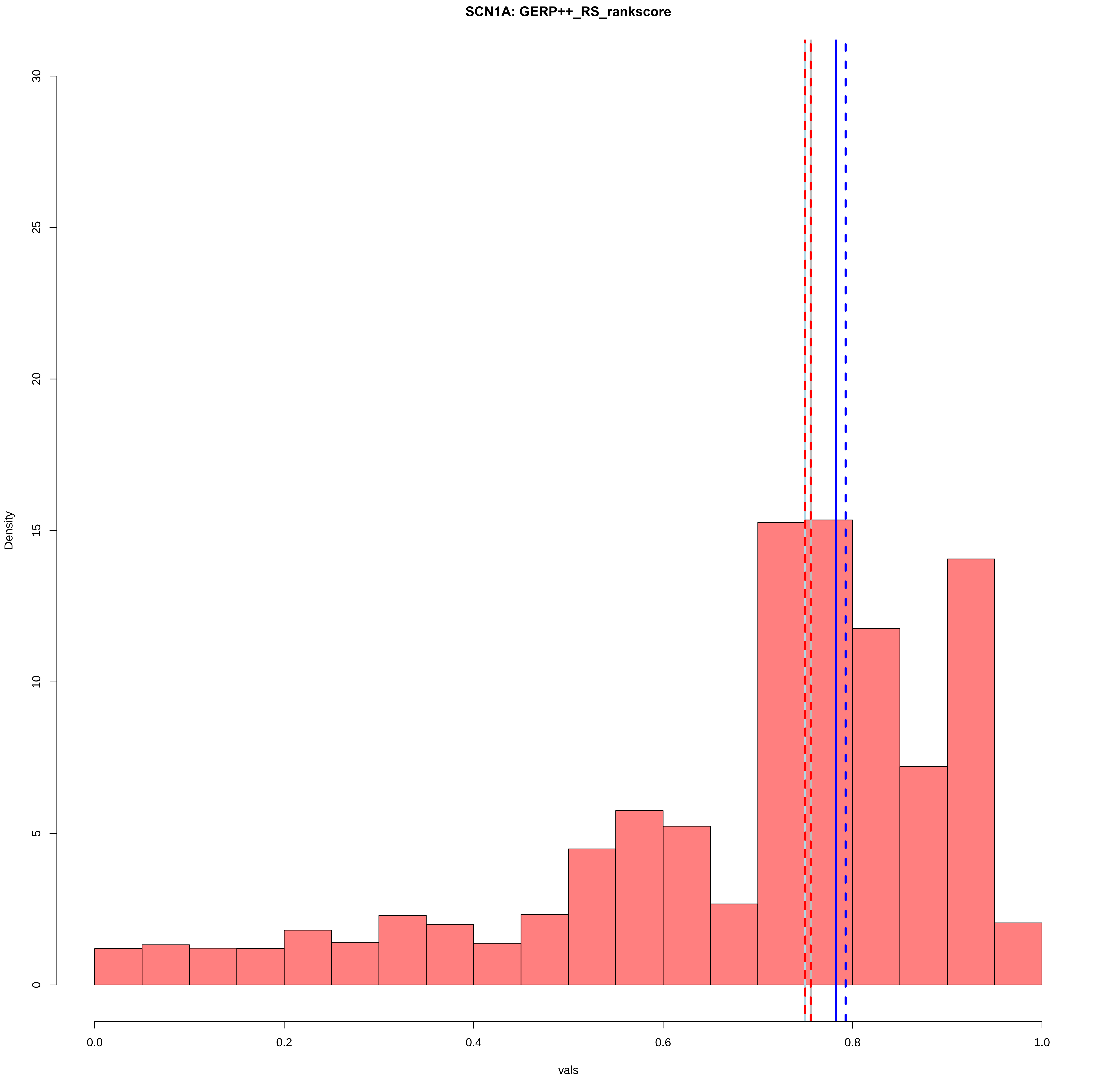
SCN1A: GERP++_RS_rankscore
Density
0
5
10
15
20
25
30
0.0
0.2
0.4
0.6
0.8
1.0
vals

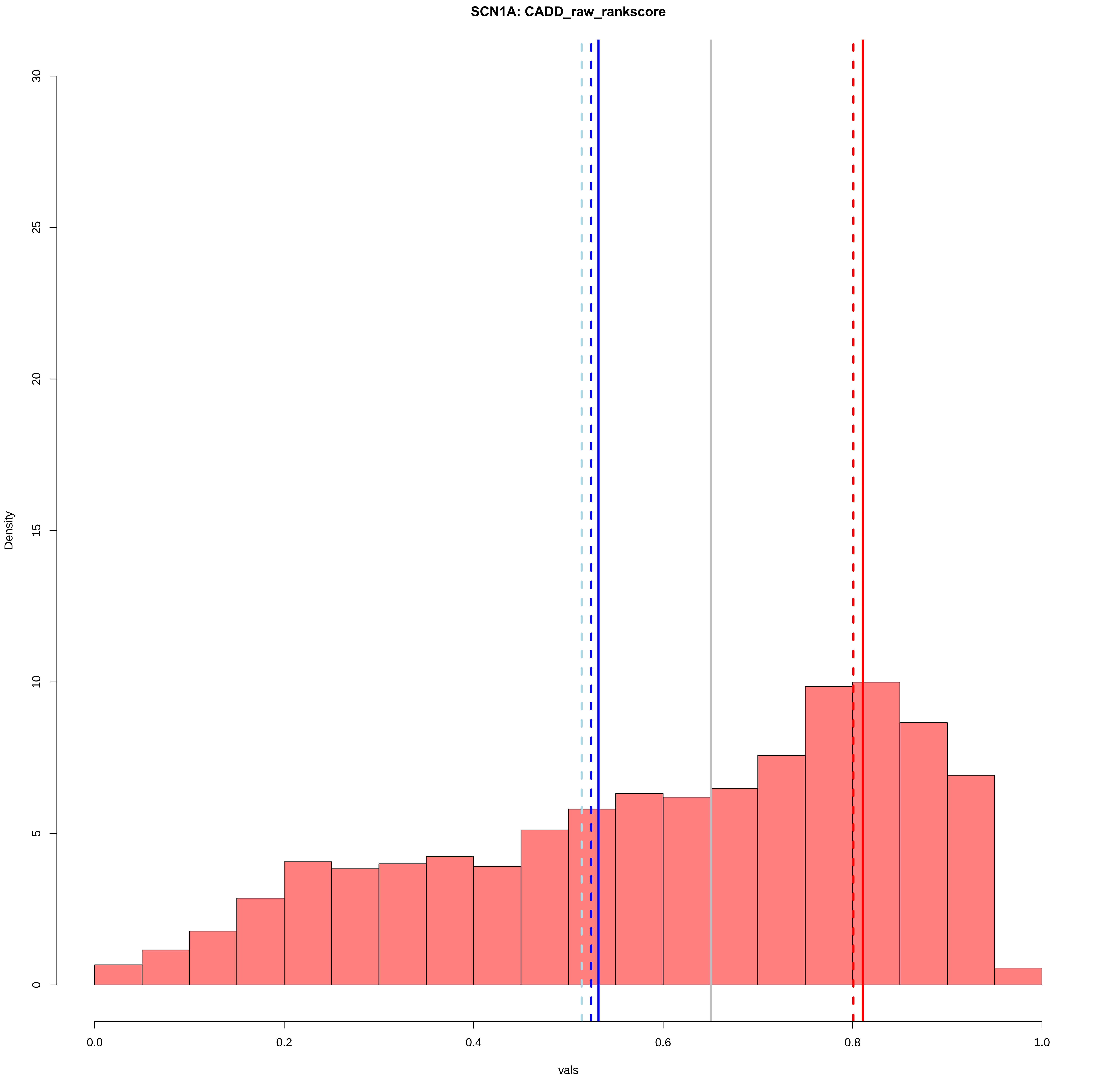

SCN1A: CADD_raw_rankscore
Density
vals
0
5
10
15
20
25
30
0.0
0.2
0.4
0.6
0.8
1.0

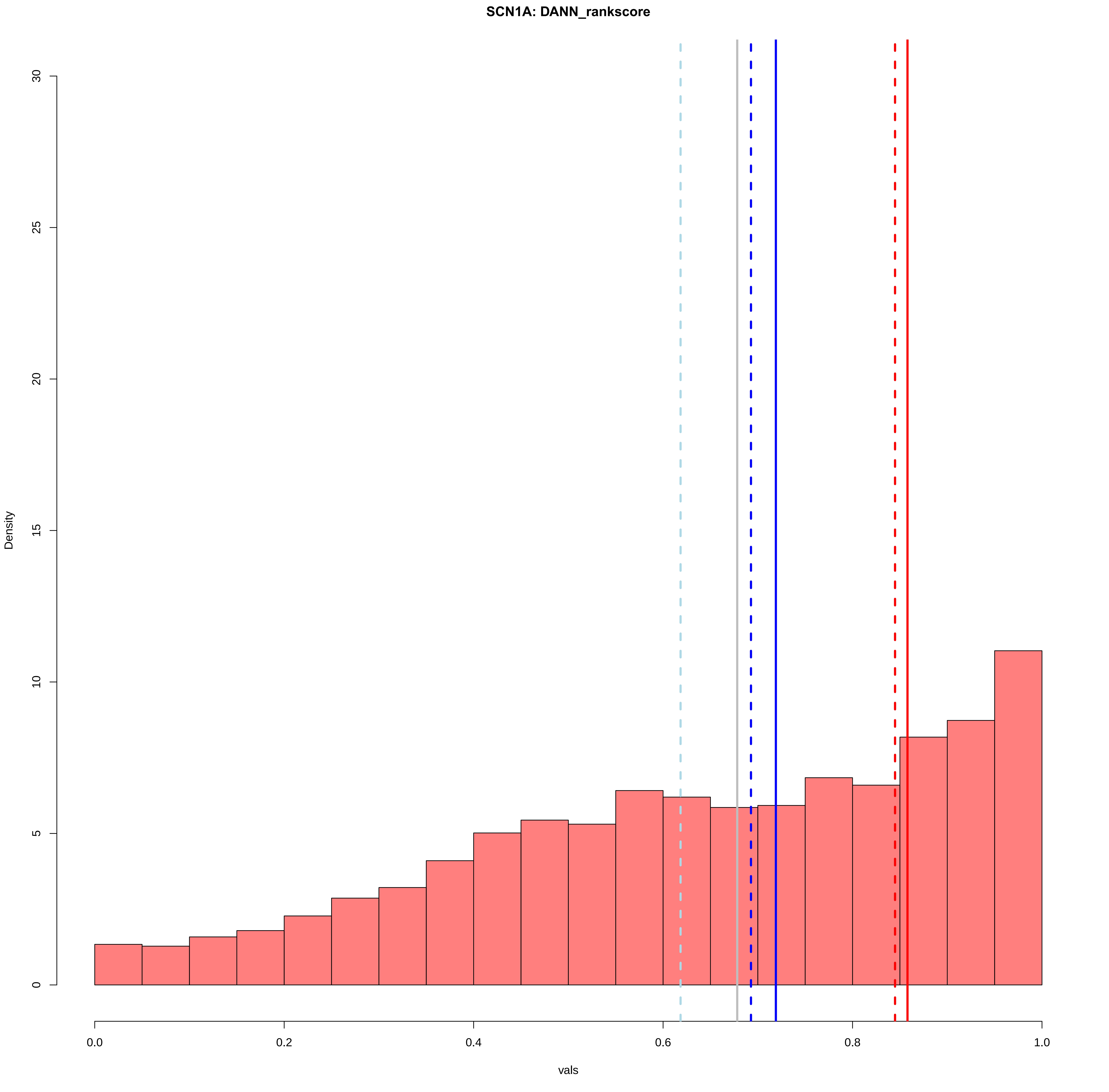
SCN1A: DANN_rankscore
Density
vals
0
5
10
15
20
25
30
0.0
0.2
0.4
0.6
0.8
1.0

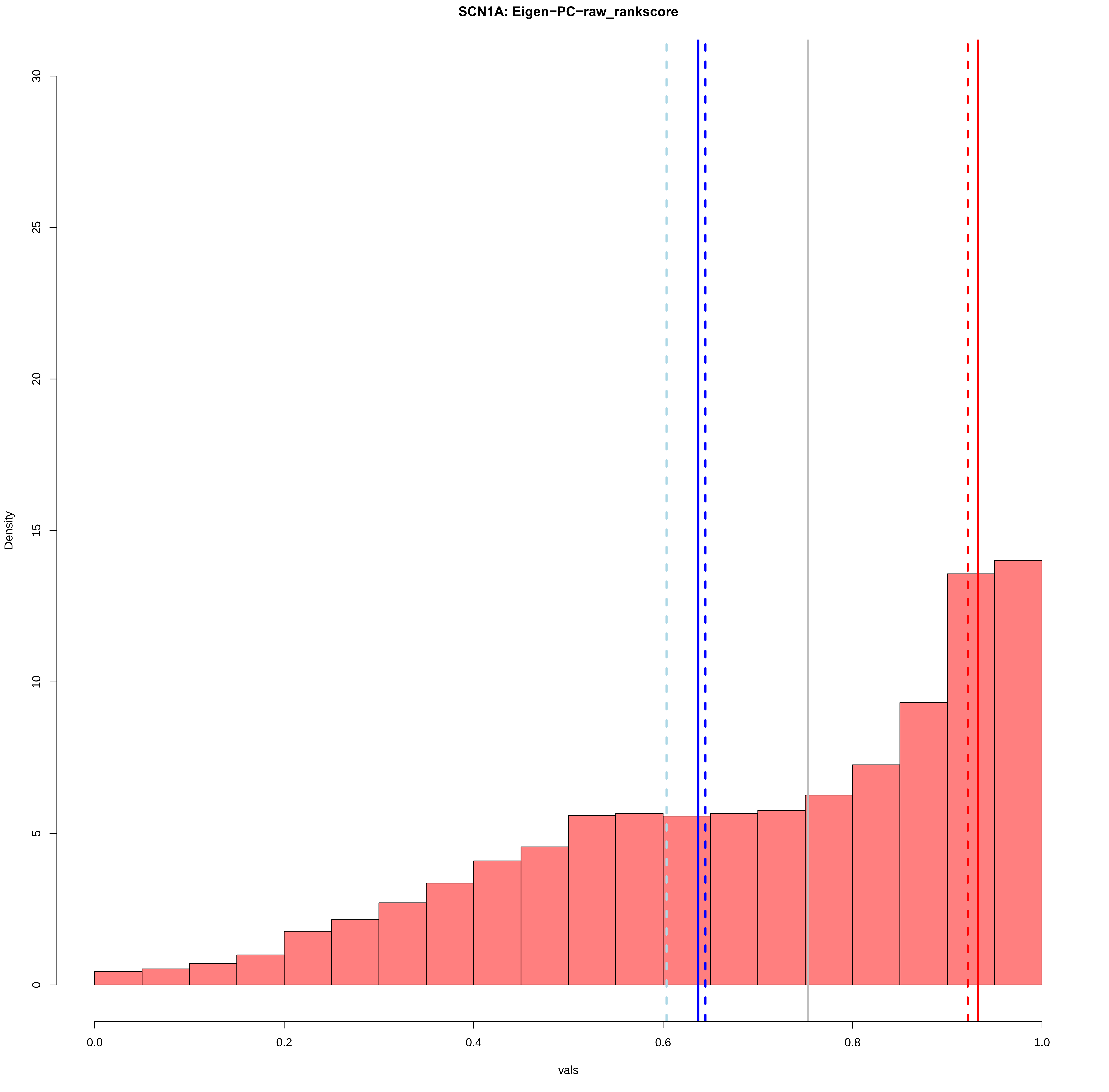

SCN1A: Eigen−PC−raw_rankscore
Density
vals
0
5
10
15
20
25
30
0.0
0.2
0.4
0.6
0.8
1.0

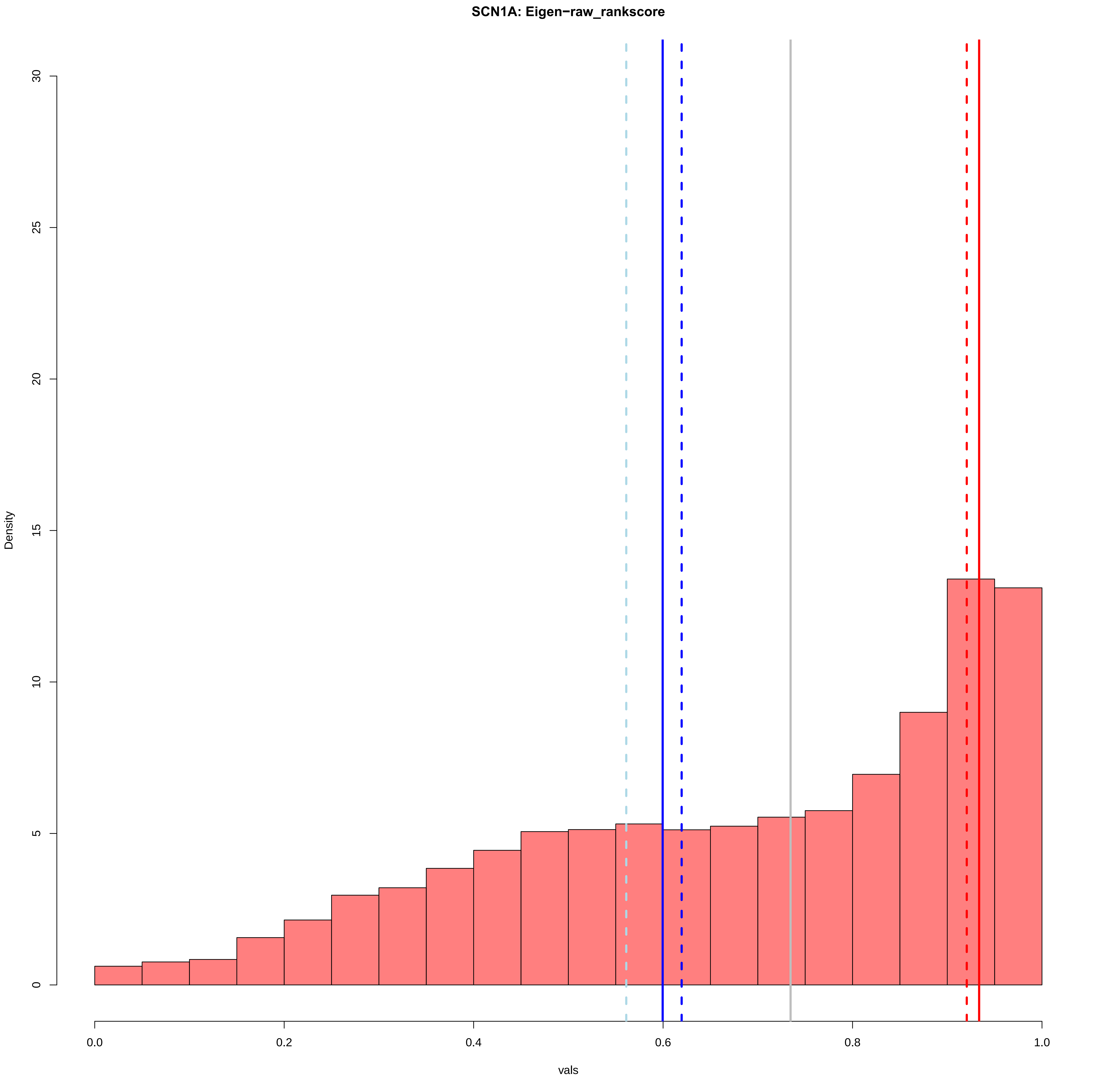

SCN1A: Eigen−raw_rankscore
Density
vals
0
5
10
15
20
25
30
0.0
0.2
0.4
0.6
0.8
1.0

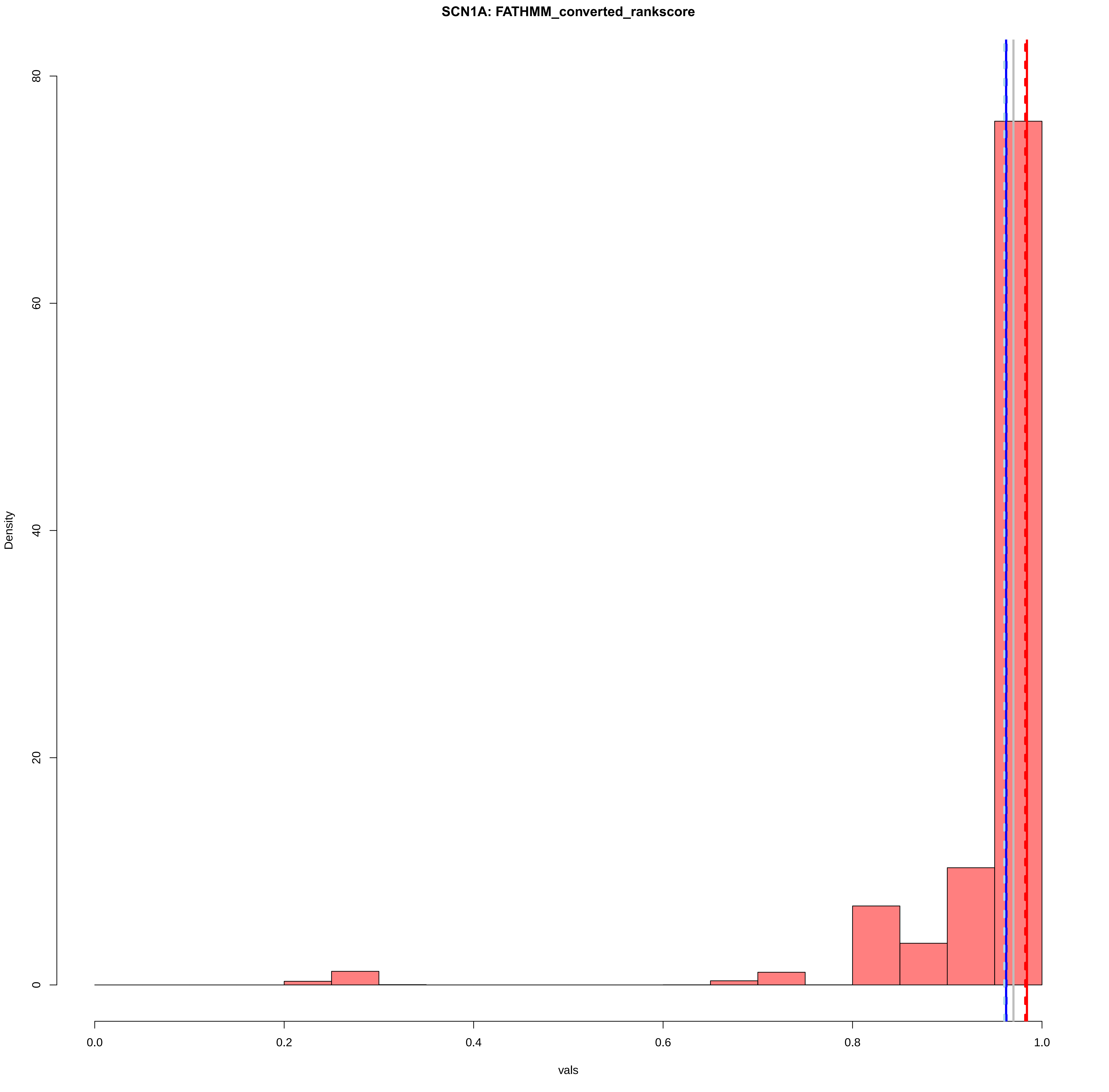

SCN1A: FATHMM_converted_rankscore
Density
0
20
40
60
80
0.0
0.2
0.4
0.6
0.8
1.0
vals

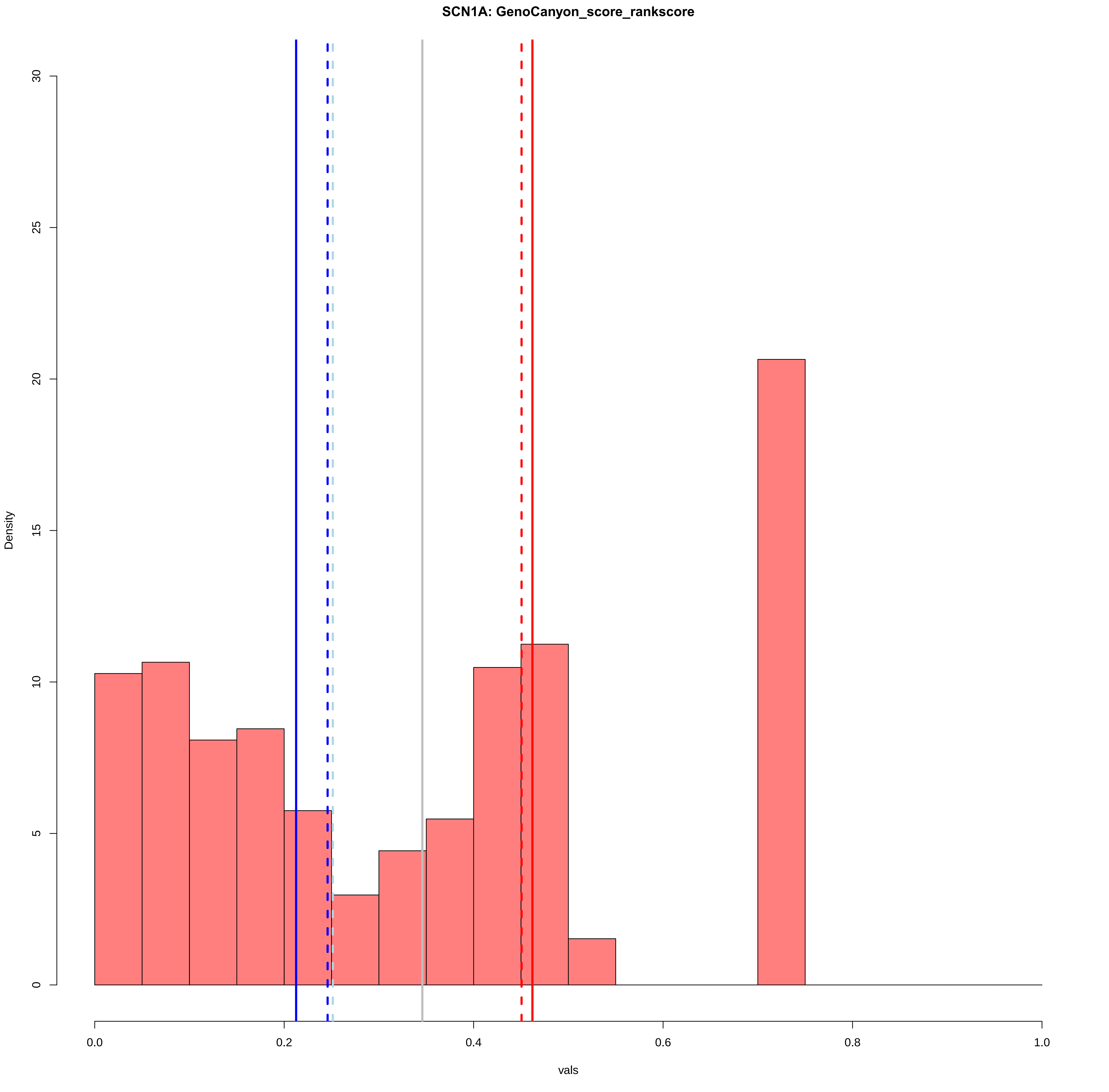

SCN1A: GenoCanyon_score_rankscore
Density
vals
0
5
10
15
20
25
30
0.0
0.2
0.4
0.6
0.8
1.0

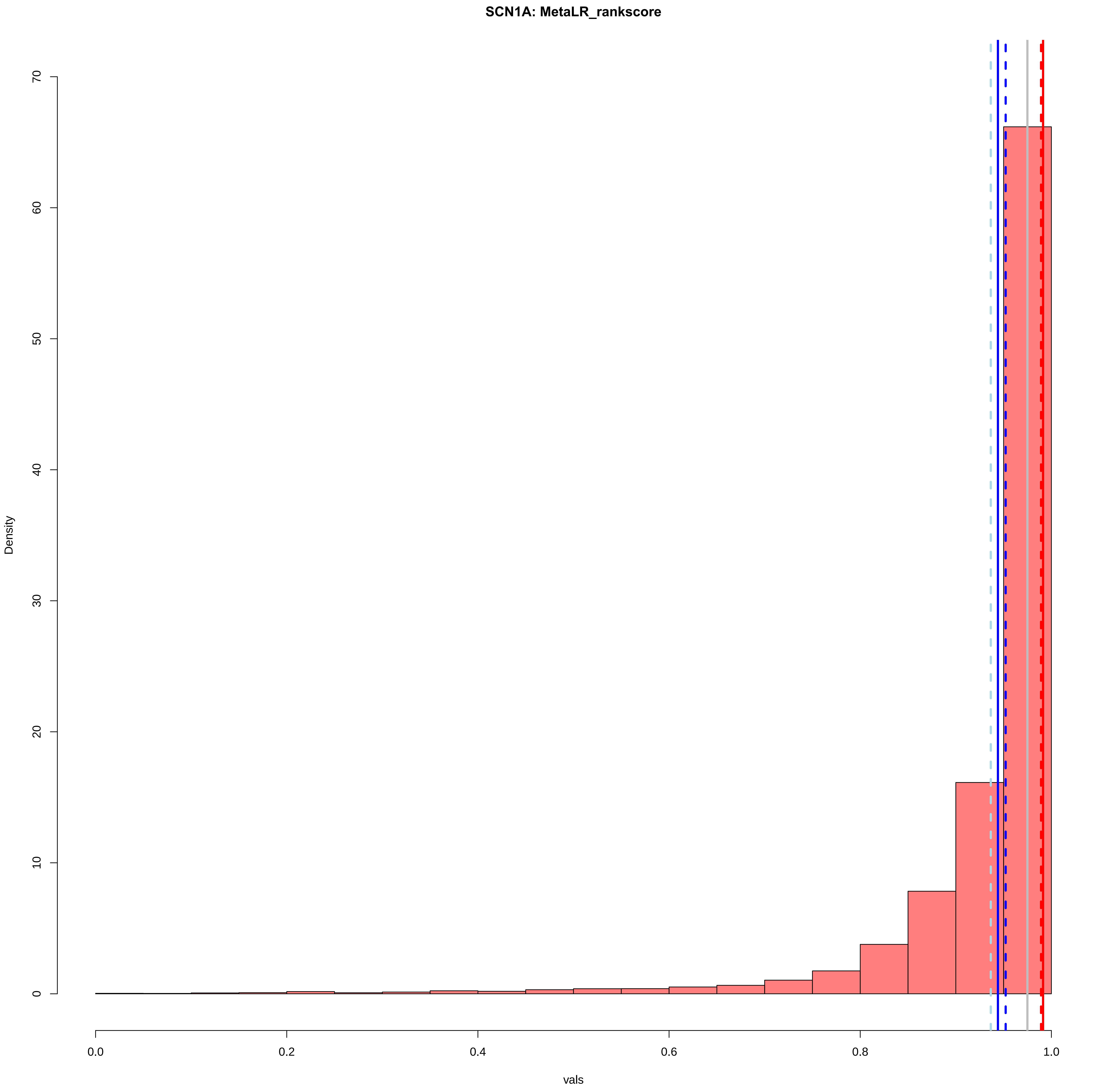

SCN1A: MetaLR_rankscore
Density
vals
0
10
20
30
40
50
60
70
0.0
0.2
0.4
0.6
0.8
1.0

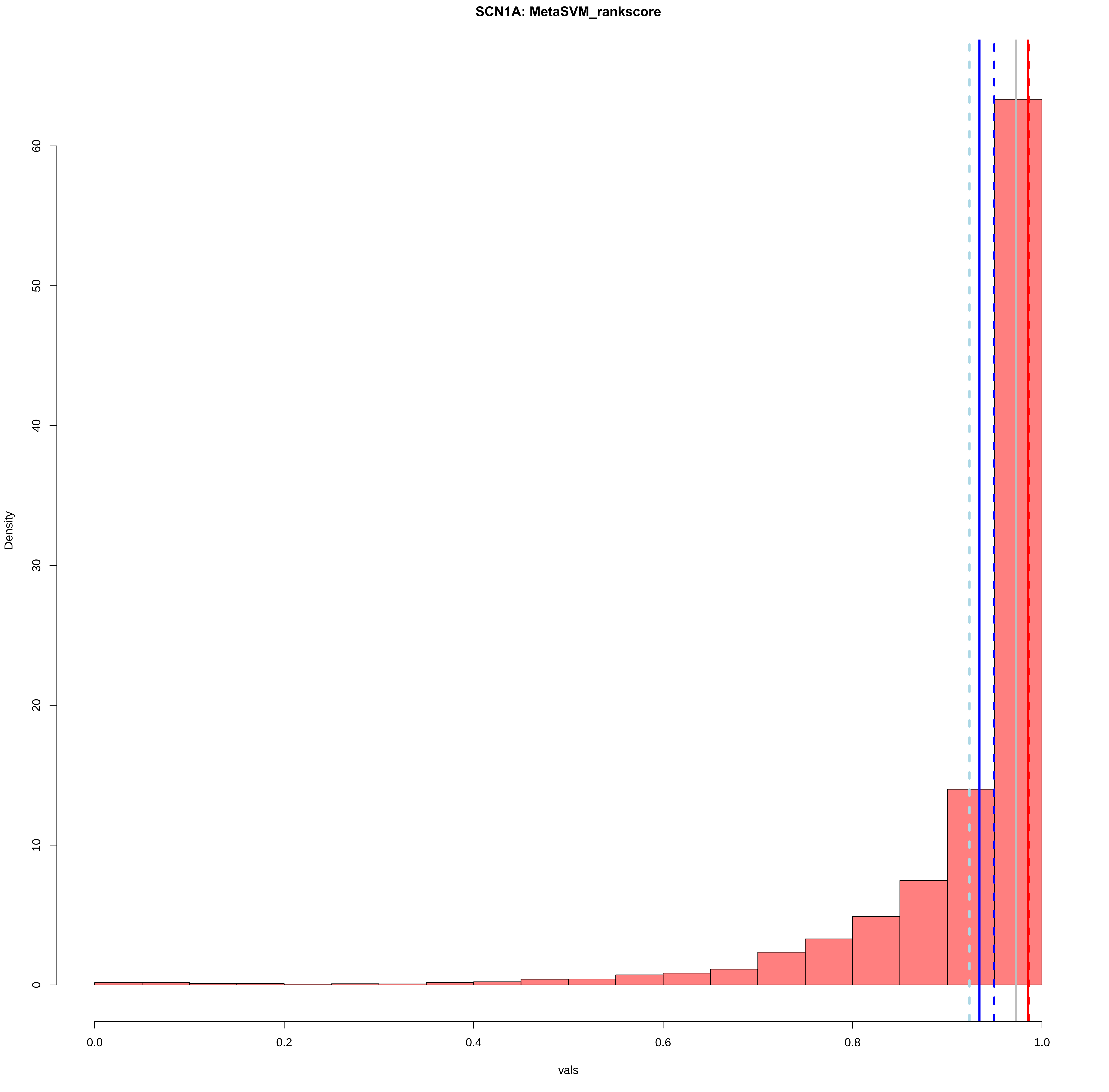

SCN1A: MetaSVM_rankscore
Density
vals
0
10
20
30
40
50
60
0.0
0.2
0.4
0.6
0.8
1.0

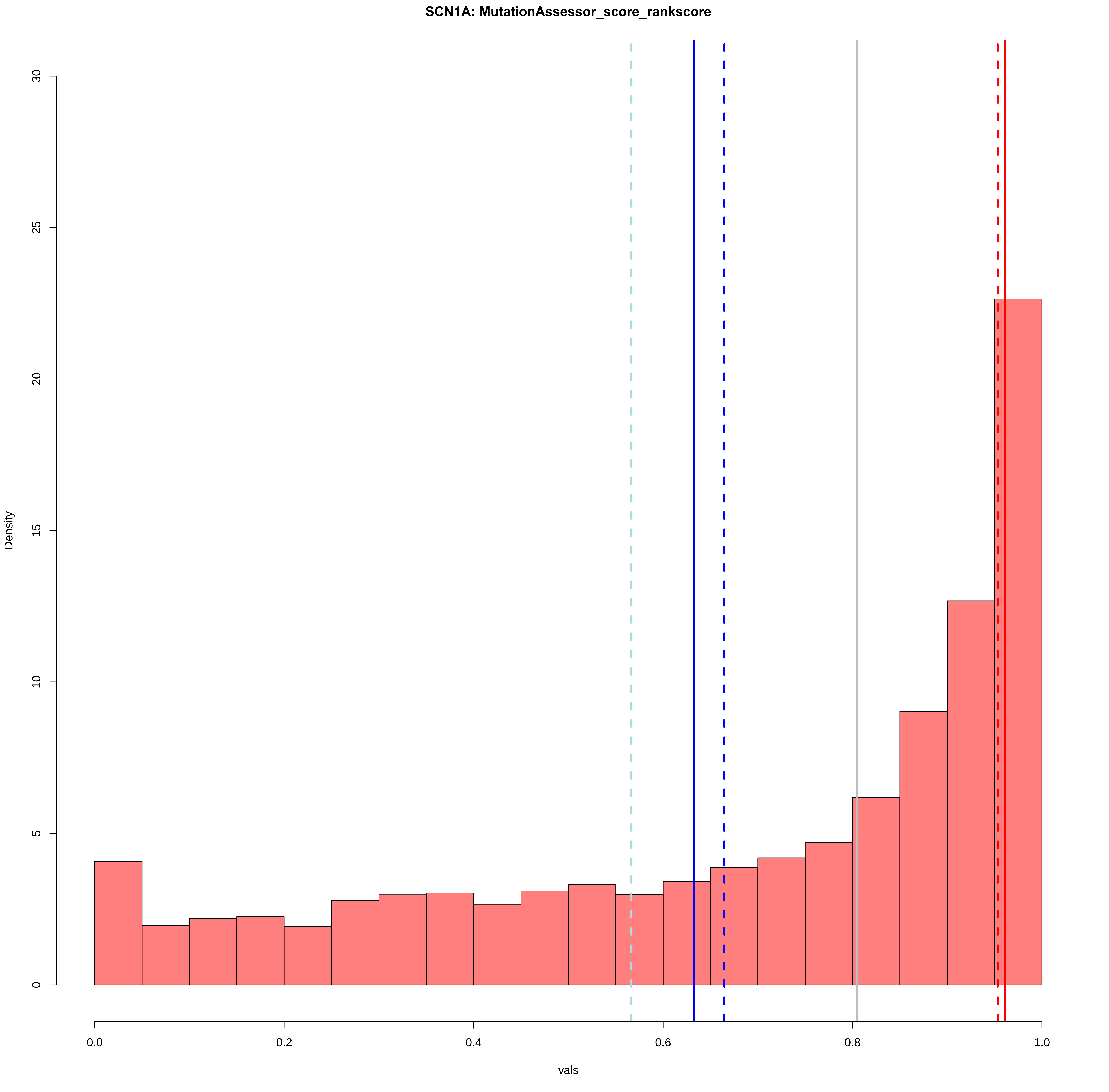
SCN1A: MutationAssessor_score_rankscore
Density
vals
0
5
10
15
20
25
30
0.0
0.2
0.4
0.6
0.8
1.0

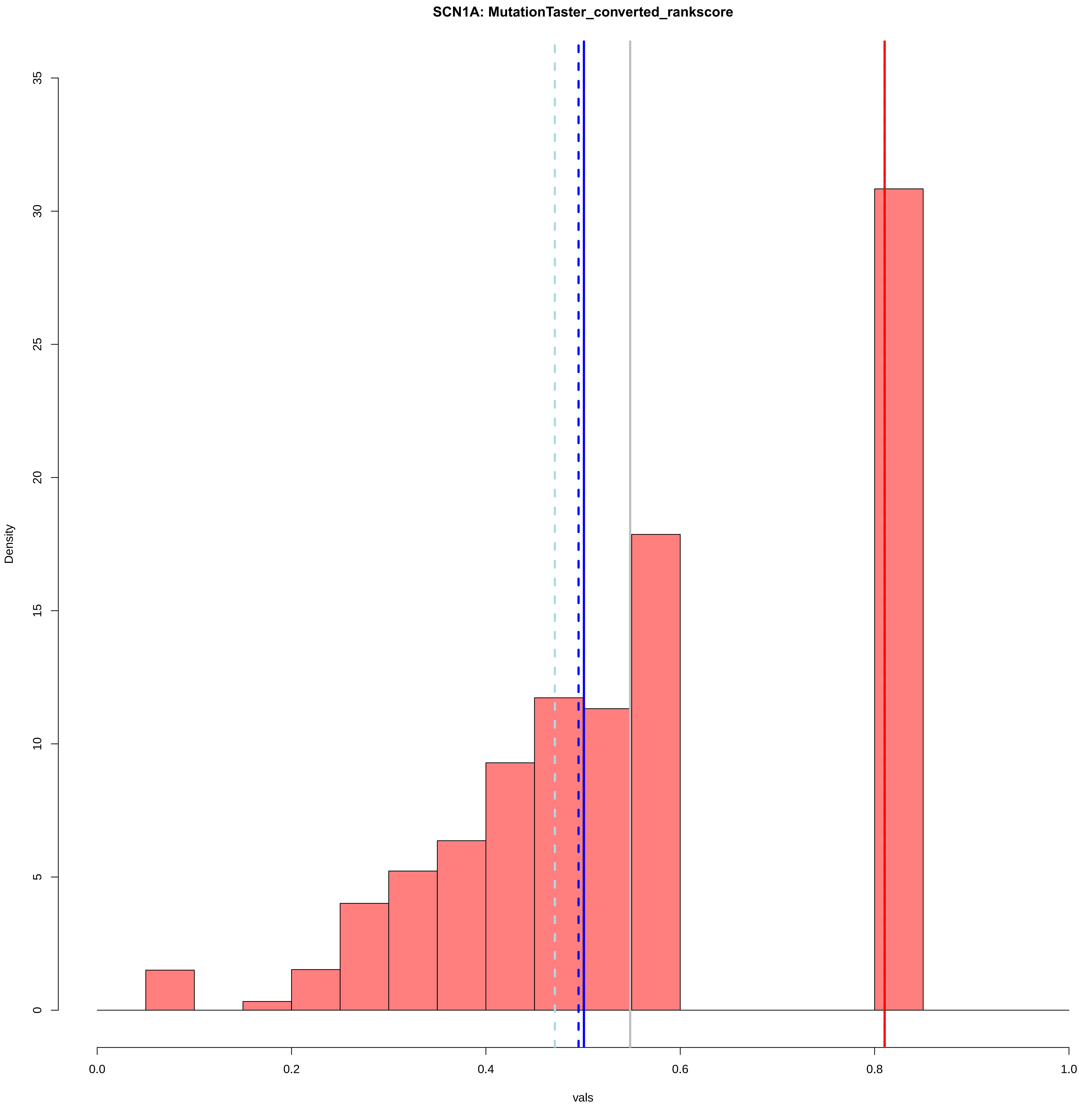
SCN1A: MutationTaster_converted_rankscore
Density
vals
0
5
10
15
20
25
30
35
0.0
0.2
0.4
0.6
0.8
1.0

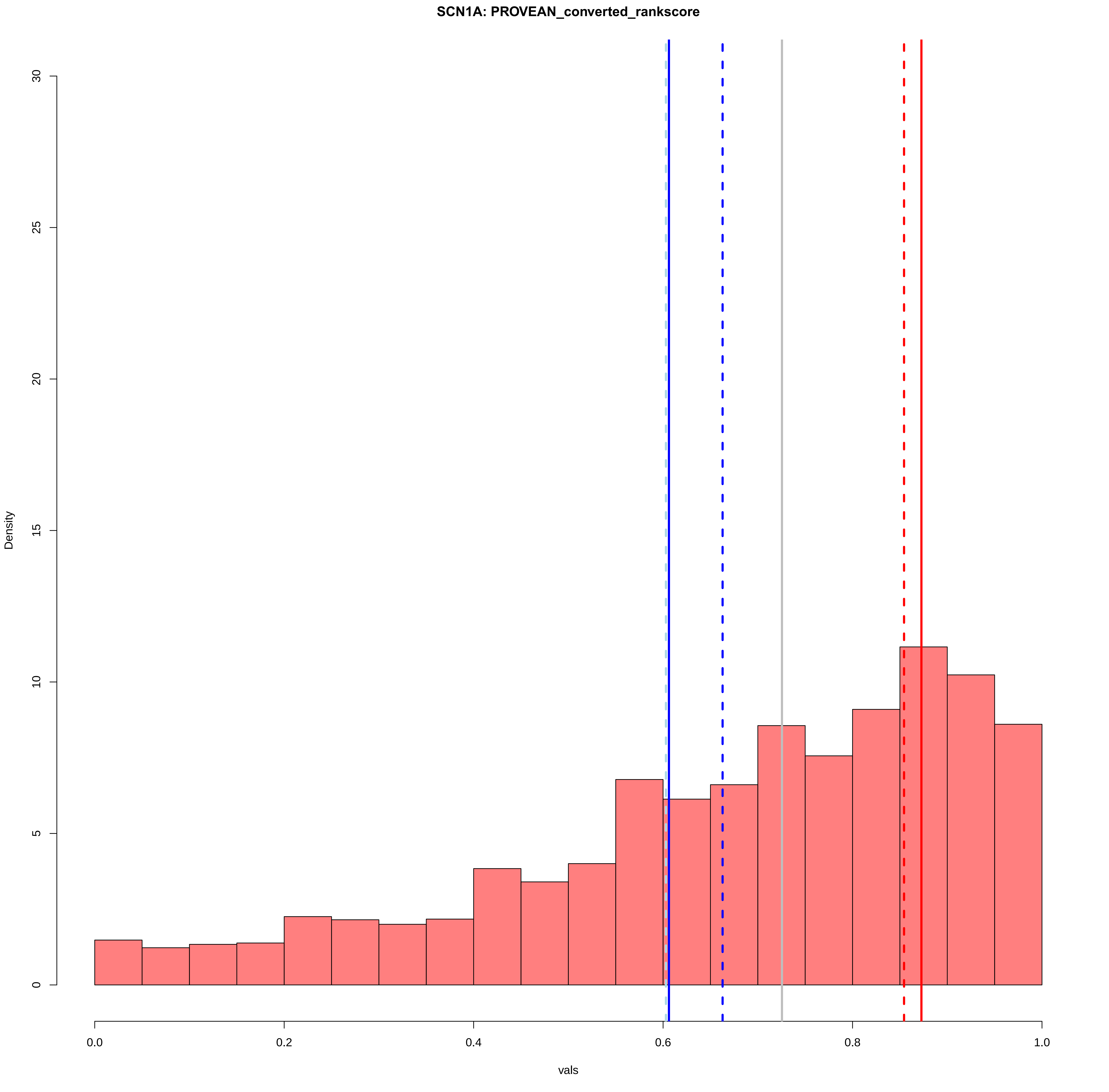

SCN1A: PROVEAN_converted_rankscore
Density
vals
0
5
10
15
20
25
30
0.0
0.2
0.4
0.6
0.8
1.0

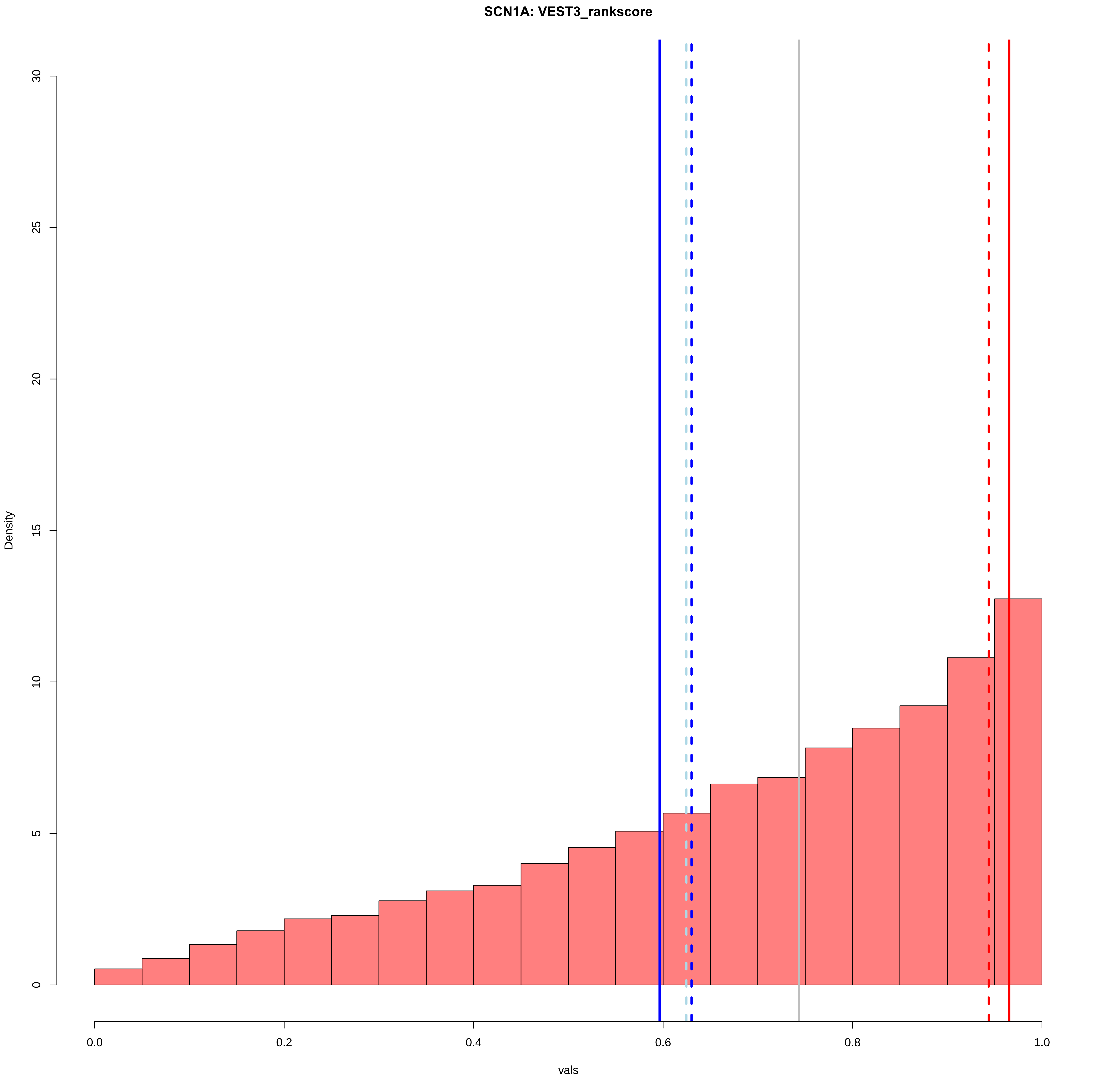
SCN1A: VEST3_rankscore
Density
vals
0
5
10
15
20
25
30
0.0
0.2
0.4
0.6
0.8
1.0

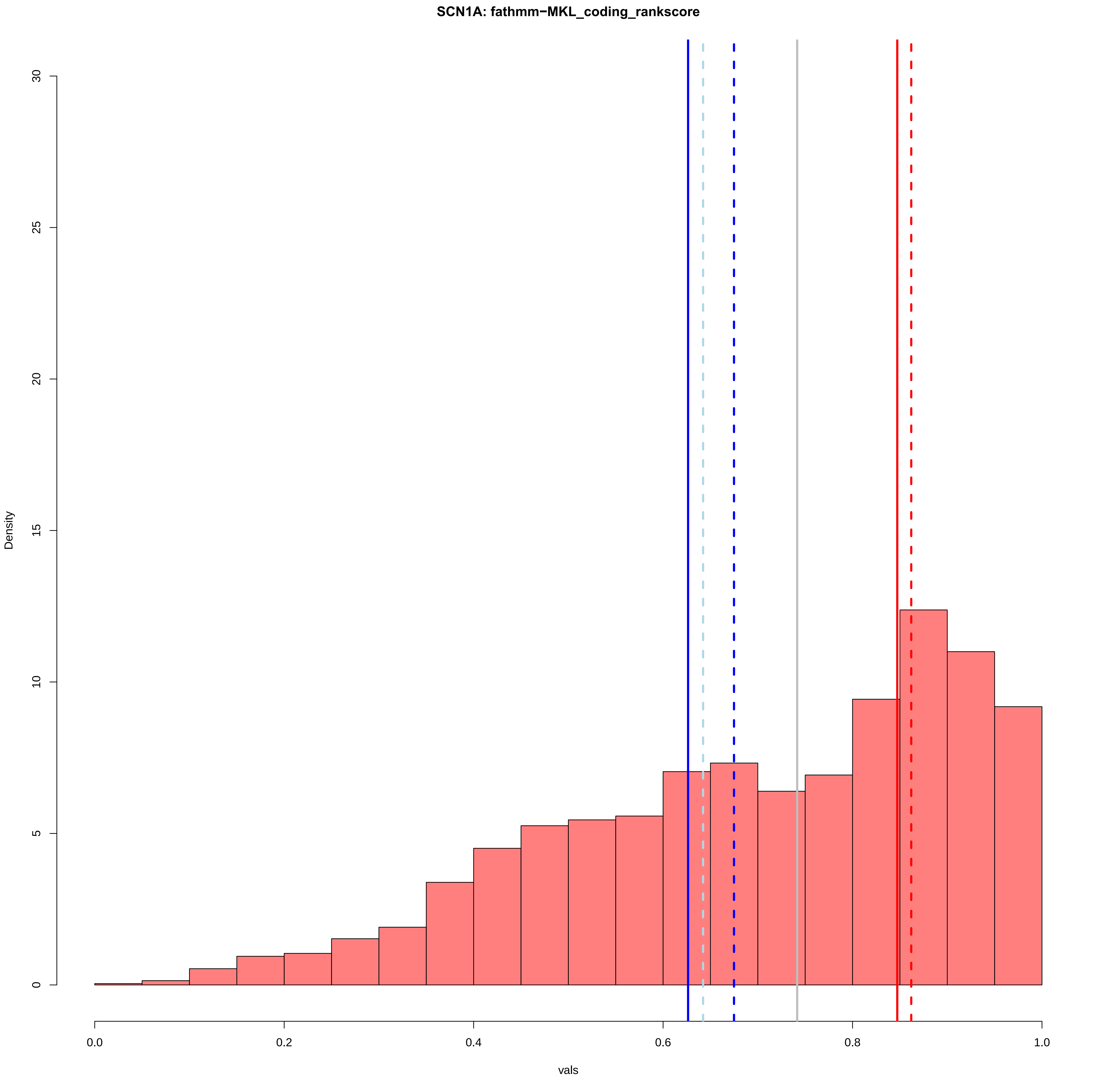
SCN1A: fathmm−MKL_coding_rankscore
Density
vals
0
5
10
15
20
25
30
0.0
0.2
0.4
0.6
0.8
1.0

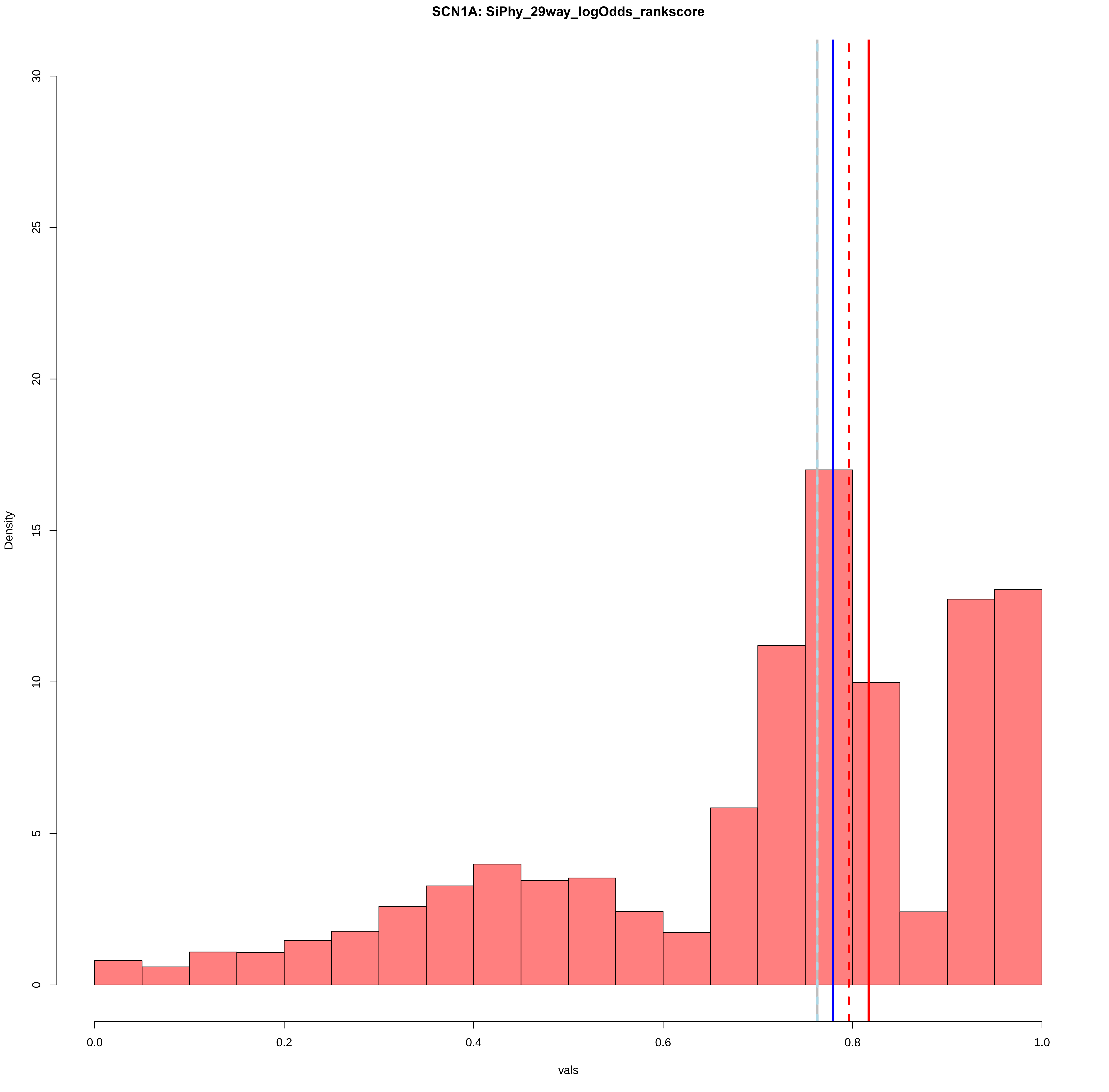
SCN1A: SiPhy_29way_logOdds_rankscore
Density
vals
0
5
10
15
20
25
30
0.0
0.2
0.4
0.6
0.8
1.0

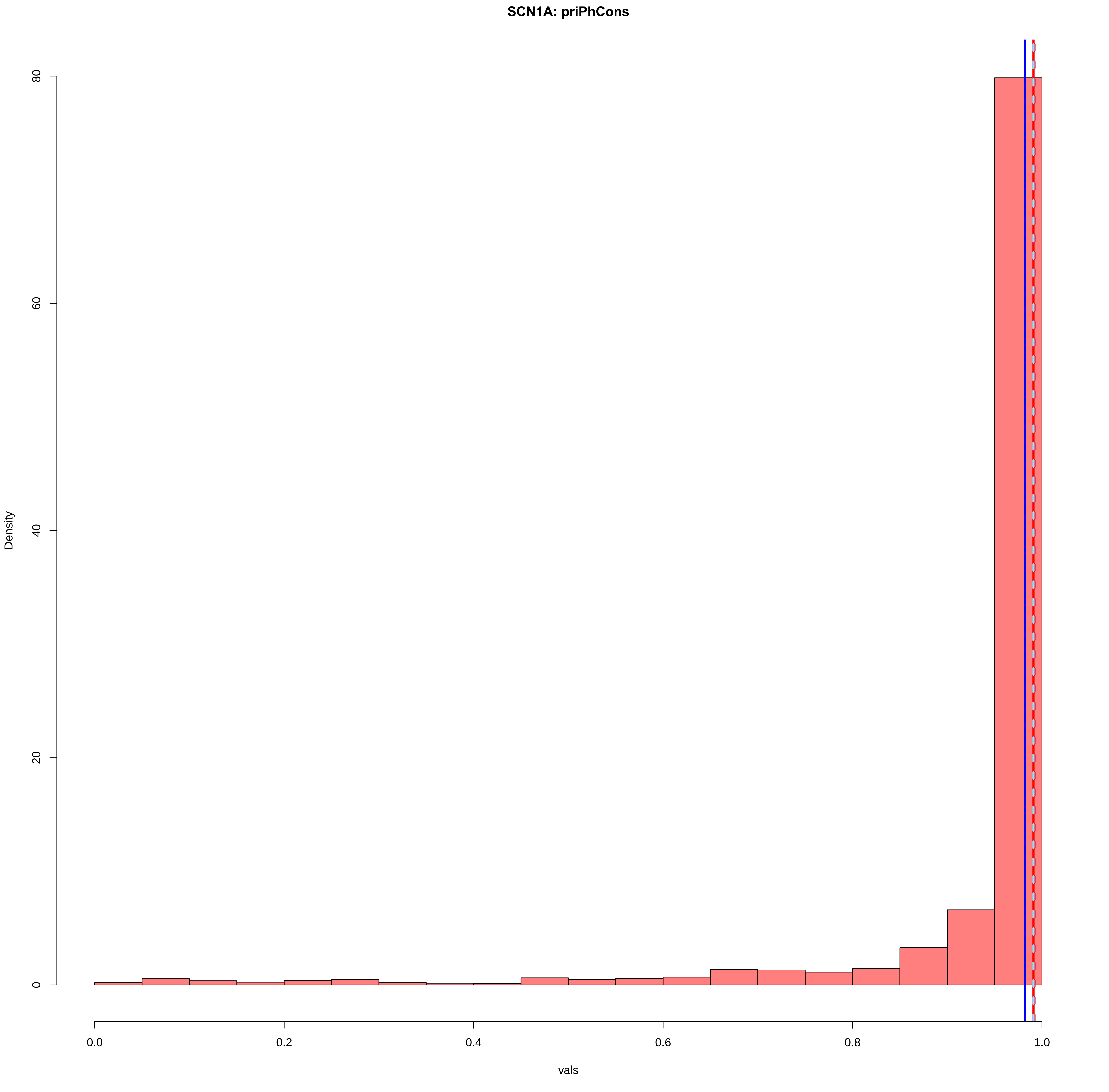

SCN1A: priPhCons
Density
vals
0
20
40
60
80
0.0
0.2
0.4
0.6
0.8
1.0

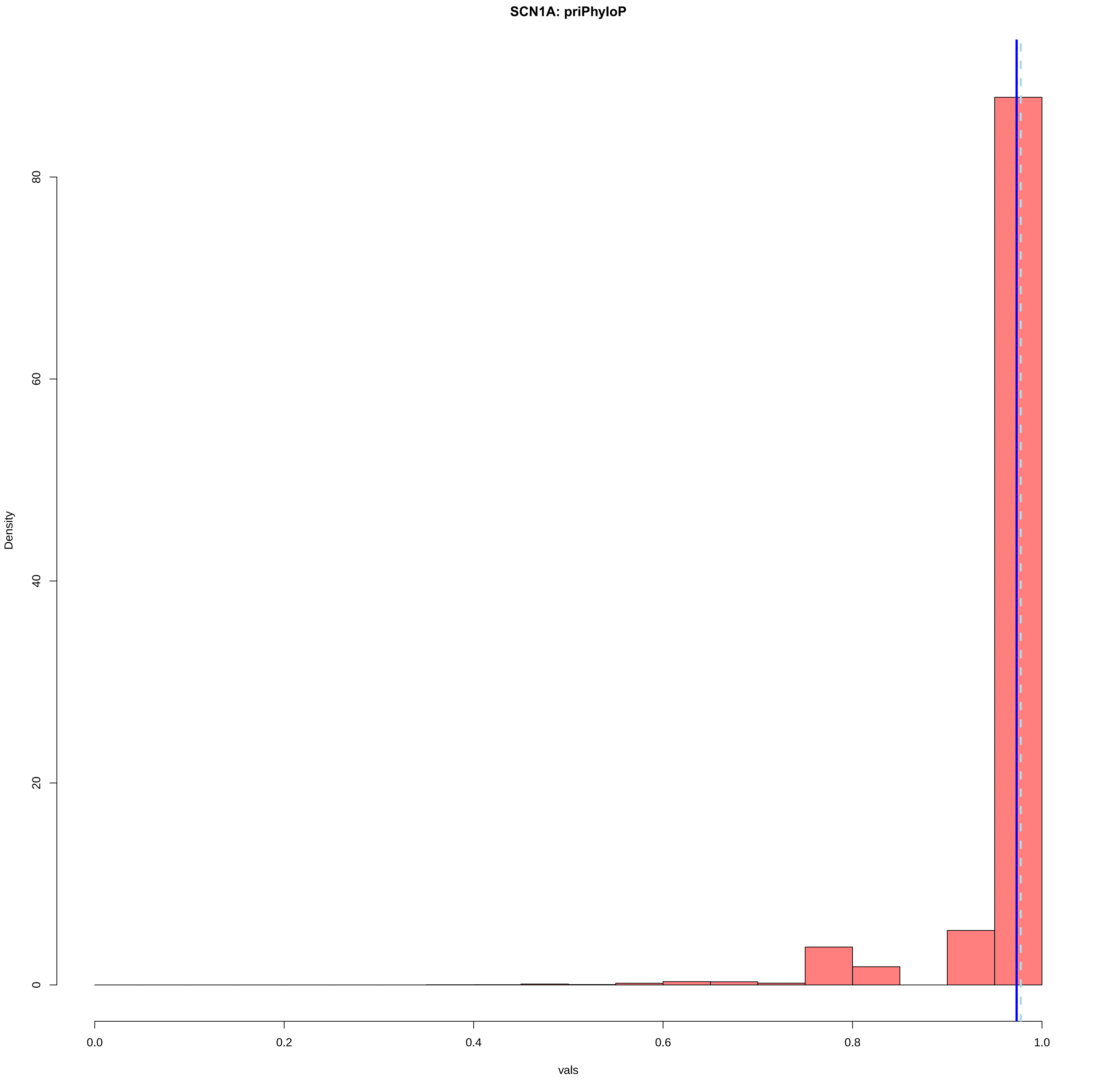
SCN1A: priPhyloP
Density
0
20
40
60
80
0.0
0.2
0.4
0.6
0.8
1.0
vals

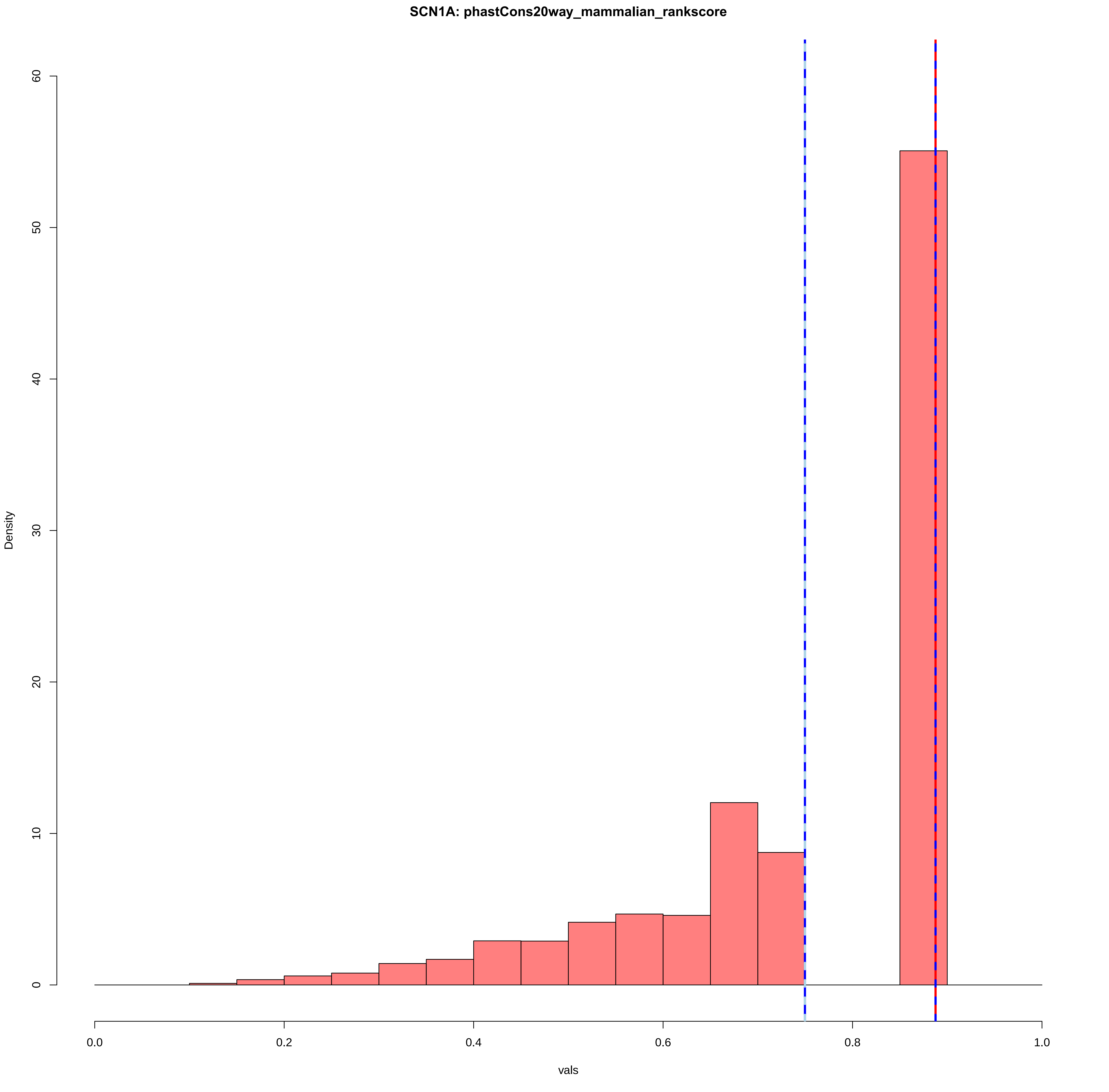
SCN1A: phastCons20way_mammalian_rankscore
Density
vals
0
10
20
30
40
50
60
0.0
0.2
0.4
0.6
0.8
1.0

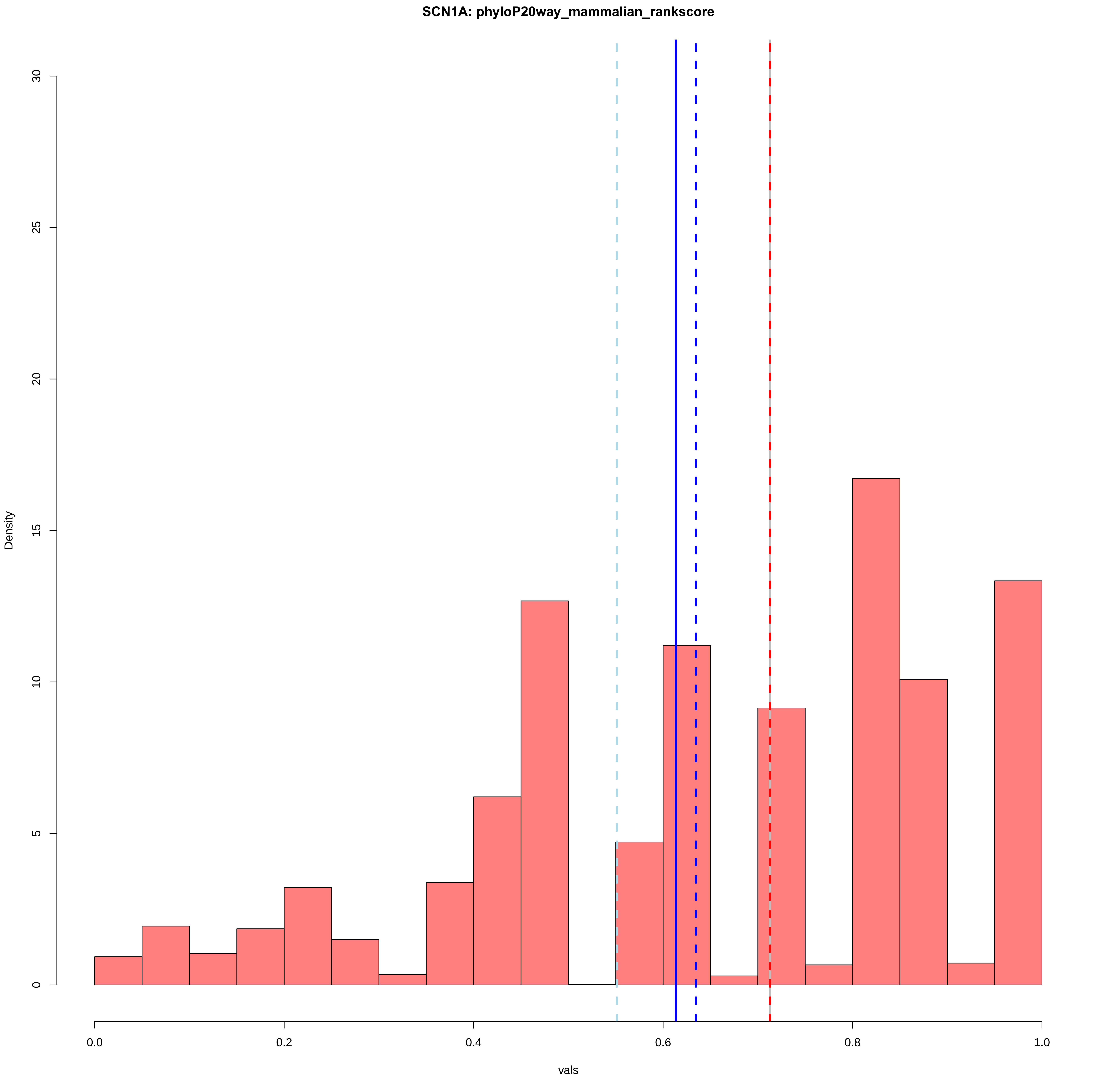

SCN1A: phyloP20way_mammalian_rankscore
Density
0
5
10
15
20
25
30
0.0
0.2
0.4
0.6
0.8
1.0
vals

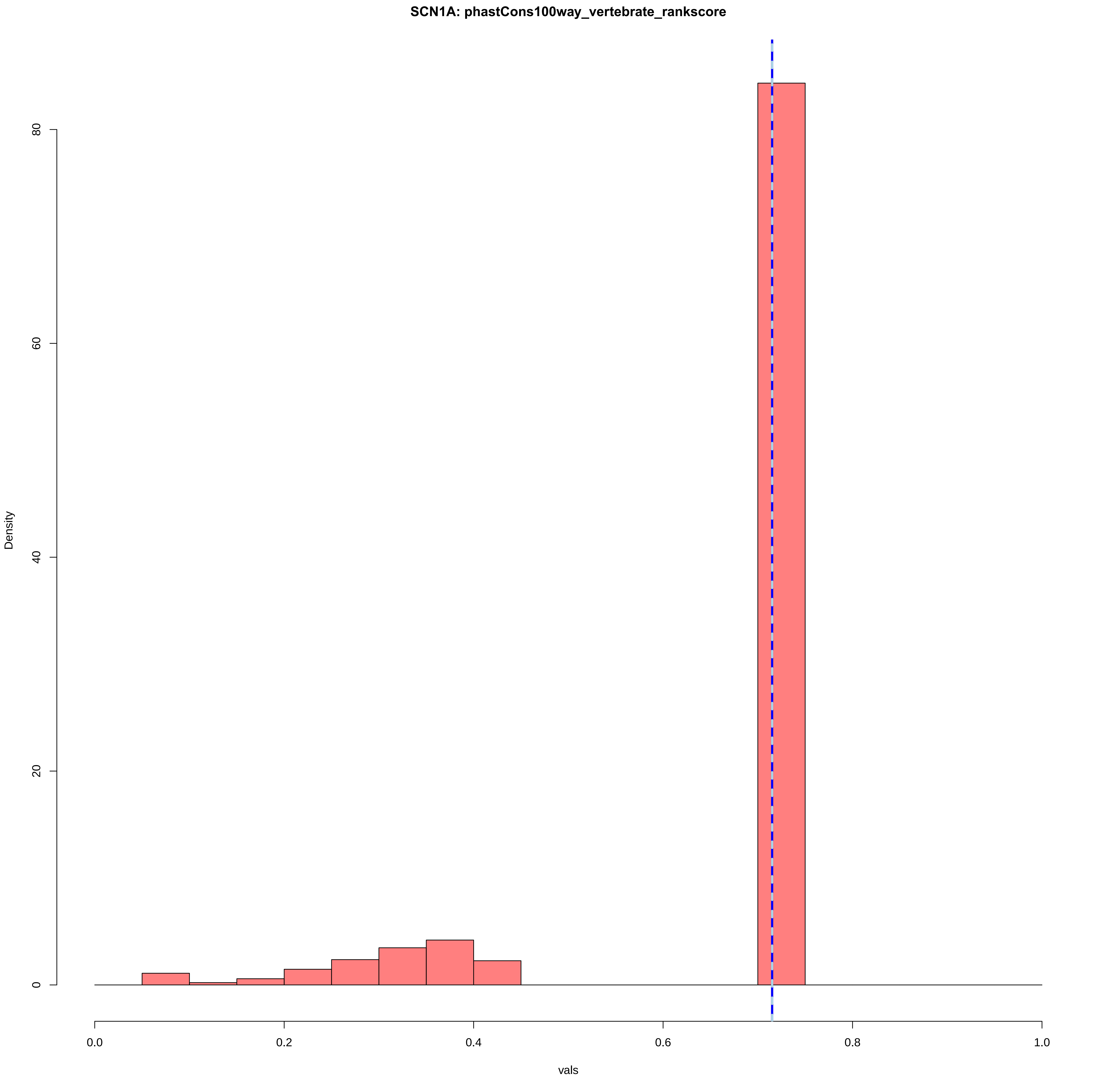

SCN1A: phastCons100way_vertebrate_rankscore
Density
0
20
40
60
80
0.0
0.2
0.4
0.6
0.8
1.0
vals

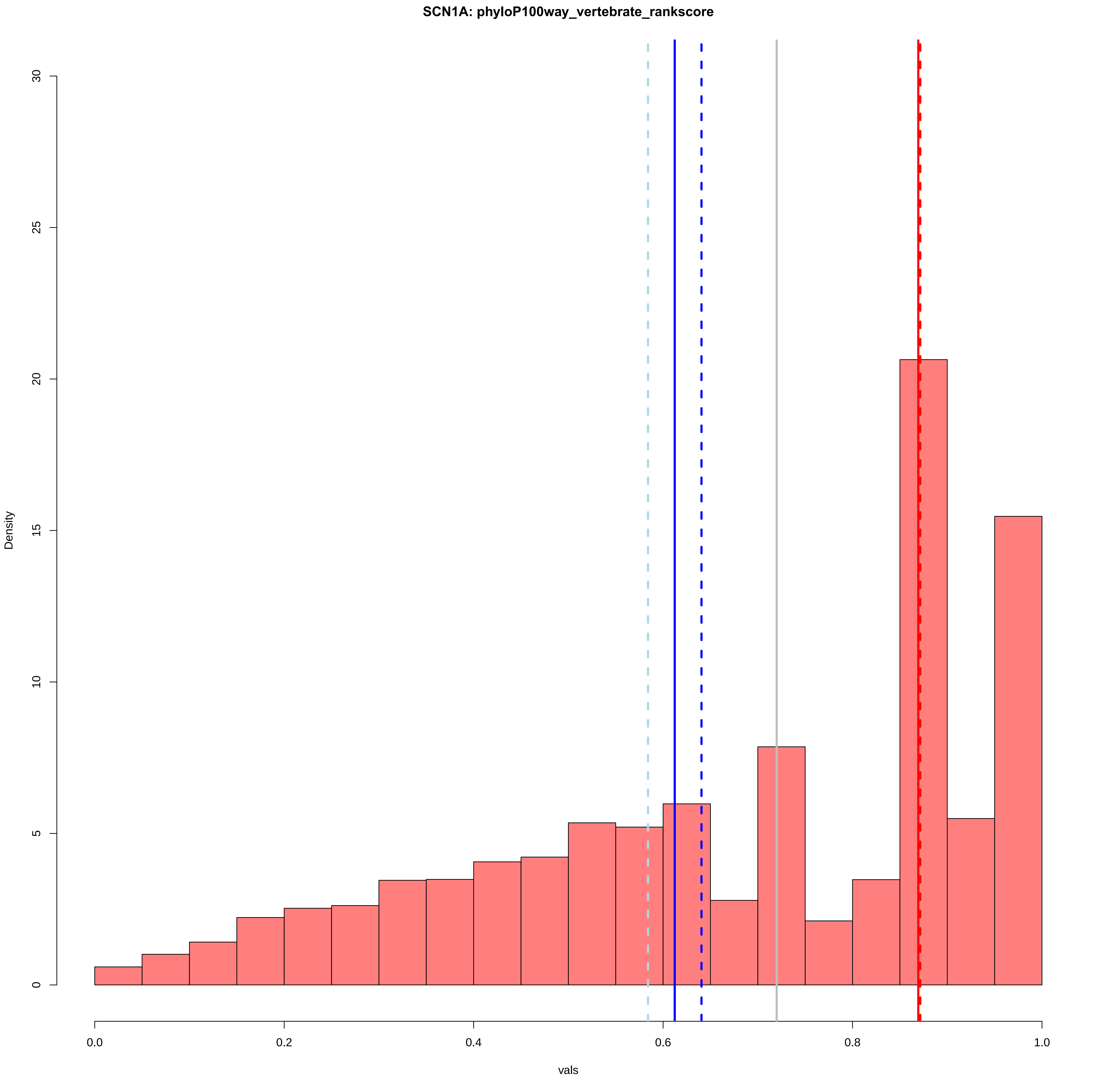

SCN1A: phyloP100way_vertebrate_rankscore
Density
vals
0
5
10
15
20
25
30
0.0
0.2
0.4
0.6
0.8
1.0

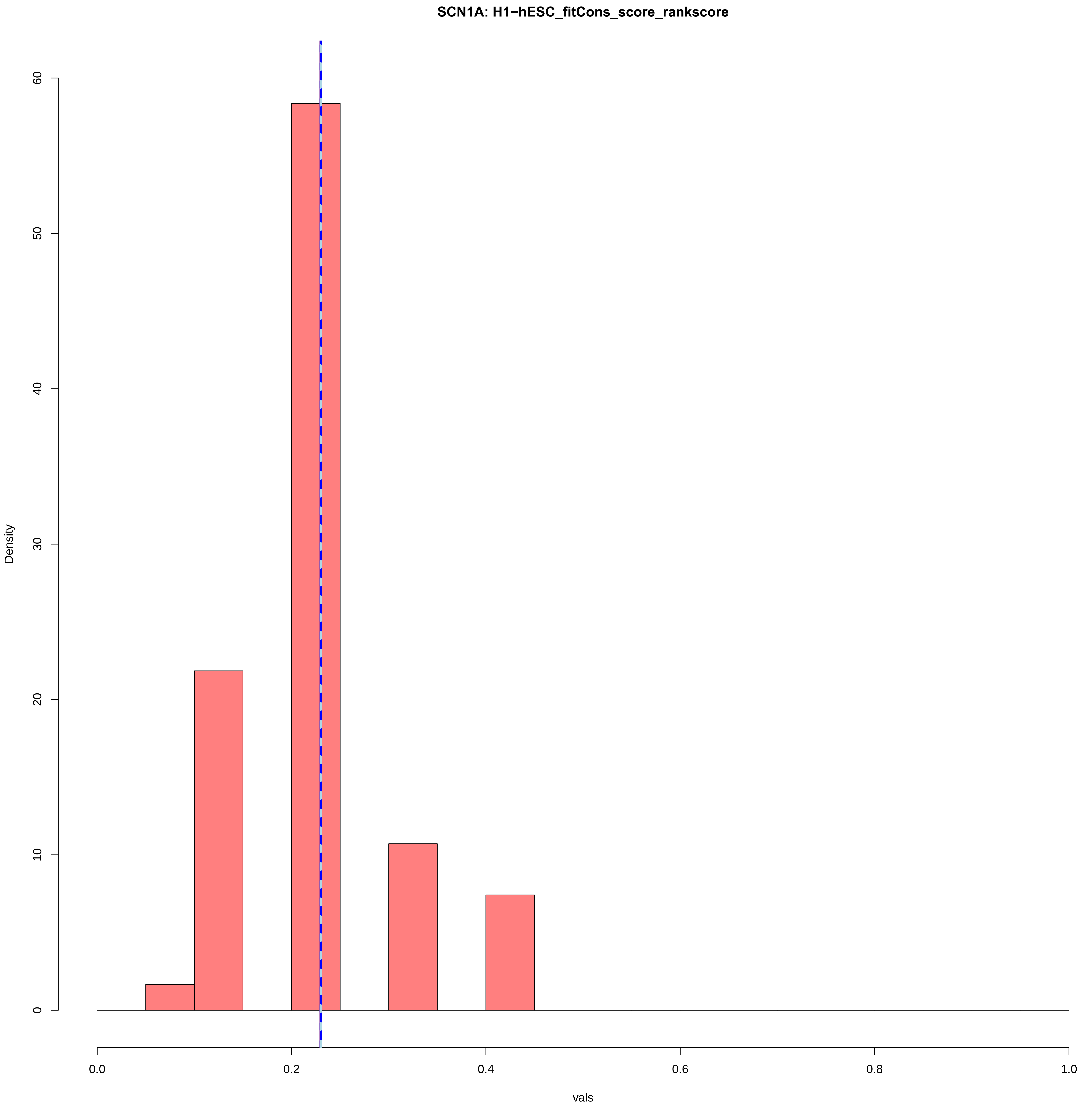

SCN1A: H1−hESC_fitCons_score_rankscore
Density
vals
0
10
20
30
40
50
60
0.0
0.2
0.4
0.6
0.8
1.0

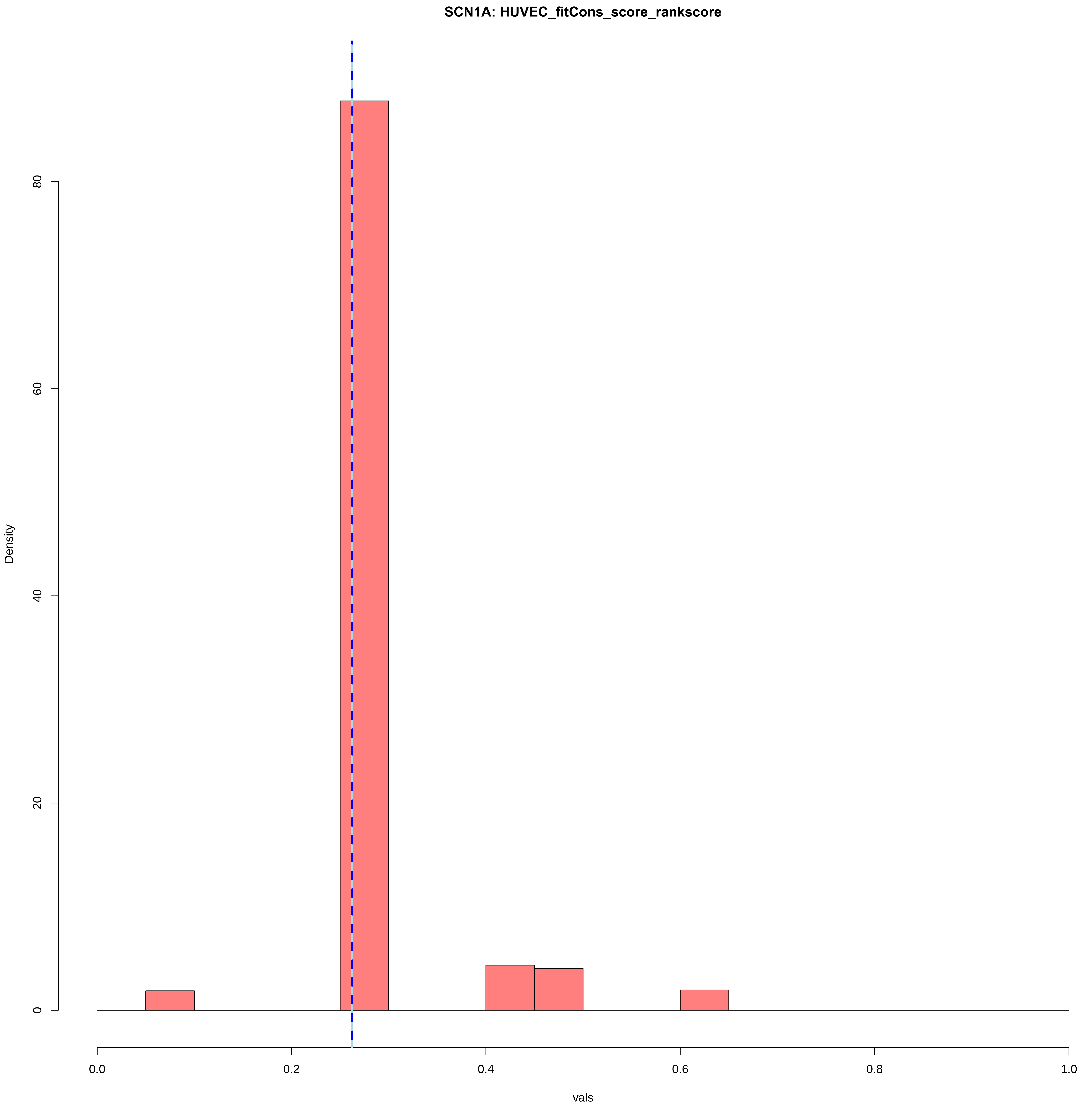

SCN1A: HUVEC_fitCons_score_rankscore
Density
0
20
40
60
80
0.0
0.2
0.4
0.6
0.8
1.0
vals

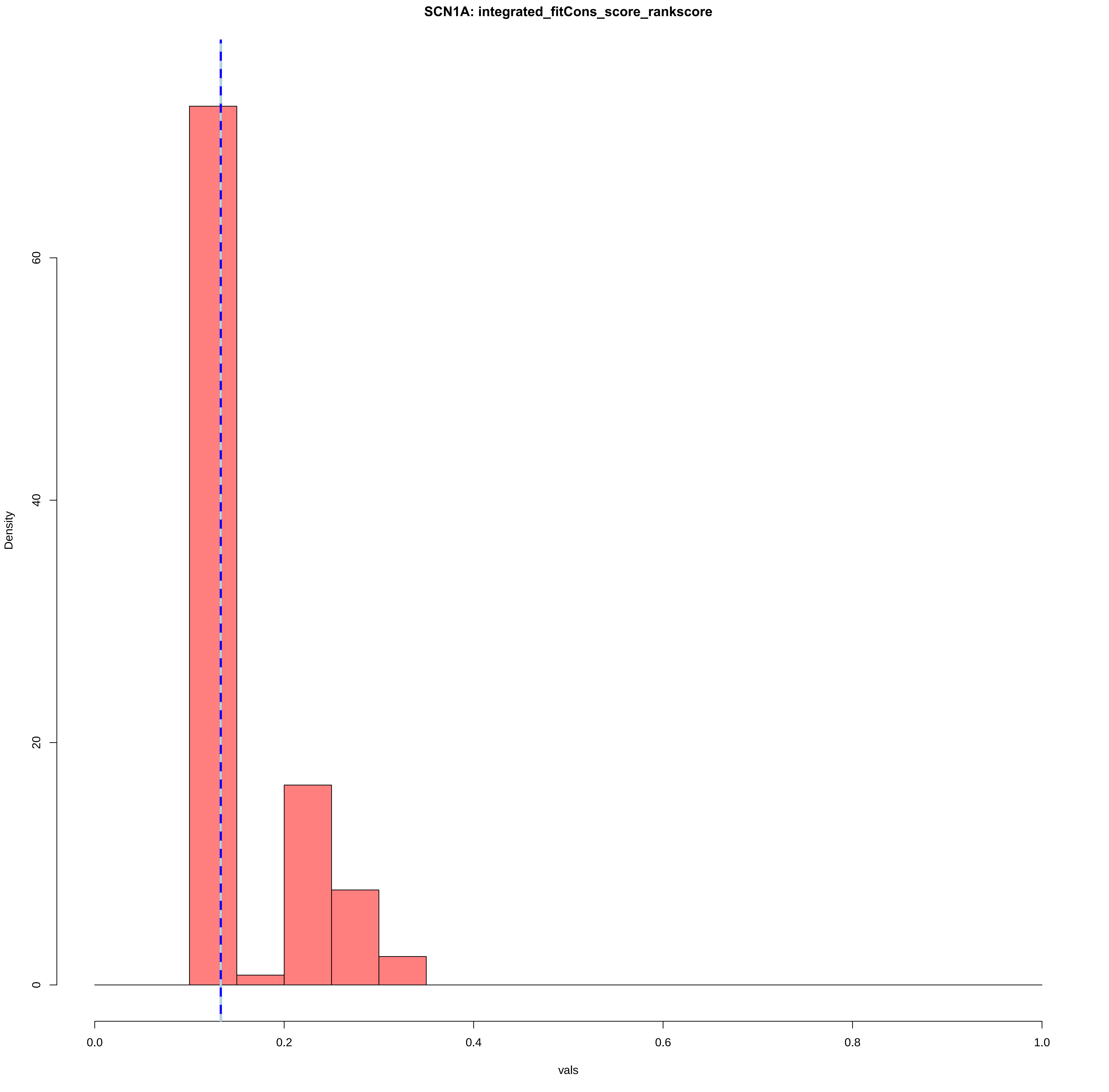

SCN1A: integrated_fitCons_score_rankscore
Density
0
20
40
60
vals
0.0
0.2
0.4
0.6
0.8
1.0

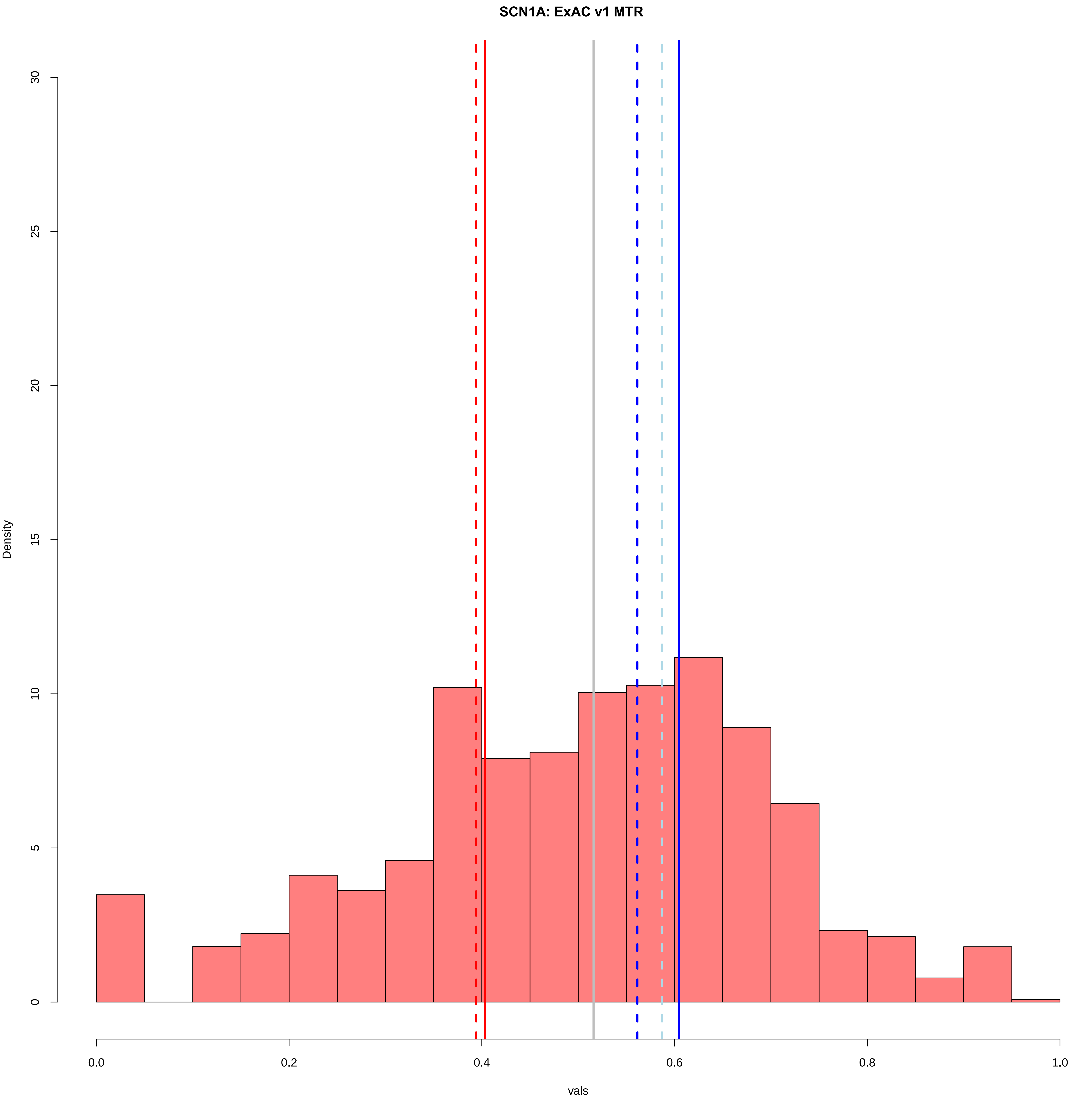
SCN1A: ExAC v1 MTR
Density
vals
0
5
10
15
20
25
30
0.0
0.2
0.4
0.6
0.8
1.0

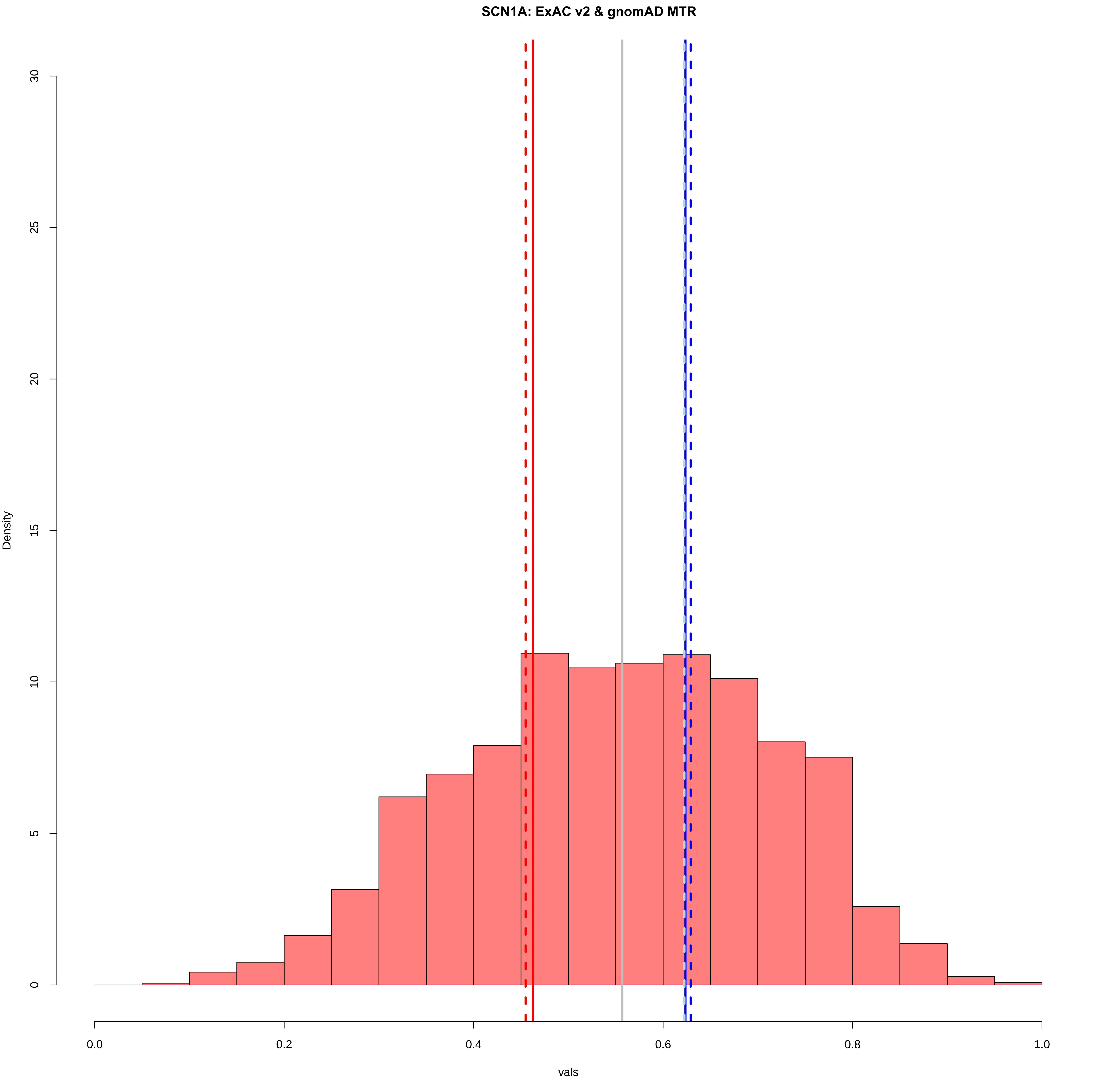
SCN1A: ExAC v2 & gnomAD MTR
Density
vals
0
5
10
15
20
25
30
0.0
0.2
0.4
0.6
0.8
1.0

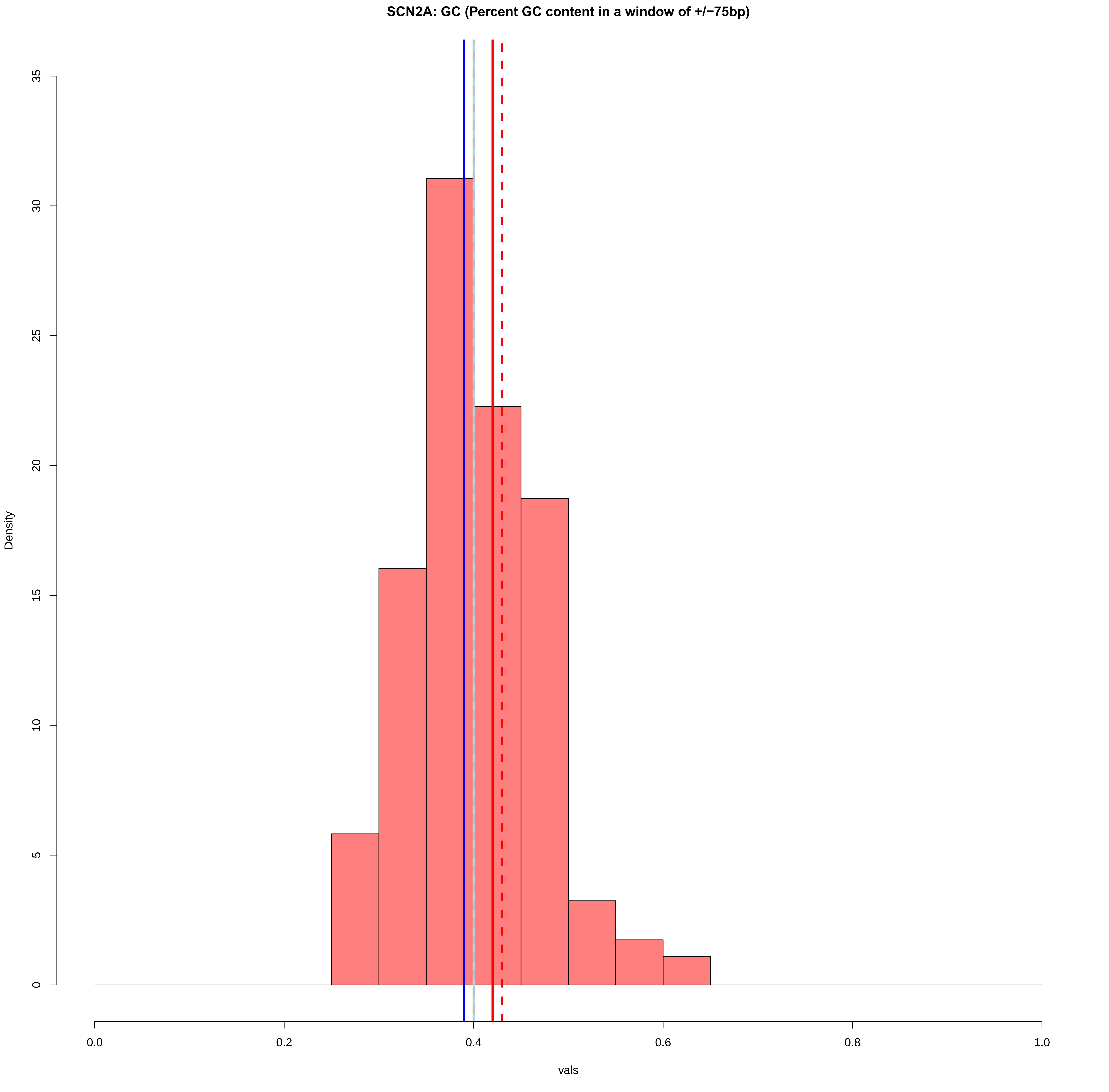

SCN2A: GC (Percent GC content in a window of +/−75bp)
Density
0
5
10
15
20
25
30
35
0.0
0.2
0.4
0.6
0.8
1.0
vals

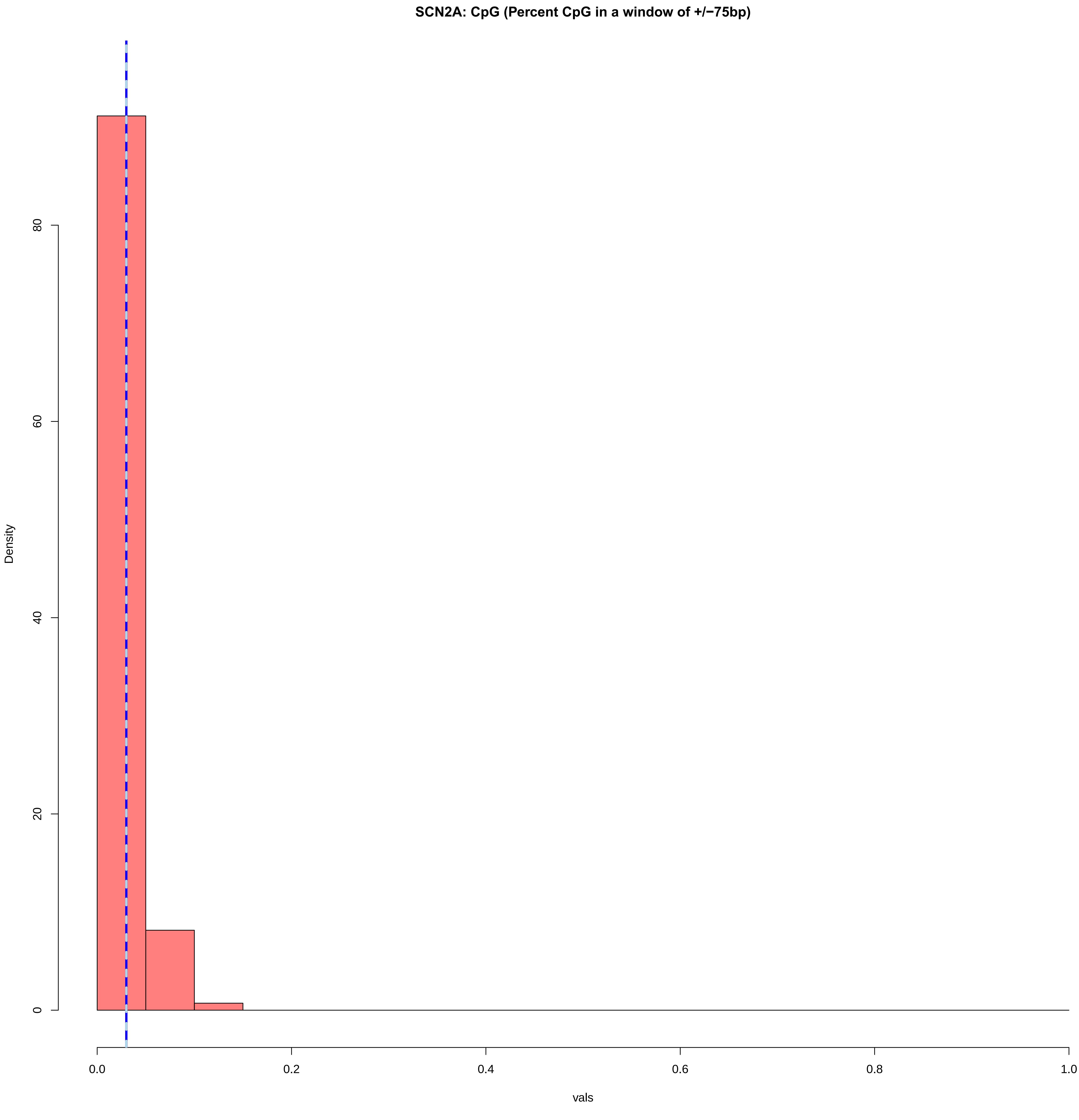

SCN2A: CpG (Percent CpG in a window of +/−75bp)
Density
0
20
40
60
80
0.0
0.2
0.4
0.6
0.8
1.0
vals

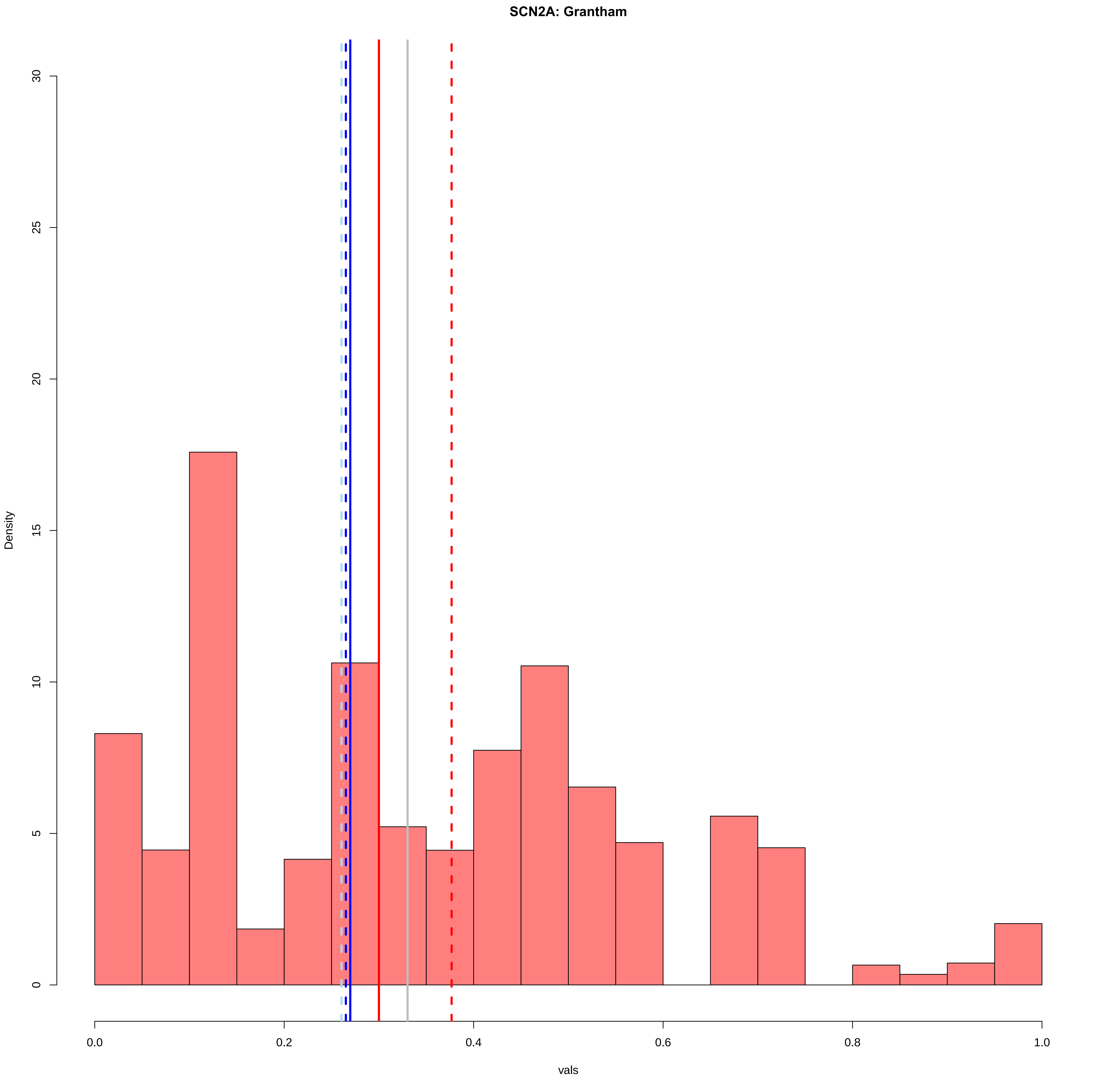

SCN2A: Grantham
Density
vals
0
5
10
15
20
25
30
0.0
0.2
0.4
0.6
0.8
1.0

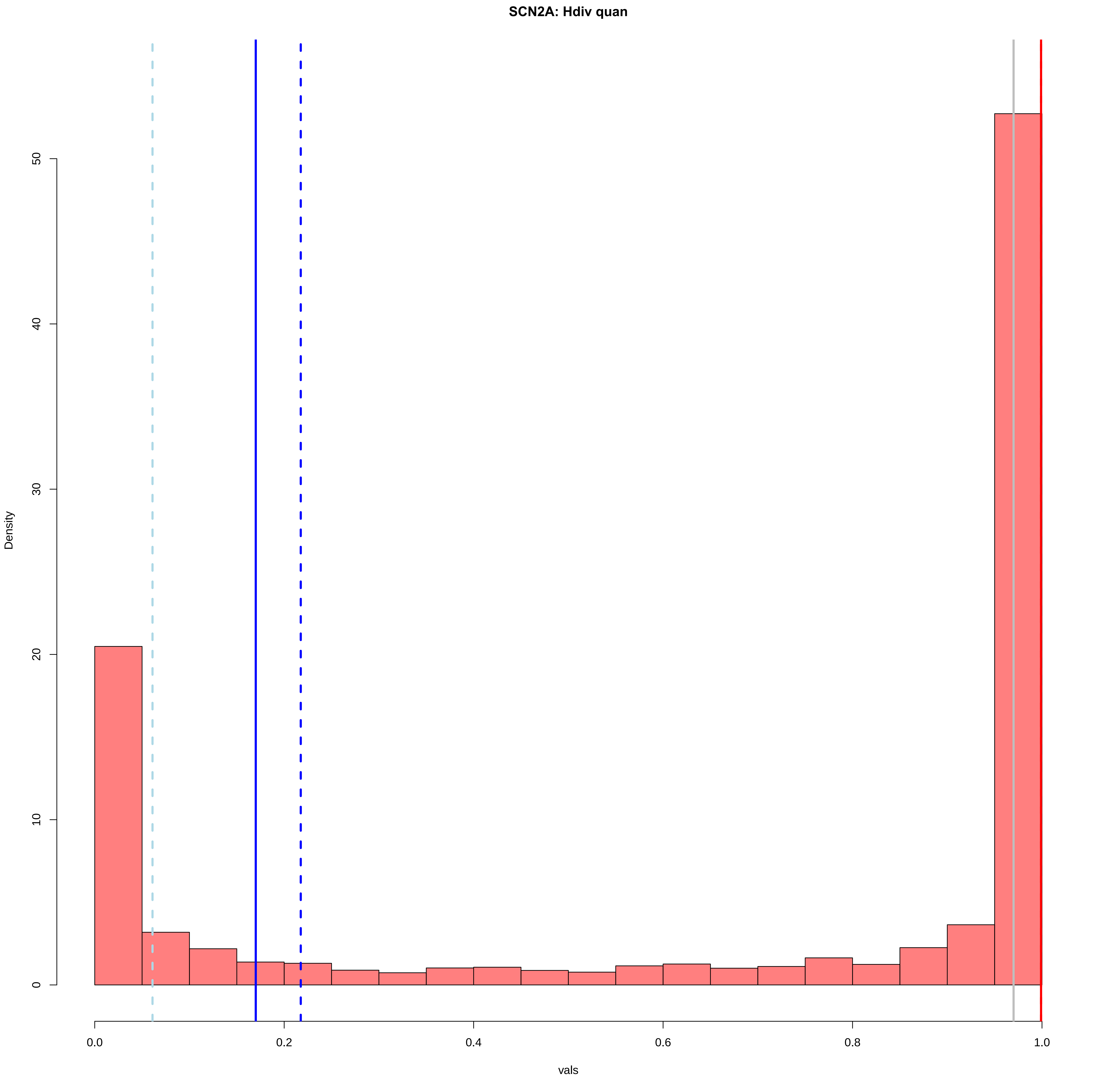
SCN2A: Hdiv quan
Density
0
10
20
30
40
50
0.0
0.2
0.4
0.6
0.8
1.0
vals

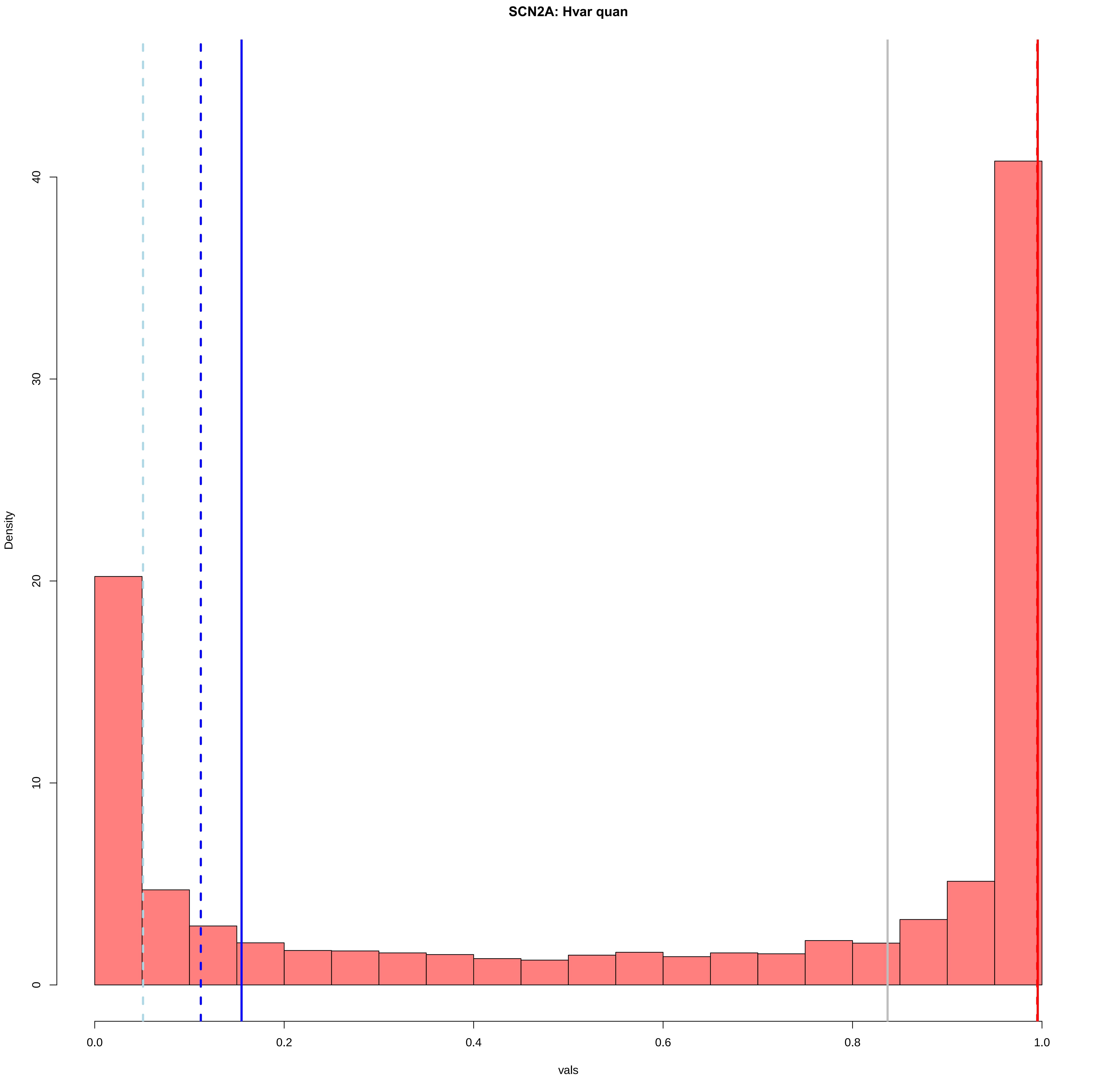

SCN2A: Hvar quan
Density
vals
0
10
20
30
40
0.0
0.2
0.4
0.6
0.8
1.0

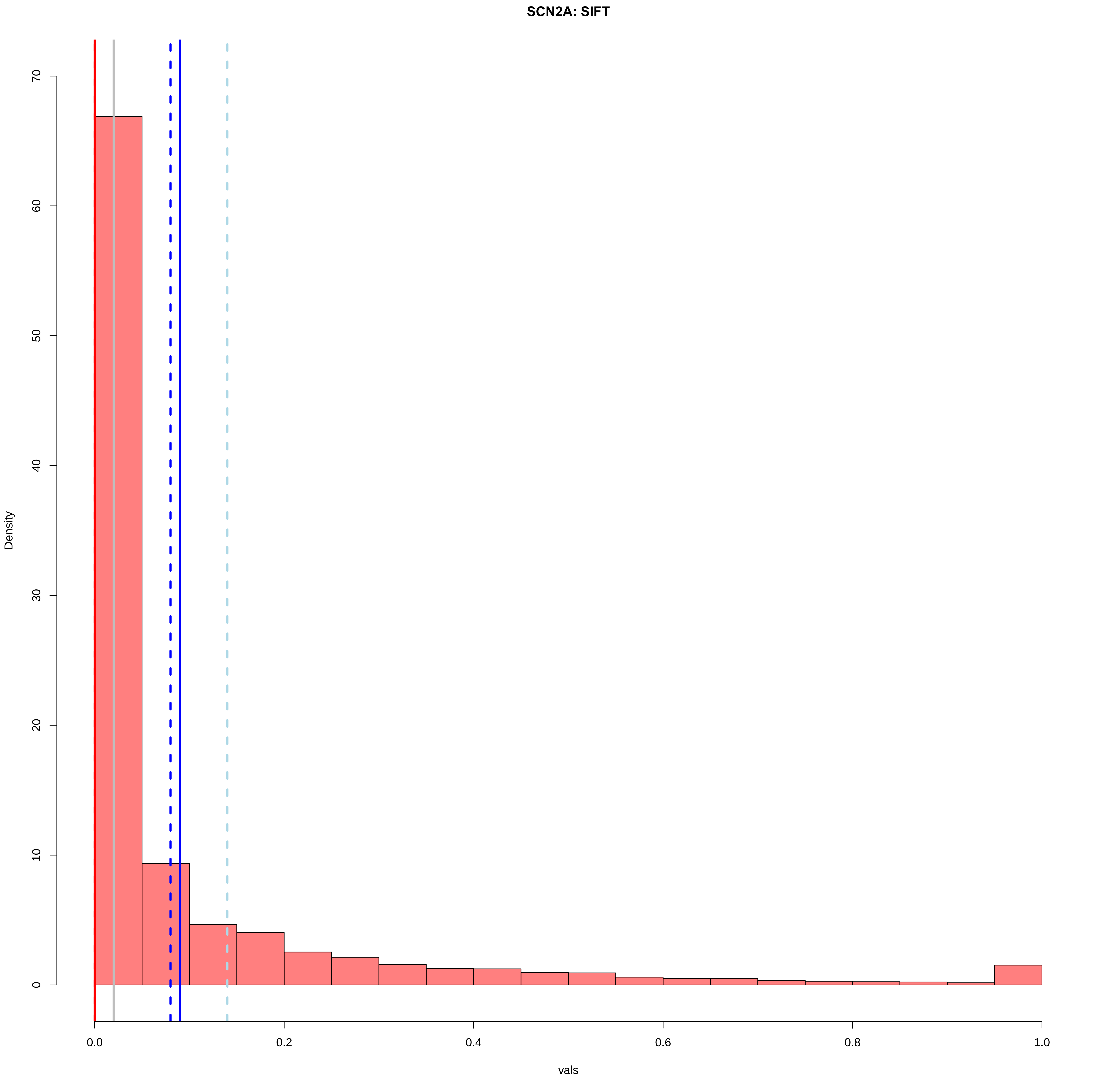
SCN2A: SIFT
Density
vals
0
10
20
30
40
50
60
70
0.0
0.2
0.4
0.6
0.8
1.0

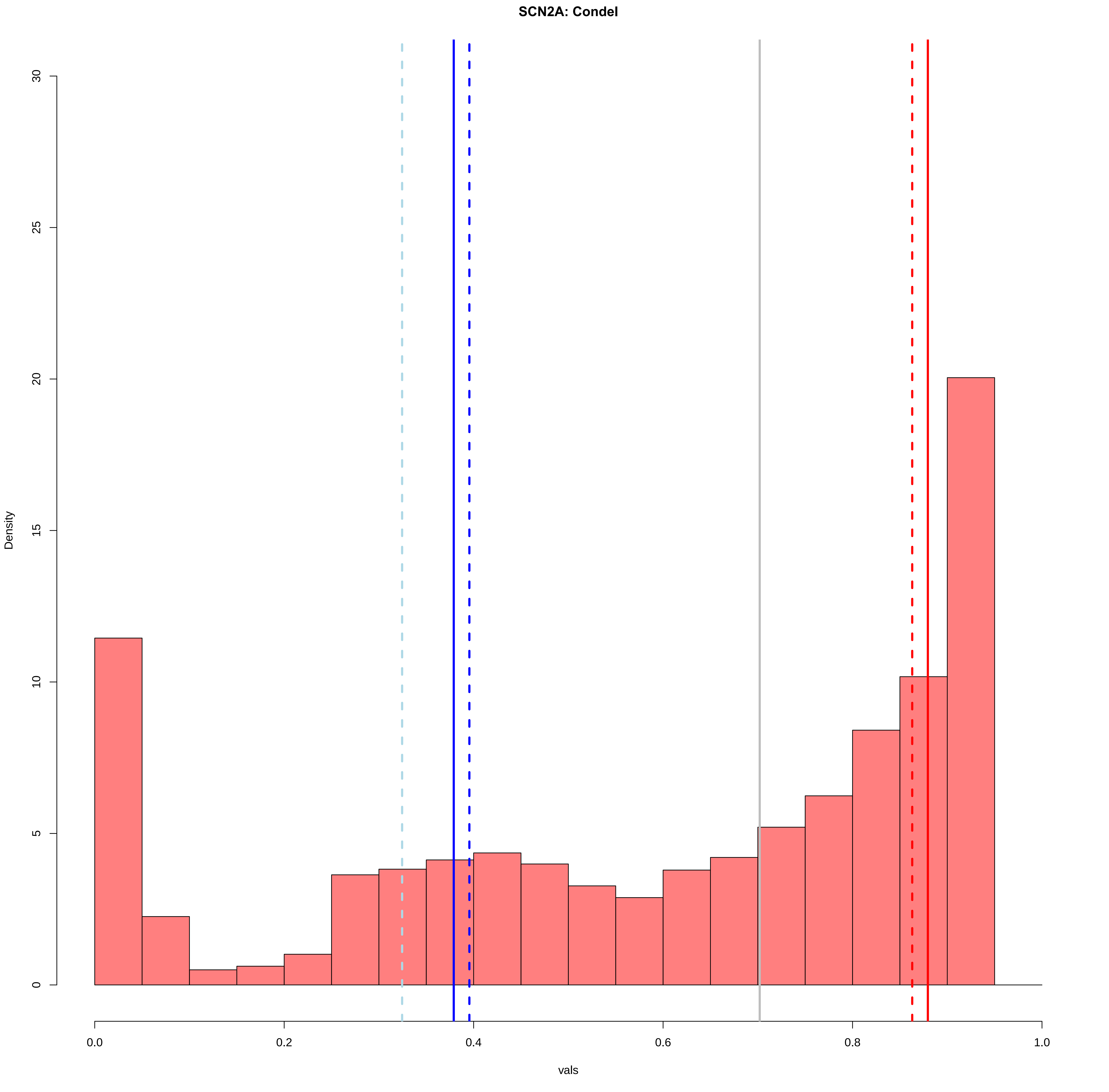
SCN2A: Condel
Density
vals
0
5
10
15
20
25
30
0.0
0.2
0.4
0.6
0.8
1.0

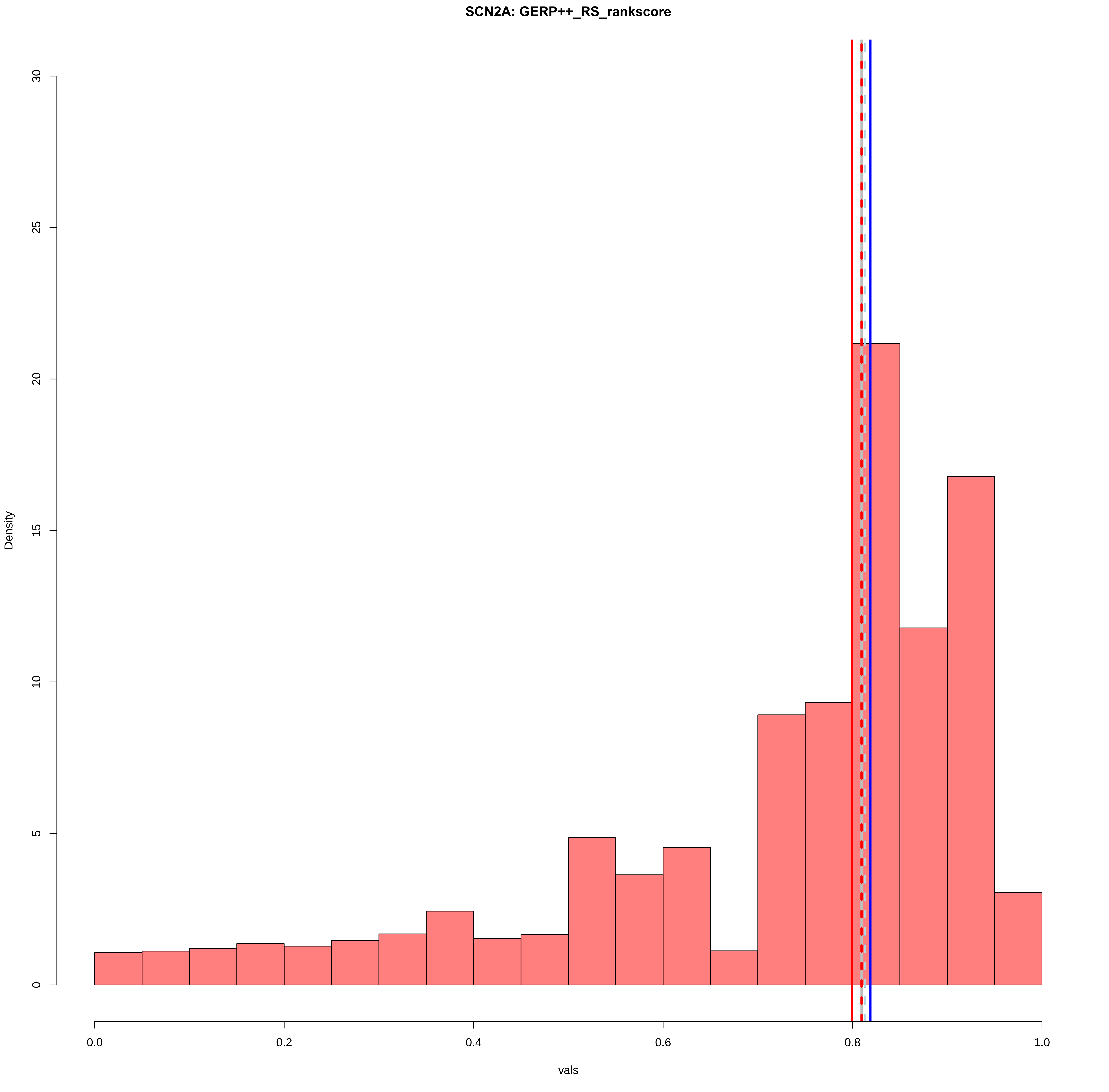

SCN2A: GERP++_RS_rankscore
Density
vals
0
5
10
15
20
25
30
0.0
0.2
0.4
0.6
0.8
1.0

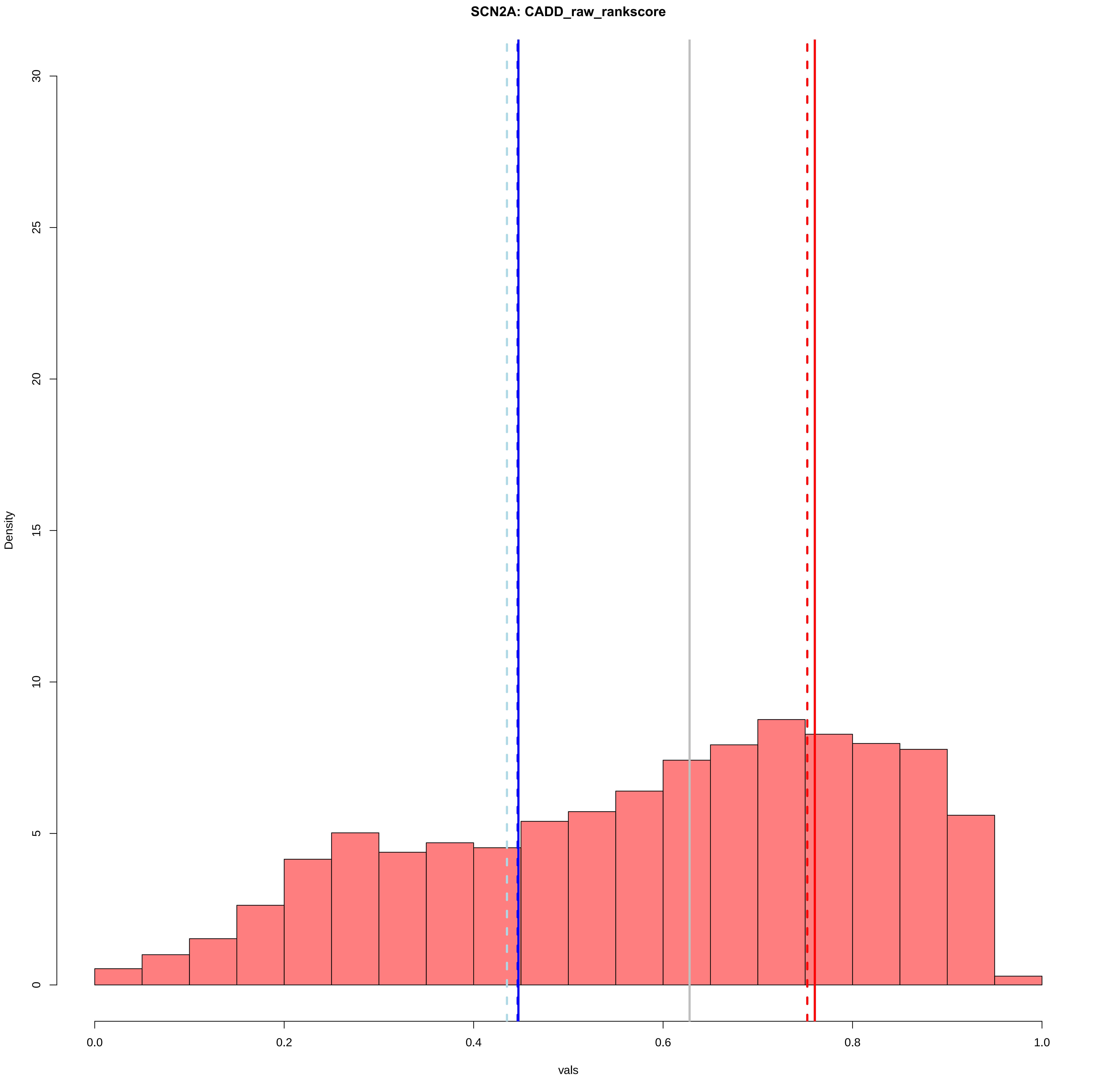

SCN2A: CADD_raw_rankscore
Density
vals
0
5
10
15
20
25
30
0.0
0.2
0.4
0.6
0.8
1.0

SCN2A: DANN_rankscore

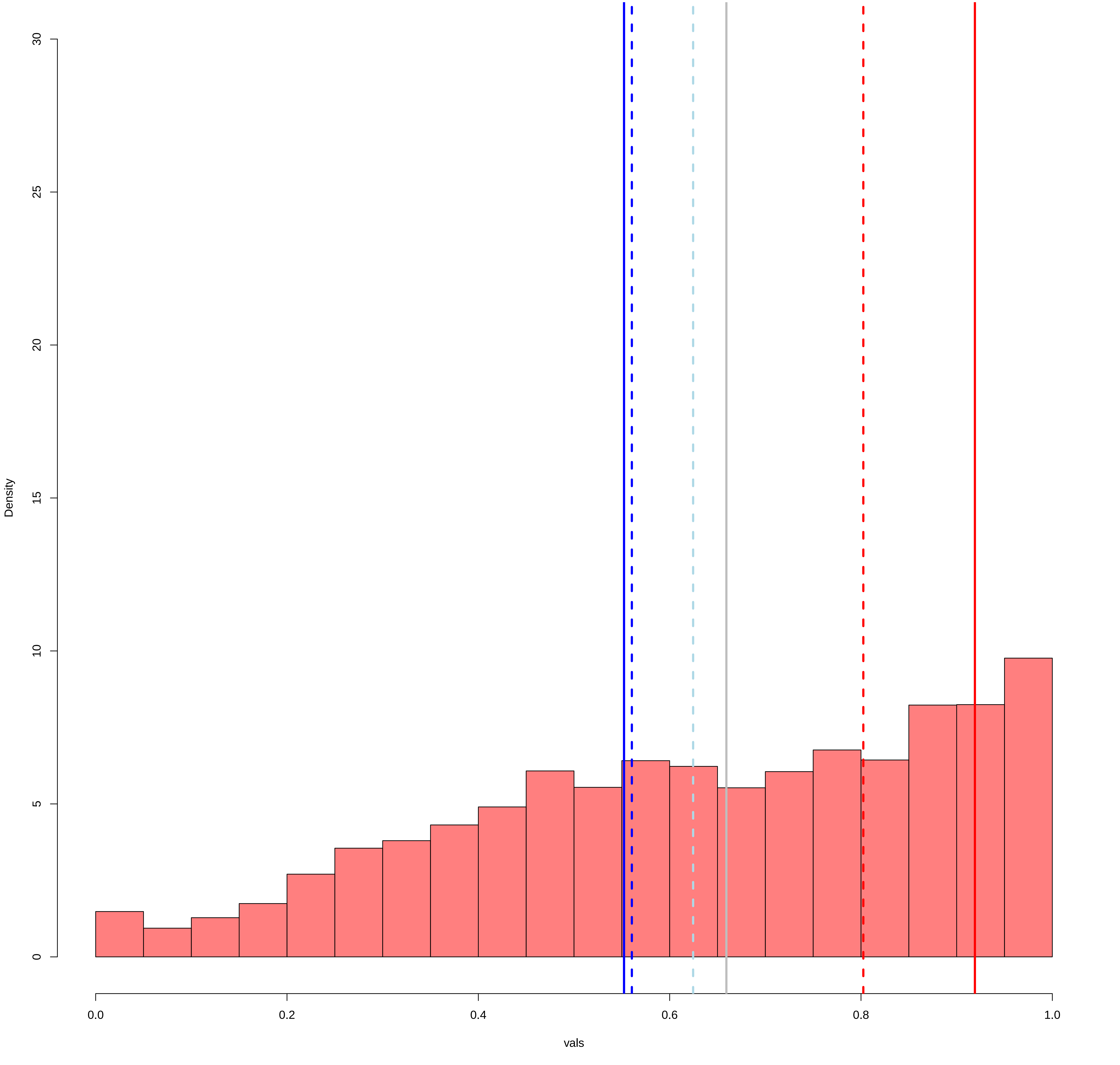

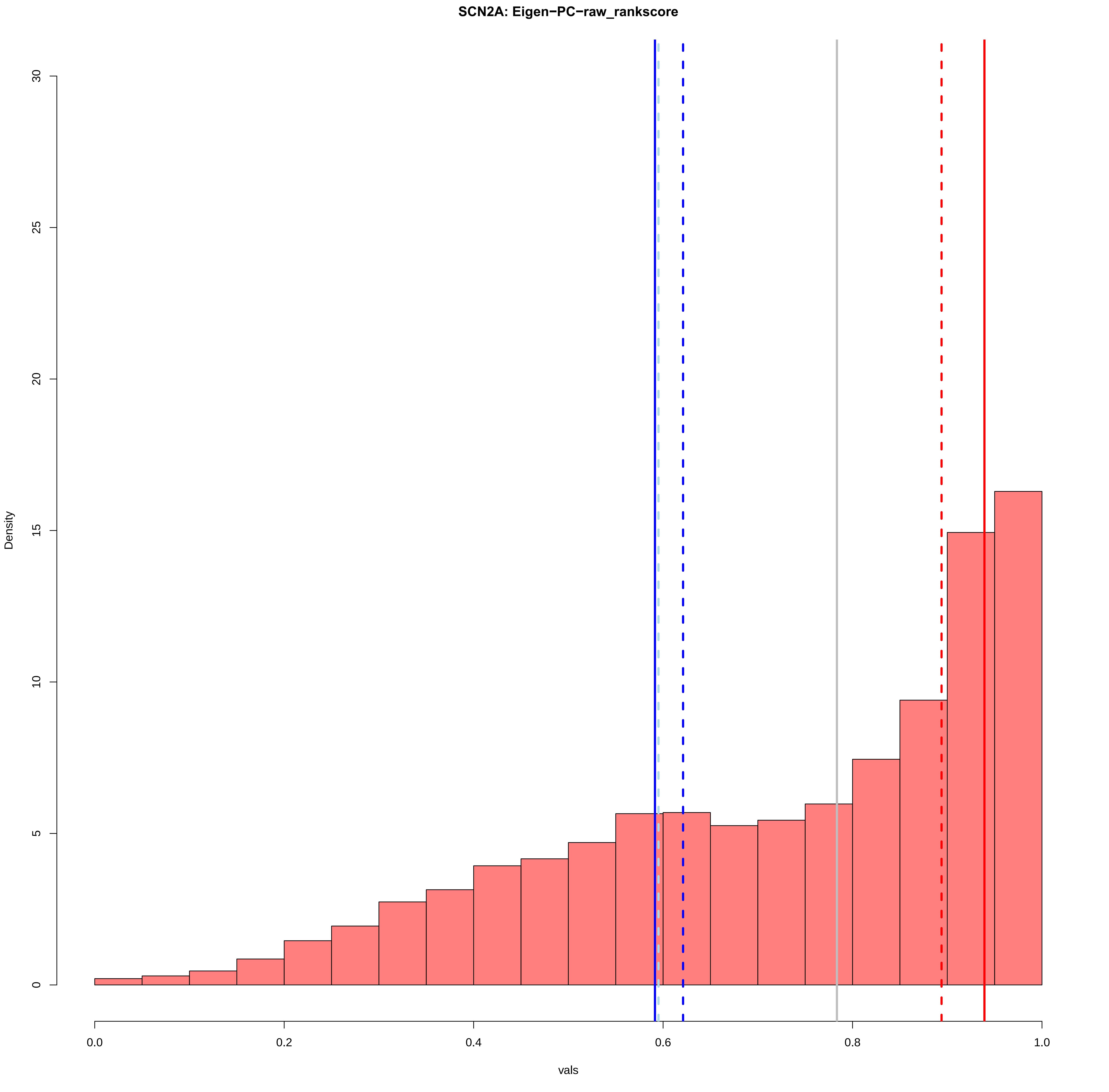

SCN2A: Eigen−PC−raw_rankscore
Density
vals
0
5
10
15
20
25
30
0.0
0.2
0.4
0.6
0.8
1.0

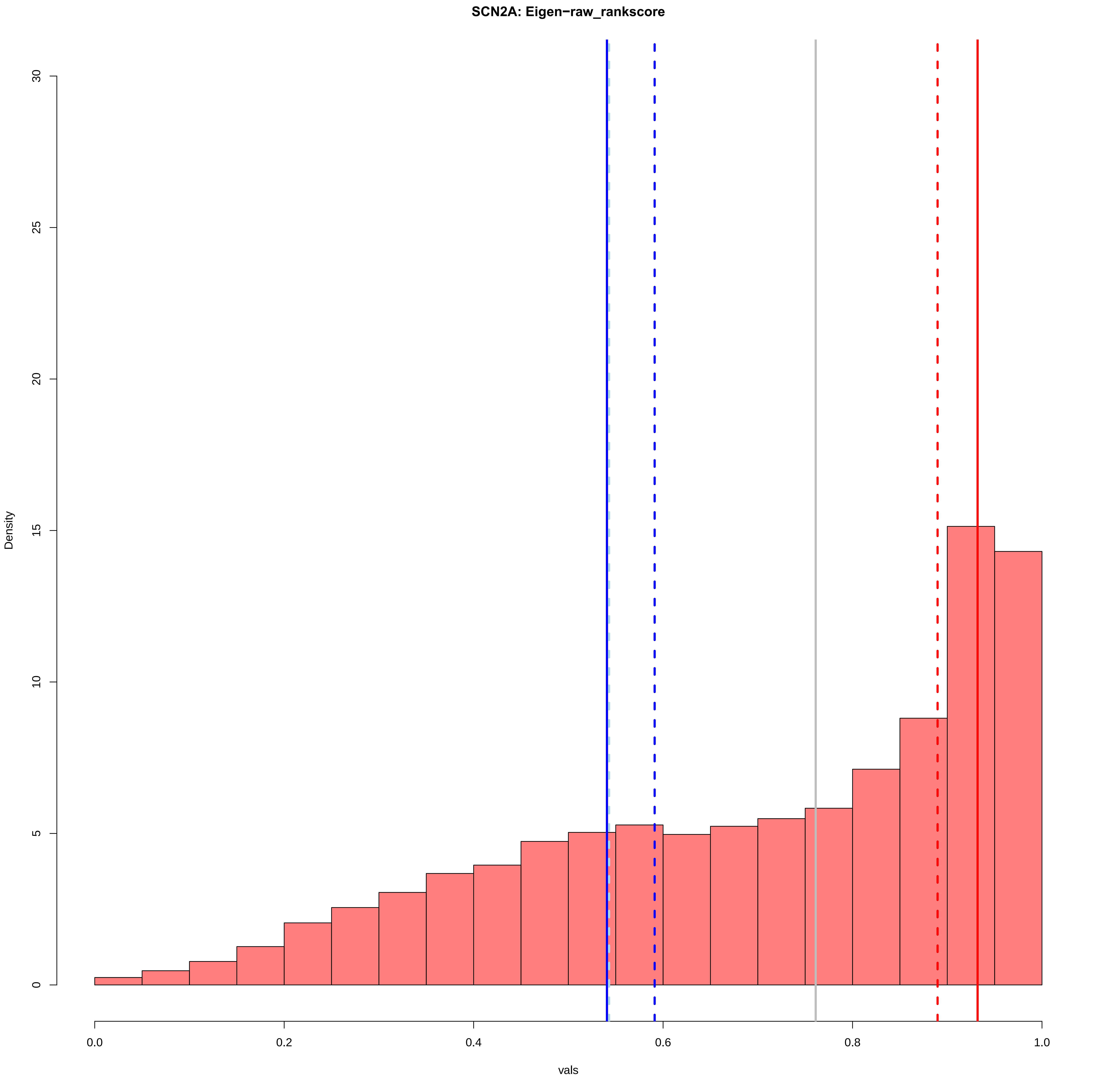
SCN2A: Eigen−raw_rankscore
Density
vals
0
5
10
15
20
25
30
0.0
0.2
0.4
0.6
0.8
1.0

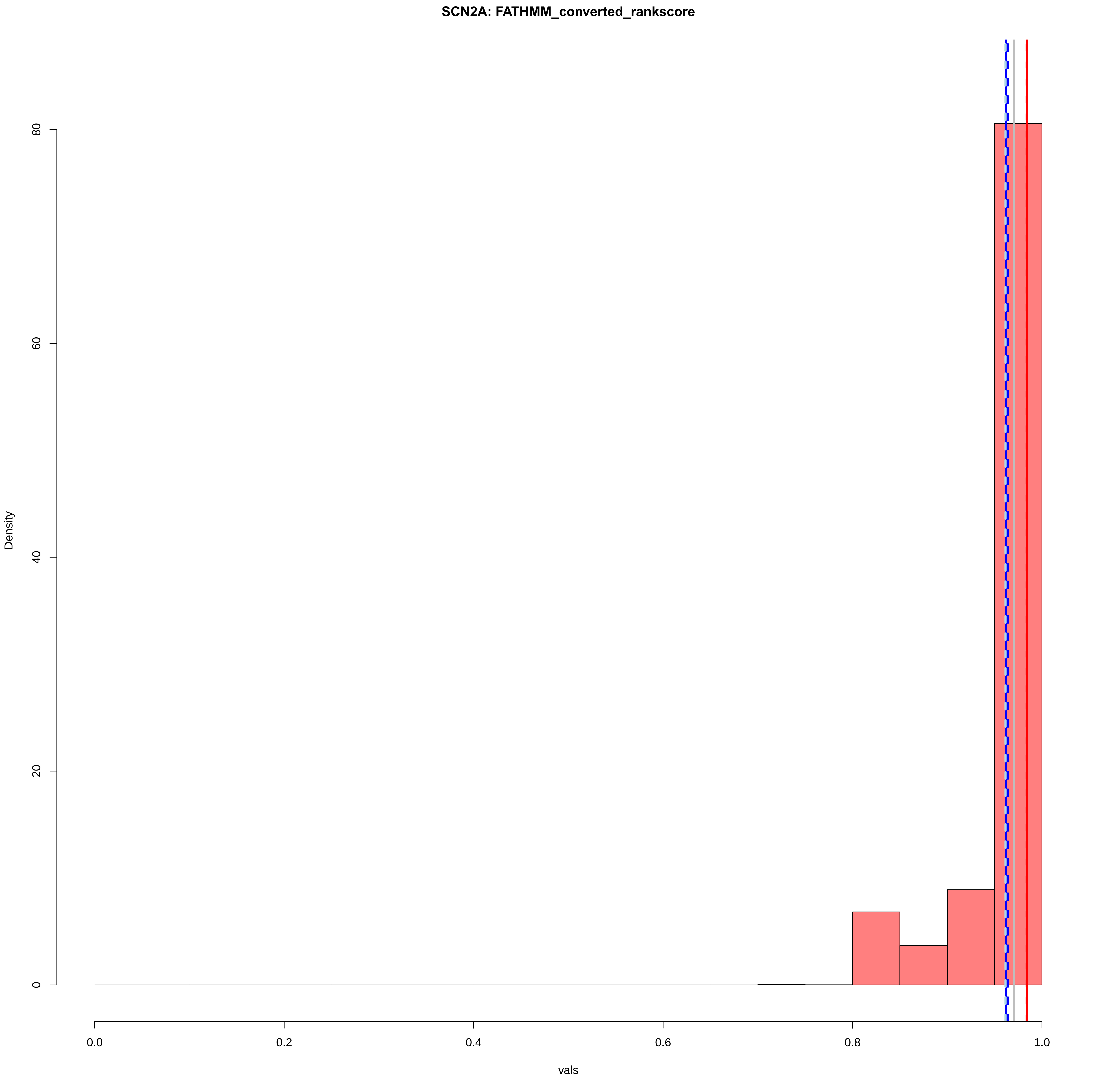

SCN2A: FATHMM_converted_rankscore
Density
vals
0
20
40
60
80
0.0
0.2
0.4
0.6
0.8
1.0

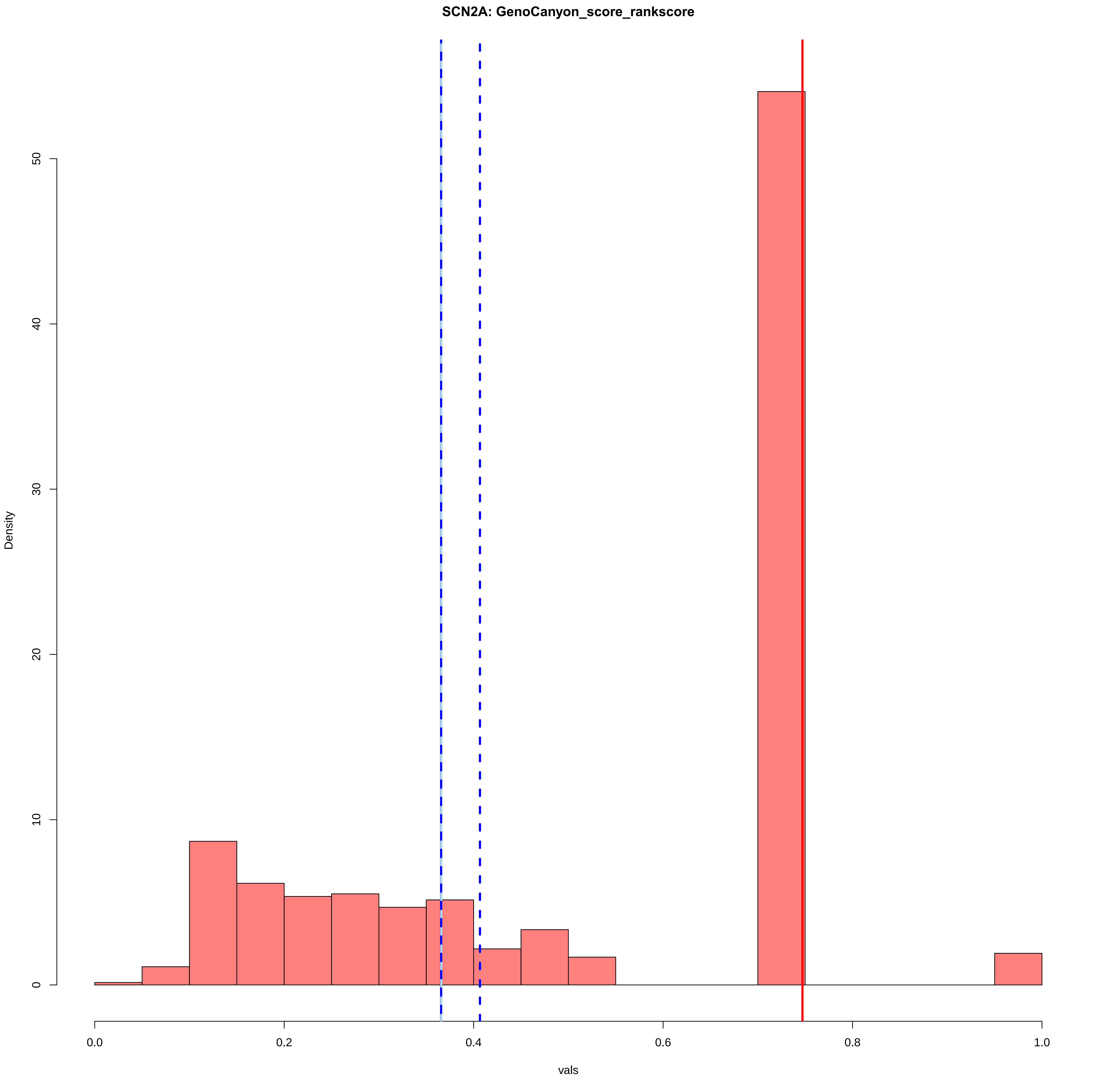
SCN2A: GenoCanyon_score_rankscore
Density
0
10
20
30
40
50
0.0
0.2
0.4
0.6
0.8
1.0
vals

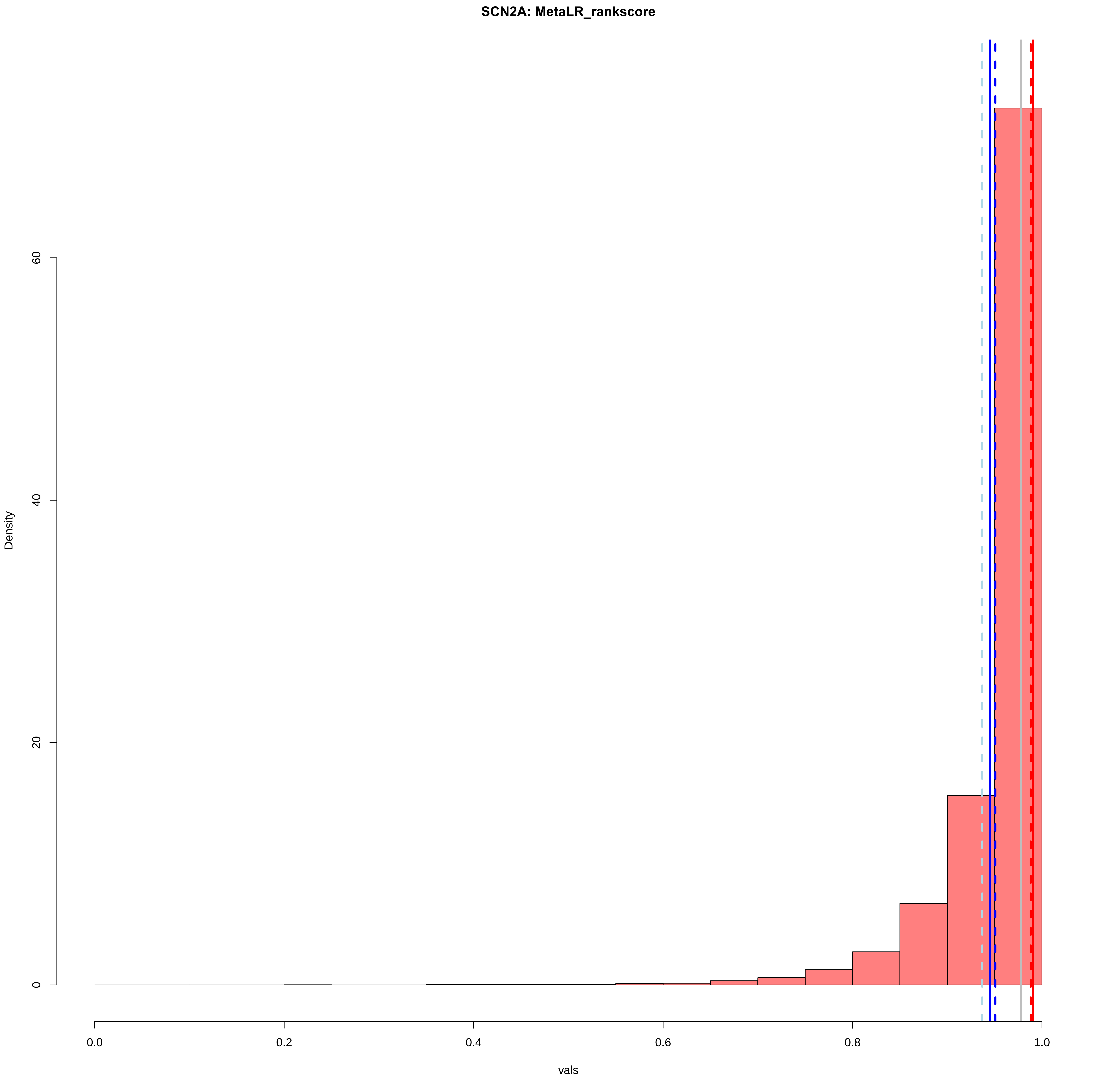

SCN2A: MetaLR_rankscore
Density
0
20
40
60
0.0
0.2
0.4
0.6
0.8
1.0
vals

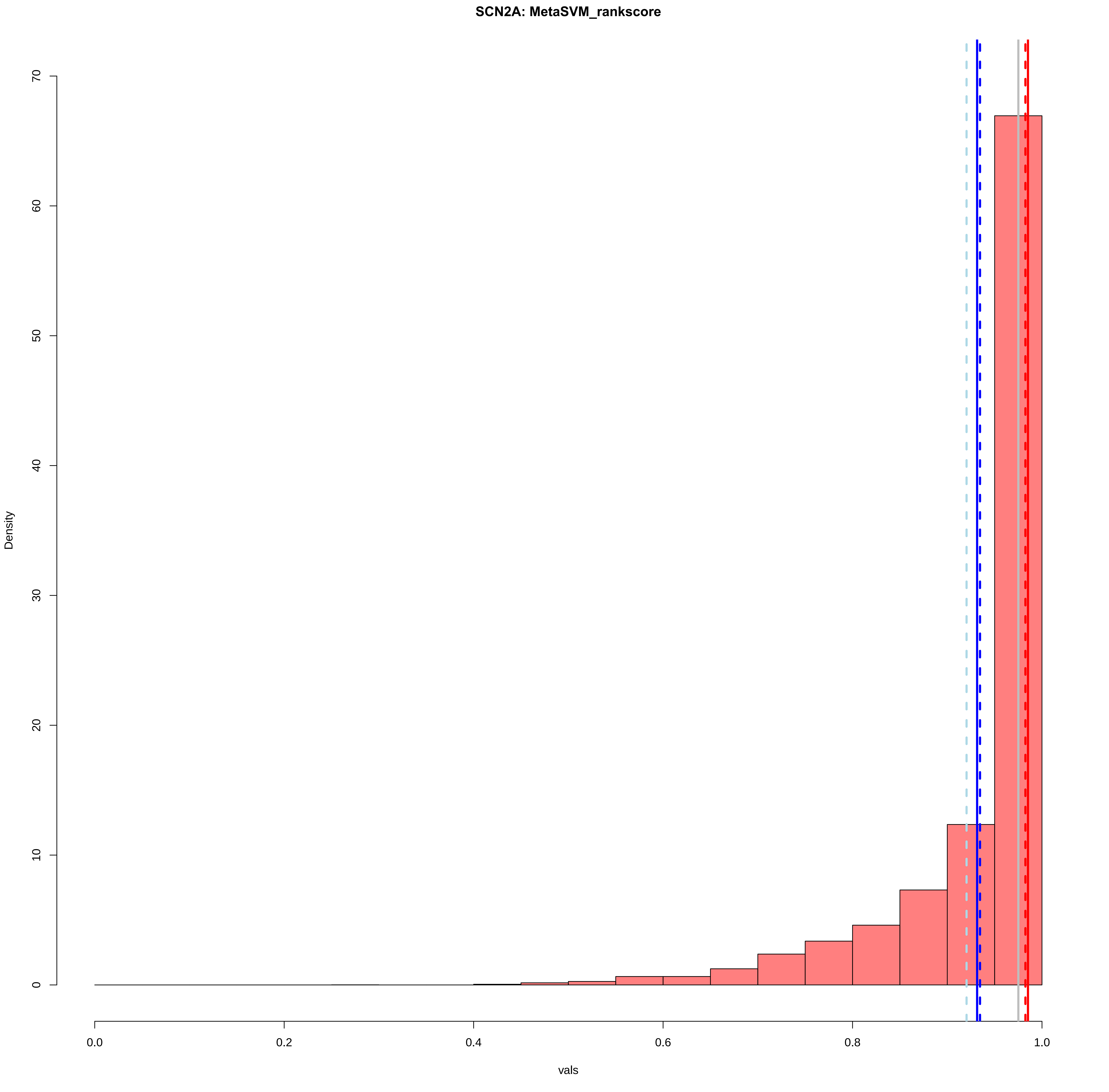
SCN2A: MetaSVM_rankscore
Density
vals
0
10
20
30
40
50
60
70
0.0
0.2
0.4
0.6
0.8
1.0

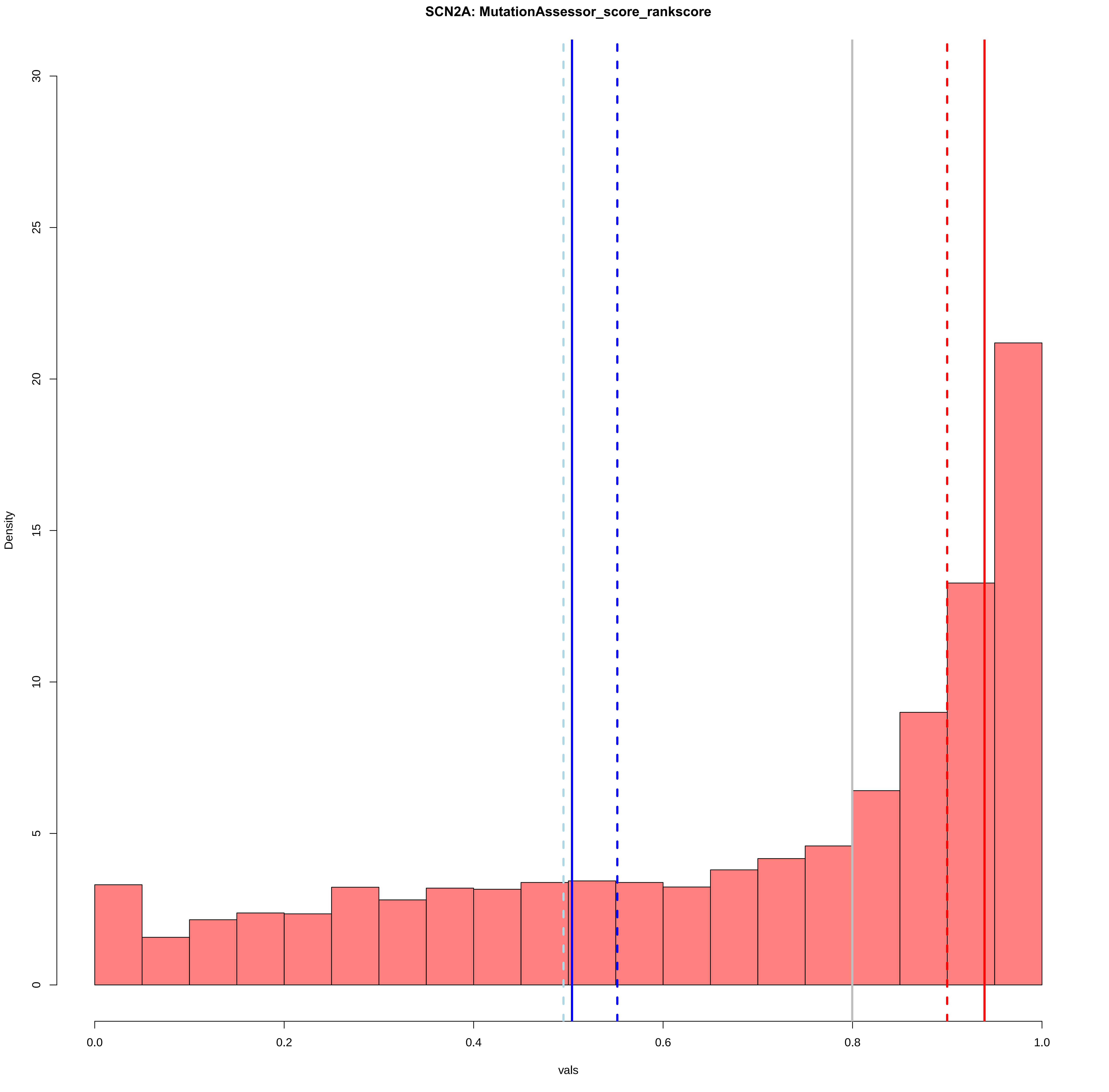
SCN2A: MutationAssessor_score_rankscore
Density
vals
0
5
10
15
20
25
30
0.0
0.2
0.4
0.6
0.8
1.0

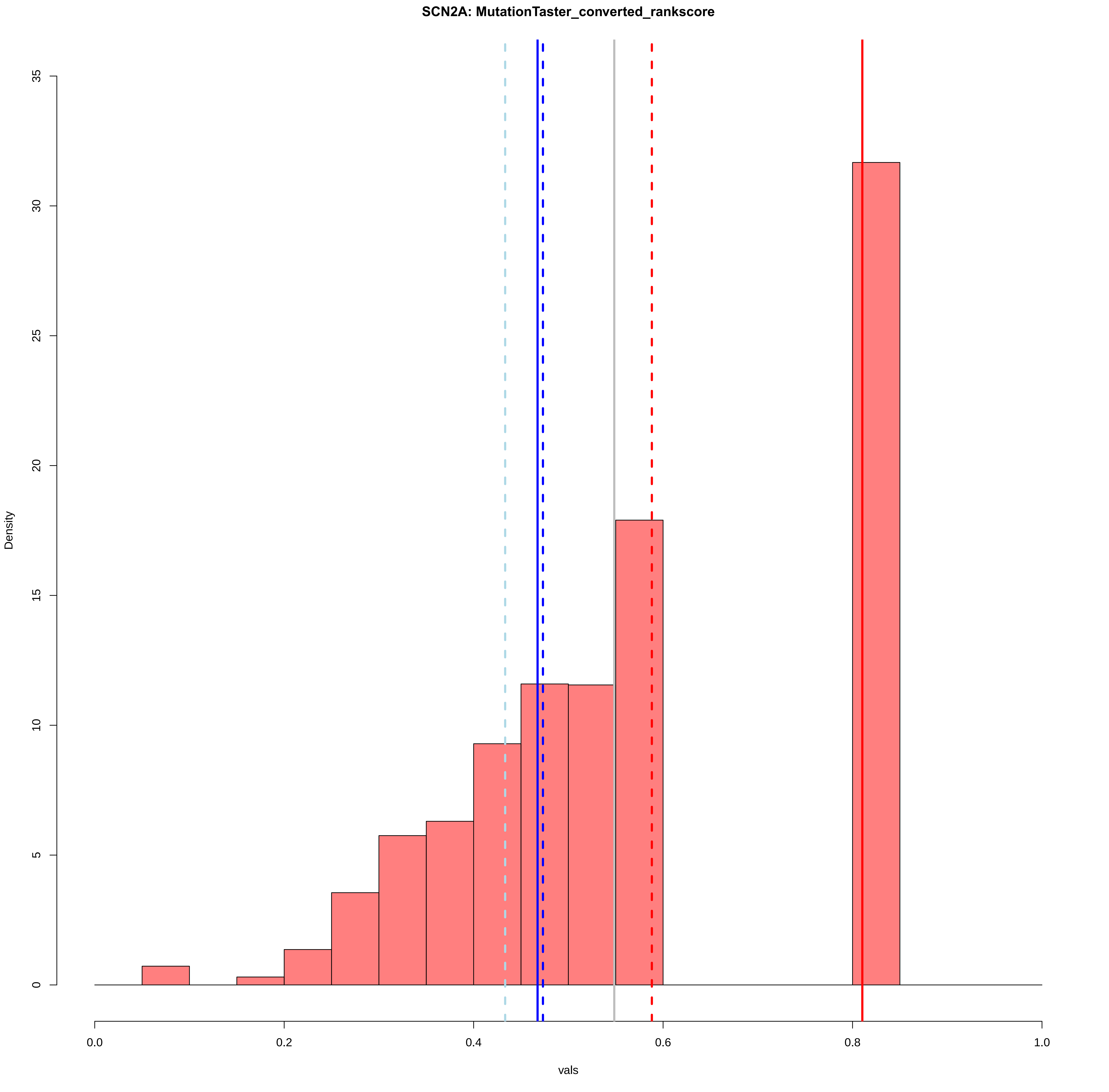
SCN2A: MutationTaster_converted_rankscore
Density
vals
0
5
10
15
20
25
30
35
0.0
0.2
0.4
0.6
0.8
1.0

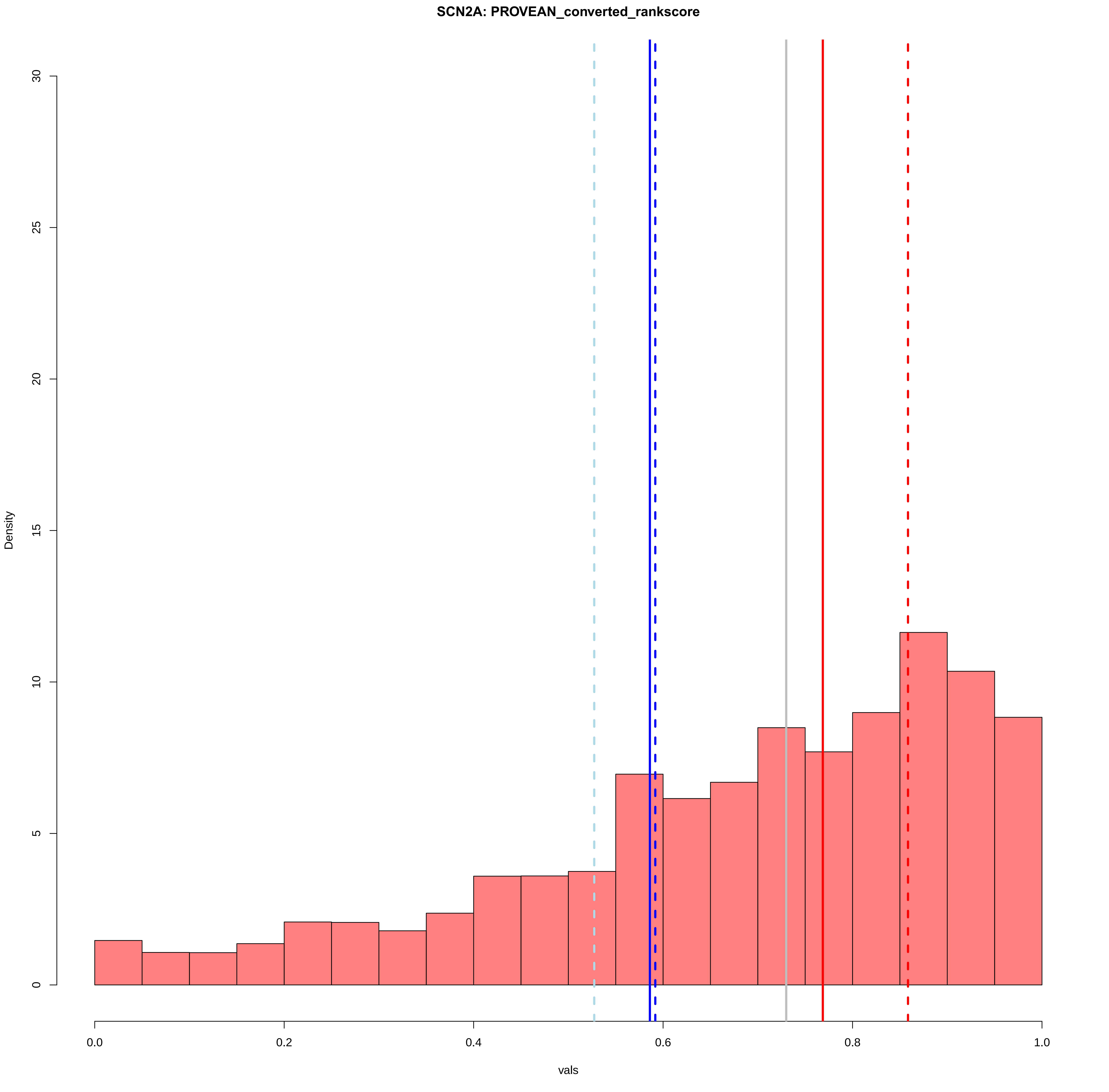

SCN2A: PROVEAN_converted_rankscore
Density
0
5
10
15
20
25
30
0.0
0.2
0.4
0.6
0.8
1.0
vals

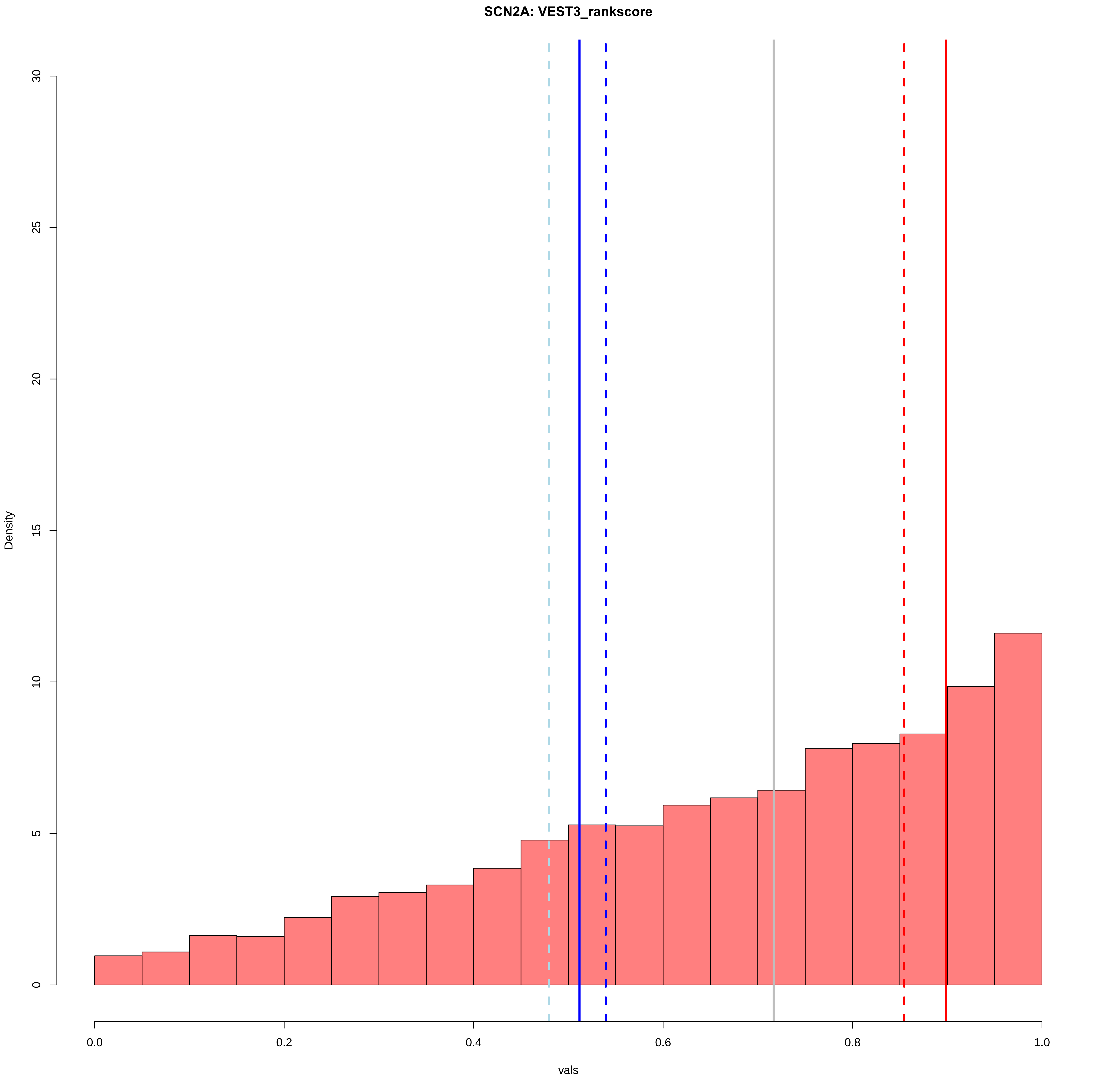

SCN2A: VEST3_rankscore
Density
vals
0
5
10
15
20
25
30
0.0
0.2
0.4
0.6
0.8
1.0

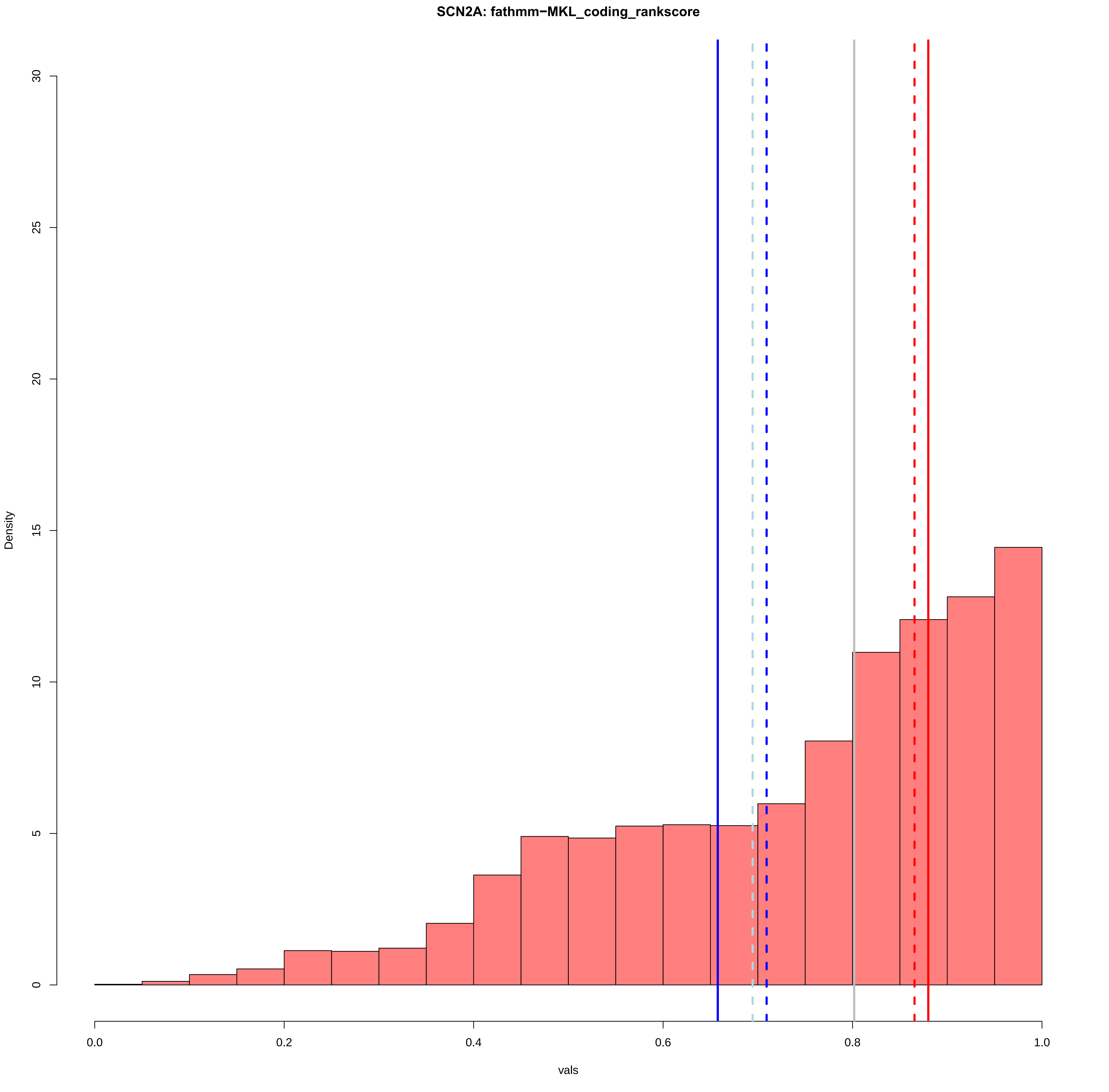

SCN2A: fathmm−MKL_coding_rankscore
Density
vals
0
5
10
15
20
25
30
0.0
0.2
0.4
0.6
0.8
1.0

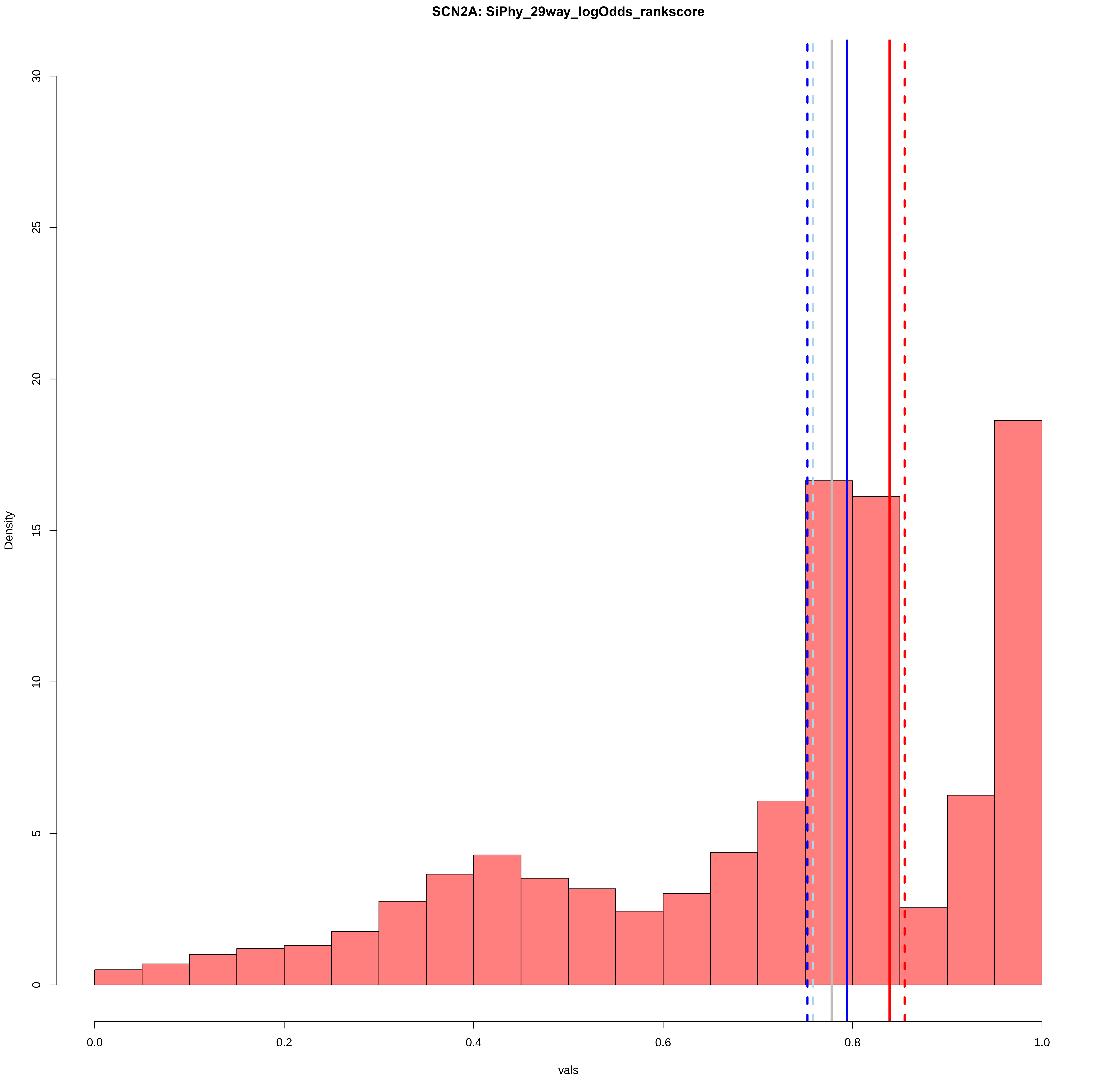
SCN2A: SiPhy_29way_logOdds_rankscore
Density
vals
0
5
10
15
20
25
30
0.0
0.2
0.4
0.6
0.8
1.0

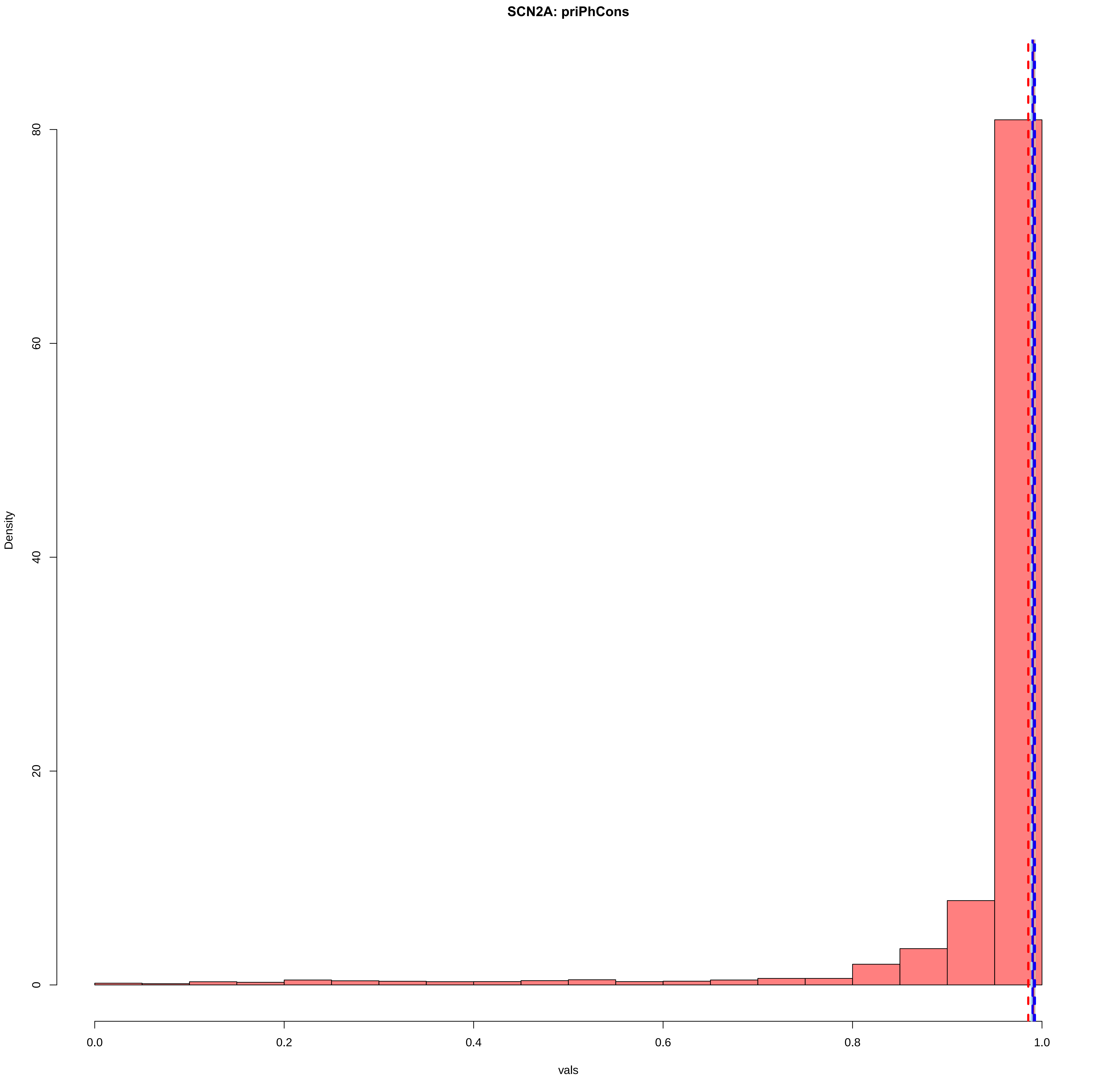
SCN2A: priPhCons
Density
vals
0
20
40
60
80
0.0
0.2
0.4
0.6
0.8
1.0

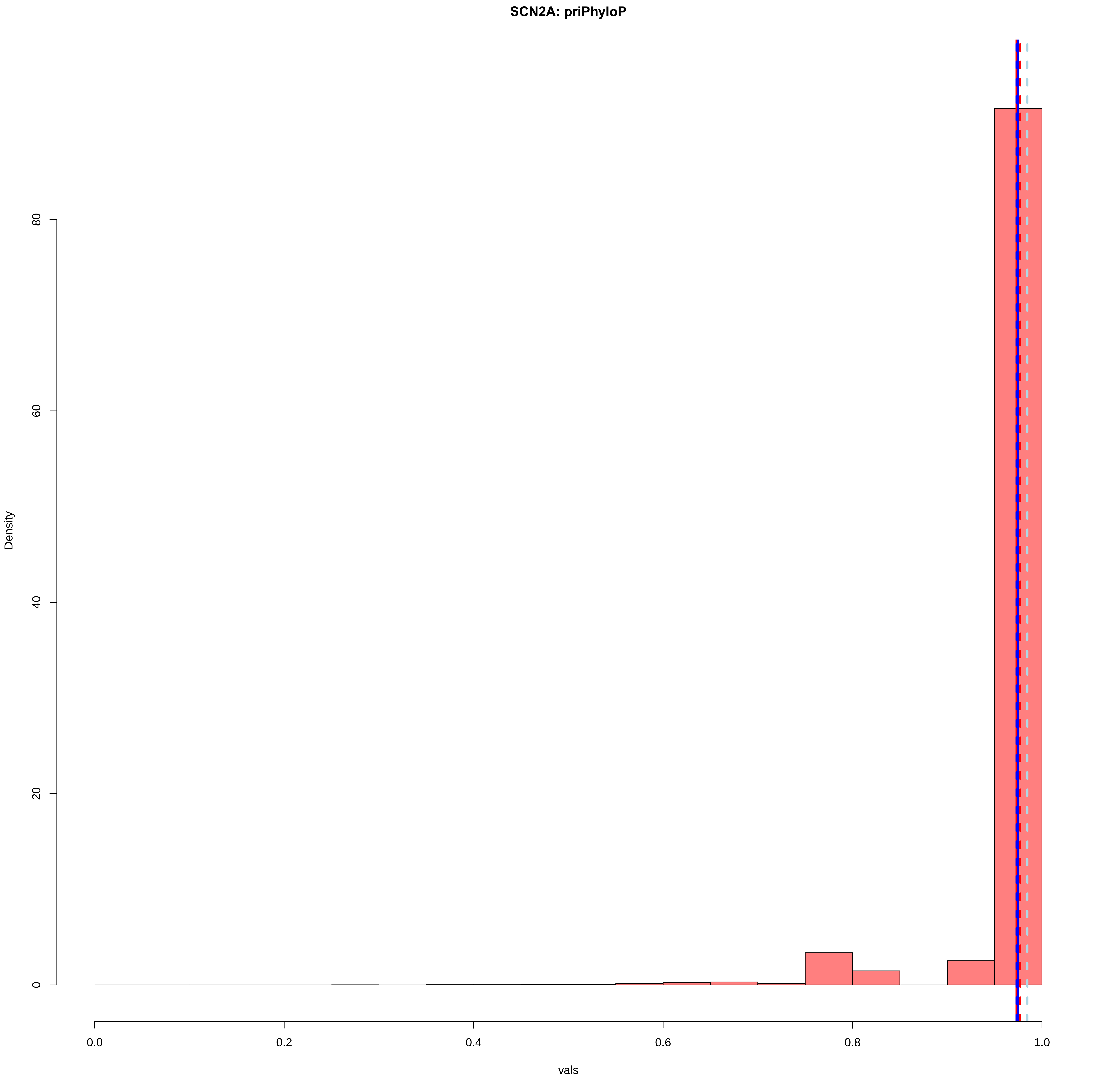

SCN2A: priPhyloP
Density
vals
0
20
40
60
80
0.0
0.2
0.4
0.6
0.8
1.0

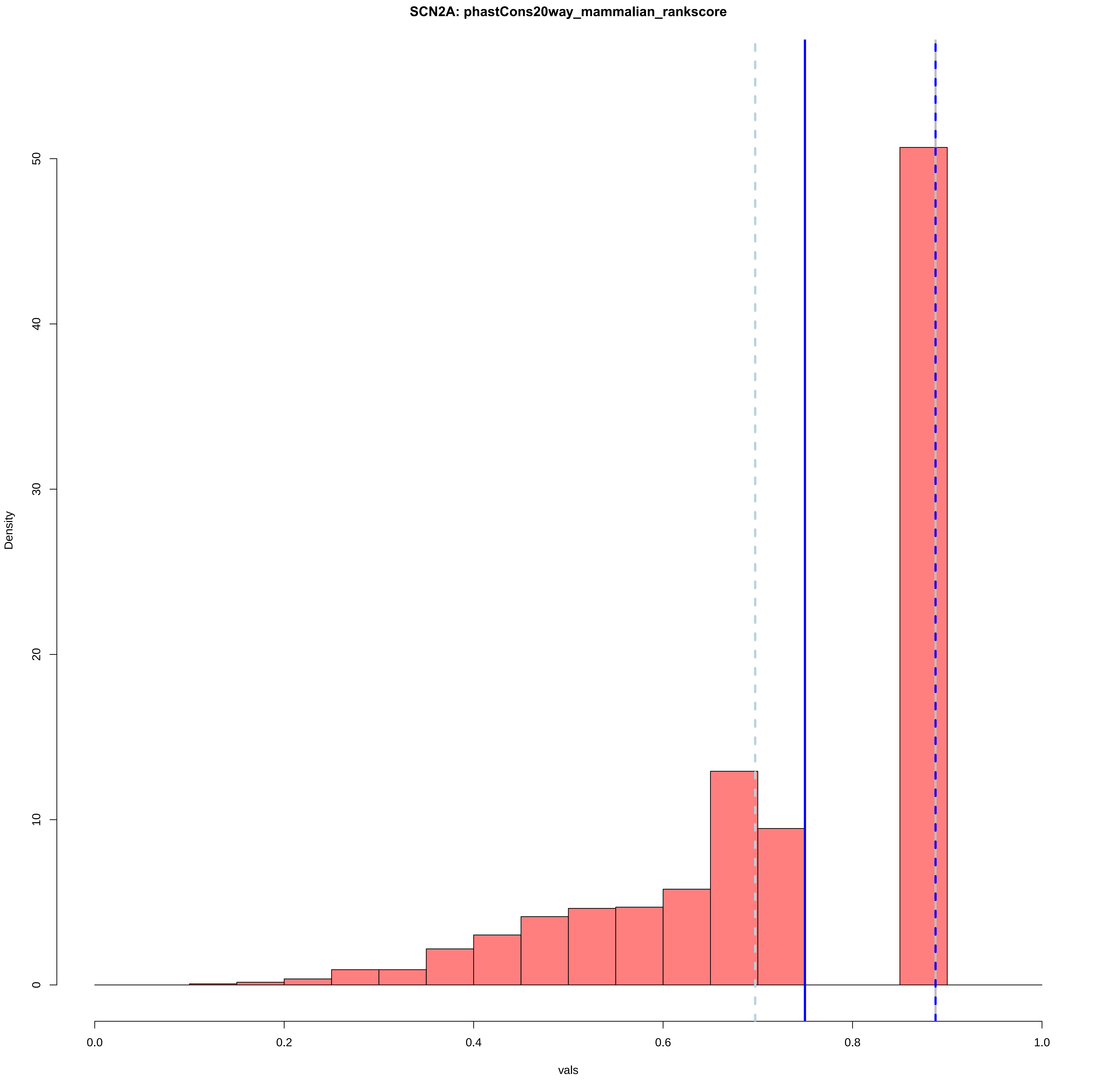
SCN2A: phastCons20way_mammalian_rankscore
Density
vals
0
10
20
30
40
50
0.0
0.2
0.4
0.6
0.8
1.0

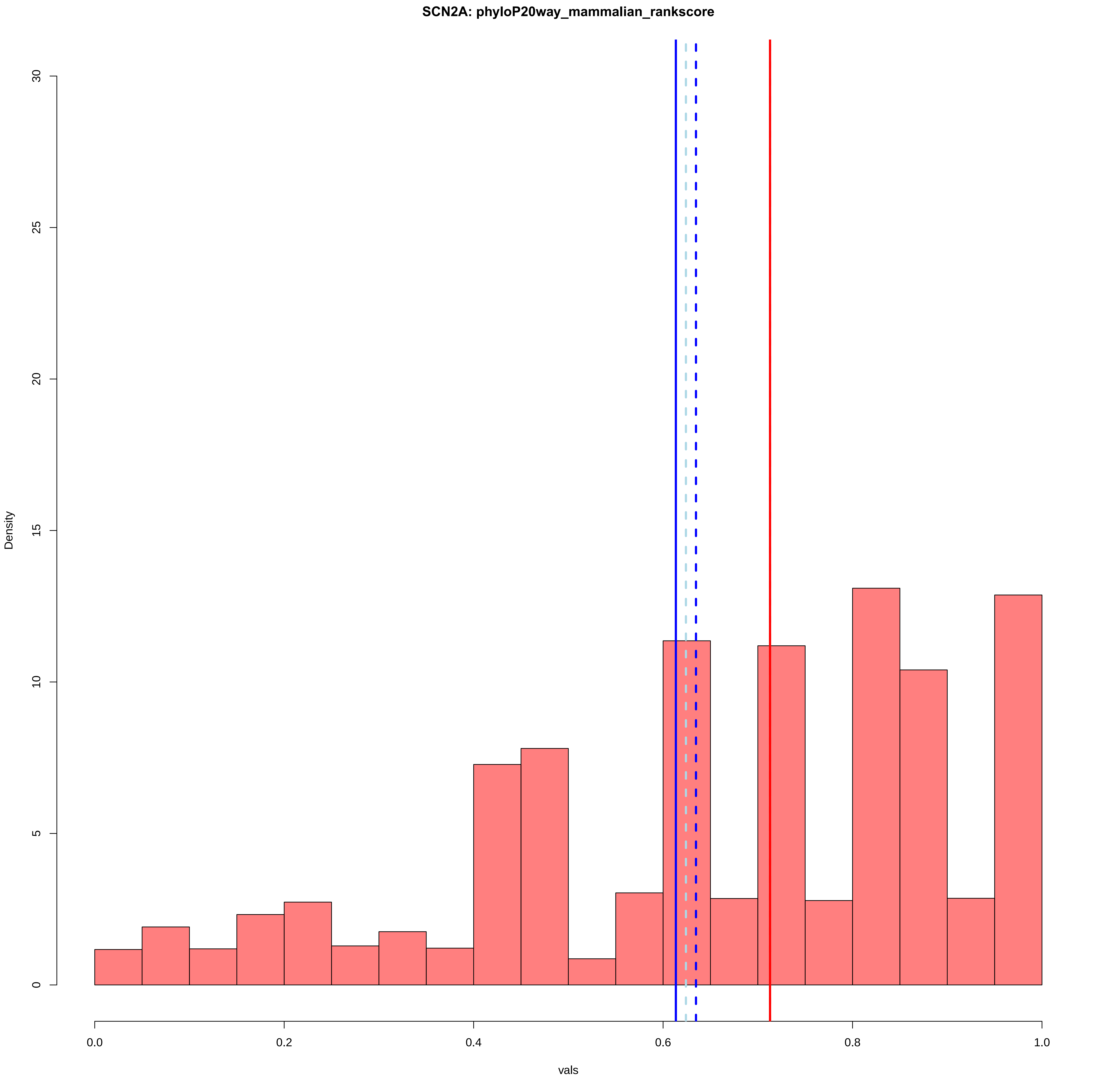

SCN2A: phyloP20way_mammalian_rankscore
Density
vals
0
5
10
15
20
25
30
0.0
0.2
0.4
0.6
0.8
1.0

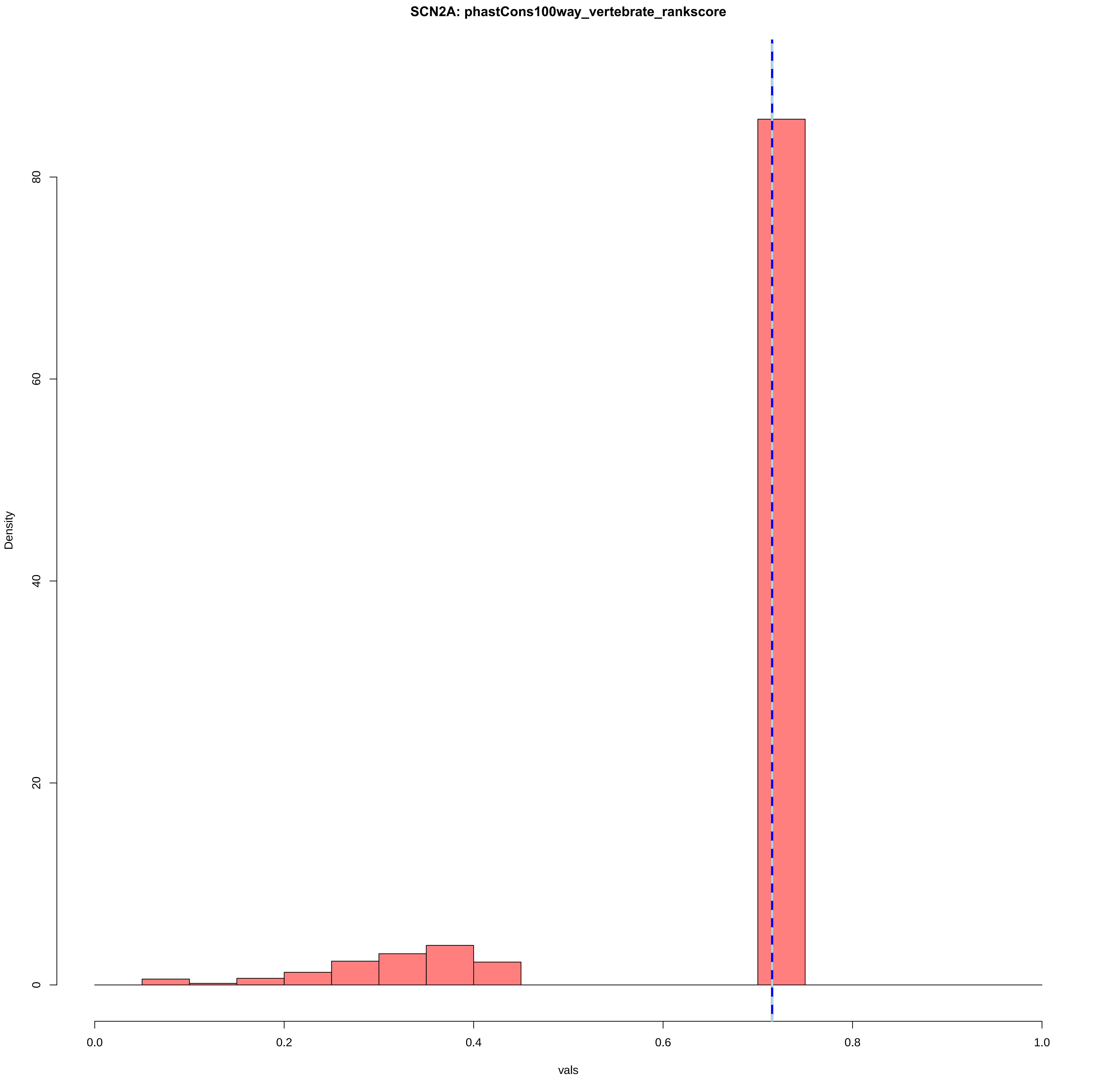

SCN2A: phastCons100way_vertebrate_rankscore
Density
vals
0
20
40
60
80
0.0
0.2
0.4
0.6
0.8
1.0

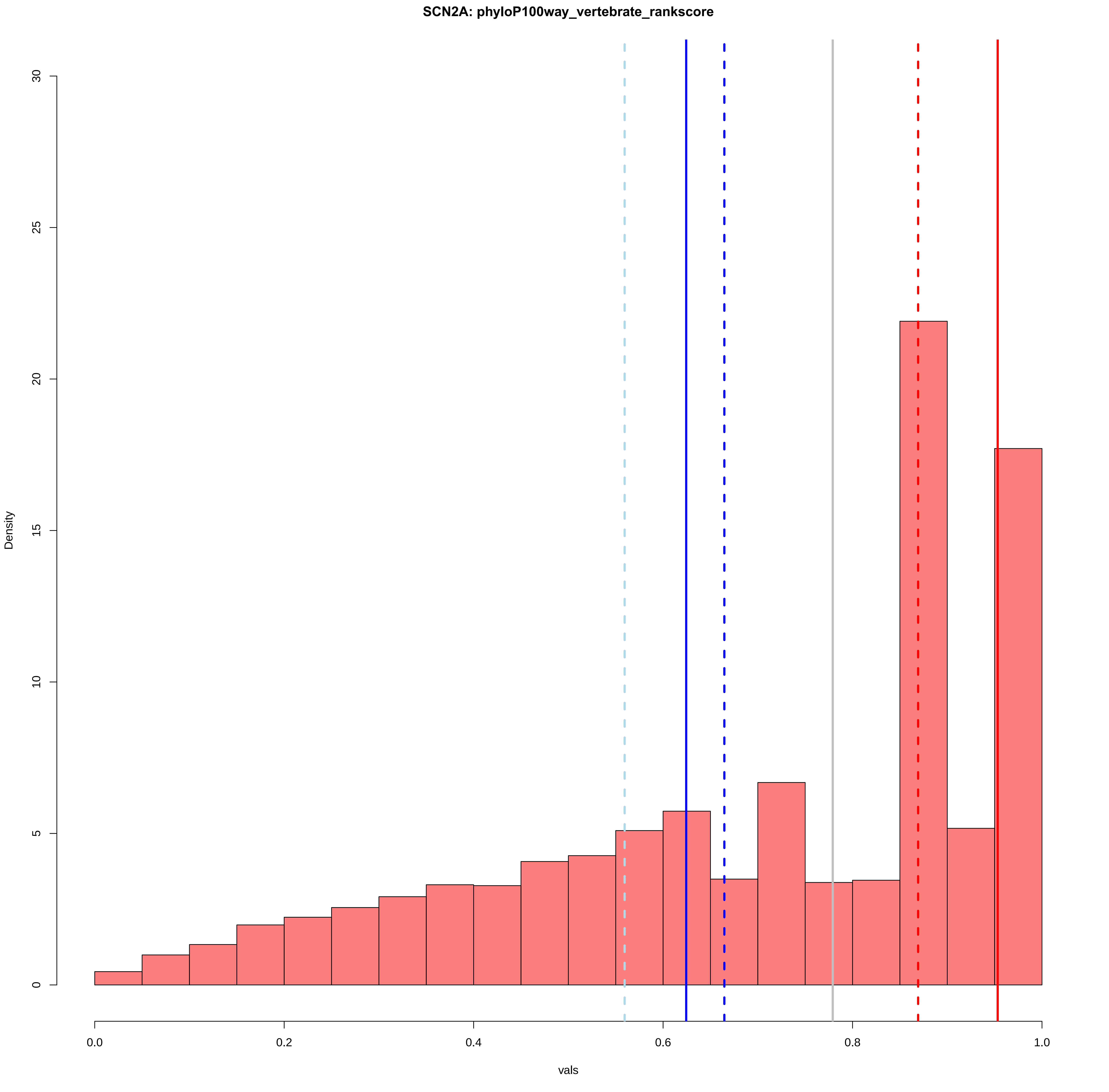
SCN2A: phyloP100way_vertebrate_rankscore
Density
vals
0
5
10
15
20
25
30
0.0
0.2
0.4
0.6
0.8
1.0

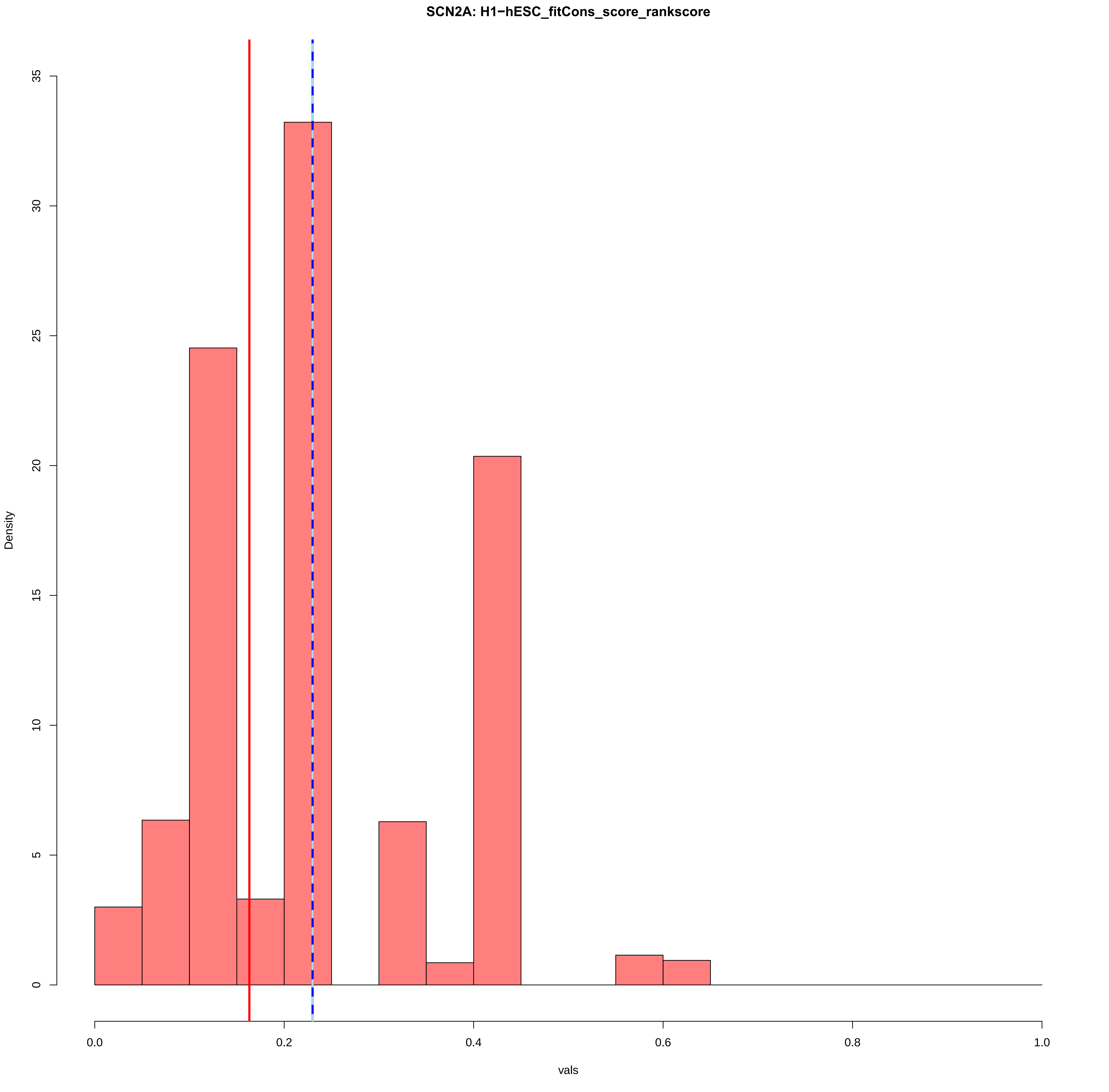
SCN2A: H1−hESC_fitCons_score_rankscore
Density
vals
0
5
10
15
20
25
30
35
0.0
0.2
0.4
0.6
0.8
1.0

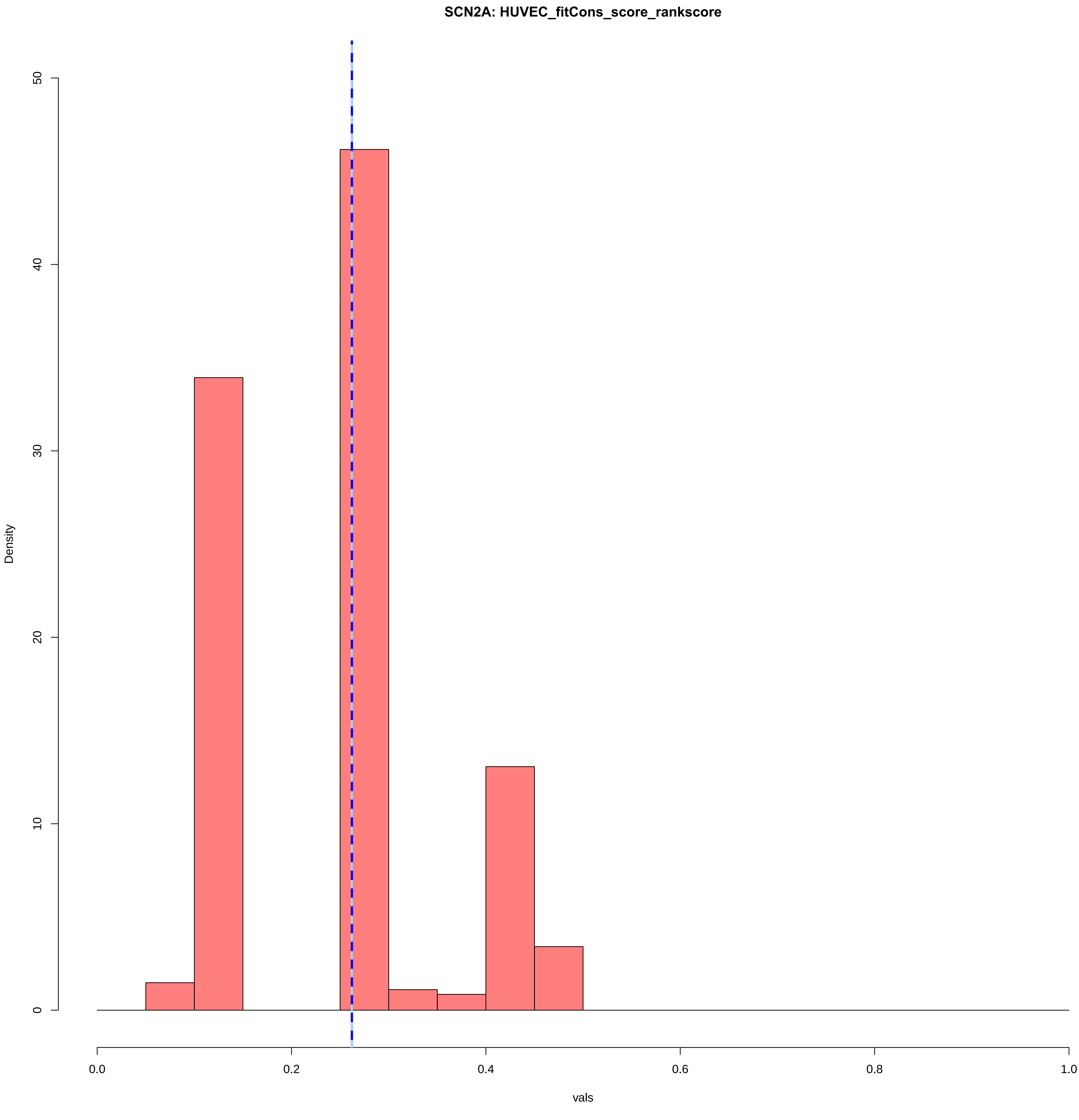

SCN2A: HUVEC_fitCons_score_rankscore
Density
vals
0
10
20
30
40
50
0.0
0.2
0.4
0.6
0.8
1.0

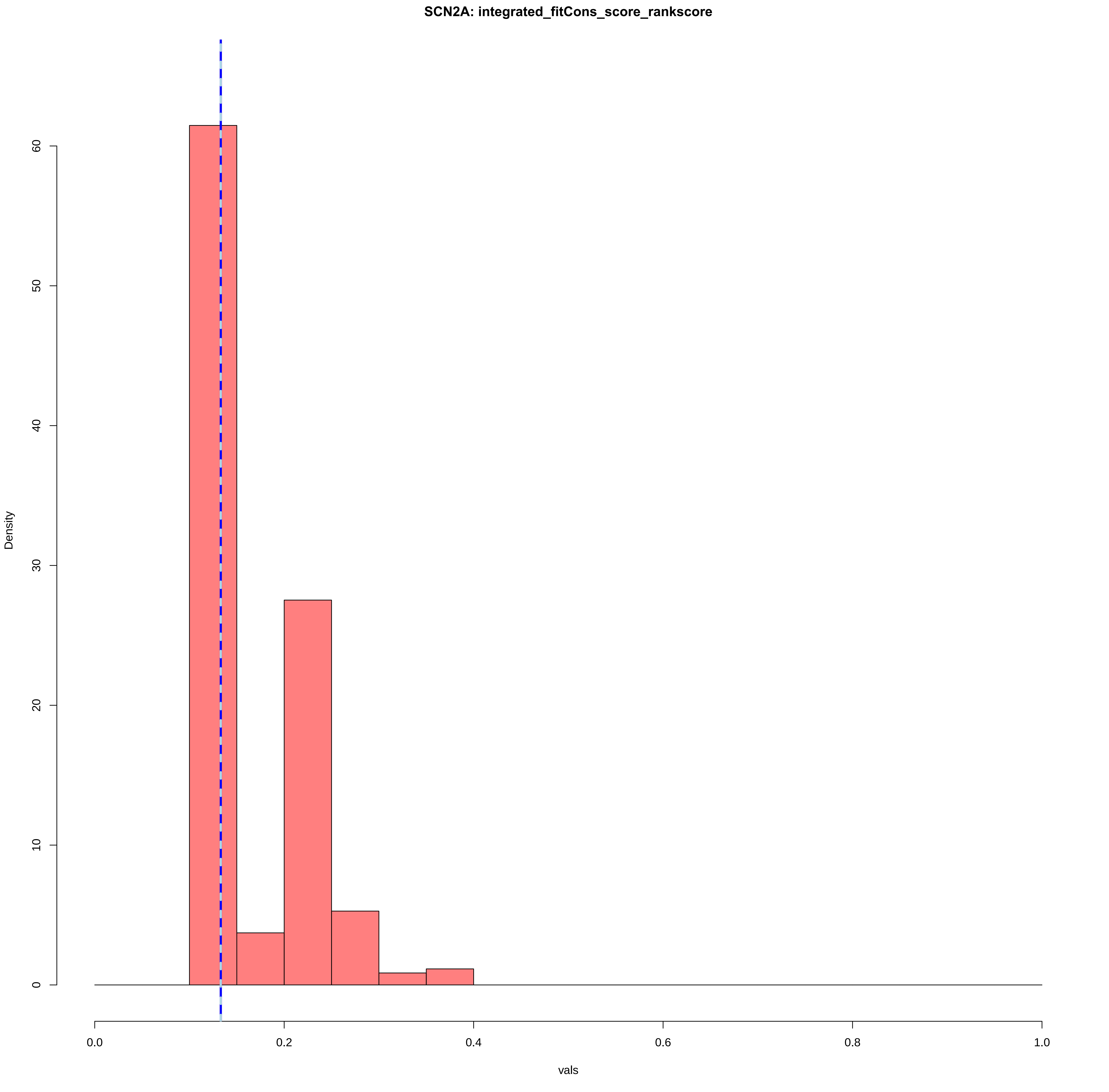

SCN2A: integrated_fitCons_score_rankscore
Density
0
10
20
30
40
50
60
0.0
0.2
0.4
0.6
0.8
1.0
vals

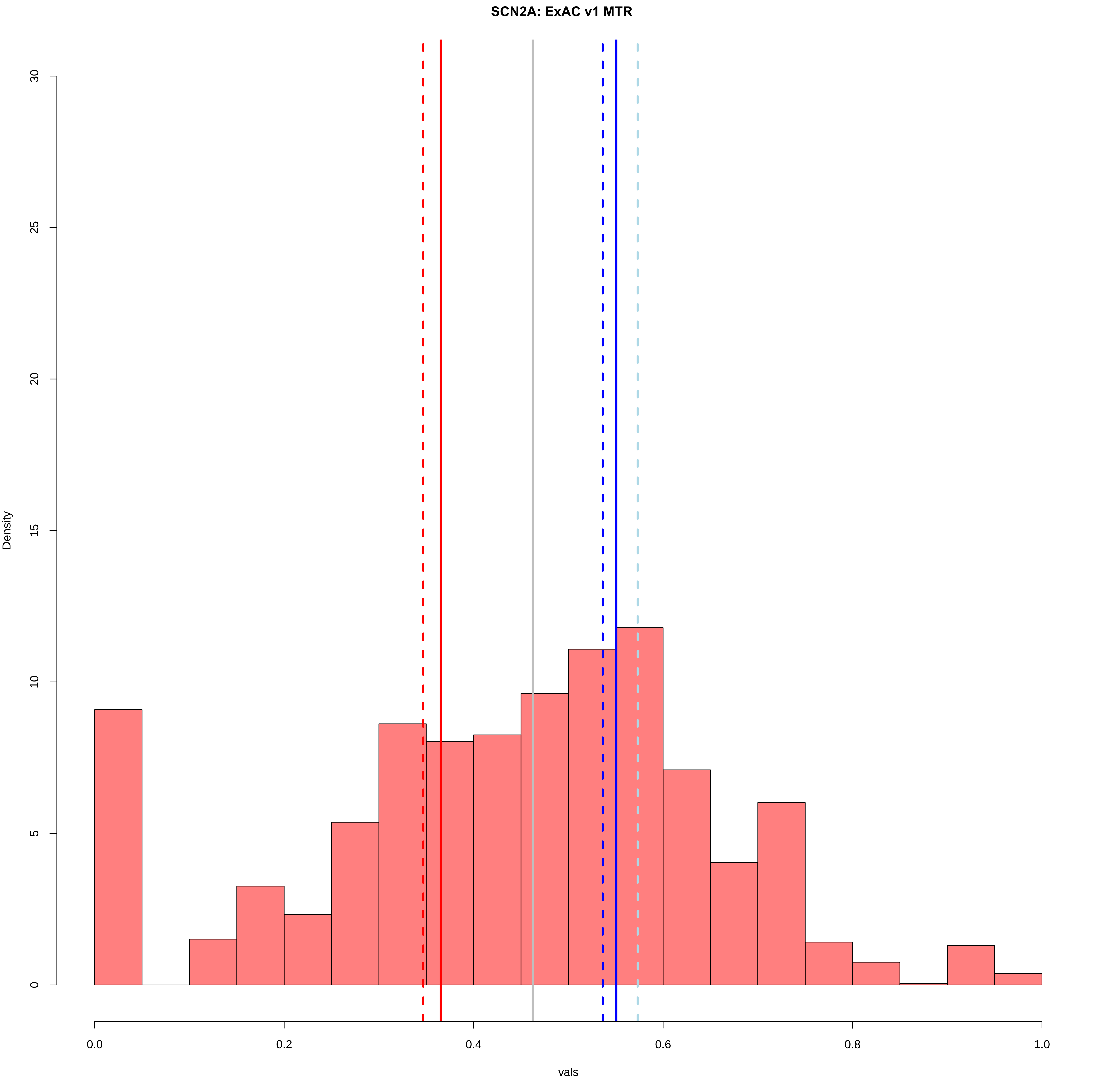

SCN2A: ExAC v1 MTR
Density
0
5
10
15
20
25
30
0.0
0.2
0.4
0.6
0.8
1.0
vals

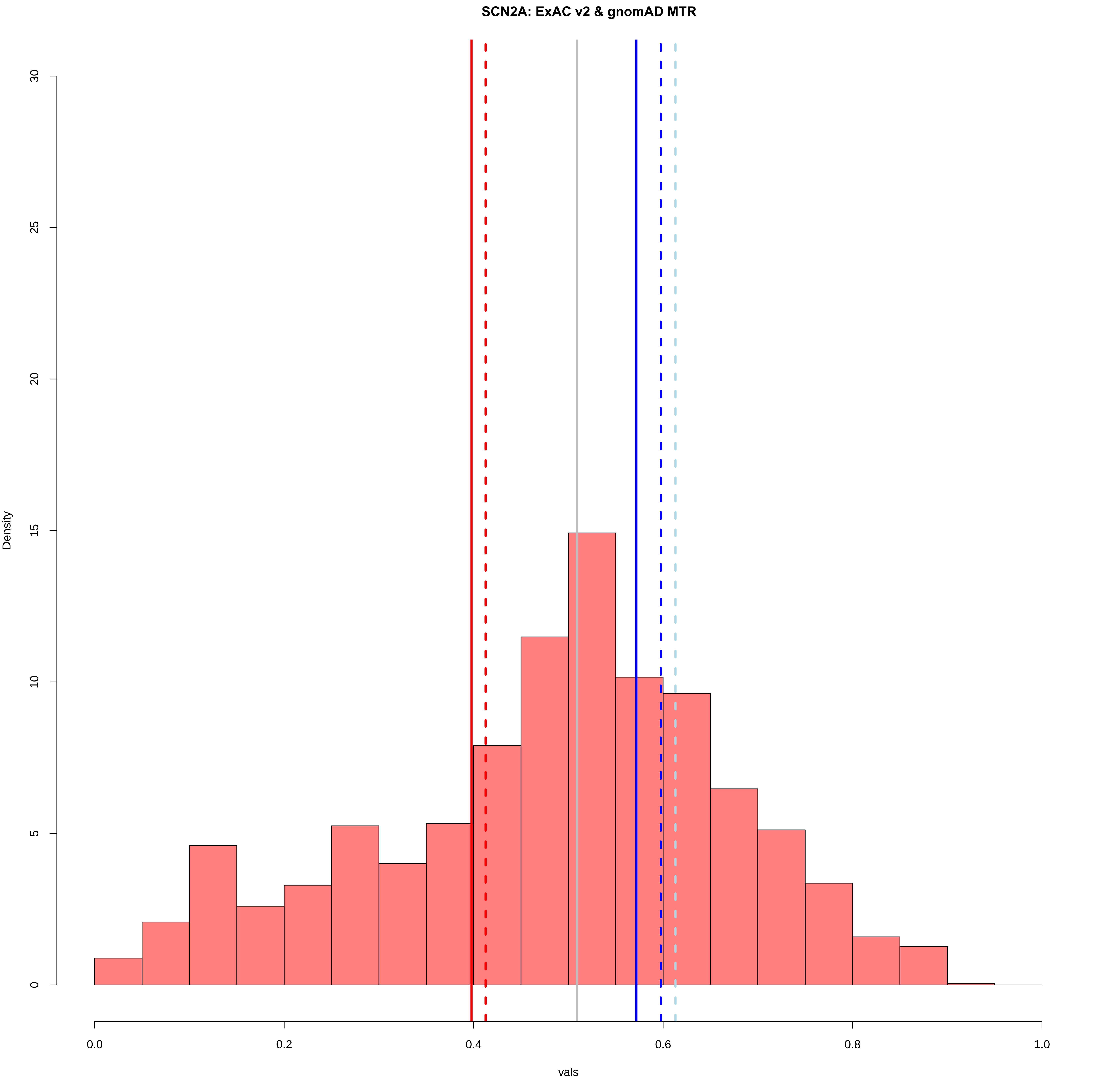

SCN2A: ExAC v2 & gnomAD MTR
Density
vals
0
5
10
15
20
25
30
0.0
0.2
0.4
0.6
0.8
1.0

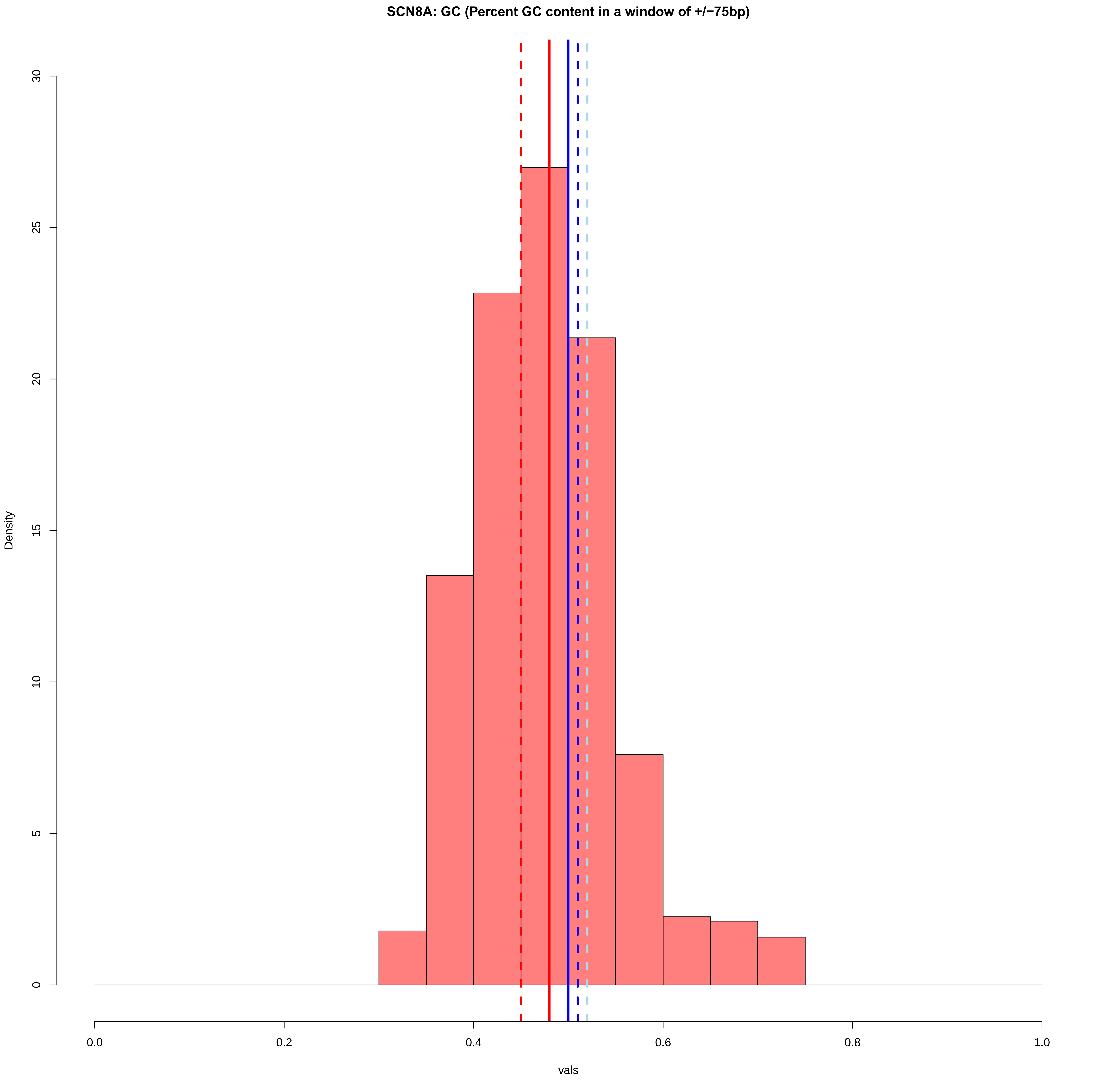
SCN8A: GC (Percent GC content in a window of +/−75bp)
Density
0
5
10
15
20
25
30
0.0
0.2
0.4
0.6
0.8
1.0
vals

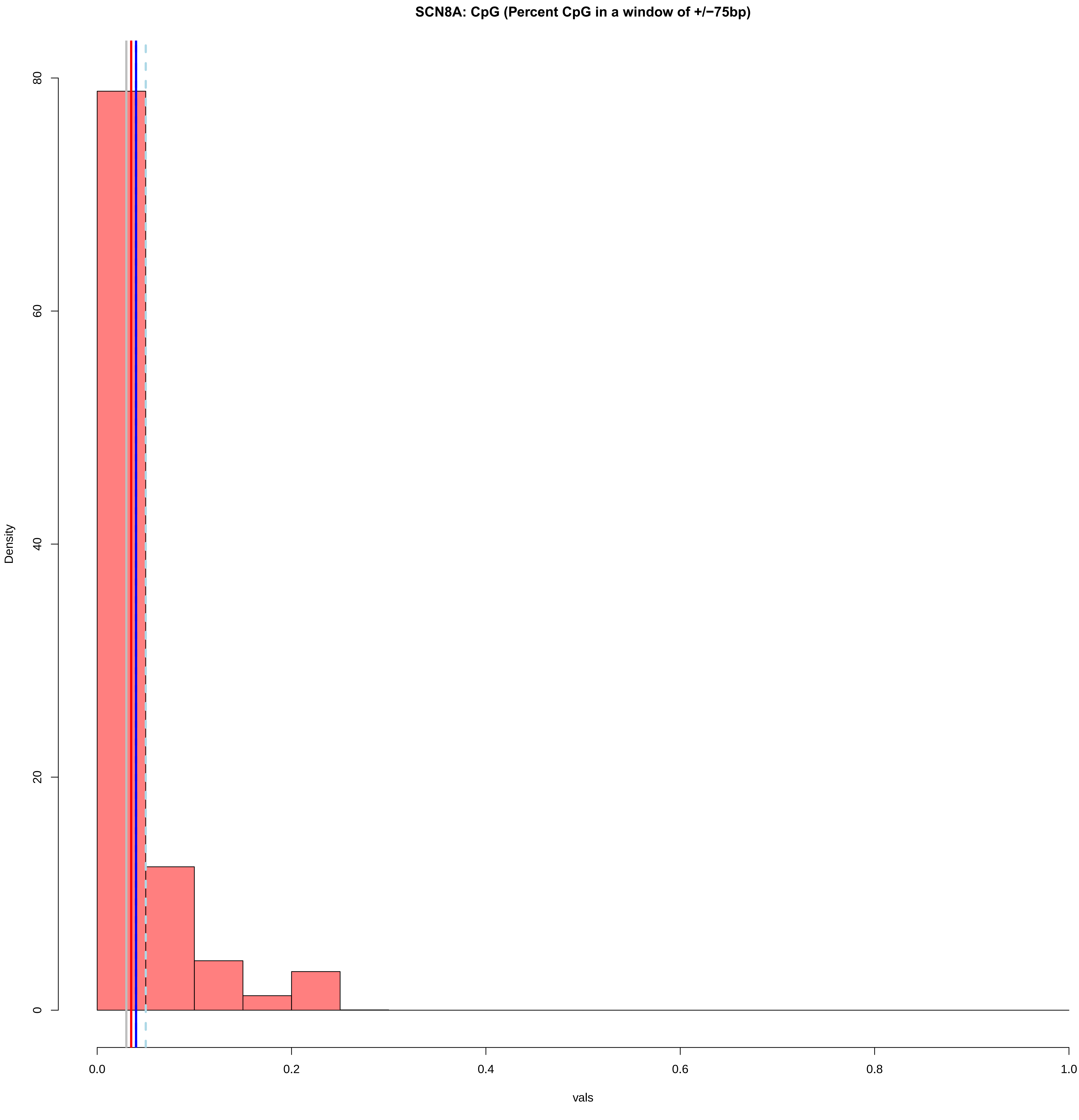
SCN8A: CpG (Percent CpG in a window of +/−75bp)
Density
0
20
40
60
80
0.0
0.2
0.4
0.6
0.8
1.0
vals

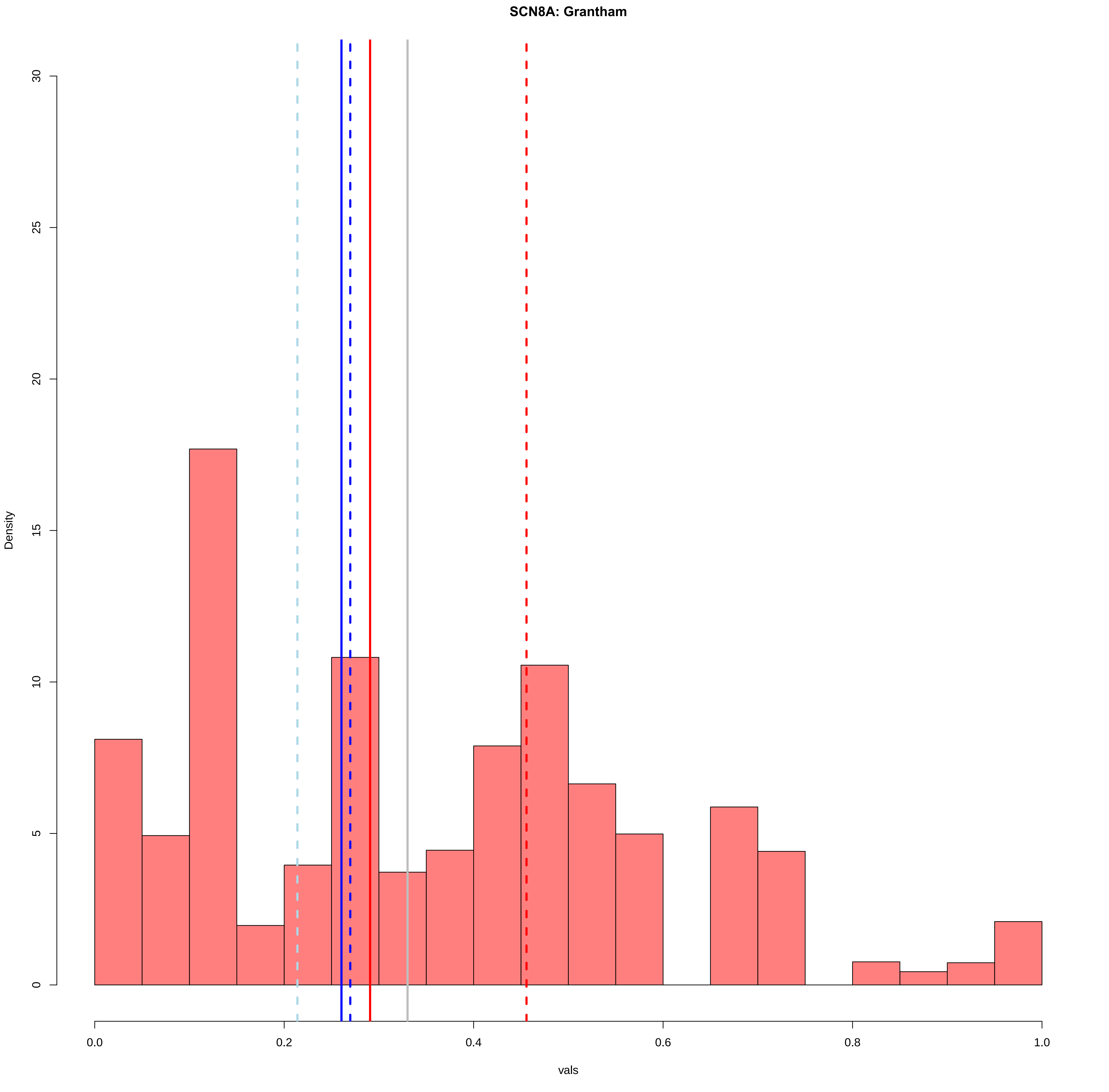

SCN8A: Grantham
Density
vals
0
5
10
15
20
25
30
0.0
0.2
0.4
0.6
0.8
1.0

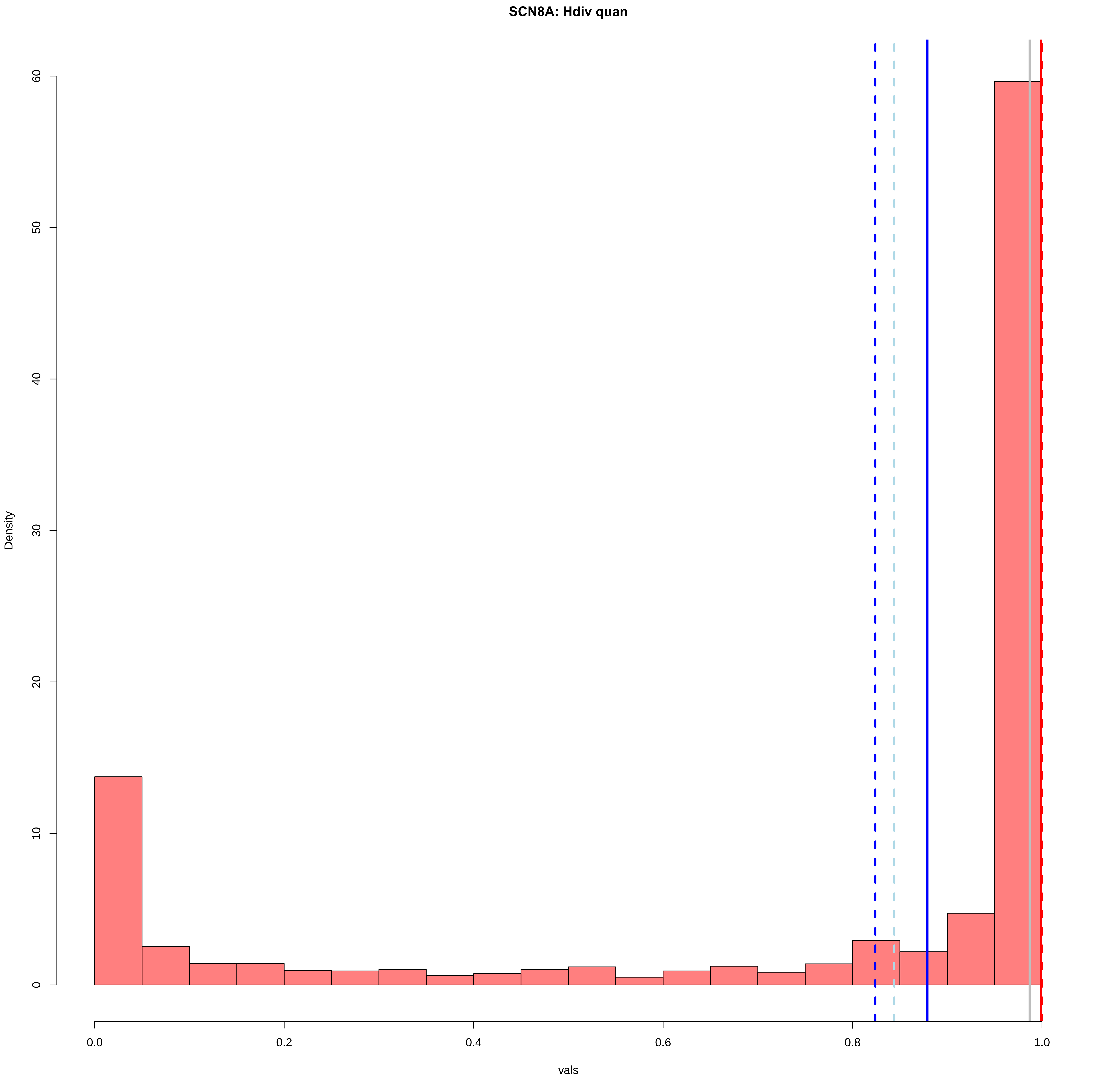

SCN8A: Hdiv quan
Density
vals
0
10
20
30
40
50
60
0.0
0.2
0.4
0.6
0.8
1.0

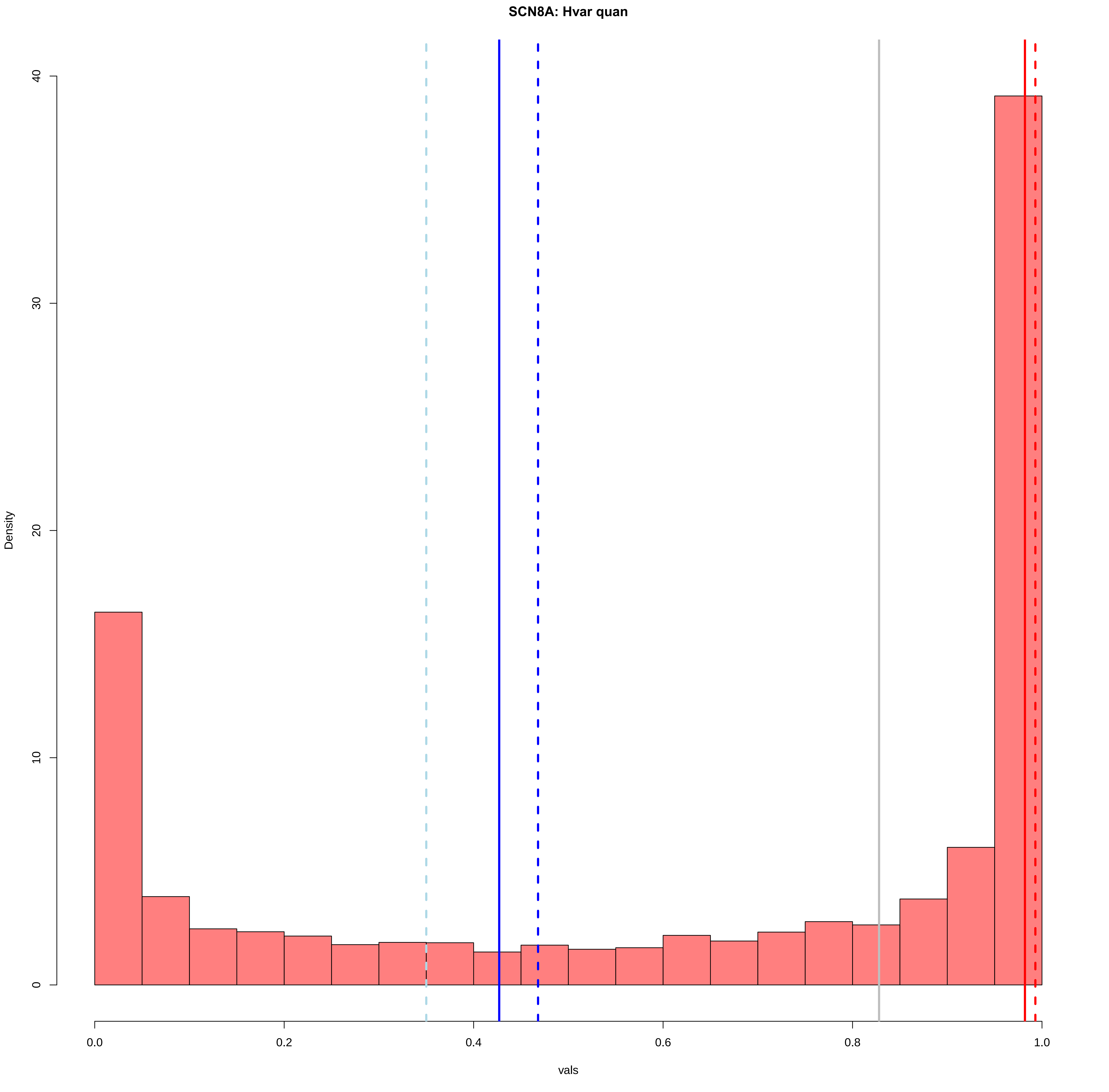
SCN8A: Hvar quan
Density
0
10
20
30
40
0.0
0.2
0.4
0.6
0.8
1.0
vals

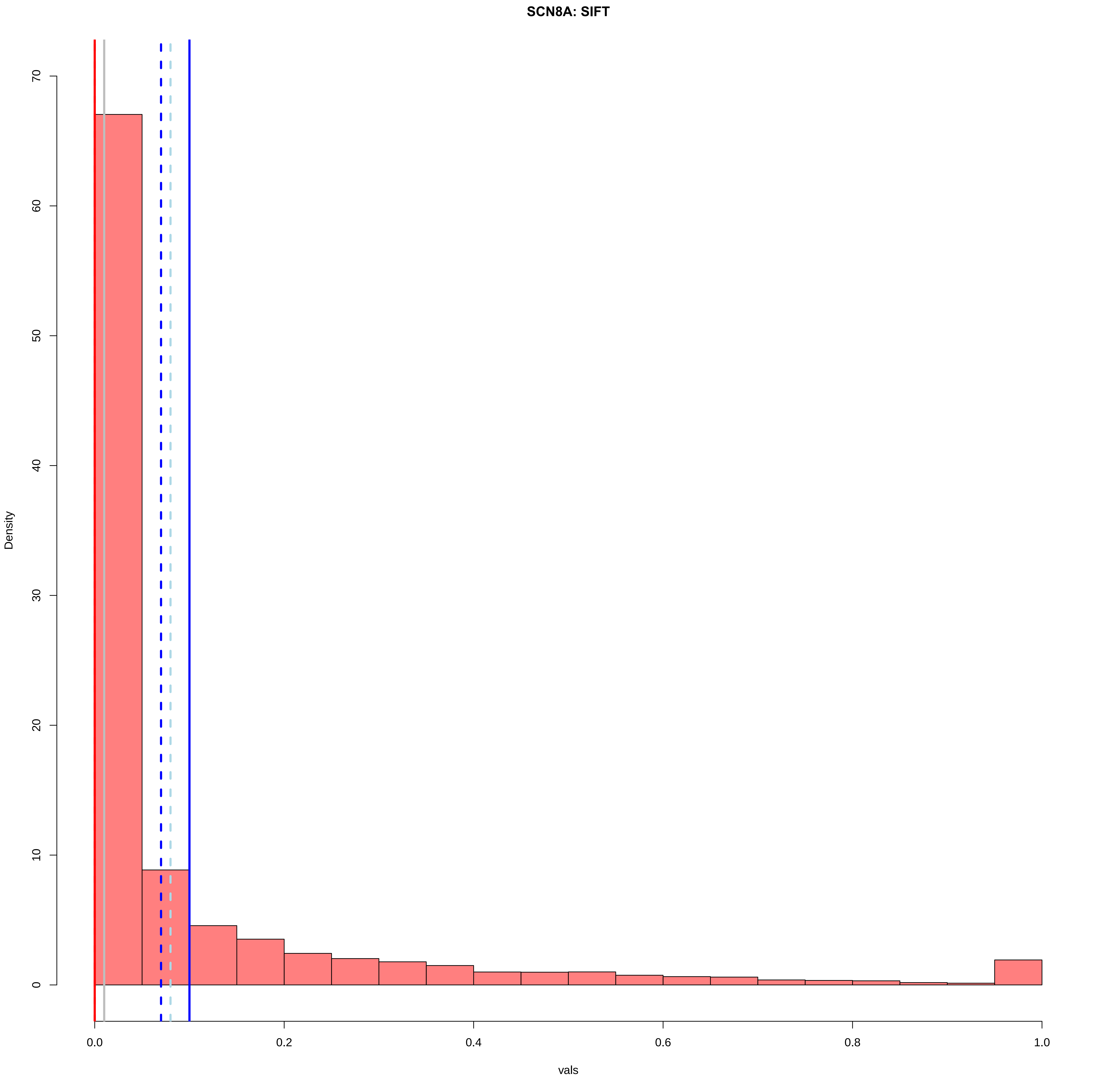

SCN8A: SIFT
Density
vals
0
10
20
30
40
50
60
70
0.0
0.2
0.4
0.6
0.8
1.0

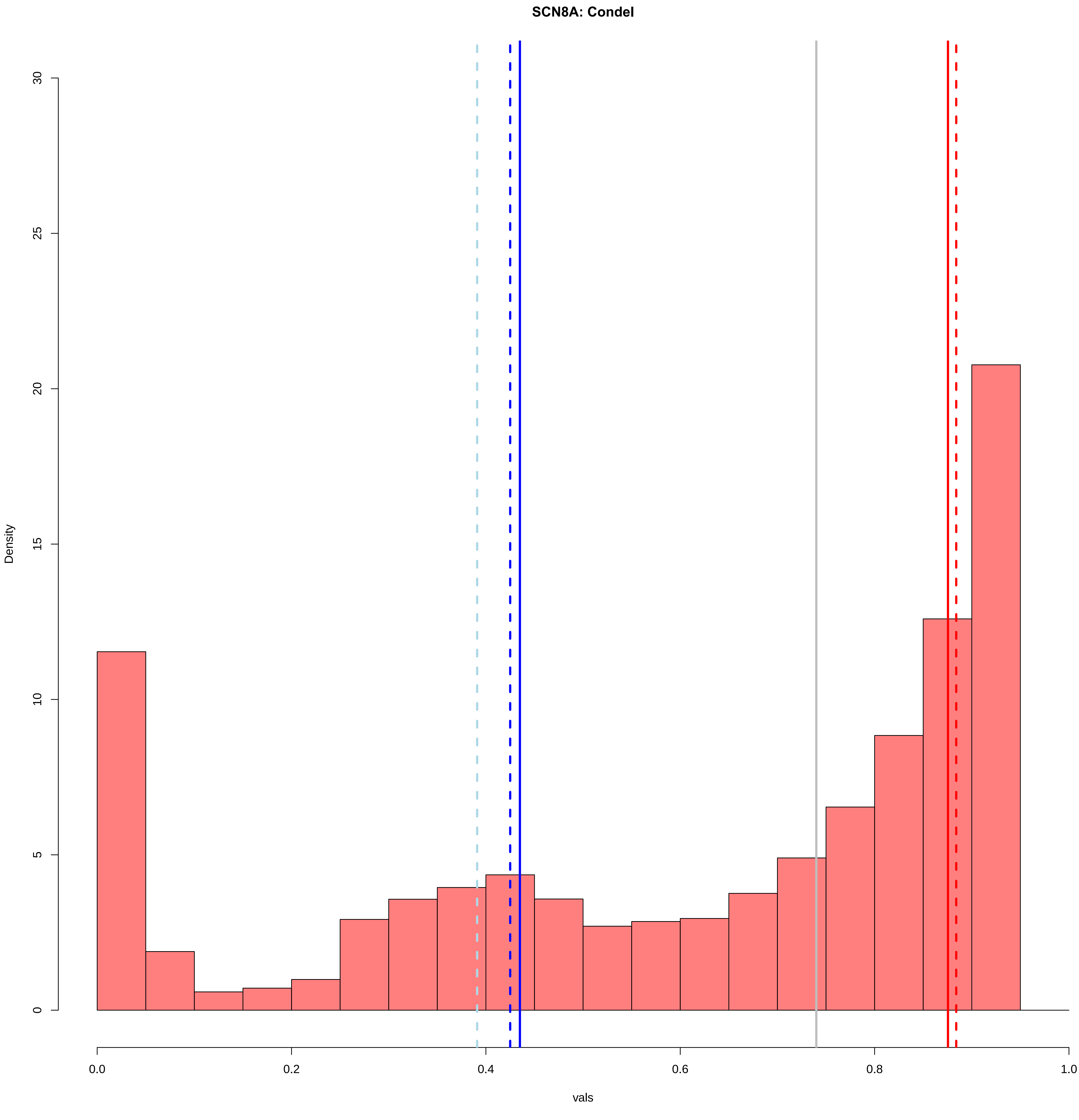

SCN8A: Condel
Density
0
5
10
15
20
25
30
0.0
0.2
0.4
0.6
0.8
1.0
vals

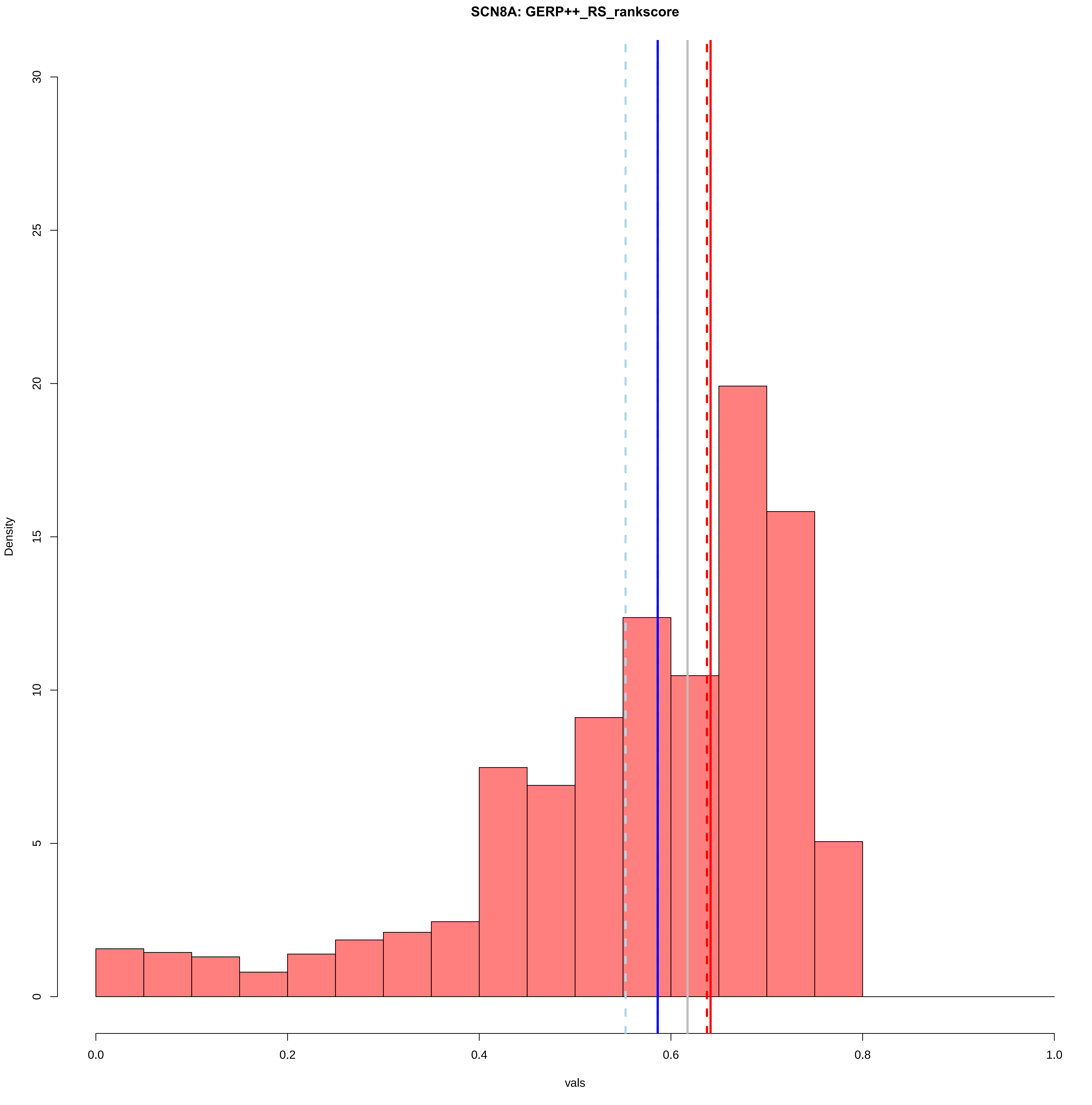
SCN8A: GERP++_RS_rankscore
Density
vals
0
5
10
15
20
25
30
0.0
0.2
0.4
0.6
0.8
1.0

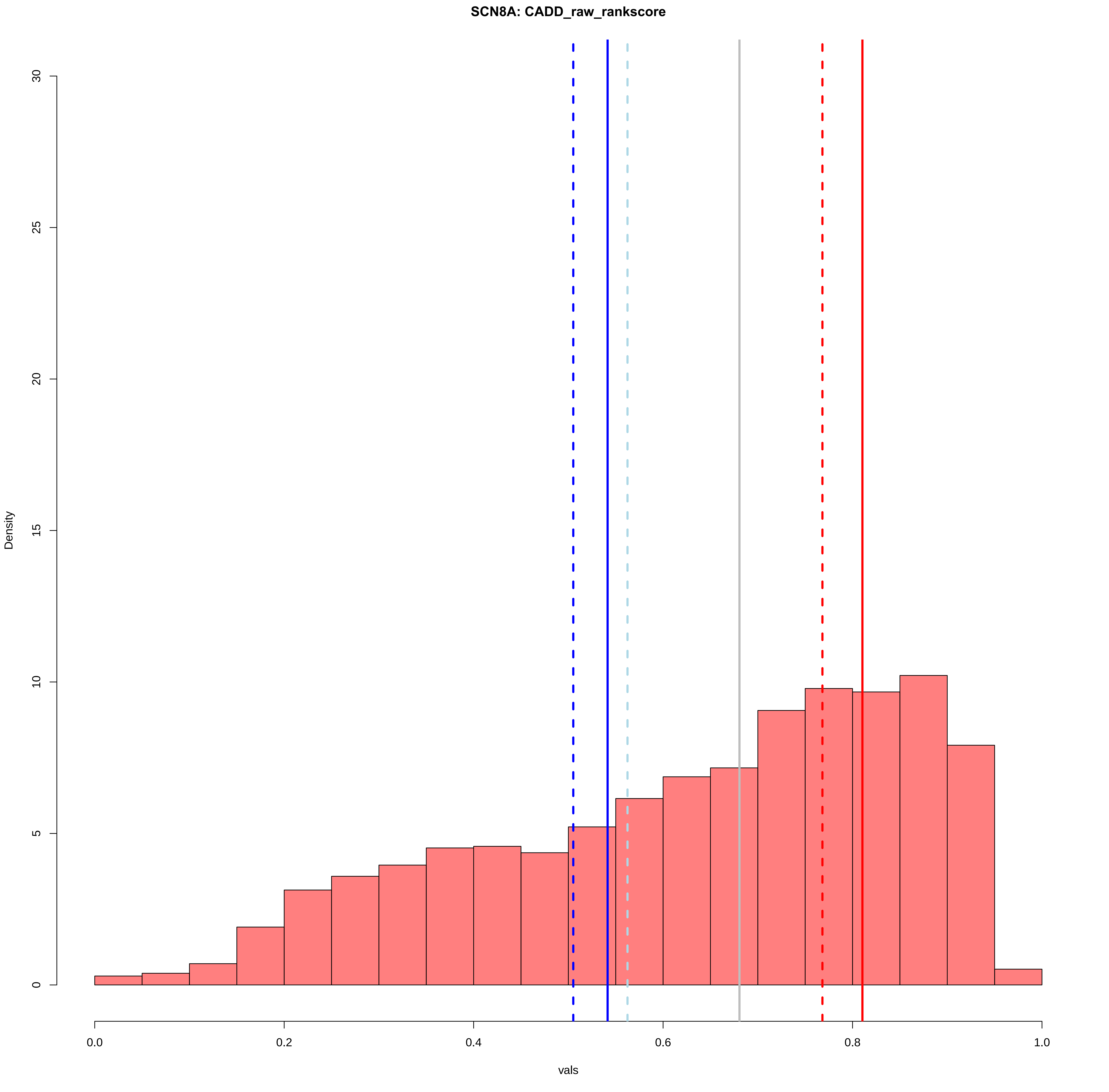

SCN8A: CADD_raw_rankscore
Density
0
5
10
15
20
25
30
0.0
0.2
0.4
0.6
0.8
1.0
vals

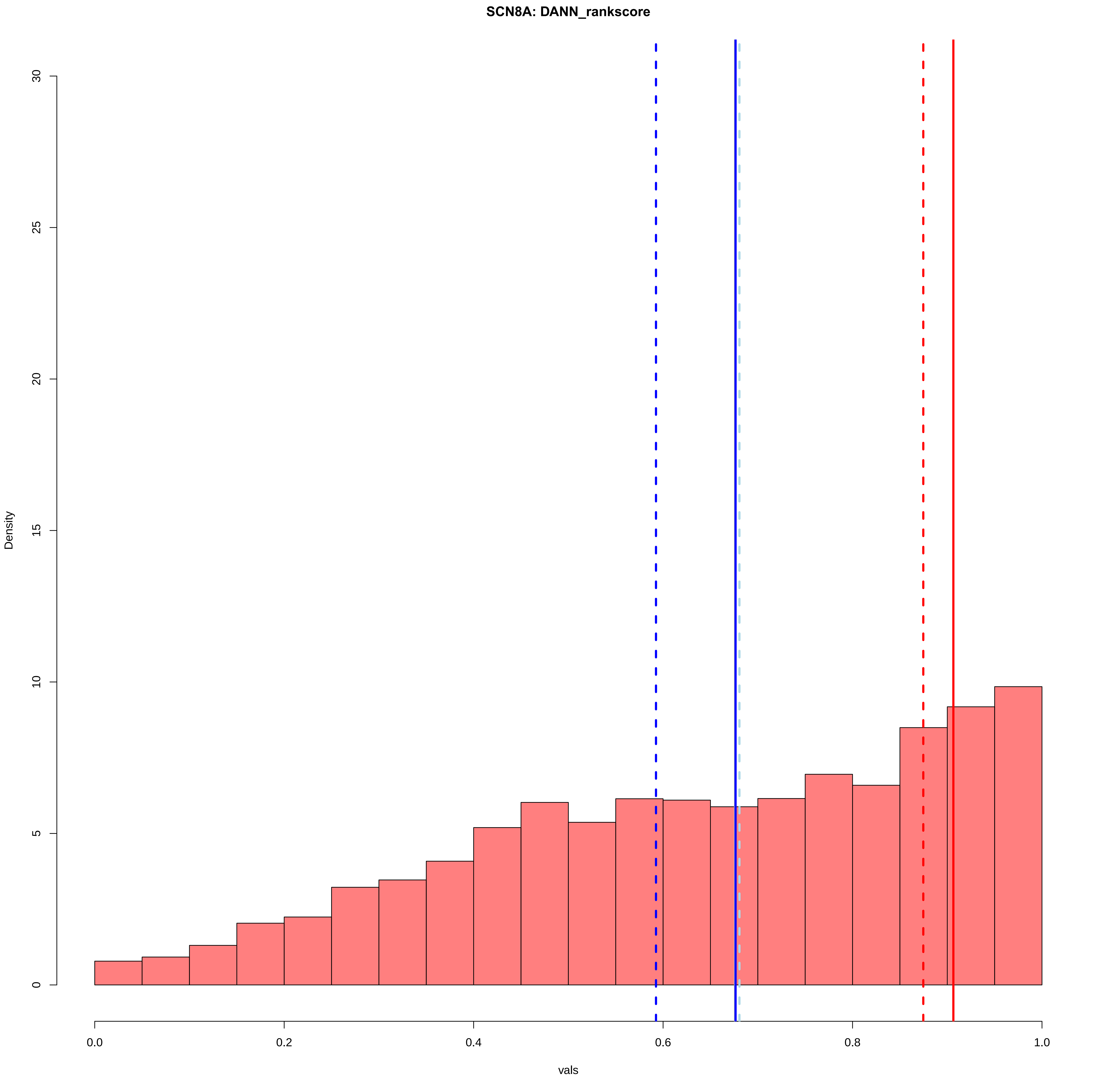
SCN8A: DANN_rankscore
Density
vals
0
5
10
15
20
25
30
0.0
0.2
0.4
0.6
0.8
1.0

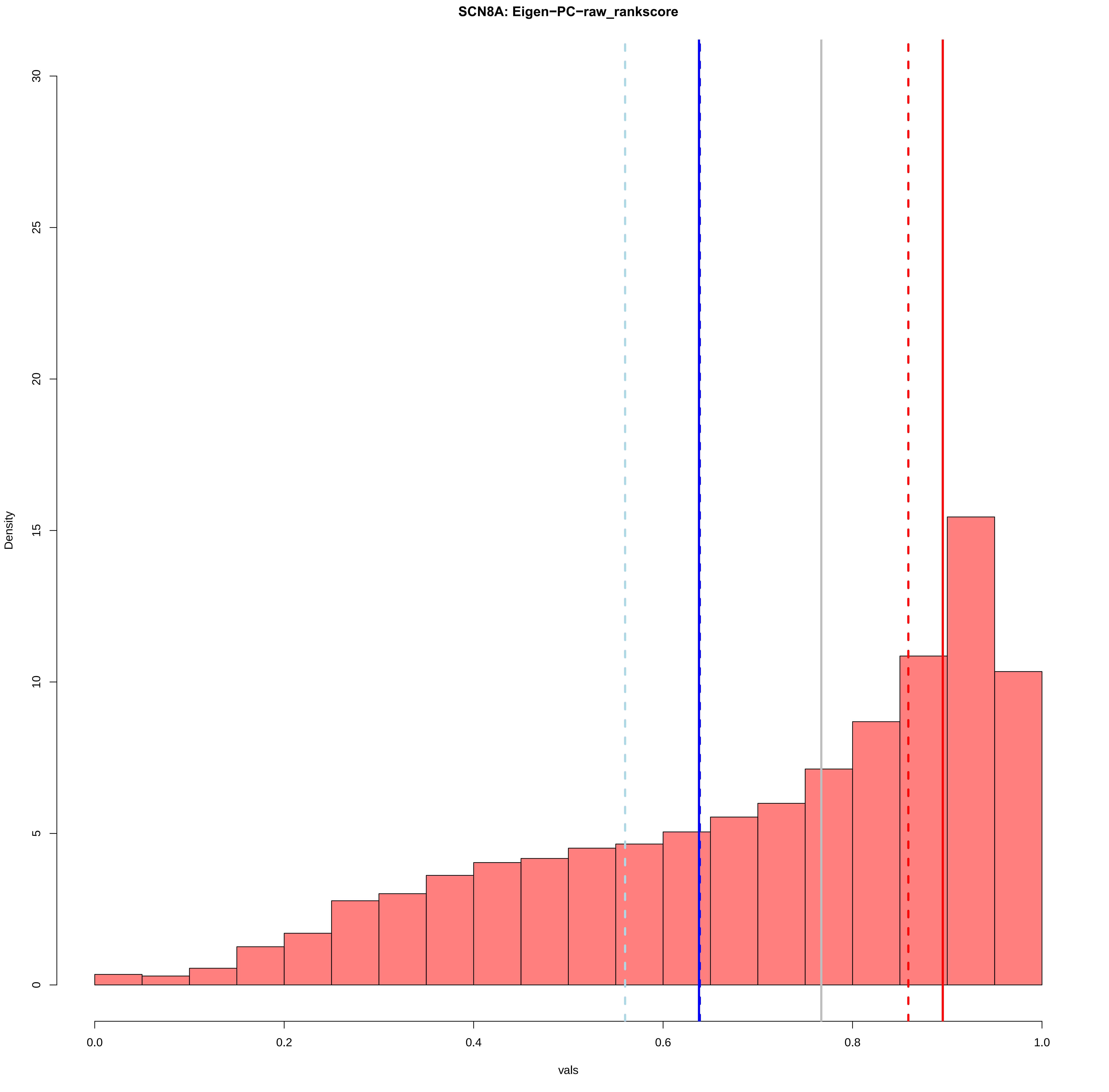
SCN8A: Eigen−PC−raw_rankscore
Density
vals
0
5
10
15
20
25
30
0.0
0.2
0.4
0.6
0.8
1.0

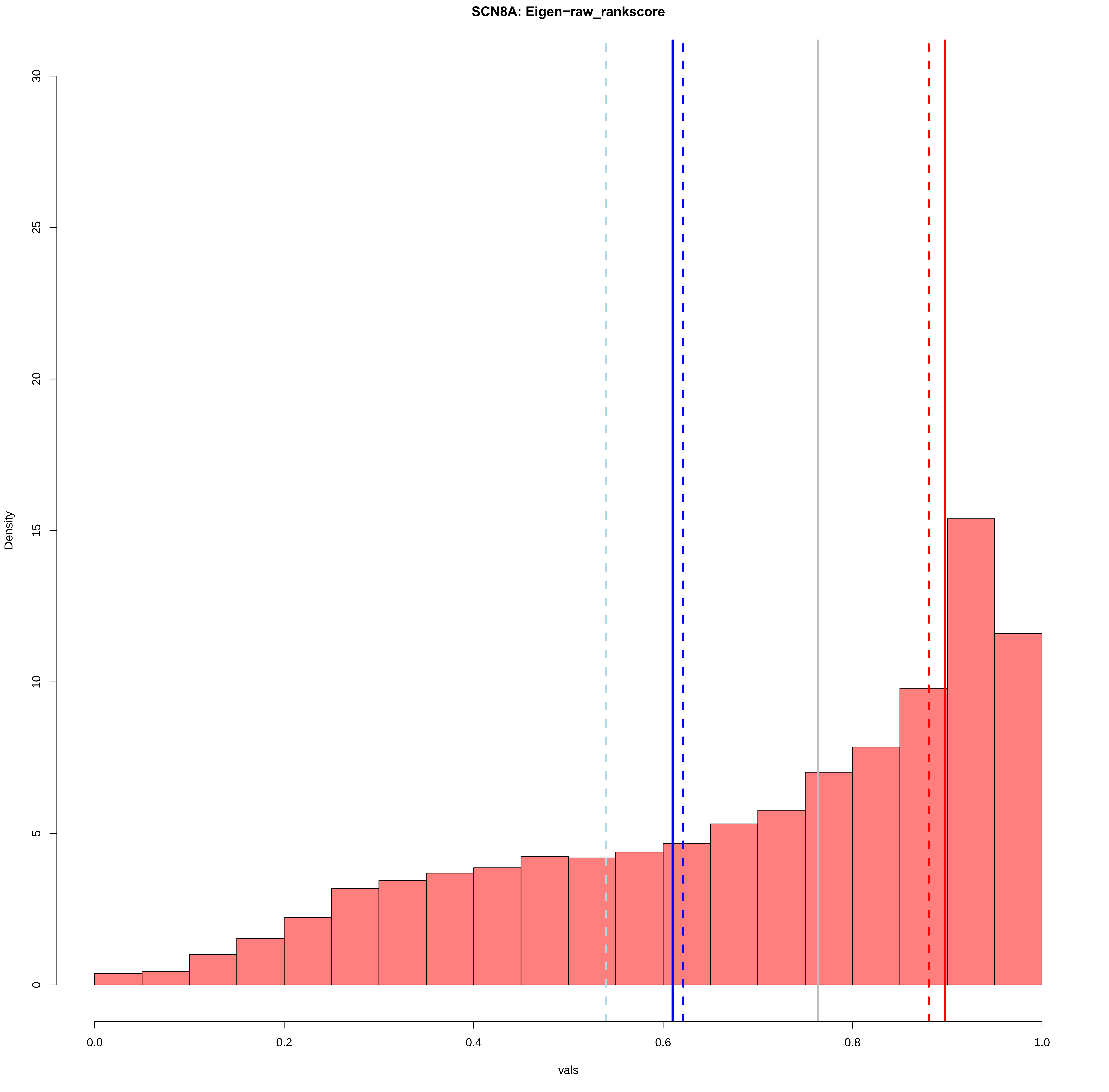
SCN8A: Eigen−raw_rankscore
Density
vals
0
5
10
15
20
25
30
0.0
0.2
0.4
0.6
0.8
1.0

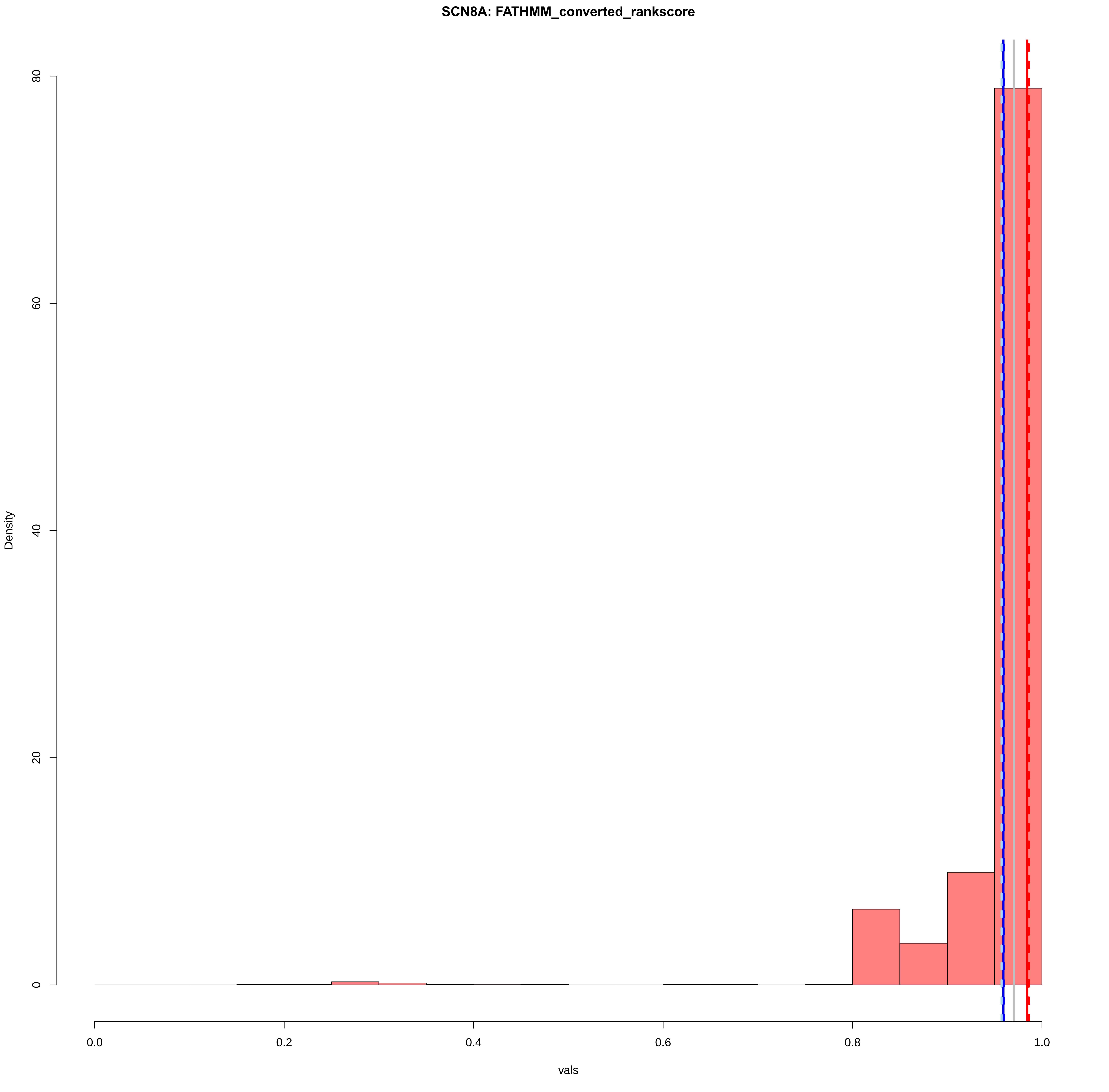
SCN8A: FATHMM_converted_rankscore
Density
vals
0
20
40
60
80
0.0
0.2
0.4
0.6
0.8
1.0

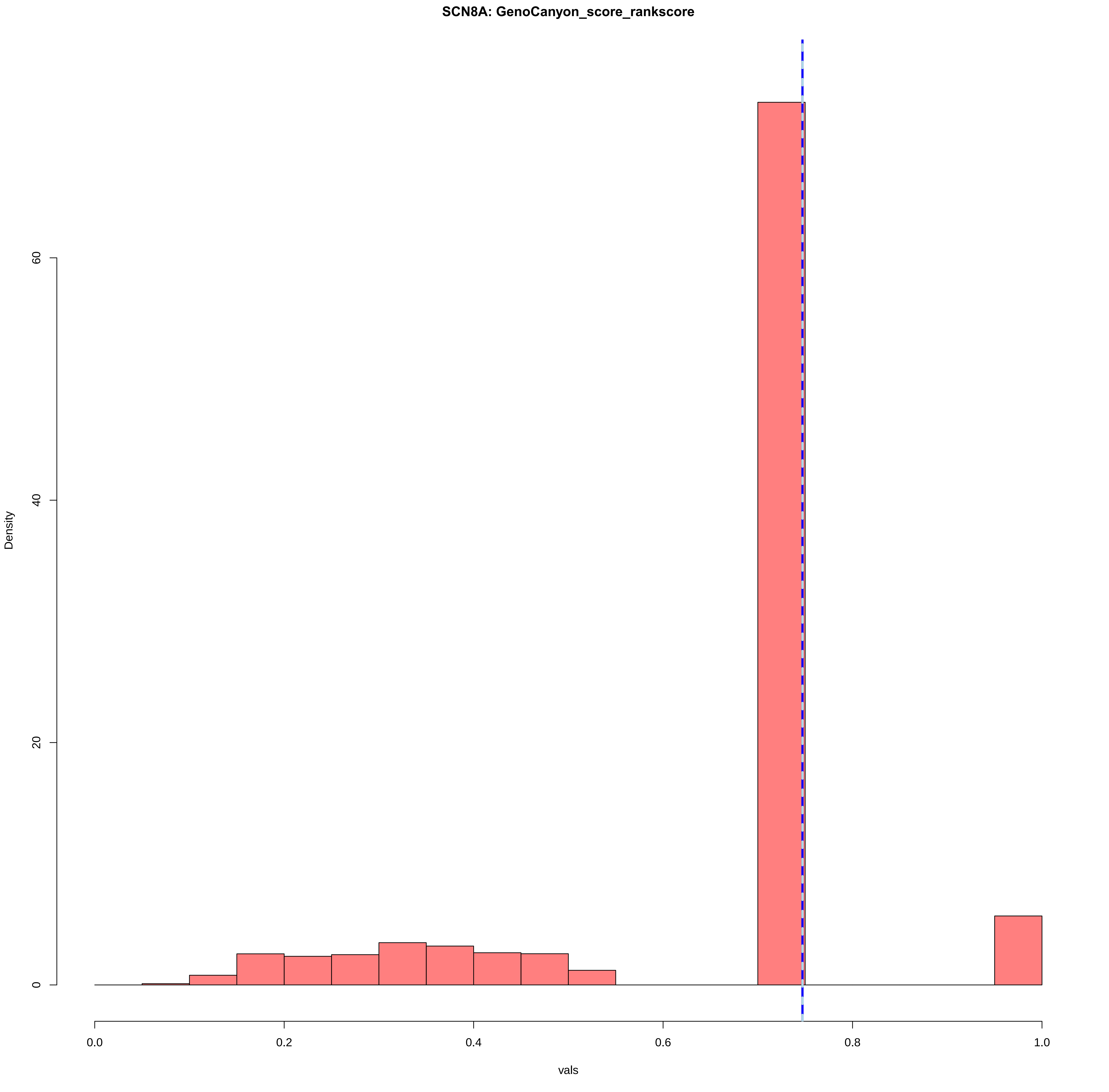
SCN8A: GenoCanyon_score_rankscore
Density
0
20
40
60
0.0
0.2
0.4
0.6
0.8
1.0
vals

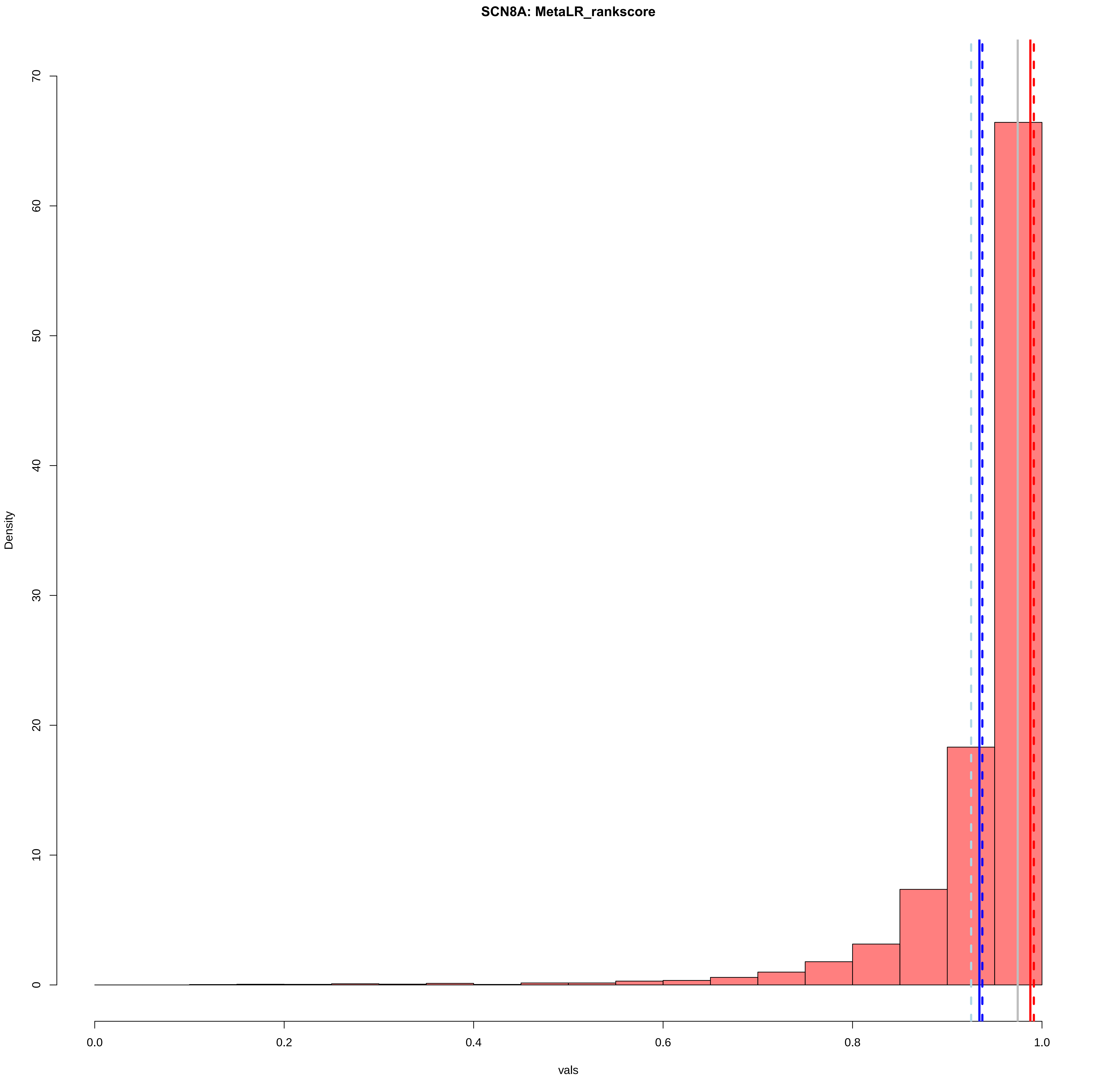

SCN8A: MetaLR_rankscore
Density
vals
0
10
20
30
40
50
60
70
0.0
0.2
0.4
0.6
0.8
1.0

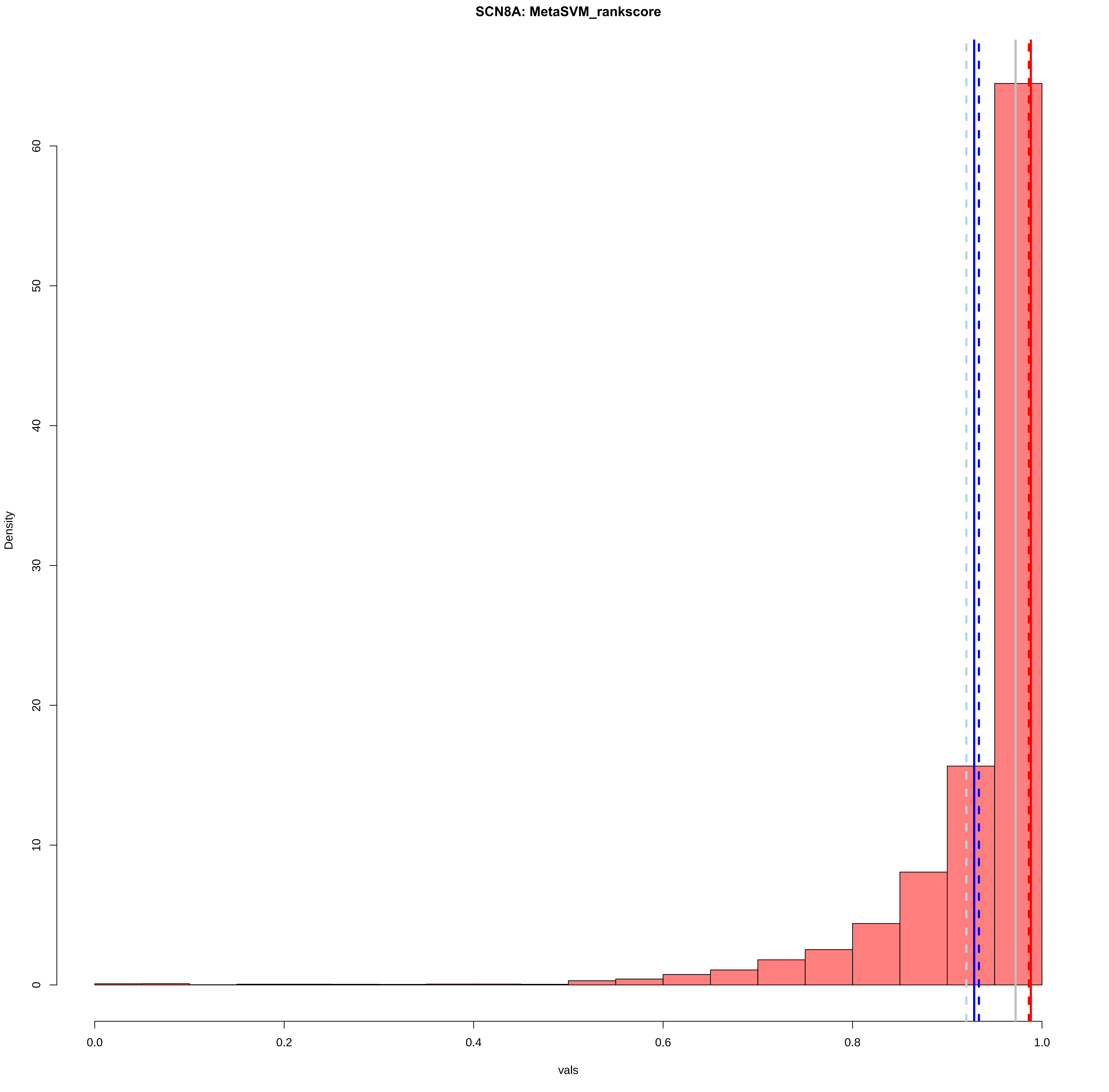
SCN8A: MetaSVM_rankscore
Density
vals
0
10
20
30
40
50
60
0.0
0.2
0.4
0.6
0.8
1.0

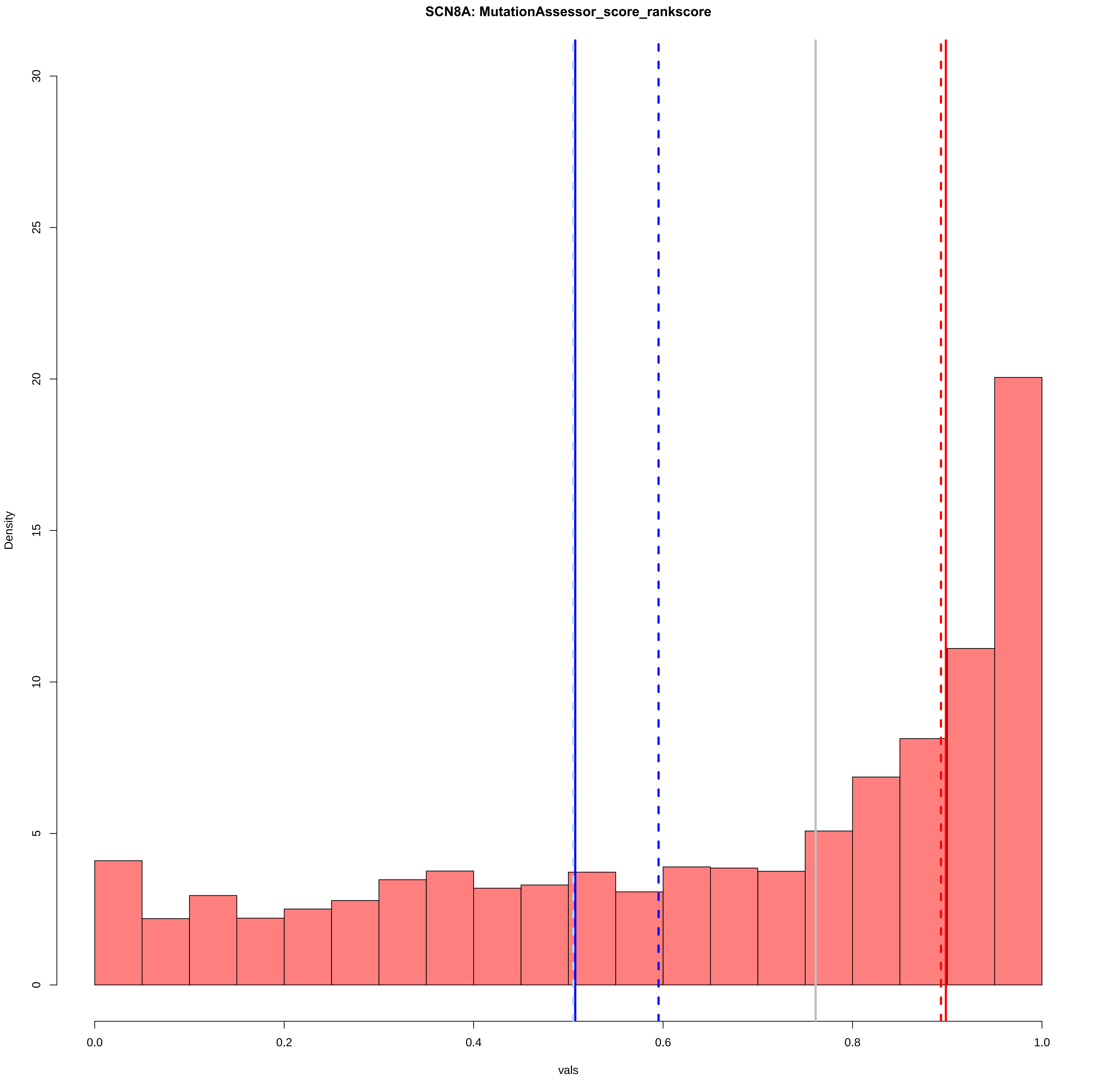
SCN8A: MutationAssessor_score_rankscore
Density
vals
0
5
10
15
20
25
30
0.0
0.2
0.4
0.6
0.8
1.0

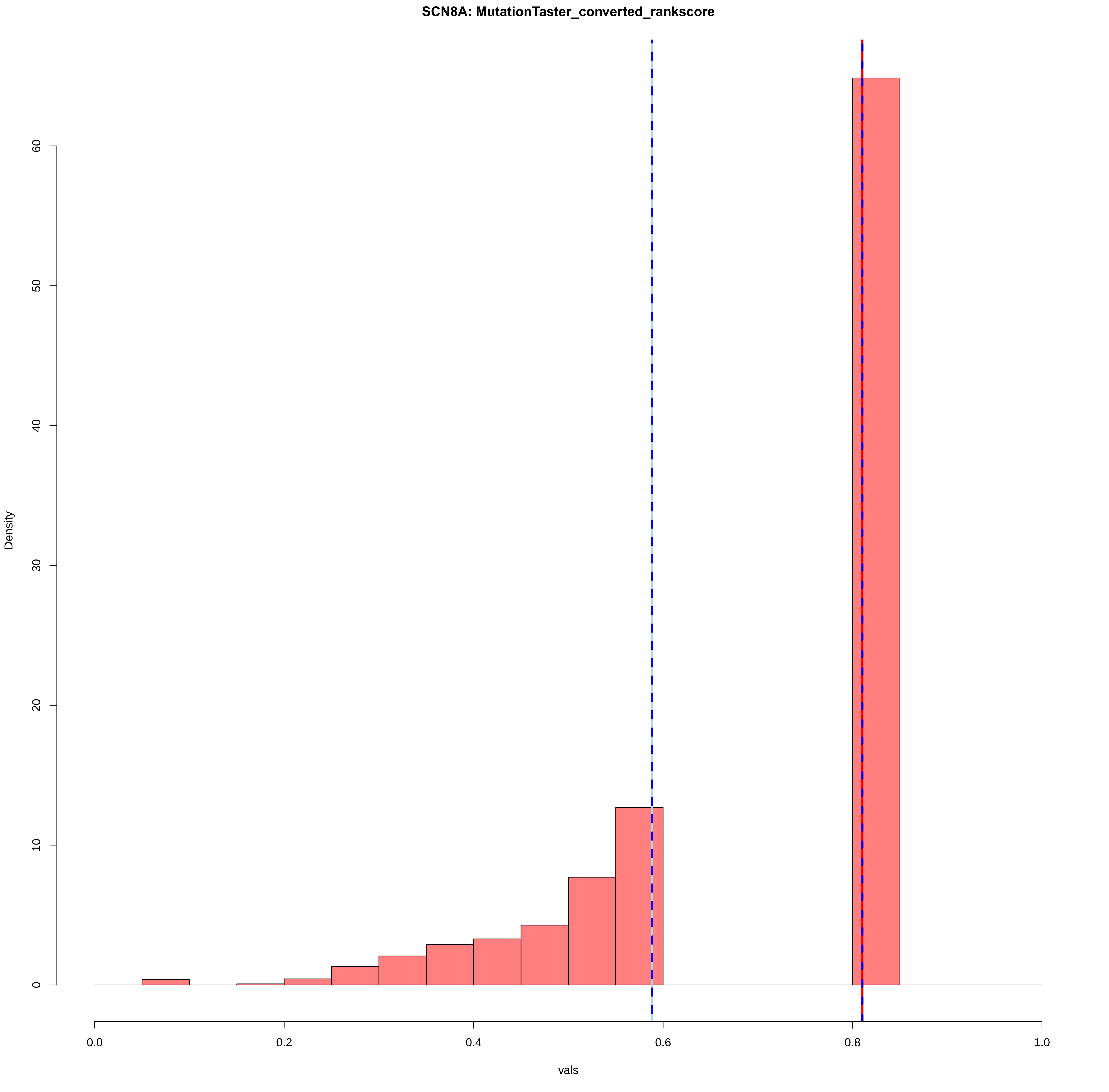

SCN8A: MutationTaster_converted_rankscore
Density
vals
0
10
20
30
40
50
60
0.0
0.2
0.4
0.6
0.8
1.0

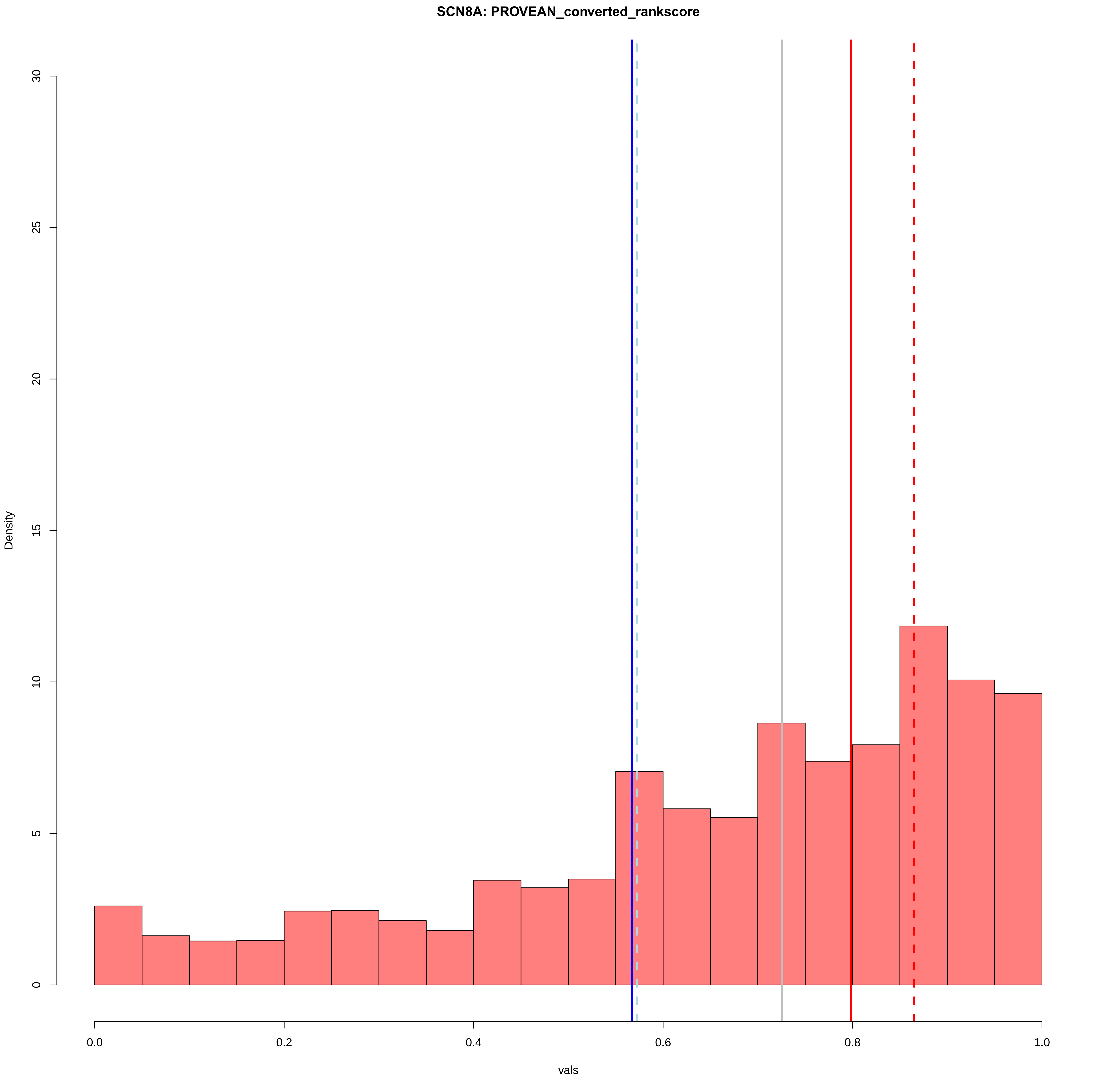

SCN8A: PROVEAN_converted_rankscore
Density
vals
0
5
10
15
20
25
30
0.0
0.2
0.4
0.6
0.8
1.0

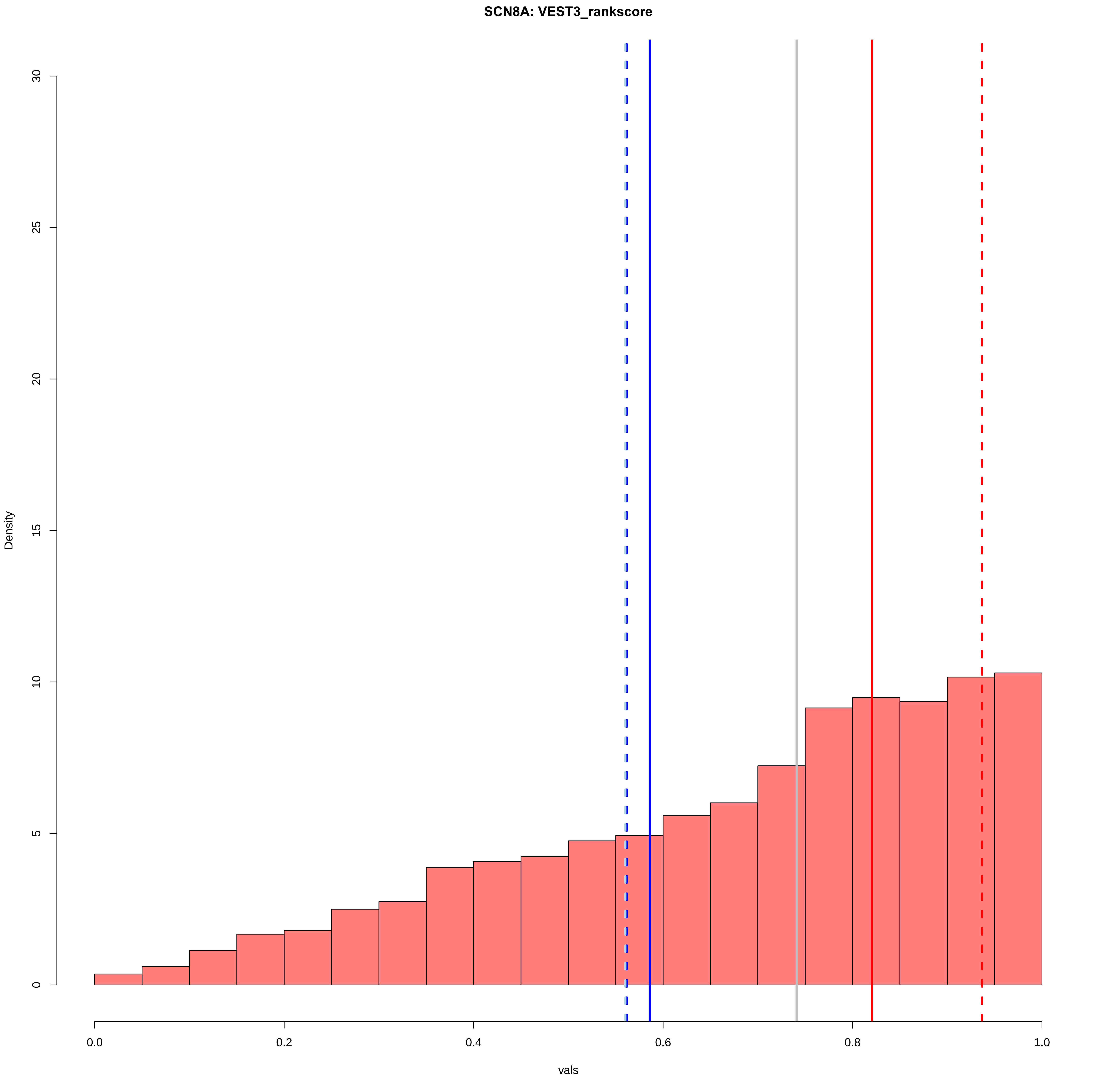

SCN8A: VEST3_rankscore
Density
vals
0
5
10
15
20
25
30
0.0
0.2
0.4
0.6
0.8
1.0

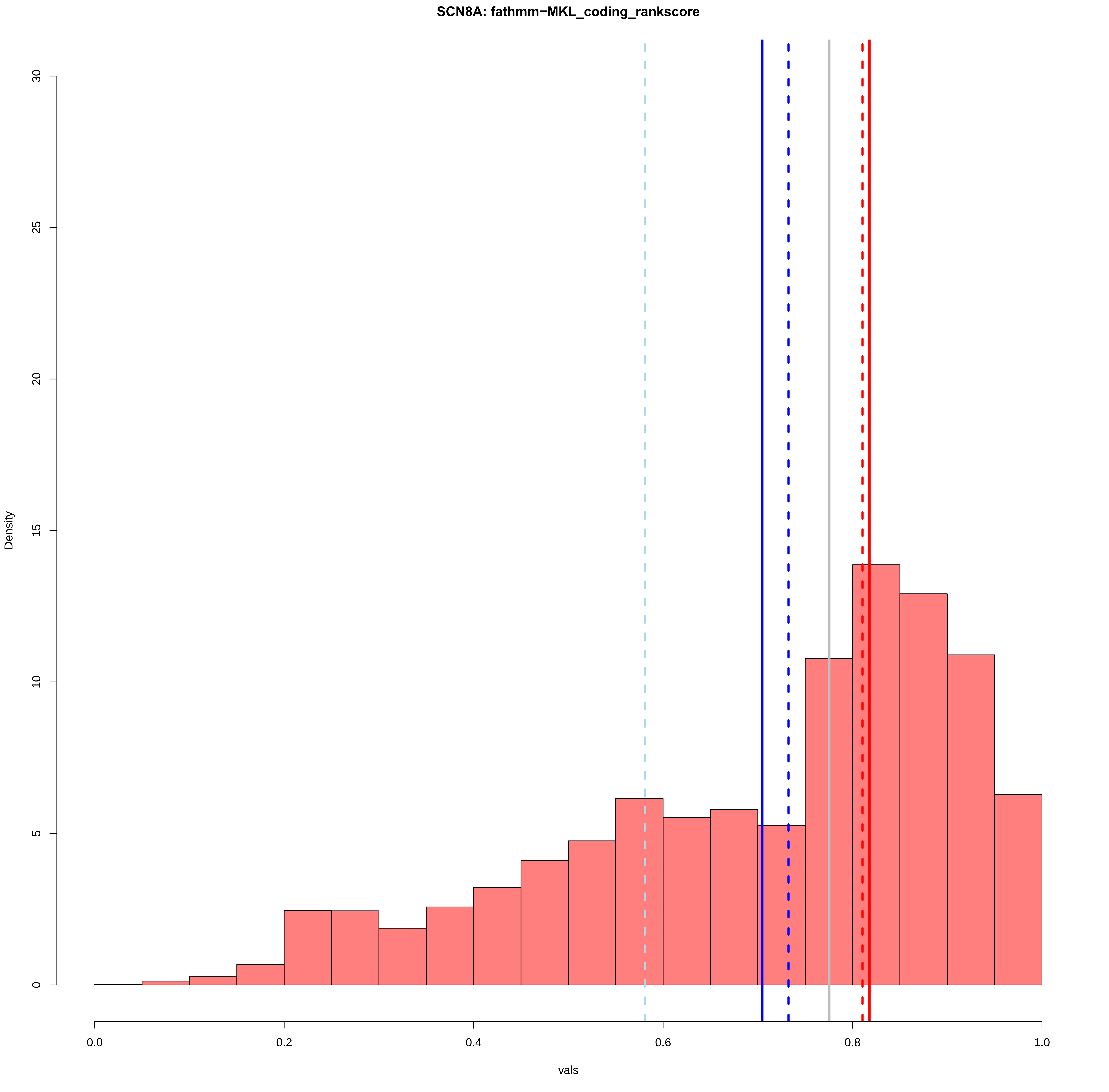
SCN8A: fathmm−MKL_coding_rankscore
Density
vals
0
5
10
15
20
25
30
0.0
0.2
0.4
0.6
0.8
1.0

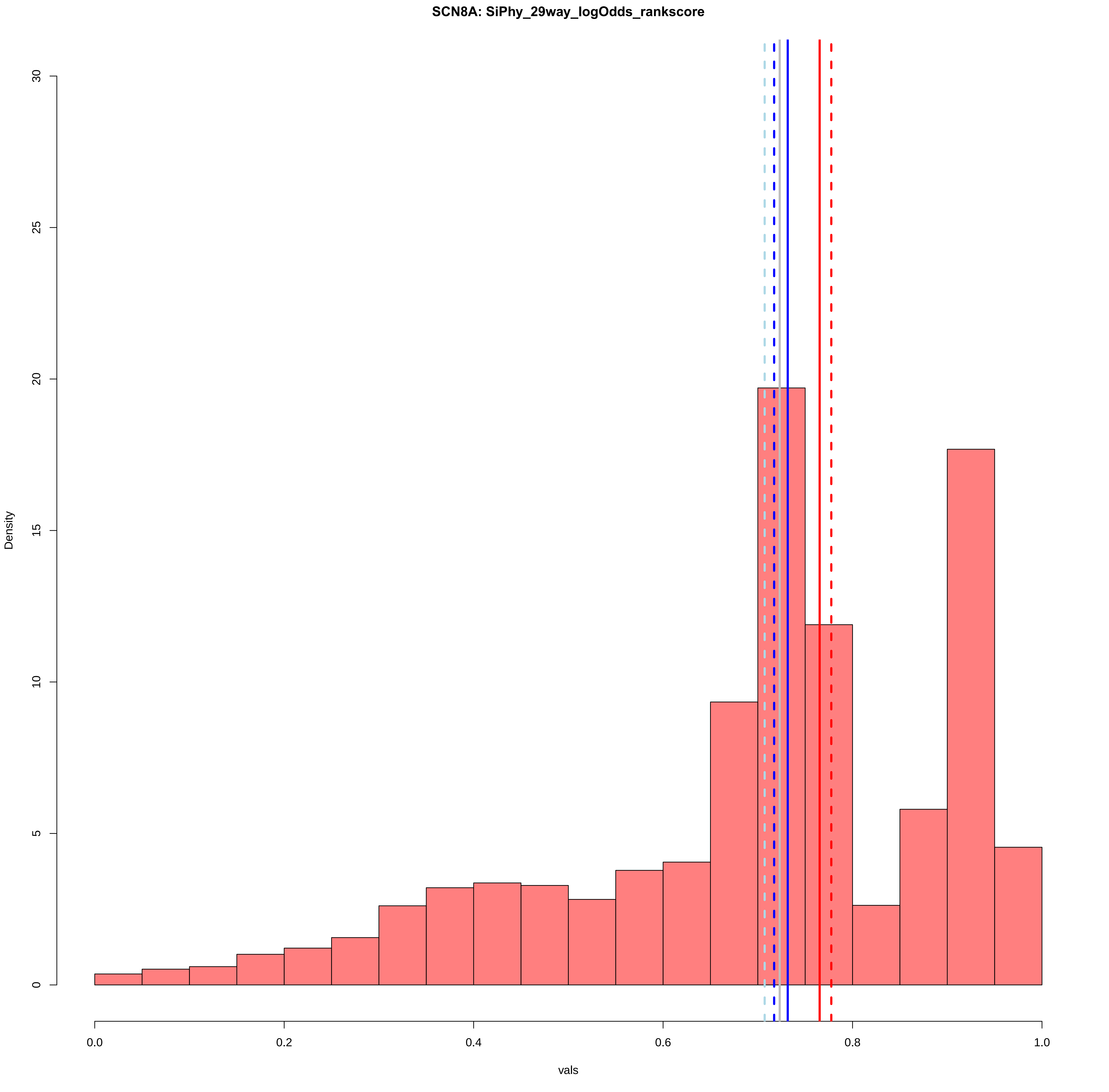

SCN8A: SiPhy_29way_logOdds_rankscore
Density
vals
0
5
10
15
20
25
30
0.0
0.2
0.4
0.6
0.8
1.0

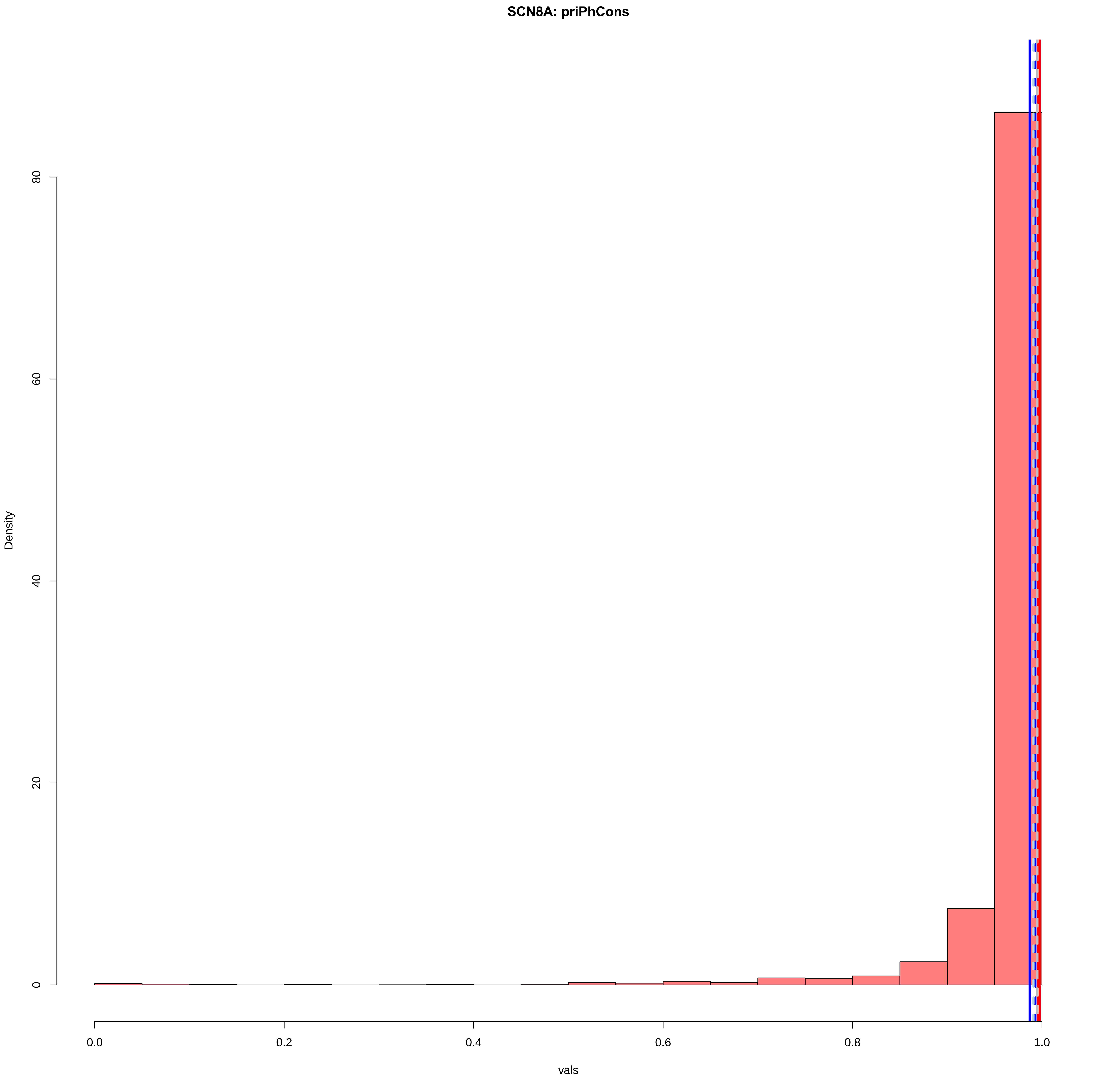
SCN8A: priPhCons
Density
vals
0
20
40
60
80
0.0
0.2
0.4
0.6
0.8
1.0

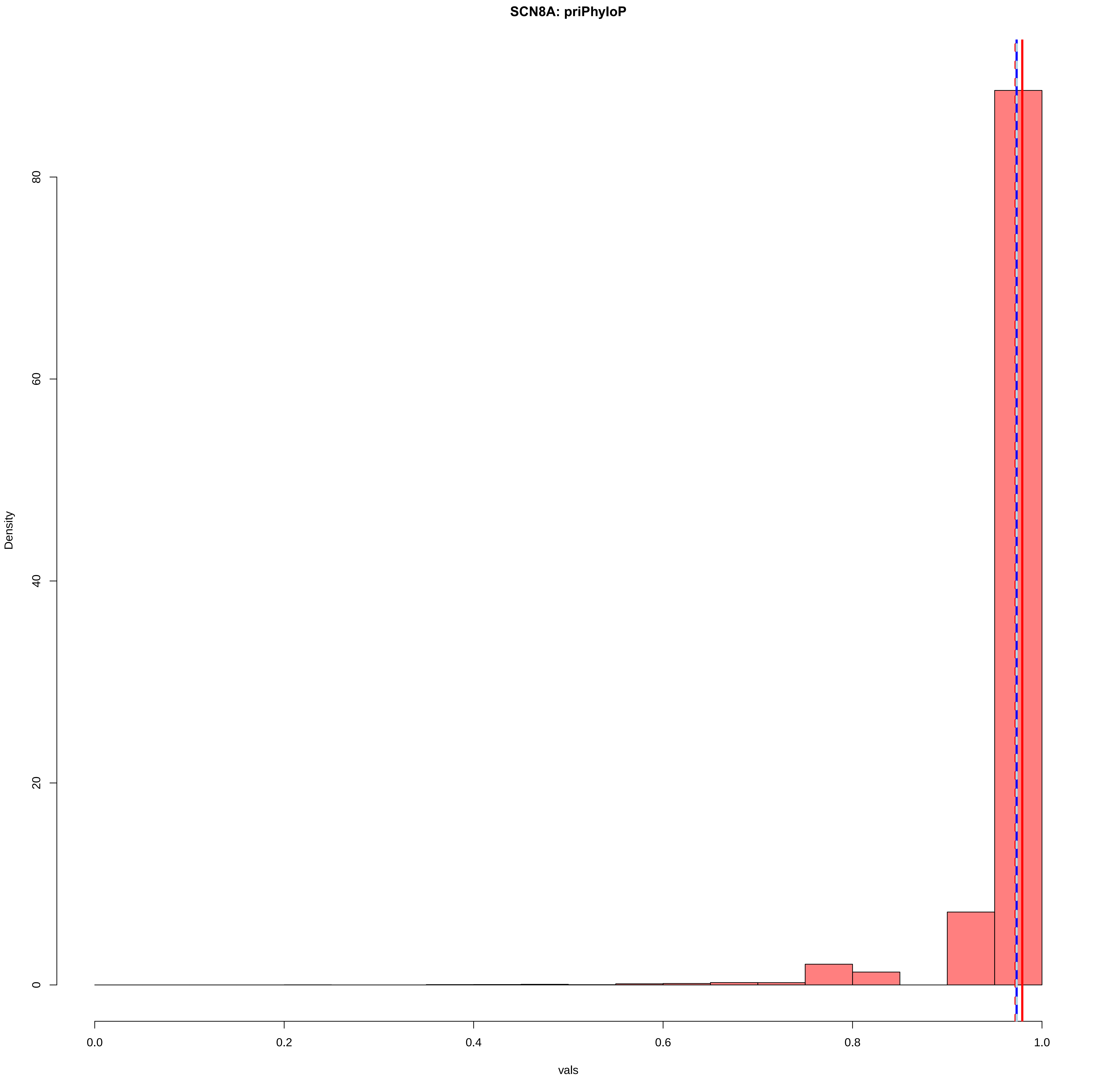

SCN8A: priPhyloP
Density
0
20
40
60
80
0.0
0.2
0.4
0.6
0.8
1.0
vals

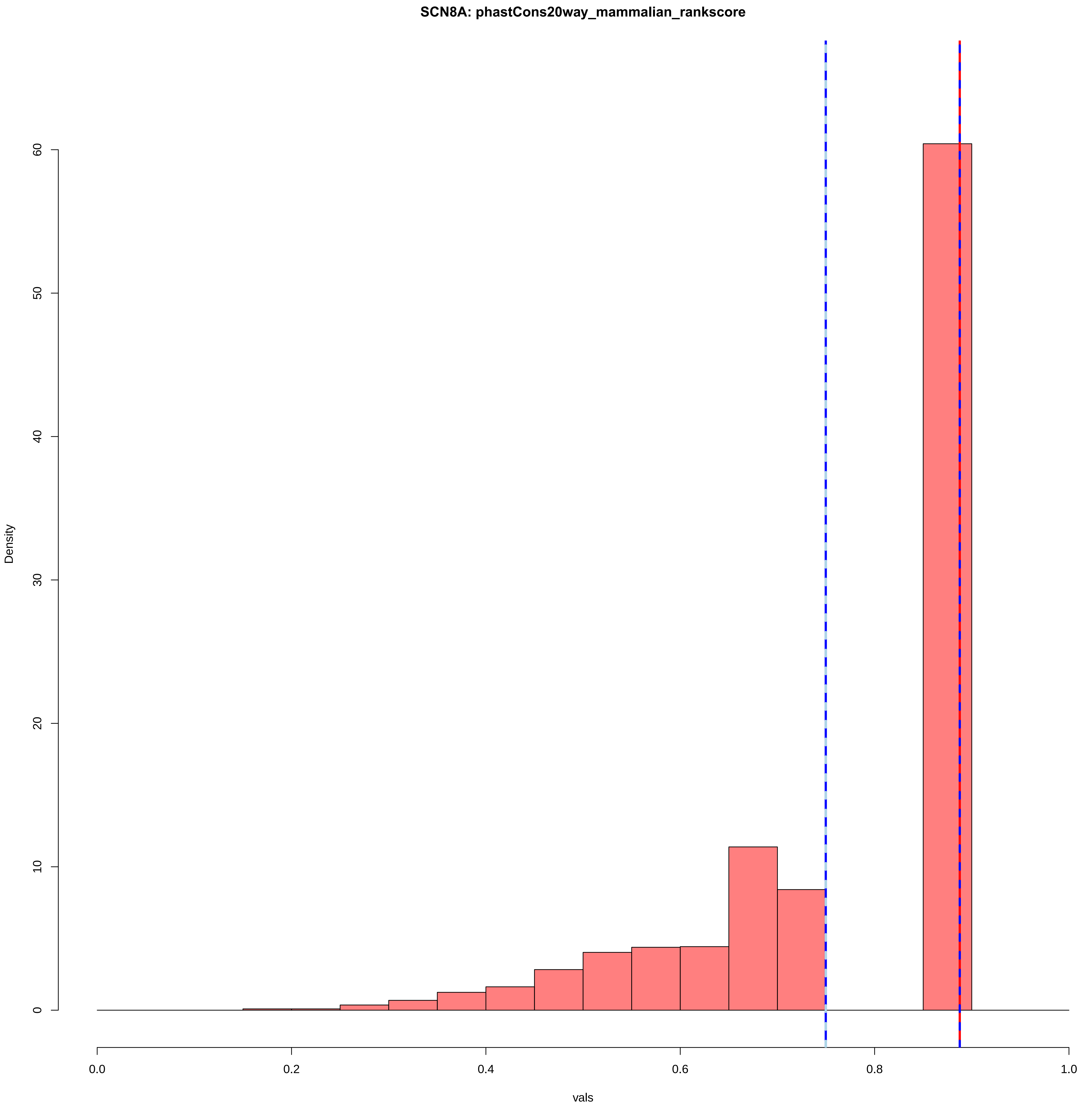
SCN8A: phastCons20way_mammalian_rankscore
Density
0
10
20
30
40
50
60
vals
0.0
0.2
0.4
0.6
0.8
1.0

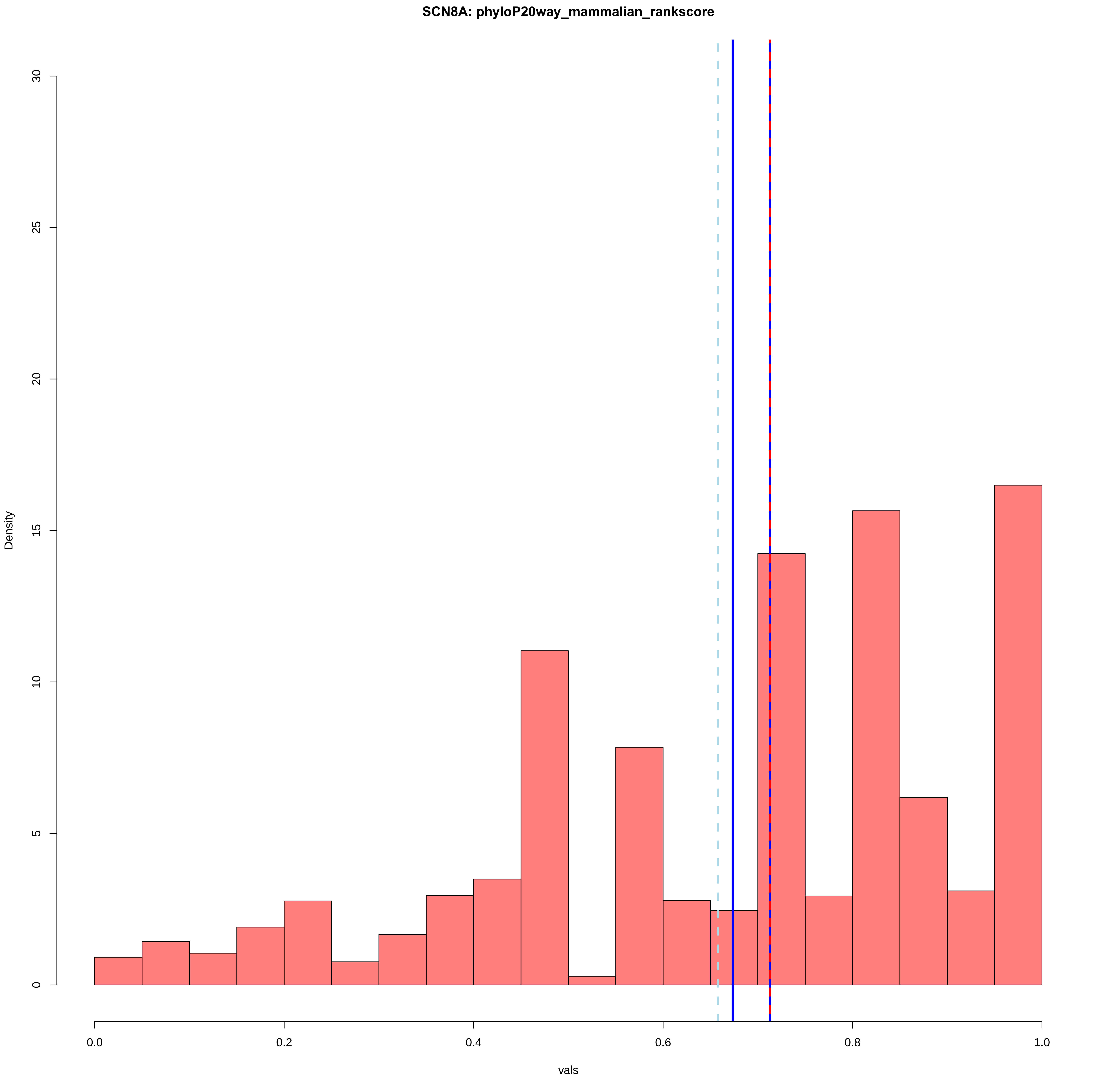

SCN8A: phyloP20way_mammalian_rankscore
Density
vals
0
5
10
15
20
25
30
0.0
0.2
0.4
0.6
0.8
1.0

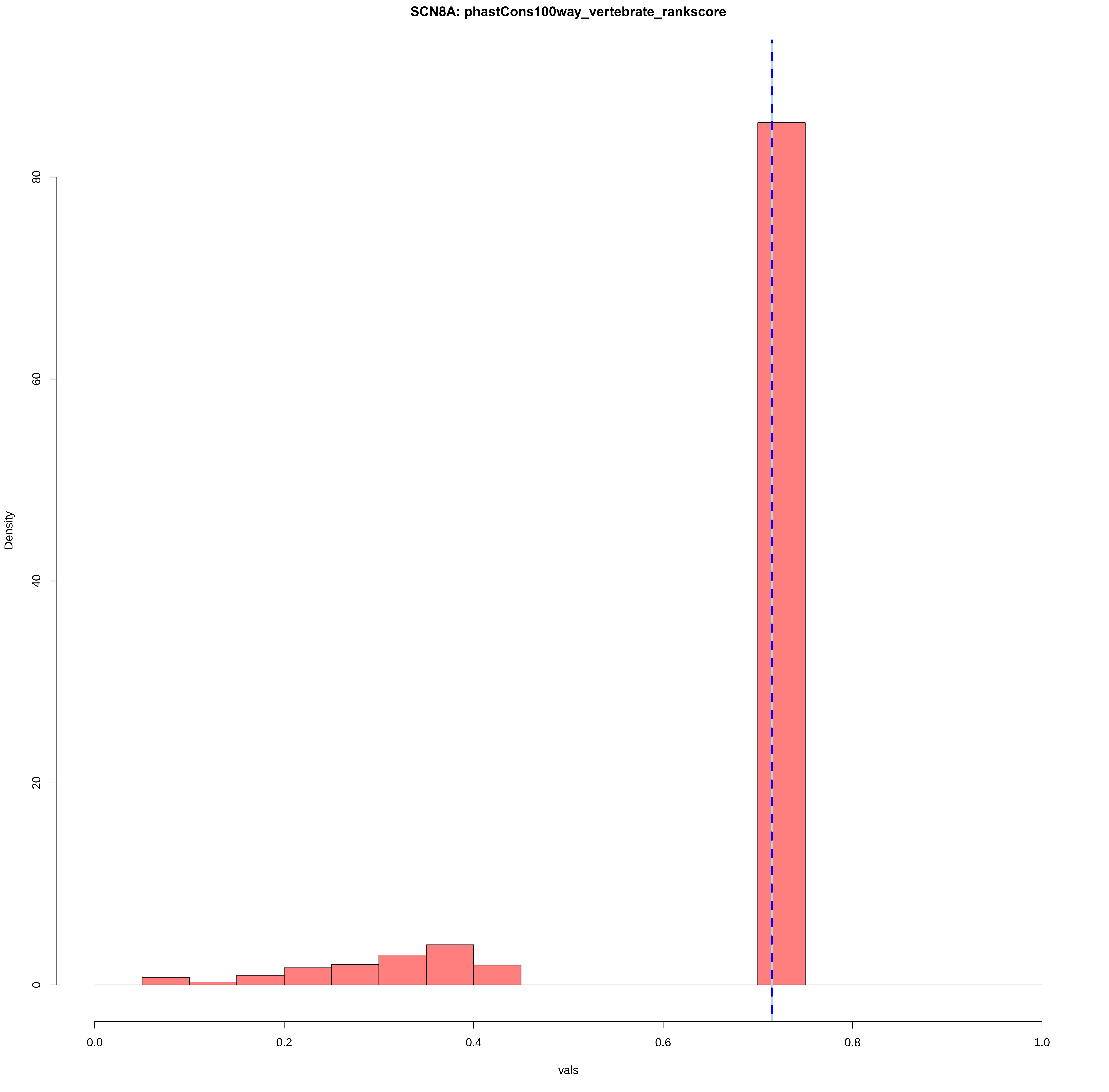

SCN8A: phastCons100way_vertebrate_rankscore
Density
0
20
40
60
80
0.0
0.2
0.4
0.6
0.8
1.0
vals

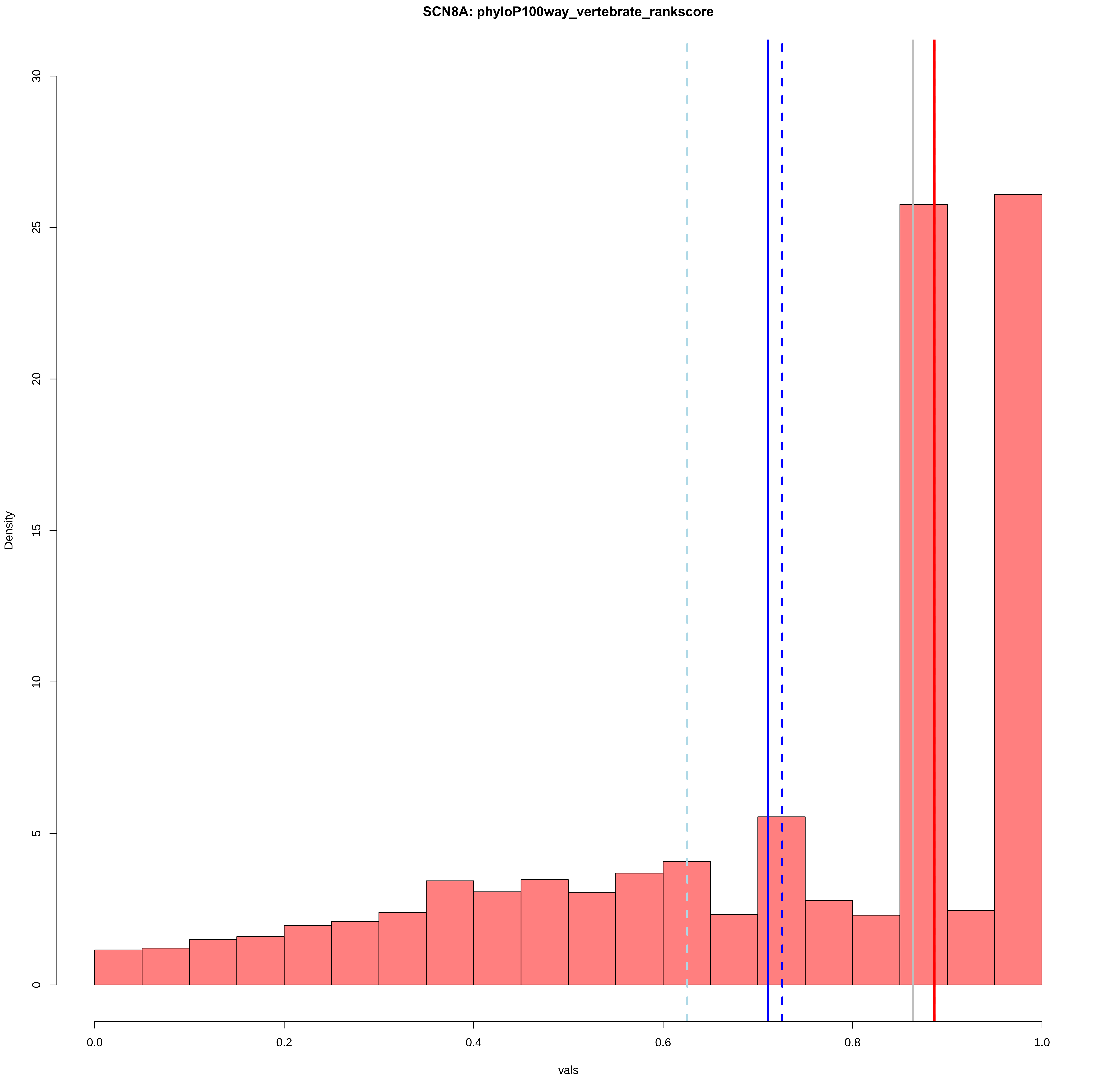

SCN8A: phyloP100way_vertebrate_rankscore
Density
vals
0
5
10
15
20
25
30
0.0
0.2
0.4
0.6
0.8
1.0

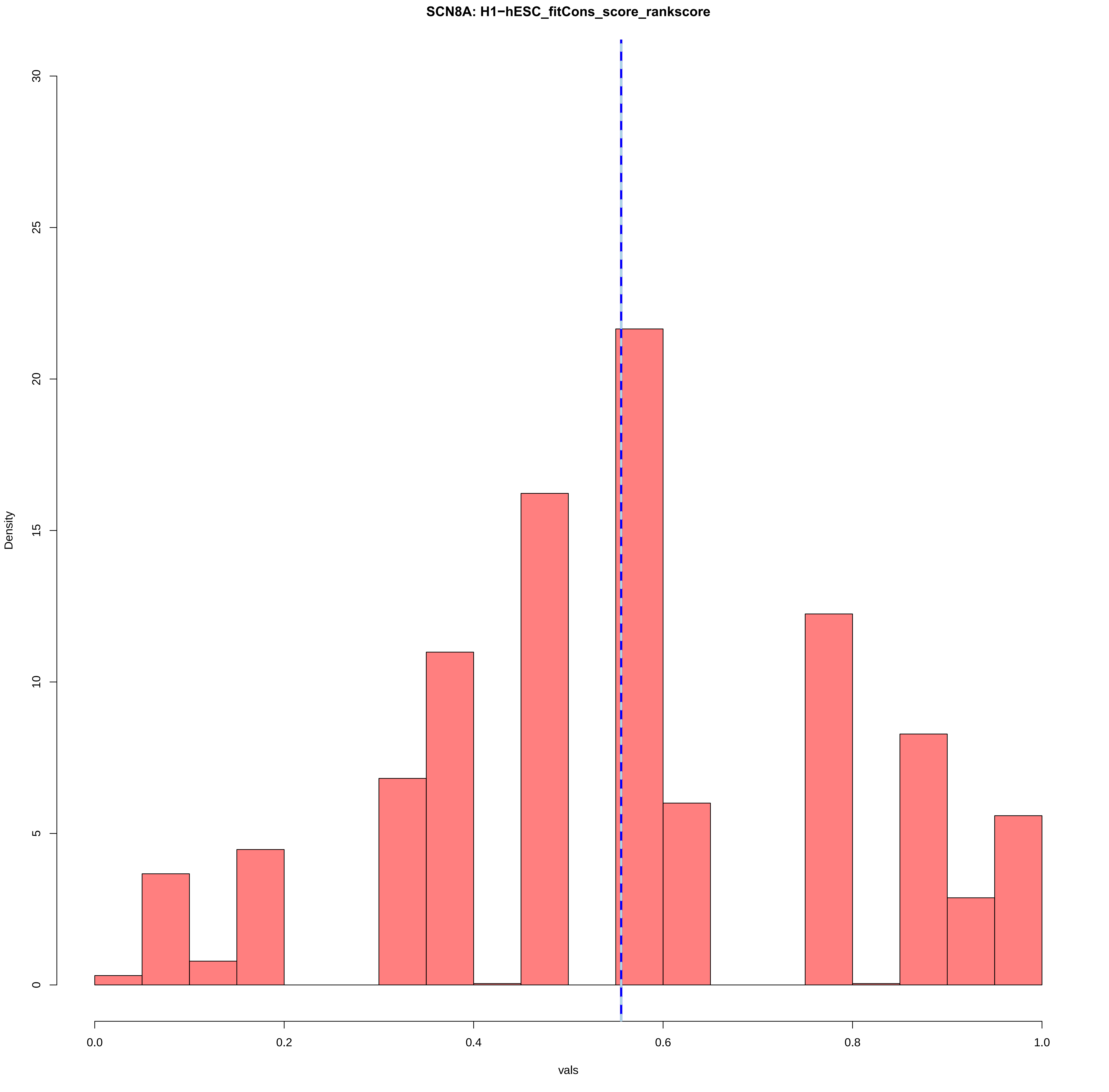
SCN8A: H1−hESC_fitCons_score_rankscore
Density
0
5
10
15
20
25
30
0.0
0.2
0.4
0.6
0.8
1.0
vals

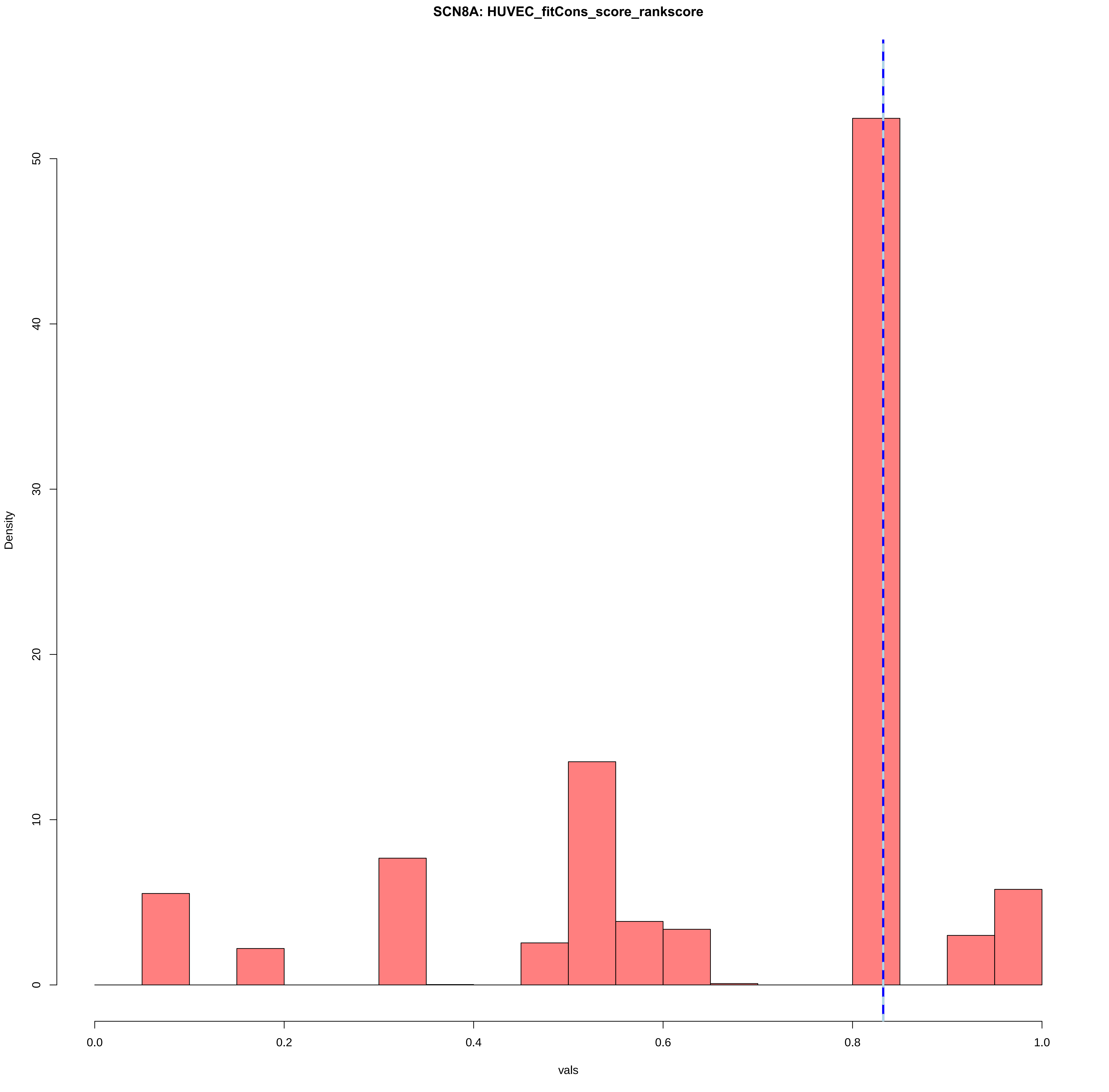
SCN8A: HUVEC_fitCons_score_rankscore
Density
vals
0
10
20
30
40
50
0.0
0.2
0.4
0.6
0.8
1.0

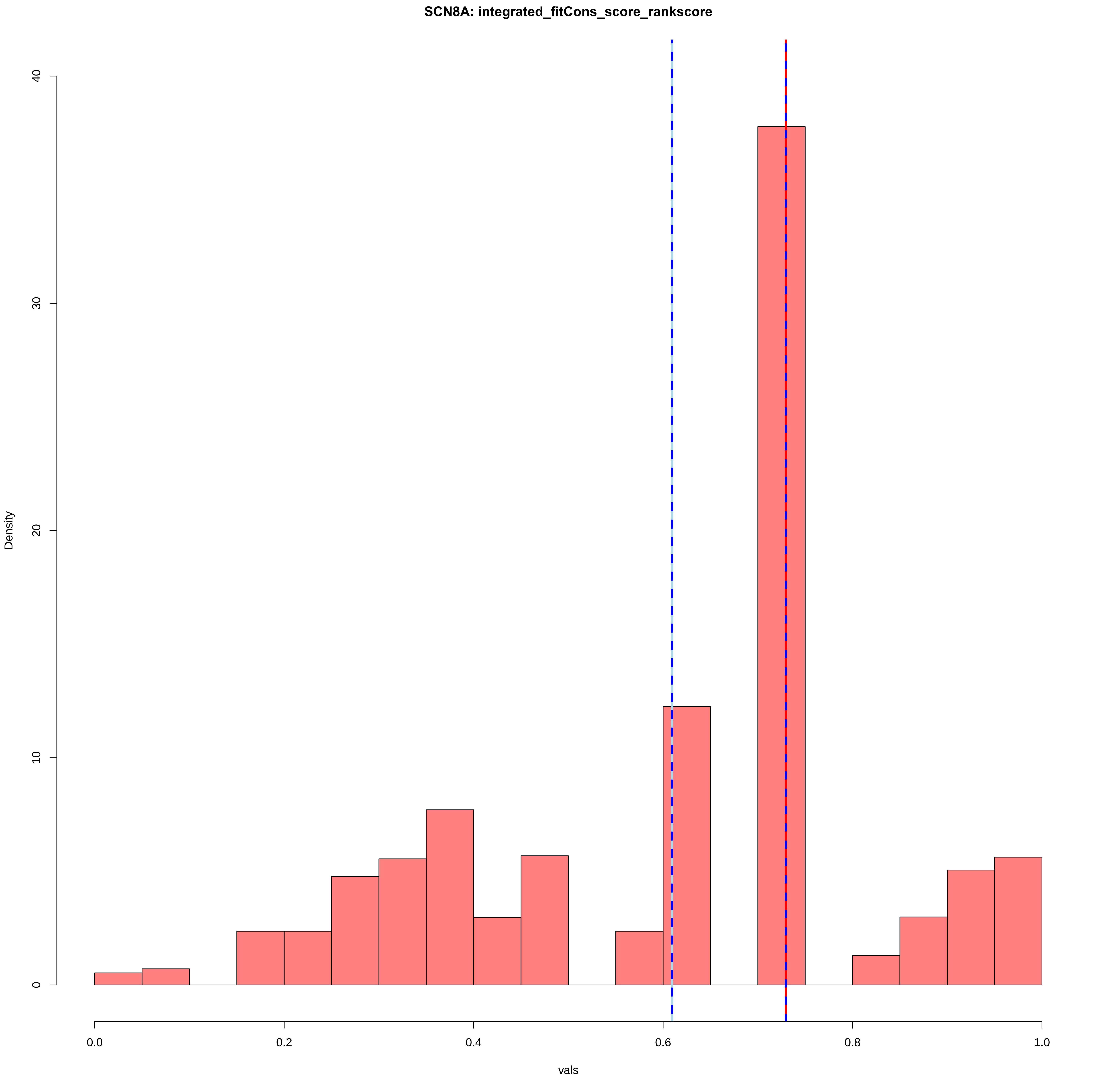
SCN8A: integrated_fitCons_score_rankscore
Density
0
10
20
30
40
0.0
0.2
0.4
0.6
0.8
1.0
vals

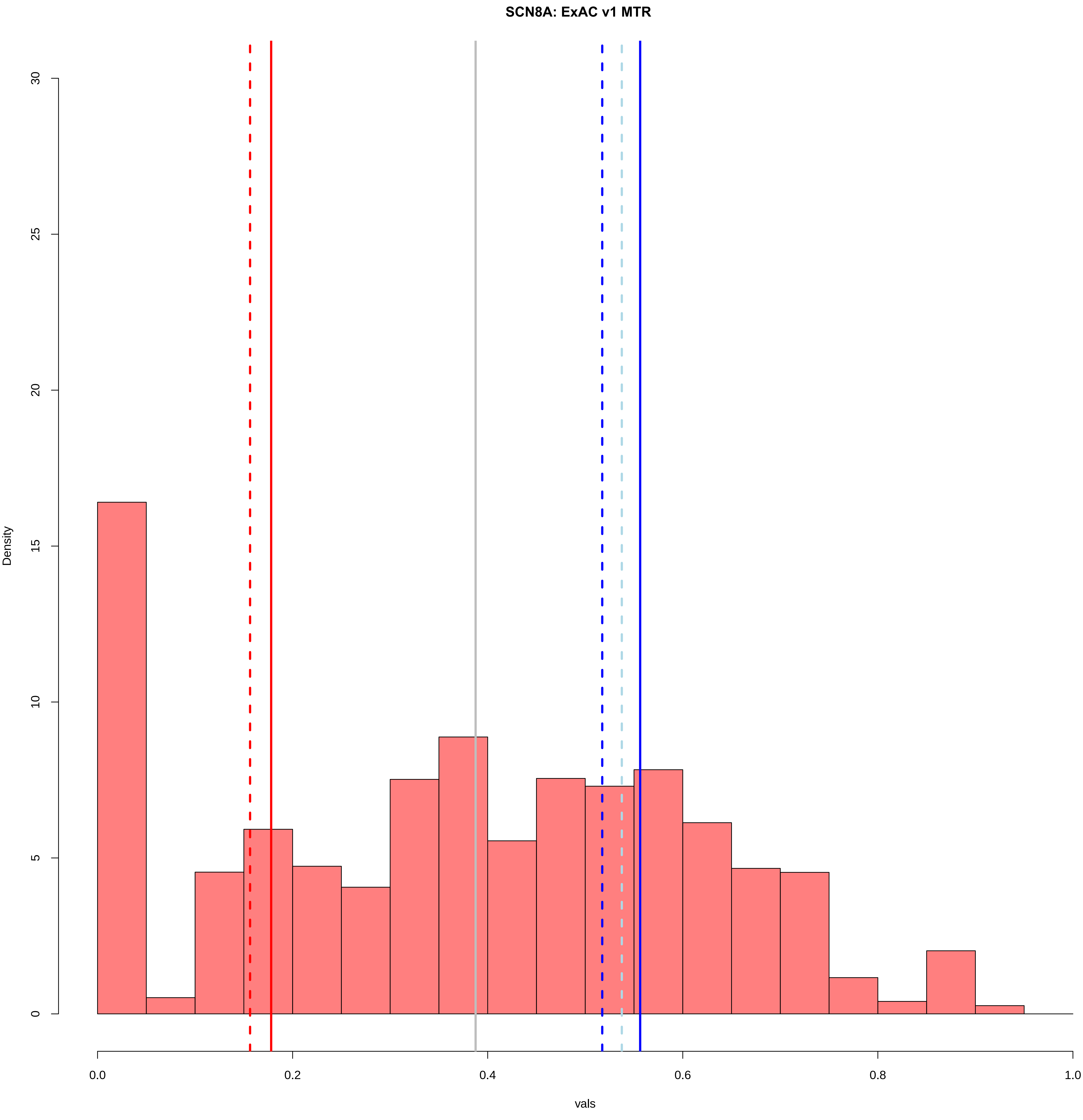

SCN8A: ExAC v1 MTR
Density
vals
0
5
10
15
20
25
30
0.0
0.2
0.4
0.6
0.8
1.0

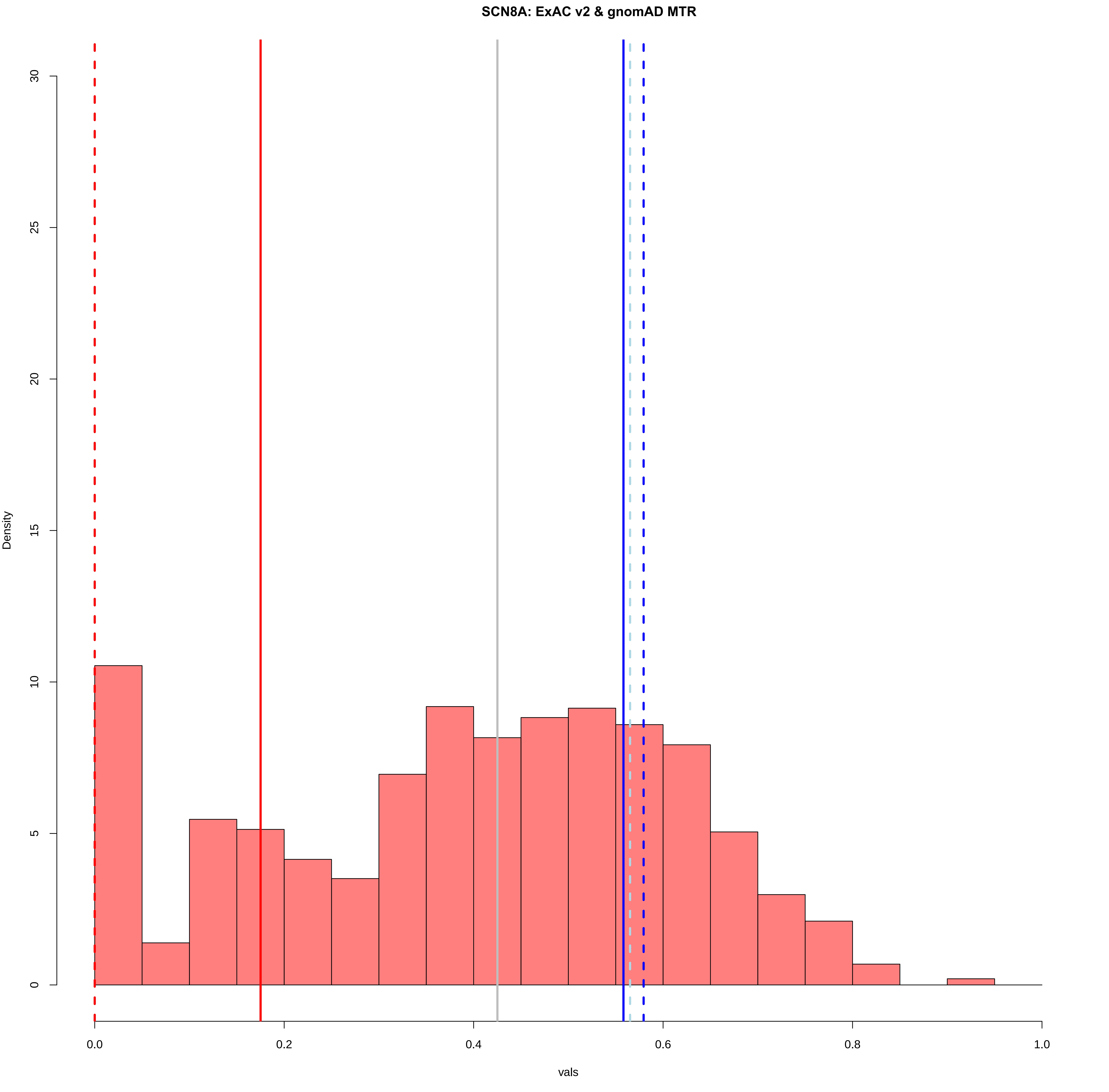

SCN8A: ExAC v2 & gnomAD MTR
Density
0
5
10
15
20
25
30
0.0
0.2
0.4
0.6
0.8
1.0
vals

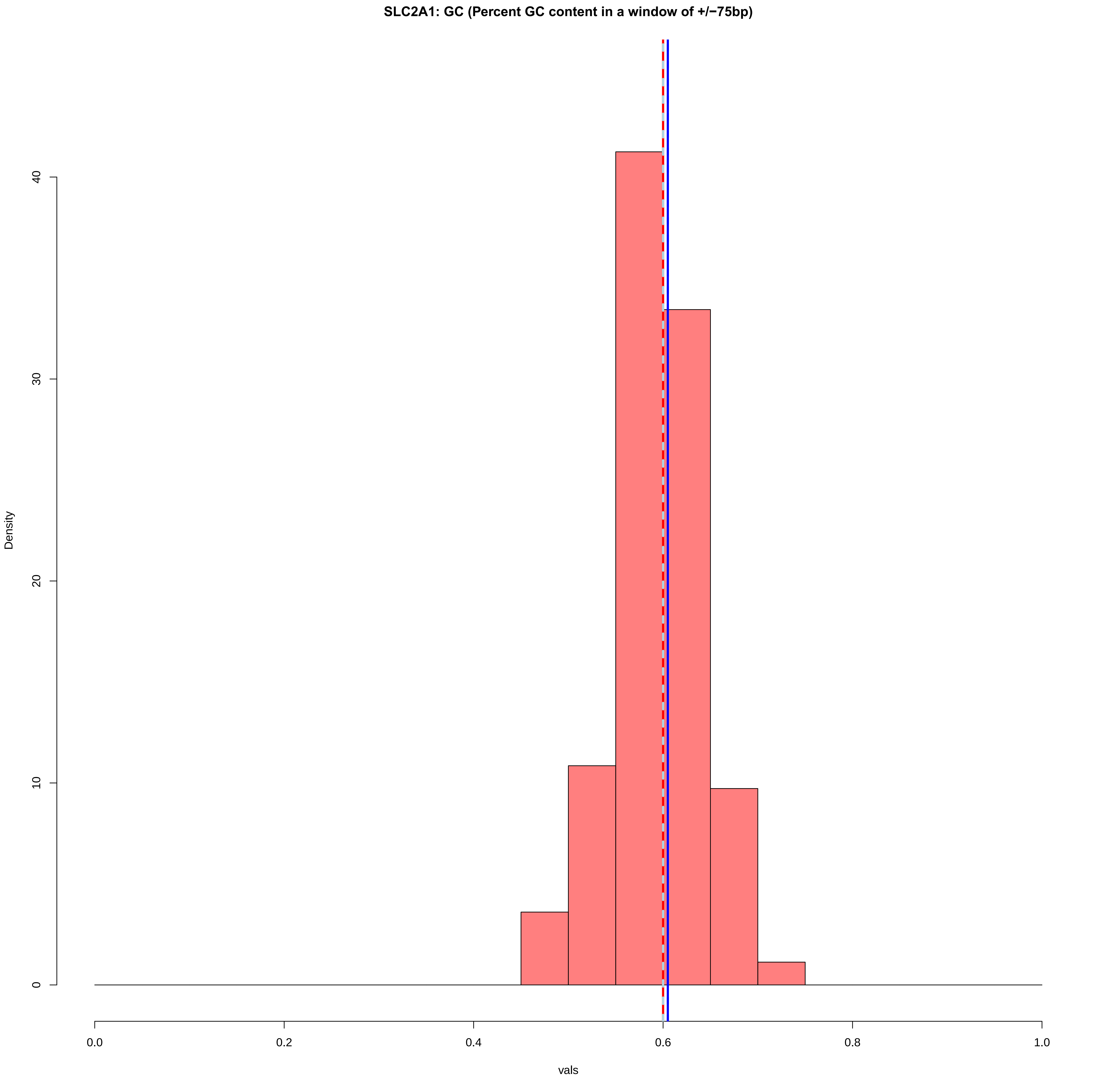
SLC2A1: GC (Percent GC content in a window of +/−75bp)
Density
vals
0
10
20
30
40
0.0
0.2
0.4
0.6
0.8
1.0

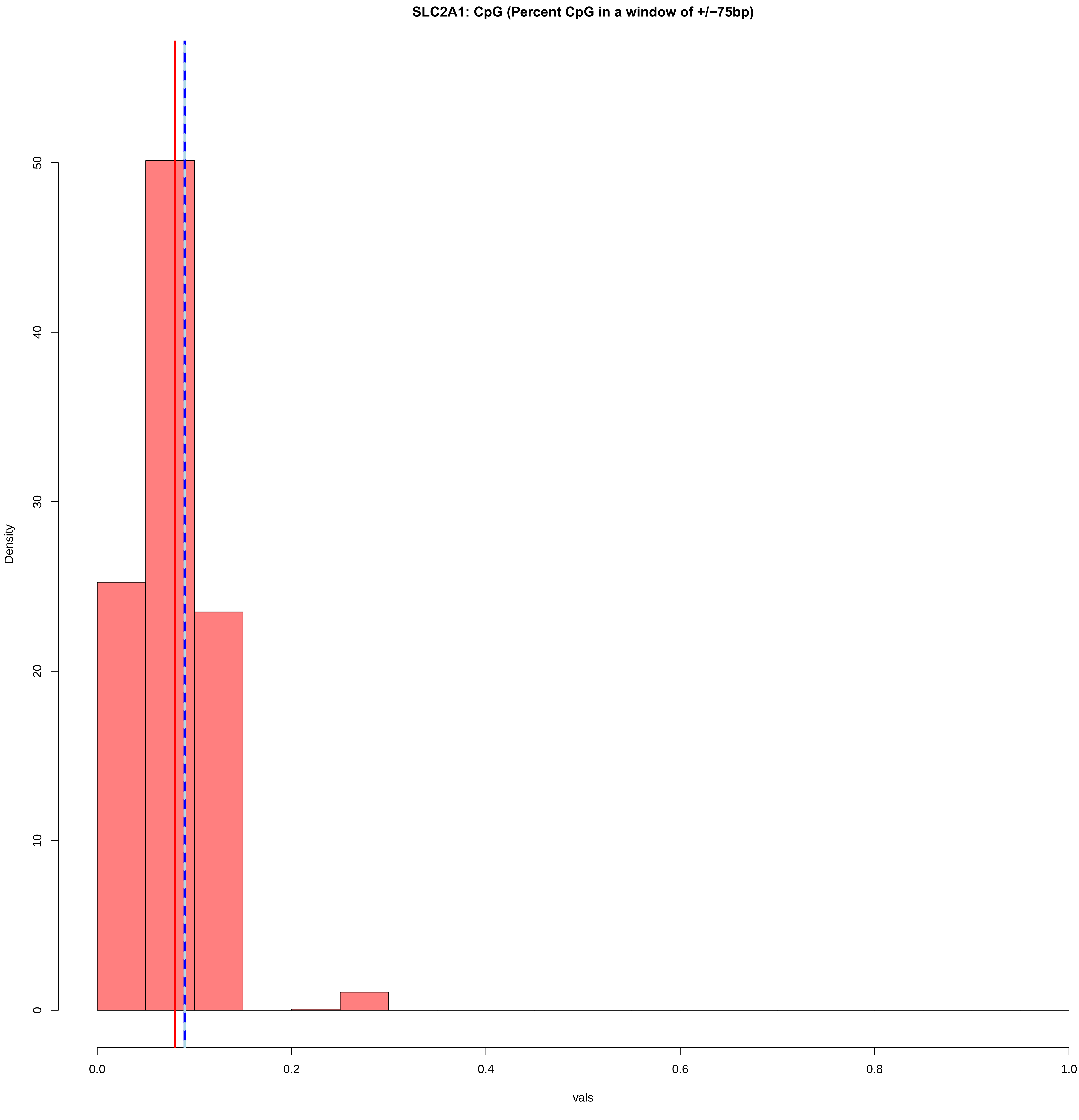
SLC2A1: CpG (Percent CpG in a window of +/−75bp)
Density
vals
0
10
20
30
40
50
0.0
0.2
0.4
0.6
0.8
1.0

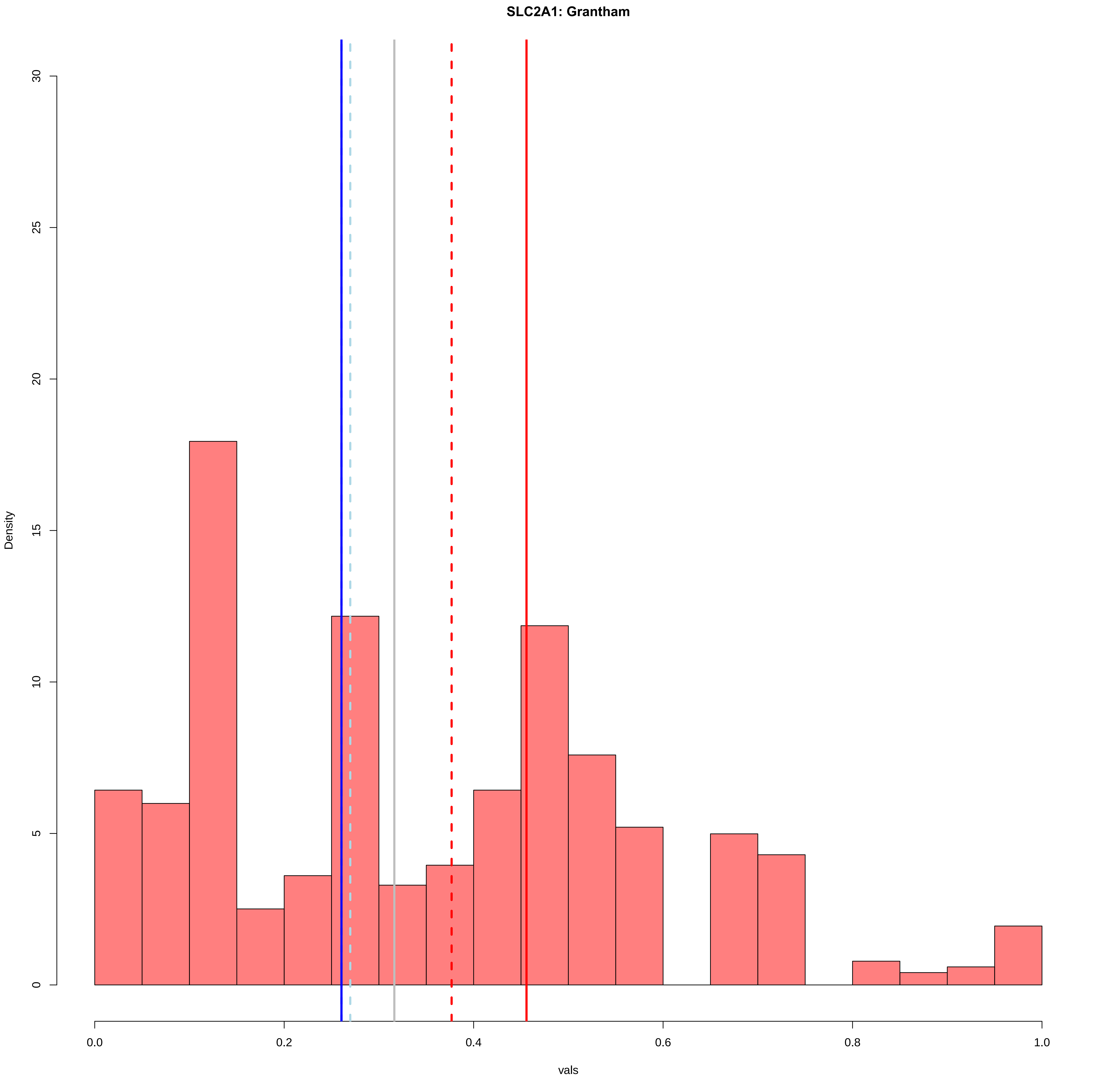

SLC2A1: Grantham
Density
0
5
10
15
20
25
30
0.0
0.2
0.4
0.6
0.8
1.0
vals

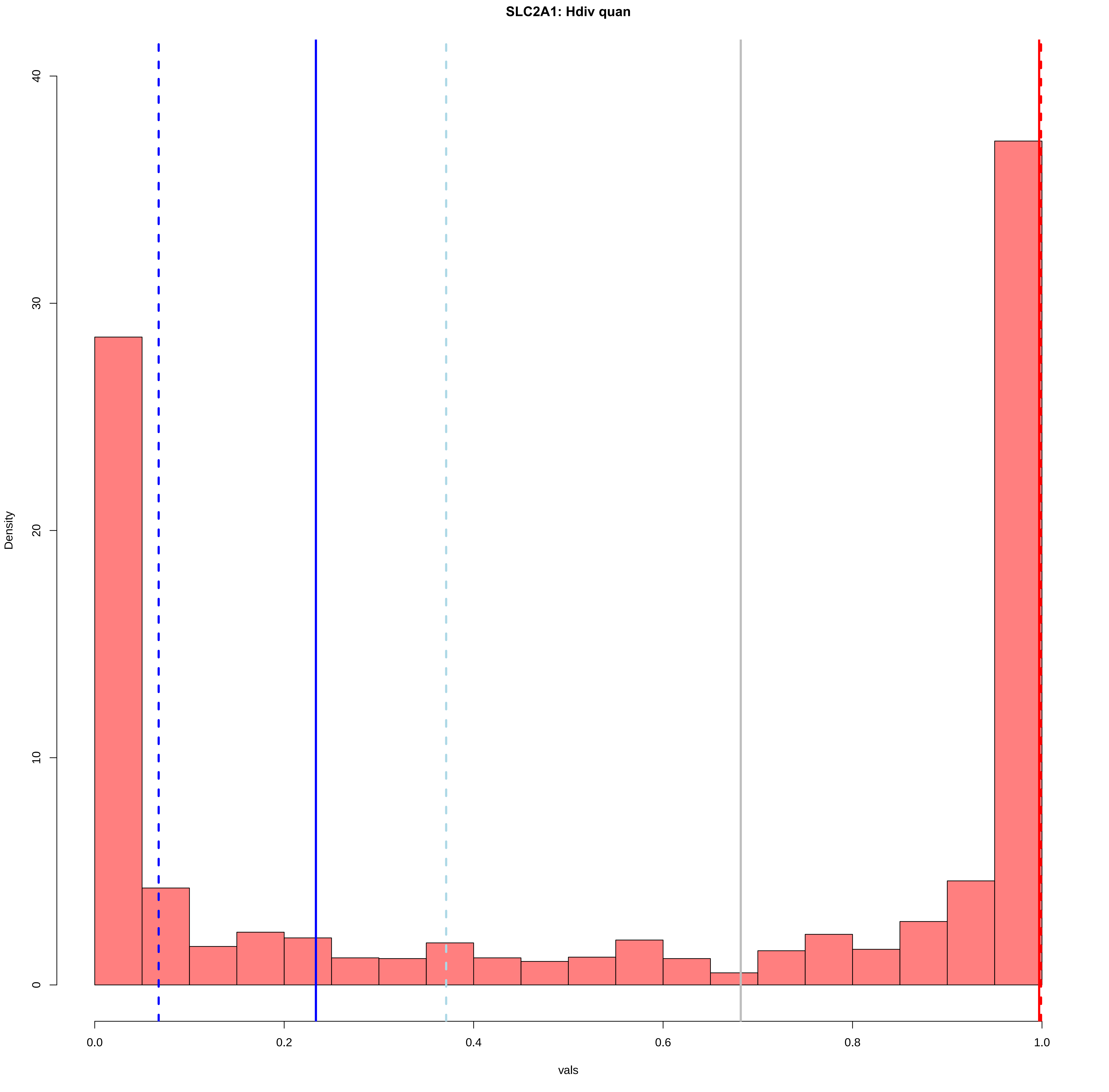

SLC2A1: Hdiv quan
Density
vals
0
10
20
30
40
0.0
0.2
0.4
0.6
0.8
1.0

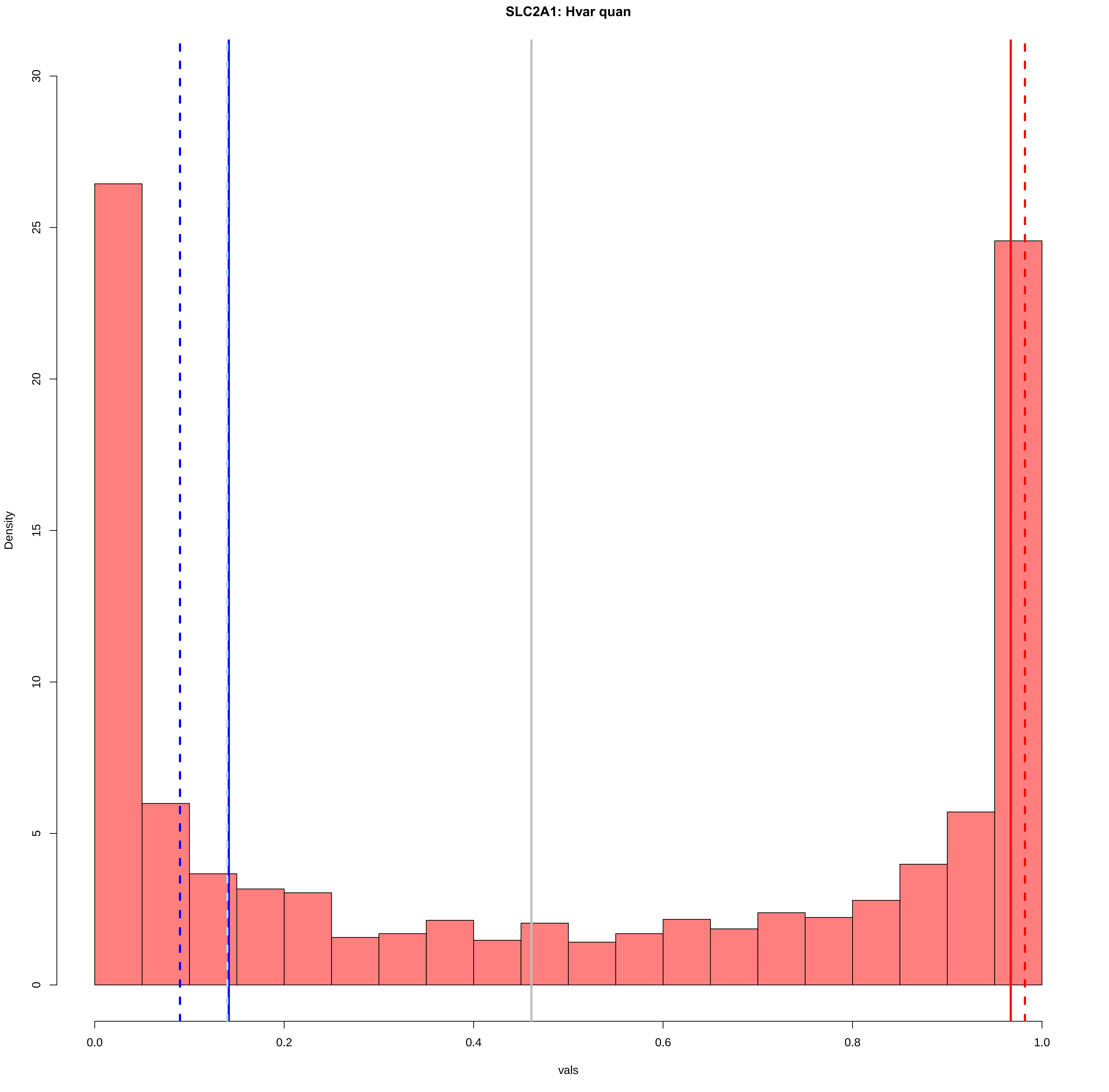
SLC2A1: Hvar quan
Density
vals
0
5
10
15
20
25
30
0.0
0.2
0.4
0.6
0.8
1.0

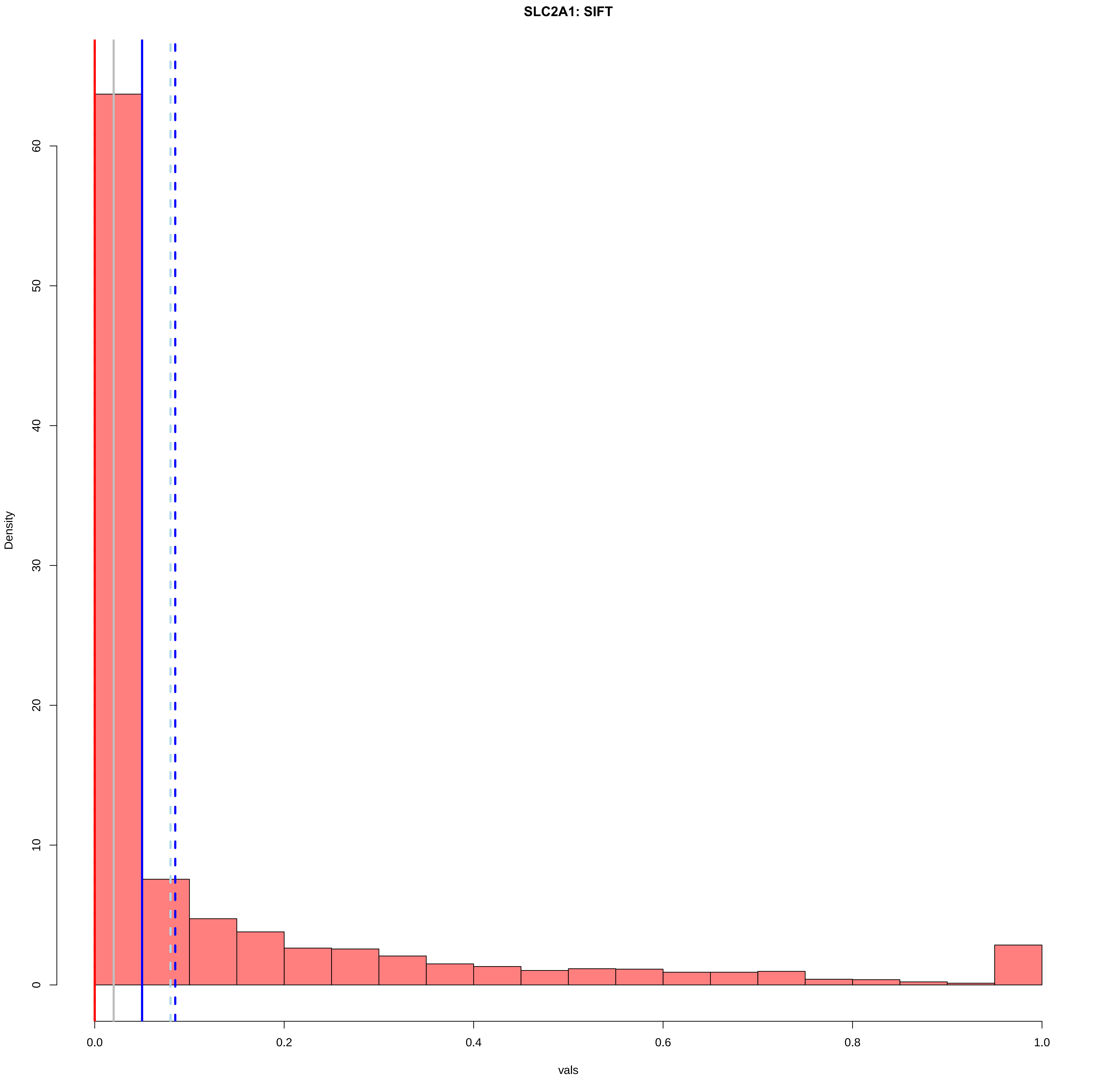
SLC2A1: SIFT
Density
vals
0
10
20
30
40
50
60
0.0
0.2
0.4
0.6
0.8
1.0

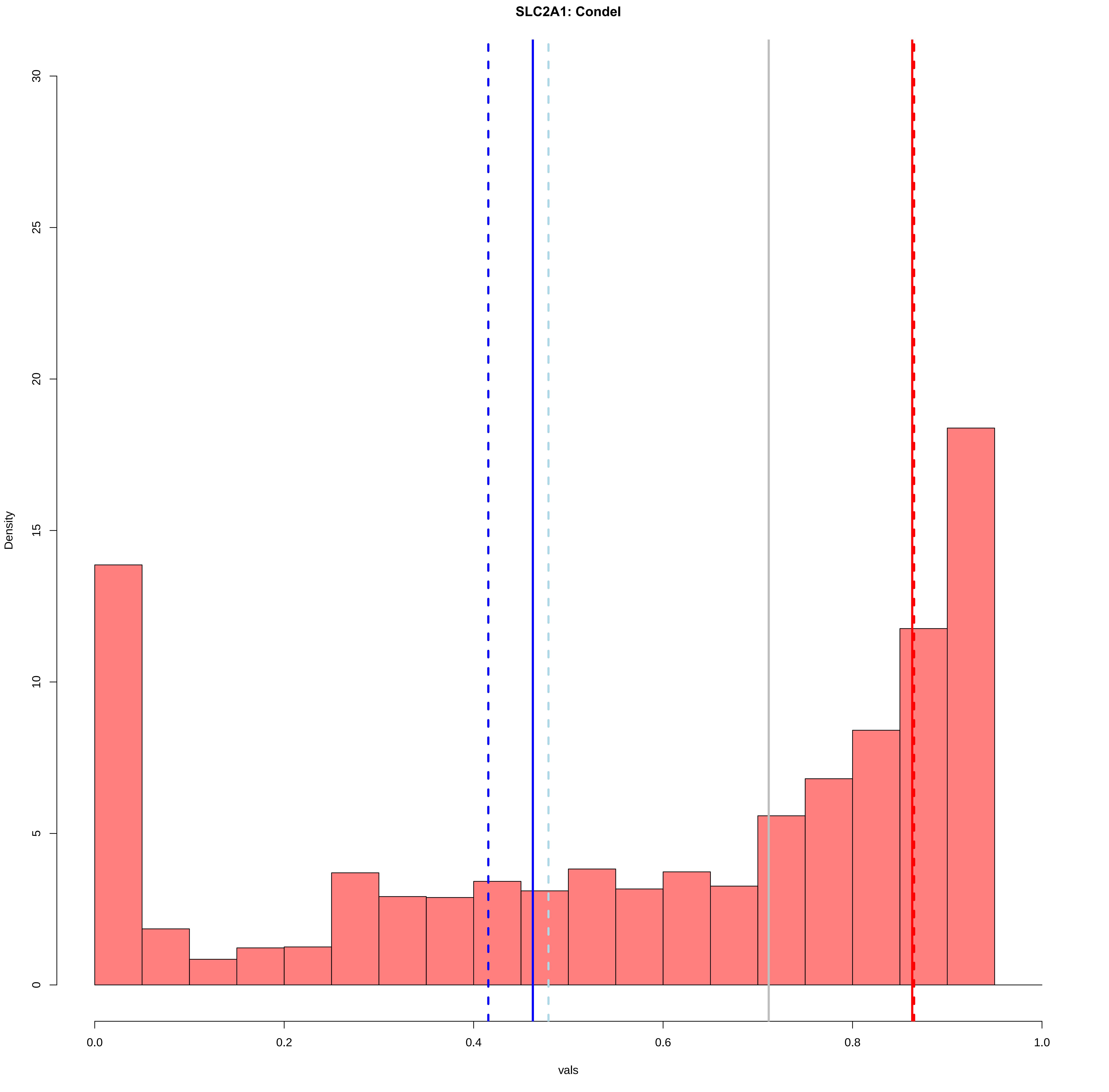

SLC2A1: Condel
Density
vals
0
5
10
15
20
25
30
0.0
0.2
0.4
0.6
0.8
1.0

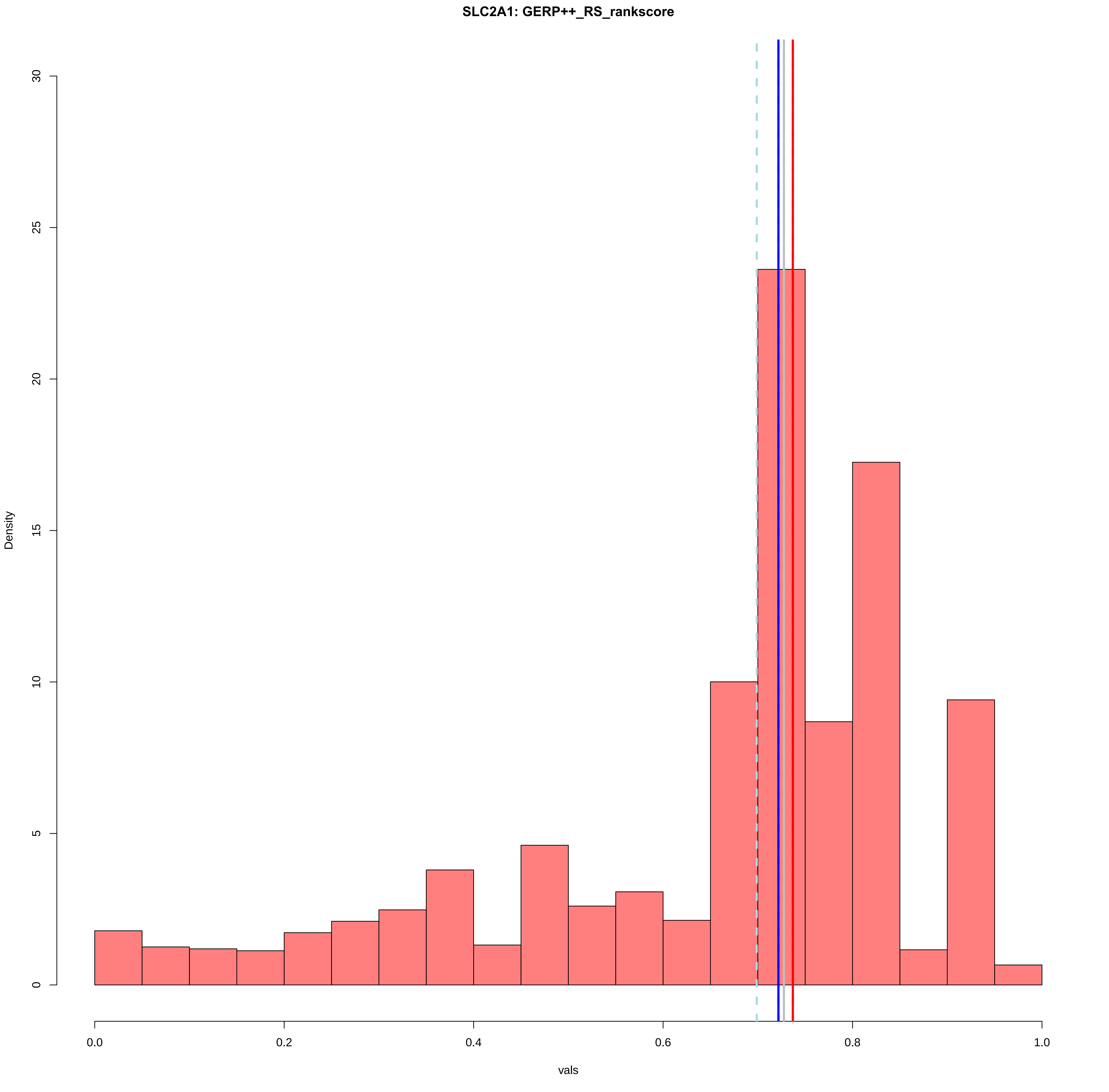

SLC2A1: GERP++_RS_rankscore
Density
vals
0
5
10
15
20
25
30
0.0
0.2
0.4
0.6
0.8
1.0

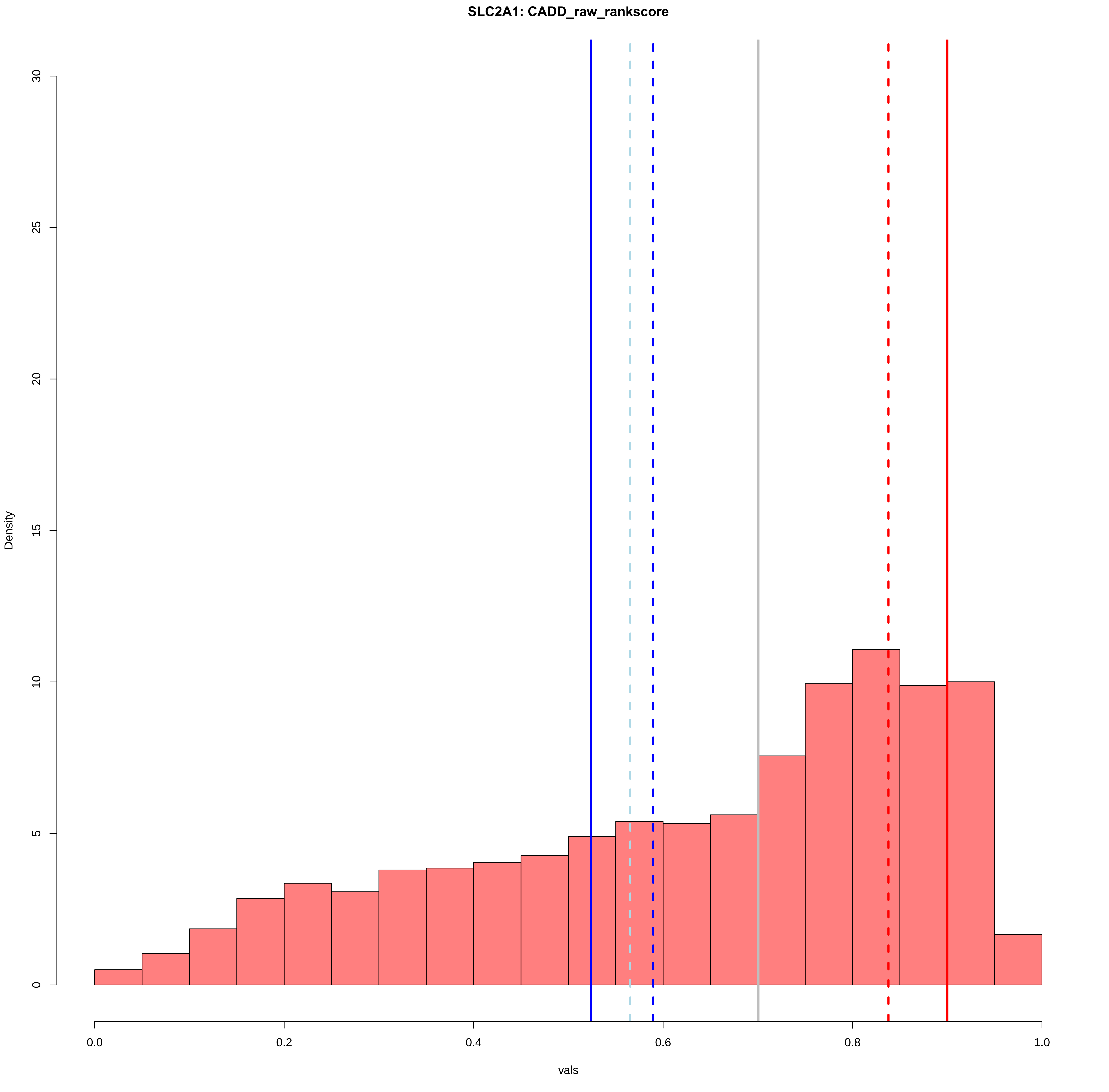
SLC2A1: CADD_raw_rankscore
Density
vals
0
5
10
15
20
25
30
0.0
0.2
0.4
0.6
0.8
1.0

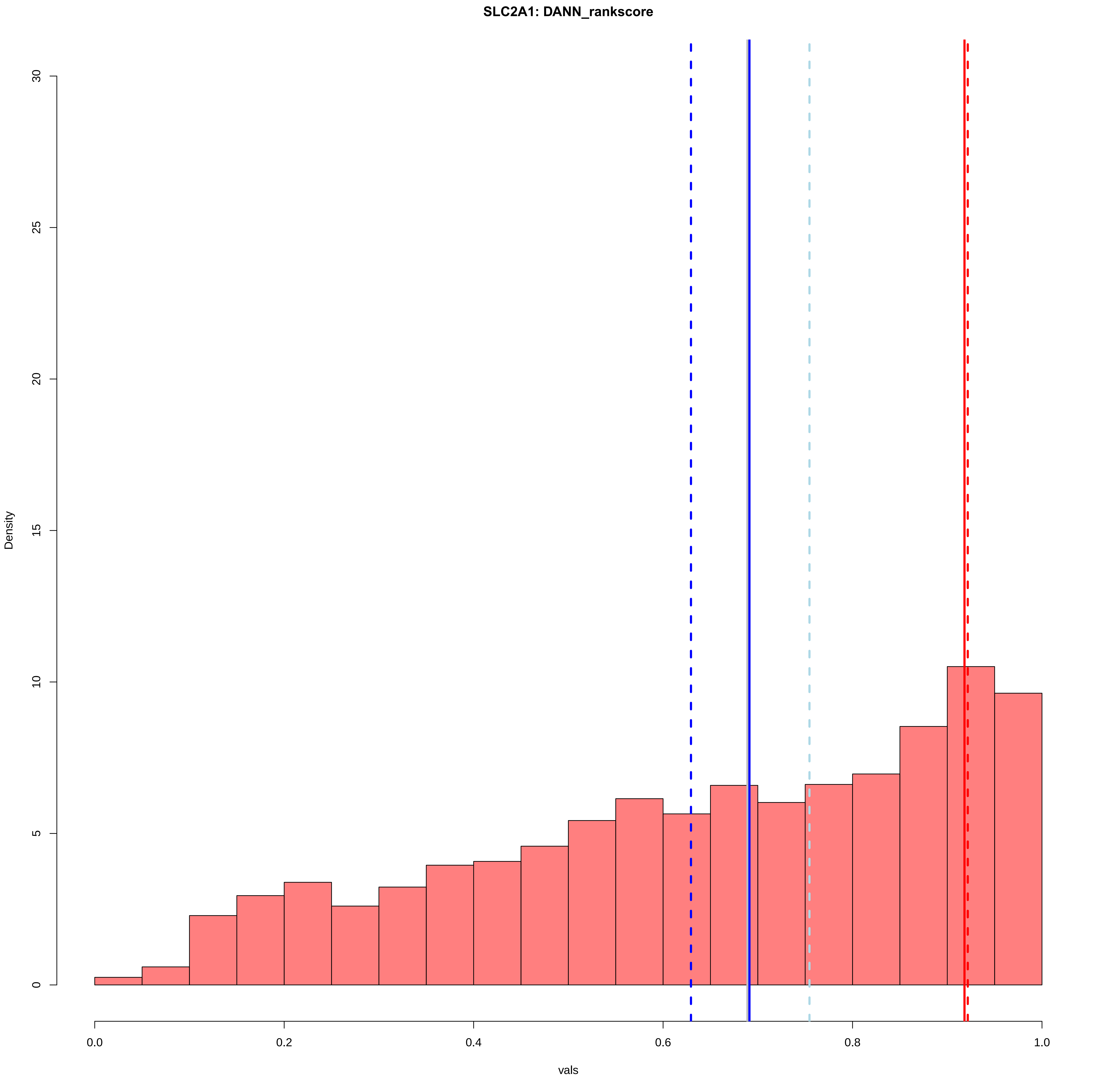

SLC2A1: DANN_rankscore
Density
vals
0
5
10
15
20
25
30
0.0
0.2
0.4
0.6
0.8
1.0

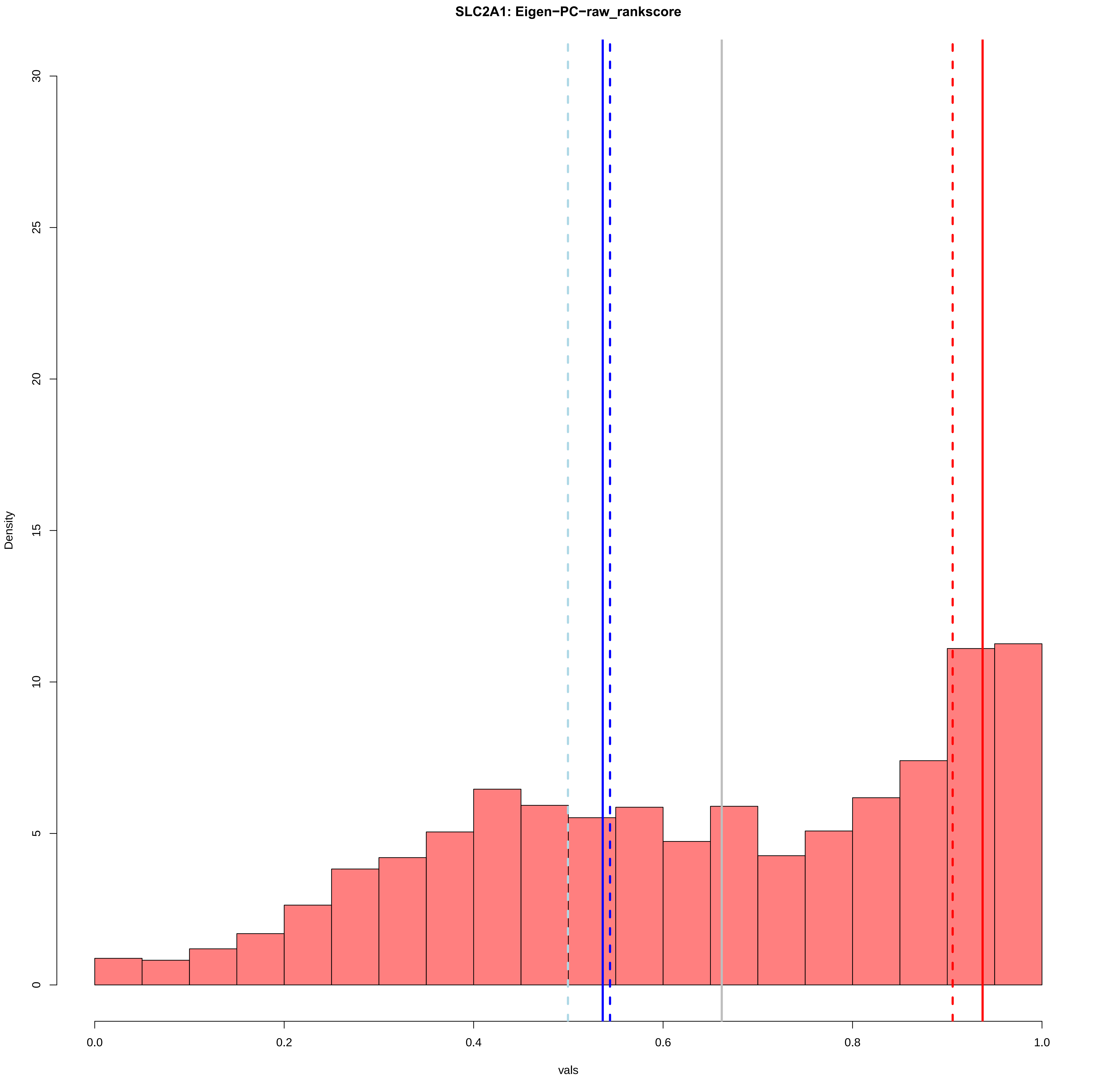

SLC2A1: Eigen−PC−raw_rankscore
Density
vals
0
5
10
15
20
25
30
0.0
0.2
0.4
0.6
0.8
1.0

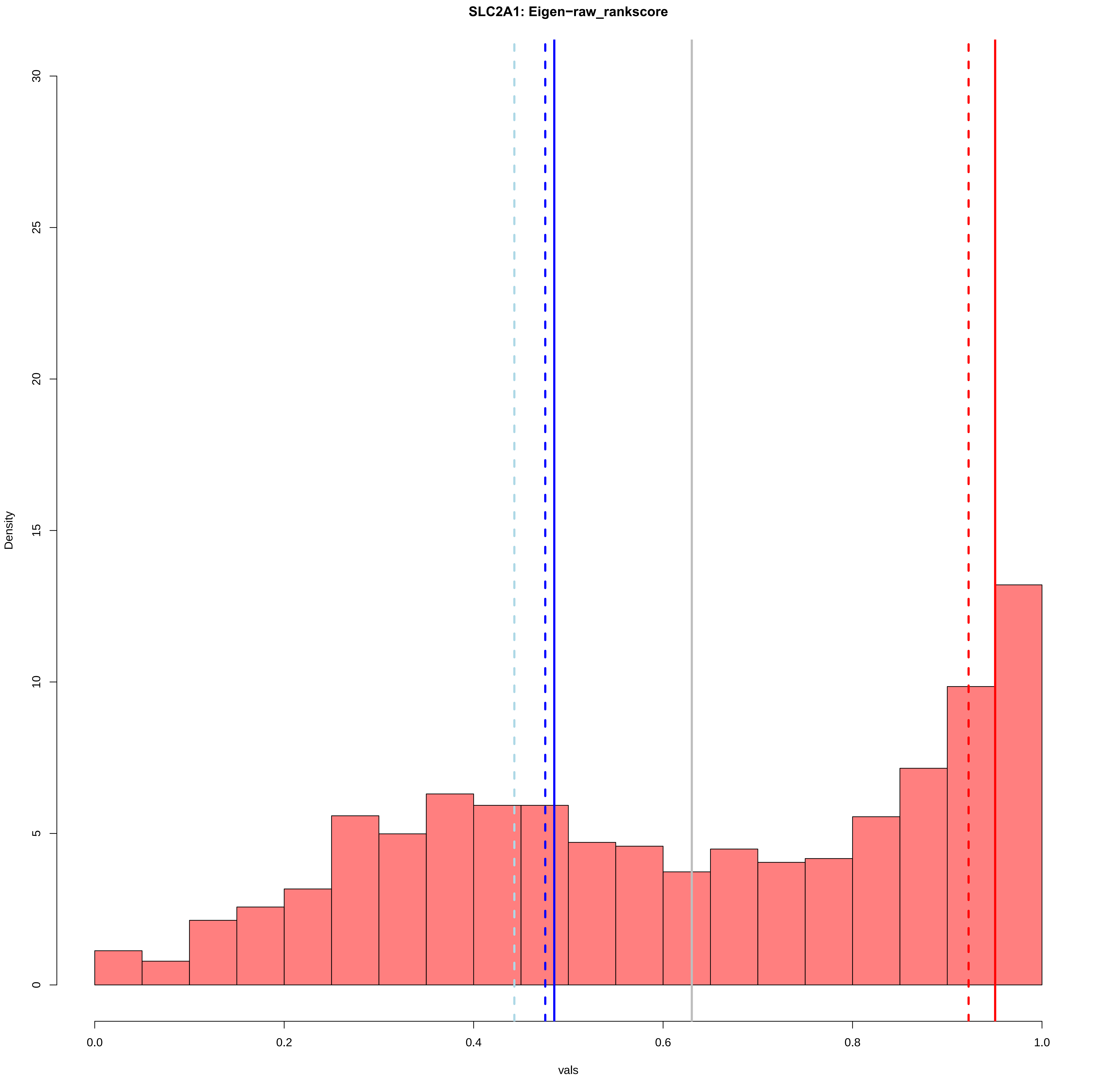

SLC2A1: Eigen−raw_rankscore
Density
vals
0
5
10
15
20
25
30
0.0
0.2
0.4
0.6
0.8
1.0

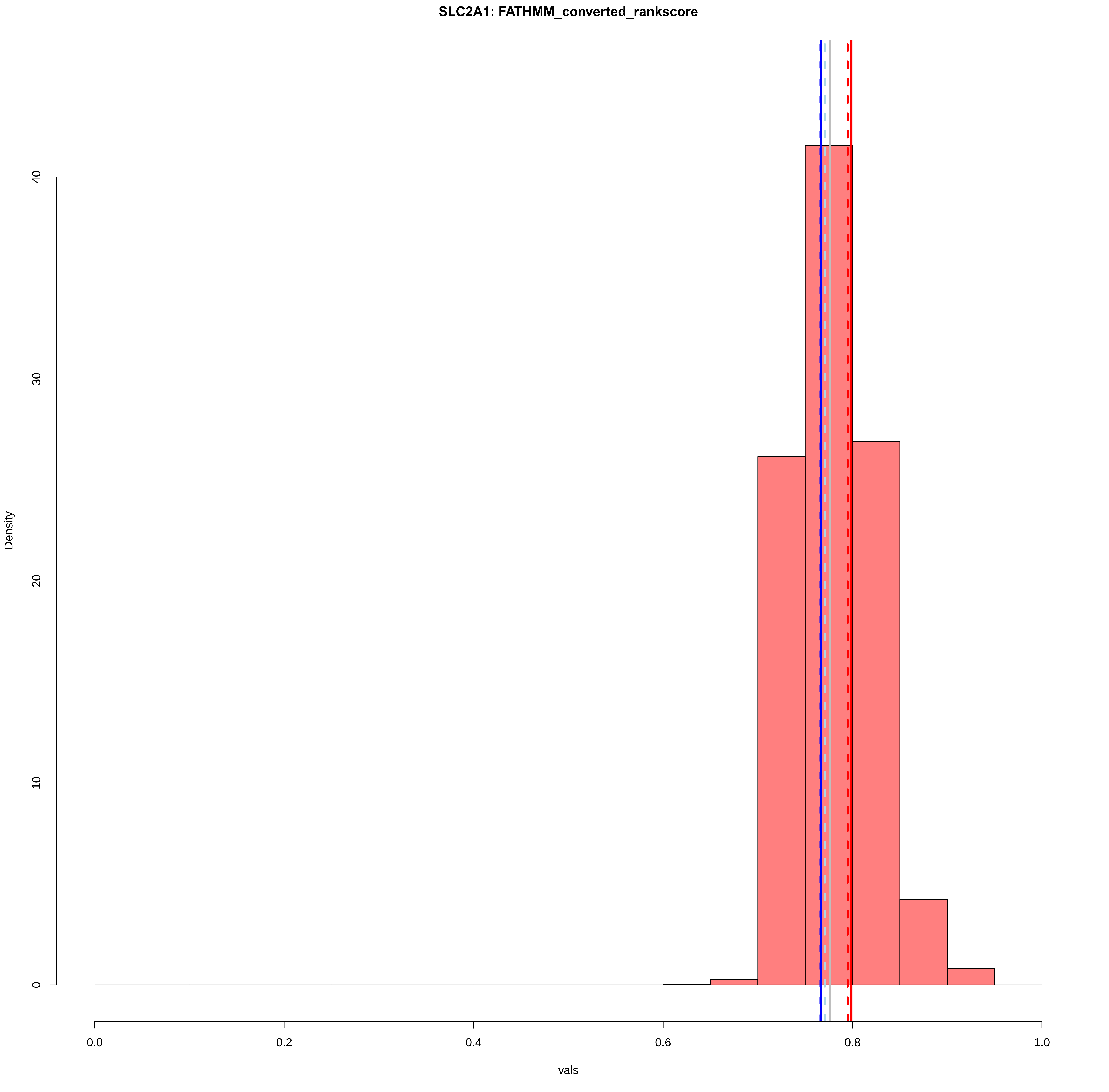
SLC2A1: FATHMM_converted_rankscore
Density
vals
0
10
20
30
40
0.0
0.2
0.4
0.6
0.8
1.0

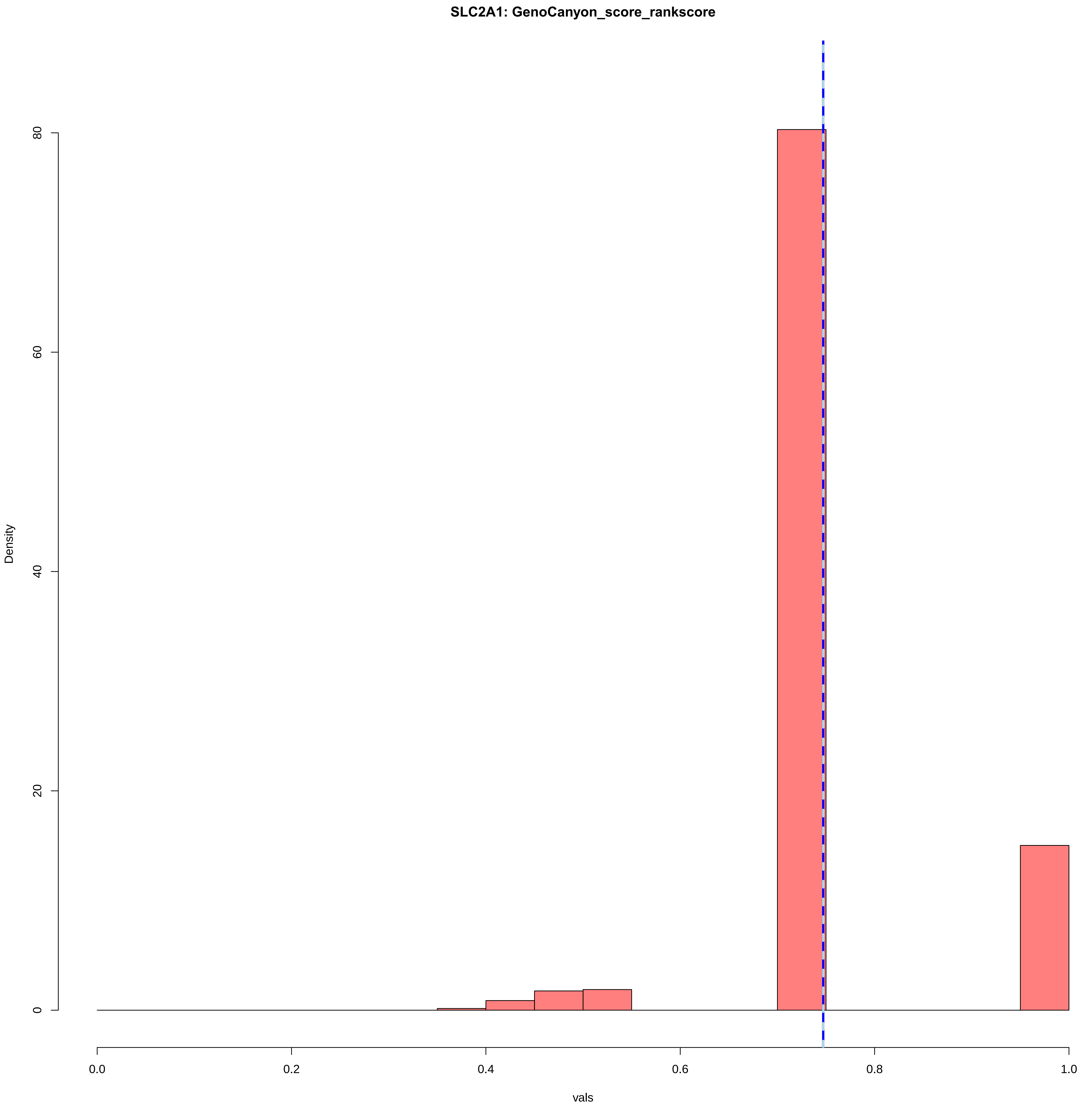
SLC2A1: GenoCanyon_score_rankscore
Density
vals
0
20
40
60
80
0.0
0.2
0.4
0.6
0.8
1.0

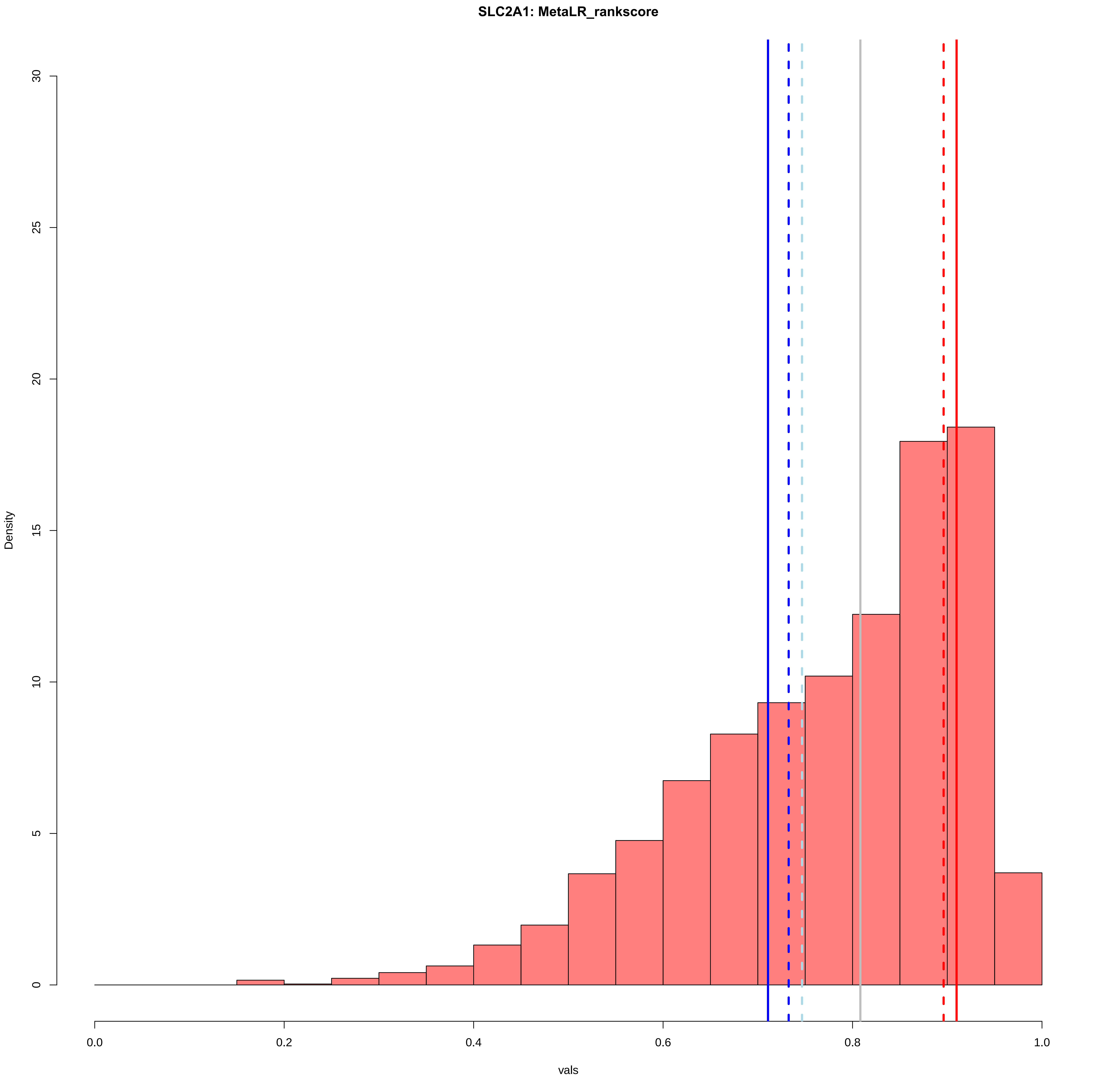

SLC2A1: MetaLR_rankscore
Density
vals
0
5
10
15
20
25
30
0.0
0.2
0.4
0.6
0.8
1.0

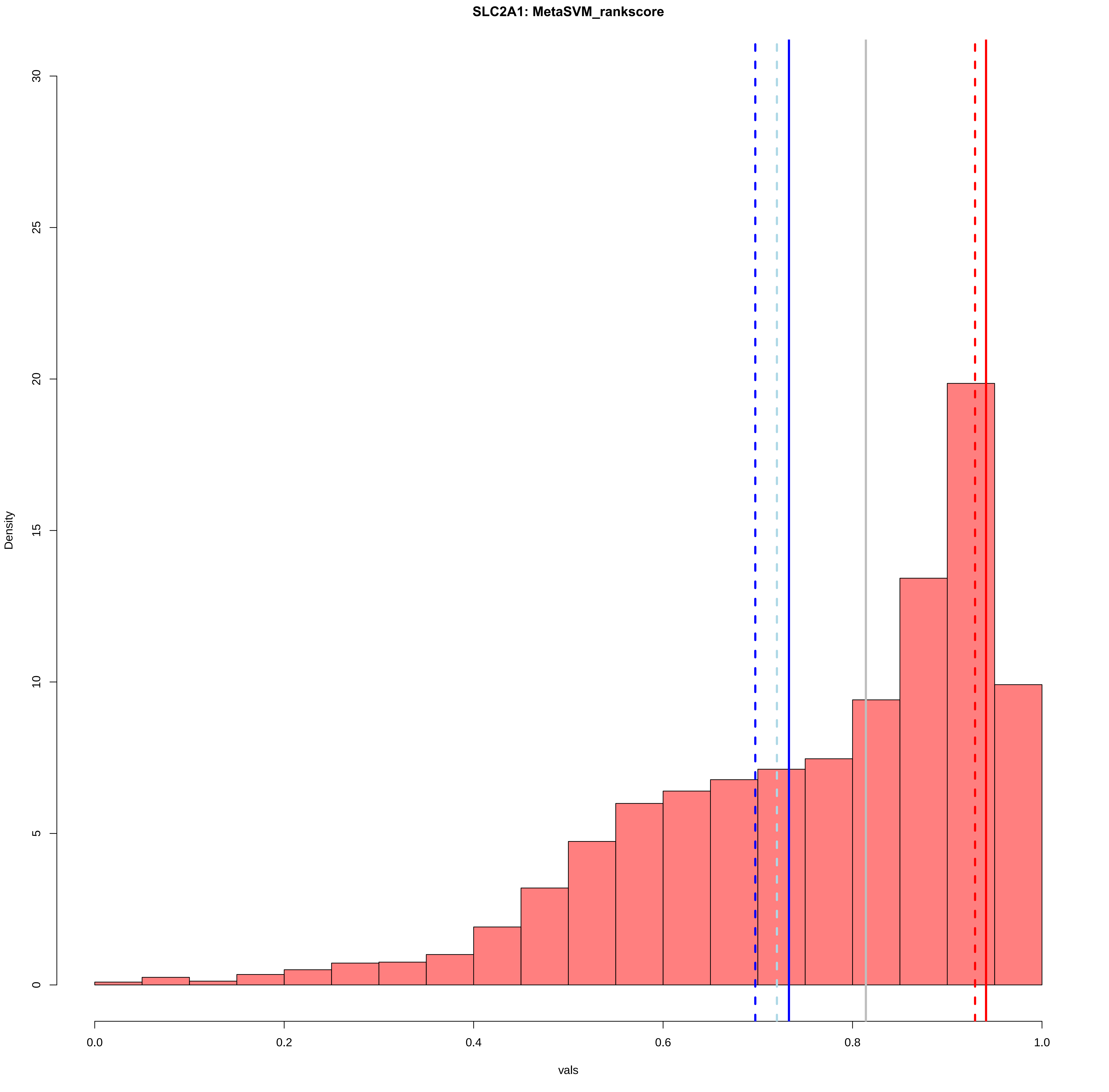

SLC2A1: MetaSVM_rankscore
Density
vals
0
5
10
15
20
25
30
0.0
0.2
0.4
0.6
0.8
1.0

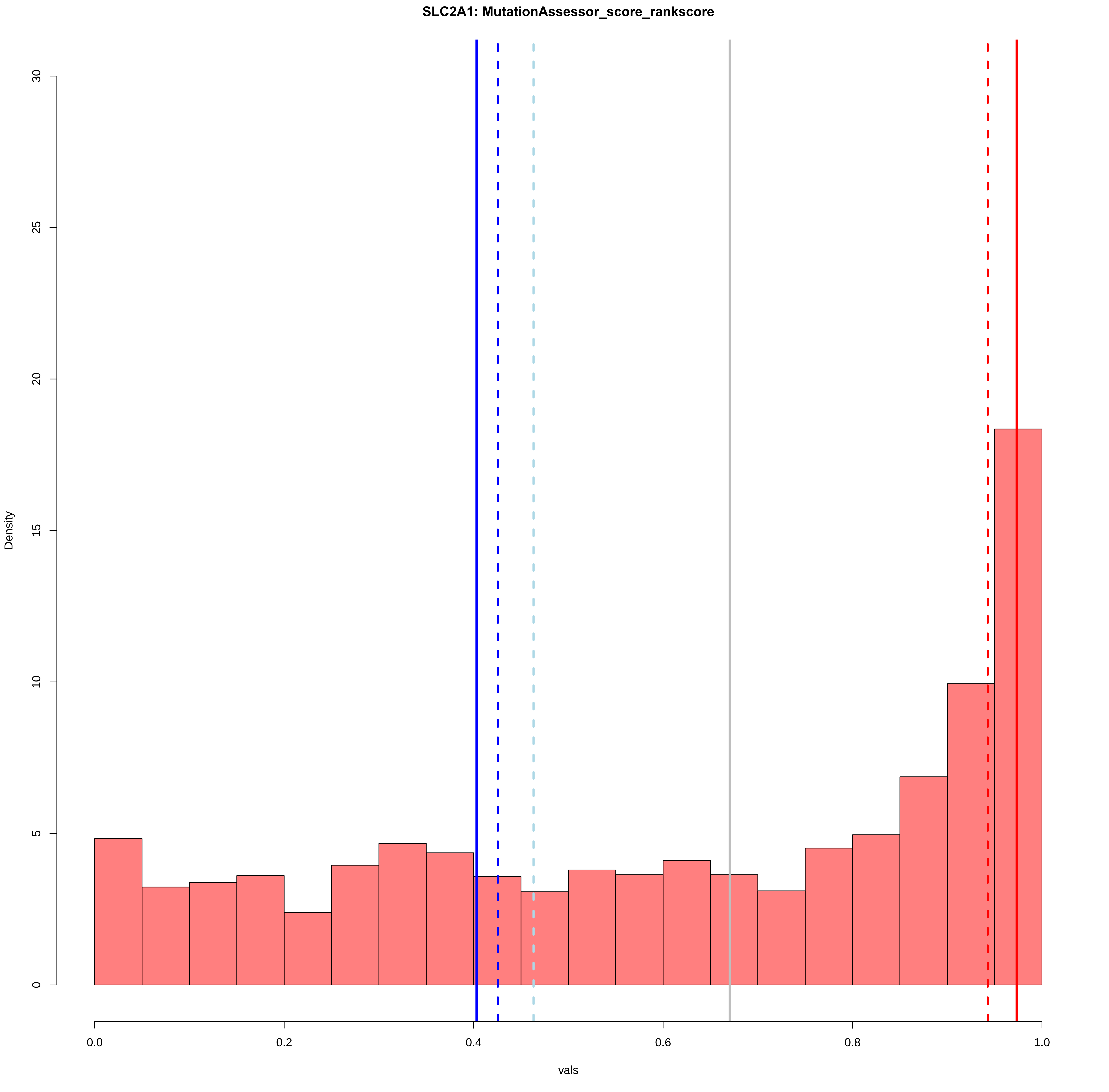

SLC2A1: MutationAssessor_score_rankscore
Density
vals
0
5
10
15
20
25
30
0.0
0.2
0.4
0.6
0.8
1.0

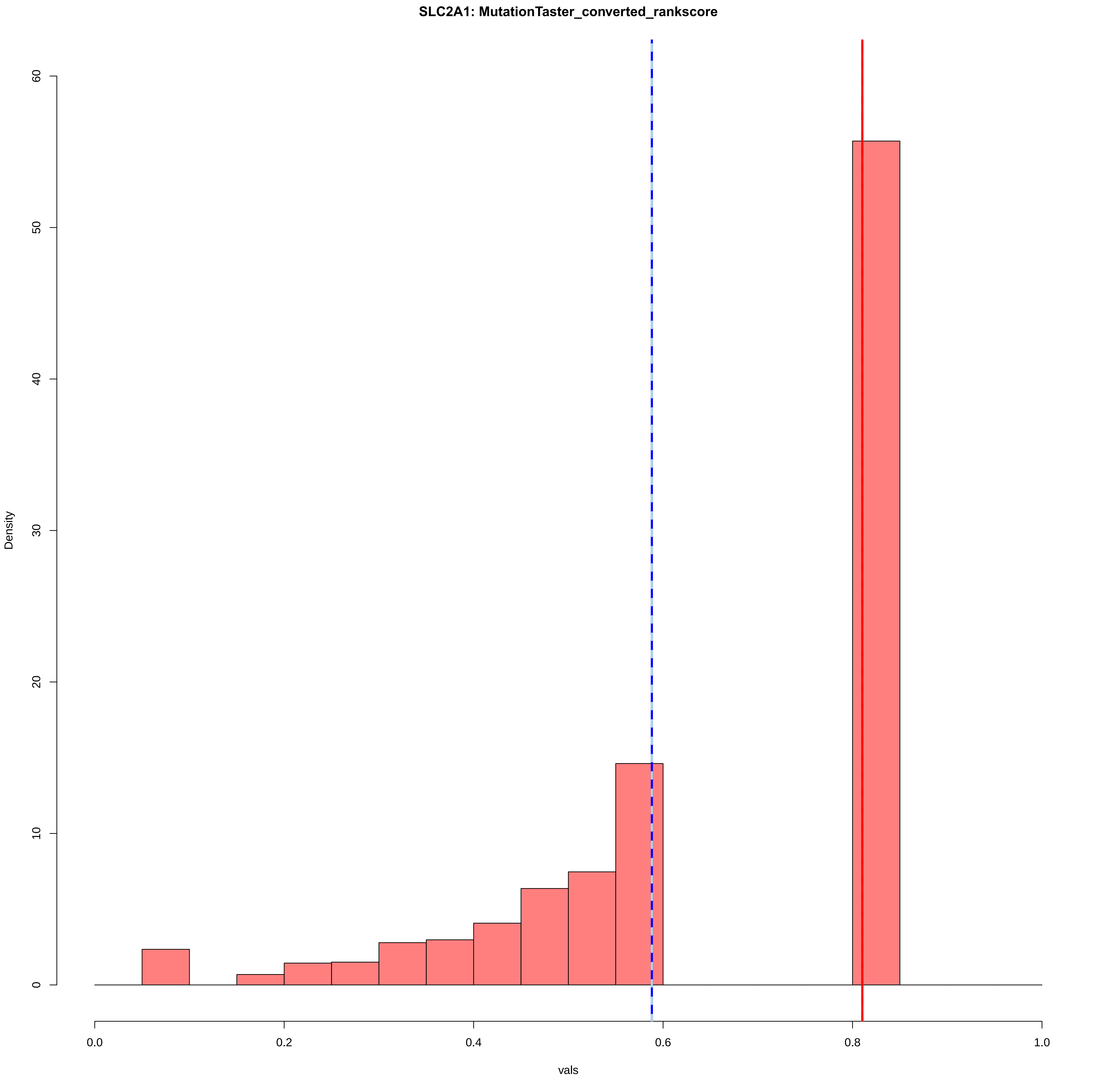
SLC2A1: MutationTaster_converted_rankscore
Density
vals
0
10
20
30
40
50
60
0.0
0.2
0.4
0.6
0.8
1.0

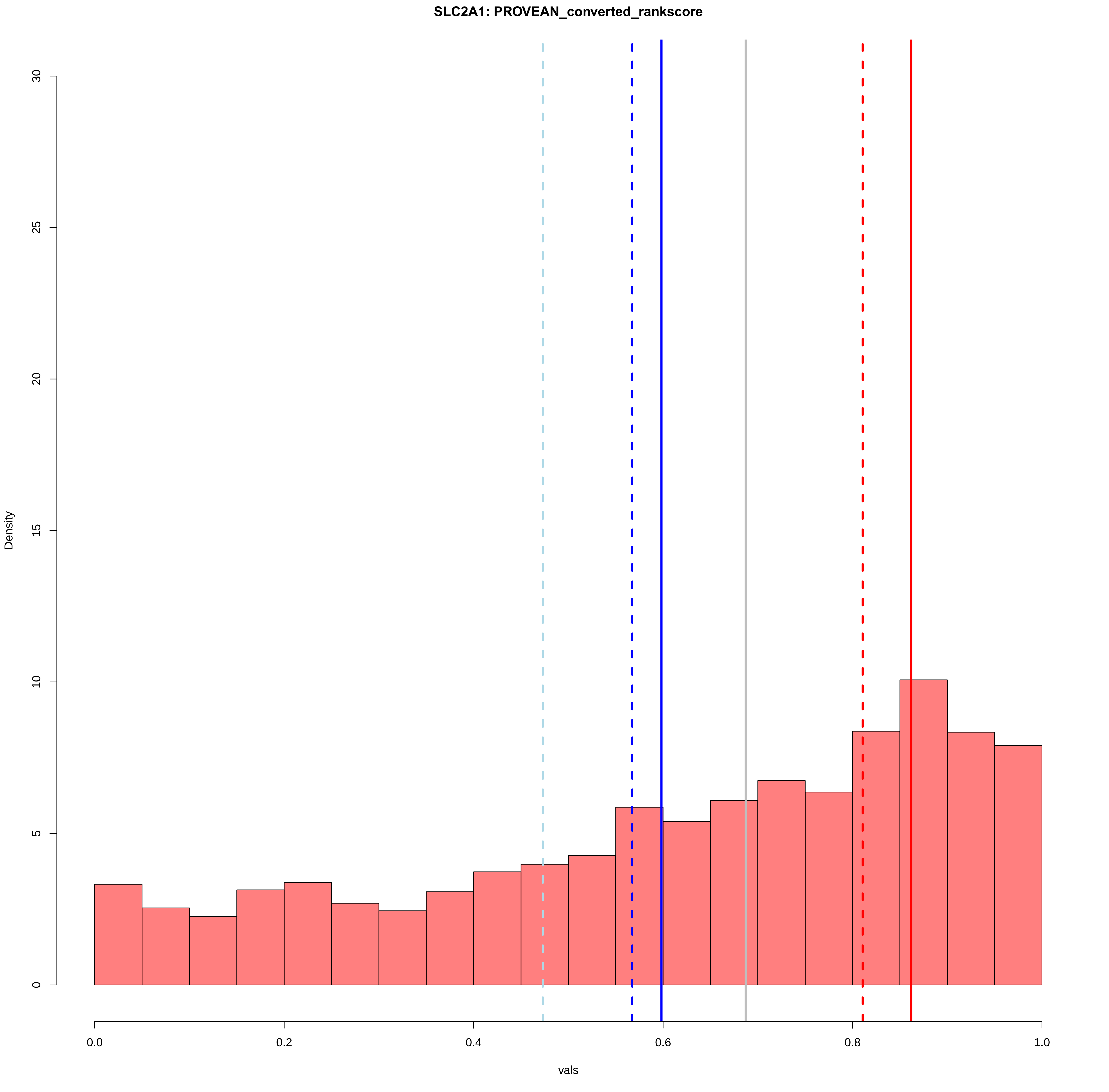

SLC2A1: PROVEAN_converted_rankscore
Density
vals
0
5
10
15
20
25
30
0.0
0.2
0.4
0.6
0.8
1.0

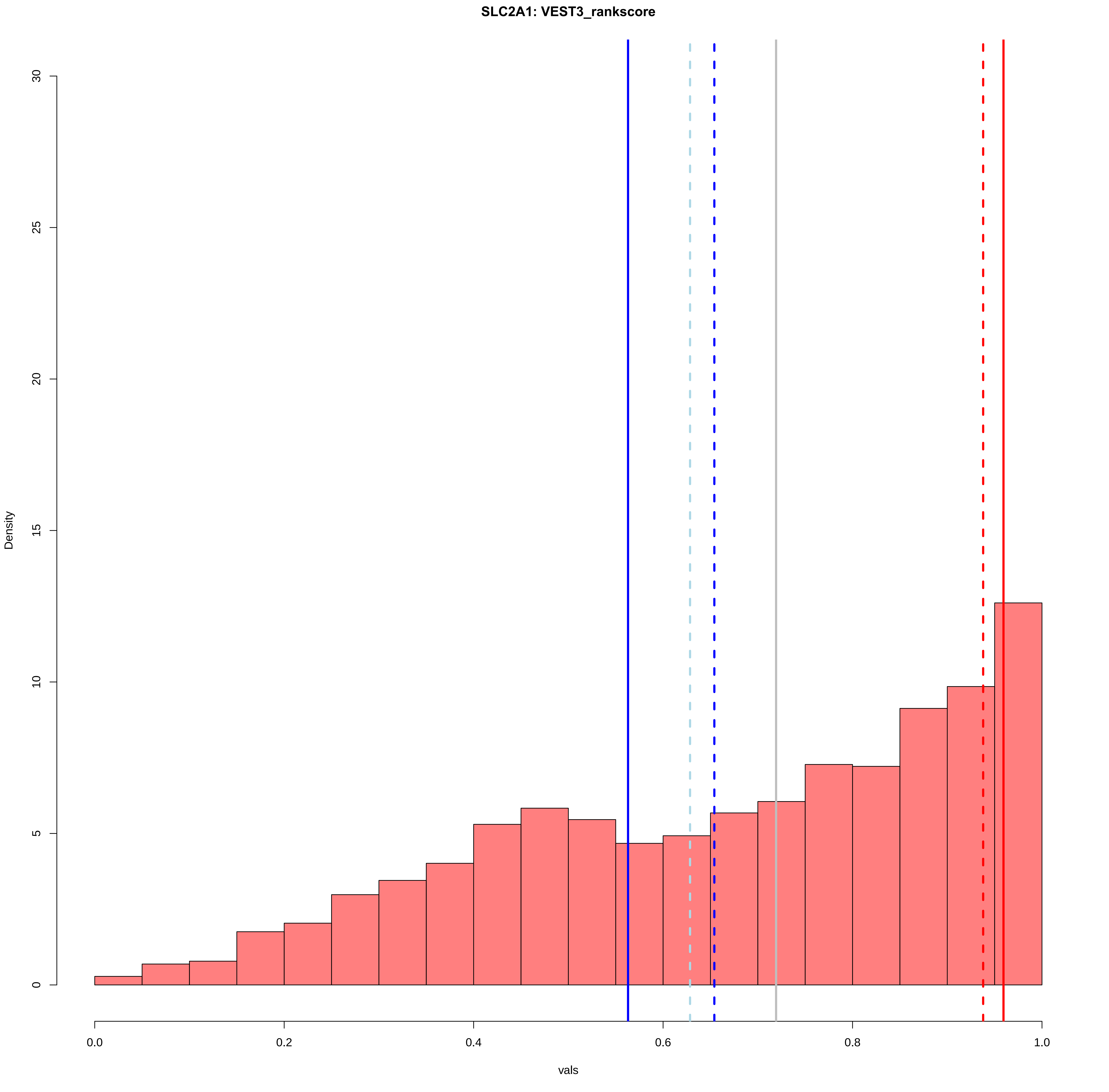

SLC2A1: VEST3_rankscore
Density
vals
0
5
10
15
20
25
30
0.0
0.2
0.4
0.6
0.8
1.0

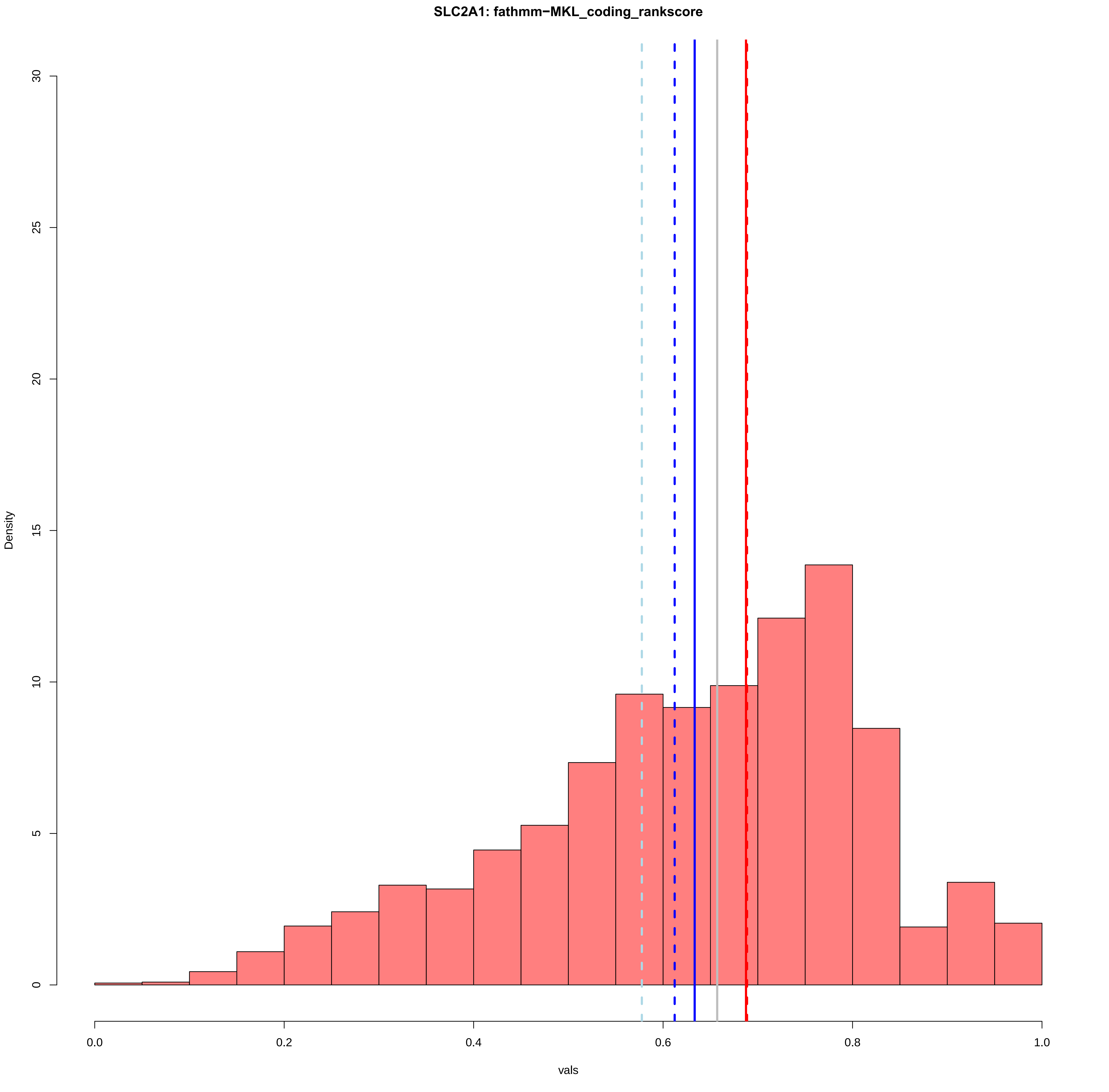

SLC2A1: fathmm−MKL_coding_rankscore
Density
vals
0
5
10
15
20
25
30
0.0
0.2
0.4
0.6
0.8
1.0

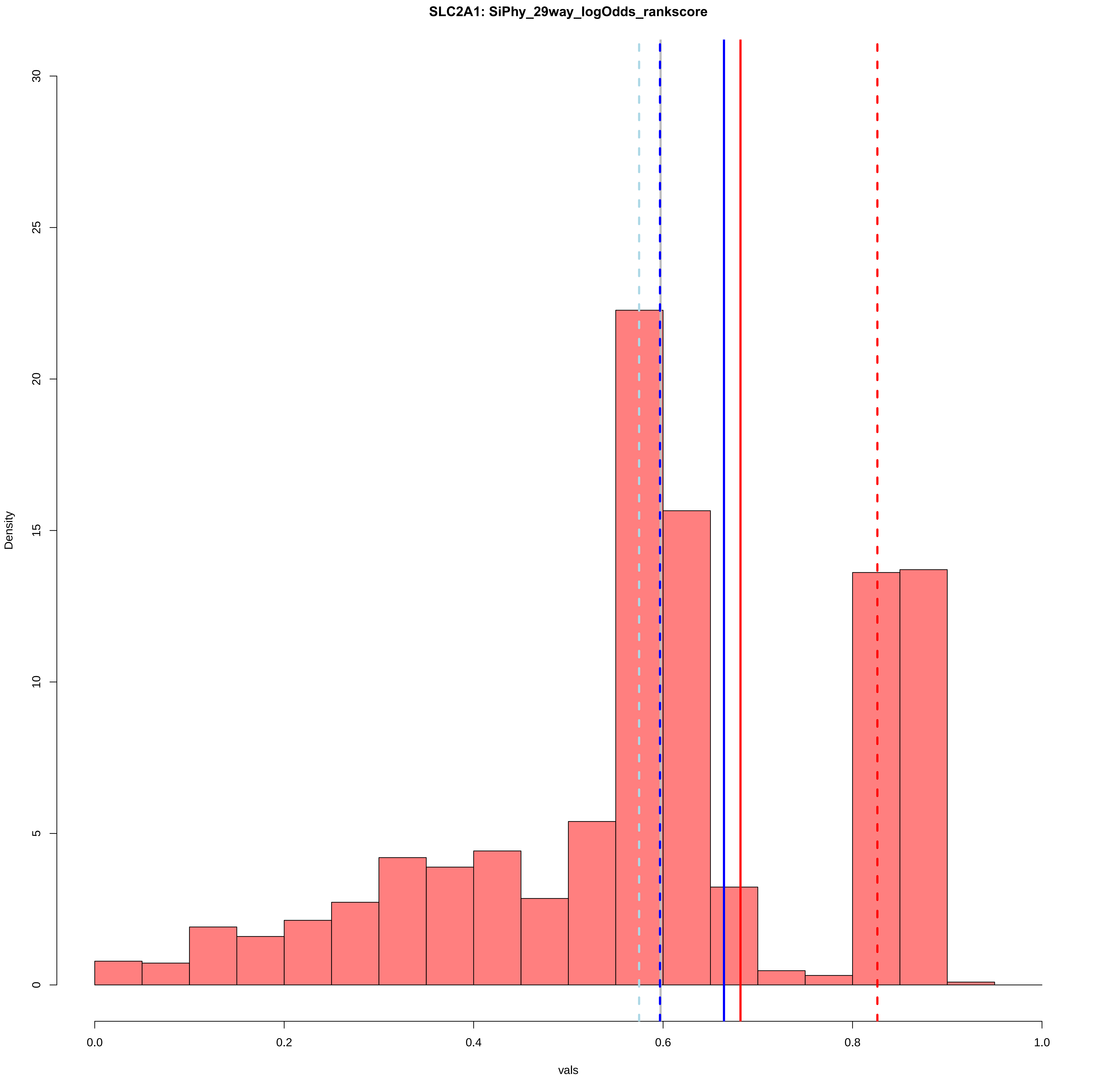

SLC2A1: SiPhy_29way_logOdds_rankscore
Density
vals
0
5
10
15
20
25
30
0.0
0.2
0.4
0.6
0.8
1.0

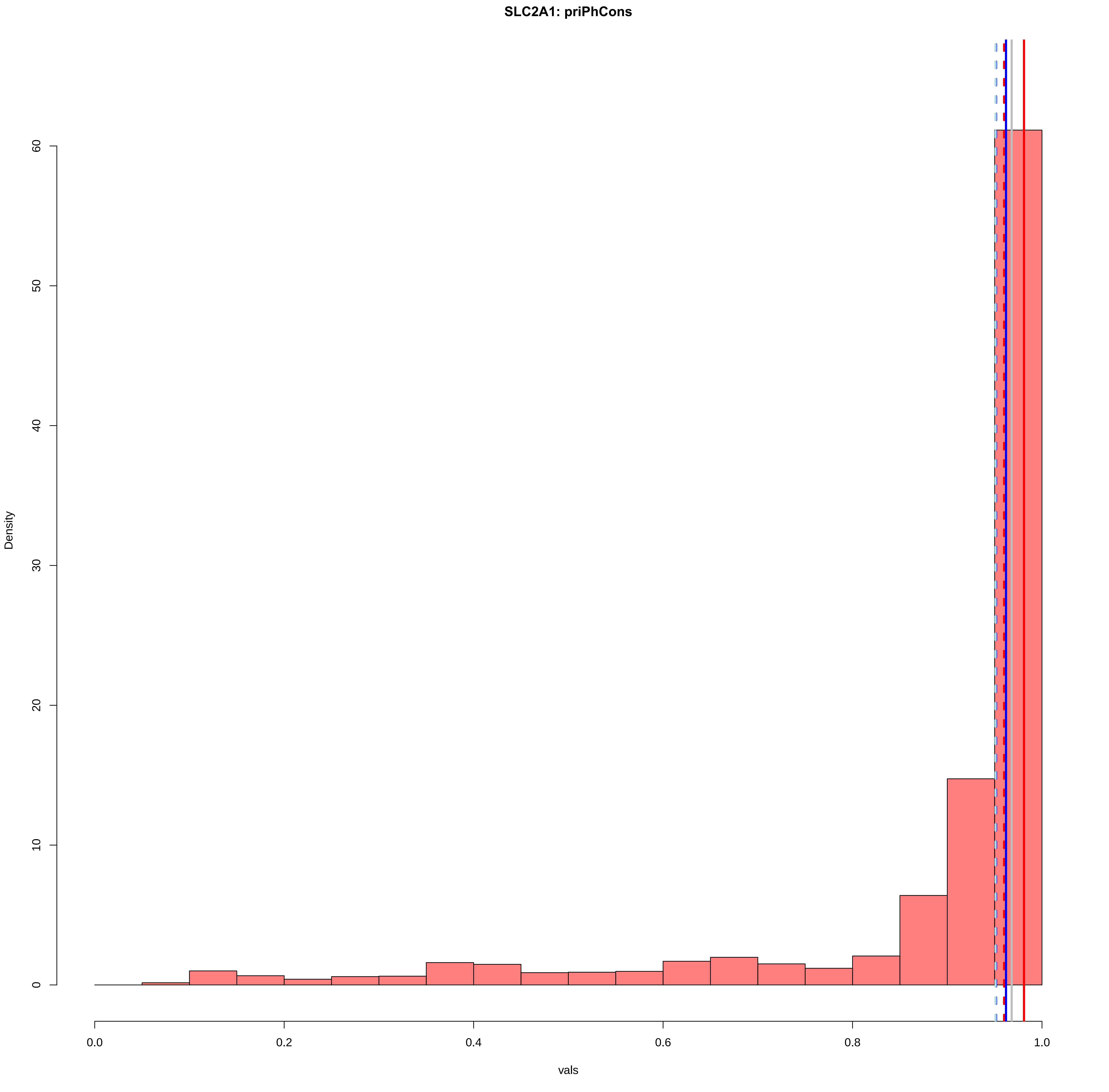

SLC2A1: priPhCons
Density
vals
0
10
20
30
40
50
60
0.0
0.2
0.4
0.6
0.8
1.0

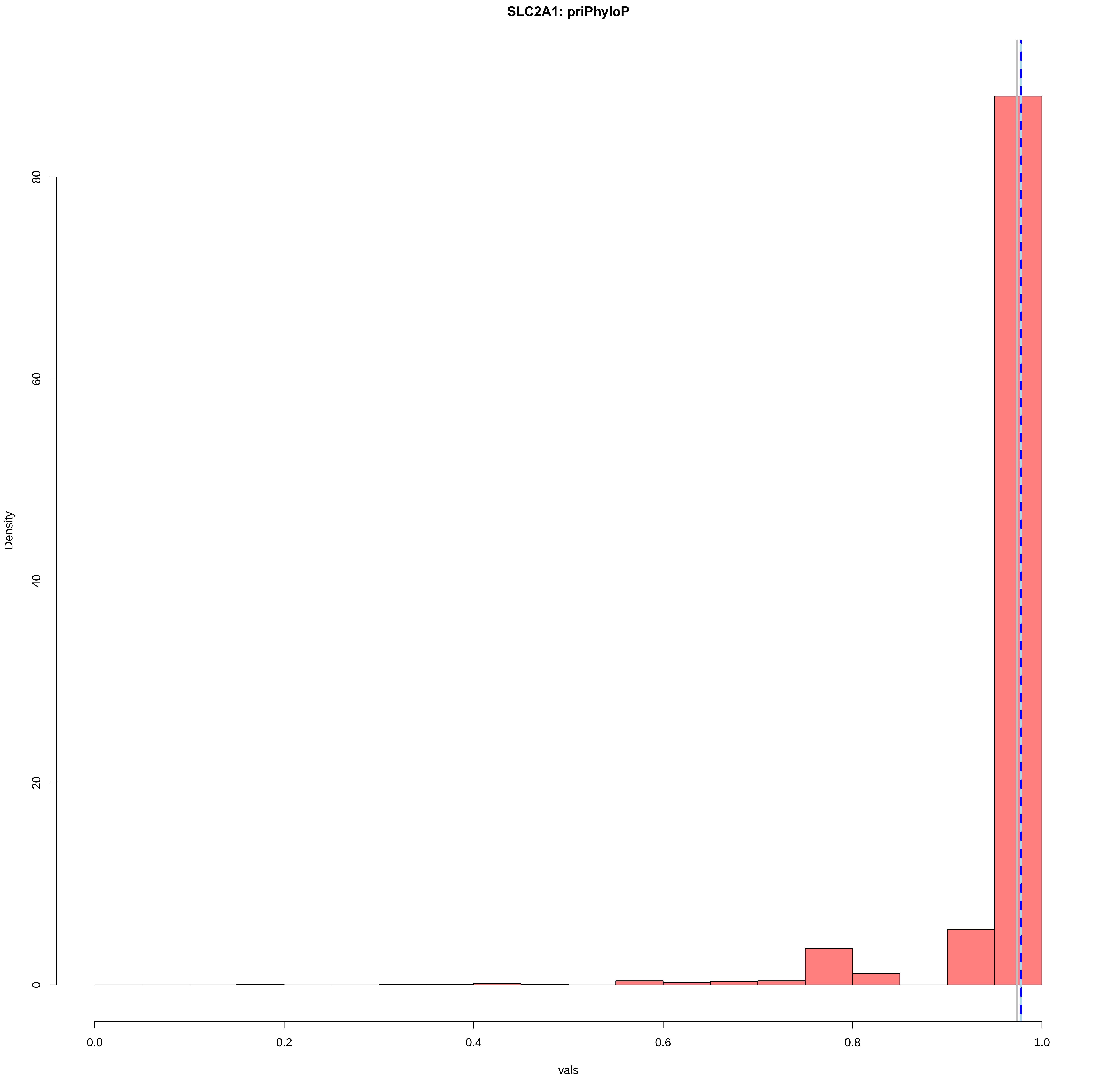
SLC2A1: priPhyloP
Density
vals
0
20
40
60
80
0.0
0.2
0.4
0.6
0.8
1.0

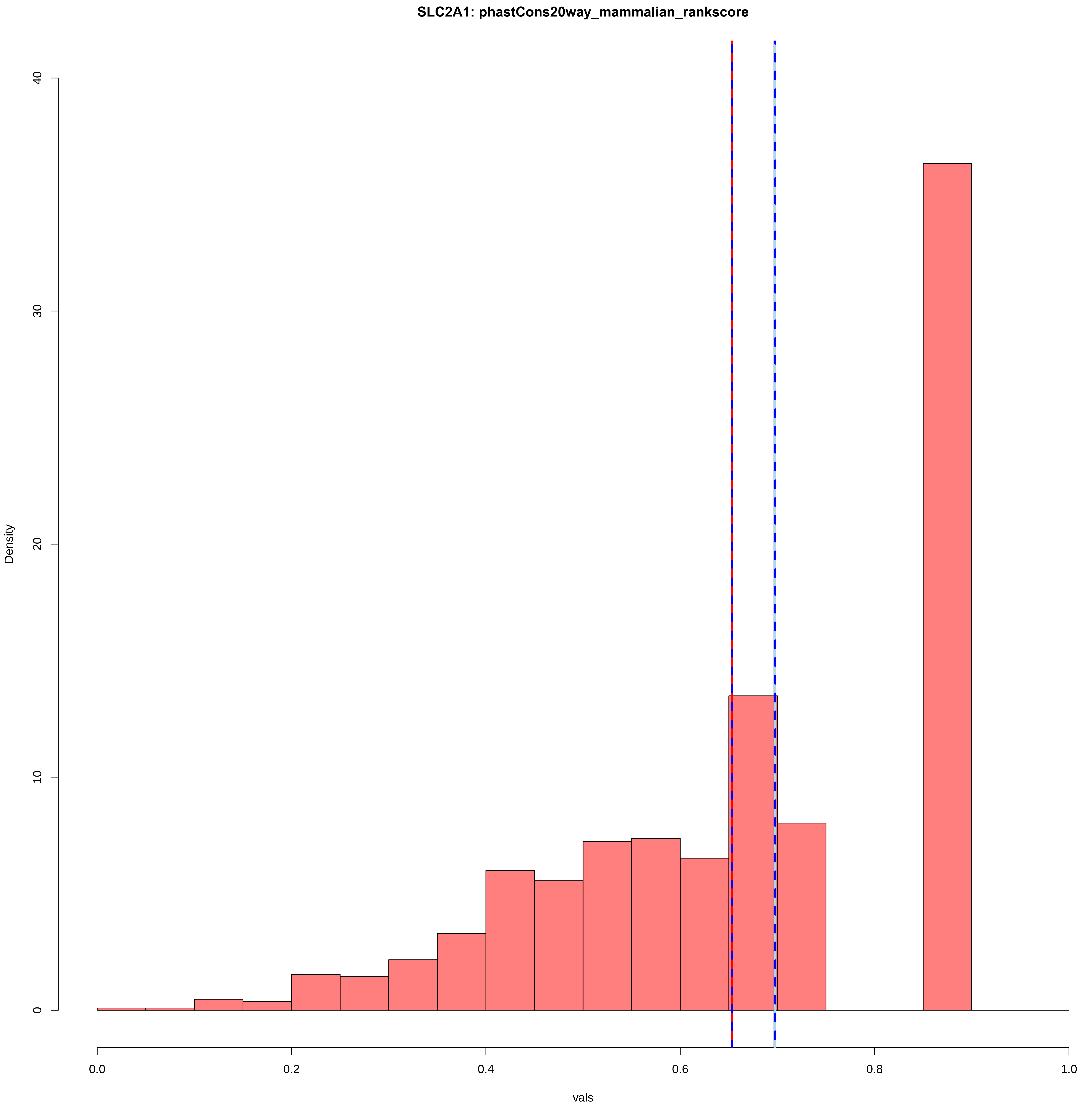
SLC2A1: phastCons20way_mammalian_rankscore
Density
vals
0
10
20
30
40
0.0
0.2
0.4
0.6
0.8
1.0

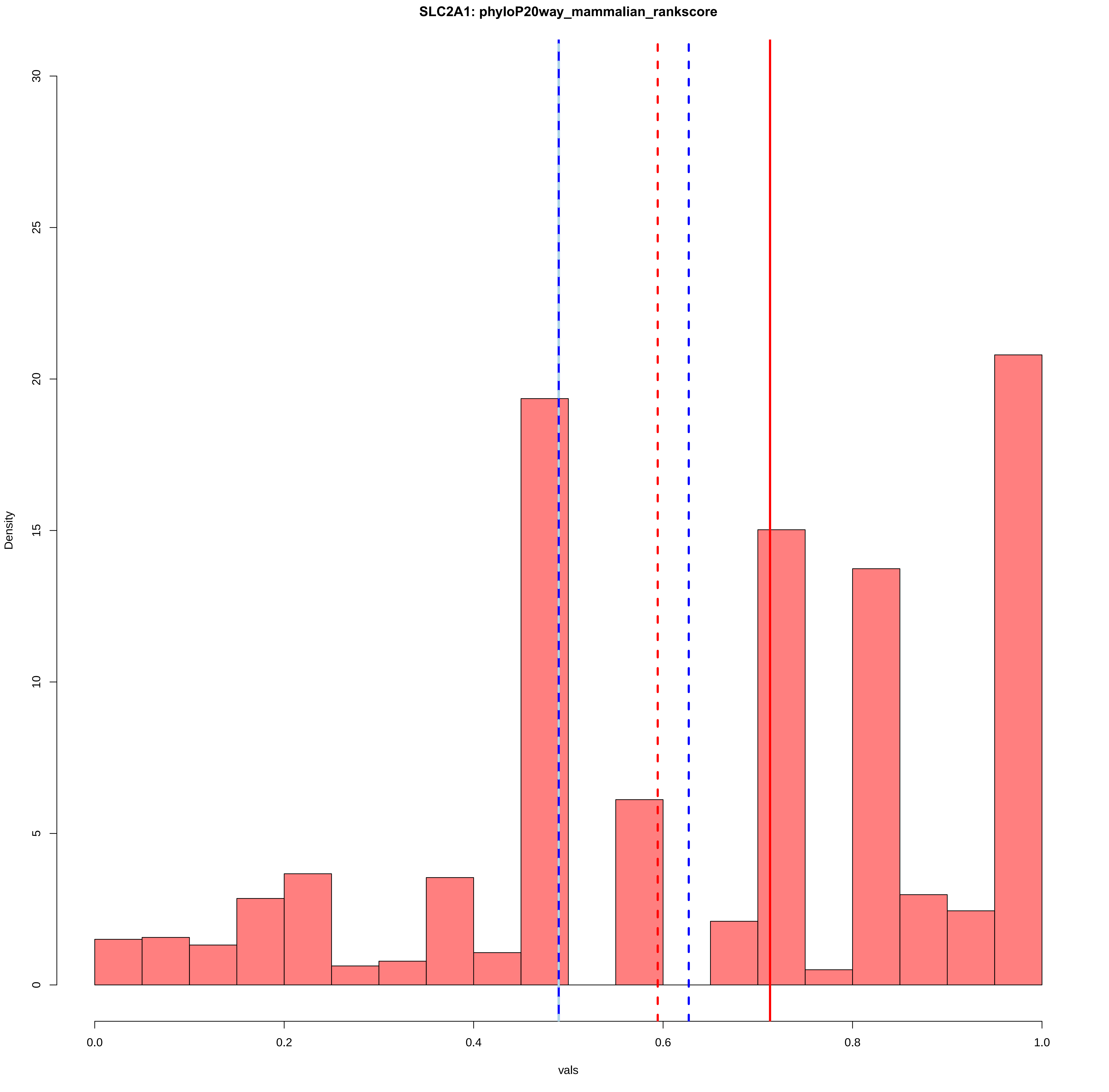

SLC2A1: phyloP20way_mammalian_rankscore
Density
vals
0
5
10
15
20
25
30
0.0
0.2
0.4
0.6
0.8
1.0

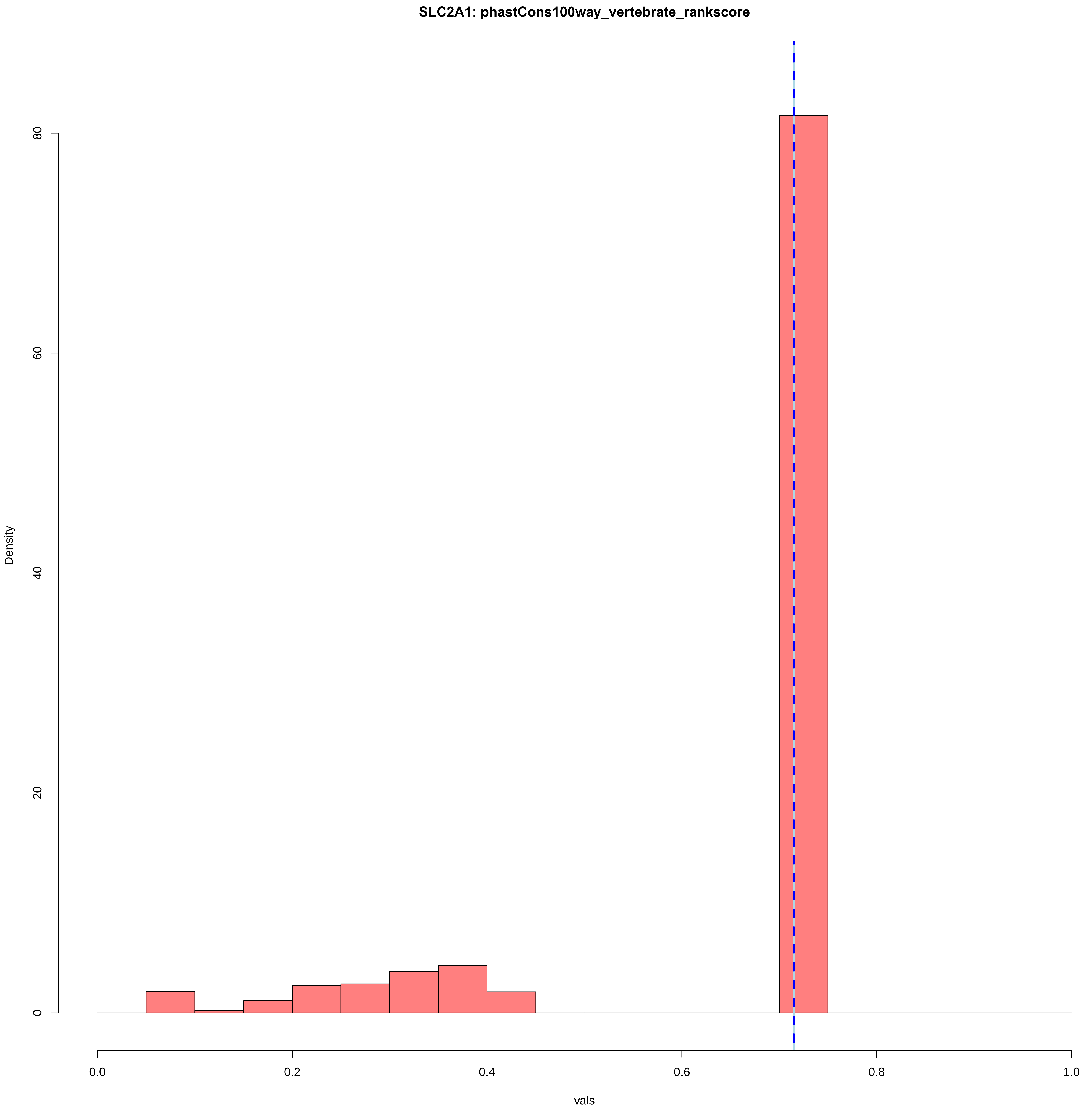
SLC2A1: phastCons100way_vertebrate_rankscore
Density
0
20
40
60
80
0.0
0.2
0.4
0.6
0.8
1.0
vals

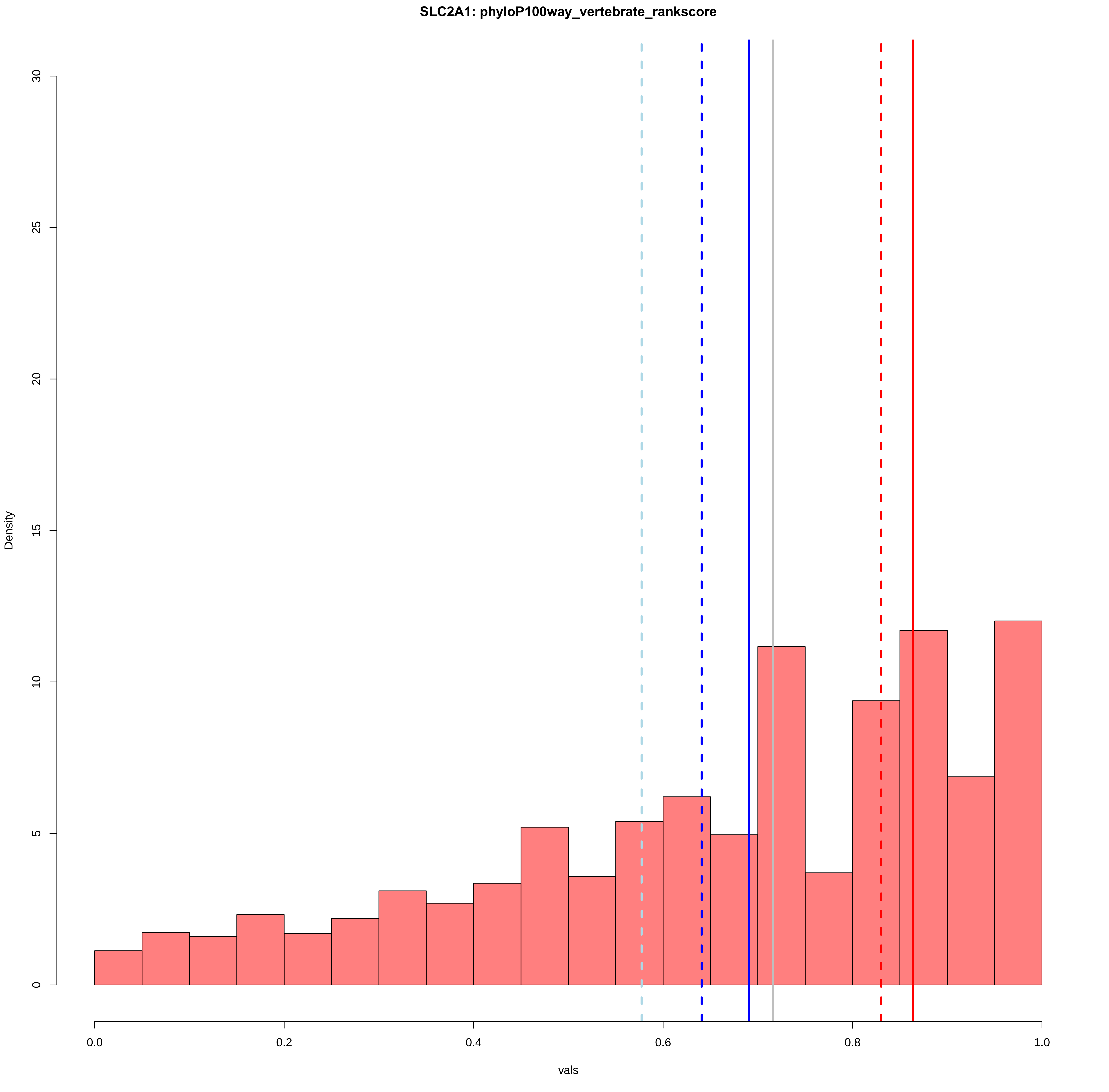
SLC2A1: phyloP100way_vertebrate_rankscore
Density
vals
0
5
10
15
20
25
30
0.0
0.2
0.4
0.6
0.8
1.0

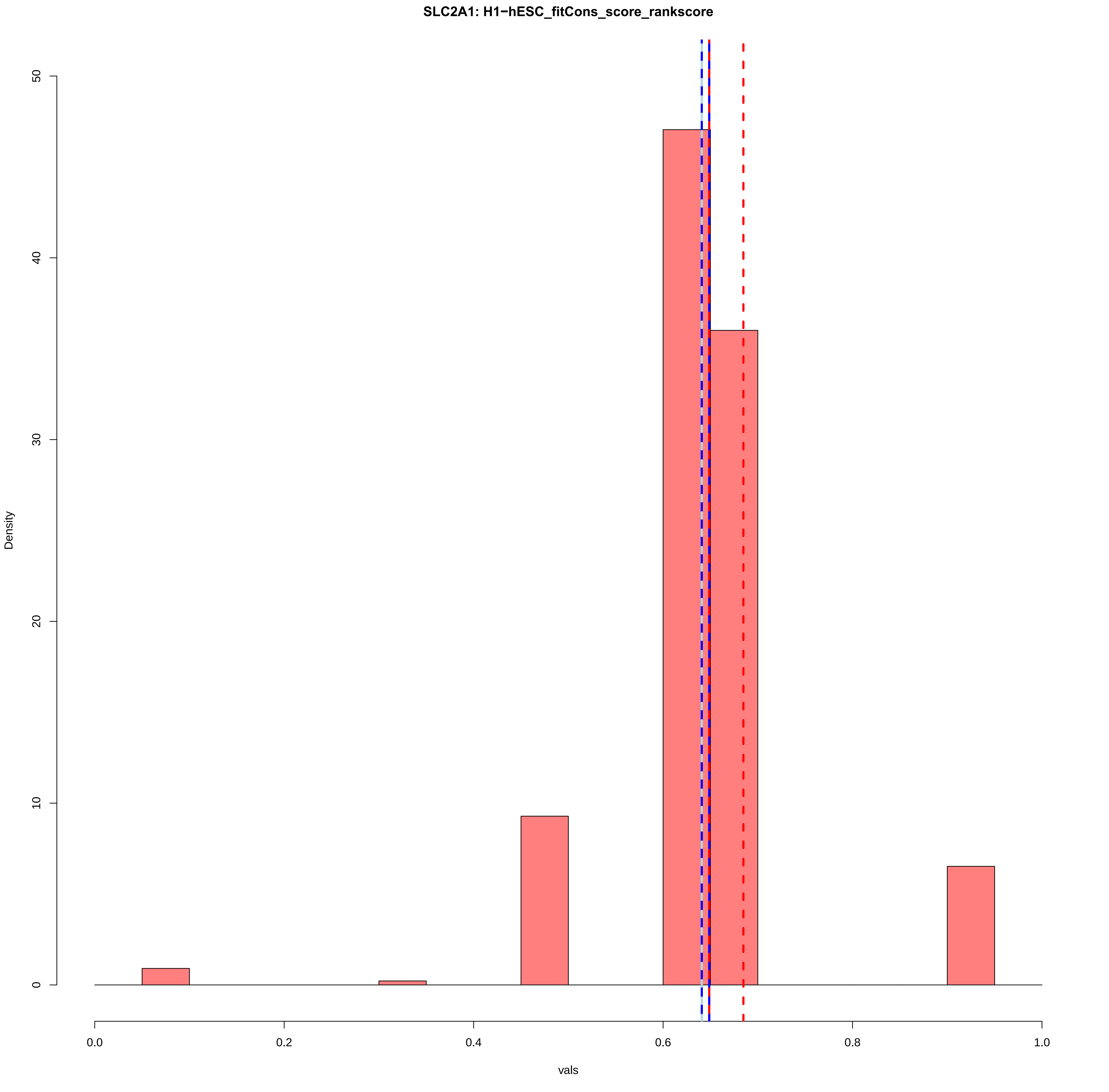
SLC2A1: H1−hESC_fitCons_score_rankscore
Density
vals
0
10
20
30
40
50
0.0
0.2
0.4
0.6
0.8
1.0

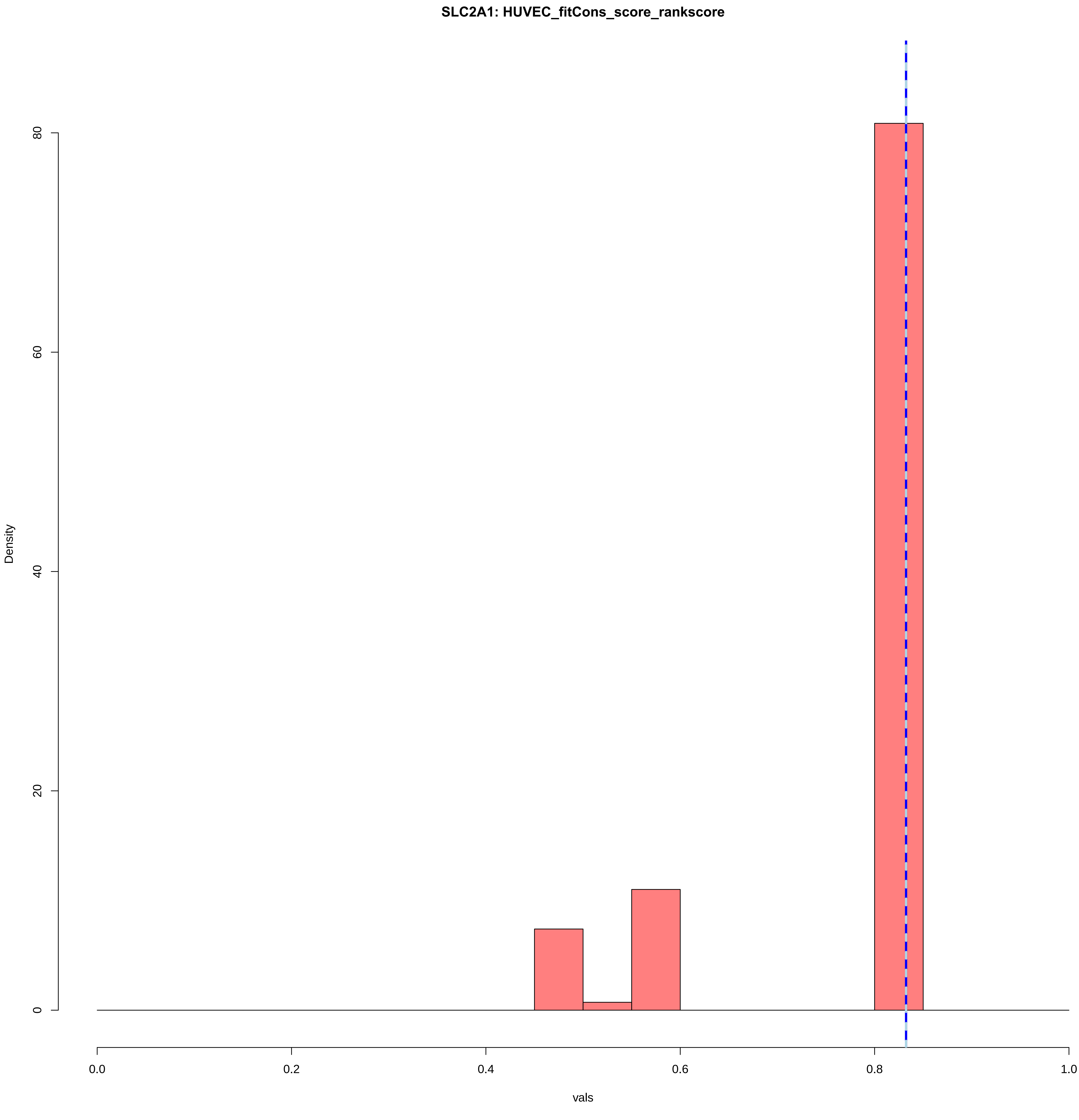

SLC2A1: HUVEC_fitCons_score_rankscore
Density
0
20
40
60
80
0.0
0.2
0.4
0.6
0.8
1.0
vals

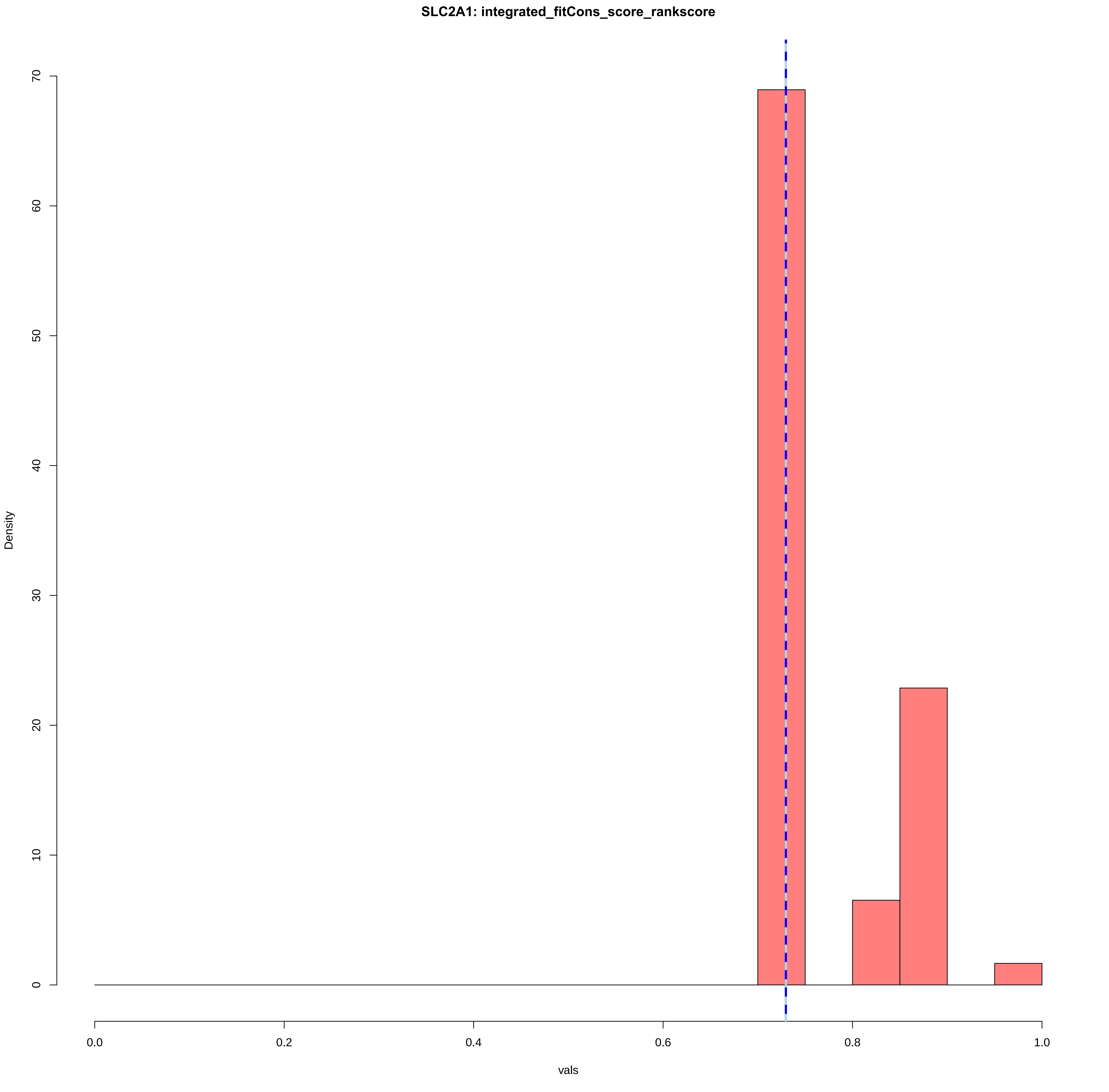

SLC2A1: integrated_fitCons_score_rankscore
Density
vals
0
10
20
30
40
50
60
70
0.0
0.2
0.4
0.6
0.8
1.0

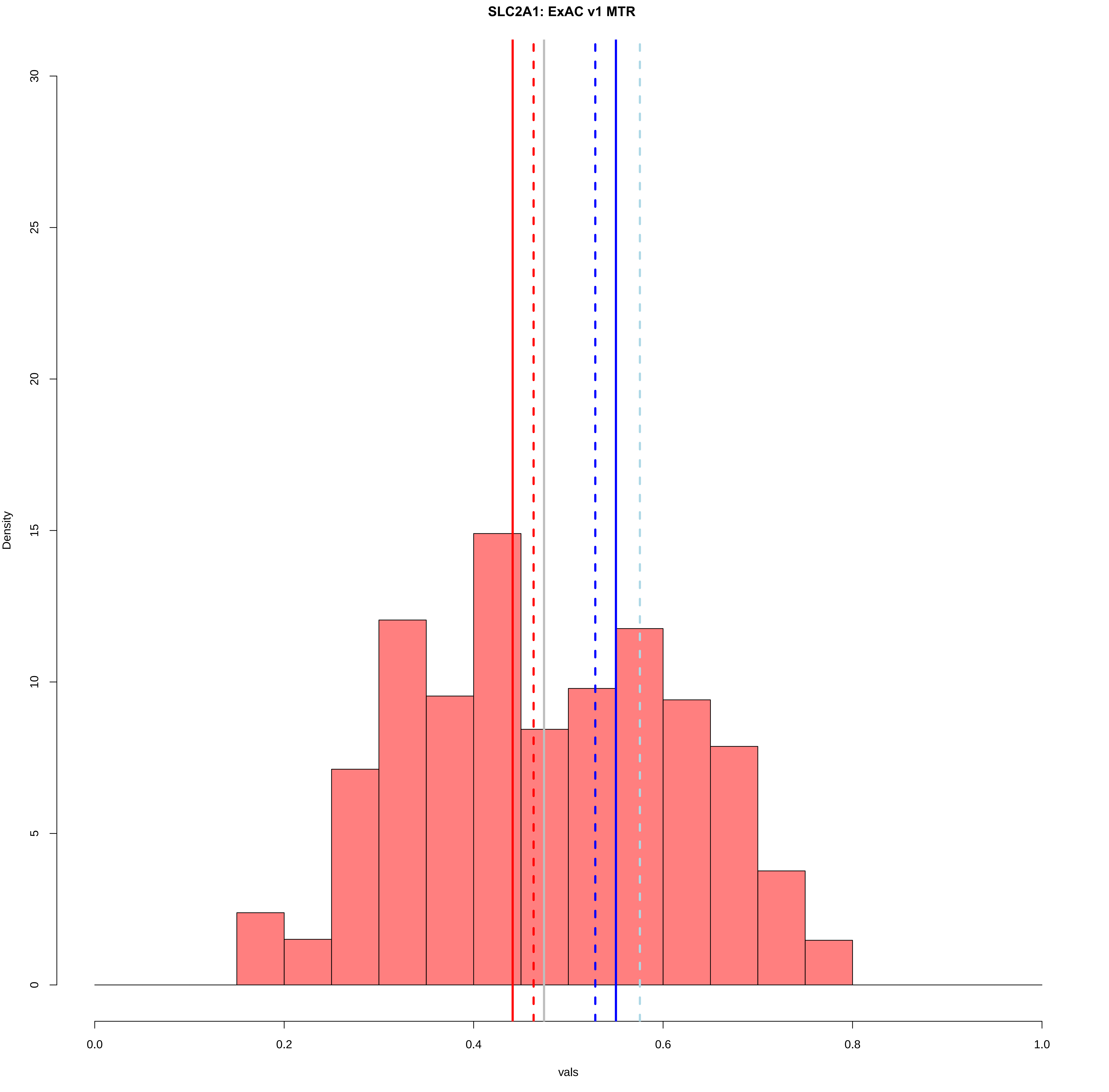

SLC2A1: ExAC v1 MTR
Density
vals
0
5
10
15
20
25
30
0.0
0.2
0.4
0.6
0.8
1.0

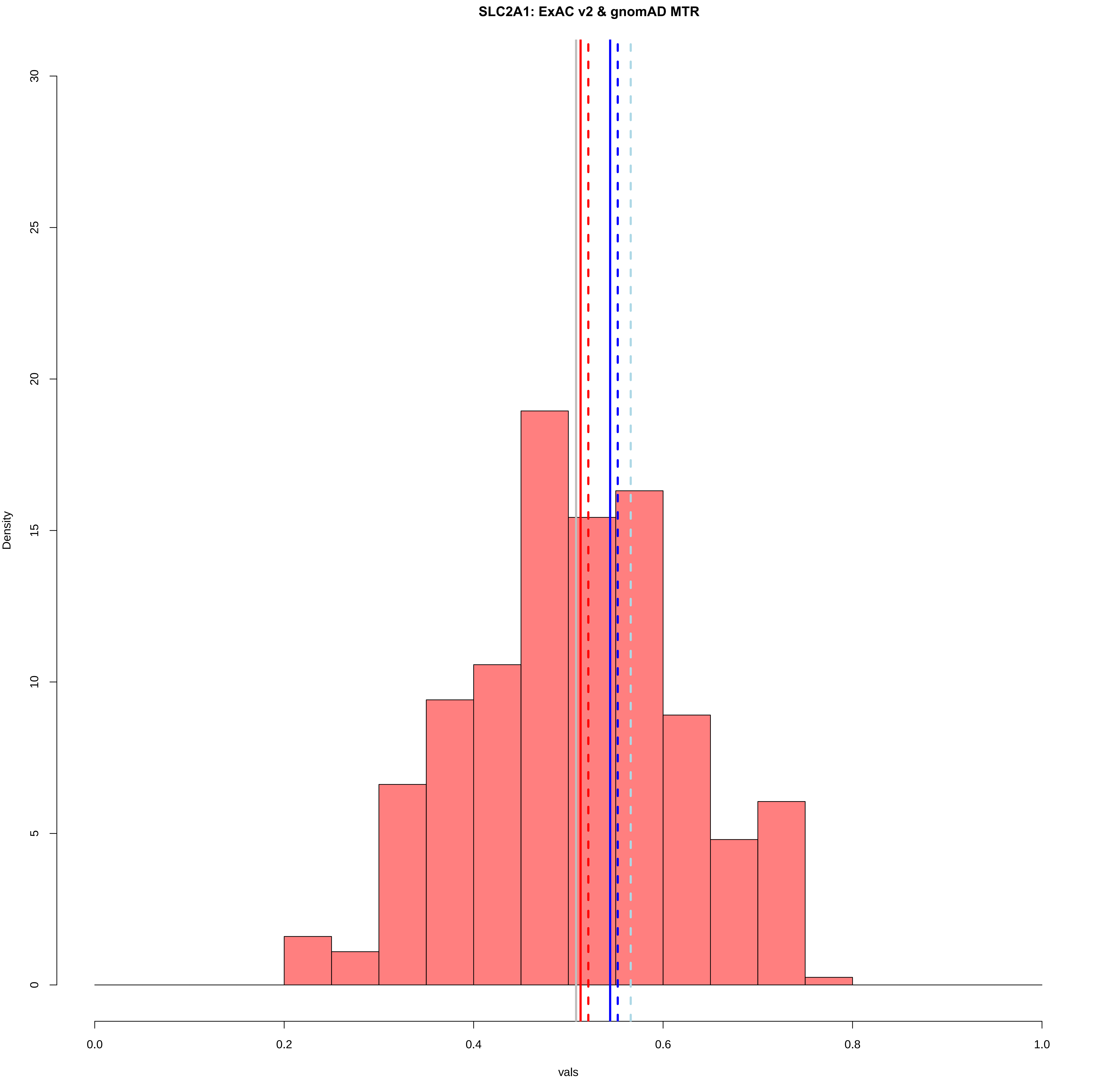

SLC2A1: ExAC v2 & gnomAD MTR
Density
vals
0
5
10
15
20
25
30
0.0
0.2
0.4
0.6
0.8
1.0

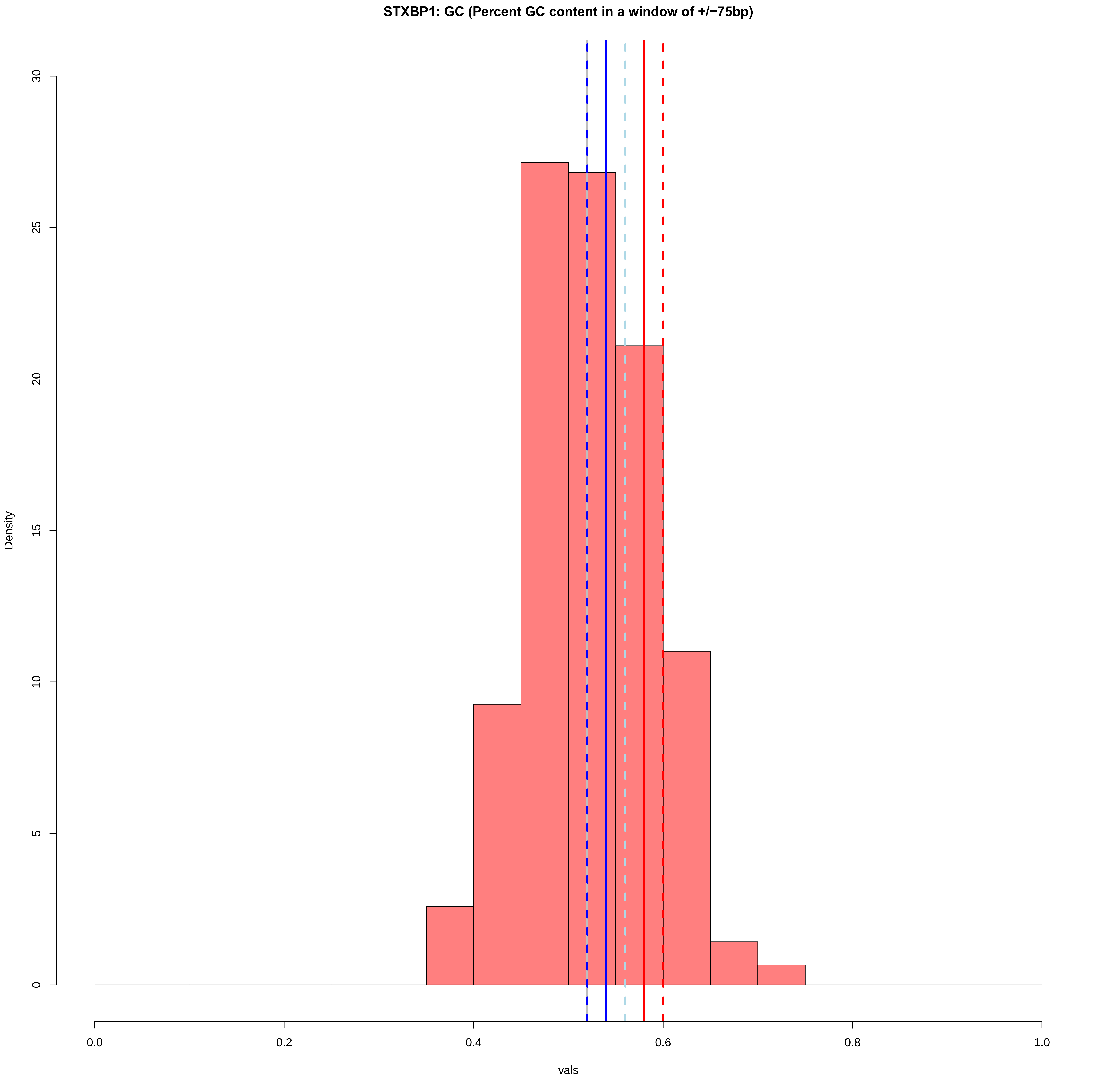

STXBP1: GC (Percent GC content in a window of +/−75bp)
Density
0
5
10
15
20
25
30
0.0
0.2
0.4
0.6
0.8
1.0
vals

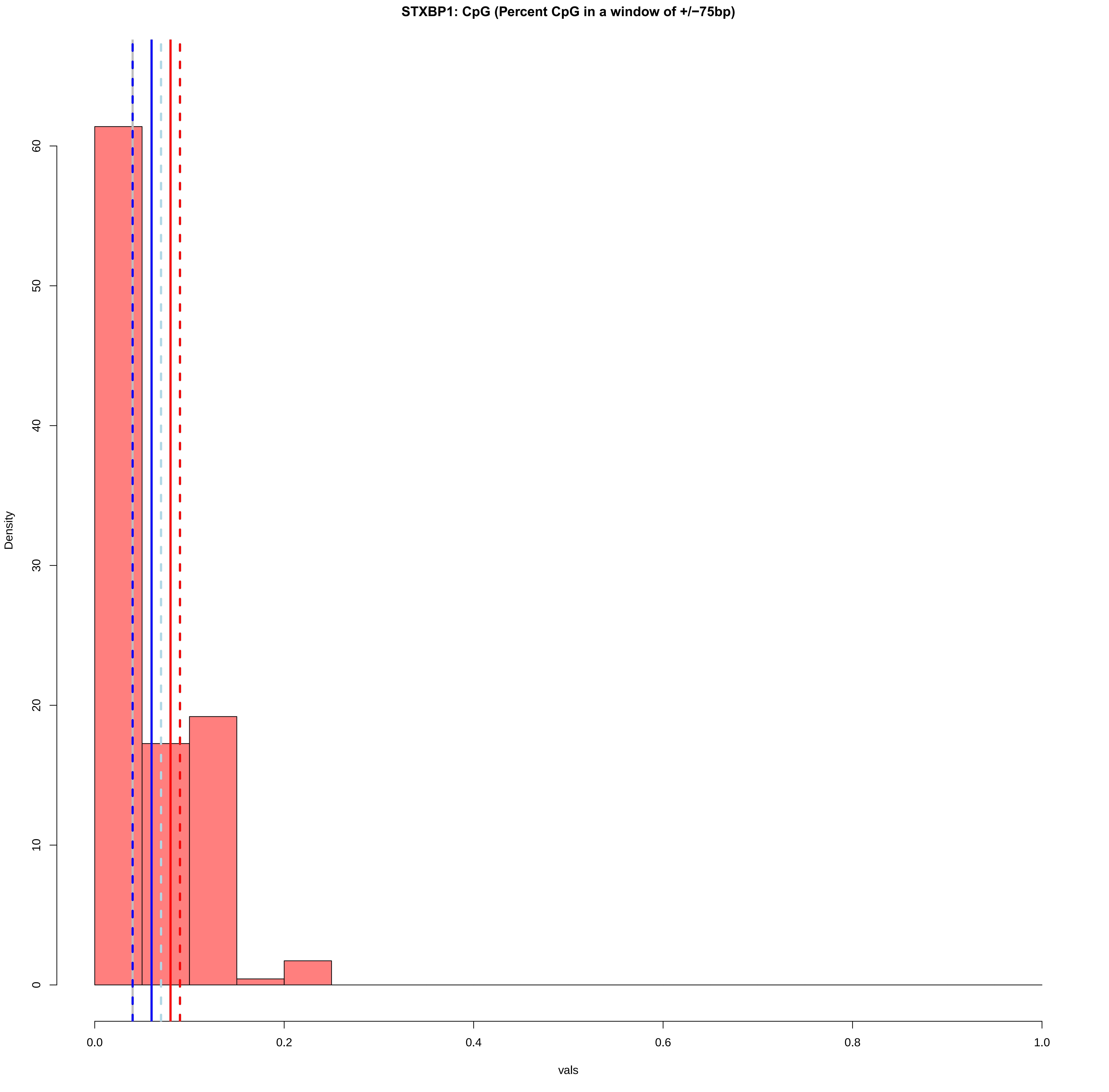

STXBP1: CpG (Percent CpG in a window of +/−75bp)
Density
vals
0
10
20
30
40
50
60
0.0
0.2
0.4
0.6
0.8
1.0

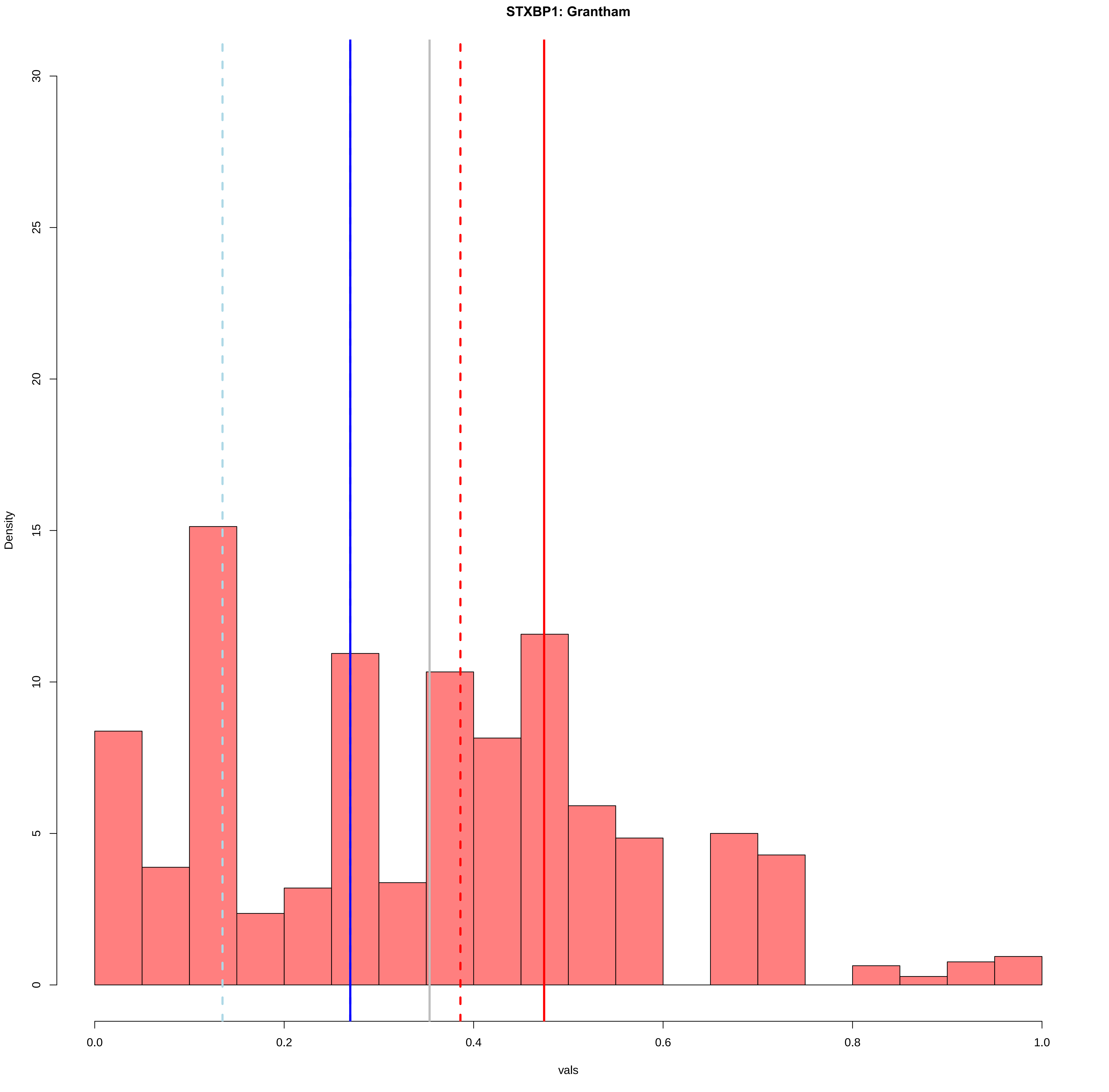

STXBP1: Grantham
Density
0
5
10
15
20
25
30
0.0
0.2
0.4
0.6
0.8
1.0
vals

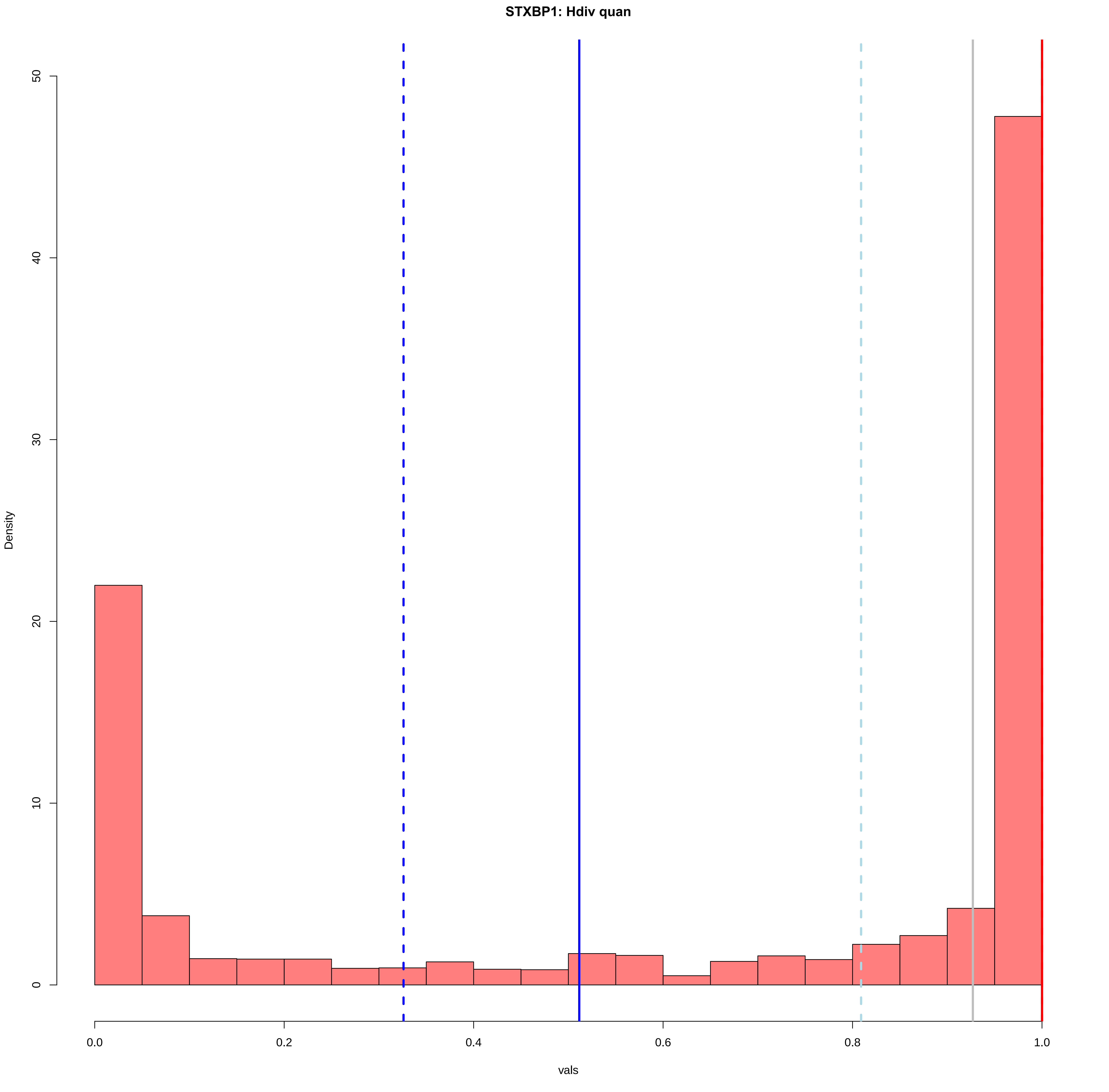
STXBP1: Hdiv quan
Density
vals
0
10
20
30
40
50
0.0
0.2
0.4
0.6
0.8
1.0

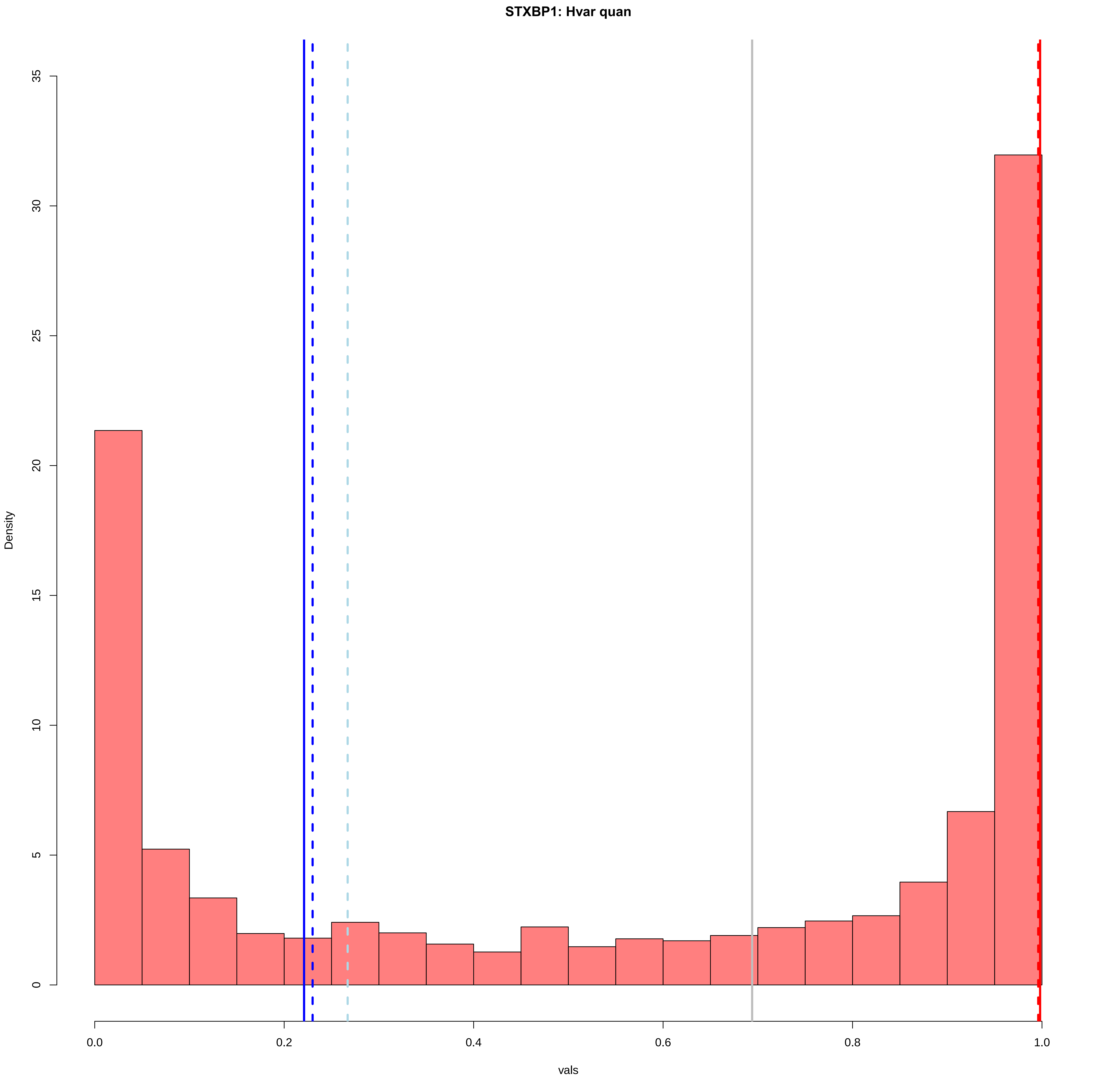
STXBP1: Hvar quan
Density
vals
0
5
10
15
20
25
30
35
0.0
0.2
0.4
0.6
0.8
1.0

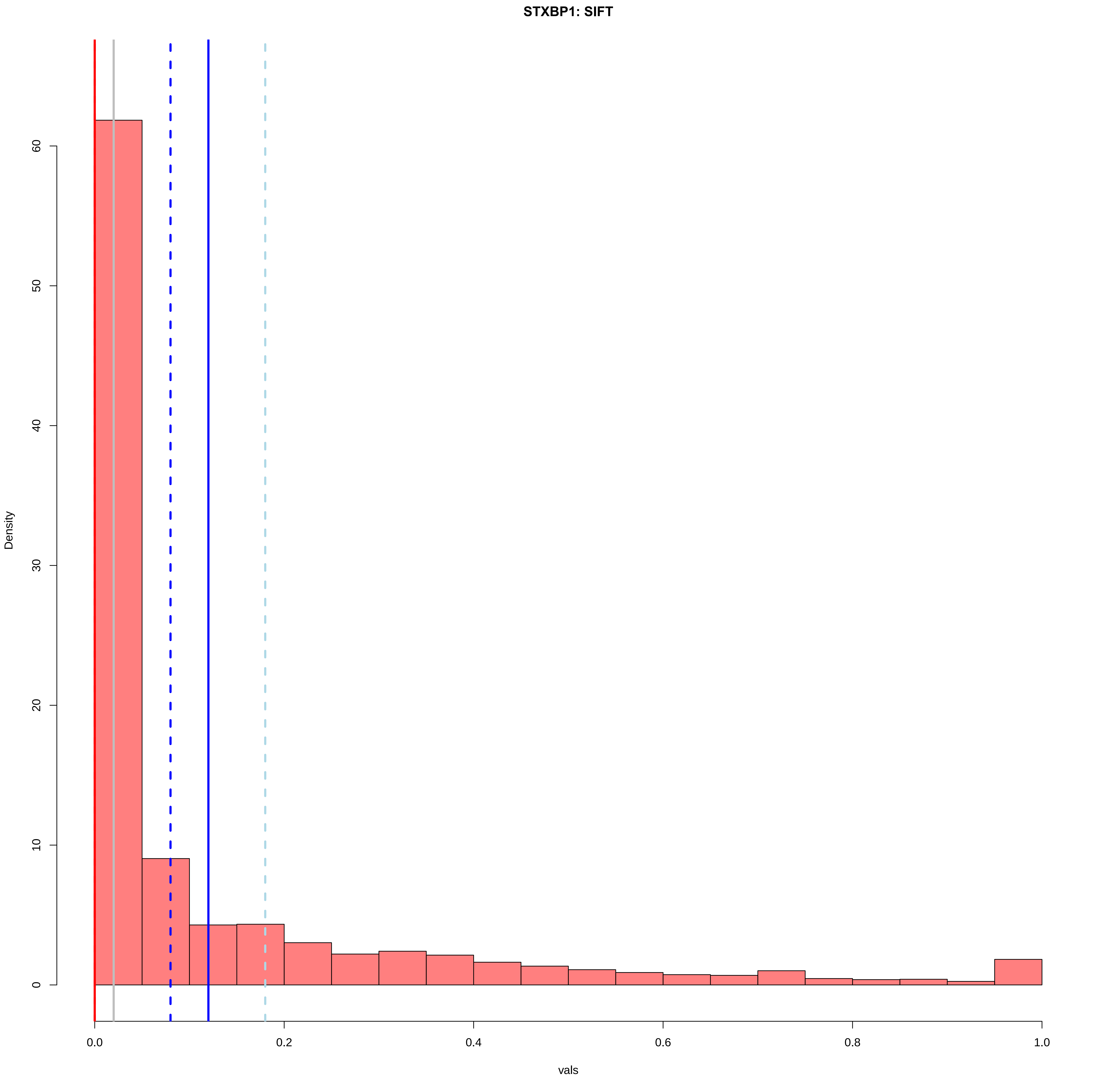

STXBP1: SIFT
Density
vals
0
10
20
30
40
50
60
0.0
0.2
0.4
0.6
0.8
1.0

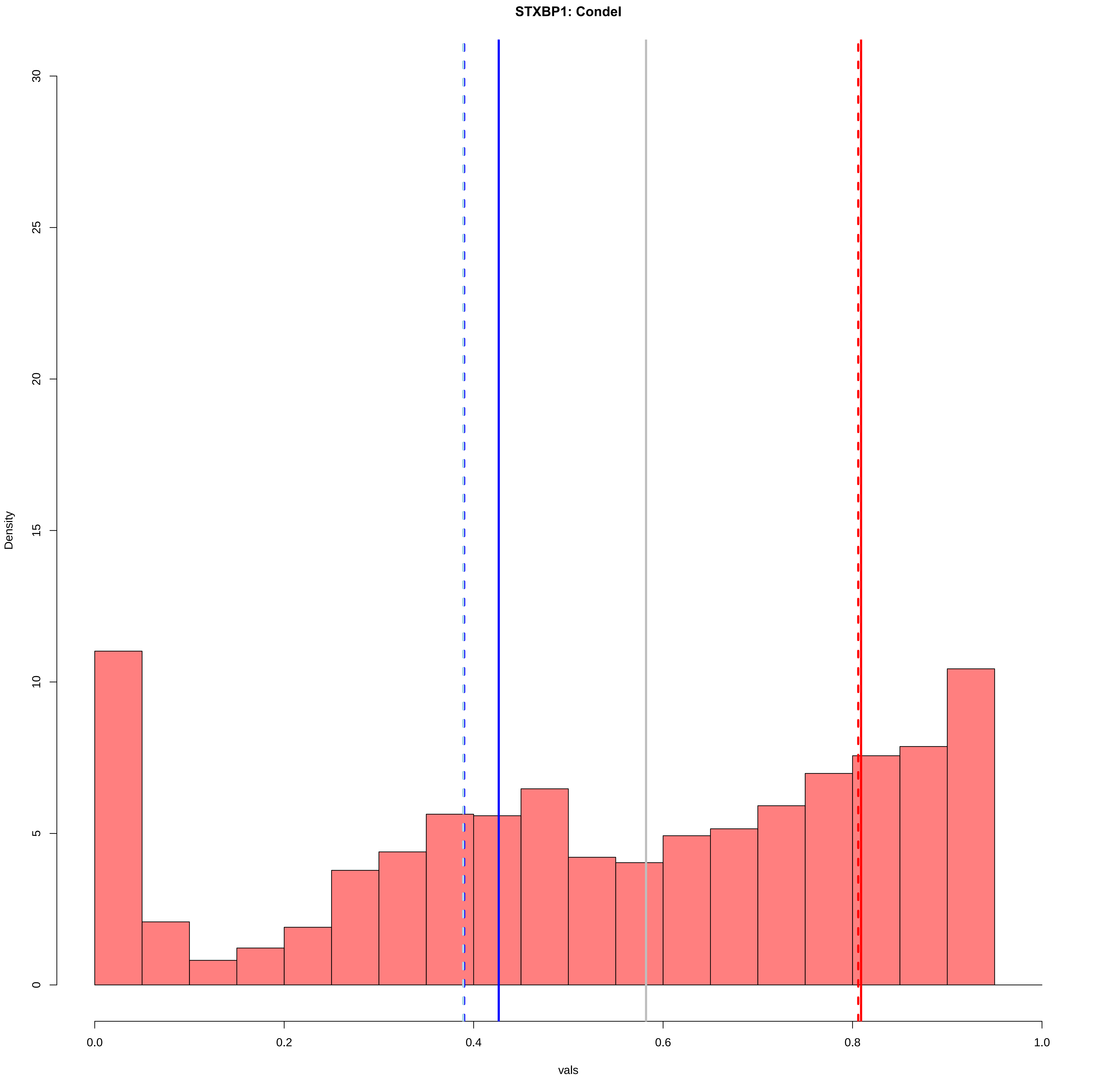
STXBP1: Condel
Density
vals
0
5
10
15
20
25
30
0.0
0.2
0.4
0.6
0.8
1.0

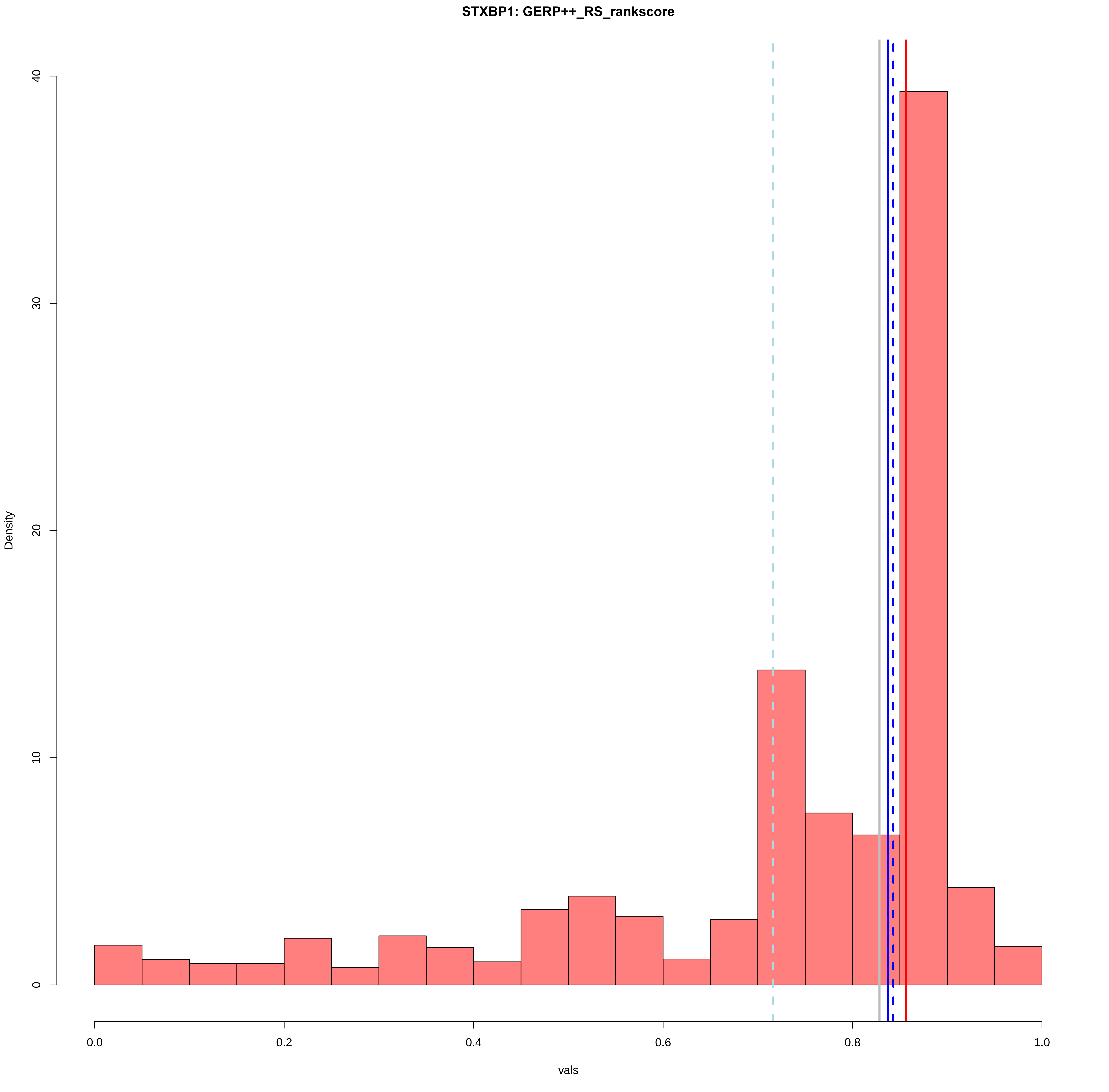

STXBP1: GERP++_RS_rankscore
Density
vals
0
10
20
30
40
0.0
0.2
0.4
0.6
0.8
1.0

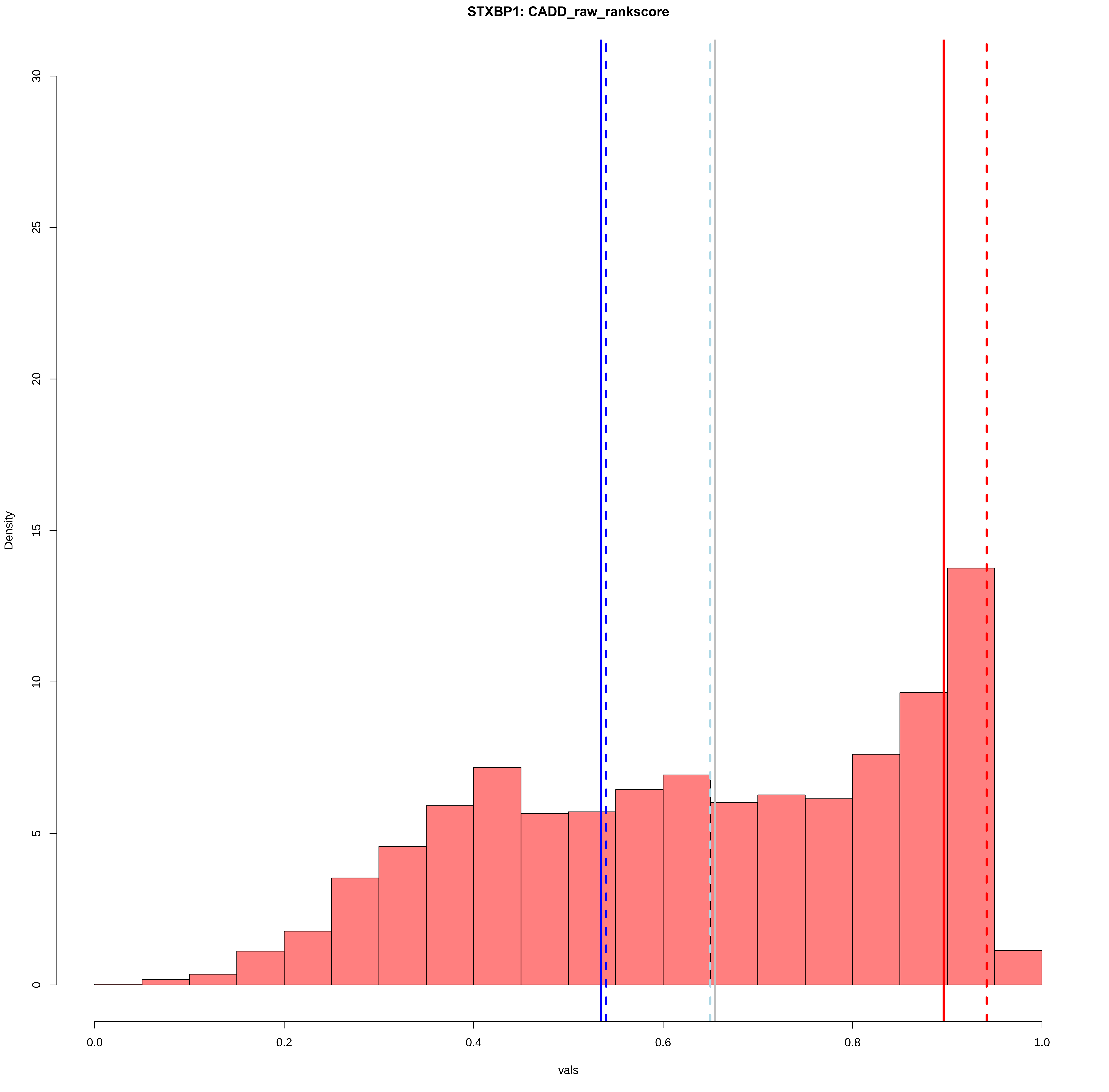

STXBP1: CADD_raw_rankscore
Density
vals
0
5
10
15
20
25
30
0.0
0.2
0.4
0.6
0.8
1.0

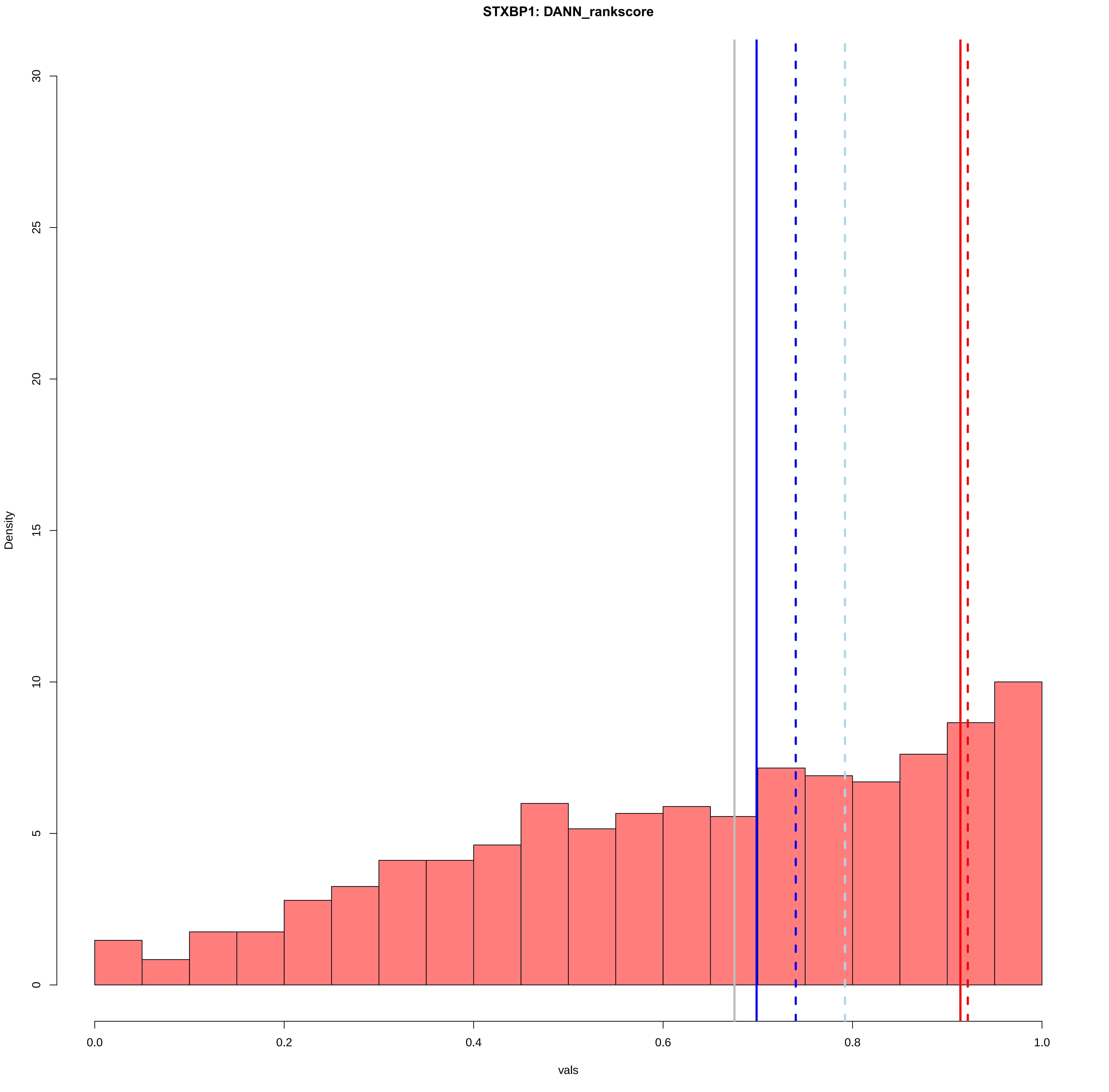

STXBP1: DANN_rankscore
Density
vals
0
5
10
15
20
25
30
0.0
0.2
0.4
0.6
0.8
1.0

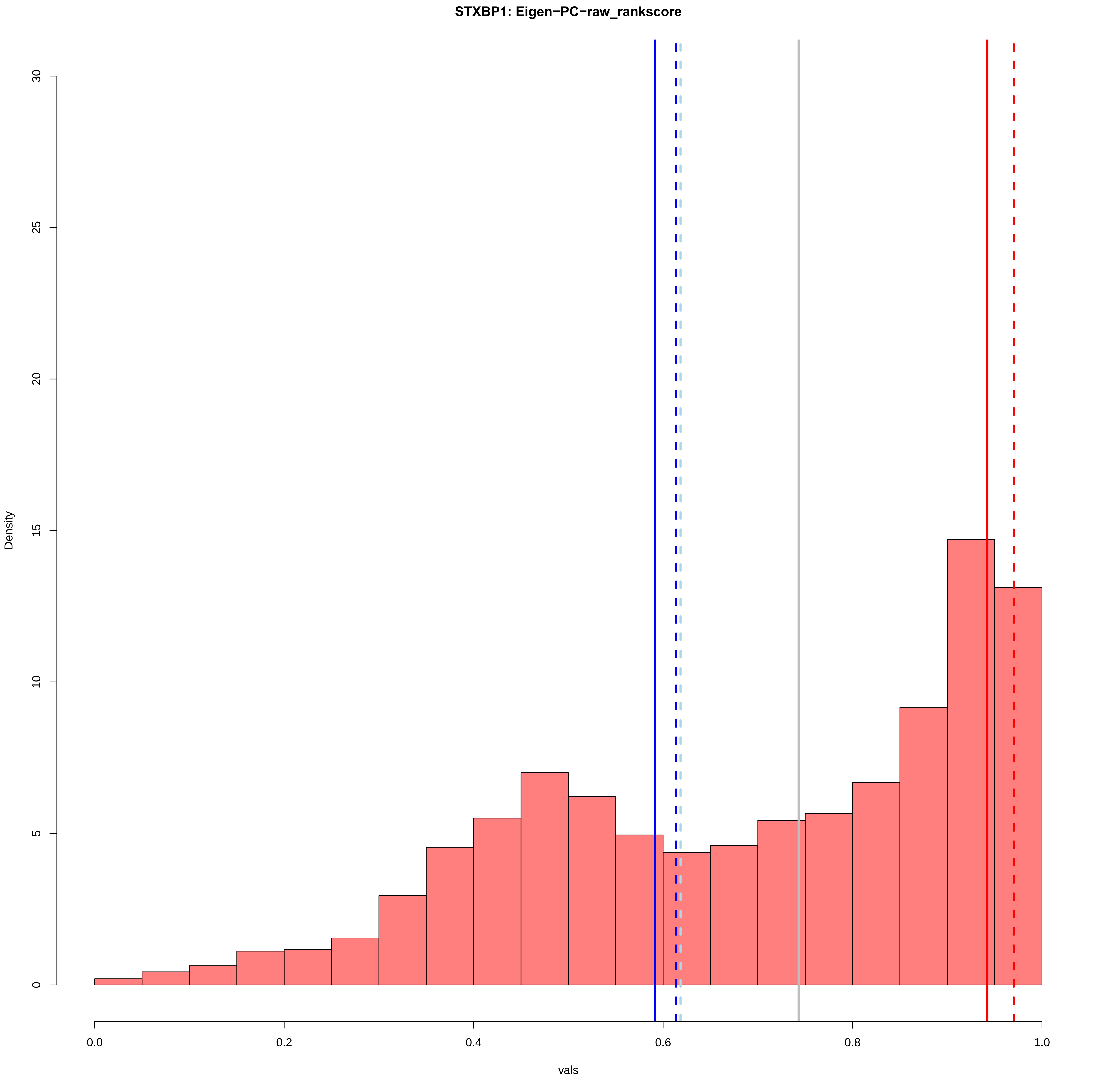

STXBP1: Eigen−PC−raw_rankscore
Density
vals
0
5
10
15
20
25
30
0.0
0.2
0.4
0.6
0.8
1.0

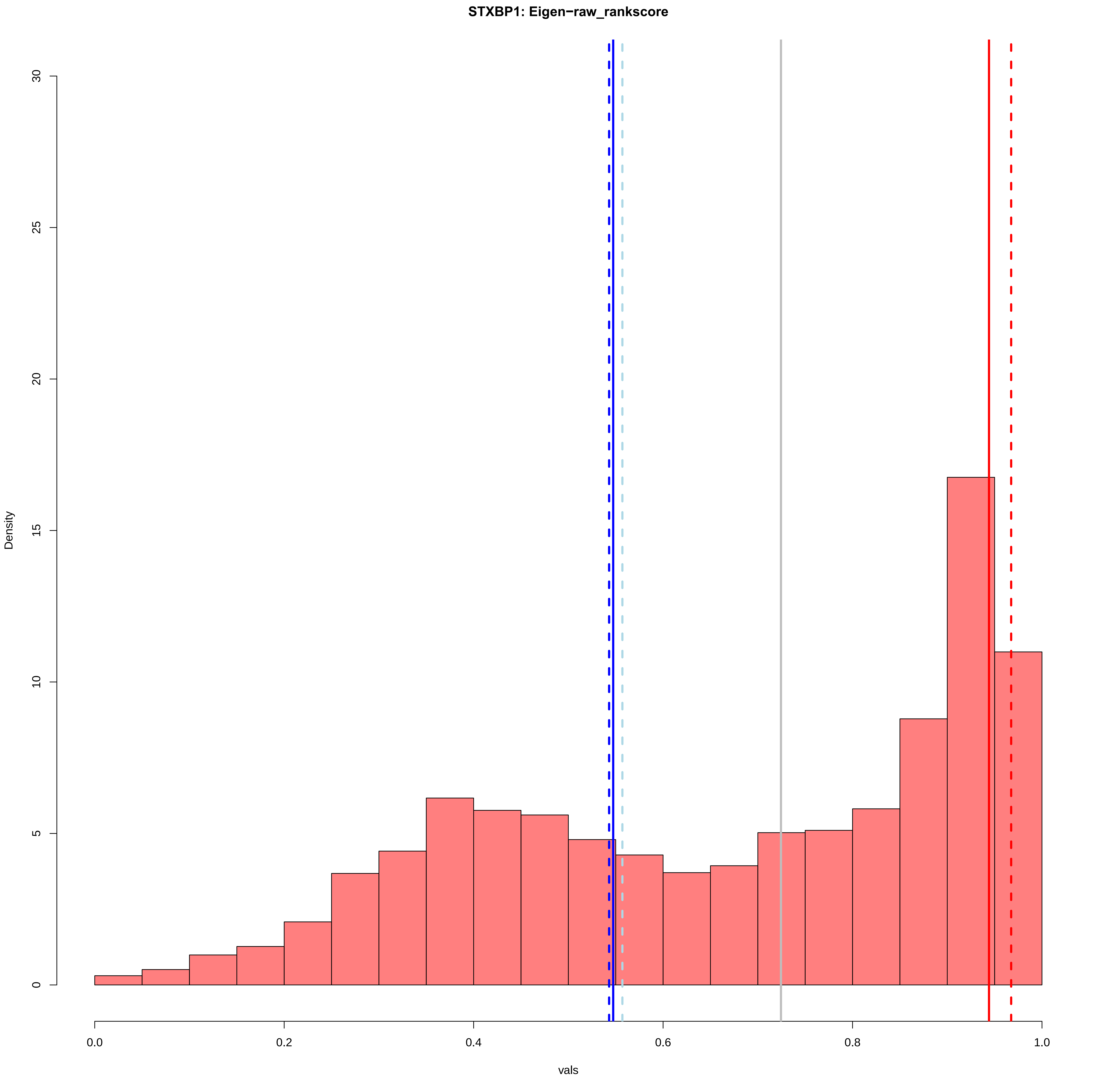
STXBP1: Eigen−raw_rankscore
Density
vals
0
5
10
15
20
25
30
0.0
0.2
0.4
0.6
0.8
1.0

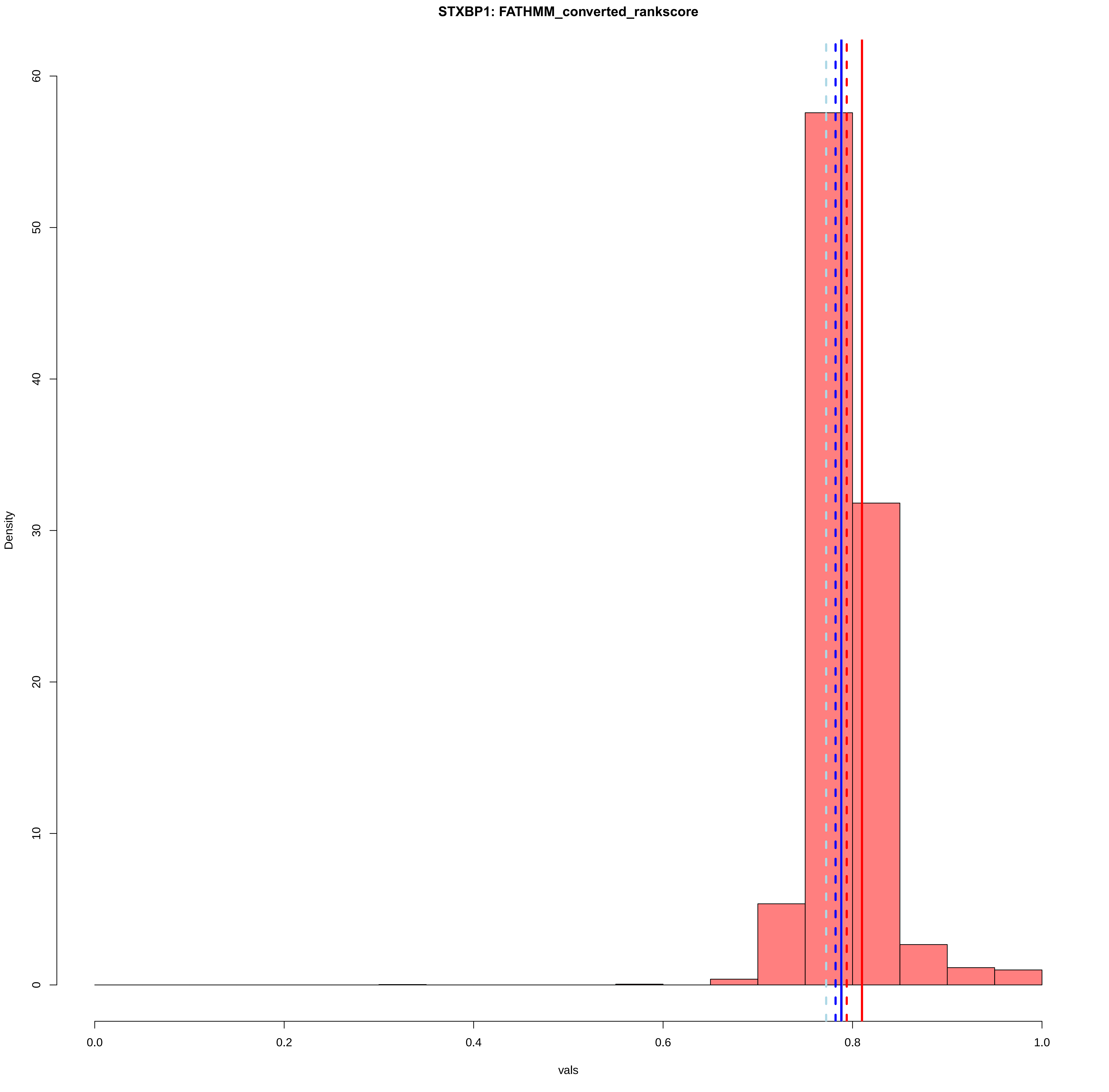

STXBP1: FATHMM_converted_rankscore
Density
vals
0
10
20
30
40
50
60
0.0
0.2
0.4
0.6
0.8
1.0

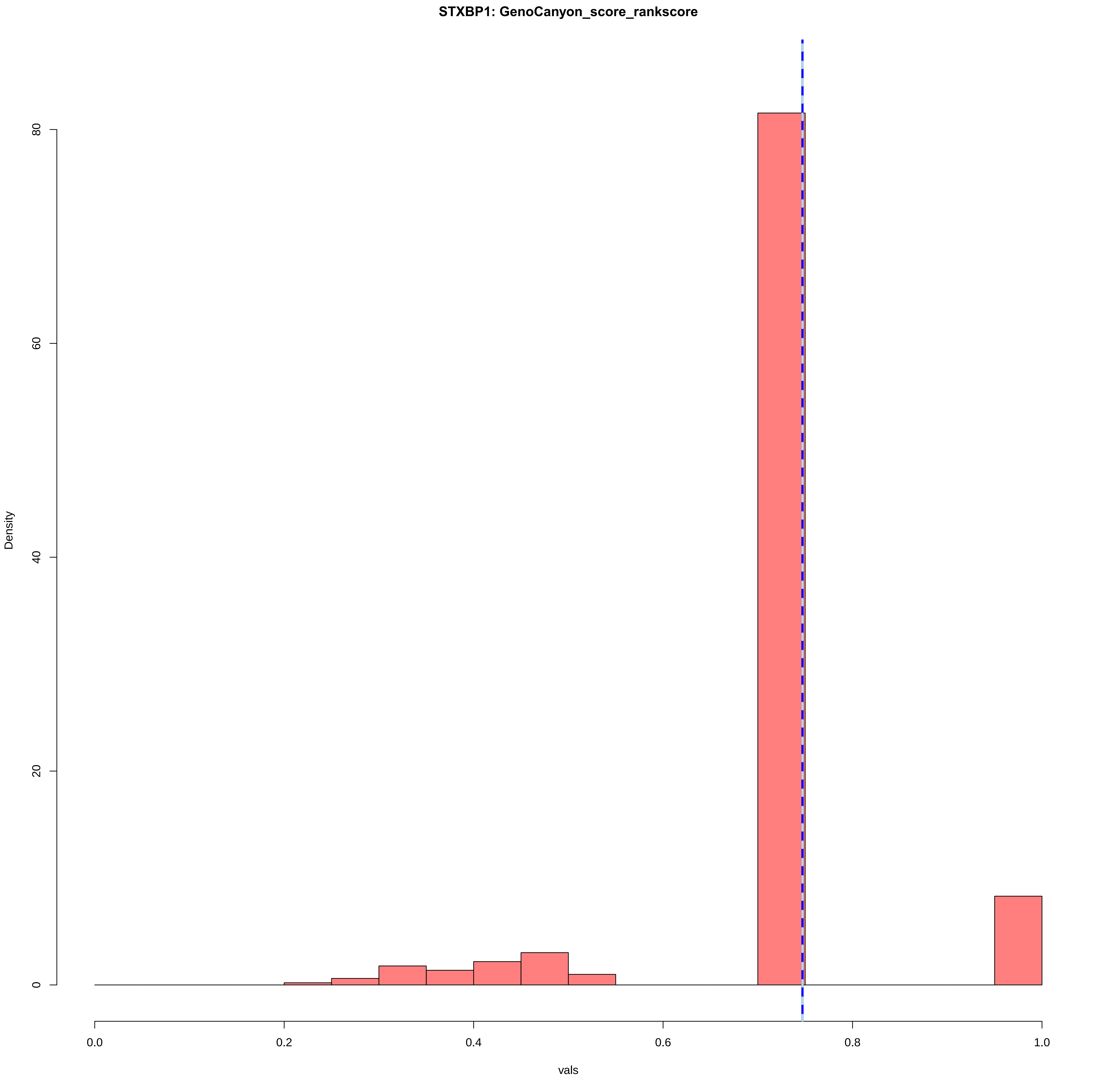
STXBP1: GenoCanyon_score_rankscore
Density
vals
0
20
40
60
80
0.0
0.2
0.4
0.6
0.8
1.0

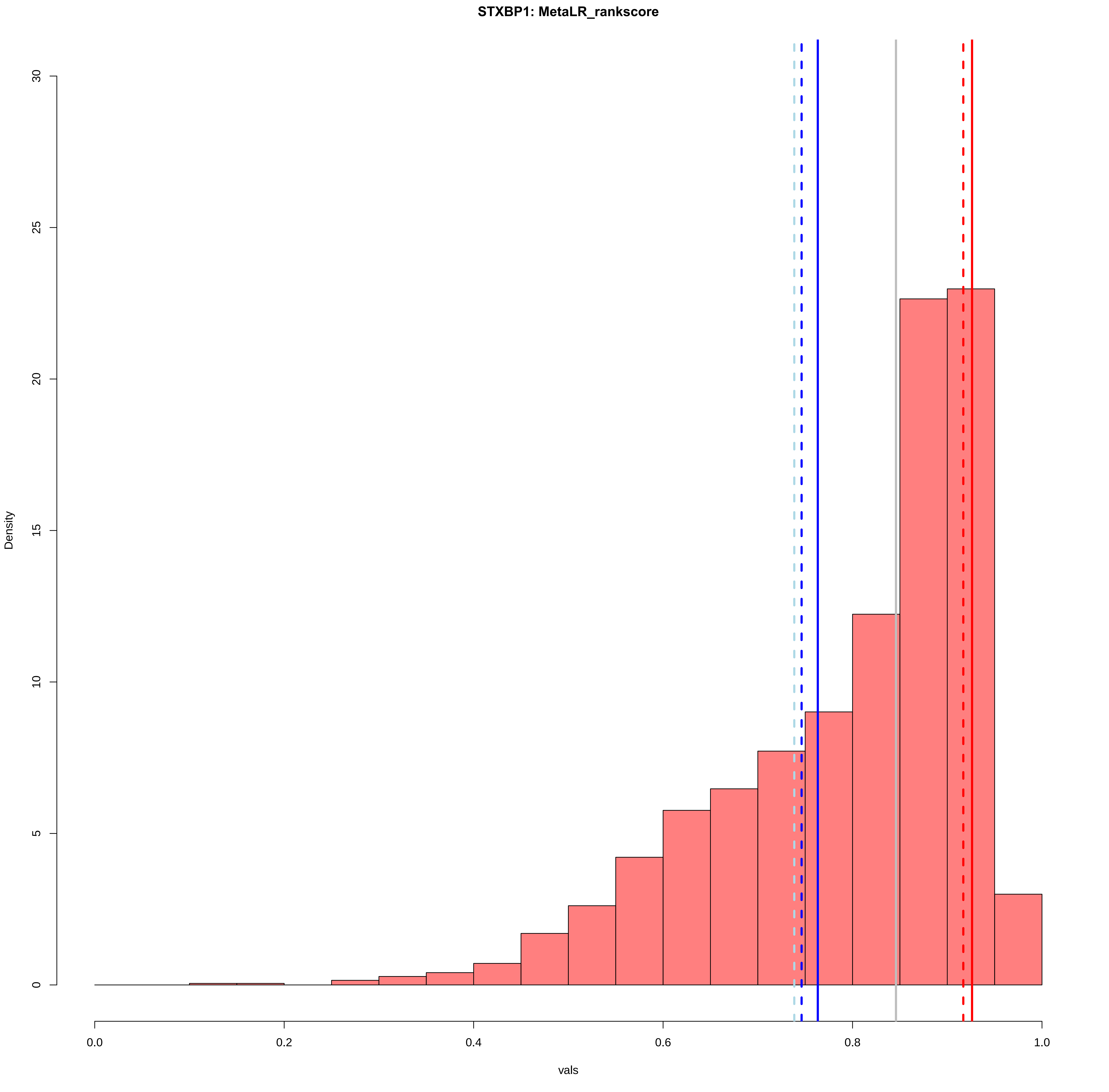

STXBP1: MetaLR_rankscore
Density
vals
0
5
10
15
20
25
30
0.0
0.2
0.4
0.6
0.8
1.0

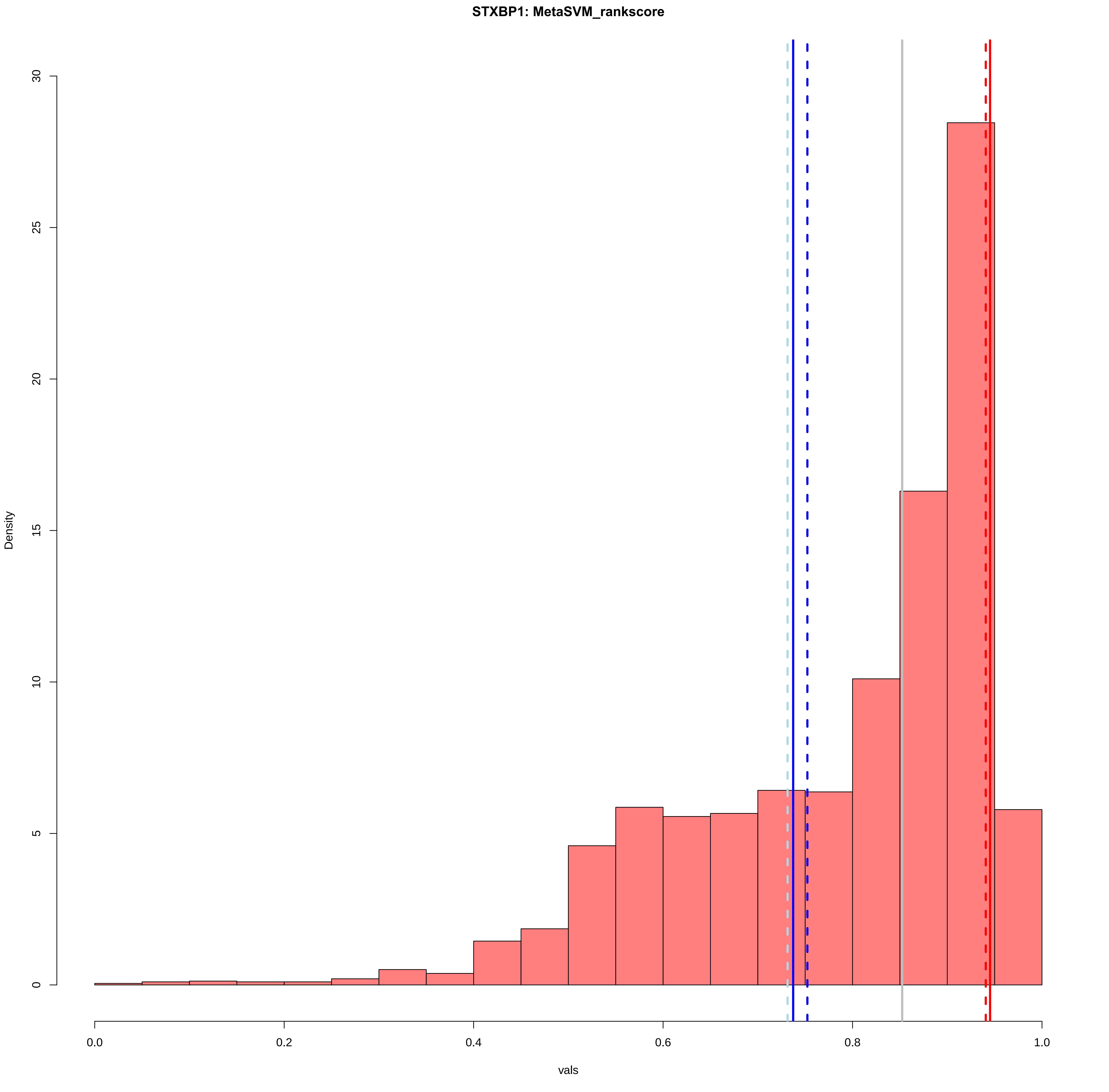

STXBP1: MetaSVM_rankscore
Density
vals
0
5
10
15
20
25
30
0.0
0.2
0.4
0.6
0.8
1.0

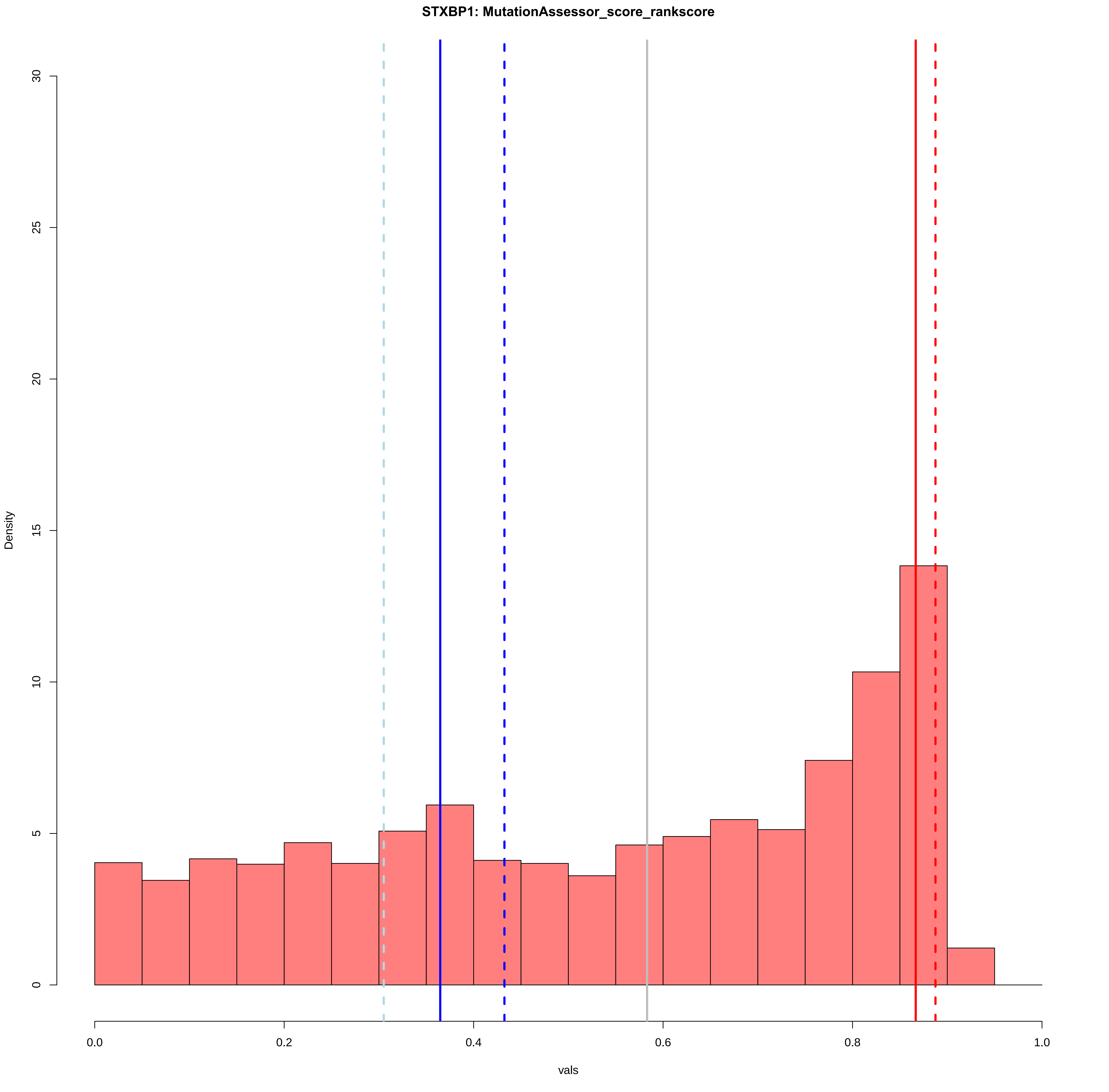
STXBP1: MutationAssessor_score_rankscore
Density
vals
0
5
10
15
20
25
30
0.0
0.2
0.4
0.6
0.8
1.0

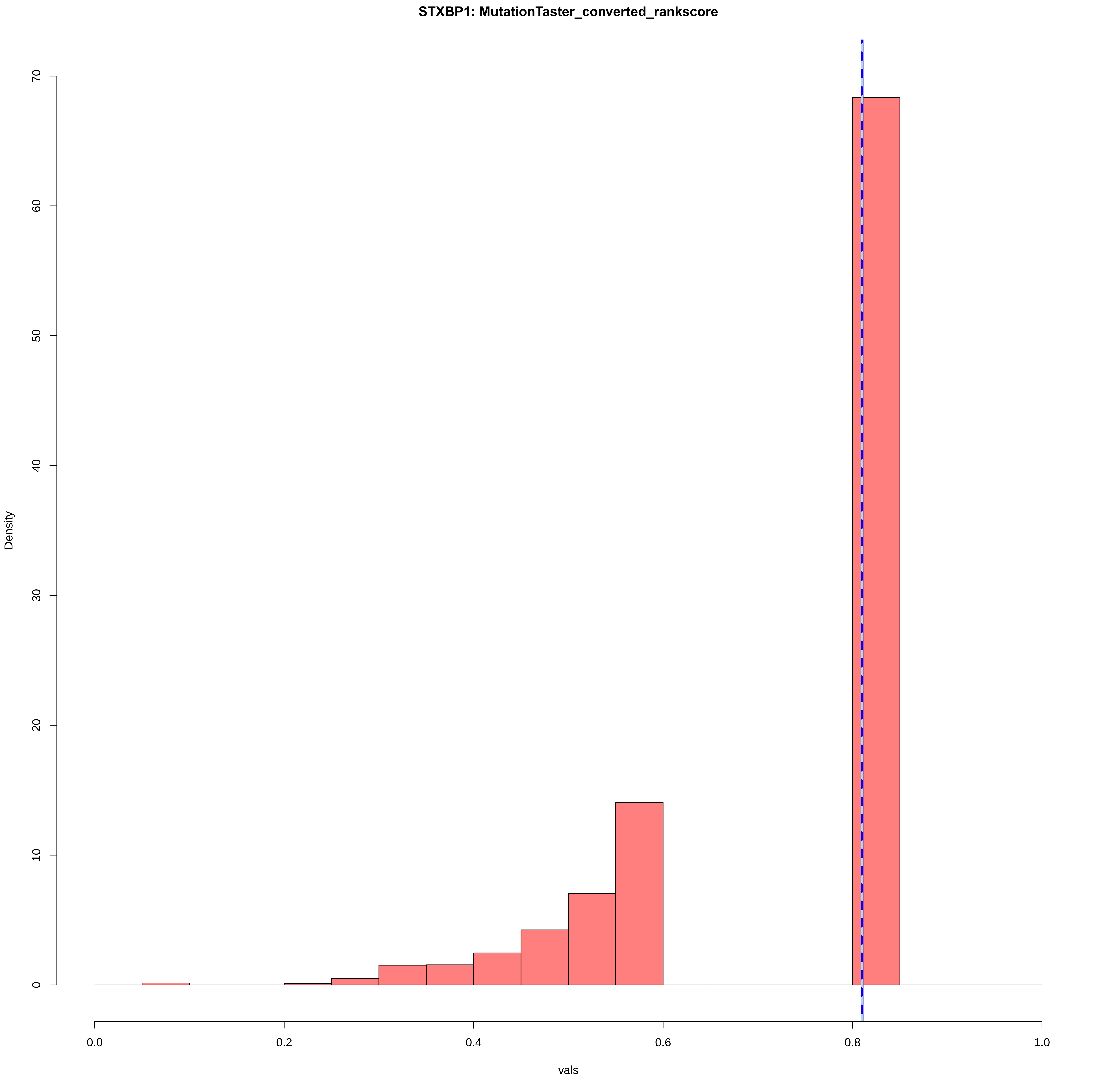
STXBP1: MutationTaster_converted_rankscore
Density
0
10
20
30
40
50
60
70
0.0
0.2
0.4
0.6
0.8
1.0
vals

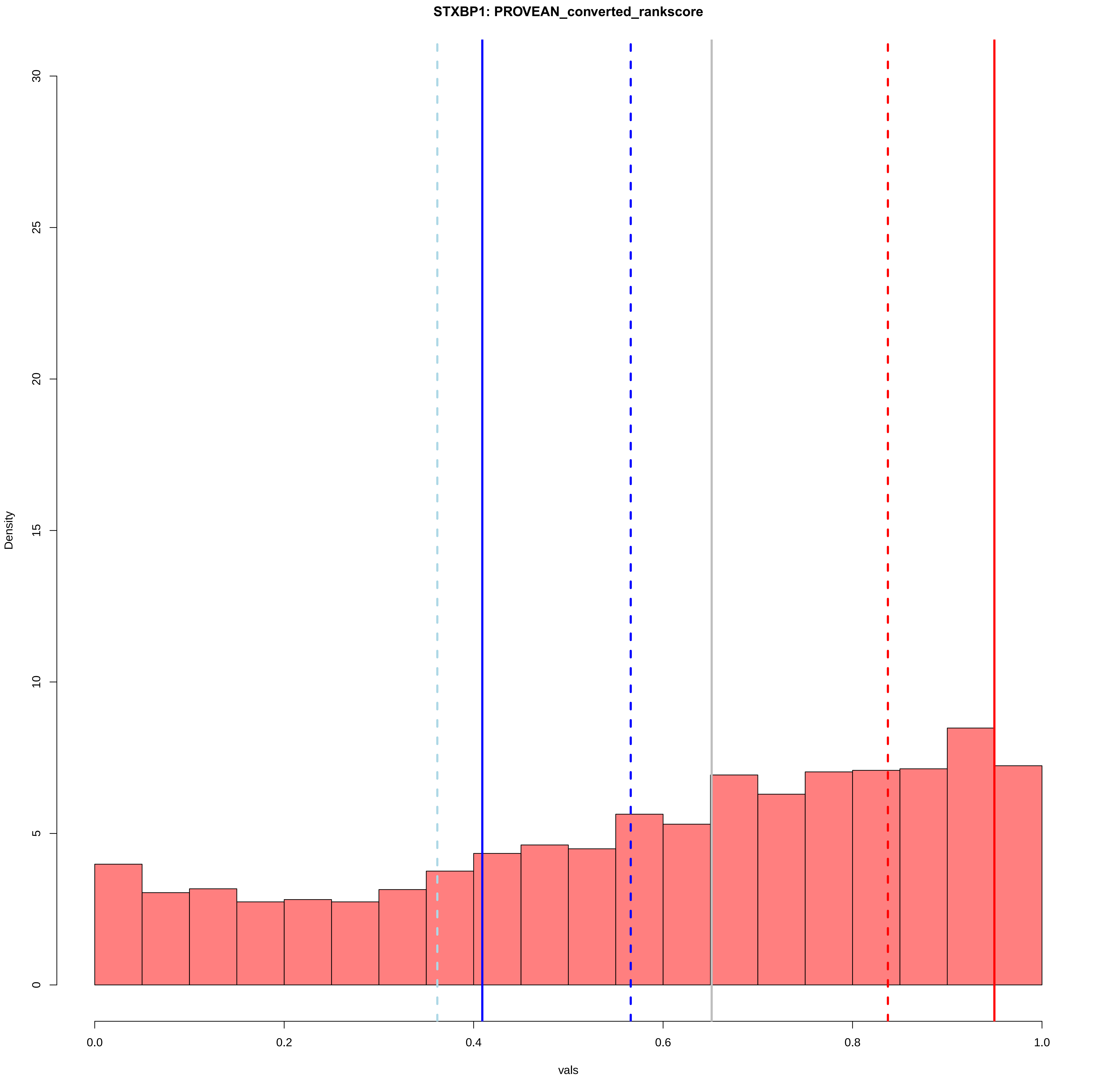

STXBP1: PROVEAN_converted_rankscore
Density
vals
0
5
10
15
20
25
30
0.0
0.2
0.4
0.6
0.8
1.0

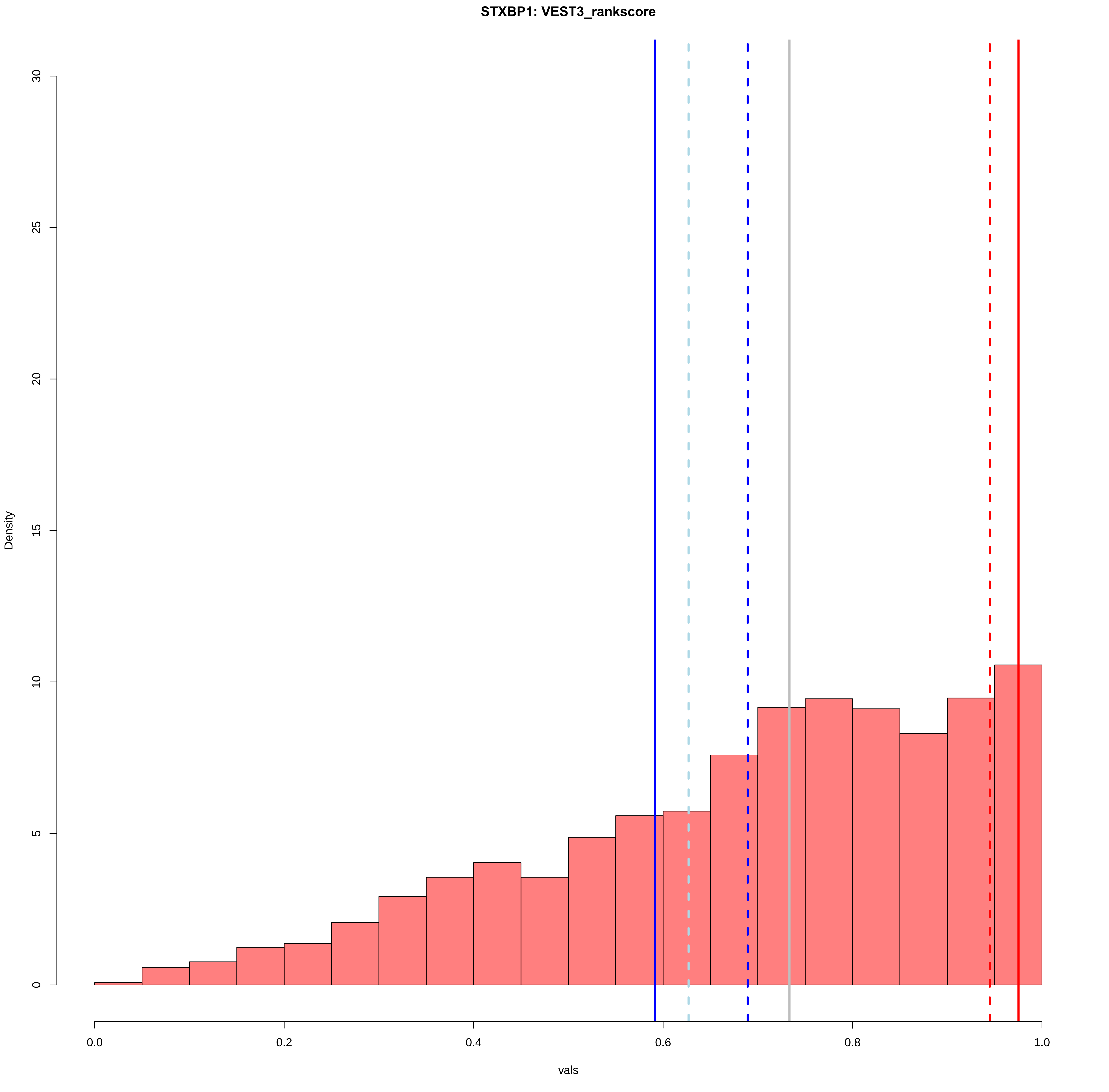

STXBP1: VEST3_rankscore
Density
0
5
10
15
20
25
30
0.0
0.2
0.4
0.6
0.8
1.0
vals

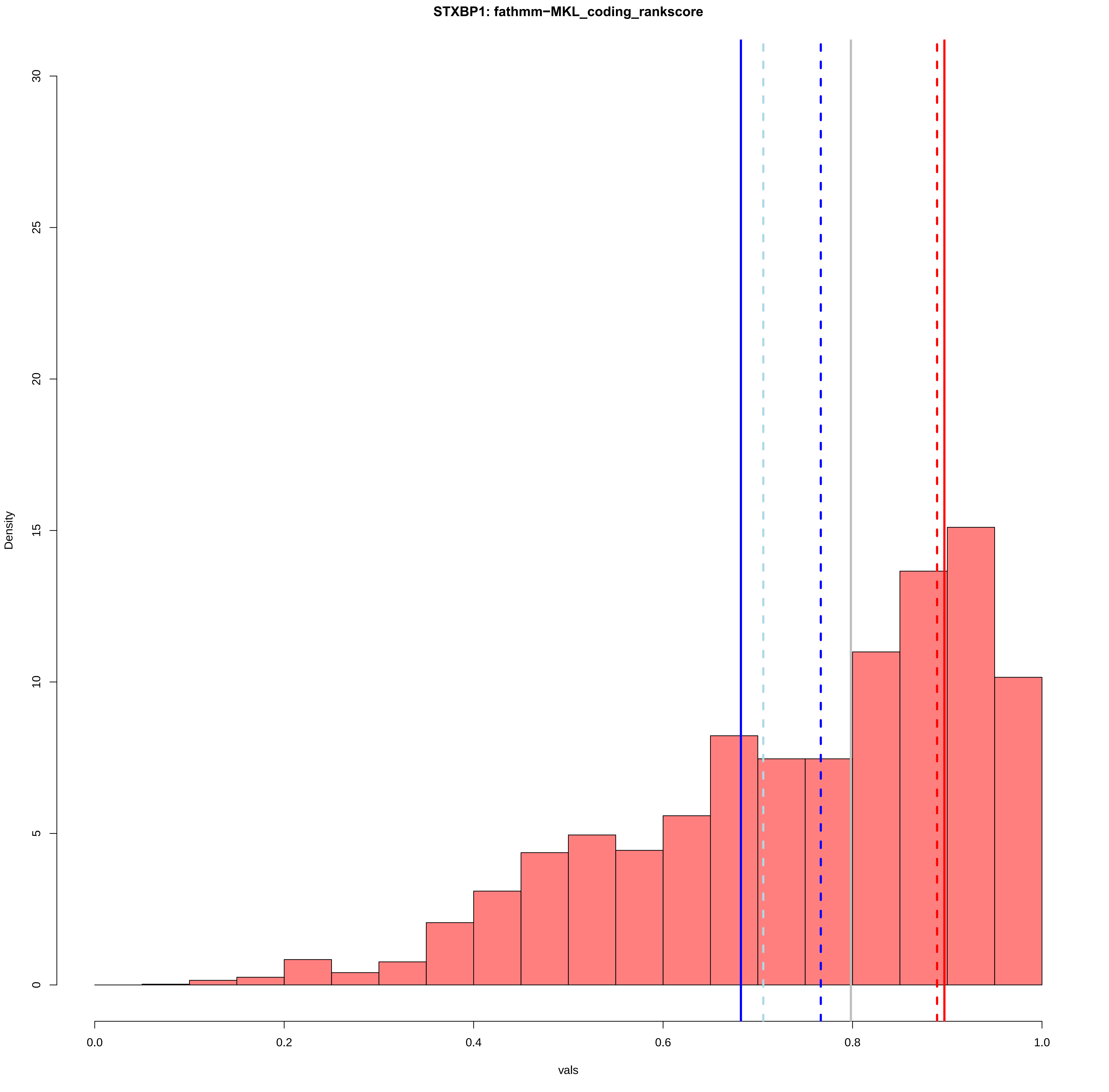
STXBP1: fathmm−MKL_coding_rankscore
Density
vals
0
5
10
15
20
25
30
0.0
0.2
0.4
0.6
0.8
1.0

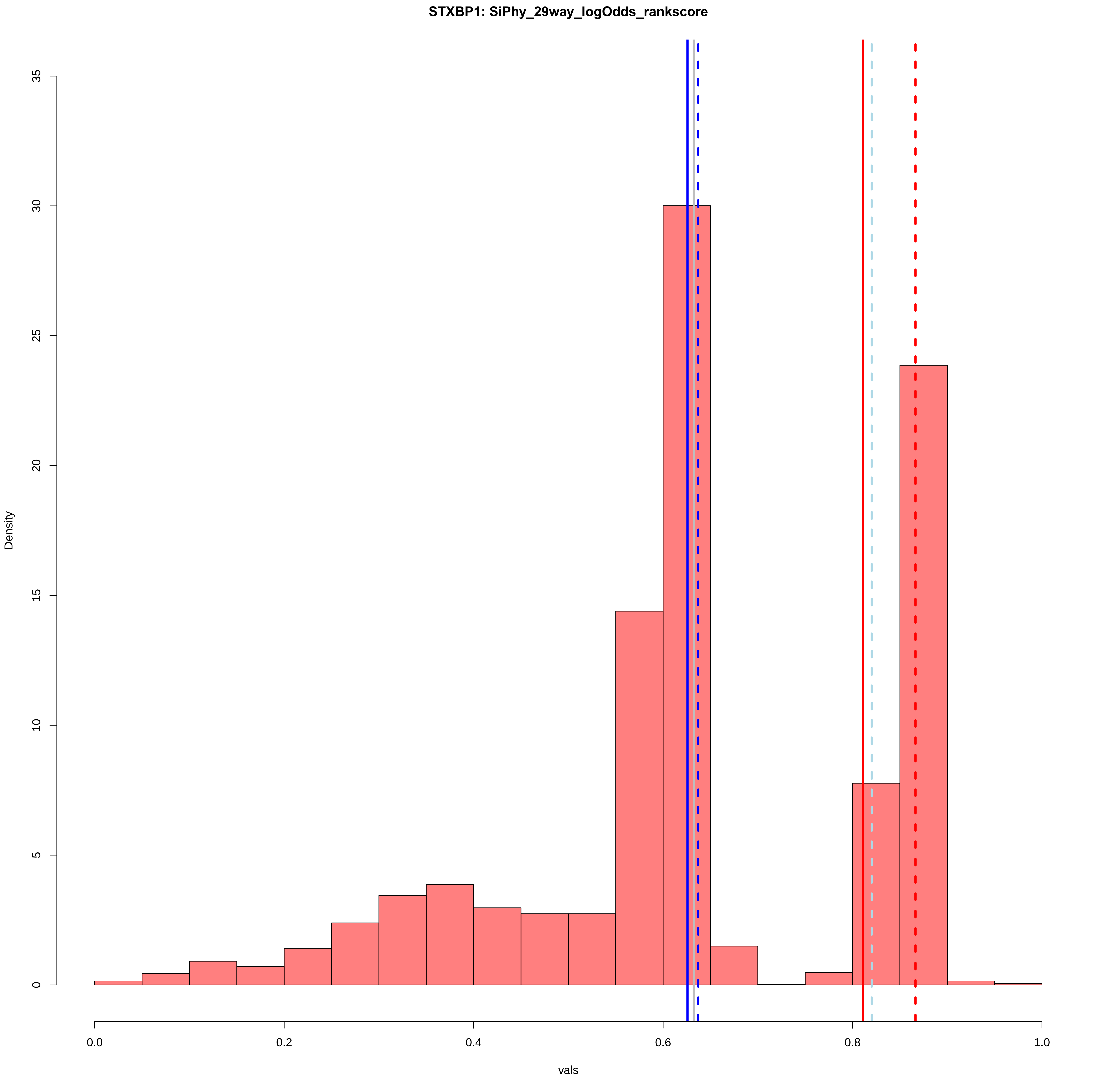

STXBP1: SiPhy_29way_logOdds_rankscore
Density
0
5
10
15
20
25
30
35
0.0
0.2
0.4
0.6
0.8
1.0
vals

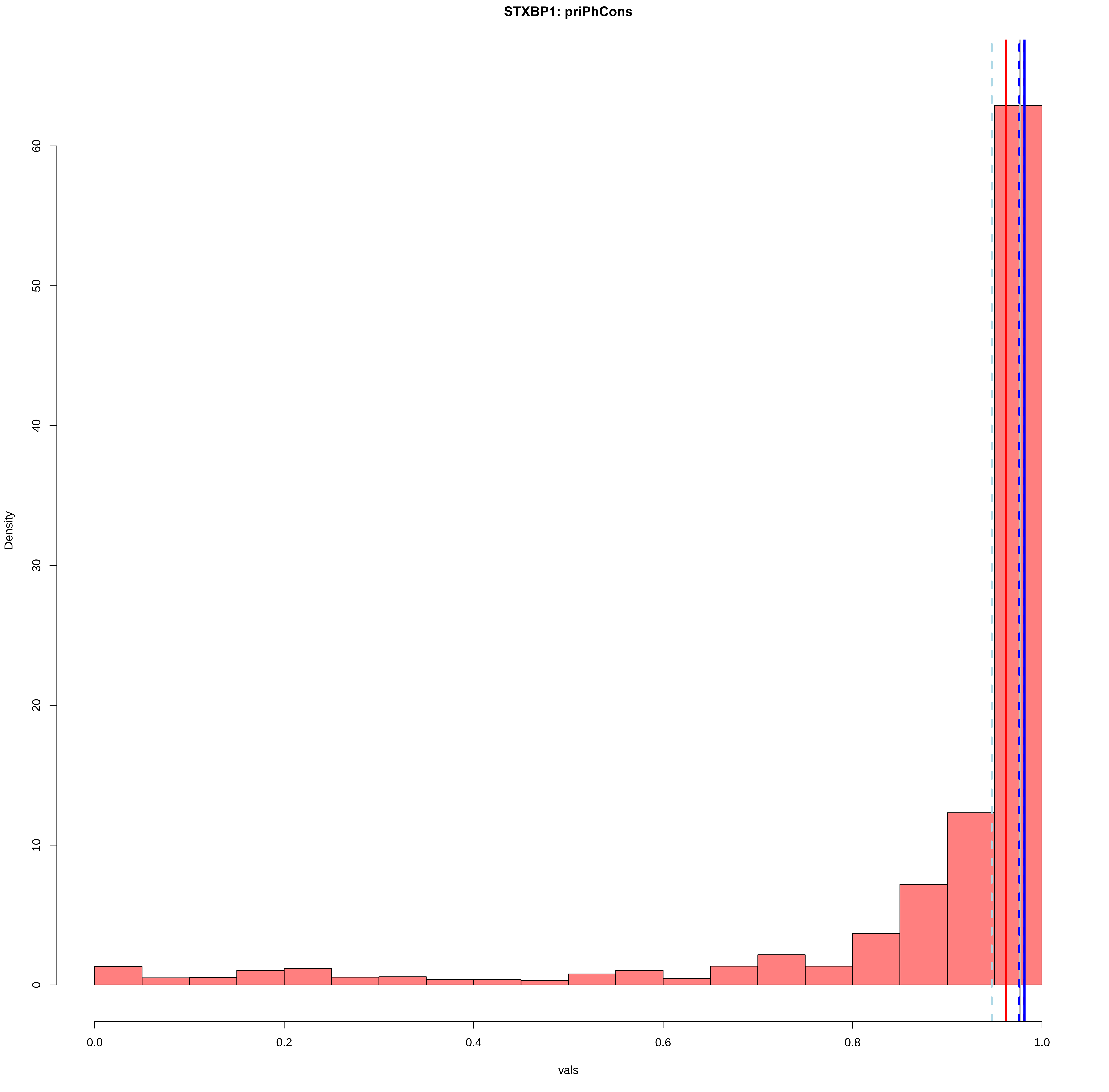

STXBP1: priPhCons
Density
vals
0
10
20
30
40
50
60
0.0
0.2
0.4
0.6
0.8
1.0

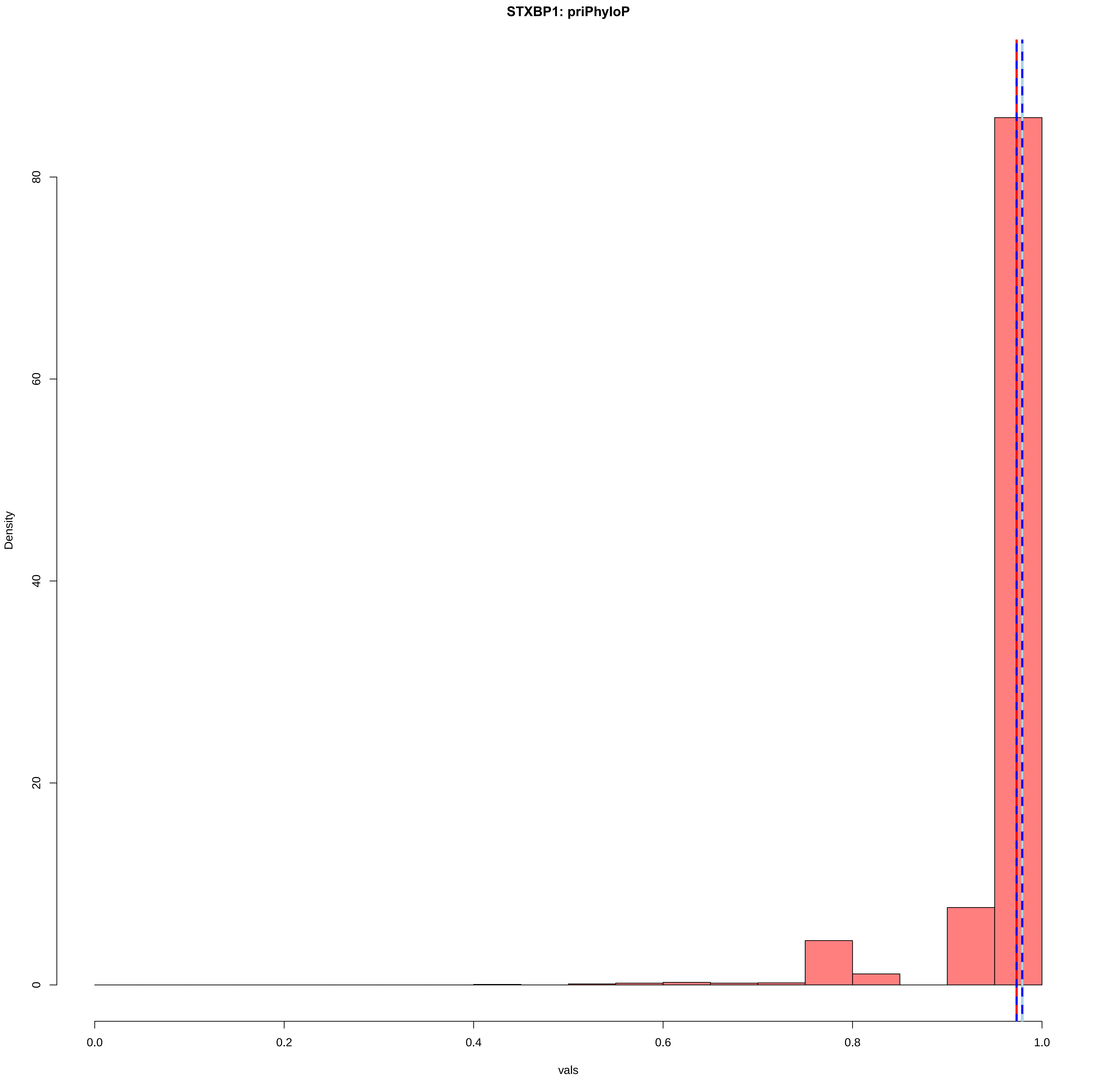

STXBP1: priPhyloP
Density
vals
0
20
40
60
80
0.0
0.2
0.4
0.6
0.8
1.0

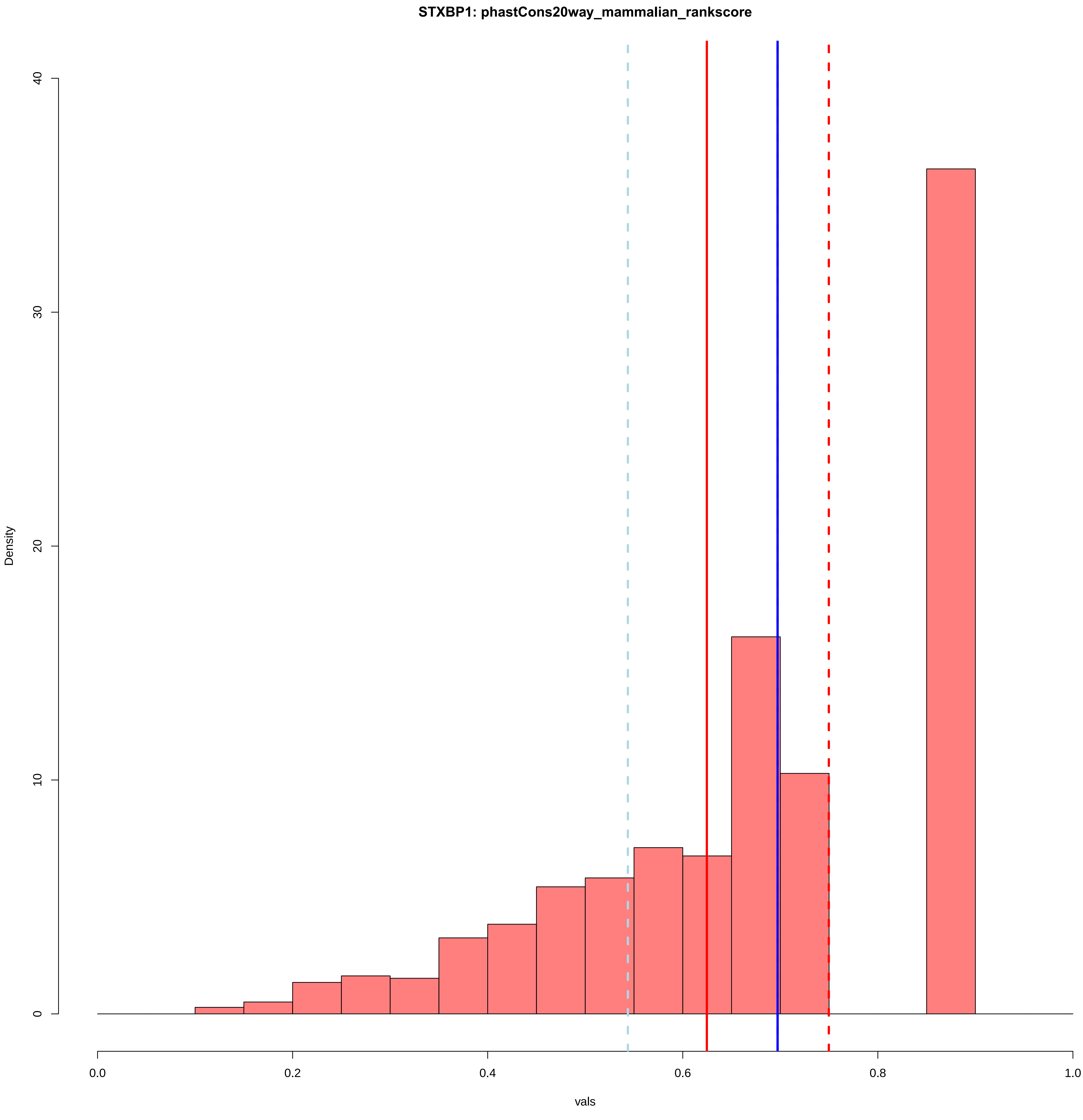

STXBP1: phastCons20way_mammalian_rankscore
Density
0
10
20
30
40
0.0
0.2
0.4
0.6
0.8
1.0
vals

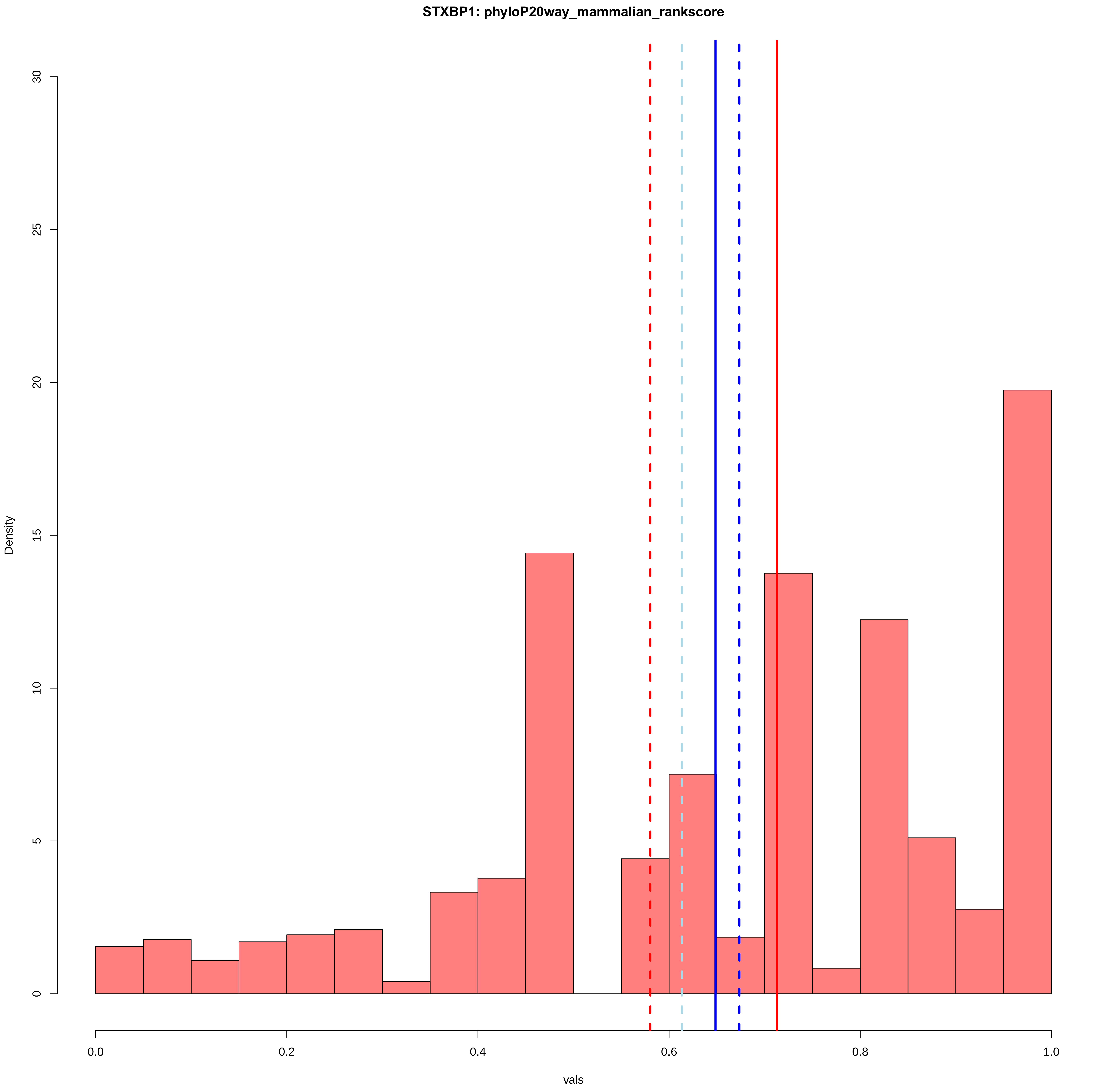
STXBP1: phyloP20way_mammalian_rankscore
Density
vals
0
5
10
15
20
25
30
0.0
0.2
0.4
0.6
0.8
1.0

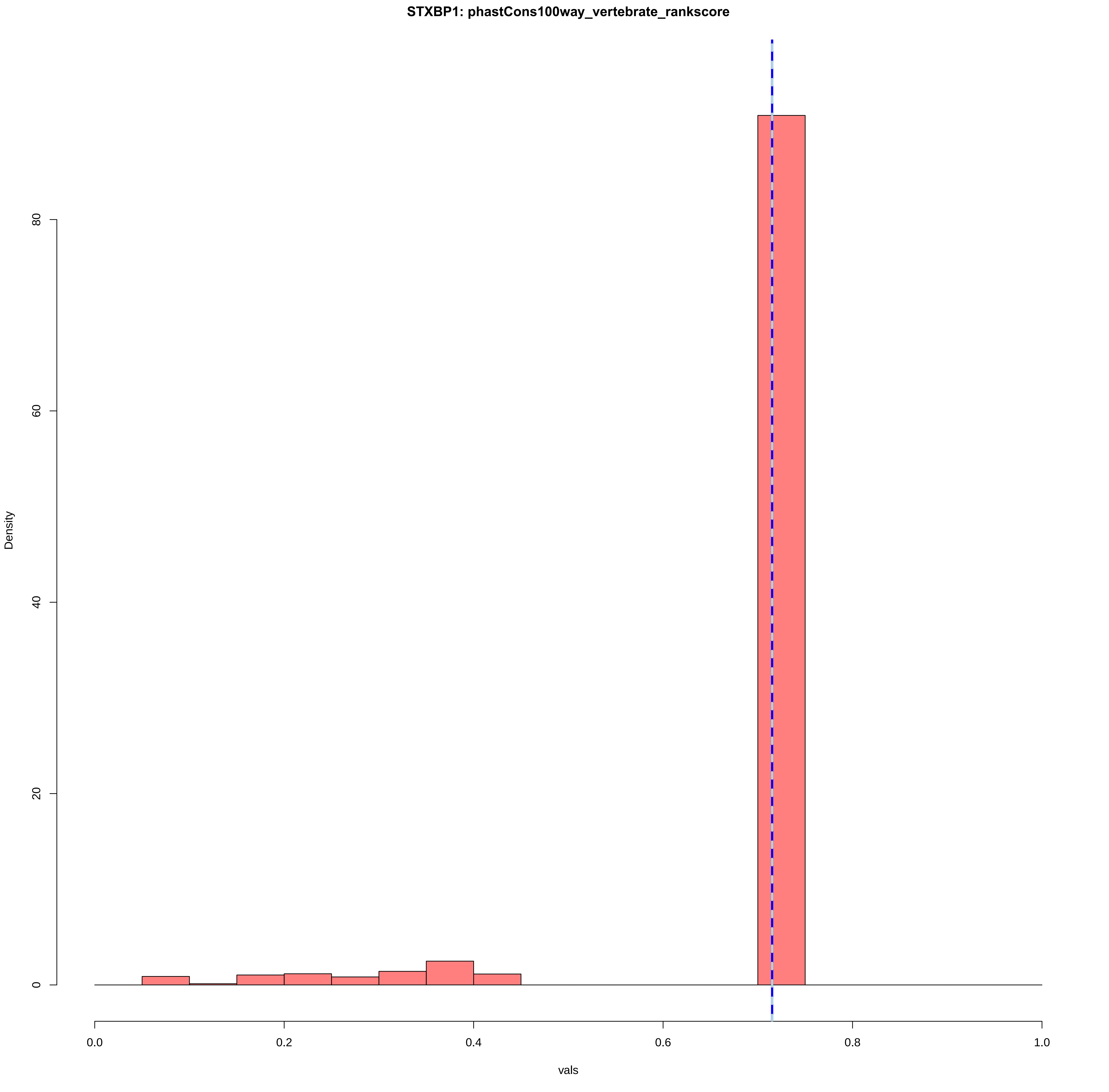
STXBP1: phastCons100way_vertebrate_rankscore
Density
vals
0
20
40
60
80
0.0
0.2
0.4
0.6
0.8
1.0

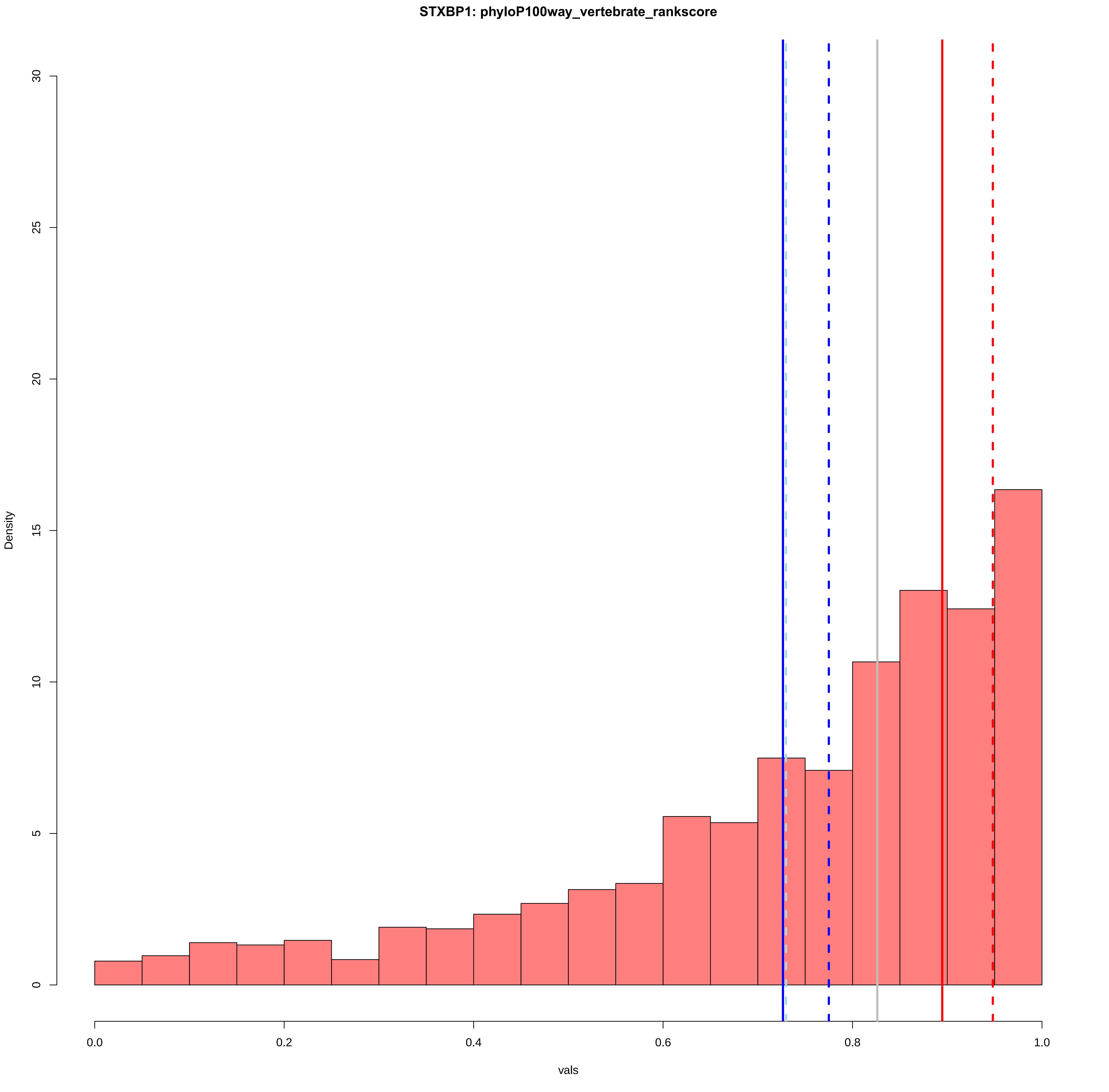

STXBP1: phyloP100way_vertebrate_rankscore
Density
vals
0
5
10
15
20
25
30
0.0
0.2
0.4
0.6
0.8
1.0

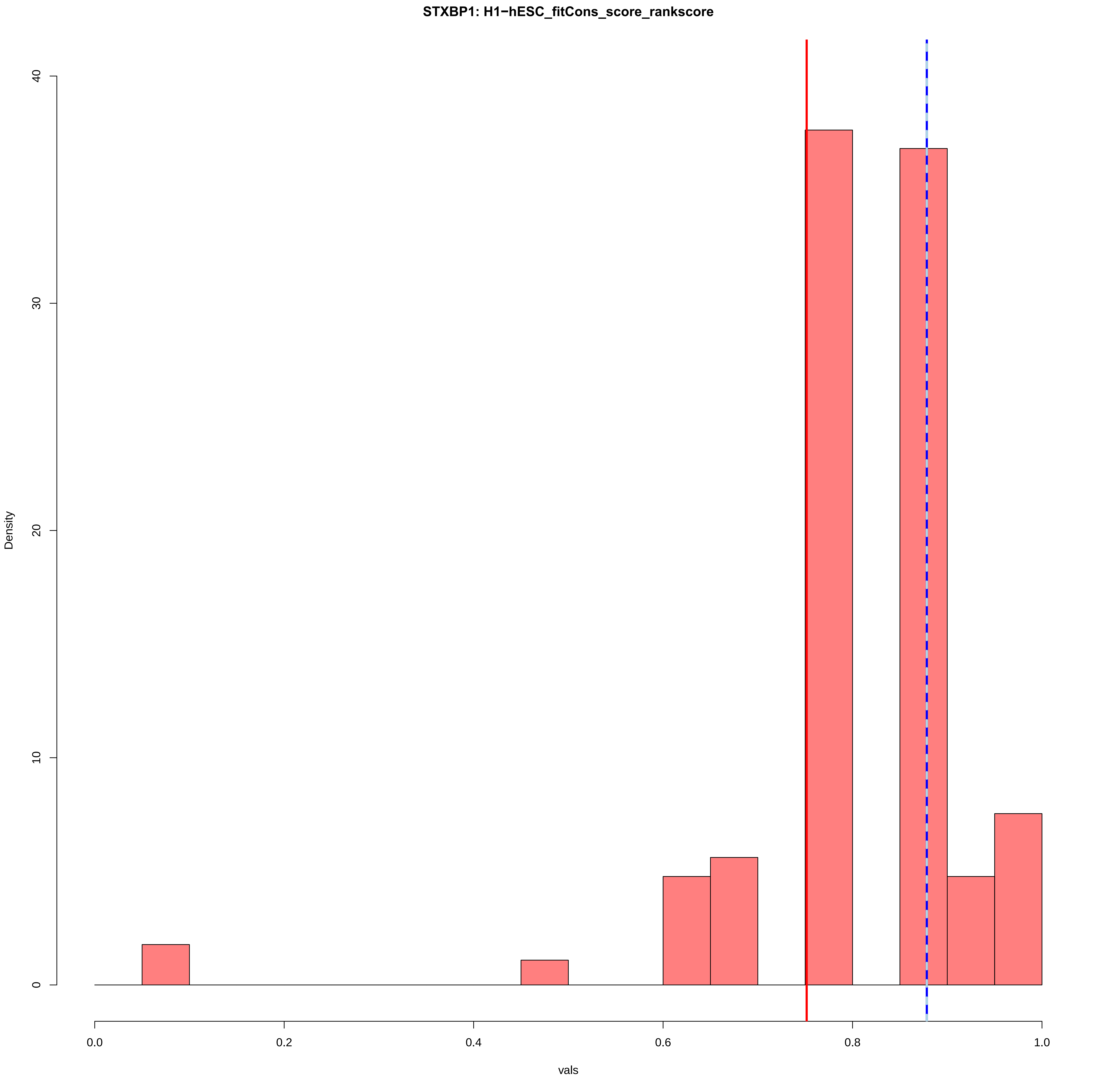
STXBP1: H1−hESC_fitCons_score_rankscore
Density
0
10
20
30
40
0.0
0.2
0.4
0.6
0.8
1.0
vals

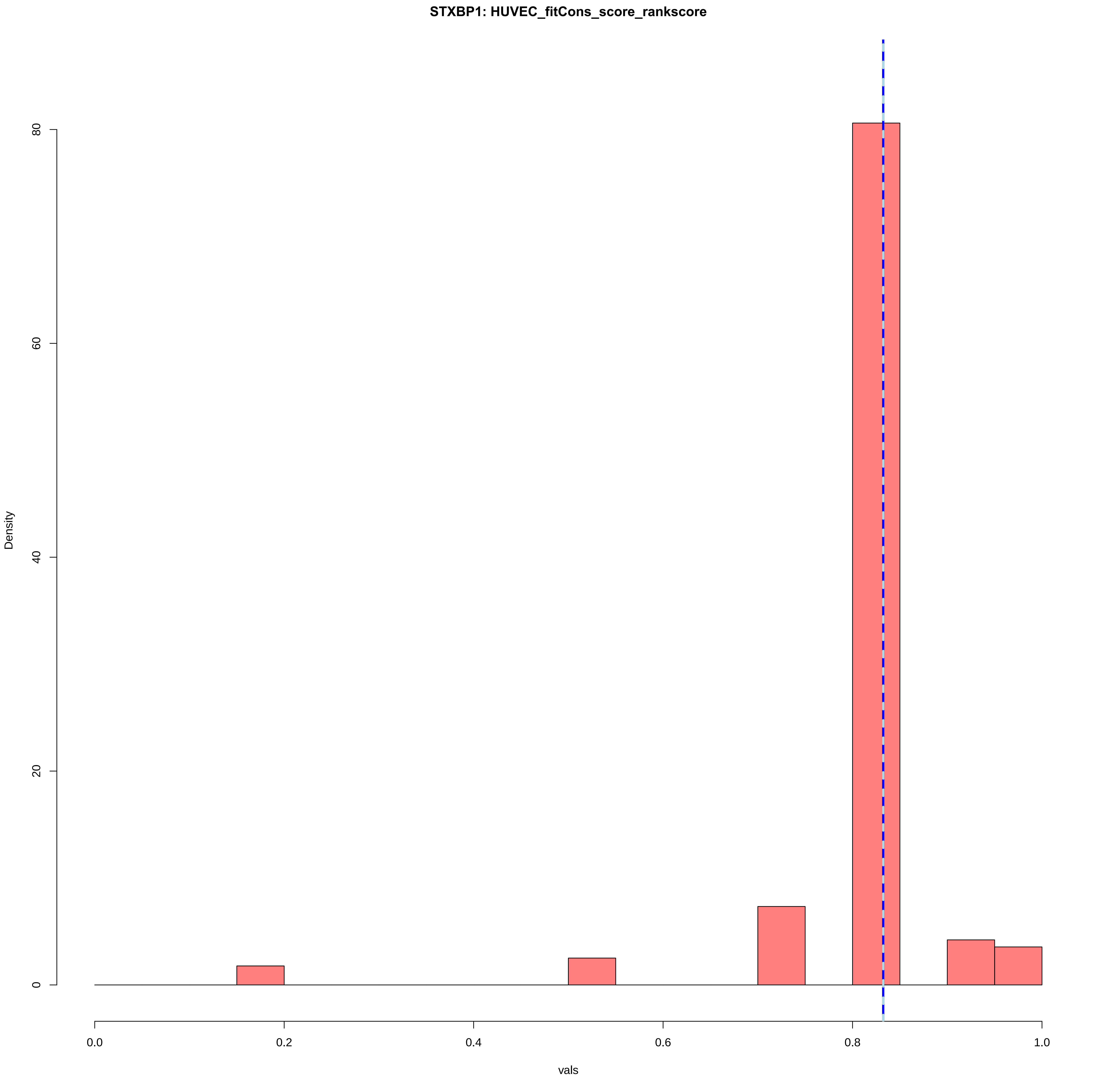

STXBP1: HUVEC_fitCons_score_rankscore
Density
0
20
40
60
80
0.0
0.2
0.4
0.6
0.8
1.0
vals

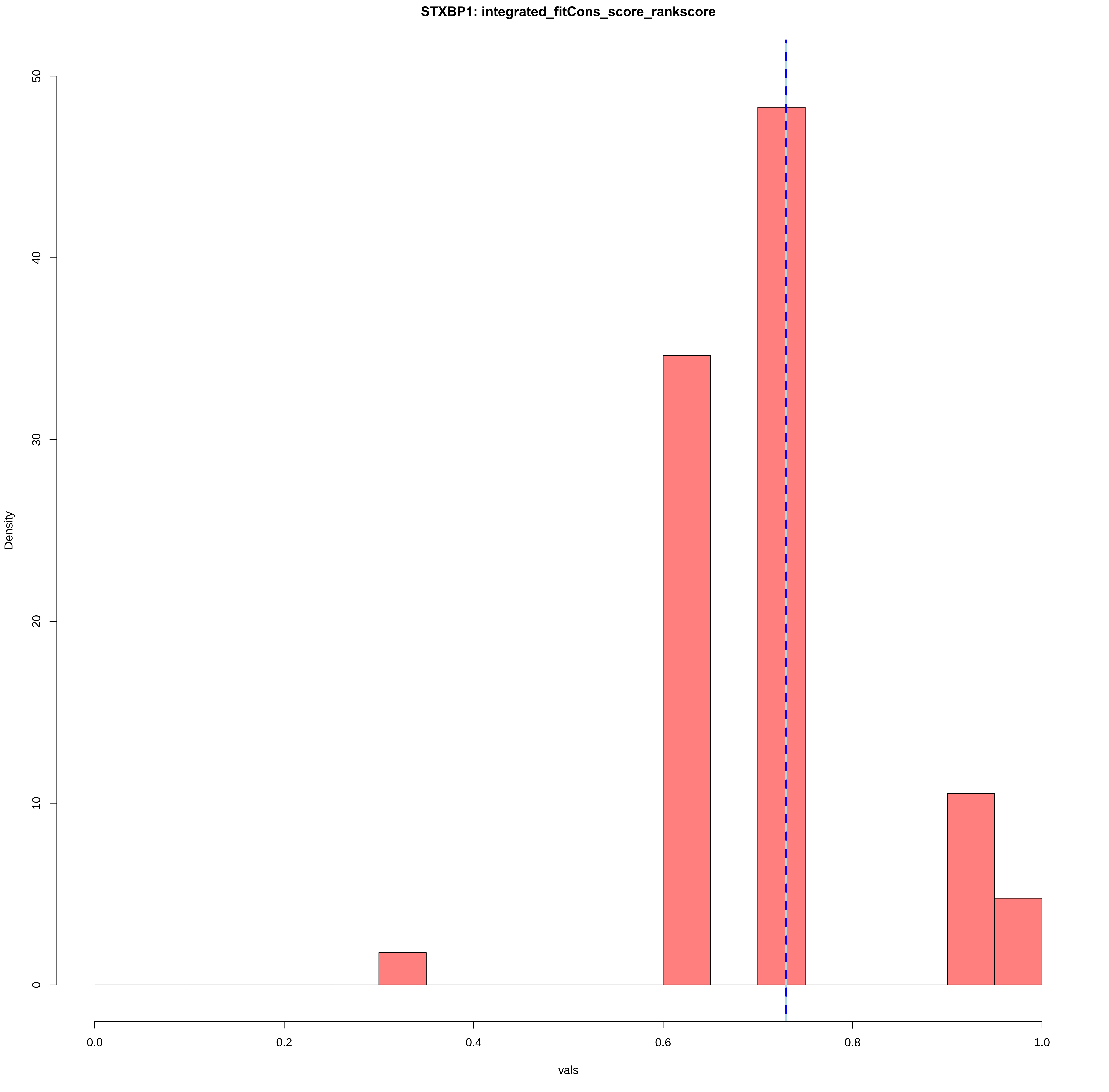

STXBP1: integrated_fitCons_score_rankscore
Density
vals
0
10
20
30
40
50
0.0
0.2
0.4
0.6
0.8
1.0

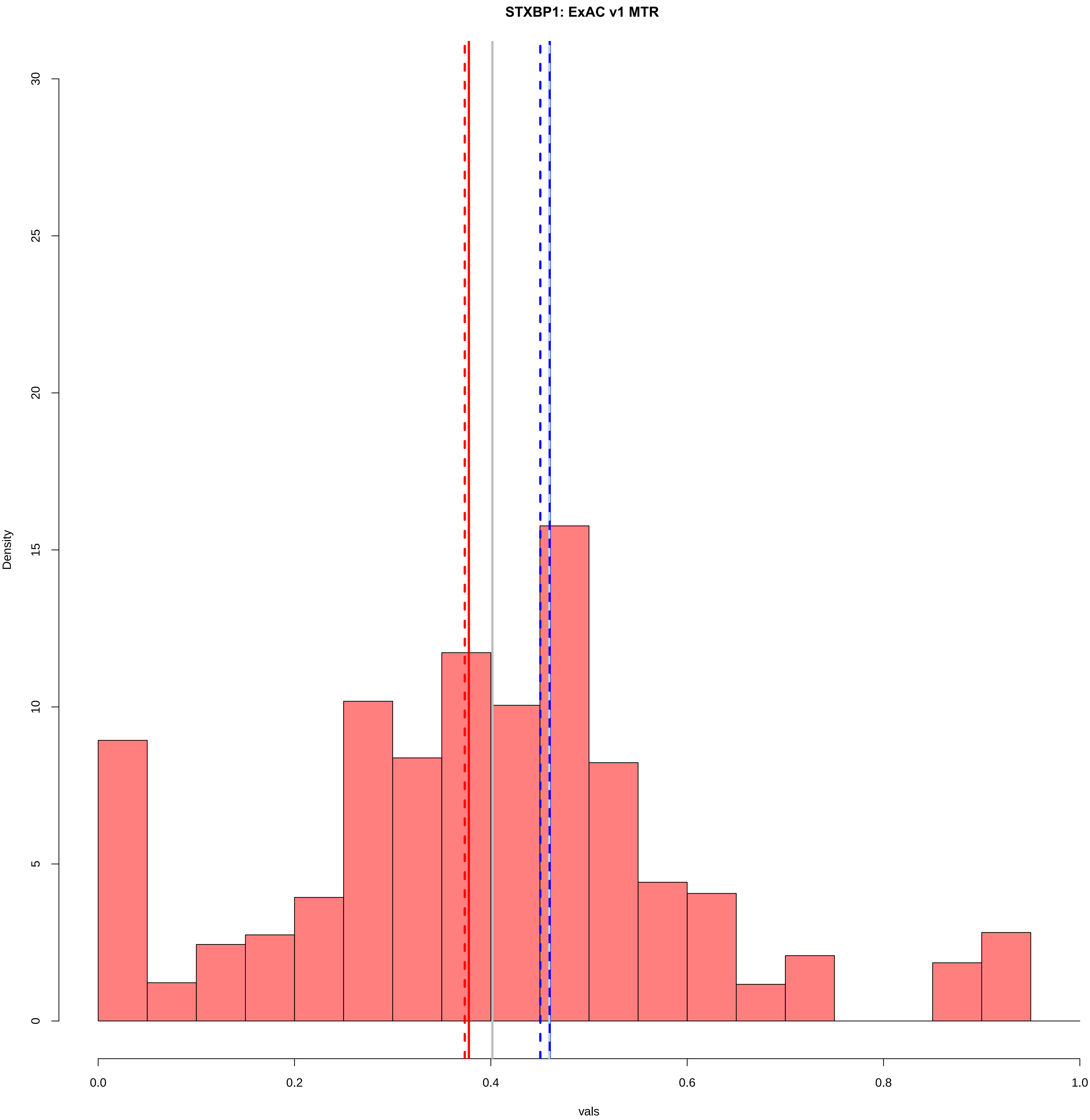

STXBP1: ExAC v1 MTR
Density
0
5
10
15
20
25
30
0.0
0.2
0.4
0.6
0.8
1.0
vals

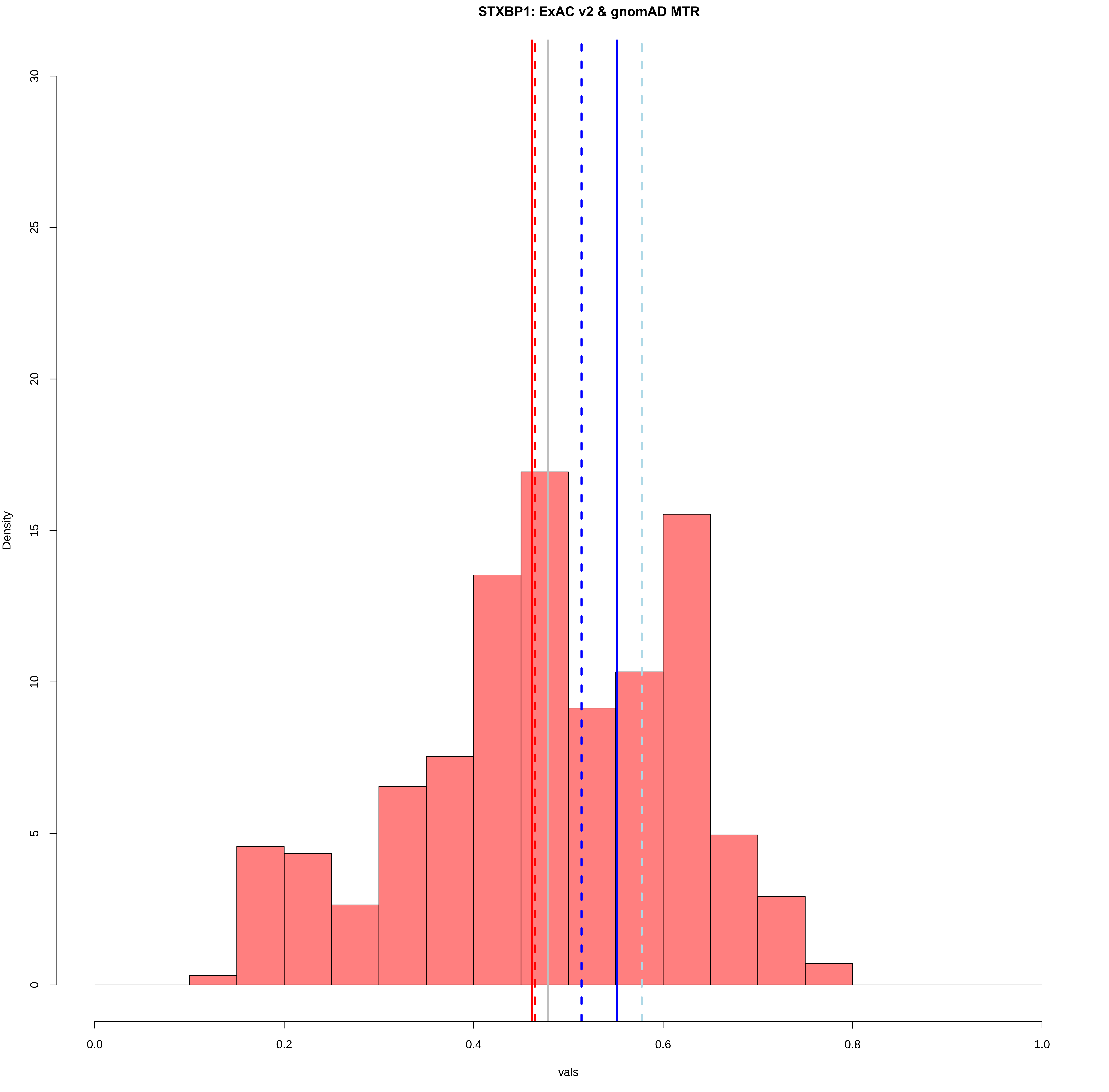
STXBP1: ExAC v2 & gnomAD MTR
Density
0
5
10
15
20
25
30
0.0
0.2
0.4
0.6
0.8
1.0
vals
