# Supplementary material for: Optimizing genomic medicine in epilepsy through a gene-customized approach to missense variant interpretation
Source: Genome Res. 2017 Oct;27(10):1715–29. doi: 10.1101/gr.226589.117 (PMC5630035; doi:10.1101/gr.226589.117)

A

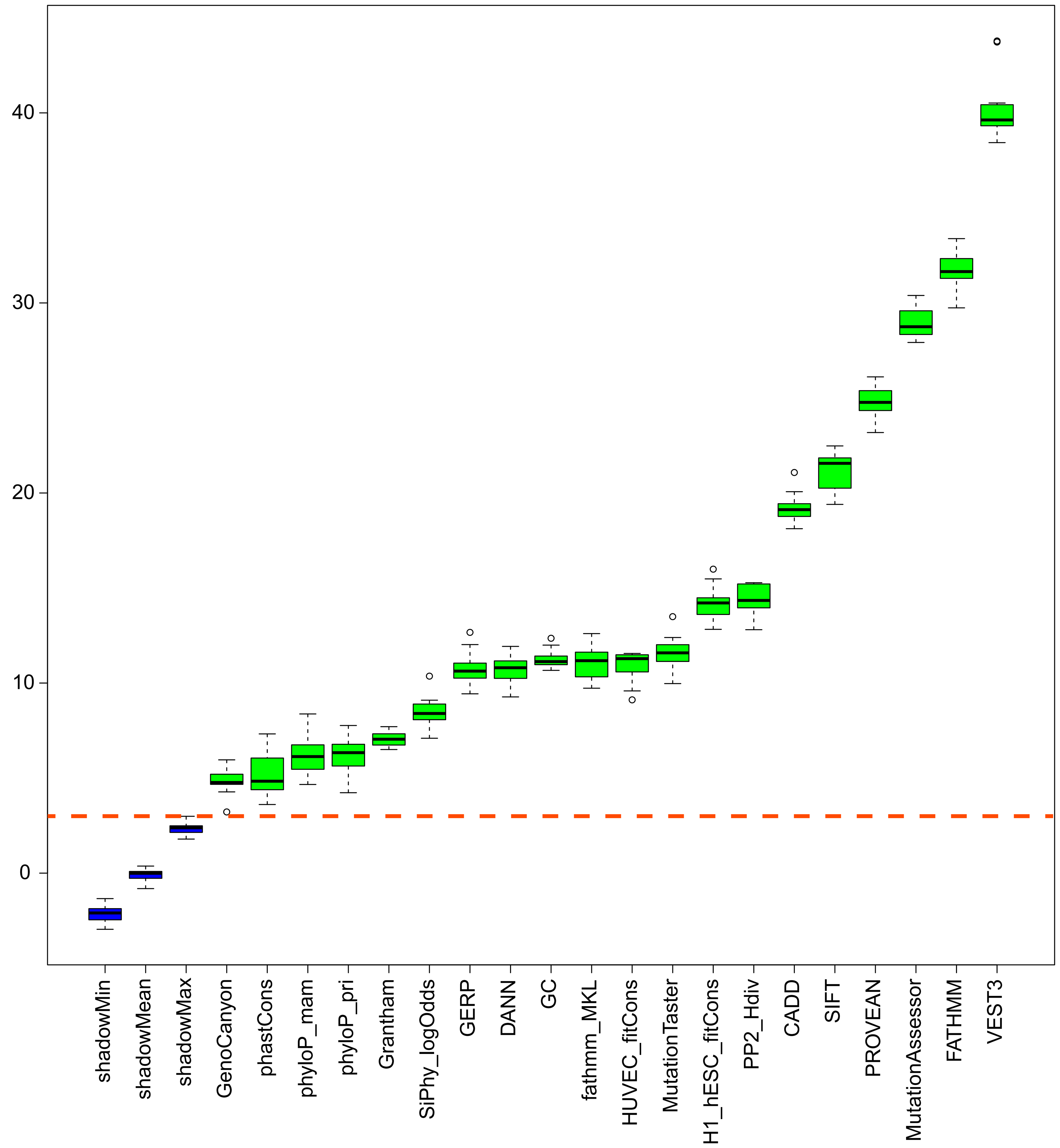

B

Combined 11 genes	Feature	ExAC v1 MTR	VEST3	FATHMM	Mutation Assessor	PROVEAN	SIFT
	AIC	2051.6	1367.6	1290.7	1221.9	1211.7	1206.8
	p-value	N/A	$p=3.0x10^{-159}$	$P=2x10^{-17}$	$P=1.1x10^{-15}$	P=0.006	P=0.09

C

Combined 11 genes	Feature	Intercept	ExAC v1 MTR	VEST3	FATHMM	Mutation Assessor	PROVEAN
	β	-7.55	-3.74	5.80	2.39	2.31	1.61
	p-value	$p=6.1x10^{-44}$	$p=5.5x10^{-34}$	$p=5.2x10^{-28}$	$p=1.5x10^{-9}$	$p=2.3x10^{-9}$	$p=5.6x10^{-4}$

D

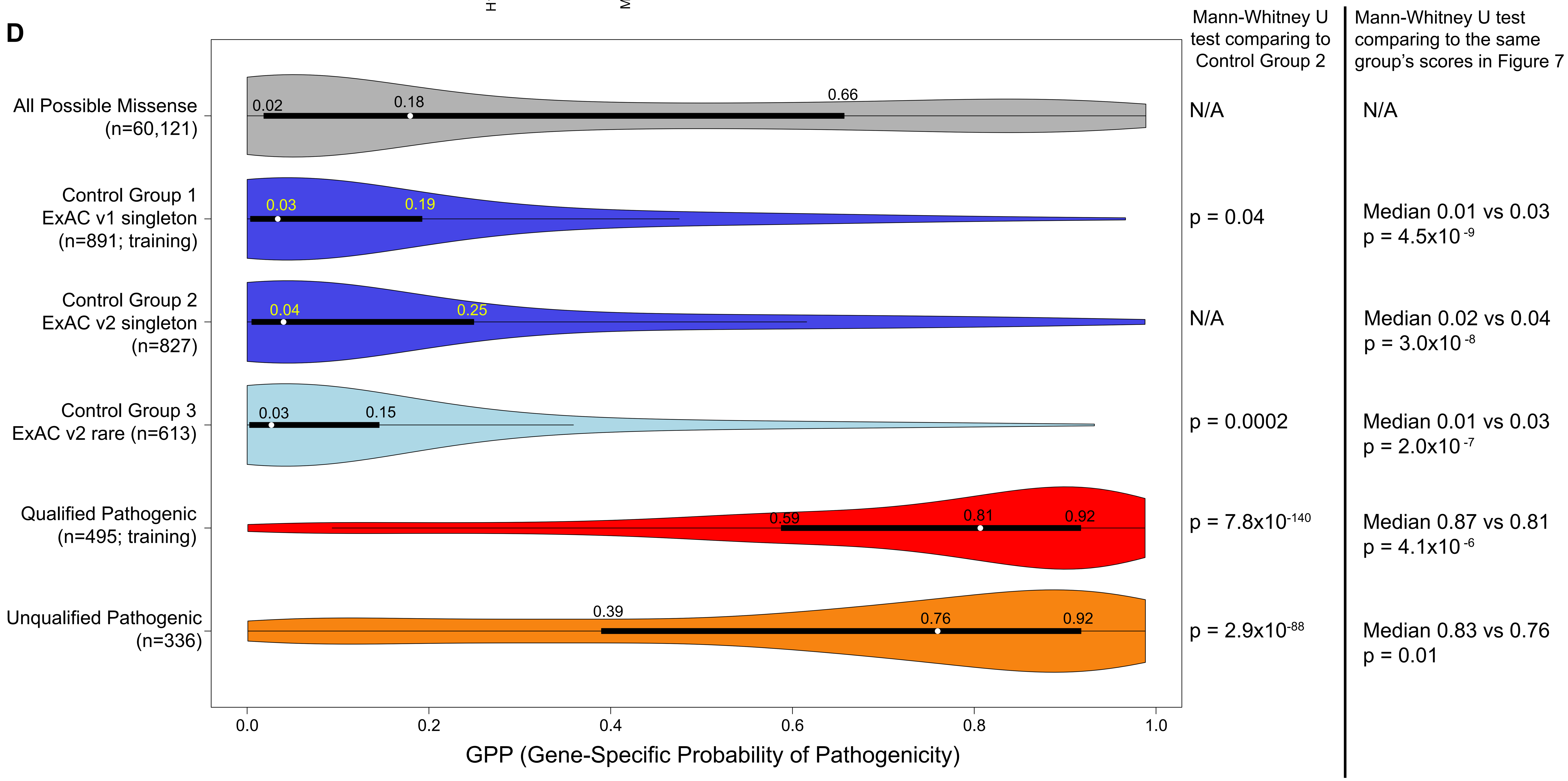

Supplement: Supplemental Material [file supp_gr.226589.117_Supplemental_Fig_S10.pdf]
