# Supplementary material for: Optimizing genomic medicine in epilepsy through a gene-customized approach to missense variant interpretation
Source: Genome Res. 2017 Oct;27(10):1715–29. doi: 10.1101/gr.226589.117 (PMC5630035; doi:10.1101/gr.226589.117)
Supplement: Supplemental Material [file supp_gr.226589.117_Supplemental_Appendix.pdf]

Supplemental Appendix. Optimizing genomic medicine in epilepsy through a gene-customized approach to missense variant interpretation

Table of Contents

Supplemental Fig S1: ExAC v2 (release 2.0) MTR plots for the eleven epilepsy genes in the context of pathogenic (qualified and unqualified) missense variant distributions 2

Supplemental Fig S2: ExAC v1 (release 0.3.1) MTR plots for the eleven epilepsy genes 6

Supplemental Fig S3: ExAC v1 MTR estimates comparing the pathogenic (red) to the ExAC v2 Control Group 2 (blue) missense variants distributions 7

Supplemental Fig S4: Correlation matrix of the evaluated bioinformatic tools (features); including the two additional MTR features 8

Supplemental Fig S5: Histogram distribution for all features across all genes 9

Supplemental Fig S6: Receiver Operating Characteristic (ROC) curves for the six genes with multivariate regression models, including individual features contributing to that model 10

Supplemental Fig S7: Gene-specific Probability of Pathogenicity (GPP) distributions, per gene........ 11

Supplemental Fig S8: Variant GPP probability of pathogenicity score distribution after fitting the model with the unqualified case variants and the ExAC v2 control missense variants 12

Supplemental Fig S9: Comparing GPP to MPC predictions for the six epilepsy genes........................ 13

Supplemental Fig S10: Gene-specific Probability of Pathogenicity (GPP) score distribution after fitting the model with the combined eleven gene data rather than a gene-customized approach.... 14

Supplemental Fig S11: Correlation of MTR with log(dN/dS) using the ExAC v1 sliding windows 15

Supplemental Fig S12: Comparison of MTR with subRVIS and regional constraint estimates 16

Supplemental Table S1: Breakdown for the curation of the eleven epilepsy genes 17

Supplemental Table S2: Summary of pathogenic classified variants among eleven epilepsy genes.. 18

Supplemental Table S3: Per gene summaries of the standing variation data found among the combined ExAC v2 and gnomAD population sample of 138,632 samples 19

Supplemental Table S4: List of the 31 tools (predictors / features) evaluated on the eleven genes . 20

Supplemental Table S5: List of the features found by the Boruta algorithm to be uninformative, informative/tentative, and highly informative for each studied gene 21

Supplemental Table S6: Summary of the top feature distribution for the nine epilepsy genes with at least one highly informative feature... 24

Supplemental Table S7: Akaike information criterion (AIC) comparisons across growing logistic regression models, per gene.. 25

Supplemental Table S8: Final multivariate logistic regression models, per gene. 26

Supplemental Data S1: Review of segregation support accompanying missense variants reported in the eleven genes among ClinVar and/or HGMD® catalogs 27

Supplemental Data S2: Summary of patient-ascertained pathogenic missense variants found to overlap with population reference cohorts 28

Supplemental Data S3: ExAC v1 and ExAC v2 MTR estimates for all windows across the eleven epilepsy genes 29

Supplemental Data S4: Contains the full set of annotations for every possible missense variant in the canonical transcript of each of the eleven epilepsy genes.. 30

Supplemental Fig. S1: ExAC v2 (release 2.0) MTR plots for the eleven epilepsy genes in the context of pathogenic (qualified and unqualified) missense variant distributions

MTR plots: *CDKL5*, *GRIN2A*, *KCNQ2*, *KCNT1*, *LGI1*, *PCHD19*, *SLC2A1, STXBP1, SCN1A*, *SCN2A*, and *SCN8A*. Regions in red achieved a study-wide FDR < 0.05 (**Supplemental Data S3**). MTR=1 is depicted by dashed blue, the gene's median MTR (dashed black), 25th centile MTR (dashed dark green) and 5th centile MTR (dashed orange). Grey dashed line reflects proportion of all ExAC v2 samples that achieved at least 10-fold coverage at the sites relevant to that codon (Methods). Beneath each MTR plot is the linear distribution of the 606 qualified (red) and 437 unqualified (grey) pathogenic missense variants.

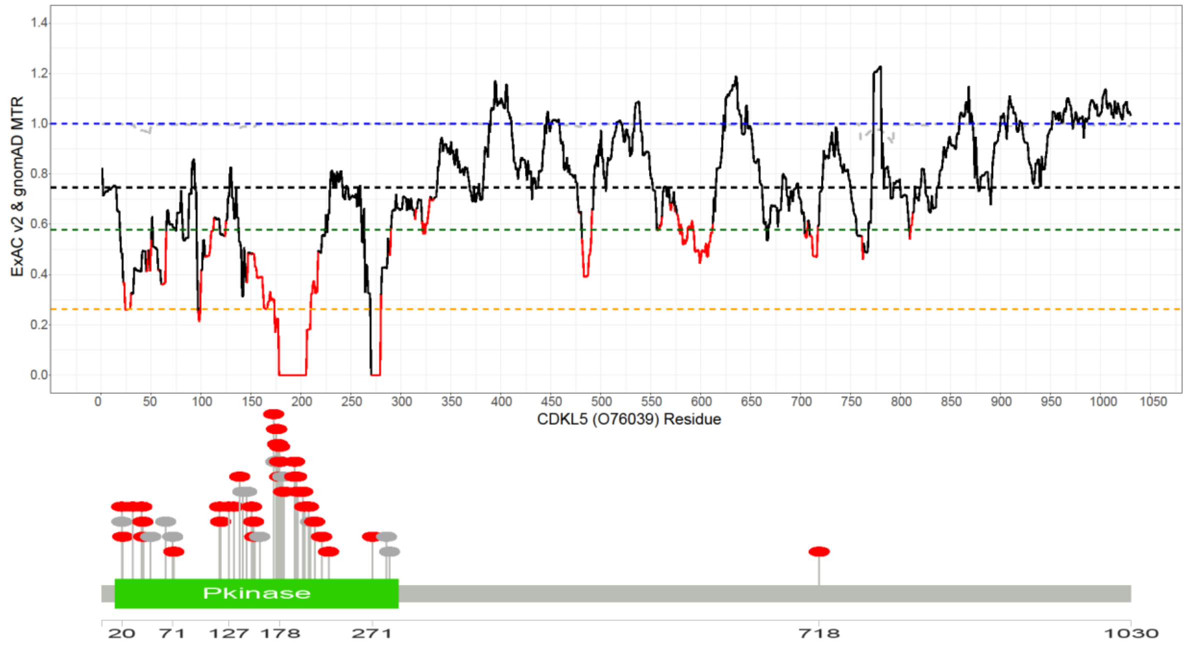

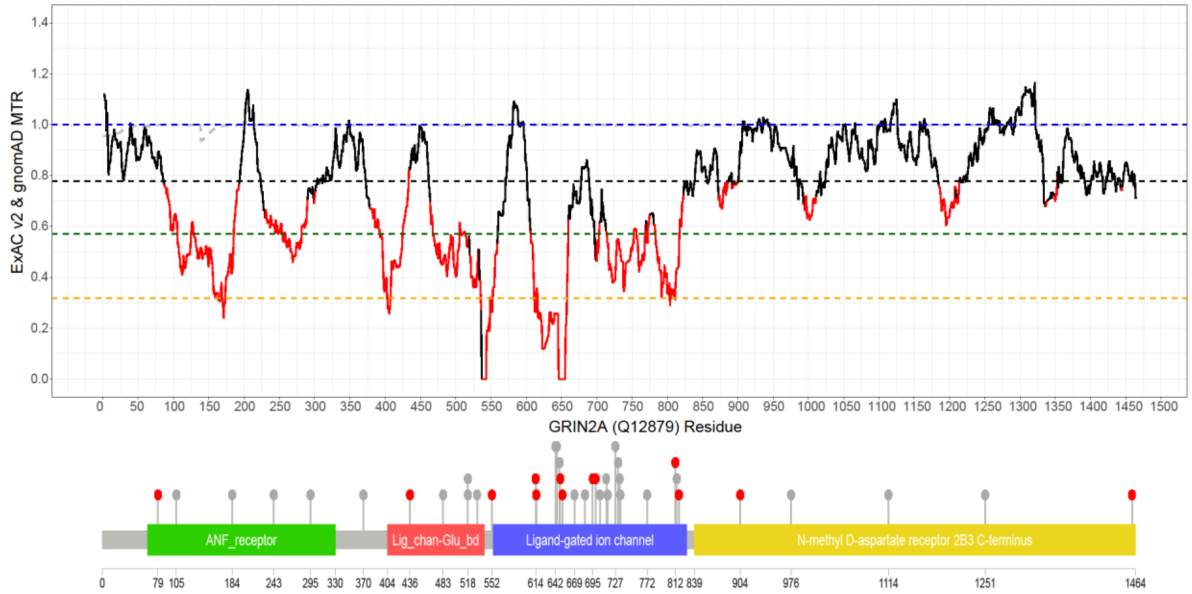

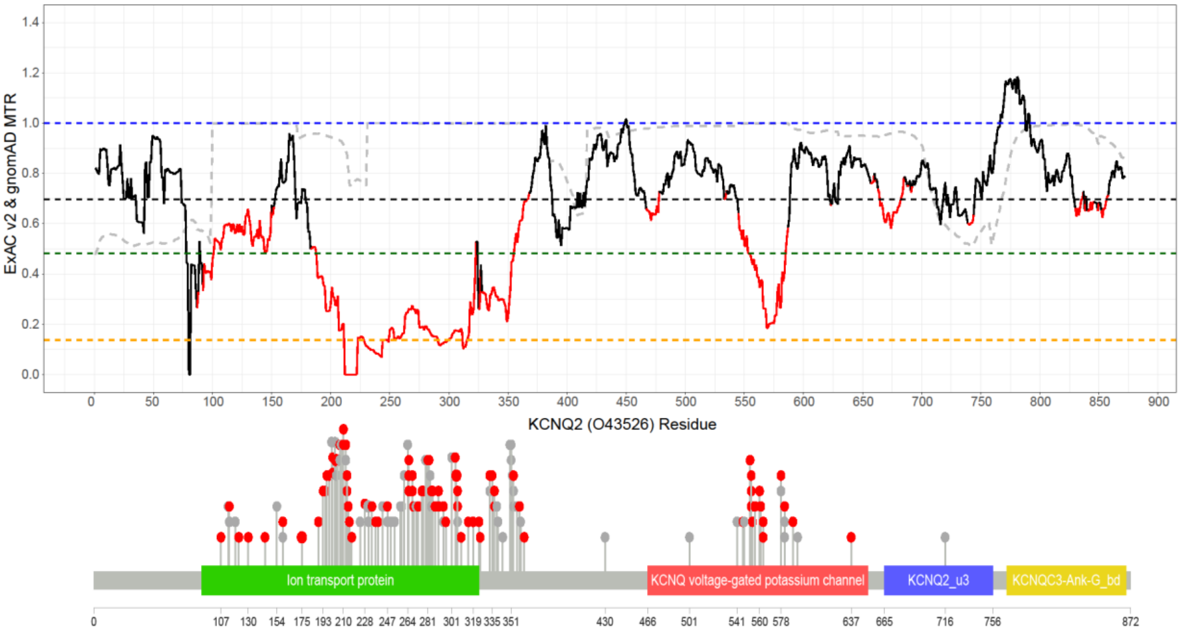
ExAC v2 & gnomAD MTR
KCNQ2 (O43526) Residue
Ion transport protein
KCNQ voltage-gated potassium channel
KCNQ2_u3
KCNQC3-Ank-G_bd
0 107 130 154 175 193 210 228 247 264 281 301 319 335 351 430 466 501 541 560 578 637 665 716 756 872

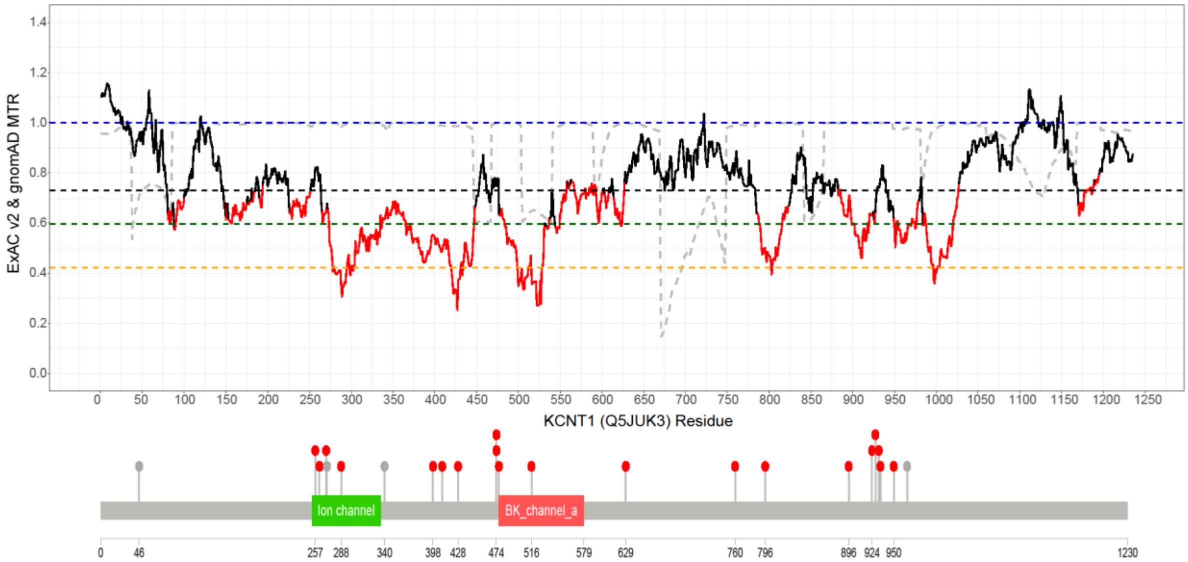
ExAC v2 & gnomAD MTR
KCNT1 (Q5JUK3) Residue
Ion channel
BK_channel_a
0 46 257 288 340 398 428 474 516 579 629 760 796 896 924 950 1230

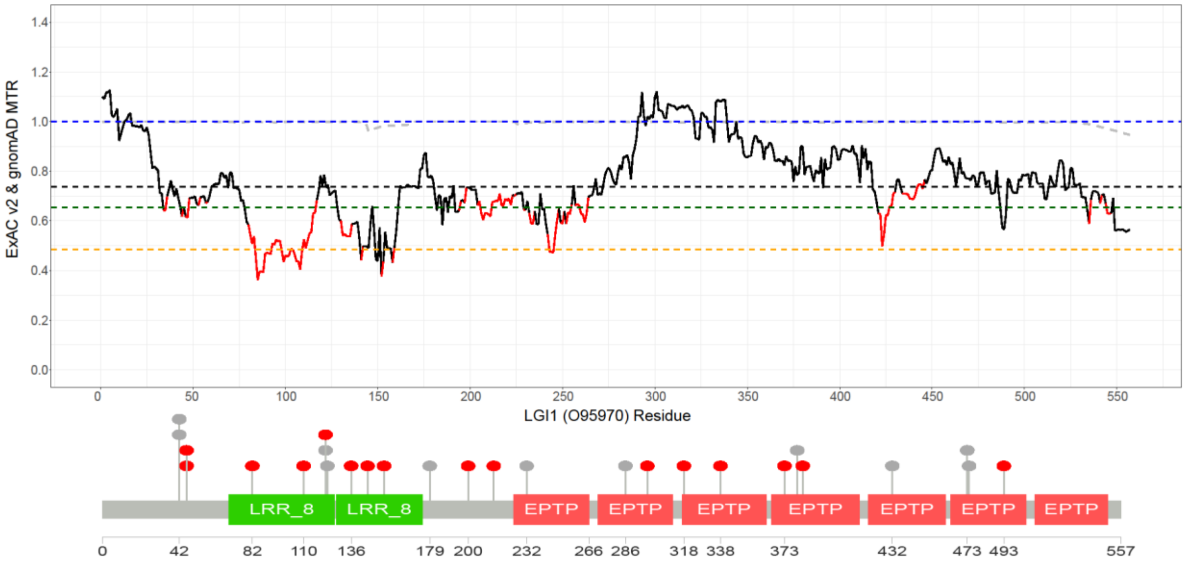
ExAC v2 & gnomAD MTR
LGI1 (O95970) Residue
LRR_8
LRR_8
EPTP
EPTP
EPTP
EPTP
EPTP
EPTP
EPTP
0 42 82 110 136 179 200 232 266 286 318 338 373 432 473 493 557

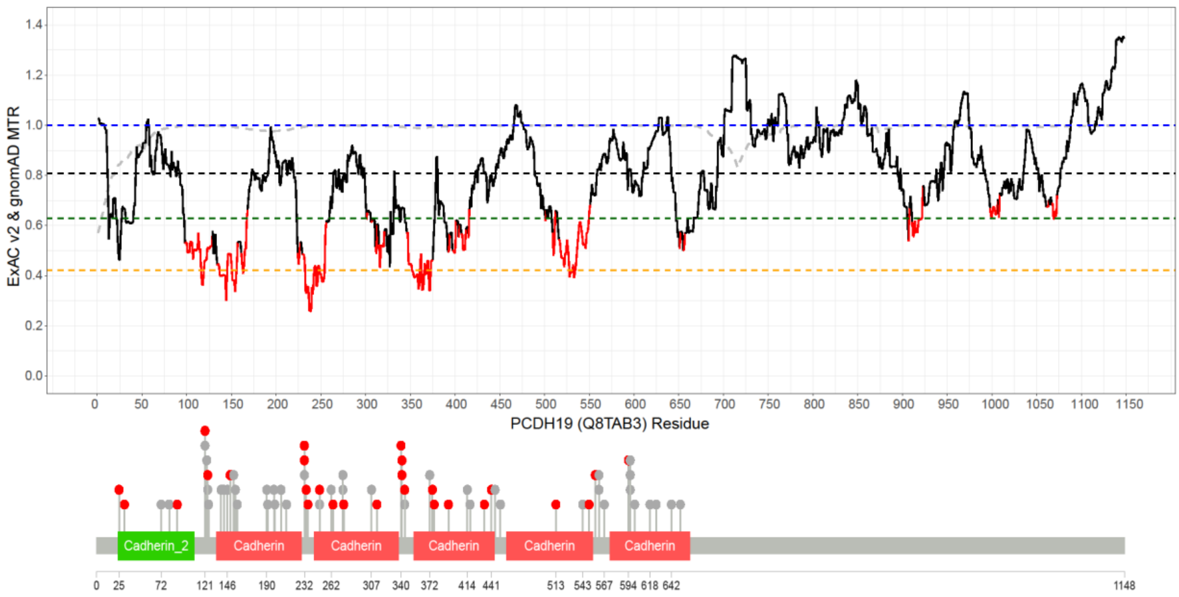
ExAC v2 & gnomAD MTR
PCDH19 (Q8TAB3) Residue
Cadherin_2
Cadherin
0 25 72 121 146 190 232 262 307 340 372 414 441 513 543 567 594 618 642 1148

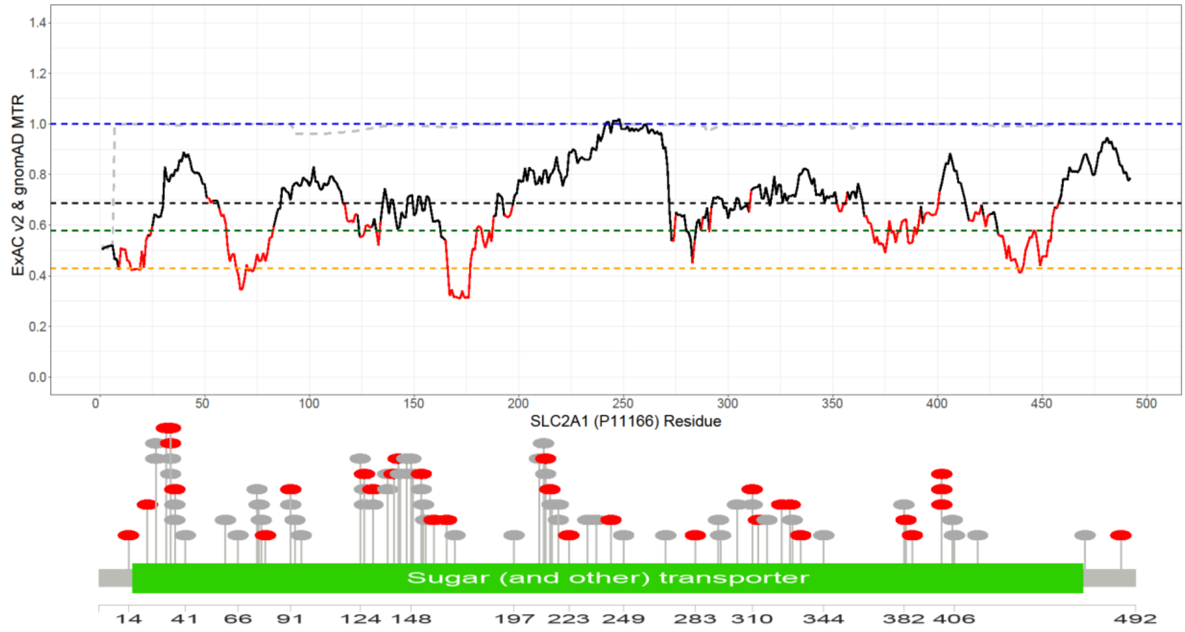
ExAC v2 & gnomAD MTR
SLC2A1 (P11166) Residue
Sugar (and other) transporter
14 41 66 91 124 148 197 223 249 283 310 344 382 406 492

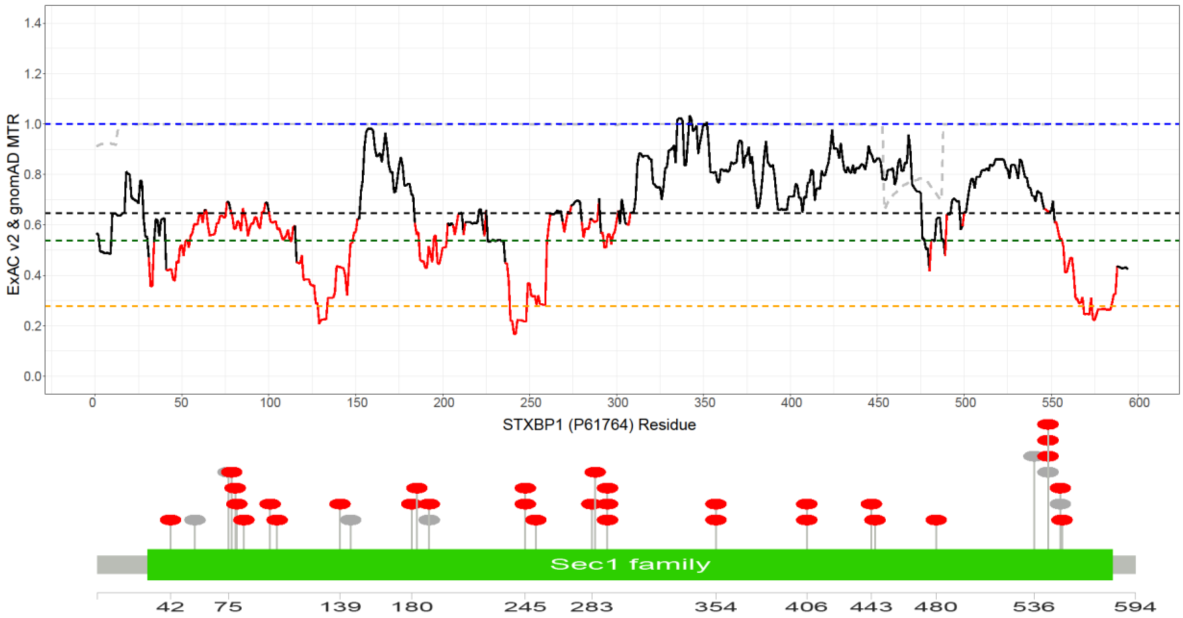
ExAC v2 & gnomAD MTR
STXBP1 (P61764) Residue
Sec1 family
42 75 139 180 245 283 354 406 443 480 536 594

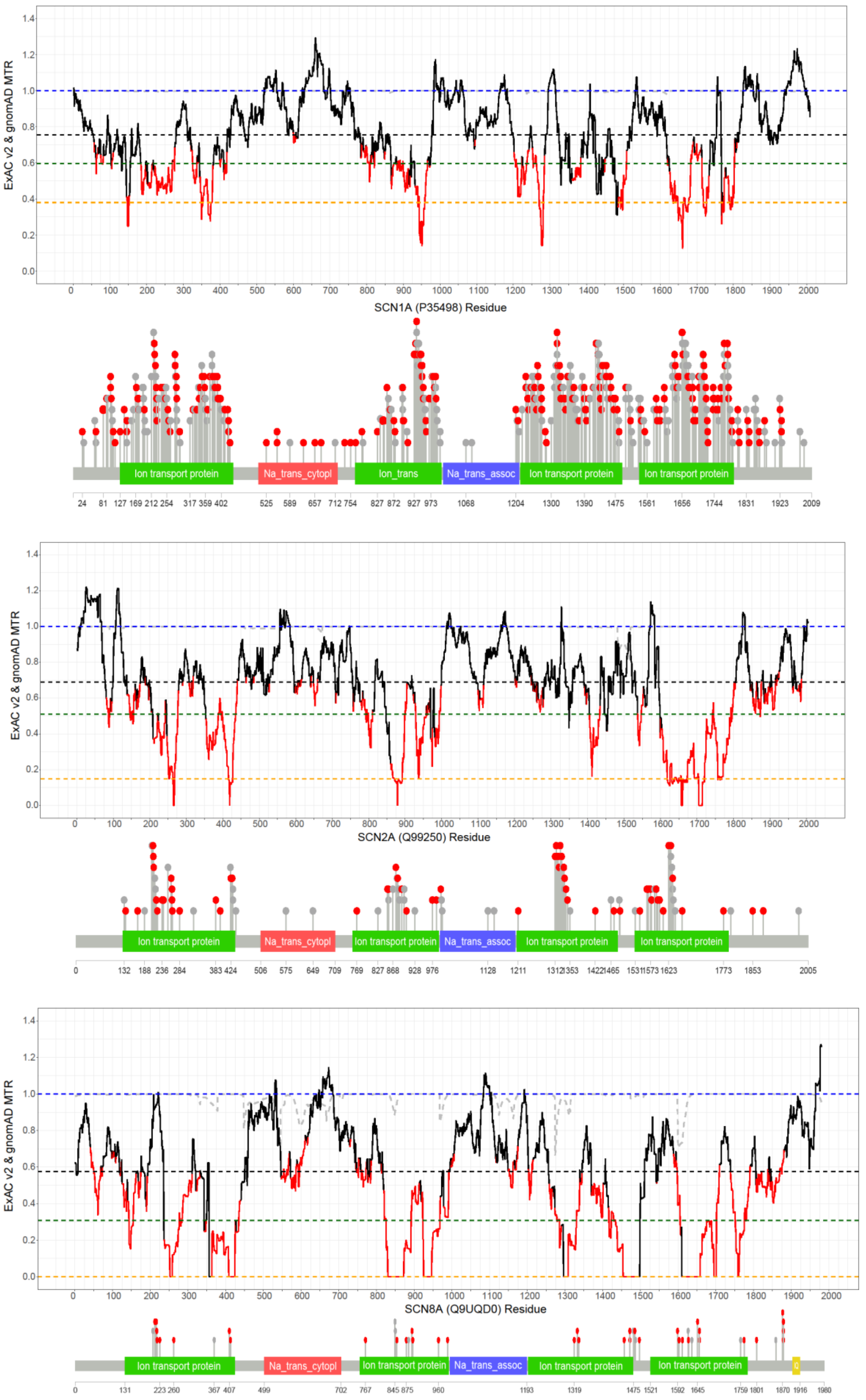

ExAC v2 & gnomAD MTR
SCN1A (P35498) Residue
Ion transport protein
Na_trans_cytopl
Ion_trans
Na_trans_assoc
Ion transport protein
Ion transport protein
24 81 127 169 212 254 317 359 402 525 589 657 712 754 827 872 927 973 1068 1204 1300 1390 1475 1561 1656 1744 1831 1923 2009
ExAC v2 & gnomAD MTR
SCN2A (Q99250) Residue
Ion transport protein
Na_trans_cytopl
Ion transport protein
Na_trans_assoc
Ion transport protein
Ion transport protein
0 132 188 236 284 383 424 506 575 649 709 769 827 868 928 976 1128 1211 1312 1353 1422 1465 1531 1573 1623 1773 1853 2005
ExAC v2 & gnomAD MTR
SCN8A (Q9UQD0) Residue
Ion transport protein
Na_trans_cytopl
Ion transport protein
Na_trans_assoc
Ion transport protein
Ion transport protein
0 131 223 260 367 407 499 702 767 845 875 960 1193 1319 1475 1521 1592 1645 1759 1801 1870 1916 1980

Supplemental Fig. S2: ExAC v1 (release 0.3.1) MTR plots for the eleven epilepsy genes

Provided as a separate file – MTR plots for *CDKL5* (**A**), *GRIN2A* (**B**), *KCNQ2* (**C**), *KCNT1* (**D**), *LGI1* (**E**), *PCHD19* (**F**), *SCN1A* (**G**), *SCN2A* (**H**), *SCN8A* (**I**), *SLC2A1* (**J**), and *STXBP1* (**K**). Regions in red achieved a study-wide FDR < 0.05 (**Supplemental Data S3**). MTR = 1 is depicted by the dashed blue line. Multiple gene-specific estimates are also depicted, including a gene's median MTR (black dashed line), 25th percentile MTR (dark green dashed line) and 5th percentile lowest MTR estimates (orange dashed line). The grey dashed line reflects how well that region of the gene was covered in the ExAC v1 sample data by showing the proportion of all ExAC v1 samples that achieved at least 10-fold coverage at the sites relevant to that codon (Methods).

Supplemental Fig. S3: ExAC v1 MTR estimates comparing the pathogenic (red) to the ExAC v2 Control Group 2 (blue) missense variants distributions

Provided as a separate file – presents the qualified pathogenic (red) and the ExAC v2 Control Group 2 (blue) missense variant distributions for the ExAC v1 MTR estimates. Figures include the median MTR estimate for all possible missense variants in the gene (grey dashed line), the 25th percentile MTR estimate (orange dashed line), and the 5th percentile MTR estimate (red dashed line). The position of the median ExAC v1 MTR estimate for the qualified pathogenic variants (red full line) and median for the Control Group 2 (blue full line) are also included. Distributions provided for *CDKL5* (**A**), *GRIN2A* (**B**), *KCNQ2* (**C**), *KCNT1* (**D**), *LGI1* (**E**), *PCHD19* (**F**), *SCN1A* (**G**), *SCN2A* (**H**), *SCN8A* (**I**), *SLC2A1* (**J**), and *STXBP1* (**K**).

Supplemental Fig. S4: Correlation matrix of the evaluated bioinformatic tools (features); including the two additional MTR features

Each cell represents the Pearson's *r* correlation coefficient.

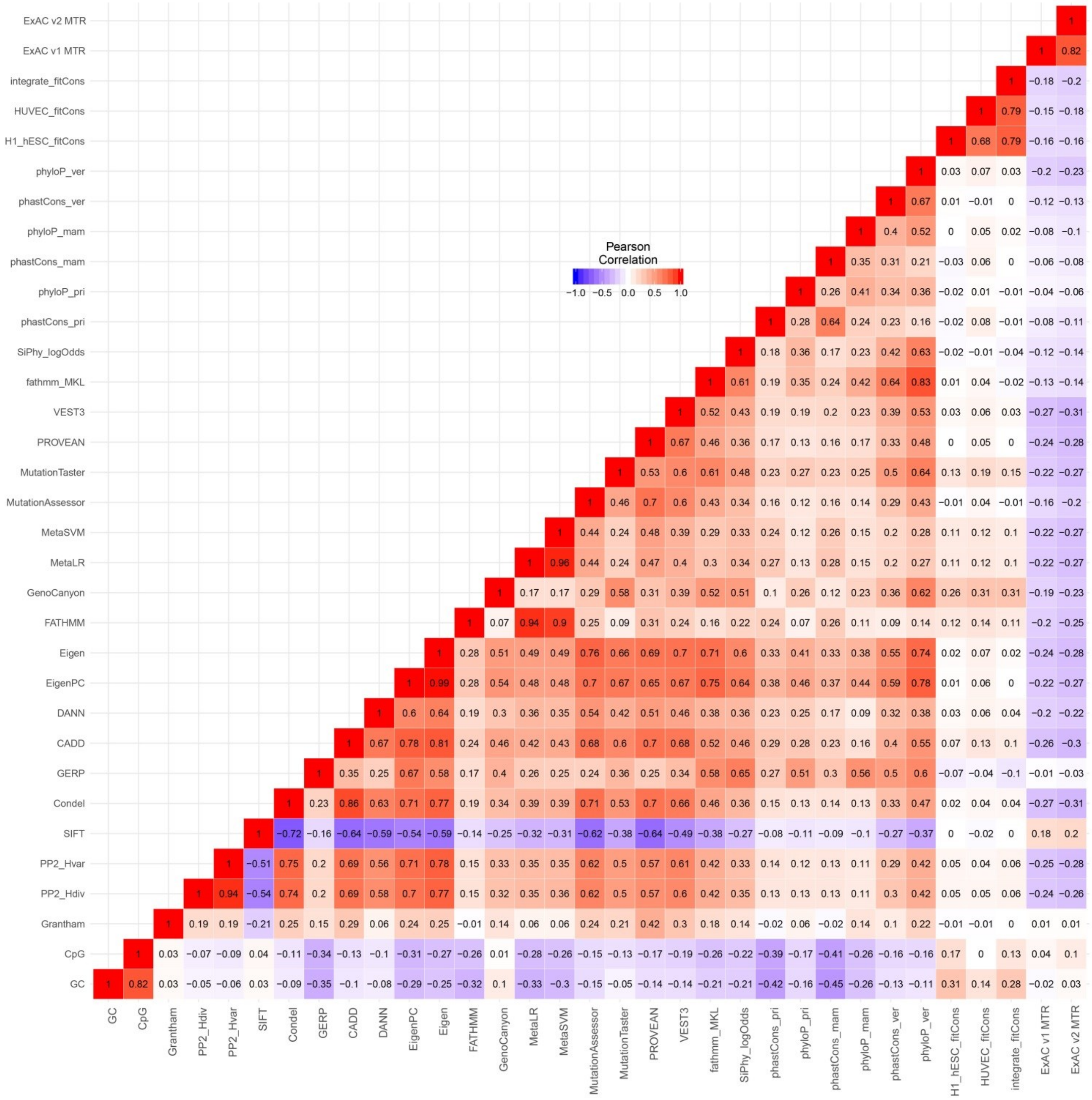

Supplemental Fig. S5: Histogram distribution for all features across all genes

Provided as a separate file – contains the entire set of score distributions for all evaluated bioinformatic tools across each of the eleven evaluated genes. The median rankscore of all possible missense variants (grey line) is illustrated relative to three mutually exclusive control population distributions: Control group 1 (ExAC v1 singletons; blue line), Control group 2 (ExAC v2 singletons not in ExAC v1; dashed blue line) and Control group 3 (ExAC v2 variants with a MAF<0.05% and not in Control groups 1 or 2; dashed light blue line). The median rankscore for two case-ascertained missense variant distributions is also indicated, representing the 606 qualified pathogenic variants (red line), and the additional 437 unqualified pathogenic-reported variants (dashed red line).

Supplemental Fig. S6: Receiver Operating Characteristic (ROC) curves for the six genes with multivariate regression models, including individual features contributing to that model

Provided as a separate file – accompanying the ROC curves for each gene is the AUC for model predictions applied on the training and separately on the test samples that did not contribute to model fitting. ROC curves for *CDKL5* (**A** and **B**), *KCNQ2* (**C** and **D**), *PCHD19* (**E** and **F**), *SCN1A* (**G** and **H**), *SCN2A* (**I** and **J**), and *SCN8A* (**K** and **L**).

Supplemental Fig. S7: Variant Gene-specific Probability of Pathogenicity (GPP) score distributions, per gene

Violin plots present the distribution of Gene-specific Probability of Pathogenicity (GPP) score for *CDKL5* (**A**), *KCNQ2* (**B**), *PCDH19* (**C**), *SCN1A* (**D**), *SCN2A* (**E**) and *SCN8A* (**F**).

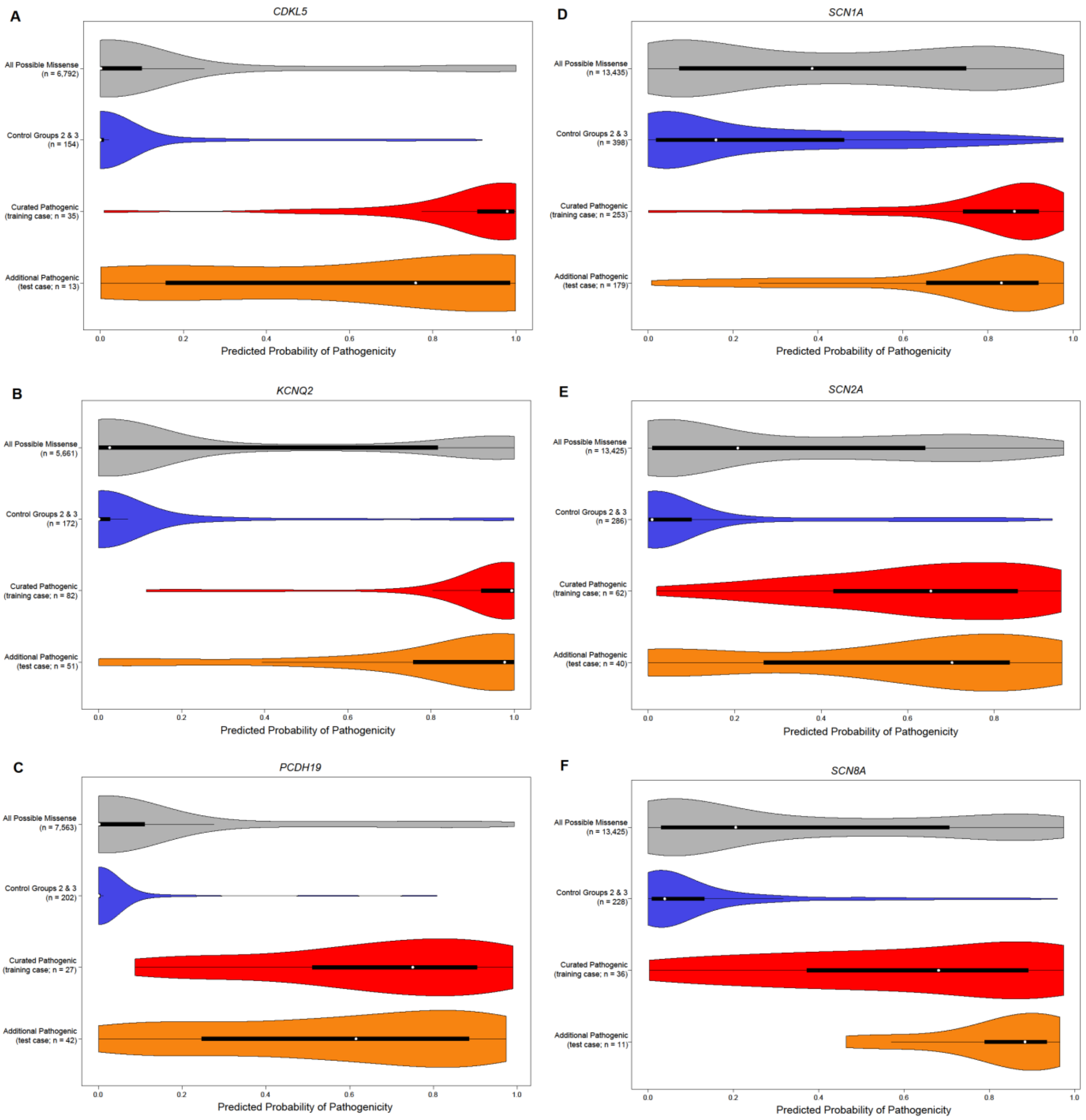

Supplemental Fig. S8: Variant GPP probability of pathogenicity score distribution after fitting the model with the unqualified case variants and the ExAC v2 control missense variants

This plot accompanies Figure 5. It displays the pooled collection of variants from the six genes with a gene-customized model. It differs to Figure 5 by replacing the training data with the test data and recalculating model predictions.

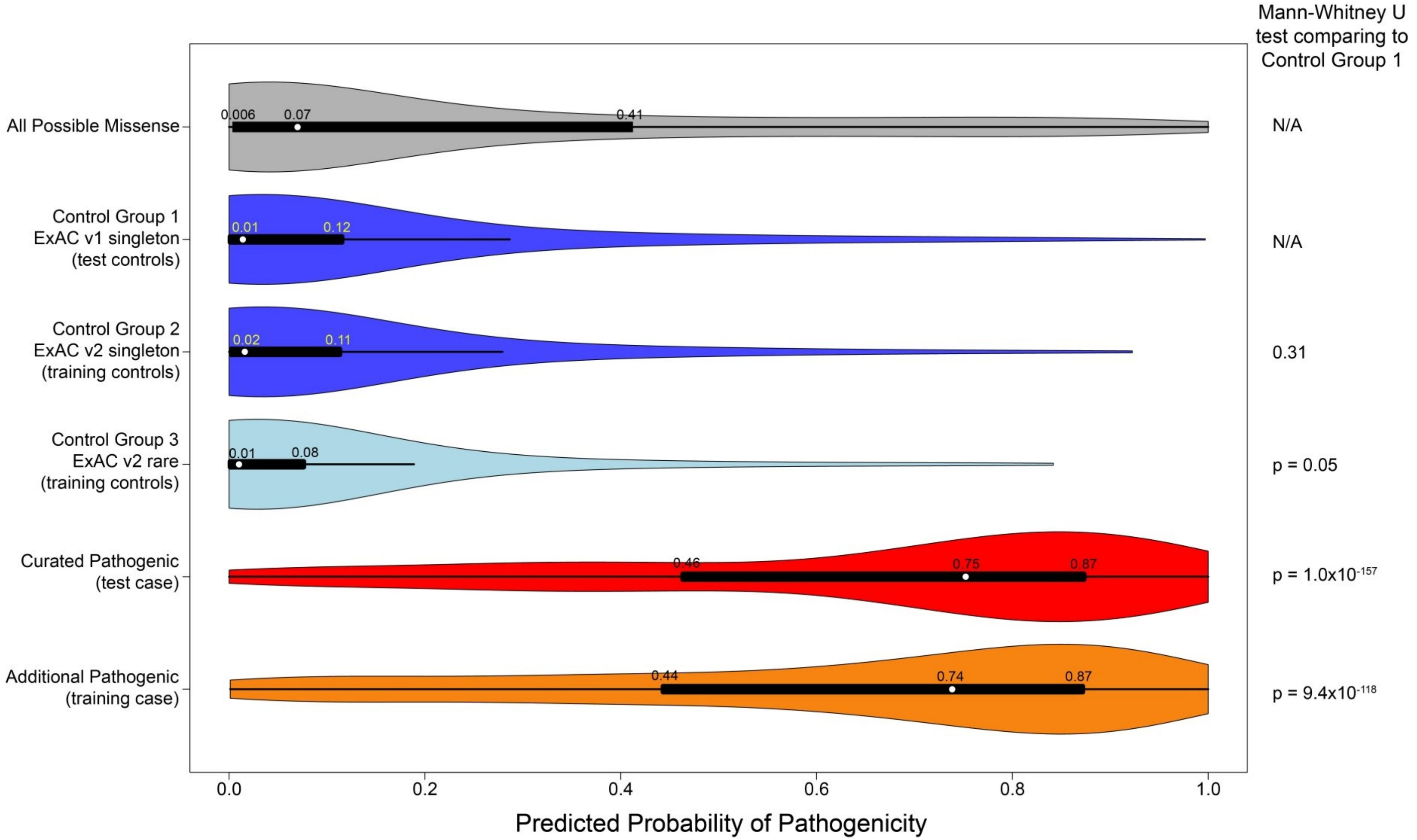

Supplemental Fig. S9: Comparing GPP to MPC predictions for the six epilepsy genes

Samocha et al. (2017 preprint) introduce a new missense variant score, referred to as MPC by the authors (MPC and component scores are available at: ftp.broadinstitute.org/pub/ExAC_release/release1/regional_missense_constraint/). The MPC combines a regional missense constraint score (variable obs_exp) with variant level information (PolyPhen-2 and "missense badness"). For all possible missense variants in the six epilepsy genes with a GPP we found low correlation between MPC and GPP (Pearson's r^2 = 0.329). Directly comparing how well the two scores predict the 336 test-set pathogenic missense variants from the 827 Control Group 2 missense variants found—across these six epilepsy genes—the GPP achieves a superior AUC of 0.91, (95% CI, 0.89 – 0.93) compared to the MPC AUC of 0.81, (95% CI, 0.79 – 0.83); (DeLong's test for two correlated ROC curves p = 4.9×10^{-17}). This evaluation is restricted to the six epilepsy genes with GPP scores.

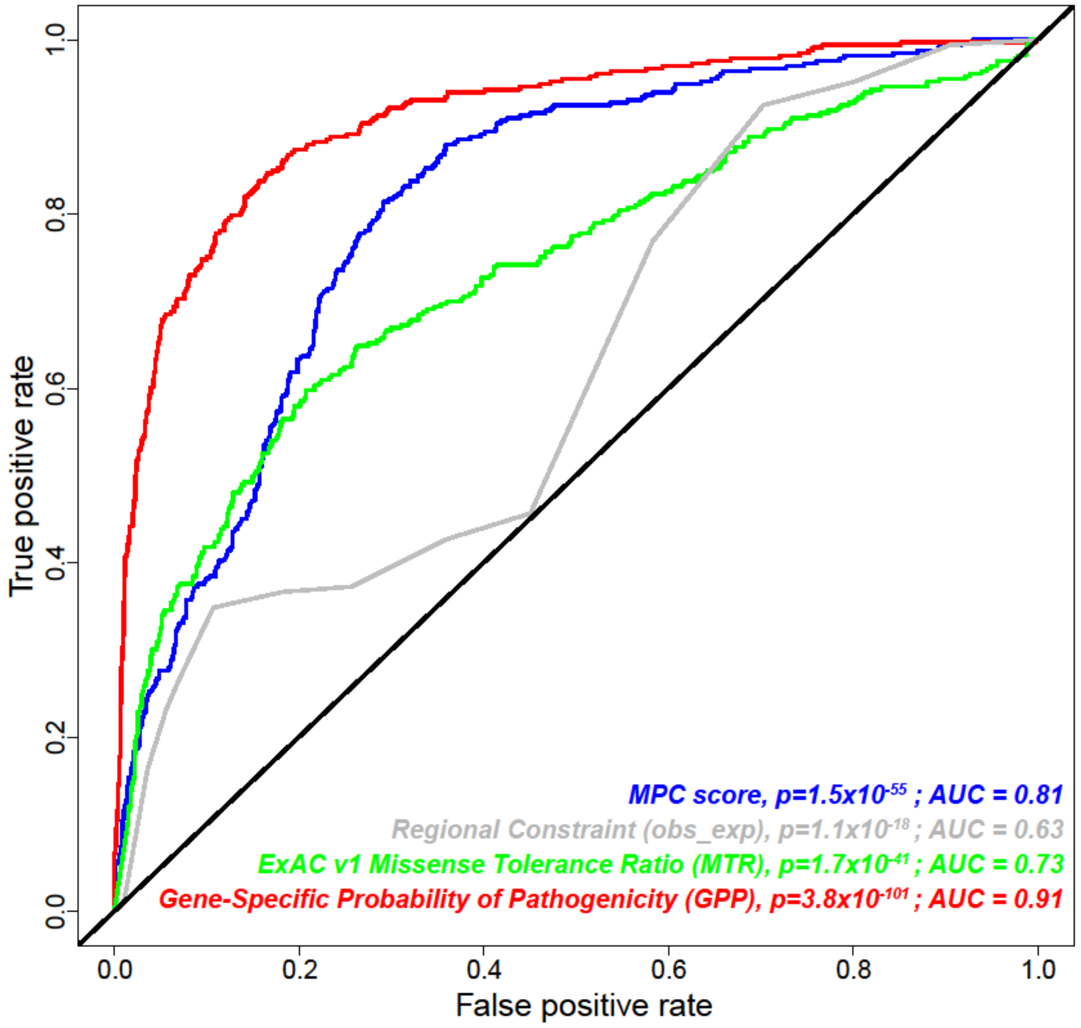

Supplemental Fig. S10: Gene-specific Probability of Pathogenicity (GPP) score distribution after fitting the model with the combined eleven gene data rather than a gene-customized approach

Provided as a separate file. This figure reproduces the model building process applied to individual genes on the pooled collection of data across the eleven epilepsy genes. (**A**) The Boruta assessment evaluating the predictive utility of the individual features in discriminating the 606 qualified pathogenic missense variants from the Control Group 1 variants. (**B**) The forward-backward selection adding features to the model until the AIC suggested that the addition of new features no longer significantly improved the model. (**C**) The resulting logistic regression model for the combined gene (global) fitted model. (**D**) Gene-specific Probability of Pathogenicity (GPP) score distribution after applying the combined-gene (global) model that was fitted to the collection of training variants from the eleven epilepsy genes.

Supplemental Fig. S11: Correlation of MTR with log(dN/dS) using the ExAC v1 sliding windows

The sliding windows from the eleven epilepsy genes were adopted (**Supplemental Data S3**). Due to singularity with $log(d_N/d_S)$ at either $D_n = 0$ or $D_s = 0$, the plot depicts the 92% of windows with $D_n > 0$ and $D_s > 0$.

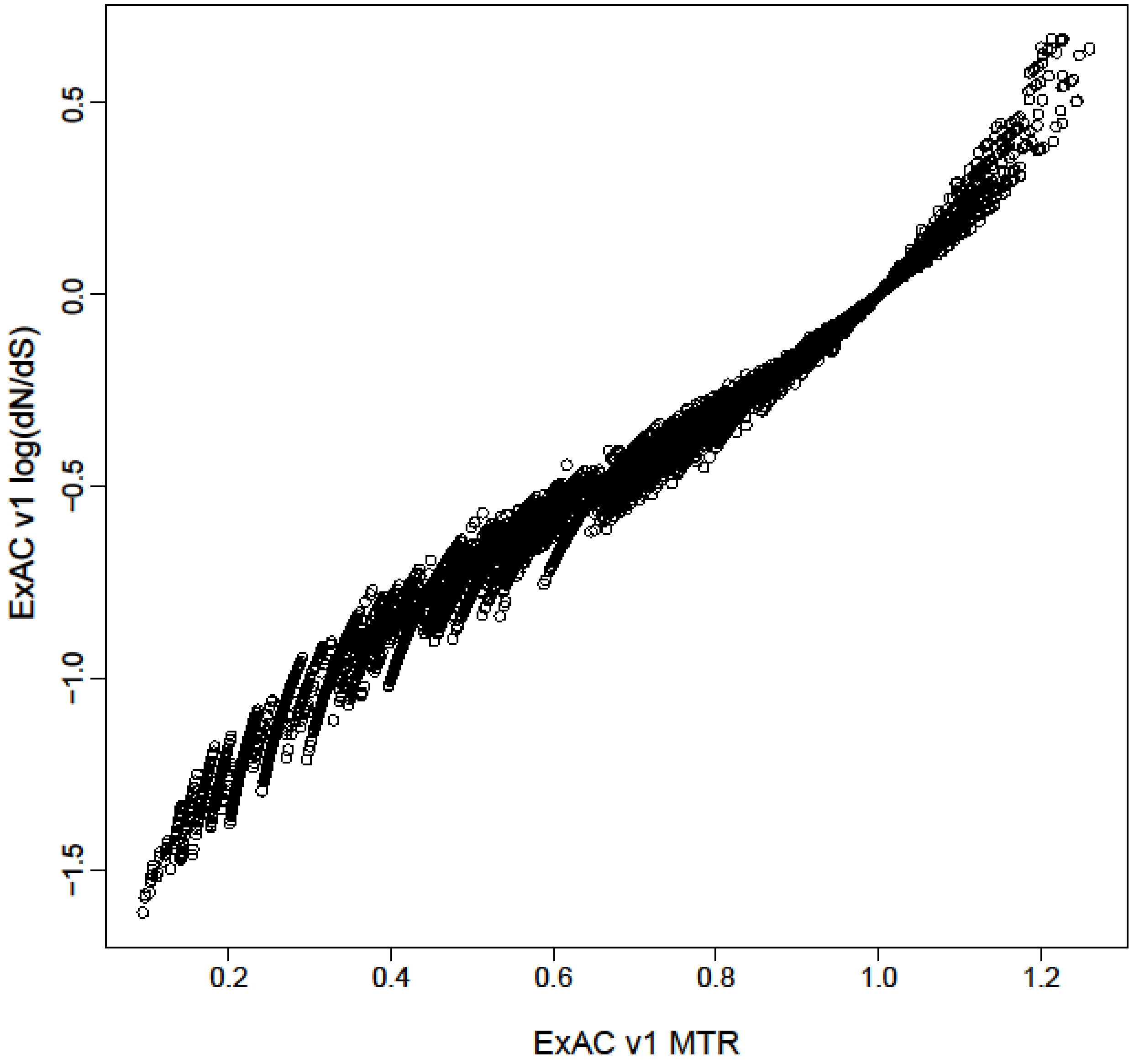

Supplemental Fig. S12: Comparison of MTR with subRVIS and regional constraint (obs_exp) estimates

Across these 11 epilepsy genes the MTR estimates (Y-axis) are compared to the distribution of **(A)** subRVIS(CDD), **(B)** subRVIS(exon) and **(C)** obs_exp (Samocha et al. [preprint] regional constraint) estimates.

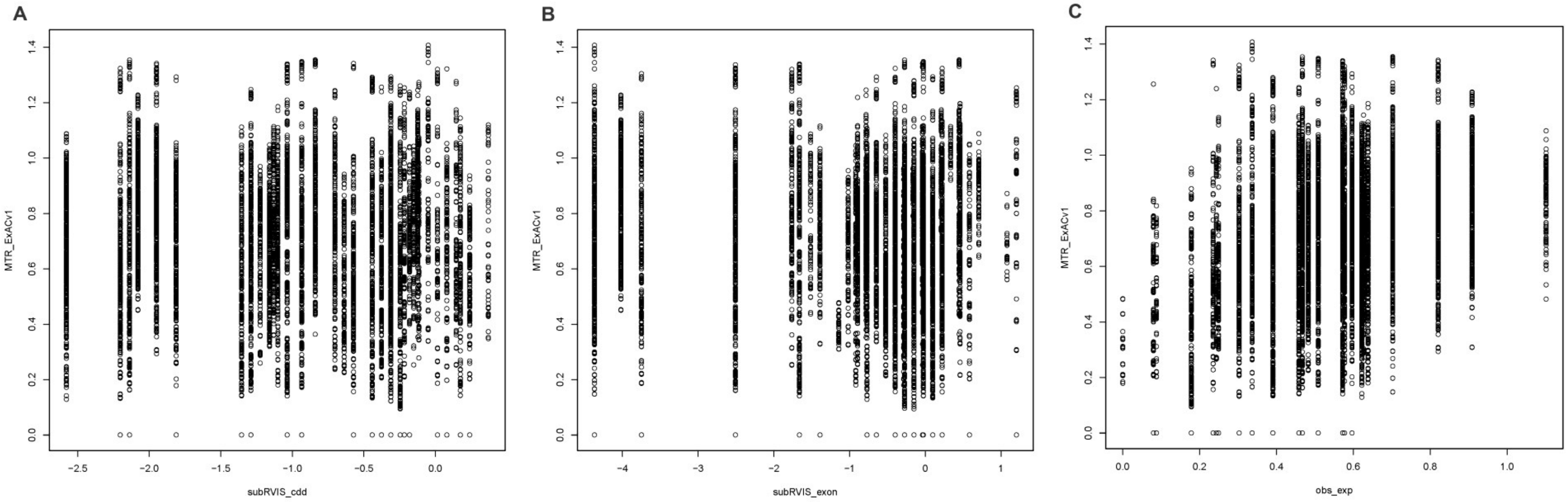

Pearson's r^2 = 0.008 Pearson's r^2 = 0.009 Pearson's r^2 = 0.153

Supplemental Table S1: Breakdown for the curation of the eleven epilepsy genes

Qualification	ClinVar	HGMD	Union (distinct)
Number of variants described in the ClinVar and/or HGMD variant databases	1,570	1,600	2,702
Number of variants classified as pathogenic	631	1,416	1,605
Number of variant effects annotated as “missense” in the canonical transcript	443	988	1,104
Number of variants with an epilepsy-related keyword among the described phenotype	435	939	1,056
Number of variants with “Pathogenic” classification when present among ClinVar and / or HGMD databases (**See Supplemental Table S2**)	422	855	1,043
Final tallies after confirming segregation support based on the associated literature (**See Supplemental Table S2**)	256	531	**606**

Supplemental Table S2: Summary of pathogenic classified variants among eleven epilepsy genes

Gene	HGMD®	ClinVar	Distinct	Qualified Tally [% of overall Distinct]
SCN1A	402	103	**432**	**253 [59%]**
KCNQ2	96	85	**133**	**82 [62%]**
SCN2A	60	63	**102**	**62 [61%]**
SLC2A1	72	26	**84**	**31 [37%]**
PCDH19	55	21	**69**	**27 [39%]**
CDKL5	32	37	**48**	**35 [73%]**
SCN8A	32	26	**47**	**36 [77%]**
GRIN2A	28	20	**39**	**13 [33%]**
STXBP1	28	21	**38**	**31 [82%]**
LGI1	27	10	**27**	**16 [59%]**
KCNT1	23	10	**24**	**20 [83%]**
**Total**	**855**	**422**	**1,043**	**606 [58%]**

Summarizing the number of pathogenic reported variants among eleven epilepsy genes where at least 20 distinct pathogenic variants were reported among ClinVar and/or HGMD databases.

Supplemental Table S3: Per gene summaries of the standing variation data found among the combined ExAC v2 and gnomAD population sample of 138,632 samples

Gene	CCDS Transcript	CCDS length protein-coding bases	CCDS SNVs protein-coding (% of possible)	Pass Database Quality Filter (% of observed)	Have synonymous or missense effects (% of observed)
CDKL5	CCDS14186.1	3,093	466 (5.0%)	417 (89.5%)	414 (88.8 %)
GRIN2A	CCDS10539.1	4,395	1,175 (8.9%)	1,070 (91.1%)	1,064 (90.6%)
KCNQ2	CCDS13520.1	2,619	765 (9.7%)	509 (66.5%)	508 (66.4%)
KCNT1	CCDS35175.2	3,708	1,330 (12.0%)	985 (74.1%)	974 (73.2%)
LGI1	CCDS7431.1	1,674	303 (6.0%)	275 (90.8%)	273 (90.1%)
PCDH19	CCDS55462.1	3,447	647 (6.3%)	535 (82.7%)	535 (82.7%)
SCN1A	CCDS54413.1	6,030	1,088 (6.0%)	1,020 (93.8%)	1,018 (93.6%)
SCN2A	CCDS33314.1	6,018	988 (5.5%)	871 (88.2%)	869 (88.0%)
SCN8A	CCDS44891.1	5,943	951 (5.3%)	789 (83.0%)	786 (82.6%)
SLC2A1	CCDS477.1	1,479	359 (8.1%)	299 (83.3%)	299 (83.3%)
STXBP1	CCDS35146.1	1,785	306 (5.7%)	260 (85.0%)	260 (85.0%)

Supplemental Table S4: List of the 31 tools (predictors / features) evaluated on the eleven genes

Database Source	Description	Feature ID in this paper
PolyPhen-2	PolyPhen-2 HumDiv score	PP2_Hdiv
PolyPhen-2	PolyPhen-2 HumVar score	PP2_Hvar
Ve!P Ensembl r87	SIFT score	SIFT
Ve!P Ensembl r87	Condel score	Condel
CADD v1.3	GC (%GC in a window of +/-75bp)	GC
CADD v1.3	CpG (%CpG in a window of +/-75bp)	CpG
CADD v1.3	Grantham score	Grantham
CADD v1.3	phastCons primate score	phastCons_pri
CADD v1.3	phyloP primate score	phyloP_pri
dbNSFP 3.2c	GERP++ RS rankscore	GERP_rs
dbNSFP 3.2c	CADD raw rankscore	CADD_rs
dbNSFP 3.2c	DANN rankscore	DANN_rs
dbNSFP 3.2c	Eigen-PC-raw rankscore^	EigenPC_rs
dbNSFP 3.2c	Eigen-raw rankscore^	Eigen_rs
dbNSFP 3.2c	FATHMM converted rankscore	FATHMM_rs
dbNSFP 3.2c	GenoCanyon score rankscore	GenoCanyon_rs
dbNSFP 3.2c	MetaLR rankscore	MetaLR_rs
dbNSFP 3.2c	MetaSVM rankscore	MetaSVM_rs
dbNSFP 3.2c	MutationAssessor score rankscore	MutationAssessor_rs
dbNSFP 3.2c	MutationTaster converted rankscore	MutationTaster_rs
dbNSFP 3.2c	PROVEAN converted rankscore	PROVEAN_rs
dbNSFP 3.2c	VEST 3.0 rankscore	VEST3_rs
dbNSFP 3.2c	fathmm-MKL coding rankscore	fathmm_MKL_rs
dbNSFP 3.2c	SiPhy 29way logOdds rankscore	SiPhy_logOdds_rs
dbNSFP 3.2c	phastCons20way mammalian rankscore	phastCons_mam_rs
dbNSFP 3.2c	phyloP20way mammalian rankscore	phyloP_mam_rs
dbNSFP 3.2c	phastCons100way vertebrate rankscore	phastCons_ver_rs
dbNSFP 3.2c	phyloP100way vertebrate rankscore	phyloP_ver_rs
dbNSFP 3.2c	H1-hESC fitCons score rankscore^	H1_hESC_fitCons_rs
dbNSFP 3.2c	HUVEC fitCons score rankscore^	HUVEC_fitCons_rs
dbNSFP 3.2c	integrated fitCons score rankscore^	integrate_fitCons_rs

^Scores were unavailable for these five tools on the X chromosome genes *CDKL5* and *PCDH19*.

Supplemental Table S5: List of the features found by the Boruta algorithm to be uninformative, informative/tentative, and highly informative for each studied gene

Gene	Uninformative	Informative / Tentative	Highly Informative
CDKL5	15. phastCons<br>16. GenoCanyon<br>17. Grantham<br>18. phyloP_mam	7. GC<br>8. PP2_Hdiv<br>9. SIFT<br>10. phyloP_pri<br>11. DANN<br>12. SiPhy_logOdds<br>13. GERP<br>14. fathmm_MKL	**1. VEST3**<br>**2. MutationAssessor**<br>**3. PROVEAN**<br>4. MutationTaster<br>5. CADD<br>6. FATHMM
GRIN2A	10. H1_hESC_fitCons<br>11. HUVEC_fitCons<br>12. GERP<br>13. PP2_Hdiv<br>14. FATHMM<br>15. phyloP_pri<br>16. fathmm_MKL<br>17. MutationTaster<br>18. Grantham<br>19. phyloP_mam<br>20. GC	1. CADD<br>2. SIFT<br>3. VEST3<br>4. MutationAssessor<br>5. PROVEAN<br>6. GenoCanyon<br>7. SiPhy_logOdds<br>8. phastCons<br>9. DANN	
KCNQ2	19. phastCons<br>20. Grantham	9. GC<br>10. CADD<br>11. PP2_Hdiv<br>12. FATHMM<br>13. SiPhy_logOdds<br>14. GenoCanyon<br>15. phyloP_pri<br>16. DANN<br>17. fathmm_MKL<br>18. phyloP_mam	**1. VEST3**<br>**2. PROVEAN**<br>**3. MutationAssessor**<br>4. H1_hESC_fitCons<br>5. HUVEC_fitCons<br>6. SIFT<br>7. GERP<br>8. MutationTaster
KCNT1	11. HUVEC_fitCons<br>12. FATHMM<br>13. GenoCanyon<br>14. phyloP_pri<br>15. H1_hESC_fitCons<br>16. phyloP_mam<br>17. fathmm_MKL<br>18. phastCons<br>19. Grantham<br>20. GC	1. VEST3<br>2. DANN<br>3. CADD<br>4. MutationAssessor<br>5. SIFT<br>6. PP2_Hdiv<br>7. PROVEAN<br>8. MutationTaster<br>9. SiPhy_logOdds<br>10. GERP	
LGI1	8. SiPhy_logOdds<br>9. DANN<br>10. GenoCanyon<br>11. MutationTaster<br>12. H1_hESC_fitCons<br>13. phastCons<br>14. phyloP_pri<br>15. GERP<br>16. GC	6. CADD<br>7. MutationAssessor	**1. VEST3**<br>2. SIFT<br>**3. PROVEAN**<br>4. PP2_Hdiv<br>5. Grantham

	17. fathmm_MKL<br>18. FATHMM<br>19. phyloP_mam<br>20. HUVEC_fitCons		
PCDH19	9. GC<br>10. MutationTaster<br>11. Grantham<br>12. DANN<br>13. fathmm_MKL<br>14. phyloP_pri<br>15. GenoCanyon<br>16. phyloP_mam<br>17. phastCons<br>18. SiPhy_logOdds	4. FATHMM<br>5. SIFT<br>6. PP2_Hdiv<br>7. CADD<br>8. GERP	**1. MutationAssessor**<br>**2. VEST3**<br>3. **PROVEAN**
SCN1A	15. DANN<br>16. phyloP_pri<br>17. HUVEC_fitCons<br>18. GC<br>19. phyloP_mam<br>20. phastCons	10. GenoCanyon<br>11. GERP<br>12. H1_hESC_fitCons<br>13. SiPhy_logOdds<br>14. Grantham	**1. VEST3**<br>2. FATHMM<br>**3. MutationAssessor**<br>4. CADD<br>5. SIFT<br>**6. PROVEAN**<br>7. MutationTaster<br>8. PP2_Hdiv<br>9. fathmm_MKL
SCN2A	14. phyloP_mam<br>15. SiPhy_logOdds<br>16. phyloP_pri<br>17. Grantham<br>18. phastCons<br>19. GC<br>20. H1_hESC_fitCons	5. fathmm_MKL<br>6. SIFT<br>7. MutationTaster<br>8. DANN<br>9. GERP<br>10. CADD<br>11. PROVEAN<br>12. HUVEC_fitCons<br>13. GenoCanyon	1. FATHMM<br>**2. VEST3**<br>3. PP2_Hdiv<br>**4. MutationAssessor**
SCN8A	13. DANN<br>14. MutationTaster<br>15. SiPhy_logOdds<br>16. GenoCanyon<br>17. phyloP_mam<br>18. fathmm_MKL<br>19. Grantham<br>20. HUVEC_fitCons	3. FATHMM<br>4. SIFT<br>5. CADD<br>6. MutationAssessor<br>7. PROVEAN<br>8. H1_hESC_fitCons<br>9. PP2_Hdiv<br>10. phyloP_pri<br>11. GERP<br>12. GC	**1. VEST3**<br>2. phastCons
SLC2A1	11. fathmm_MKL<br>12. SiPhy_logOdds<br>13. GERP<br>14. H1_hESC_fitCons<br>15. phastCons<br>16. FATHMM<br>17. GenoCanyon<br>18. HUVEC_fitCons<br>19. phyloP_pri<br>20. GC	2. CADD<br>3. PP2_Hdiv<br>4. VEST3<br>5. SIFT<br>6. phyloP_mam<br>7. PROVEAN<br>8. Grantham<br>9. MutationTaster<br>10. DANN	**1. MutationAssessor**
STXBP1	9. Grantham<br>10. SiPhy_logOdds	5. FATHMM<br>6. PP2_Hdiv	**1. VEST3**<br>**2. MutationAssessor**

	11. CADD<br>12. GERP<br>13. phastCons<br>14. DANN<br>15. phyloP_pri<br>16. GC<br>17. H1_hESC_fitCons<br>18. phyloP_mam<br>19. HUVEC_fitCons<br>20. GenoCanyon	7. fathmm_MKL<br>8. MutationTaster	**3. PROVEAN**<br>4. SIFT

Within each category the tools are further sorted from highest to lowest median z-scores.

Supplemental Table S6: Summary of the top feature distribution for the nine epilepsy genes with at least one highly informative feature

Gene	Top-ranked feature	Gene-wide	Control Group 1: ExACv1 (training)	Control Group 2: ExACv2 (test)	Control Group 3: ExACv2 (MAF<0.05)	Qual. Case (training)	Non-Qual Case (test)	MWU p-value (*ExACv2 vs Non-Qual Case*)	MWU p-value (*ExACv2 vs ExACv1*)
CDKL5	VEST3	6792; 0.504 [0.24 - 0.73]	104; 0.302 [0.11 - 0.63]	79; 0.336 [0.14 - 0.68]	75; 0.221 [0.09 - 0.55]	35; 0.973 [0.89 - 0.99]	13; 0.963 [0.93 - 0.99]	$1.4x10^{-6}$	0.29
KCNQ2	VEST3	5661; 0.668 [0.45 - 0.83]	98; 0.616 [0.43 - 0.73]	89; 0.535 [0.30 - 0.72]	83; 0.541 [0.28 - 0.72]	82; 0.892 [0.81 - 0.95]	51; 0.853 [0.75 - 0.93]	$9.4x10^{-12}$	0.22
LGI1	VEST3	3690; 0.697 [0.47 - 0.85]	67; 0.489 [0.29 - 0.68]	53; 0.528 [0.33 - 0.71]	40; 0.468 [0.33 - 0.69]	16; 0.972 [0.96 - 0.99]	11; 0.982 [0.97 - 0.99]	$1.4x10^{-6}$	0.58
PCDH19	Mutation Assessor	7563; 0.323 [0.13 - 0.72]	125; 0.298 [0.13 - 0.67]	117; 0.251 [0.13 - 0.50]	85; 0.225 [0.11 - 0.50]	27; 0.982 [0.92 - 0.99]	42; 0.972 [0.92 - 0.99]	$3.3x10^{-17}$	0.07
SCN1A	VEST3	13435; 0.74 [0.53 - 0.89]	207; 0.596 [0.34 - 0.79]	229; 0.630 [0.41 - 0.79]	169; 0.625 [0.31 - 0.77]	253; 0.965 [0.91 - 0.99]	179; 0.944 [0.90 - 0.98]	$6.3x10^{-39}$	0.40
SCN2A	FATHMM	13425; 0.97 [0.96 - 0.98]	190; 0.962 [0.91 - 0.97]	188; 0.964 [0.91 - 0.97]	98; 0.961 [0.91 - 0.97]	62; 0.984 [0.98 - 0.99]	40; 0.984 [0.97 - 0.99]	$8.6x10^{-7}$	0.48
SCN8A	VEST3	13245; 0.74 [0.52 - 0.88]	167; 0.586 [0.35 - 0.73]	125; 0.562 [0.36 - 0.77]	103; 0.560 [0.37 - 0.71]	36; 0.821 [0.74 - 0.93]	11; 0.937 [0.89 - 0.95]	$1.1x10^{-5}$	0.69
SLC2A1	Mutation Assessor	3188; 0.670 [0.34 - 0.92]	50; 0.403 [0.21 - 0.80]	52; 0.426 [0.19 - 0.69]	51; 0.463 [0.22 - 0.73]	31; 0.973 [0.89 - 0.99]	53; 0.943 [0.76 - 0.98]	$2.9x10^{-10}$	0.52
STXBP1	VEST3	3939; 0.733 [0.55 - 0.87]	46; 0.592 [0.47 - 0.74]	55; 0.689 [0.39 - 0.78]	27; 0.627 [0.31 - 0.73]	31; 0.975 [0.96 - 0.99]	7; 0.945 [0.84 - 0.95]	$3.6x10^{-10}$	0.60

The individual cells represent the total number of variants; the median rankscore of the corresponding variant distribution accompanied by the lower and upper quantile estimates of their distribution [Q1 - Q3]. MWU: Mann-Whitney U test

Supplemental Table S7: Akaike information criterion (AIC) comparisons across growing logistic regression models, per gene

Gene	Data	Feature 1	Feature 2	Feature 3	Feature 4	Feature 5	Feature 6
CDKL5	Feature	**MTR ExAC v1**	**VEST3**	**Mutation Assessor**	PROVEAN		
	AIC	**100.36**	**44.537**	**35.657**	34.433		
	p-value	**N/A**	**$p=7.6x10^{-13}$**	**p=0.012**	p=0.542		
GRIN2A	Feature	**CADD**	SIFT				
	AIC	**92.783**	87.234				
	p-value	**N/A**	p=0.062				
KCNQ2	Feature	**MTR ExAC v1**	**VEST3**	**PROVEAN**	**Mutation Assessor**	H1 hESC fitCons	
	AIC	**126.01**	**81.641**	**68.426**	**56.827**	56.353	
	p-value	**N/A**	**$p=2.3x10^{-10}$**	**p=0.001**	**p=0.003**	p=0.79	
KCNT1	Feature	**VEST3**	DANN				
	AIC	**122.49**	120.19				
	p-value	**N/A**	p=0.32				
LGI1	Feature	**VEST3**	SIFT				
	AIC	**21.271**	17.639				
	p-value	**N/A**	p=0.16				
PCDH19	Feature	**MTR ExAC v1**	**Mutation Assessor**	**VEST3**	**PROVEAN**	FATHMM	
	AIC	**136.55**	**90.366**	**81.272**	**67.384**	68.077	
	p-value	**N/A**	**$p=9.4x10^{-11}$**	**p=0.011**	**$p=9.6x10^{-4}$**	P=1	
SCN1A	Feature	**MTR ExAC v1**	**VEST3**	FATHMM			
	AIC	**533.26**	**348.37**	346.03			
	p-value	**N/A**	**$p=7.1x10^{-41}$**	p=0.31			
SCN2A	Feature	**MTR ExAC v1**	**FATHMM**	**VEST3**	**PolyPhen-2 HumDiv**	Mutation Assessor	
	AIC	**223.77**	**183.27**	**160.92**	**152.08**	153.47	
	p-value	**N/A**	**$p=1.6x10^{-9}$**	**$p=1.4x10^{-5}$**	**p=0.012**	p=1	
SCN8A	Feature	**MTR ExAC v1**	**VEST3**	phastCons primate			
	AIC	**126.23**	**104.68**	103.12			
	p-value	**N/A**	**$p=2.1x10^{-5}$**	p=0.46			
SLC2A1	Feature	**Mutation Assessor**	CADD				
	AIC	**84.088**	82.522				
	p-value	**N/A**	p=0.46				
STXBP1	Feature	**VEST3**	Mutation Assessor				
	AIC	**36.27**	33.695				
	p-value	**N/A**	p=0.28				

AIC = Akaike information criterion; MTR = Missense Tolerance Ratio

Supplemental Table S8: Final multivariate logistic regression models, per gene.

Gene	Data	Intercept	X_1	X_2	X_3	X_4	X_5
CDKL5	**Feature**	**N/A**	**MTR ExAC v1**	**VEST3**	**Mutation Assessor**		
	β	**-11.72**	**-5.66**	**13.35**	**7.35**		
	**p-value**	**p=0.0065**	**p=0.028**	**$p=6.4x10^{-4}$**	**p=0.016**		
KCNQ2	**Feature**	**N/A**	**MTR ExAC v1**	**VEST3**	**PROVEAN**	**Mutation Assessor**	
	β	**-9.70**	**-11.65**	**7.64**	**6.31**	**8.40**	
	**p-value**	**$p=2.7x10^{-4}$**	**$p=4.7x10^{-7}$**	**p=0.003**	**p=0.01**	**$p=6.6x10^{-4}$**	
PCDH19	**Feature**	**N/A**	**MTR ExAC v1**	**Mutation Assessor**	**VEST3**	**PROVEAN**	
	β	**-11.81**	**-3.77**	**3.27**	**4.84**	**9.62**	
	**p-value**	**$p=5.7x10^{-5}$**	**p=0.008**	**p=0.05**	**p=0.03**	**p=0.002**	
SCN1A	**Feature**	**N/A**	**MTR ExAC v1**	**VEST3**			
	β	**-5.16**	**-2.69**	**8.98**			
	**p-value**	**$p=2.7x10^{-8}$**	**$p=1.3x10^{-6}$**	**$p=3.3x10^{-20}$**			
SCN2A	**Feature**	**N/A**	**MTR ExAC v1**	**FATHMM**	**VEST3**	**PolyPhen-2 HumDiv**	
	β	**-35.33**	**-2.97**	**31.98**	**3.68**	**3.00**	
	**p-value**	**p=0.02**	**$p=1.9x10^{-4}$**	**p=0.04**	**p=0.002**	**p=0.02**	
SCN8A	**Feature**	**N/A**	**MTR ExAC v1**	**VEST3**			
	β	**-3.25**	**-5.40**	**6.91**			
	**p-value**	**p=0.01**	**$p=2.8x10^{-8}$**	**$p=1.3x10^{-4}$**			

P-values for each X_i tests the null hypothesis that the coefficient of that feature has no effect.
MTR = Missense Tolerance Ratio.

Supplemental Data S1: Review of segregation support accompanying missense variants reported in the eleven genes among ClinVar and/or HGMD® catalogs

Separate data file submission.

Supplemental Data S2: Summary of patient-ascertained pathogenic missense variants found to overlap with population reference cohorts

Separate data file submission.

Supplemental Data S3: ExAC v1 and ExAC v2 MTR estimates for all windows across the eleven epilepsy genes

Separate data file submission.

Supplemental Data S4: Contains the full set of annotations for every possible missense variant in the canonical transcript of each of the eleven epilepsy genes

Separate data file submission.
